# Late Embryogenesis Abundant (LEA)5 Regulates Translation in Mitochondria and Chloroplasts to Enhance Growth and Stress Tolerance

**DOI:** 10.3389/fpls.2022.875799

**Published:** 2022-06-16

**Authors:** Barbara Karpinska, Nurhayati Razak, Daniel S. Shaw, William Plumb, Eveline Van De Slijke, Jennifer Stephens, Geert De Jaeger, Monika W. Murcha, Christine H. Foyer

**Affiliations:** ^1^School of Biosciences, College of Life and Environmental Sciences, University of Birmingham, Birmingham, United Kingdom; ^2^Centre for Plant Sciences, School of Biology, Faculty of Biological Sciences, University of Leeds, Leeds, United Kingdom; ^3^Department of Plant Biotechnology and Bioinformatics, Ghent University, Ghent, Belgium; ^4^VIB Center for Plant Systems Biology, Ghent, Belgium; ^5^Cell and Molecular Sciences, The James Hutton Institute, Dundee, United Kingdom; ^6^School of Molecular Sciences, Perth, WA, Australia

**Keywords:** respiration, mitochondria, chloroplasts, translation, signaling

## Abstract

The late embryogenesis abundant (LEA)5 protein is predominantly expressed in Arabidopsis leaves in the dark, the levels of *LEA5* transcripts decreasing rapidly upon illumination. LEA5 is important in plant responses to environmental stresses but the mechanisms involved have not been elucidated. We therefore explored LEA5 functions in Arabidopsis mutants (*lea5*) and transgenic Arabidopsis plants constitutively expressing LEA5 (OEX 2-5), as well as in transgenic barley lines expressing the Arabidopsis *LEA5* gene. The OEX 2-5 plants grew better than controls and *lea5* mutants in the presence of the prooxidants methyl viologen and menadione. Confocal microscopy of Arabidopsis mesophyll protoplasts expressing a LEA5-YFP fusion protein demonstrated that LEA5 could be localized to chloroplasts as well as mitochondria in Arabidopsis protoplasts. Tandem affinity purification (TAP) analysis revealed LEA5 interacts with the chloroplast DEAD-box ATP-dependent RNA helicase 22 (RH22) in Arabidopsis cells. Split YFP analysis confirmed the interaction between RH22 and LEA5 in chloroplasts. The abundance of translated protein products in chloroplasts was decreased in transgenic Arabidopsis plants and increased in *lea5* knockout mutants. Conversely, the abundance of translated mitochondrial protein products was increased in OEX 2-5 plants and decreased in *lea5* mutants. Mitochondrial electron transport rates were higher in the OEX 2-5 plants than the wild type. The transformed barley lines expressing the Arabidopsis LEA5 had increased seed yields, but they showed a greater drought-induced inhibition of photosynthesis than controls. Taken together, these data demonstrate that LEA5 regulates organellar translation, in order to enhance respiration relative to photosynthesis in response to stress.

## Introduction

Late embryogenesis abundant (LEA) proteins are a diverse family of small and hydrophilic polypeptides (10–30 kD) that protect plant tissues against a wide range of abiotic stresses by acting as molecular chaperones that bind to enzymes, membranes, DNA/RNA, and ROS (Umezawa et al., 2006; Tunnacliffe and Wise, 2007; Lan et al., 2013; Lee et al., 2013). They accumulate at the later stages of embryogenesis during seed desiccation and they are also abundant in vegetative organs (Shao et al., 2005; Lan et al., 2013; Hatanaka et al., 2015). LEA proteins, which are rich in alanine, glycine and serine, are characterized by different conserved sequence motifs composed of repeating arrangements of hydrophilic amino acids. They are intrinsically disordered in the fully hydrated state, but they form α-helical ordered structures upon exposure to stresses such as drought. For example, overexpression of a melon Y3SK2-type LEA, which belongs to the dehydrin (DHNs) group, in tobacco conferred drought and salt tolerance (Poku et al., 2020).

LEA proteins are found in many subcellular compartments, particularly mitochondria, chloroplasts, and the cytosol (Huang et al., 2016; Magwanga et al., 2018). Phytohormones such as abscisic acid (ABA) and stress-driven signals regulate LEA expression (Artur et al., 2019). For example, LEA5 (also called LEA 38, At4g02380), which belongs to an anomalous LEA protein group (type 3) in Arabidopsis (Avelange-Macherel et al., 2018), is expressed in response to ABA and oxidative stress (Mowla et al., 2006). In addition to transcriptional regulation, LEA proteins undergo translational or posttranslational modifications, reflecting the complexity of the regulation of these proteins (Sun et al., 2013; Verdier et al., 2013).

The LEA type 3 family are characterized by the presence of a tryptophan-containing W-motif (Singh and Graether, 2020). They are small (11.1 kD) in size, having an 11-amino acid sequence (TAQAAKEKAGE) repeated 13-times and they are disordered in solution (Jaspard et al., 2012). Over 450 LEA3 proteins are found in the Phytozome v13 database (Singh and Graether, 2020). LEA5 is paralogous to LEA2 (At1g02820). However, LEA5 has an additional 11 residue-long stretch of amino acids (AQGSVSSGGRS), which is required for mitochondrial targeting (Avelange-Macherel et al., 2018).

Earlier reports had shown that LEA5 fulfills functions in plant growth and abiotic stress tolerance (Mowla et al., 2006; MohdSalleh et al., 2012). Moreover, LEA5 was identified in a genome-wide association study (GWAS) seeking to identify Arabidopsis loci involved in local geographic adaptation, suggesting that LEA5 plays an important role in adaptations to environmental conditions (Fournier-Level et al., 2011). A single nucleotide polymorphism (SNP) at position 1046738 (A/T), which is in the first exon of *AtLEA5*, was associated with local adaptation to temperature stress in Finland (Fournier-Level et al., 2011). The T allele, which is the less frequent allele across Arabidopsis ecotypes, was associated with poor survival in Finland, in relation to low-temperature stress. It is likely therefore that LEA5 contributes to stress tolerance under field conditions (Fournier-Level et al., 2011).

In the absence of stress, LEA5 is expressed in leaves only in the dark and the levels of *LEA5* transcripts decline rapidly when leaves are exposed to light (Mowla et al., 2006). The dark-induced expression of LEA5 does not appear to be subject to circadian regulation. LEA5 expression is triggered in the light upon exposure to biotic or abiotic stresses or stress hormones (Mowla et al., 2006; MohdSalleh et al., 2012). Transgenic Arabidopsis lines overexpressing *AtLEA5* had similar photosynthesis rates to the wild type plants under optimal growth conditions. Interestingly, photosynthetic CO_2_ assimilation rates were more severely inhibited by drought in the LEA5 overexpression lines than the wild type controls (Mowla et al., 2006). However, LEA5 overexpression had a beneficial impact on plant growth even under drought stress (Mowla et al., 2006). The following studies were performed to explore the mechanisms by which LEA5 regulates plant growth, development and stress tolerance in model, and crop plants.

## Materials and Methods

### Plant Material and Growth Conditions

Seeds of wild type Arabidopsis (Col-0), a transgenic line overexpressing LEA5 (OEX2-5; Mowla et al., 2006) and T-DNA insertion mutant lines (*lea5*) were used in the following studies. The *lea5* T-DNA SALK line (SALK_099663), which has an insertion in the second exon of AT4G02380, was obtained from TAIR. A T-DNA insertion *rh22* mutant line with a T-DNA insertion in sixth intron of the At1g59990 gene, was also used in these studies. The seeds of this line were a gift from Professor Masatake Kanai (Kanai et al., 2013). Genotyping was performed as recommended by the T-DNA primer design website http://signal.salk.edu/tdnaprimers.2.html.

Arabidopsis seeds were vapor-sterilized using commercial bleach with concentrated HCL for 2 h, then sown onto Petri dishes containing 0.5x Murashige and Skoog (MS) basal salts, 1% agar, pH 5.7 and sealed with micro pore tape and later stratified for 2 days at 4°C.

The plates were then either transferred to controlled environment chambers at 20°C and grown under a long photoperiod (16 h light and 8 h dark), with a light intensity of 150 μmol m^–2^ s^–1^ and relative humidity (70%) for 14 days, or germinating seeds were transplanted to soil and grown in controlled environment chambers, under growth conditions, as described above.

Seeds of wild type barley (*Hordeum vulgare* CV Golden Promise) and transgenic barley lines expressing the Arabidopsis LEA5 sequence (9.1, 10.1, and 11.2) were sterilized using 10% (*^v^*/_*V*_) bleach for 15 min, followed by 3 washes (each 10 min) with 80% of ethanol and 5 washes with 50 ml of sterilized water in a laminar flow cabinet. Plants were grown on compost under controlled environment conditions with a 16 h light/8 h dark photoperiod regime (400 μmol m^–2^ s^–1^).

### Oxidative Stress Treatments

For these studies, seeds were sown onto Petri dishes, as described above, except that the plates contained either no added oxidants (Control), or methyl viologen (0.1 μM), or menadione (0.1 mM). Seedlings were grown under a 16 h photoperiod (150 μmol.m^–2^. s^–1^ irradiance) at 22°C ± 2°C for 21 days prior to analysis.

### Drought Treatments

For these studies, seedlings of wild type barley, transgenic lines expressing the Arabidopsis LEA5 sequence (9.1, 10.1, and 11.2) and empty vector and GUS controls were grown for 7 days on soil under well-watered conditions in controlled environments, as described above. Thereafter, half of the plants were maintained under well-watered conditions and half were deprived of water for 7 days. Photosynthesis measurements were then performed.

### Organelle and Protoplast Isolation

Intact chloroplast and mitochondria fractions were isolated from 14-day old plate-grown seedlings according to protocols described by Aronsson and Jarvis (2011) and Murcha and Whelan (2015), respectively. Tape-Arabidopsis Sandwich protocols were used for protoplast isolation and PEG transformation (Wu et al., 2009).

### Measurements of the Integrity of Mitochondrial Fractions

The percentage of intact mitochondria present in isolated fractions was measured via the latency of cytochrome C oxidase (COX) activity in the absence and presence of Triton X-100 using Clark-type oxygen electrode (Neuberger et al., 1982; Shaw et al., 2017).

### Complex II Respiratory Control

Respiratory control at the level of Complex 1 was measured as described by Shaw et al. (2017). Firstly, mitochondria were added to a 1 ml liquid phase oxygen electrode chamber at final concentration 100 μg/ml. Pyruvate, thiamine pyrophosphate (TPP) and malate were added to the final concentrations of 2, 3, and 2 mM, respectively. After 2 min 20 μl of 100 mM ADP was added and after a further 2 min the reaction was stopped with the addition of 2 μg/ml oligomycin. Then 0.5 μM carbonyl cyanide p-trifluoromethoxyphenylhydrazone (FCCP) was added.

### Complex II Respiratory Control

Respiratory control at the level of Complex II was measured as described by Shaw et al. (2017). Mitochondria were first added to a 1 ml liquid phase oxygen electrode chamber at final concentration 100 μg/ml. Then 10 mM succinate was added to the chamber. After 1 min, 0.5 μM rotenone was added to inhibit complex I activity. Thereafter, 20 μl of 100 mM ADP was added followed 2 μg/ml oligomycin and 0.5 μM FCCP.

### Labeling of Mitochondrial and Chloroplast Translation Products With [^35^S] Met “in Organello”

Intact organelle fractions were suspended in assay buffer, prior to analysis. Aliquots (60–100 μg protein) were added to buffer containing the mixture of 19 amino acids. Reactions were started by the addition of 225 uCi [35S] methionine (1,000 Ci/mM) and incubated at 25°C with constant rotatory shaking for 60 min. Mitochondrial fractions were incubated in the dark, while chloroplast fractions were incubated at low light conditions (100 μmol m^–2^ s^–1^). Reactions were stopped by the addition of cold buffer containing non-labeled methionine. Proteins were separated on 14% SDS-PAGE gels. The incorporation of [35S]-Met into protein bands was determined by soaking the gels in ENLIGHTNING™ Rapid Autoradiography Enhancer. After drying, the gels were analyzed using a phosphorimager (Bio Rad) at high resolution for 48 h.

### PCR Reactions

Reverse transcription of 1 μg of RNA aliquots into cDNA was performed using the QuantiTect Reverse Transcription Kit (Qiagen). Thereafter, qPCR was performed using QuantiFast SYBR Green PCR kit (Qiagen) in the presence of 0.5 μM primers in a CFX96 thermocycler (Biorad, Hercules, CA, United States) following the manufacturer’s instructions. The two-step cycling protocol was programmed as follows: incubation at 95°C for 5 min; 40 cycles of amplification comprised of 95°C for 10 s, 60°C for 30 s and 72°C for 30 s. The mean value of three replicates was normalized using actin 11 as internal controls. All amplifications for the cloning purposes were done with Thermo Scientific Phusion High-Fidelity DNA Polymerase according to manufacturer’s instructions. All primer sequences are listed in Supplementary Table 2.

### Confocal Microscopy

Intact leaf mesophyll protoplasts were prepared from 14-day old Arabidopsis seedlings expressing the LEA5-YFP protein. Images of these protoplasts or 5 days old plate-grown seedlings expressing the LEA5-YFP protein were prepared using a Zeiss LSM700 laser scanning confocal microscope using 20×/0.8 Plan-Apochromat, 40×/1.2 W C-Apochromat or 63 × /1.4 Oil Plan-Apochromat in multi-track channel mode. Three fluorescent proteins were excited with the following excitation wavelengths: GFP with 488 nm, YFP with 514 nm, and RFP with 651 nm. Mitochondria were labeled with red-fluorescent dye, Mitotracker Red CMXRos (Thermo Fisher Scientific) after excitation at 578 nm. Images were collected using LP emission filters 500–710 nm and processed using Zeiss ZEN 2011 (black edition).

### Preparation of the Late Embryogenesis Abundant 5 and RNA Helicase 22 Constructs

#### Cloning Late Embryogenesis Abundant 5-YFP Fusion Protein Constructs

PCR products containing LEA5 joined in frame to YFP were cloned into pDONR207 using the Gateway BP recombination reaction (Invitrogen). After verification of the nucleotide sequence of the fragment, Gateway LR recombination reaction (Invitrogen) was used to transfer the LEA5-YFP into pBRACT214 vector containing ubiquitin promoter and hygromycin B resistance for positive selection of transformed plants.

#### Cloning for Split YFP Experiments

The DEA (D/H)-box RNA helicase 22 (AT1G59990) and LEA5 sequences were cloned without a STOP codon into the pDONR201 Gateway vector and then transferred to the split YFP vectors (pDH51-GW-YFPn and pDH51-GW-YFPc).

#### Cloning of Constructs Containing Late Embryogenesis Abundant 5 Used for Tandem Affinity Purification Experiments

Forward primers contained the Kozak sequence, while the reverse primers were designed without a stop codon in order to perform C-terminal fusion. Fragments were then cloned in pDONR201 and used to produce constructs for TAP tagging. All cloning steps were performed using Gateway Technology.

### Split YFP Experiments

A range of constructs (RH22-YFPn and LEA5-YFPc or RH22-YFPc and LEA5-YFPn) were designed to study protein-protein interactions and transiently expressed in Arabidopsis mesophyll protoplasts. Homologous co-transfections using either LEA5-YFPn and LEA5-YFPc or RH22-YFPn and RH22-YFPc were performed as controls. For these studies protoplasts were co-transfected with 10 μg of plasmid mixture the appropriate constructs, as described by Wu et al. (2009). Images of the interactions were prepared using confocal microscopy (Horstman et al., 2014).

### Barley Transformation

Barley immature embryos were extracted and co-cultivated with Agrobacterium cells containing the BRACT214_SAG21-YFP for 3 days in the dark (Comadira, 2015). After callus induction and regeneration of shoots on hygromycin selection, putative transgenic plantlets were transferred to soil and grown in the glasshouse. PCR was performed using hygromycin B and Arabidopsis-YFP primers to confirm the presence of transgene. Thirty-six lines were selected as positive. Homozygous seedlings were selected from T3 generation in 0.5x MS plates supplemented with hygromycin B. Three lines with the closest 3:1 ratio (resistant: sensitive) were subsequently chosen; 9.1; 10.1 and 11.2. For controls, barley plants were transformed in parallel with pBRACT214 empty vector and pBRACT204 vector containing the GUS fragment (Comadira, 2015).

### Biomass and Seed Yield Measurements

T3 generation plants, control lines and the wild type were grown to maturity in compost in glasshouses at the University of Leeds and the James Hutton Institute in Scotland under a 16 h/8 h day photoperiod regime with supplemental lighting. The number of fertile tillers were counted and the total seed yield quantified.

### Tandem Affinity Purification

*Arabidopsis* cell suspension cultures (PSB-D) were transformed using a co-cultivation method with direct selection in liquid medium, as described previously (Van Leene et al., 2011). TAP tagging of protein complexes was performed using a GS^rhino^ tag (Van Leene et al., 2015). Aliquots (200 mg) of protein were precipitated. Proteins were then separated on SDS-PAGE as described by Bürckstümmer et al. (2006) and Van Leene et al. (2015). Protein interactors were identified by mass spectrometry using an LTQ Orbitrap Velos mass spectrometer. Proteins with at least two matched high confident peptides were retained for further analysis (Supplementary Table 1). Background proteins were filtered out based on the frequency of occurrence in the co-purified proteins in a large dataset containing 543 TAP experiments using 115 different baits (Van Leene et al., 2015).

### Northern Blot Analysis

Samples (5 μg of total RNA) were denatured in 1 volume of NorthernMax™-Gly Sample Loading Dye for 30 min at 50°C. They were then separated on a 1.2% (w/v) agarose gel, blotted to positively charged nylon membrane by capillary blotting, and then fixed by cross-linking. Hybridization was performed with ^32^P-labeled probes in 5 × SSC, 5 × Denhardt’s solution, and 0.5% SDS at 65°C overnight. The membranes were washed twice in 2 × SSC, 0.1% SDS and once in 1 × SSC, 0.1% SDS at 65°C.

Hybridization probes were prepared by PCR amplification of *23S-4.5S rrn* region (primer sequences listed in Supplementary Table 2). The fragments were separated on 1.5% agarose gels and extracted using QIAquick Gel extraction kit (Qiagen). They were then labeled with 20 μCi [α-32P]dCTP, 3000 Ci/mmol (PerkinElmer) using a “Random primed” DNA labeling kit (Roche).

### Photosynthesis Measurements

Light response curves for photosynthesis were measured using a portable Infrared Gas Analyzer (model LI-6400XT) LI-COR. Measurements were performed at 20°C and a CO2 concentration of (400 μmol mol^–1^) in the leaf chamber. The leaves were exposed to each of the following light intensities: [(0, 50, 250, 500, 750, 1,000, 1,250, 1,500, and 1,750 μmol m^–2^ s^–1^) photosynthetically active radiation (PAR)] allowing the leaves to acclimatize to each irradiance for at least 15 min prior to measurement to allow stabilization of parameters. Measurements were made on 3 plants per line per experiment.

## Results

### The Late Embryogenesis Abundant 5 Protein Plays a Role in Protection Against Oxidative Stress

**FIGURE 1:**
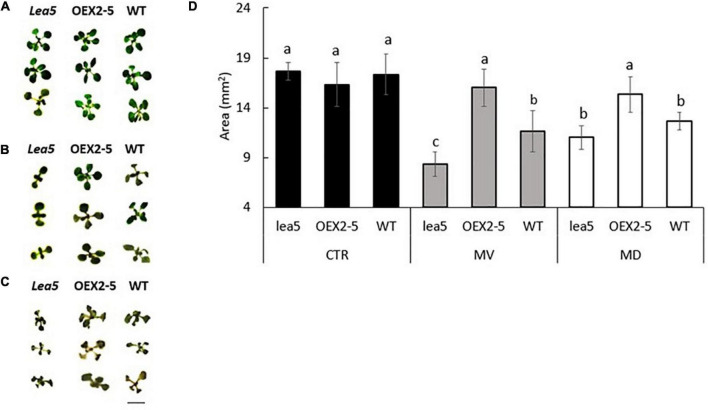
The rosette phenotypes **(A–C)** and growth (determined as rosette area) of wild type Arabidopsis (WT), mutants defective in LEA 5 (*lea5*) and a transgenic line overexpressing the LEA5 protein (OEX2-5) in the absence or presence of the prooxidants menadione and paraquat **(D)**. **(A)** Controls, **(B)** seedlings grown in the presence of 0.1 μM paraquat or **(C)** 0.1 mM menadione. Error bars represent mean +/– standard error. Different letters indicate significance. Statistical analysis was performed using One Way ANOVA and *post hoc* Tukey test. Scale bar is 5 mm.

The *lea5* mutants and the transgenic plants overexpressing the LEA5 protein (OEX2-5) had a similar vegetative growth phenotype to the wild type (Figures 1A,D). The growth of the wild type seedlings was decreased in the presence of the prooxidants menadione (Figures 1B,D). and paraquat (Figures 1C,D). The prooxidant-related decreases in plant growth were greater in the *lea5* mutant seedlings than the wild type (Figures 1B–D). However, decreases in growth caused by the prooxidants were much less pronounced in the OEX2-5 seedlings than the wild type (Figures 1B–D).

### Intracellular Localization of the Late Embryogenesis Abundant 5 Protein

**FIGURE 2:**
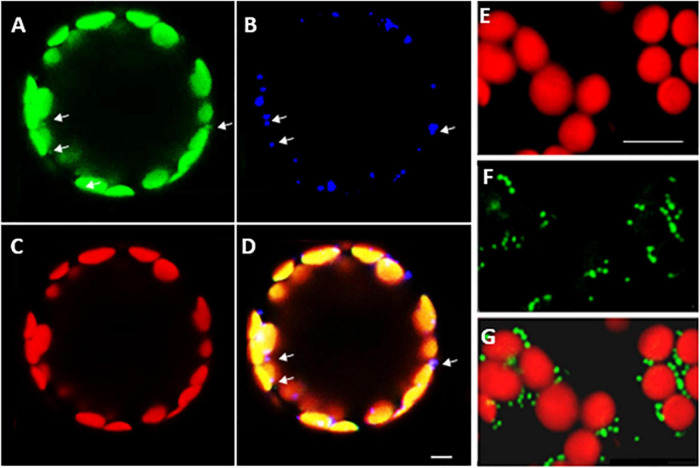
Intracellular localization of LEA5 in mesophyll protoplasts and mesophyll tissue of Arabidopsis leaves. Mesophyll protoplasts **(A–D)** and leaf mesophyll cells **(E–G)** taken from 5 days old *Arabidopsi*s seedlings (T_5_ generation) expressing the LEA5-YFP protein. Green: YFP signal **(A)**, blue: MitoTracker Red CMXRos **(B)**, red: chlorophyll autofluorescence **(C)** and overlay **(D)**. The detection of the LEA5-YFP protein in the mitochondria of the mesophyll cells of 5-day old leaves of Arabidopsis seedlings **(F,G)**. Arrows indicate mitochondria. Scale bar = 10 μm.

Confocal microscopy of Arabidopsis mesophyll protoplasts expressing a LEA5-YFP fusion protein, revealed that the LEA5 protein was localized to the chloroplasts as well as the mitochondria (Figures 2A–D). In contrast, LEA5-YFP protein was only detected in the mitochondria of the mesophyll cells of 5-day old leaves of Arabidopsis seedlings (Figures 2F,G).

### Analysis of the Late Embryogenesis Abundant 5 Interactome

**FIGURE 3:**
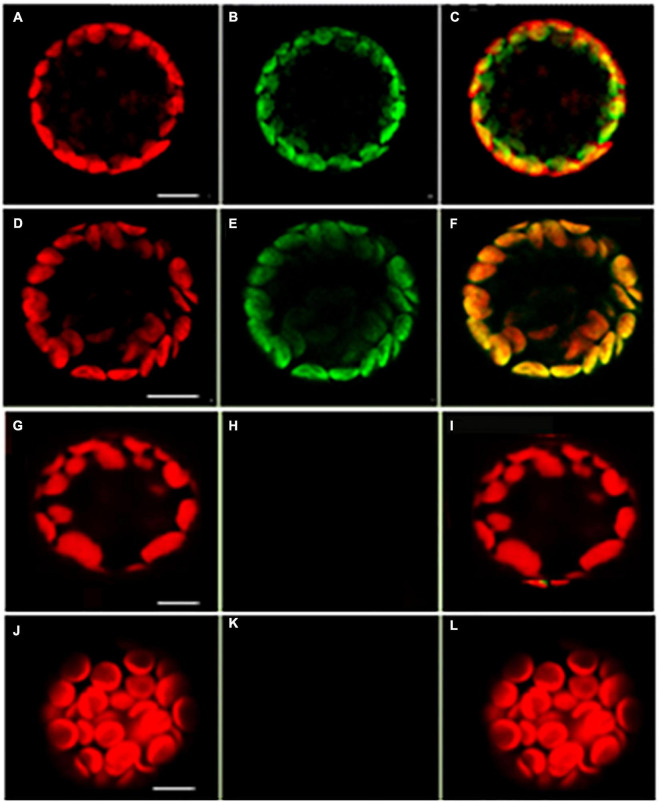
Confocal microscopy images of Arabidopsis mesophyll protoplasts showing interactions between LEA5 and DEA (D/H)-box RNA helicase 22. Protoplasts were co-transfected with LEA5-YFPc and DEA (D/H)-box RNA helicase 22-YFPn **(A–C)** and LEA5-YFPn with DEA (D/H)-box RNA helicase 22-YFPc **(D–F)**. Co-transfection of DEA (D/H)-box RNA helicase 22-YFPn and DEA (D/H)-box RNA helicase 22-YFPc gives no positive signal of interaction **(G–I)**. Overlay of fluorescence signals **(C,F,I,L)**. The interaction between LEA5-YFPn and LEA5-YFPc in mitochondria is demonstrated by co-transfection of LEA5-YFPn and LEA-YFPc **(J–L)**. Scale bar = 10 μm.

A tandem affinity purification (TAP) approach was used to identify protein interaction partners for LEA5. For these studies, a LEA*5-YFP* construct was constitutively expressed in transgenic Arabidopsis ecotype Landsberg *erecta* suspension cultures grown either in the dark (PSB-D) or the light (PSB-L). We characterized the proteins that interacted with *LEA5* in the absence or presence of hydrogen peroxide (H_2_O_2_) in order to determine whether oxidative stress altered the repertoire of LEA5 protein binding partners. Several proteins that interacted with the LEA5 protein in the cultures grown in the absence or presence of H_2_O_2_ were identified (Table 1). The list includes a chloroplast-localized DEA (D/H)-box RNA helicase family protein (RH22; AT1G59990), which is involved in chloroplast ribosome biogenesis and plays a key role in Arabidopsis growth and stress responses (Gu et al., 2014; Liu and Imai, 2018), as well as another DEA(D/H)-box RNA helicase family protein (RH20) and pumilio (PUM) 24, which regulates mRNA degradation and translation repression. Since LEA5 interacted with RH22 in all tagging experiments, we analyzed this interaction further using bimolecular fluorescence complementation (BIFC) assays. This method confirmed the interaction of LEA5 with RH22 specifically in the chloroplasts of Arabidopsis leaf protoplasts (Figure 3).

**TABLE 1:** Tandem affinity purification tagging identification of proteins interacting with LEA5.

Gene accession	Annotation	Dark –H_2_O_2_	Dark +H_2_O_2_	Light –H_2_O_2_	Light +H_2_O_2_
At1g59990	RH22/DEA(D/H)-box helicase family protein 22, regulates ribosome assembly and rRNA processing.	*	*	*	*
At3g16810	PUM24/pumilio 24, mRNA degradation and translation repression	*	*	*	
At1g55150	RH20/DEA(D/H)-box RNA helicase family protein.	*	*		
At2g02100	PDF2.2/low molecular weight cysteine-rich 69		*	*	
At2g36200	P-loop containing nucleoside triphosphate hydrolases superfamily protein		*	*	

*The asterisks denote the presence of an interaction.*

### Late Embryogenesis Abundant 5 Modulates Organellar Translation and Increases Respiratory Electron Transport

**FIGURE 4:**
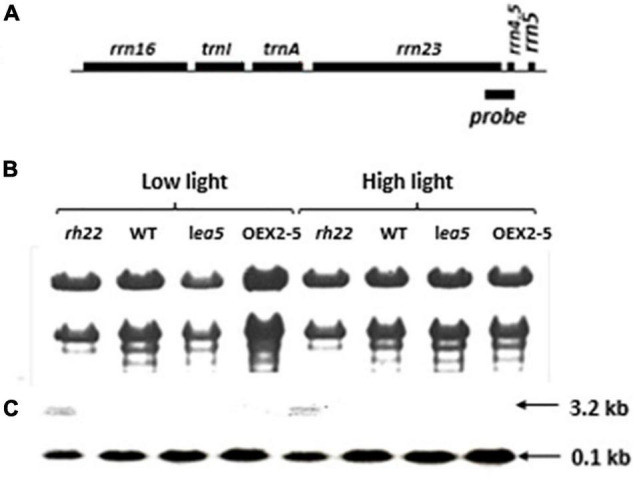
Expression and processing of chloroplast rRNA in Wild type (WT) Arabidopsis, a knockout line defective in DEA (D/H)-box RNA helicase 22 (Δrh22), a knockout line defective in LEA5 (*lea5*) and a transformed Arabidopsis line overexpressing LEA5 (OEX2-5). **(A)** A diagram of the chloroplast rRNA operon showing the location of the probe used for the RNA gel-blot analysis. **(B)** Ethidium bromide staining of glyoxylated RNA separated on 1.2% agarose extracted from plants grown under either low or high light conditions. **(C)** Northern blot analysis.

To explore the functions of LEA5 in organelles, the expression and processing of chloroplast rRNA was measured in wild type Arabidopsis and in mutants lacking RH22 (*rh22*), *lea5* mutants and in plants overexpressing LEA5 (OEX2-5; Figure 4). In contrast to the *rh22* mutant, which showed aberrant chloroplast ribosome processing, the *lea5* mutant and OEX2-5 plants showed similar levels of chloroplast rRNAs to the wild type (Figure 4).

**FIGURE 5:**
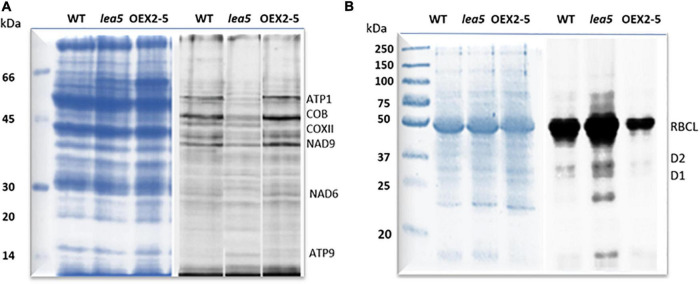
A comparison of mitochondrial and chloroplast translation products in wild type (WT) Arabidopsis, a knockout line defective in LEA5 (*lea5*) and a transformed Arabidopsis line overexpressing LEA5 (OEX2-5). Mitochondrial **(A)** and chloroplast proteins **(B)** were labeled with ^35^S-Met. A shows the ATP synthase subunits, ATP1 and ATP9, the NADH:ubiquinone oxidoreductase (NAD) 7 and 9 subunits, cytochrome b (cob), and cytochrome c oxidase subunit II (COXII). **(B)** Shows labeling in the photosystem II reaction center proteins D1 and D2 proteins and the large subunit of ribuoose-1, 5-bisphosphate carboxylase oxygenase (RBCL).

Finally, organellar translation in chloroplasts and mitochondria was analyzed in wild type, OEX 2-5 and *lea5* by *in organello* translation assays which measures the incorporation [35S] methionine to newly synthesized proteins of isolated organelles (Figure 5). The intensity of labeling for some mitochondrial protein products was seen to be decreased in mitochondria isolated from *lea5* and increased in mitochondria isolated from OEX 2-5 relative to the wild type (Figure 5A). Conversely, the intensity of labeling of some translated chloroplast protein products was observed to increase in chloroplasts isolated from *lea5* mutants and decreased in chloroplasts isolated from OEX 2-5 relative to the wild type (Figure 5B). Measurement of mitochondrial respiration revealed that mitochondria isolated from the OEX 2-5 exhibited significantly higher rates of respiratory electron transport to the cytochrome C oxidase than mitochondria from wild type controls (Table 2).

**TABLE 2:** Respiratory electron transport rates in isolated intact mitochondria from intact wild type Arabidopsis leaves.

A				

Maximal COX activity		Percentage of intact mitochondria		
	
WT	OEX2-5	WT	OEX2-5	
142.9 ± 7.9	166.2 ± 9.8*	85.4 ± 3.2	89.8 ± 1.3	N=24

**B**					

**Genotype**	**CI substrates**	**ADP**	**Oligomycin**	**FCCP**	

WT	23.0 ± 2.5	18.6 ± 1.1	1.0 ± 0.5	20.4 ± 3.8	
OEX2-5	22.0 ± 2.5	22.5 ± 5.2	1.4 ± 0.3	14.0 ± 4.1	N=4

**C**						

	**Succinate**	**Rotenone**	**ADP**	**Oligomycin**	**FCCP**	

WT	23.1 ± 3.4	26.2 ± 3.0	41.0 ± 3.3	26.2 ± 4.6	32.6 ± 3.4	
OEX2-5	22.8 ± 4.2	24.2 ± 2.6	33.4 ± 3.9	27.5 ± 3.5	24.3 ± 4.6	N=15

(A) Maximal respiration rates through complex I (CI) cytochrome C oxidase (COX). COX activity expressed as nmol O_2_ * min^–1^ * mg protein^–1^; (B) electron transport through respiratory complex I (CI) in the absence or presence of ADP, oligomycin or p-triflouromethoxyphenylhydrazone (FCCP); (C) electron transport through respiratory complex II.

### The Expression of *AtLEA5* in Barley Increases Seed Production

**FIGURE 6:**
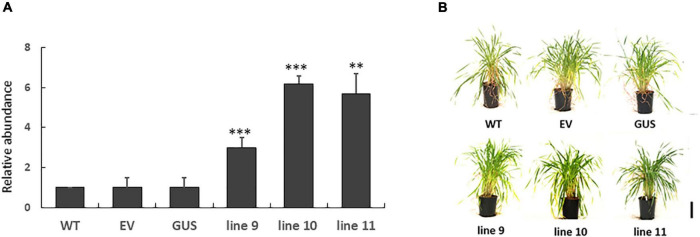
The expression of Arabidopsis *LEA5* in transgenic barley lines (9, 10, 11) relative to controls (WT, EV, GUS) and the growth phenotypes of these lines. The relative abundance of *LEA5* in barley lines expressing the *Arabidopsis* LEA5 sequence (9, 10, 11), control lines expressing empty vector (EV) and beta-glucuronidase (GUS) and WT **(A)**. The shoot phenotypes of the lines at 4 weeks **(B)**. Two asterisks indicates significant differences at *p* < 0.001 and three asterisks indicates significance at *p* < 0.0001.

LEA5 overexpression has previously been reported to increase the growth and flowering of Arabidopsis plants (Mowla et al., 2006; MohdSalleh et al., 2012). We therefore set out to investigate whether LEA5 overexpression also had a positive effect on the growth and productivity of crop species. Barley was selected for these studies because an efficient transformation system is available for this important crop. The Arabidopsis LEA5 protein in barley was characterized for the growth phenotypes, yield parameters and photosynthesis in a large number of independent transgenic lines. Vegetative and reproductive phenotypes were compared in three overexpression lines (9, 10, and 11) that had high levels of *LEA5* expression compared to the wild type barley plants and empty vector and GUS control lines (Figure 6). Total biomass, the number of tillers, flowers, heads numbers, total seed yield per plant and seed yield per tiller were measured in T3 generation plants that had been grown for 6 months under glasshouse conditions (Figure 6 and Table 3). The number of fertile tillers and seeds produced in transformed barley lines (9; 10, and 11) were significantly higher than the controls (Table 3).

**TABLE 3:** Yield parameters in transgenic barley lines expressing LEA5 in comparison to the wild type (WT) and a control barley line expressing beta-glucuronidase (GUS).

	WT	GUS	Line 9	Line 10	Line 11
Number of fertile tillers	12	13	22**	25***	19**
Total seed yield (g)	4.37	4.51	8.09**	8.16**	6.57**
Seed yield per fertile tiller (g)	0.36	0.38	0.67**	0.68**	0.55**

*Data are mean values ± SE (n = 10). Significantly differences according to the Students t-test are indicated by asterisk.*

### The Expression of *AtLEA5* Increases the Drought-Induced Inhibition of Photosynthesis

**FIGURE 7:**
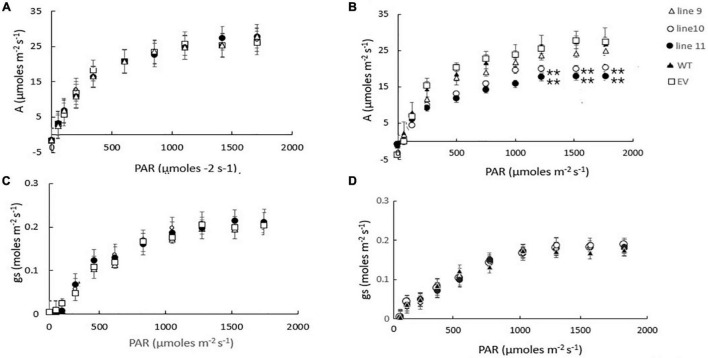
A comparison of the light saturation curves for photosynthesis **(A,B)** and stomatal conductance **(C,D)** in wild type barley plants (WT), transgenic lines expressing the Arabidopsis LEA 5 sequence (lines 9, 10, and 11) and empty vector (EV) controls. Plants were grown for either 14 days under well-watered conditions **(A,C)** or for 7 days under well-watered conditions followed by 7 days without watering **(B,D)**. Asterisks indicate significant differences between the transgenic lines and control as estimated by the Student’s *t*-test (**p < 0.01).

We have previously shown that the expression of AtLEA5 was strongly induced by drought, such that the levels of transcripts correlated with the drought-dependent inhibition of photosynthesis (Mowla et al., 2006). Moreover, Arabidopsis lines LEA5 were more susceptible to drought than the wild type, in terms of the drought-induced inhibition of photosynthesis (Mowla et al., 2006). We therefore next explored the responses of the transformed barley lines (9.1; 10.1, and 11.2) to drought by withholding water for 7 days. Photosynthetic CO2 assimilation was monitored throughout the period of drought course using infrared gas analysis. The AtLEA5-overexpressing barley lines exhibited comparable rates of photosynthesis to the wild type under water-replete conditions (Figure 7A), with comparable rates of stomatal conductance (Figure 7C). The light response curves for photosynthesis were similar in all lines grown under well-watered conditions (Figure 7A). Photosynthetic CO_2_ assimilation rates were decreased in all lines following exposure to drought (Figure 7B). However, the light-saturated rates of photosynthesis were lower in the overexpression lines than controls after 7 days of water deprivation (Figure 7B). Stomatal conductance was decreased in all lines following exposure to drought (Figure 7D). All lines showed similar changes in stomatal conductance in response to increasing irradiance under these conditions (Figure 7D).

### *AtLEA5* Is Localized in the Mitochondria of Transgenic Barley Lines

**FIGURE 8:**
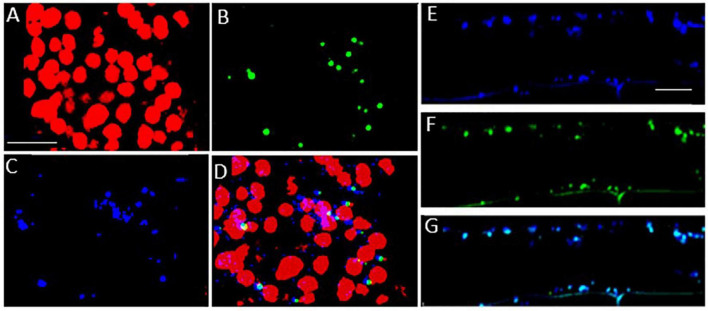
Subcellular localization of the *Arabidopsis* LEA5 in barley leaves and roots. Confocal microscopy of 5-day old transgenic barley leaves **(A–D)** and roots **(E–G)** expressing the 35S-LEA5-YFP fusion protein. Chlorophyll autofluorescence is shown in red **(A,D)**, YFP signal is in green **(B,F)**, MitoTracker Red CMXRos is in blue **(C,E)**. **(D and G)** are merged images of **(A–C,E,F)**, respectively. All scale bars are set to 10 μm.

To investigate the subcellular localization of the Arabidopsis LEA5 protein in the leaves and roots of the transgenic barley lines, lines expressing 35S-LEA5-YFP were generated and analyzed. Confocal microscopy images revealed that the LEA5-YFP protein was present exclusively in the mitochondria of the leaves and roots of the transgenic barley lines (Figure 8).

## Discussion

Chloroplasts and mitochondria are the powerhouses of plant cells that drive cell metabolism, plant growth and development. The photosynthetic and respiratory electron transport chains have the potential to produce reactive oxygen species (ROS) as signals that alter gene expression and facilitate responses to environmental stress (Foyer and Hanke, 2022). Chloroplasts act as environmental sensors, the rates of photosynthesis decreasing and respiration rates increasing in response to abiotic stresses such as drought, when leaf relative water contents decline (Chaves et al., 2002; Xu et al., 2015). Drought inhibits photosynthesis through effects on stomatal aperture, alterations in the stromal redox state, inhibition of enzyme activities and changes in gene expression.

The control of organellar translation contributes to the regulation of plastome and chondrome gene expression in response to developmental, environmental, and physiological cues (Planchard et al., 2018; Zoschke and Bock, 2018). We present evidence that altered expression of LEA5 influences the levels of mitochondrial and chloroplast translation products, as indicated by labeling with ^35^S-Methionine. The *lea5* mutants have less mitochondrial translation products than the wild type and transformed plants overexpressing LEA5 (OEX2-5). Conversely, the transformed plants overexpressing LEA5 (OEX2-5) have less chloroplast translation products than the wild type or *lea5* mutants. The mechanisms by which the LEA5 protein is able to achieve this regulation are unknown but it must occur via interactions with other proteins involved in organellar translation in the mitochondria and chloroplasts. Our studies on the LEA5 protein interactome revealed that LEA5 interacts with the RH22/DEA(D/H)-box helicase family protein 22 (HS3) that regulates ribosome assembly and rRNA processing in the chloroplasts (Kanai et al., 2013).

Organellar protein synthesis is performed on bacteria-like 70S ribosomes, which are composed of a small 30S and a large 50S subunit. RNA processing, intron splicing, RNA editing, turnover and translational control are regulated by a variety of nucleus-encoded RNA-binding proteins (RBPs) that are targeted to chloroplasts or mitochondria, where they play essential roles in organellar RNA metabolism. The RBPs include DEAD-box RNA helicases (RHs) that regulate RNA structures and metabolism and abiotic stress responses (Nawaz and Kang, 2017). The *rh22* mutants that lack RH22 clearly show aberrant chloroplast ribosome processing compared to the wild type (Figure 4). However, the *lea5* mutants and OEX2-5 plants showed similar levels of chloroplast rRNAs to the wild type (Figure 4), demonstrating that LEA5 is not involved in pre-rRNA processing in chloroplasts. We therefore explored the physiological roles of LEA5 in organellar translation via incorporation [35S] methionine into newly synthesized proteins (Figure 4). Data are presented showing that the presence of LEA5 increases the abundance of some translated mitochondrial protein products and decreases chloroplast protein products. Moreover, isolated intact mitochondria from the OEX 2-5 plants exhibited higher rates of cytochrome C oxidase activity than the wild type controls (Table 2). Taken together, these findings show that the LEA5 protein is able to modify the abundance of specific translation products in mitochondria and chloroplasts. Specifically, the levels of the photosystem II reaction center proteins D1 and D2 proteins and the large subunit of ribulose-1, 5-bisphosphate carboxylase oxygenase (RBCL) were decreased in the chloroplasts from the OEX 2-5 plants and increased in *lea5* mutant chloroplasts relative to the wild type. These results suggest that presence of the LEA5 protein specifically decreases proteins involved in photosynthetic electron transport and carbon assimilation. This result is surprising given that the LEA5 protein is targeted to mitochondria and not to chloroplasts. We could not detect the LEA5 protein in intact 35S-LEA5-YFP expressing Arabidopsis leaves or the leaves or roots of the transformed barley lines. In all cases the LEA5 protein was detected only in mitochondria. While we were able to detect the LEA5-YFP in the chloroplasts of the mesophyll protoplasts isolated from the Arabidopsis leaves (Figure 2), other authors have reported that the LEA5 protein is only present in the mitochondria of isolated Arabidopsis protoplasts (Candat et al., 2014). Further studies are required to determine whether the LEA5 protein can re-distribute to the chloroplasts as well as mitochondria of certain tissues, under specific developmental or stress conditions. It may be that the LEA5 protein can localize in specialized sensory plastids that have been described in the epidermis and vascular parenchyma. These “sensory” plastids participate in environmental stress sensing and trigger tissue-specific signaling and systemic stress responses (Beltrán et al., 2018). Moreover, accumulating evidence suggests that the protein complement of any given intracellular compartment is not precisely fixed and that some proteins can move between compartments in response to metabolic or environmental triggers (Foyer et al., 2020). A recent example is the WHIRLY2 protein that is targeted in mitochondria but can also localize to chloroplasts (Huang et al., 2020). In addition, the LEA5 protein may function in the integration of chloroplast- and mitochondria-derived signals that are processed by the nuclear gene expression system. For example, the nuclear cyclin-dependent kinase E is implicated in the expression of both chloroplast and mitochondrial components in response to limitations in either the chloroplast or mitochondrial electron transport chains or H_2_O_2_ treatment (Blanco et al., 2014; Ng et al., 2014). Similarly, the RADICAL-INDUCED CELL DEATH1 (RCD1) protein integrates oxidative signals emitted by both mitochondria and chloroplasts to suppress ANAC013 and ANAC017 functions (Shapiguzov et al., 2019). The presence of LEA5 in the mitochondria of leaves in the light may result in signaling that modulates chloroplast translation, as well as mitochondrial translation.

Mitochondrial respiration involves five multi-subunit protein complexes, four of which (Complexes I–IV) constitute the mitochondrial respiratory chain that moves electrons from NADH and succinate to oxygen, activating the ATP synthase. The levels of the alpha subunit of the ATP synthase (ATP1) and ATP9, which encodes subunit 9, were higher in the mitochondria from the OEX 2-5 plants and lower in *lea5* mutants. Similarly the levels of the NADH:ubiquinone oxidoreductase (NAD) 7 and 9 subunits of Complex I (CI) were higher in OEX 2-5 the mitochondria from lower in *lea5* mitochondria, as were the levels of mitochondrial cytochrome b (cob), and cytochrome c oxidase subunit II (COXII). These findings suggest that mitochondrial electron transport and oxidative phosphorylation are higher when LEA5 is present in the mitochondria. The LEA5 protein therefore has a positive function in plant mitochondria, support mitochondrial respiration, and ATP synthesis.

The precise mechanisms by which LEA5 regulates chloroplast and mitochondrial ribosomal functions remains to be elucidated. However, the data presented here suggest that LEA binds to organellar proteins that regulate mRNA stability and translation in chloroplasts. In this way, LEA5 is likely to modify the responses of photosynthesis and respiration to environmental stresses such as drought. The expression patterns of LEA5 would support this notion. LEA5 is not normally expressed in leaves in the light (Mowla et al., 2006). However, the expression of LEA5 is triggered in leaves in the light by abiotic stresses such as drought and by stress hormones such as abscisic acid (ABA) and jasmonate (Mowla et al., 2006; MohdSalleh et al., 2012). Stress-induced expression of LEA5 in the light may serve to decrease oxidative pressure in chloroplasts under stress conditions by decreasing the synthesis of essential components of the photosynthetic electron transport chain, particularly D1 and D2, while stimulating respiration to maintain ATP production. Chloroplast translation is regulated in response to light through changes in the redox state of chloroplast components such as NADPH thioredoxin reductase C (NTRC; González et al., 2019). The data presented here suggests that LEA5 may be an additional major player in the regulation of chloroplast translation.

We have previously shown that photosynthesis is decreased to a greater extent in transgenic Arabidopsis lines overexpressing LEA5 than the wild type under drought stress conditions (Mowla et al., 2006). We discussed these findings in terms of the decreased stress-induced oxidative load that would occur as a consequence of an increased inhibition of photosynthesis in the transformed lines under these conditions (Mowla et al., 2006). The expression of *AtLEA5* is induced in leaves in the light by oxidants and by oxidative stress. Moreover, as shown in Figure 1, overexpression of *AtLEA5* enhances tolerance to oxidate stress. One might predict therefore that overexpression of *AtLEA5* would increase photosynthesis rates in plants subjected to stresses such as drought. However, the opposite effect was observed both in Arabidopsis (Mowla et al., 2006) and in barley, as shown in Figure 7. The data presented in Figure 7B clearly demonstrate that drought-induced inhibition of photosynthetic carbon assimilation is increased when the Arabidopsis LEA5 protein is constitutively expressed in barley (Figure 7B). While photosynthetic CO_2_ assimilation rates were comparable in all lines in the absence of stress (Figure 7A), the light-saturated rates of photosynthesis were significantly lower in barley lines 9.1; 10.1, and 11.2 under drought stress conditions than controls (Figure 7B). Since stomatal conductance was decreased to a similar extent in all lines in response to drought (Figure 7D), we conclude that overexpression of *AtLEA5* in barley has no effect on stomatal regulation and hence it exerts effects directly in the photosynthetic mesophyll cells. Taken together, findings suggest that the expression of the Arabidopsis LEA5 protein limits chloroplast translation when the barley plants are exposed to drought and that this in turn has a negative impact on photosynthesis rates. However, the concurrent stimulation of mitochondrial translation and respiration rates must be sufficient to offset and compensate for any restrictions on metabolism caused by impaired chloroplast translation. Moreover, the LEA5-dependent increases in mitochondrial translation and respiration are likely to underpin the greater seed yields observed in the transgenic barley lines overexpressing LEA5 compared to controls (Figure 6).

The Arabidopsis LEA5 protein was localized in the mitochondria of the leaves and roots of the transgenic barley lines expressing 35S-LEA5-YFP. While the protein sequence contains a putative chloroplast transit peptide (Emanuelsson et al., 2000), it is not clear whether or how AtLEA5 can localize to plastids. We conclude that the improved yields observed in the barley lines expressing the Arabidopsis LEA5 protein are related to improved regulation of organellar translation in these lines, particularly under stress conditions. These data not only provide new evidence of LEA5 functions in plants, but they also shed new light on the factors that regulate translation in order to facilitate energy and metabolite homeostasis.

## Data Availability Statement

The original contributions presented in the study are included in the article/Supplementary Material, further inquiries can be directed to the corresponding author/s.

## Author Contributions

CF, MM, and GD planned the experiments. BK, JS, NR, and DSS undertook the experimental work and data analysis. BK produced the figures. CF wrote the manuscript. All authors contributed to the article and approved the submitted version.

## Conflict of Interest

The authors declare that the research was conducted in the absence of any commercial or financial relationships that could be construed as a potential conflict of interest.

## Publisher’s Note

All claims expressed in this article are solely those of the authors and do not necessarily represent those of their affiliated organizations, or those of the publisher, the editors and the reviewers. Any product that may be evaluated in this article, or claim that may be made by its manufacturer, is not guaranteed or endorsed by the publisher.
