# Supplementary material for: Late Embryogenesis Abundant (LEA)5 Regulates Translation in Mitochondria and Chloroplasts to Enhance Growth and Stress Tolerance
Source: Front Plant Sci. 2022 Jun 16;13:875799. doi: 10.3389/fpls.2022.875799 (PMC9244843; doi:10.3389/fpls.2022.875799)
Supplement: Supplementary file 1 [file Table_1.pdf]

Table S1: Protein Identification details obtained with the LTQ Orbitrap Velos (Thermo Fisher Scientific) and Mascot Distiller

prot_score: protein score; **prot_mass**: protein mass; **prot_matches_sig:** number of assigned peptide matches above thres to 10, where 1 is the best match; **pep_isbold**: peptide is in bold red (Red and bold typefaces are used to highlight the most scoring protein containing that match. This means that protein hits with many peptide matches that are both bold and red **pep_exp_z**: observed peptide charge state; **pep_calc_mr**: calculated relative molecular mass; **pep_delta**: difference (error) for the peptide match (The number of times we would expect to obtain an equal or higher score, purely by chance. The lov in the peptide

Exp	ID number	Class	prot_acc	prot_desc	prot_score
Dark-1	1	Background	AT5G62690	Symbols: TUB2 | tubulin beta chain 2 | chr5:251	1637
Dark-1	1	Background	AT5G62690	Symbols: TUB2 | tubulin beta chain 2 | chr5:251	1637
Dark-1	1	Background	AT5G62690	Symbols: TUB2 | tubulin beta chain 2 | chr5:251	1637
Dark-1	1	Background	AT5G62690	Symbols: TUB2 | tubulin beta chain 2 | chr5:251	1637
Dark-1	1	Background	AT5G62690	Symbols: TUB2 | tubulin beta chain 2 | chr5:251	1637
Dark-1	1	Background	AT5G62690	Symbols: TUB2 | tubulin beta chain 2 | chr5:251	1637
Dark-1	1	Background	AT5G62690	Symbols: TUB2 | tubulin beta chain 2 | chr5:251	1637
Dark-1	1	Background	AT5G62690	Symbols: TUB2 | tubulin beta chain 2 | chr5:251	1637
Dark-1	1	Background	AT5G62690	Symbols: TUB2 | tubulin beta chain 2 | chr5:251	1637
Dark-1	1	Background	AT5G62690	Symbols: TUB2 | tubulin beta chain 2 | chr5:251	1637
Dark-1	1	Background	AT5G62690	Symbols: TUB2 | tubulin beta chain 2 | chr5:251	1637
Dark-1	1	Background	AT5G62690	Symbols: TUB2 | tubulin beta chain 2 | chr5:251	1637
Dark-1	1	Background	AT5G62690	Symbols: TUB2 | tubulin beta chain 2 | chr5:251	1637
Dark-1	1	Background	AT5G62690	Symbols: TUB2 | tubulin beta chain 2 | chr5:251	1637
Dark-1	1	Background	AT5G62690	Symbols: TUB2 | tubulin beta chain 2 | chr5:251	1637
Dark-1	1	Background	AT5G62690	Symbols: TUB2 | tubulin beta chain 2 | chr5:251	1637
Dark-1	1	Background	AT5G62690	Symbols: TUB2 | tubulin beta chain 2 | chr5:251	1637
Dark-1	1	Background	AT5G62690	Symbols: TUB2 | tubulin beta chain 2 | chr5:251	1637
Dark-1	1	Background	AT5G62690	Symbols: TUB2 | tubulin beta chain 2 | chr5:251	1637
Dark-1	1	Background	AT5G62690	Symbols: TUB2 | tubulin beta chain 2 | chr5:251	1637
Dark-1	1	Background	AT5G62690	Symbols: TUB2 | tubulin beta chain 2 | chr5:251	1637
Dark-1	1	Background	AT5G62690	Symbols: TUB2 | tubulin beta chain 2 | chr5:251	1637
Dark-1	2	Background	AT5G44340	Symbols: TUB4 | tubulin beta chain 4 | chr5:178	1575
Dark-1	2	Background	AT5G44340	Symbols: TUB4 | tubulin beta chain 4 | chr5:178	1575
Dark-1	2	Background	AT5G44340	Symbols: TUB4 | tubulin beta chain 4 | chr5:178	1575
Dark-1	2	Background	AT5G44340	Symbols: TUB4 | tubulin beta chain 4 | chr5:178	1575
Dark-1	2	Background	AT5G44340	Symbols: TUB4 | tubulin beta chain 4 | chr5:178	1575
Dark-1	2	Background	AT5G44340	Symbols: TUB4 | tubulin beta chain 4 | chr5:178	1575
Dark-1	2	Background	AT5G44340	Symbols: TUB4 | tubulin beta chain 4 | chr5:178	1575
Dark-1	2	Background	AT5G44340	Symbols: TUB4 | tubulin beta chain 4 | chr5:178	1575
Dark-1	2	Background	AT5G44340	Symbols: TUB4 | tubulin beta chain 4 | chr5:178	1575
Dark-1	2	Background	AT5G44340	Symbols: TUB4 | tubulin beta chain 4 | chr5:178	1575
Dark-1	2	Background	AT5G44340	Symbols: TUB4 | tubulin beta chain 4 | chr5:178	1575
Dark-1	2	Background	AT5G44340	Symbols: TUB4 | tubulin beta chain 4 | chr5:178	1575
Dark-1	2	Background	AT5G44340	Symbols: TUB4 | tubulin beta chain 4 | chr5:178	1575
Dark-1	2	Background	AT5G44340	Symbols: TUB4 | tubulin beta chain 4 | chr5:178	1575
Dark-1	2	Background	AT5G44340	Symbols: TUB4 | tubulin beta chain 4 | chr5:178	1575
Dark-1	2	Background	AT5G44340	Symbols: TUB4 | tubulin beta chain 4 | chr5:178	1575
Dark-1	2	Background	AT5G44340	Symbols: TUB4 | tubulin beta chain 4 | chr5:178	1575
Dark-1	2	Background	AT5G44340	Symbols: TUB4 | tubulin beta chain 4 | chr5:178	1575
Dark-1	2	Background	AT5G44340	Symbols: TUB4 | tubulin beta chain 4 | chr5:178	1575
Dark-1	2	Background	AT5G44340	Symbols: TUB4 | tubulin beta chain 4 | chr5:178	1575
Dark-1	3	Background	AT5G23860	Symbols: TUB8 | tubulin beta 8 | chr5:8042962-	1470
Dark-1	3	Background	AT5G23860	Symbols: TUB8 | tubulin beta 8 | chr5:8042962-	1470
Dark-1	3	Background	AT5G23860	Symbols: TUB8 | tubulin beta 8 | chr5:8042962-	1470
Dark-1	3	Background	AT5G23860	Symbols: TUB8 | tubulin beta 8 | chr5:8042962-	1470
Dark-1	3	Background	AT5G23860	Symbols: TUB8 | tubulin beta 8 | chr5:8042962-	1470
Dark-1	3	Background	AT5G23860	Symbols: TUB8 | tubulin beta 8 | chr5:8042962-	1470

Dark-1	3	Background	AT5G23860	Symbols: TUB8 | tubulin beta 8 | chr5:8042962-	1470
Dark-1	3	Background	AT5G23860	Symbols: TUB8 | tubulin beta 8 | chr5:8042962-	1470
Dark-1	3	Background	AT5G23860	Symbols: TUB8 | tubulin beta 8 | chr5:8042962-	1470
Dark-1	3	Background	AT5G23860	Symbols: TUB8 | tubulin beta 8 | chr5:8042962-	1470
Dark-1	3	Background	AT5G23860	Symbols: TUB8 | tubulin beta 8 | chr5:8042962-	1470
Dark-1	3	Background	AT5G23860	Symbols: TUB8 | tubulin beta 8 | chr5:8042962-	1470
Dark-1	3	Background	AT5G23860	Symbols: TUB8 | tubulin beta 8 | chr5:8042962-	1470
Dark-1	3	Background	AT5G23860	Symbols: TUB8 | tubulin beta 8 | chr5:8042962-	1470
Dark-1	3	Background	AT5G23860	Symbols: TUB8 | tubulin beta 8 | chr5:8042962-	1470
Dark-1	3	Background	AT5G23860	Symbols: TUB8 | tubulin beta 8 | chr5:8042962-	1470
Dark-1	3	Background	AT5G23860	Symbols: TUB8 | tubulin beta 8 | chr5:8042962-	1470
Dark-1	3	Background	AT5G23860	Symbols: TUB8 | tubulin beta 8 | chr5:8042962-	1470
Dark-1	3	Background	AT5G23860	Symbols: TUB8 | tubulin beta 8 | chr5:8042962-	1470
Dark-1	4	Background	AT2G29550	Symbols: TUB7 | tubulin beta-7 chain | chr2:126	1432
Dark-1	4	Background	AT2G29550	Symbols: TUB7 | tubulin beta-7 chain | chr2:126	1432
Dark-1	4	Background	AT2G29550	Symbols: TUB7 | tubulin beta-7 chain | chr2:126	1432
Dark-1	4	Background	AT2G29550	Symbols: TUB7 | tubulin beta-7 chain | chr2:126	1432
Dark-1	4	Background	AT2G29550	Symbols: TUB7 | tubulin beta-7 chain | chr2:126	1432
Dark-1	4	Background	AT2G29550	Symbols: TUB7 | tubulin beta-7 chain | chr2:126	1432
Dark-1	4	Background	AT2G29550	Symbols: TUB7 | tubulin beta-7 chain | chr2:126	1432
Dark-1	4	Background	AT2G29550	Symbols: TUB7 | tubulin beta-7 chain | chr2:126	1432
Dark-1	4	Background	AT2G29550	Symbols: TUB7 | tubulin beta-7 chain | chr2:126	1432
Dark-1	4	Background	AT2G29550	Symbols: TUB7 | tubulin beta-7 chain | chr2:126	1432
Dark-1	4	Background	AT2G29550	Symbols: TUB7 | tubulin beta-7 chain | chr2:126	1432
Dark-1	4	Background	AT2G29550	Symbols: TUB7 | tubulin beta-7 chain | chr2:126	1432
Dark-1	4	Background	AT2G29550	Symbols: TUB7 | tubulin beta-7 chain | chr2:126	1432
Dark-1	4	Background	AT2G29550	Symbols: TUB7 | tubulin beta-7 chain | chr2:126	1432
Dark-1	4	Background	AT2G29550	Symbols: TUB7 | tubulin beta-7 chain | chr2:126	1432
Dark-1	4	Background	AT2G29550	Symbols: TUB7 | tubulin beta-7 chain | chr2:126	1432
Dark-1	4	Background	AT2G29550	Symbols: TUB7 | tubulin beta-7 chain | chr2:126	1432
Dark-1	4	Background	AT2G29550	Symbols: TUB7 | tubulin beta-7 chain | chr2:126	1432
Dark-1	4	Background	AT2G29550	Symbols: TUB7 | tubulin beta-7 chain | chr2:126	1432
Dark-1	5	Background	AT5G02500	Symbols: HSC70-1, HSP70-1, AT-HSC70-1, HSC70	1152
Dark-1	5	Background	AT5G02500	Symbols: HSC70-1, HSP70-1, AT-HSC70-1, HSC70	1152
Dark-1	5	Background	AT5G02500	Symbols: HSC70-1, HSP70-1, AT-HSC70-1, HSC70	1152
Dark-1	5	Background	AT5G02500	Symbols: HSC70-1, HSP70-1, AT-HSC70-1, HSC70	1152
Dark-1	5	Background	AT5G02500	Symbols: HSC70-1, HSP70-1, AT-HSC70-1, HSC70	1152
Dark-1	5	Background	AT5G02500	Symbols: HSC70-1, HSP70-1, AT-HSC70-1, HSC70	1152
Dark-1	5	Background	AT5G02500	Symbols: HSC70-1, HSP70-1, AT-HSC70-1, HSC70	1152
Dark-1	5	Background	AT5G02500	Symbols: HSC70-1, HSP70-1, AT-HSC70-1, HSC70	1152
Dark-1	5	Background	AT5G02500	Symbols: HSC70-1, HSP70-1, AT-HSC70-1, HSC70	1152
Dark-1	5	Background	AT5G02500	Symbols: HSC70-1, HSP70-1, AT-HSC70-1, HSC70	1152
Dark-1	5	Background	AT5G02500	Symbols: HSC70-1, HSP70-1, AT-HSC70-1, HSC70	1152
Dark-1	5	Background	AT5G02500	Symbols: HSC70-1, HSP70-1, AT-HSC70-1, HSC70	1152
Dark-1	5	Background	AT5G02500	Symbols: HSC70-1, HSP70-1, AT-HSC70-1, HSC70	1152
Dark-1	5	Background	AT5G02500	Symbols: HSC70-1, HSP70-1, AT-HSC70-1, HSC70	1152
Dark-1	5	Background	AT5G02500	Symbols: HSC70-1, HSP70-1, AT-HSC70-1, HSC70	1152
Dark-1	5	Background	AT5G02500	Symbols: HSC70-1, HSP70-1, AT-HSC70-1, HSC70	1152
Dark-1	5	Background	AT5G02500	Symbols: HSC70-1, HSP70-1, AT-HSC70-1, HSC70	1152
Dark-1	5	Background	AT5G02500	Symbols: HSC70-1, HSP70-1, AT-HSC70-1, HSC70	1152
Dark-1	5	Background	AT5G02500	Symbols: HSC70-1, HSP70-1, AT-HSC70-1, HSC70	1152
Dark-1	5	Background	AT5G02500	Symbols: HSC70-1, HSP70-1, AT-HSC70-1, HSC70	1152
Dark-1	5	Background	AT5G02500	Symbols: HSC70-1, HSP70-1, AT-HSC70-1, HSC70	1152
Dark-1	6	Background	AT1G54270	Symbols: EIF4A-2 | eif4a-2 | chr1:20260495-202	1070
Dark-1	6	Background	AT1G54270	Symbols: EIF4A-2 | eif4a-2 | chr1:20260495-202	1070
Dark-1	6	Background	AT1G54270	Symbols: EIF4A-2 | eif4a-2 | chr1:20260495-202	1070
Dark-1	6	Background	AT1G54270	Symbols: EIF4A-2 | eif4a-2 | chr1:20260495-202	1070
Dark-1	6	Background	AT1G54270	Symbols: EIF4A-2 | eif4a-2 | chr1:20260495-202	1070

Dark-1	6	Background	AT1G54270	Symbols: EIF4A-2 | eif4a-2 | chr1:20260495-202	1070
Dark-1	6	Background	AT1G54270	Symbols: EIF4A-2 | eif4a-2 | chr1:20260495-202	1070
Dark-1	6	Background	AT1G54270	Symbols: EIF4A-2 | eif4a-2 | chr1:20260495-202	1070
Dark-1	6	Background	AT1G54270	Symbols: EIF4A-2 | eif4a-2 | chr1:20260495-202	1070
Dark-1	6	Background	AT1G54270	Symbols: EIF4A-2 | eif4a-2 | chr1:20260495-202	1070
Dark-1	6	Background	AT1G54270	Symbols: EIF4A-2 | eif4a-2 | chr1:20260495-202	1070
Dark-1	6	Background	AT1G54270	Symbols: EIF4A-2 | eif4a-2 | chr1:20260495-202	1070
Dark-1	6	Background	AT1G54270	Symbols: EIF4A-2 | eif4a-2 | chr1:20260495-202	1070
Dark-1	6	Background	AT1G54270	Symbols: EIF4A-2 | eif4a-2 | chr1:20260495-202	1070
Dark-1	6	Background	AT1G54270	Symbols: EIF4A-2 | eif4a-2 | chr1:20260495-202	1070
Dark-1	6	Background	AT1G54270	Symbols: EIF4A-2 | eif4a-2 | chr1:20260495-202	1070
Dark-1	6	Background	AT1G54270	Symbols: EIF4A-2 | eif4a-2 | chr1:20260495-202	1070
Dark-1	6	Background	AT1G54270	Symbols: EIF4A-2 | eif4a-2 | chr1:20260495-202	1070
Dark-1	7	Background	AT4G14960	Symbols: TUA6 | Tubulin/FtsZ family protein | cl	1049
Dark-1	7	Background	AT4G14960	Symbols: TUA6 | Tubulin/FtsZ family protein | cl	1049
Dark-1	7	Background	AT4G14960	Symbols: TUA6 | Tubulin/FtsZ family protein | cl	1049
Dark-1	7	Background	AT4G14960	Symbols: TUA6 | Tubulin/FtsZ family protein | cl	1049
Dark-1	7	Background	AT4G14960	Symbols: TUA6 | Tubulin/FtsZ family protein | cl	1049
Dark-1	7	Background	AT4G14960	Symbols: TUA6 | Tubulin/FtsZ family protein | cl	1049
Dark-1	7	Background	AT4G14960	Symbols: TUA6 | Tubulin/FtsZ family protein | cl	1049
Dark-1	7	Background	AT4G14960	Symbols: TUA6 | Tubulin/FtsZ family protein | cl	1049
Dark-1	7	Background	AT4G14960	Symbols: TUA6 | Tubulin/FtsZ family protein | cl	1049
Dark-1	7	Background	AT4G14960	Symbols: TUA6 | Tubulin/FtsZ family protein | cl	1049
Dark-1	7	Background	AT4G14960	Symbols: TUA6 | Tubulin/FtsZ family protein | cl	1049
Dark-1	7	Background	AT4G14960	Symbols: TUA6 | Tubulin/FtsZ family protein | cl	1049
Dark-1	7	Background	AT4G14960	Symbols: TUA6 | Tubulin/FtsZ family protein | cl	1049
Dark-1	7	Background	AT4G14960	Symbols: TUA6 | Tubulin/FtsZ family protein | cl	1049
Dark-1	7	Background	AT4G14960	Symbols: TUA6 | Tubulin/FtsZ family protein | cl	1049
Dark-1	7	Background	AT4G14960	Symbols: TUA6 | Tubulin/FtsZ family protein | cl	1049
Dark-1	8	Background	AT1G04820	Symbols: TUA4, TOR2 | tubulin alpha-4 chain | c	1016
Dark-1	8	Background	AT1G04820	Symbols: TUA4, TOR2 | tubulin alpha-4 chain | c	1016
Dark-1	8	Background	AT1G04820	Symbols: TUA4, TOR2 | tubulin alpha-4 chain | c	1016
Dark-1	8	Background	AT1G04820	Symbols: TUA4, TOR2 | tubulin alpha-4 chain | c	1016
Dark-1	8	Background	AT1G04820	Symbols: TUA4, TOR2 | tubulin alpha-4 chain | c	1016
Dark-1	8	Background	AT1G04820	Symbols: TUA4, TOR2 | tubulin alpha-4 chain | c	1016
Dark-1	8	Background	AT1G04820	Symbols: TUA4, TOR2 | tubulin alpha-4 chain | c	1016
Dark-1	8	Background	AT1G04820	Symbols: TUA4, TOR2 | tubulin alpha-4 chain | c	1016
Dark-1	8	Background	AT1G04820	Symbols: TUA4, TOR2 | tubulin alpha-4 chain | c	1016
Dark-1	8	Background	AT1G04820	Symbols: TUA4, TOR2 | tubulin alpha-4 chain | c	1016
Dark-1	8	Background	AT1G04820	Symbols: TUA4, TOR2 | tubulin alpha-4 chain | c	1016
Dark-1	8	Background	AT1G04820	Symbols: TUA4, TOR2 | tubulin alpha-4 chain | c	1016
Dark-1	8	Background	AT1G04820	Symbols: TUA4, TOR2 | tubulin alpha-4 chain | c	1016
Dark-1	8	Background	AT1G04820	Symbols: TUA4, TOR2 | tubulin alpha-4 chain | c	1016
Dark-1	8	Background	AT1G04820	Symbols: TUA4, TOR2 | tubulin alpha-4 chain | c	1016
Dark-1	8	Background	AT1G04820	Symbols: TUA4, TOR2 | tubulin alpha-4 chain | c	1016
Dark-1	9	Background	AT3G13920	Symbols: EIF4A1, RH4, TIF4A1 | eukaryotic trans	987
Dark-1	9	Background	AT3G13920	Symbols: EIF4A1, RH4, TIF4A1 | eukaryotic trans	987
Dark-1	9	Background	AT3G13920	Symbols: EIF4A1, RH4, TIF4A1 | eukaryotic trans	987
Dark-1	9	Background	AT3G13920	Symbols: EIF4A1, RH4, TIF4A1 | eukaryotic trans	987
Dark-1	9	Background	AT3G13920	Symbols: EIF4A1, RH4, TIF4A1 | eukaryotic trans	987
Dark-1	9	Background	AT3G13920	Symbols: EIF4A1, RH4, TIF4A1 | eukaryotic trans	987
Dark-1	9	Background	AT3G13920	Symbols: EIF4A1, RH4, TIF4A1 | eukaryotic trans	987
Dark-1	9	Background	AT3G13920	Symbols: EIF4A1, RH4, TIF4A1 | eukaryotic trans	987
Dark-1	9	Background	AT3G13920	Symbols: EIF4A1, RH4, TIF4A1 | eukaryotic trans	987
Dark-1	9	Background	AT3G13920	Symbols: EIF4A1, RH4, TIF4A1 | eukaryotic trans	987
Dark-1	9	Background	AT3G13920	Symbols: EIF4A1, RH4, TIF4A1 | eukaryotic trans	987
Dark-1	9	Background	AT3G13920	Symbols: EIF4A1, RH4, TIF4A1 | eukaryotic trans	987
Dark-1	9	Background	AT3G13920	Symbols: EIF4A1, RH4, TIF4A1 | eukaryotic trans	987

Dark-1	9	Background	AT3G13920	Symbols: EIF4A1, RH4, TIF4A1 | eukaryotic trans	987
Dark-1	9	Background	AT3G13920	Symbols: EIF4A1, RH4, TIF4A1 | eukaryotic trans	987
Dark-1	9	Background	AT3G13920	Symbols: EIF4A1, RH4, TIF4A1 | eukaryotic trans	987
Dark-1	9	Background	AT3G13920	Symbols: EIF4A1, RH4, TIF4A1 | eukaryotic trans	987
Dark-1	10	Background	AT3G09440	Symbols: | Heat shock protein 70 (Hsp 70) famil	978
Dark-1	10	Background	AT3G09440	Symbols: | Heat shock protein 70 (Hsp 70) famil	978
Dark-1	10	Background	AT3G09440	Symbols: | Heat shock protein 70 (Hsp 70) famil	978
Dark-1	10	Background	AT3G09440	Symbols: | Heat shock protein 70 (Hsp 70) famil	978
Dark-1	10	Background	AT3G09440	Symbols: | Heat shock protein 70 (Hsp 70) famil	978
Dark-1	10	Background	AT3G09440	Symbols: | Heat shock protein 70 (Hsp 70) famil	978
Dark-1	10	Background	AT3G09440	Symbols: | Heat shock protein 70 (Hsp 70) famil	978
Dark-1	10	Background	AT3G09440	Symbols: | Heat shock protein 70 (Hsp 70) famil	978
Dark-1	10	Background	AT3G09440	Symbols: | Heat shock protein 70 (Hsp 70) famil	978
Dark-1	10	Background	AT3G09440	Symbols: | Heat shock protein 70 (Hsp 70) famil	978
Dark-1	10	Background	AT3G09440	Symbols: | Heat shock protein 70 (Hsp 70) famil	978
Dark-1	10	Background	AT3G09440	Symbols: | Heat shock protein 70 (Hsp 70) famil	978
Dark-1	10	Background	AT3G09440	Symbols: | Heat shock protein 70 (Hsp 70) famil	978
Dark-1	10	Background	AT3G09440	Symbols: | Heat shock protein 70 (Hsp 70) famil	978
Dark-1	10	Background	AT3G09440	Symbols: | Heat shock protein 70 (Hsp 70) famil	978
Dark-1	10	Background	AT3G09440	Symbols: | Heat shock protein 70 (Hsp 70) famil	978
Dark-1	10	Background	AT3G09440	Symbols: | Heat shock protein 70 (Hsp 70) famil	978
Dark-1	10	Background	AT3G09440	Symbols: | Heat shock protein 70 (Hsp 70) famil	978
Dark-1	10	Background	AT3G09440	Symbols: | Heat shock protein 70 (Hsp 70) famil	978
Dark-1	10	Background	AT3G09440	Symbols: | Heat shock protein 70 (Hsp 70) famil	978
Dark-1	11	Background	AT5G35360	Symbols: CAC2 | acetyl Co-enzyme a carboxylase	847
Dark-1	11	Background	AT5G35360	Symbols: CAC2 | acetyl Co-enzyme a carboxylase	847
Dark-1	11	Background	AT5G35360	Symbols: CAC2 | acetyl Co-enzyme a carboxylase	847
Dark-1	11	Background	AT5G35360	Symbols: CAC2 | acetyl Co-enzyme a carboxylase	847
Dark-1	11	Background	AT5G35360	Symbols: CAC2 | acetyl Co-enzyme a carboxylase	847
Dark-1	11	Background	AT5G35360	Symbols: CAC2 | acetyl Co-enzyme a carboxylase	847
Dark-1	11	Background	AT5G35360	Symbols: CAC2 | acetyl Co-enzyme a carboxylase	847
Dark-1	11	Background	AT5G35360	Symbols: CAC2 | acetyl Co-enzyme a carboxylase	847
Dark-1	11	Background	AT5G35360	Symbols: CAC2 | acetyl Co-enzyme a carboxylase	847
Dark-1	11	Background	AT5G35360	Symbols: CAC2 | acetyl Co-enzyme a carboxylase	847
Dark-1	11	Background	AT5G35360	Symbols: CAC2 | acetyl Co-enzyme a carboxylase	847
Dark-1	11	Background	AT5G35360	Symbols: CAC2 | acetyl Co-enzyme a carboxylase	847
Dark-1	11	Background	AT5G35360	Symbols: CAC2 | acetyl Co-enzyme a carboxylase	847
Dark-1	11	Background	AT5G35360	Symbols: CAC2 | acetyl Co-enzyme a carboxylase	847
Dark-1	11	Background	AT5G35360	Symbols: CAC2 | acetyl Co-enzyme a carboxylase	847
Dark-1	11	Background	AT5G35360	Symbols: CAC2 | acetyl Co-enzyme a carboxylase	847
Dark-1	11	Background	AT5G35360	Symbols: CAC2 | acetyl Co-enzyme a carboxylase	847
Dark-1	11	Background	AT5G35360	Symbols: CAC2 | acetyl Co-enzyme a carboxylase	847
Dark-1	11	Background	AT5G35360	Symbols: CAC2 | acetyl Co-enzyme a carboxylase	847
Dark-1	11	Background	AT5G35360	Symbols: CAC2 | acetyl Co-enzyme a carboxylase	847
Dark-1	12	Background	AT5G12250	Symbols: TUB6 | beta-6 tubulin | chr5:3961317-	787
Dark-1	12	Background	AT5G12250	Symbols: TUB6 | beta-6 tubulin | chr5:3961317-	787
Dark-1	12	Background	AT5G12250	Symbols: TUB6 | beta-6 tubulin | chr5:3961317-	787
Dark-1	12	Background	AT5G12250	Symbols: TUB6 | beta-6 tubulin | chr5:3961317-	787
Dark-1	12	Background	AT5G12250	Symbols: TUB6 | beta-6 tubulin | chr5:3961317-	787
Dark-1	12	Background	AT5G12250	Symbols: TUB6 | beta-6 tubulin | chr5:3961317-	787
Dark-1	12	Background	AT5G12250	Symbols: TUB6 | beta-6 tubulin | chr5:3961317-	787
Dark-1	12	Background	AT5G12250	Symbols: TUB6 | beta-6 tubulin | chr5:3961317-	787
Dark-1	12	Background	AT5G12250	Symbols: TUB6 | beta-6 tubulin | chr5:3961317-	787
Dark-1	12	Background	AT5G12250	Symbols: TUB6 | beta-6 tubulin | chr5:3961317-	787
Dark-1	12	Background	AT5G12250	Symbols: TUB6 | beta-6 tubulin | chr5:3961317-	787
Dark-1	12	Background	AT5G12250	Symbols: TUB6 | beta-6 tubulin | chr5:3961317-	787
Dark-1	12	Background	AT5G12250	Symbols: TUB6 | beta-6 tubulin | chr5:3961317-	787
Dark-1	12	Background	AT5G12250	Symbols: TUB6 | beta-6 tubulin | chr5:3961317-	787

Dark-1	12	Background	AT5G12250	Symbols: TUB6 | beta-6 tubulin | chr5:3961317-	787
Dark-1	13	Background	AT5G02490	Symbols: | Heat shock protein 70 (Hsp 70) famil	695
Dark-1	13	Background	AT5G02490	Symbols: | Heat shock protein 70 (Hsp 70) famil	695
Dark-1	13	Background	AT5G02490	Symbols: | Heat shock protein 70 (Hsp 70) famil	695
Dark-1	13	Background	AT5G02490	Symbols: | Heat shock protein 70 (Hsp 70) famil	695
Dark-1	13	Background	AT5G02490	Symbols: | Heat shock protein 70 (Hsp 70) famil	695
Dark-1	13	Background	AT5G02490	Symbols: | Heat shock protein 70 (Hsp 70) famil	695
Dark-1	13	Background	AT5G02490	Symbols: | Heat shock protein 70 (Hsp 70) famil	695
Dark-1	13	Background	AT5G02490	Symbols: | Heat shock protein 70 (Hsp 70) famil	695
Dark-1	13	Background	AT5G02490	Symbols: | Heat shock protein 70 (Hsp 70) famil	695
Dark-1	13	Background	AT5G02490	Symbols: | Heat shock protein 70 (Hsp 70) famil	695
Dark-1	13	Background	AT5G02490	Symbols: | Heat shock protein 70 (Hsp 70) famil	695
Dark-1	13	Background	AT5G02490	Symbols: | Heat shock protein 70 (Hsp 70) famil	695
Dark-1	13	Background	AT5G02490	Symbols: | Heat shock protein 70 (Hsp 70) famil	695
Dark-1	13	Background	AT5G02490	Symbols: | Heat shock protein 70 (Hsp 70) famil	695
Dark-1	14	Background	AT1G72730	Symbols: | DEA(D/H)-box RNA helicase family p	667
Dark-1	14	Background	AT1G72730	Symbols: | DEA(D/H)-box RNA helicase family p	667
Dark-1	14	Background	AT1G72730	Symbols: | DEA(D/H)-box RNA helicase family p	667
Dark-1	14	Background	AT1G72730	Symbols: | DEA(D/H)-box RNA helicase family p	667
Dark-1	14	Background	AT1G72730	Symbols: | DEA(D/H)-box RNA helicase family p	667
Dark-1	14	Background	AT1G72730	Symbols: | DEA(D/H)-box RNA helicase family p	667
Dark-1	14	Background	AT1G72730	Symbols: | DEA(D/H)-box RNA helicase family p	667
Dark-1	14	Background	AT1G72730	Symbols: | DEA(D/H)-box RNA helicase family p	667
Dark-1	14	Background	AT1G72730	Symbols: | DEA(D/H)-box RNA helicase family p	667
Dark-1	14	Background	AT1G72730	Symbols: | DEA(D/H)-box RNA helicase family p	667
Dark-1	14	Background	AT1G72730	Symbols: | DEA(D/H)-box RNA helicase family p	667
Dark-1	14	Background	AT1G72730	Symbols: | DEA(D/H)-box RNA helicase family p	667
Dark-1	14	Background	AT1G72730	Symbols: | DEA(D/H)-box RNA helicase family p	667
Dark-1	15	Background	AT5G19770	Symbols: TUA3 | tubulin alpha-3 | chr5:6682761	639
Dark-1	15	Background	AT5G19770	Symbols: TUA3 | tubulin alpha-3 | chr5:6682761	639
Dark-1	15	Background	AT5G19770	Symbols: TUA3 | tubulin alpha-3 | chr5:6682761	639
Dark-1	15	Background	AT5G19770	Symbols: TUA3 | tubulin alpha-3 | chr5:6682761	639
Dark-1	15	Background	AT5G19770	Symbols: TUA3 | tubulin alpha-3 | chr5:6682761	639
Dark-1	15	Background	AT5G19770	Symbols: TUA3 | tubulin alpha-3 | chr5:6682761	639
Dark-1	15	Background	AT5G19770	Symbols: TUA3 | tubulin alpha-3 | chr5:6682761	639
Dark-1	15	Background	AT5G19770	Symbols: TUA3 | tubulin alpha-3 | chr5:6682761	639
Dark-1	15	Background	AT5G19770	Symbols: TUA3 | tubulin alpha-3 | chr5:6682761	639
Dark-1	15	Background	AT5G19770	Symbols: TUA3 | tubulin alpha-3 | chr5:6682761	639
Dark-1	15	Background	AT5G19770	Symbols: TUA3 | tubulin alpha-3 | chr5:6682761	639
Dark-1	15	Background	AT5G19770	Symbols: TUA3 | tubulin alpha-3 | chr5:6682761	639
Dark-1	15	Background	AT5G19770	Symbols: TUA3 | tubulin alpha-3 | chr5:6682761	639
Dark-1	15	Background	AT5G19770	Symbols: TUA3 | tubulin alpha-3 | chr5:6682761	639
Dark-1	15	Background	AT5G19770	Symbols: TUA3 | tubulin alpha-3 | chr5:6682761	639
Dark-1	16	Background	AT4G37910	Symbols: mtHsc70-1 | mitochondrial heat shock	567
Dark-1	16	Background	AT4G37910	Symbols: mtHsc70-1 | mitochondrial heat shock	567
Dark-1	16	Background	AT4G37910	Symbols: mtHsc70-1 | mitochondrial heat shock	567
Dark-1	16	Background	AT4G37910	Symbols: mtHsc70-1 | mitochondrial heat shock	567
Dark-1	16	Background	AT4G37910	Symbols: mtHsc70-1 | mitochondrial heat shock	567
Dark-1	16	Background	AT4G37910	Symbols: mtHsc70-1 | mitochondrial heat shock	567
Dark-1	16	Background	AT4G37910	Symbols: mtHsc70-1 | mitochondrial heat shock	567
Dark-1	16	Background	AT4G37910	Symbols: mtHsc70-1 | mitochondrial heat shock	567
Dark-1	16	Background	AT4G37910	Symbols: mtHsc70-1 | mitochondrial heat shock	567
Dark-1	16	Background	AT4G37910	Symbols: mtHsc70-1 | mitochondrial heat shock	567
Dark-1	16	Background	AT4G37910	Symbols: mtHsc70-1 | mitochondrial heat shock	567
Dark-1	16	Background	AT4G37910	Symbols: mtHsc70-1 | mitochondrial heat shock	567
Dark-1	17	Background	AT1G13440	Symbols: GAPC-2, GAPC2 | glyceraldehyde-3-pho	538
Dark-1	17	Background	AT1G13440	Symbols: GAPC-2, GAPC2 | glyceraldehyde-3-pho	538
Dark-1	17	Background	AT1G13440	Symbols: GAPC-2, GAPC2 | glyceraldehyde-3-pho	538

Dark-1	17	Background	AT1G13440	Symbols: GAPC-2, GAPC2 | glyceraldehyde-3-ph	538
Dark-1	17	Background	AT1G13440	Symbols: GAPC-2, GAPC2 | glyceraldehyde-3-ph	538
Dark-1	17	Background	AT1G13440	Symbols: GAPC-2, GAPC2 | glyceraldehyde-3-ph	538
Dark-1	17	Background	AT1G13440	Symbols: GAPC-2, GAPC2 | glyceraldehyde-3-ph	538
Dark-1	17	Background	AT1G13440	Symbols: GAPC-2, GAPC2 | glyceraldehyde-3-ph	538
Dark-1	17	Background	AT1G13440	Symbols: GAPC-2, GAPC2 | glyceraldehyde-3-ph	538
Dark-1	17	Background	AT1G13440	Symbols: GAPC-2, GAPC2 | glyceraldehyde-3-ph	538
Dark-1	18	Background	AT2G02560	Symbols: CAND1, ATCAND1, ETA2, TIP120, HVE	526
Dark-1	18	Background	AT2G02560	Symbols: CAND1, ATCAND1, ETA2, TIP120, HVE	526
Dark-1	18	Background	AT2G02560	Symbols: CAND1, ATCAND1, ETA2, TIP120, HVE	526
Dark-1	18	Background	AT2G02560	Symbols: CAND1, ATCAND1, ETA2, TIP120, HVE	526
Dark-1	18	Background	AT2G02560	Symbols: CAND1, ATCAND1, ETA2, TIP120, HVE	526
Dark-1	18	Background	AT2G02560	Symbols: CAND1, ATCAND1, ETA2, TIP120, HVE	526
Dark-1	18	Background	AT2G02560	Symbols: CAND1, ATCAND1, ETA2, TIP120, HVE	526
Dark-1	18	Background	AT2G02560	Symbols: CAND1, ATCAND1, ETA2, TIP120, HVE	526
Dark-1	18	Background	AT2G02560	Symbols: CAND1, ATCAND1, ETA2, TIP120, HVE	526
Dark-1	18	Background	AT2G02560	Symbols: CAND1, ATCAND1, ETA2, TIP120, HVE	526
Dark-1	18	Background	AT2G02560	Symbols: CAND1, ATCAND1, ETA2, TIP120, HVE	526
Dark-1	18	Background	AT2G02560	Symbols: CAND1, ATCAND1, ETA2, TIP120, HVE	526
Dark-1	19	Background	AT5G03290	Symbols: IDH-V | isocitrate dehydrogenase V | c	498
Dark-1	19	Background	AT5G03290	Symbols: IDH-V | isocitrate dehydrogenase V | c	498
Dark-1	19	Background	AT5G03290	Symbols: IDH-V | isocitrate dehydrogenase V | c	498
Dark-1	19	Background	AT5G03290	Symbols: IDH-V | isocitrate dehydrogenase V | c	498
Dark-1	19	Background	AT5G03290	Symbols: IDH-V | isocitrate dehydrogenase V | c	498
Dark-1	19	Background	AT5G03290	Symbols: IDH-V | isocitrate dehydrogenase V | c	498
Dark-1	19	Background	AT5G03290	Symbols: IDH-V | isocitrate dehydrogenase V | c	498
Dark-1	19	Background	AT5G03290	Symbols: IDH-V | isocitrate dehydrogenase V | c	498
Dark-1	19	Background	AT5G03290	Symbols: IDH-V | isocitrate dehydrogenase V | c	498
Dark-1	20	Background	AT5G09810	Symbols: ACT7 | actin 7 | chr5:3052809-305422	485
Dark-1	20	Background	AT5G09810	Symbols: ACT7 | actin 7 | chr5:3052809-305422	485
Dark-1	20	Background	AT5G09810	Symbols: ACT7 | actin 7 | chr5:3052809-305422	485
Dark-1	20	Background	AT5G09810	Symbols: ACT7 | actin 7 | chr5:3052809-305422	485
Dark-1	20	Background	AT5G09810	Symbols: ACT7 | actin 7 | chr5:3052809-305422	485
Dark-1	20	Background	AT5G09810	Symbols: ACT7 | actin 7 | chr5:3052809-305422	485
Dark-1	20	Background	AT5G09810	Symbols: ACT7 | actin 7 | chr5:3052809-305422	485
Dark-1	20	Background	AT5G09810	Symbols: ACT7 | actin 7 | chr5:3052809-305422	485
Dark-1	20	Background	AT5G09810	Symbols: ACT7 | actin 7 | chr5:3052809-305422	485
Dark-1	20	Background	AT5G09810	Symbols: ACT7 | actin 7 | chr5:3052809-305422	485
Dark-1	21	Background	AT4G20360	Symbols: ATRAB8D, ATRABE1B, RABE1b | RAB G	480
Dark-1	21	Background	AT4G20360	Symbols: ATRAB8D, ATRABE1B, RABE1b | RAB G	480
Dark-1	21	Background	AT4G20360	Symbols: ATRAB8D, ATRABE1B, RABE1b | RAB G	480
Dark-1	21	Background	AT4G20360	Symbols: ATRAB8D, ATRABE1B, RABE1b | RAB G	480
Dark-1	21	Background	AT4G20360	Symbols: ATRAB8D, ATRABE1B, RABE1b | RAB G	480
Dark-1	21	Background	AT4G20360	Symbols: ATRAB8D, ATRABE1B, RABE1b | RAB G	480
Dark-1	21	Background	AT4G20360	Symbols: ATRAB8D, ATRABE1B, RABE1b | RAB G	480
Dark-1	21	Background	AT4G20360	Symbols: ATRAB8D, ATRABE1B, RABE1b | RAB G	480
Dark-1	21	Background	AT4G20360	Symbols: ATRAB8D, ATRABE1B, RABE1b | RAB G	480
Dark-1	22	Background	AT3G48870	Symbols: ATCLPC, ATHSP93-III, HSP93-III | Clp A	467
Dark-1	22	Background	AT3G48870	Symbols: ATCLPC, ATHSP93-III, HSP93-III | Clp A	467
Dark-1	22	Background	AT3G48870	Symbols: ATCLPC, ATHSP93-III, HSP93-III | Clp A	467
Dark-1	22	Background	AT3G48870	Symbols: ATCLPC, ATHSP93-III, HSP93-III | Clp A	467
Dark-1	22	Background	AT3G48870	Symbols: ATCLPC, ATHSP93-III, HSP93-III | Clp A	467
Dark-1	22	Background	AT3G48870	Symbols: ATCLPC, ATHSP93-III, HSP93-III | Clp A	467
Dark-1	22	Background	AT3G48870	Symbols: ATCLPC, ATHSP93-III, HSP93-III | Clp A	467
Dark-1	22	Background	AT3G48870	Symbols: ATCLPC, ATHSP93-III, HSP93-III | Clp A	467
Dark-1	22	Background	AT3G48870	Symbols: ATCLPC, ATHSP93-III, HSP93-III | Clp A	467
Dark-1	22	Background	AT3G48870	Symbols: ATCLPC, ATHSP93-III, HSP93-III | Clp A	467
Dark-1	22	Background	AT3G48870	Symbols: ATCLPC, ATHSP93-III, HSP93-III | Clp A	467

Dark-1	22	Background	AT3G48870	Symbols: ATCLPC, ATHSP93-III, HSP93-III | Clp A	467
Dark-1	22	Background	AT3G48870	Symbols: ATCLPC, ATHSP93-III, HSP93-III | Clp A	467
Dark-1	22	Background	AT3G48870	Symbols: ATCLPC, ATHSP93-III, HSP93-III | Clp A	467
Dark-1	22	Background	AT3G48870	Symbols: ATCLPC, ATHSP93-III, HSP93-III | Clp A	467
Dark-1	23	Table 1 Filtere	AT4G02380	Symbols: SAG21, AtLEA5 | senescence-associate	459
Dark-1	23	Table 1 Filtere	AT4G02380	Symbols: SAG21, AtLEA5 | senescence-associate	459
Dark-1	23	Table 1 Filtere	AT4G02380	Symbols: SAG21, AtLEA5 | senescence-associate	459
Dark-1	23	Table 1 Filtere	AT4G02380	Symbols: SAG21, AtLEA5 | senescence-associate	459
Dark-1	23	Table 1 Filtere	AT4G02380	Symbols: SAG21, AtLEA5 | senescence-associate	459
Dark-1	24	Background	AT1G07920	Symbols: | GTP binding Elongation factor Tu fan	450
Dark-1	24	Background	AT1G07920	Symbols: | GTP binding Elongation factor Tu fan	450
Dark-1	24	Background	AT1G07920	Symbols: | GTP binding Elongation factor Tu fan	450
Dark-1	24	Background	AT1G07920	Symbols: | GTP binding Elongation factor Tu fan	450
Dark-1	24	Background	AT1G07920	Symbols: | GTP binding Elongation factor Tu fan	450
Dark-1	24	Background	AT1G07920	Symbols: | GTP binding Elongation factor Tu fan	450
Dark-1	24	Background	AT1G07920	Symbols: | GTP binding Elongation factor Tu fan	450
Dark-1	24	Background	AT1G07920	Symbols: | GTP binding Elongation factor Tu fan	450
Dark-1	24	Background	AT1G07920	Symbols: | GTP binding Elongation factor Tu fan	450
Dark-1	24	Background	AT1G07920	Symbols: | GTP binding Elongation factor Tu fan	450
Dark-1	24	Background	AT1G07920	Symbols: | GTP binding Elongation factor Tu fan	450
Dark-1	25	Background	AT2G44060	Symbols: | Late embryogenesis abundant prote	447
Dark-1	25	Background	AT2G44060	Symbols: | Late embryogenesis abundant prote	447
Dark-1	25	Background	AT2G44060	Symbols: | Late embryogenesis abundant prote	447
Dark-1	25	Background	AT2G44060	Symbols: | Late embryogenesis abundant prote	447
Dark-1	25	Background	AT2G44060	Symbols: | Late embryogenesis abundant prote	447
Dark-1	25	Background	AT2G44060	Symbols: | Late embryogenesis abundant prote	447
Dark-1	25	Background	AT2G44060	Symbols: | Late embryogenesis abundant prote	447
Dark-1	25	Background	AT2G44060	Symbols: | Late embryogenesis abundant prote	447
Dark-1	26	Background	AT1G52670	Symbols: | Single hybrid motif superfamily prot	419
Dark-1	26	Background	AT1G52670	Symbols: | Single hybrid motif superfamily prot	419
Dark-1	26	Background	AT1G52670	Symbols: | Single hybrid motif superfamily prot	419
Dark-1	26	Background	AT1G52670	Symbols: | Single hybrid motif superfamily prot	419
Dark-1	26	Background	AT1G52670	Symbols: | Single hybrid motif superfamily prot	419
Dark-1	27	Background	AT3G22330	Symbols: PMH2, ATRH53 | putative mitochondri	371
Dark-1	27	Background	AT3G22330	Symbols: PMH2, ATRH53 | putative mitochondri	371
Dark-1	27	Background	AT3G22330	Symbols: PMH2, ATRH53 | putative mitochondri	371
Dark-1	27	Background	AT3G22330	Symbols: PMH2, ATRH53 | putative mitochondri	371
Dark-1	27	Background	AT3G22330	Symbols: PMH2, ATRH53 | putative mitochondri	371
Dark-1	27	Background	AT3G22330	Symbols: PMH2, ATRH53 | putative mitochondri	371
Dark-1	27	Background	AT3G22330	Symbols: PMH2, ATRH53 | putative mitochondri	371
Dark-1	27	Background	AT3G22330	Symbols: PMH2, ATRH53 | putative mitochondri	371
Dark-1	27	Background	AT3G22330	Symbols: PMH2, ATRH53 | putative mitochondri	371
Dark-1	27	Background	AT3G22330	Symbols: PMH2, ATRH53 | putative mitochondri	371
Dark-1	27	Background	AT3G22330	Symbols: PMH2, ATRH53 | putative mitochondri	371
Dark-1	27	Background	AT3G22330	Symbols: PMH2, ATRH53 | putative mitochondri	371
Dark-1	28	Background	AT3G09810	Symbols: IDH-VI | isocitrate dehydrogenase VI |	346
Dark-1	28	Background	AT3G09810	Symbols: IDH-VI | isocitrate dehydrogenase VI |	346
Dark-1	28	Background	AT3G09810	Symbols: IDH-VI | isocitrate dehydrogenase VI |	346
Dark-1	28	Background	AT3G09810	Symbols: IDH-VI | isocitrate dehydrogenase VI |	346
Dark-1	28	Background	AT3G09810	Symbols: IDH-VI | isocitrate dehydrogenase VI |	346
Dark-1	28	Background	AT3G09810	Symbols: IDH-VI | isocitrate dehydrogenase VI |	346
Dark-1	28	Background	AT3G09810	Symbols: IDH-VI | isocitrate dehydrogenase VI |	346
Dark-1	29	Background	AT3G08580	Symbols: AAC1 | ADP/ATP carrier 1 | chr3:2605	332
Dark-1	29	Background	AT3G08580	Symbols: AAC1 | ADP/ATP carrier 1 | chr3:2605	332
Dark-1	29	Background	AT3G08580	Symbols: AAC1 | ADP/ATP carrier 1 | chr3:2605	332
Dark-1	29	Background	AT3G08580	Symbols: AAC1 | ADP/ATP carrier 1 | chr3:2605	332
Dark-1	29	Background	AT3G08580	Symbols: AAC1 | ADP/ATP carrier 1 | chr3:2605	332
Dark-1	29	Background	AT3G08580	Symbols: AAC1 | ADP/ATP carrier 1 | chr3:2605	332

Dark-1	30	Background	AT1G24300	Symbols: | GYF domain-containing protein | chr	313
Dark-1	30	Background	AT1G24300	Symbols: | GYF domain-containing protein | chr	313
Dark-1	30	Background	AT1G24300	Symbols: | GYF domain-containing protein | chr	313
Dark-1	30	Background	AT1G24300	Symbols: | GYF domain-containing protein | chr	313
Dark-1	30	Background	AT1G24300	Symbols: | GYF domain-containing protein | chr	313
Dark-1	30	Background	AT1G24300	Symbols: | GYF domain-containing protein | chr	313
Dark-1	30	Background	AT1G24300	Symbols: | GYF domain-containing protein | chr	313
Dark-1	30	Background	AT1G24300	Symbols: | GYF domain-containing protein | chr	313
Dark-1	30	Background	AT1G24300	Symbols: | GYF domain-containing protein | chr	313
Dark-1	30	Background	AT1G24300	Symbols: | GYF domain-containing protein | chr	313
Dark-1	30	Background	AT1G24300	Symbols: | GYF domain-containing protein | chr	313
Dark-1	31	Background	AT5G09590	Symbols: MTHSC70-2, HSC70-5 | mitochondrial	313
Dark-1	31	Background	AT5G09590	Symbols: MTHSC70-2, HSC70-5 | mitochondrial	313
Dark-1	31	Background	AT5G09590	Symbols: MTHSC70-2, HSC70-5 | mitochondrial	313
Dark-1	31	Background	AT5G09590	Symbols: MTHSC70-2, HSC70-5 | mitochondrial	313
Dark-1	31	Background	AT5G09590	Symbols: MTHSC70-2, HSC70-5 | mitochondrial	313
Dark-1	31	Background	AT5G09590	Symbols: MTHSC70-2, HSC70-5 | mitochondrial	313
Dark-1	31	Background	AT5G09590	Symbols: MTHSC70-2, HSC70-5 | mitochondrial	313
Dark-1	31	Background	AT5G09590	Symbols: MTHSC70-2, HSC70-5 | mitochondrial	313
Dark-1	31	Background	AT5G09590	Symbols: MTHSC70-2, HSC70-5 | mitochondrial	313
Dark-1	31	Background	AT5G09590	Symbols: MTHSC70-2, HSC70-5 | mitochondrial	313
Dark-1	32	Background	AT4G35260	Symbols: IDH1, IDH-I | isocitrate dehydrogenase	307
Dark-1	32	Background	AT4G35260	Symbols: IDH1, IDH-I | isocitrate dehydrogenase	307
Dark-1	32	Background	AT4G35260	Symbols: IDH1, IDH-I | isocitrate dehydrogenase	307
Dark-1	32	Background	AT4G35260	Symbols: IDH1, IDH-I | isocitrate dehydrogenase	307
Dark-1	32	Background	AT4G35260	Symbols: IDH1, IDH-I | isocitrate dehydrogenase	307
Dark-1	32	Background	AT4G35260	Symbols: IDH1, IDH-I | isocitrate dehydrogenase	307
Dark-1	32	Background	AT4G35260	Symbols: IDH1, IDH-I | isocitrate dehydrogenase	307
Dark-1	32	Background	AT4G35260	Symbols: IDH1, IDH-I | isocitrate dehydrogenase	307
Dark-1	33	Background	AT1G09100	Symbols: RPT5B | 26S proteasome AAA-ATPase	301
Dark-1	33	Background	AT1G09100	Symbols: RPT5B | 26S proteasome AAA-ATPase	301
Dark-1	33	Background	AT1G09100	Symbols: RPT5B | 26S proteasome AAA-ATPase	301
Dark-1	33	Background	AT1G09100	Symbols: RPT5B | 26S proteasome AAA-ATPase	301
Dark-1	33	Background	AT1G09100	Symbols: RPT5B | 26S proteasome AAA-ATPase	301
Dark-1	33	Background	AT1G09100	Symbols: RPT5B | 26S proteasome AAA-ATPase	301
Dark-1	34	Background	AT2G39730	Symbols: RCA | rubisco activase | chr2:1657095	267
Dark-1	34	Background	AT2G39730	Symbols: RCA | rubisco activase | chr2:1657095	267
Dark-1	34	Background	AT2G39730	Symbols: RCA | rubisco activase | chr2:1657095	267
Dark-1	34	Background	AT2G39730	Symbols: RCA | rubisco activase | chr2:1657095	267
Dark-1	35	Background	AT5G49910	Symbols: CPHSC70-2EAT SHOCK PROTEIN 70-2, I	266
Dark-1	35	Background	AT5G49910	Symbols: CPHSC70-2EAT SHOCK PROTEIN 70-2, I	266
Dark-1	35	Background	AT5G49910	Symbols: CPHSC70-2EAT SHOCK PROTEIN 70-2, I	266
Dark-1	35	Background	AT5G49910	Symbols: CPHSC70-2EAT SHOCK PROTEIN 70-2, I	266
Dark-1	35	Background	AT5G49910	Symbols: CPHSC70-2EAT SHOCK PROTEIN 70-2, I	266
Dark-1	35	Background	AT5G49910	Symbols: CPHSC70-2EAT SHOCK PROTEIN 70-2, I	266
Dark-1	35	Background	AT5G49910	Symbols: CPHSC70-2EAT SHOCK PROTEIN 70-2, I	266
Dark-1	35	Background	AT5G49910	Symbols: CPHSC70-2EAT SHOCK PROTEIN 70-2, I	266
Dark-1	36	Background	AT2G37620	Symbols: ACT1, AAc1 | actin 1 | chr2:15779761-	248
Dark-1	36	Background	AT2G37620	Symbols: ACT1, AAc1 | actin 1 | chr2:15779761-	248
Dark-1	36	Background	AT2G37620	Symbols: ACT1, AAc1 | actin 1 | chr2:15779761-	248
Dark-1	36	Background	AT2G37620	Symbols: ACT1, AAc1 | actin 1 | chr2:15779761-	248
Dark-1	36	Background	AT2G37620	Symbols: ACT1, AAc1 | actin 1 | chr2:15779761-	248
Dark-1	36	Background	AT2G37620	Symbols: ACT1, AAc1 | actin 1 | chr2:15779761-	248
Dark-1	36	Background	AT2G37620	Symbols: ACT1, AAc1 | actin 1 | chr2:15779761-	248
Dark-1	37	Background	AT2G20580	Symbols: RPN1A, ATRPN1A | 26S proteasome re	247
Dark-1	37	Background	AT2G20580	Symbols: RPN1A, ATRPN1A | 26S proteasome re	247
Dark-1	37	Background	AT2G20580	Symbols: RPN1A, ATRPN1A | 26S proteasome re	247
Dark-1	37	Background	AT2G20580	Symbols: RPN1A, ATRPN1A | 26S proteasome re	247

Dark-1	37	Background	AT2G20580	Symbols: RPN1A, ATRPN1A | 26S proteasome re	247
Dark-1	37	Background	AT2G20580	Symbols: RPN1A, ATRPN1A | 26S proteasome re	247
Dark-1	37	Background	AT2G20580	Symbols: RPN1A, ATRPN1A | 26S proteasome re	247
Dark-1	38	Background	AT1G69840	Symbols: | SPFH/Band 7/PHB domain-containin	241
Dark-1	38	Background	AT1G69840	Symbols: | SPFH/Band 7/PHB domain-containin	241
Dark-1	38	Background	AT1G69840	Symbols: | SPFH/Band 7/PHB domain-containin	241
Dark-1	38	Background	AT1G69840	Symbols: | SPFH/Band 7/PHB domain-containin	241
Dark-1	38	Background	AT1G69840	Symbols: | SPFH/Band 7/PHB domain-containin	241
Dark-1	38	Background	AT1G69840	Symbols: | SPFH/Band 7/PHB domain-containin	241
Dark-1	39	Background	AT3G56150	Symbols: EIF3C, ATEIF3C-1, EIF3C-1, ATTIF3C1, T	241
Dark-1	39	Background	AT3G56150	Symbols: EIF3C, ATEIF3C-1, EIF3C-1, ATTIF3C1, T	241
Dark-1	39	Background	AT3G56150	Symbols: EIF3C, ATEIF3C-1, EIF3C-1, ATTIF3C1, T	241
Dark-1	39	Background	AT3G56150	Symbols: EIF3C, ATEIF3C-1, EIF3C-1, ATTIF3C1, T	241
Dark-1	40	Background	AT3G63460	Symbols: | transducin family protein / WD-40 re	236
Dark-1	40	Background	AT3G63460	Symbols: | transducin family protein / WD-40 re	236
Dark-1	40	Background	AT3G63460	Symbols: | transducin family protein / WD-40 re	236
Dark-1	40	Background	AT3G63460	Symbols: | transducin family protein / WD-40 re	236
Dark-1	40	Background	AT3G63460	Symbols: | transducin family protein / WD-40 re	236
Dark-1	40	Background	AT3G63460	Symbols: | transducin family protein / WD-40 re	236
Dark-1	41	Background	AT2G31610	Symbols: | Ribosomal protein S3 family protein	220
Dark-1	41	Background	AT2G31610	Symbols: | Ribosomal protein S3 family protein	220
Dark-1	41	Background	AT2G31610	Symbols: | Ribosomal protein S3 family protein	220
Dark-1	42	Background	AT4G02930	Symbols: | GTP binding Elongation factor Tu fan	219
Dark-1	42	Background	AT4G02930	Symbols: | GTP binding Elongation factor Tu fan	219
Dark-1	42	Background	AT4G02930	Symbols: | GTP binding Elongation factor Tu fan	219
Dark-1	42	Background	AT4G02930	Symbols: | GTP binding Elongation factor Tu fan	219
Dark-1	43	Background	AT5G62190	Symbols: PRH75 | DEAD box RNA helicase (PRH7	217
Dark-1	43	Background	AT5G62190	Symbols: PRH75 | DEAD box RNA helicase (PRH7	217
Dark-1	43	Background	AT5G62190	Symbols: PRH75 | DEAD box RNA helicase (PRH7	217
Dark-1	44	Background	AT1G55490	Symbols: CPN60B, LEN1 | chaperonin 60 beta |	215
Dark-1	44	Background	AT1G55490	Symbols: CPN60B, LEN1 | chaperonin 60 beta |	215
Dark-1	44	Background	AT1G55490	Symbols: CPN60B, LEN1 | chaperonin 60 beta |	215
Dark-1	44	Background	AT1G55490	Symbols: CPN60B, LEN1 | chaperonin 60 beta |	215
Dark-1	44	Background	AT1G55490	Symbols: CPN60B, LEN1 | chaperonin 60 beta |	215
Dark-1	45	Background	AT2G17130	Symbols: IDH2, IDH-II | isocitrate dehydrogenase	212
Dark-1	45	Background	AT2G17130	Symbols: IDH2, IDH-II | isocitrate dehydrogenase	212
Dark-1	45	Background	AT2G17130	Symbols: IDH2, IDH-II | isocitrate dehydrogenase	212
Dark-1	45	Background	AT2G17130	Symbols: IDH2, IDH-II | isocitrate dehydrogenase	212
Dark-1	45	Background	AT2G17130	Symbols: IDH2, IDH-II | isocitrate dehydrogenase	212
Dark-1	46	Background	AT2G46520	Symbols: | cellular apoptosis susceptibility prote	207
Dark-1	46	Background	AT2G46520	Symbols: | cellular apoptosis susceptibility prote	207
Dark-1	46	Background	AT2G46520	Symbols: | cellular apoptosis susceptibility prote	207
Dark-1	47	Background	AT2G37270	Symbols: ATRPS5B, RPS5B | ribosomal protein 5	206
Dark-1	47	Background	AT2G37270	Symbols: ATRPS5B, RPS5B | ribosomal protein 5	206
Dark-1	47	Background	AT2G37270	Symbols: ATRPS5B, RPS5B | ribosomal protein 5	206
Dark-1	47	Background	AT2G37270	Symbols: ATRPS5B, RPS5B | ribosomal protein 5	206
Dark-1	48	Background	AT1G72370	Symbols: P40, AP40, RP40, RPSAA | 40s ribosom	205
Dark-1	48	Background	AT1G72370	Symbols: P40, AP40, RP40, RPSAA | 40s ribosom	205
Dark-1	48	Background	AT1G72370	Symbols: P40, AP40, RP40, RPSAA | 40s ribosom	205
Dark-1	48	Background	AT1G72370	Symbols: P40, AP40, RP40, RPSAA | 40s ribosom	205
Dark-1	49	Background	AT5G56500	Symbols: | TCP-1/cpn60 chaperonin family prot	203
Dark-1	49	Background	AT5G56500	Symbols: | TCP-1/cpn60 chaperonin family prot	203
Dark-1	49	Background	AT5G56500	Symbols: | TCP-1/cpn60 chaperonin family prot	203
Dark-1	49	Background	AT5G56500	Symbols: | TCP-1/cpn60 chaperonin family prot	203
Dark-1	49	Background	AT5G56500	Symbols: | TCP-1/cpn60 chaperonin family prot	203
Dark-1	50	Background	AT1G56070	Symbols: LOS1 | Ribosomal protein S5/Elongatic	202
Dark-1	50	Background	AT1G56070	Symbols: LOS1 | Ribosomal protein S5/Elongatic	202
Dark-1	50	Background	AT1G56070	Symbols: LOS1 | Ribosomal protein S5/Elongatic	202

Dark-1	50	Background	AT1G56070	Symbols: LOS1 | Ribosomal protein S5/Elongatic	202
Dark-1	51	Background	AT3G22310	Symbols: PMH1, ATRH9 | putative mitochondria	200
Dark-1	51	Background	AT3G22310	Symbols: PMH1, ATRH9 | putative mitochondria	200
Dark-1	51	Background	AT3G22310	Symbols: PMH1, ATRH9 | putative mitochondria	200
Dark-1	51	Background	AT3G22310	Symbols: PMH1, ATRH9 | putative mitochondria	200
Dark-1	51	Background	AT3G22310	Symbols: PMH1, ATRH9 | putative mitochondria	200
Dark-1	51	Background	AT3G22310	Symbols: PMH1, ATRH9 | putative mitochondria	200
Dark-1	51	Background	AT3G22310	Symbols: PMH1, ATRH9 | putative mitochondria	200
Dark-1	52	Background	AT2G21390	Symbols: | Coatomer, alpha subunit | chr2:915[illegible]	199
Dark-1	52	Background	AT2G21390	Symbols: | Coatomer, alpha subunit | chr2:915[illegible]	199
Dark-1	52	Background	AT2G21390	Symbols: | Coatomer, alpha subunit | chr2:915[illegible]	199
Dark-1	52	Background	AT2G21390	Symbols: | Coatomer, alpha subunit | chr2:915[illegible]	199
Dark-1	53	Background	AT2G20140	Symbols: | AAA-type ATPase family protein | ch	199
Dark-1	53	Background	AT2G20140	Symbols: | AAA-type ATPase family protein | ch	199
Dark-1	54	Background	AT2G16950	Symbols: TRN1, ATTRN1 | transportin 1 | chr2:7	197
Dark-1	54	Background	AT2G16950	Symbols: TRN1, ATTRN1 | transportin 1 | chr2:7	197
Dark-1	54	Background	AT2G16950	Symbols: TRN1, ATTRN1 | transportin 1 | chr2:7	197
Dark-1	55	Background	AT5G13490	Symbols: AAC2 | ADP/ATP carrier 2 | chr5:4336[illegible]	191
Dark-1	55	Background	AT5G13490	Symbols: AAC2 | ADP/ATP carrier 2 | chr5:4336[illegible]	191
Dark-1	55	Background	AT5G13490	Symbols: AAC2 | ADP/ATP carrier 2 | chr5:4336[illegible]	191
Dark-1	55	Background	AT5G13490	Symbols: AAC2 | ADP/ATP carrier 2 | chr5:4336[illegible]	191
Dark-1	55	Background	AT5G13490	Symbols: AAC2 | ADP/ATP carrier 2 | chr5:4336[illegible]	191
Dark-1	55	Background	AT5G13490	Symbols: AAC2 | ADP/ATP carrier 2 | chr5:4336[illegible]	191
Dark-1	56	Background	AT1G33120	Symbols: | Ribosomal protein L6 family | chr1:1	187
Dark-1	56	Background	AT1G33120	Symbols: | Ribosomal protein L6 family | chr1:1	187
Dark-1	56	Background	AT1G33120	Symbols: | Ribosomal protein L6 family | chr1:1	187
Dark-1	56	Background	AT1G33120	Symbols: | Ribosomal protein L6 family | chr1:1	187
Dark-1	56	Background	AT1G33120	Symbols: | Ribosomal protein L6 family | chr1:1	187
Dark-1	57	Background	AT1G43800	Symbols: | Plant stearoyl-acyl-carrier-protein de	186
Dark-1	57	Background	AT1G43800	Symbols: | Plant stearoyl-acyl-carrier-protein de	186
Dark-1	57	Background	AT1G43800	Symbols: | Plant stearoyl-acyl-carrier-protein de	186
Dark-1	57	Background	AT1G43800	Symbols: | Plant stearoyl-acyl-carrier-protein de	186
Dark-1	57	Background	AT1G43800	Symbols: | Plant stearoyl-acyl-carrier-protein de	186
Dark-1	58	Background	AT5G08670	Symbols: | ATP synthase alpha/beta family prot	185
Dark-1	58	Background	AT5G08670	Symbols: | ATP synthase alpha/beta family prot	185
Dark-1	58	Background	AT5G08670	Symbols: | ATP synthase alpha/beta family prot	185
Dark-1	59	Background	AT2G07698	Symbols: | ATPase, F1 complex, alpha subunit p	179
Dark-1	59	Background	AT2G07698	Symbols: | ATPase, F1 complex, alpha subunit p	179
Dark-1	59	Background	AT2G07698	Symbols: | ATPase, F1 complex, alpha subunit p	179
Dark-1	60	Table 1 Filtere	AT3G16810	Symbols: APUM24, PUM24 | pumilio 24 | chr3:5	179
Dark-1	60	Table 1 Filtere	AT3G16810	Symbols: APUM24, PUM24 | pumilio 24 | chr3:5	179
Dark-1	61	Background	AT3G61650	Symbols: TUBG1 | gamma-tubulin | chr3:22812[illegible]	177
Dark-1	61	Background	AT3G61650	Symbols: TUBG1 | gamma-tubulin | chr3:22812[illegible]	177
Dark-1	61	Background	AT3G61650	Symbols: TUBG1 | gamma-tubulin | chr3:22812[illegible]	177
Dark-1	61	Background	AT3G61650	Symbols: TUBG1 | gamma-tubulin | chr3:22812[illegible]	177
Dark-1	61	Background	AT3G61650	Symbols: TUBG1 | gamma-tubulin | chr3:22812[illegible]	177
Dark-1	62	Background	AT1G67120	Symbols: | ATPases;nucleotide binding;ATP binc	172
Dark-1	62	Background	AT1G67120	Symbols: | ATPases;nucleotide binding;ATP binc	172
Dark-1	62	Background	AT1G67120	Symbols: | ATPases;nucleotide binding;ATP binc	172
Dark-1	62	Background	AT1G67120	Symbols: | ATPases;nucleotide binding;ATP binc	172
Dark-1	62	Background	AT1G67120	Symbols: | ATPases;nucleotide binding;ATP binc	172
Dark-1	63	Background	AT4G24820	Symbols: | 26S proteasome, regulatory subunit	171
Dark-1	63	Background	AT4G24820	Symbols: | 26S proteasome, regulatory subunit	171
Dark-1	63	Background	AT4G24820	Symbols: | 26S proteasome, regulatory subunit	171
Dark-1	64	Background	AT2G41800	Symbols: | Protein of unknown function, DUF64	167
Dark-1	64	Background	AT2G41800	Symbols: | Protein of unknown function, DUF64	167
Dark-1	64	Background	AT2G41800	Symbols: | Protein of unknown function, DUF64	167
Dark-1	64	Background	AT2G41800	Symbols: | Protein of unknown function, DUF64	167

Dark-1	64	Background	AT2G41800	Symbols: | Protein of unknown function, DUF64	167
Dark-1	65	Background	AT2G15860	Symbols: | unknown protein; FUNCTIONS IN: m	167
Dark-1	65	Background	AT2G15860	Symbols: | unknown protein; FUNCTIONS IN: m	167
Dark-1	66	Background	AT5G43010	Symbols: RPT4A | regulatory particle triple-A AT	166
Dark-1	66	Background	AT5G43010	Symbols: RPT4A | regulatory particle triple-A AT	166
Dark-1	66	Background	AT5G43010	Symbols: RPT4A | regulatory particle triple-A AT	166
Dark-1	66	Background	AT5G43010	Symbols: RPT4A | regulatory particle triple-A AT	166
Dark-1	66	Background	AT5G43010	Symbols: RPT4A | regulatory particle triple-A AT	166
Dark-1	67	Background	AT2G42520	Symbols: | P-loop containing nucleoside triphos	162
Dark-1	67	Background	AT2G42520	Symbols: | P-loop containing nucleoside triphos	162
Dark-1	67	Background	AT2G42520	Symbols: | P-loop containing nucleoside triphos	162
Dark-1	67	Background	AT2G42520	Symbols: | P-loop containing nucleoside triphos	162
Dark-1	67	Background	AT2G42520	Symbols: | P-loop containing nucleoside triphos	162
Dark-1	68	Background	AT3G09200	Symbols: | Ribosomal protein L10 family protei	162
Dark-1	68	Background	AT3G09200	Symbols: | Ribosomal protein L10 family protei	162
Dark-1	68	Background	AT3G09200	Symbols: | Ribosomal protein L10 family protei	162
Dark-1	68	Background	AT3G09200	Symbols: | Ribosomal protein L10 family protei	162
Dark-1	69	Background	AT5G20290	Symbols: | Ribosomal protein S8e family protei	161
Dark-1	69	Background	AT5G20290	Symbols: | Ribosomal protein S8e family protei	161
Dark-1	69	Background	AT5G20290	Symbols: | Ribosomal protein S8e family protei	161
Dark-1	70	Background	AT3G12050	Symbols: | Aha1 domain-containing protein | cl	155
Dark-1	70	Background	AT3G12050	Symbols: | Aha1 domain-containing protein | cl	155
Dark-1	70	Background	AT3G12050	Symbols: | Aha1 domain-containing protein | cl	155
Dark-1	71	Background	AT1G64790	Symbols: ILA | ILITYHIA | chr1:24065232-24081	153
Dark-1	71	Background	AT1G64790	Symbols: ILA | ILITYHIA | chr1:24065232-24081	153
Dark-1	71	Background	AT1G64790	Symbols: ILA | ILITYHIA | chr1:24065232-24081	153
Dark-1	71	Background	AT1G64790	Symbols: ILA | ILITYHIA | chr1:24065232-24081	153
Dark-1	71	Background	AT1G64790	Symbols: ILA | ILITYHIA | chr1:24065232-24081	153
Dark-1	72	Background	AT5G56000	Symbols: Hsp81.4, AtHsp90.4 | HEAT SHOCK PR	153
Dark-1	72	Background	AT5G56000	Symbols: Hsp81.4, AtHsp90.4 | HEAT SHOCK PR	153
Dark-1	72	Background	AT5G56000	Symbols: Hsp81.4, AtHsp90.4 | HEAT SHOCK PR	153
Dark-1	72	Background	AT5G56000	Symbols: Hsp81.4, AtHsp90.4 | HEAT SHOCK PR	153
Dark-1	72	Background	AT5G56000	Symbols: Hsp81.4, AtHsp90.4 | HEAT SHOCK PR	153
Dark-1	73	Background	AT2G24050	Symbols: eIFiso4G2 | MIF4G domain-containing	147
Dark-1	73	Background	AT2G24050	Symbols: eIFiso4G2 | MIF4G domain-containing	147
Dark-1	73	Background	AT2G24050	Symbols: eIFiso4G2 | MIF4G domain-containing	147
Dark-1	73	Background	AT2G24050	Symbols: eIFiso4G2 | MIF4G domain-containing	147
Dark-1	74	Background	AT1G43170	Symbols: ARP1, emb2207, RPL3A, RP1 | ribosom	146
Dark-1	74	Background	AT1G43170	Symbols: ARP1, emb2207, RPL3A, RP1 | ribosom	146
Dark-1	74	Background	AT1G43170	Symbols: ARP1, emb2207, RPL3A, RP1 | ribosom	146
Dark-1	74	Background	AT1G43170	Symbols: ARP1, emb2207, RPL3A, RP1 | ribosom	146
Dark-1	74	Background	AT1G43170	Symbols: ARP1, emb2207, RPL3A, RP1 | ribosom	146
Dark-1	74	Background	AT1G43170	Symbols: ARP1, emb2207, RPL3A, RP1 | ribosom	146
Dark-1	74	Background	AT1G43170	Symbols: ARP1, emb2207, RPL3A, RP1 | ribosom	146
Dark-1	75	Background	AT5G58290	Symbols: RPT3 | regulatory particle triple-A ATP	145
Dark-1	75	Background	AT5G58290	Symbols: RPT3 | regulatory particle triple-A ATP	145
Dark-1	75	Background	AT5G58290	Symbols: RPT3 | regulatory particle triple-A ATP	145
Dark-1	75	Background	AT5G58290	Symbols: RPT3 | regulatory particle triple-A ATP	145
Dark-1	76	Background	AT1G79530	Symbols: GAPCP-1 | glyceraldehyde-3-phosphat	141
Dark-1	76	Background	AT1G79530	Symbols: GAPCP-1 | glyceraldehyde-3-phosphat	141
Dark-1	77	Background	AT4G01100	Symbols: ADNT1 | adenine nucleotide transport	140
Dark-1	77	Background	AT4G01100	Symbols: ADNT1 | adenine nucleotide transport	140
Dark-1	78	Background	AT5G62740	Symbols: HIR1, ATHIR1 | SPFH/Band 7/PHB dom	140
Dark-1	78	Background	AT5G62740	Symbols: HIR1, ATHIR1 | SPFH/Band 7/PHB dom	140
Dark-1	78	Background	AT5G62740	Symbols: HIR1, ATHIR1 | SPFH/Band 7/PHB dom	140
Dark-1	79	Background	AT5G12370	Symbols: SEC10 | exocyst complex component s	139
Dark-1	79	Background	AT5G12370	Symbols: SEC10 | exocyst complex component s	139
Dark-1	80	Table 1 Filtere	AT1G59990	Symbols: | DEA(D/H)-box RNA helicase family p	136

Dark-1	80	Table 1 Filtere	AT1G59990	Symbols: | DEA(D/H)-box RNA helicase family p	136
Dark-1	80	Table 1 Filtere	AT1G59990	Symbols: | DEA(D/H)-box RNA helicase family p	136
Dark-1	81	Background	AT3G09630	Symbols: | Ribosomal protein L4/L1 family | chr	134
Dark-1	81	Background	AT3G09630	Symbols: | Ribosomal protein L4/L1 family | chr	134
Dark-1	81	Background	AT3G09630	Symbols: | Ribosomal protein L4/L1 family | chr	134
Dark-1	81	Background	AT3G09630	Symbols: | Ribosomal protein L4/L1 family | chr	134
Dark-1	82	Background	AT3G04840	Symbols: | Ribosomal protein S3Ae | chr3:1329	133
Dark-1	82	Background	AT3G04840	Symbols: | Ribosomal protein S3Ae | chr3:1329	133
Dark-1	82	Background	AT3G04840	Symbols: | Ribosomal protein S3Ae | chr3:1329	133
Dark-1	83	Background	AT1G22780	Symbols: PFL, RPS18A, PFL1 | Ribosomal protein	132
Dark-1	83	Background	AT1G22780	Symbols: PFL, RPS18A, PFL1 | Ribosomal protein	132
Dark-1	83	Background	AT1G22780	Symbols: PFL, RPS18A, PFL1 | Ribosomal protein	132
Dark-1	84	Background	AT5G14040	Symbols: PHT3;1 | phosphate transporter 3;1 | c	130
Dark-1	84	Background	AT5G14040	Symbols: PHT3;1 | phosphate transporter 3;1 | c	130
Dark-1	85	Background	AT3G08530	Symbols: | Clathrin, heavy chain | chr3:258717	124
Dark-1	85	Background	AT3G08530	Symbols: | Clathrin, heavy chain | chr3:258717	124
Dark-1	85	Background	AT3G08530	Symbols: | Clathrin, heavy chain | chr3:258717	124
Dark-1	85	Background	AT3G08530	Symbols: | Clathrin, heavy chain | chr3:258717	124
Dark-1	86	Background	AT1G36180	Symbols: ACC2 | acetyl-CoA carboxylase 2 | chr1	122
Dark-1	86	Background	AT1G36180	Symbols: ACC2 | acetyl-CoA carboxylase 2 | chr1	122
Dark-1	87	Background	AT3G05590	Symbols: RPL18 | ribosomal protein L18 | chr3:1	121
Dark-1	87	Background	AT3G05590	Symbols: RPL18 | ribosomal protein L18 | chr3:1	121
Dark-1	88	Background	AT5G19990	Symbols: RPT6A, ATSUG1 | regulatory particle tr	119
Dark-1	88	Background	AT5G19990	Symbols: RPT6A, ATSUG1 | regulatory particle tr	119
Dark-1	88	Background	AT5G19990	Symbols: RPT6A, ATSUG1 | regulatory particle tr	119
Dark-1	88	Background	AT5G19990	Symbols: RPT6A, ATSUG1 | regulatory particle tr	119
Dark-1	89	Background	AT1G10630	Symbols: ATARFA1F, ARFA1F | ADP-ribosylation	116
Dark-1	89	Background	AT1G10630	Symbols: ATARFA1F, ARFA1F | ADP-ribosylation	116
Dark-1	89	Background	AT1G10630	Symbols: ATARFA1F, ARFA1F | ADP-ribosylation	116
Dark-1	90	Background	AT2G36620	Symbols: RPL24A | ribosomal protein L24 | chr2	114
Dark-1	90	Background	AT2G36620	Symbols: RPL24A | ribosomal protein L24 | chr2	114
Dark-1	91	Background	AT1G80270	Symbols: PPR596 | PENTATRICOPEPTIDE REPEAT	114
Dark-1	91	Background	AT1G80270	Symbols: PPR596 | PENTATRICOPEPTIDE REPEAT	114
Dark-1	92	Background	AT2G42740	Symbols: RPL16A | ribosomal protein large subu	113
Dark-1	92	Background	AT2G42740	Symbols: RPL16A | ribosomal protein large subu	113
Dark-1	92	Background	AT2G42740	Symbols: RPL16A | ribosomal protein large subu	113
Dark-1	93	Background	AT1G80670	Symbols: | Transducin/WD40 repeat-like superf	111
Dark-1	93	Background	AT1G80670	Symbols: | Transducin/WD40 repeat-like superf	111
Dark-1	94	Background	AT5G37510	Symbols: EMB1467, CI76 | NADH-ubiquinone de	111
Dark-1	94	Background	AT5G37510	Symbols: EMB1467, CI76 | NADH-ubiquinone de	111
Dark-1	95	Background	AT3G57290	Symbols: EIF3E, TIF3E1, ATEIF3E-1, INT-6, ATINT	109
Dark-1	95	Background	AT3G57290	Symbols: EIF3E, TIF3E1, ATEIF3E-1, INT-6, ATINT	109
Dark-1	96	Background	AT3G23990	Symbols: HSP60, HSP60-3B | heat shock protein	107
Dark-1	96	Background	AT3G23990	Symbols: HSP60, HSP60-3B | heat shock protein	107
Dark-1	97	Background	AT5G28540	Symbols: BIP1 | heat shock protein 70 (Hsp 70) f	106
Dark-1	97	Background	AT5G28540	Symbols: BIP1 | heat shock protein 70 (Hsp 70) f	106
Dark-1	97	Background	AT5G28540	Symbols: BIP1 | heat shock protein 70 (Hsp 70) f	106
Dark-1	98	Background	AT1G20200	Symbols: EMB2719, HAP15 | PAM domain (PCI/	104
Dark-1	98	Background	AT1G20200	Symbols: EMB2719, HAP15 | PAM domain (PCI/	104
Dark-1	98	Background	AT1G20200	Symbols: EMB2719, HAP15 | PAM domain (PCI/	104
Dark-1	99	Background	AT2G31660	Symbols: SAD2, URM9 | ARM repeat superfamily	103
Dark-1	99	Background	AT2G31660	Symbols: SAD2, URM9 | ARM repeat superfamily	103
Dark-1	100	Background	AT3G17390	Symbols: MTO3, SAMS3, MAT4 | S-adenosylmet	103
Dark-1	100	Background	AT3G17390	Symbols: MTO3, SAMS3, MAT4 | S-adenosylmet	103
Dark-1	100	Background	AT3G17390	Symbols: MTO3, SAMS3, MAT4 | S-adenosylmet	103
Dark-1	101	Background	AT3G05560	Symbols: | Ribosomal L22e protein family | chr3	102
Dark-1	101	Background	AT3G05560	Symbols: | Ribosomal L22e protein family | chr3	102
Dark-1	102	Background	AT2G02790	Symbols: IQD29 | IQ-domain 29 | chr2:788708-7	101

Dark-1	102	Background	AT2G02790	Symbols: IQD29 | IQ-domain 29 | chr2:788708-7	101
Dark-1	103	Background	AT1G53750	Symbols: RPT1A | regulatory particle triple-A 1A	100
Dark-1	103	Background	AT1G53750	Symbols: RPT1A | regulatory particle triple-A 1A	100
Dark-1	104	Background	AT5G19820	Symbols: emb2734 | ARM repeat superfamily pr	100
Dark-1	104	Background	AT5G19820	Symbols: emb2734 | ARM repeat superfamily pr	100
Dark-1	105	Background	AT2G17360	Symbols: | Ribosomal protein S4 (RPS4A) family	99
Dark-1	105	Background	AT2G17360	Symbols: | Ribosomal protein S4 (RPS4A) family	99
Dark-1	105	Background	AT2G17360	Symbols: | Ribosomal protein S4 (RPS4A) family	99
Dark-1	105	Background	AT2G17360	Symbols: | Ribosomal protein S4 (RPS4A) family	99
Dark-1	106	Background	AT2G15620	Symbols: NIR1, NIR, ATHNIR | nitrite reductase 1	98
Dark-1	106	Background	AT2G15620	Symbols: NIR1, NIR, ATHNIR | nitrite reductase 1	98
Dark-1	106	Background	AT2G15620	Symbols: NIR1, NIR, ATHNIR | nitrite reductase 1	98
Dark-1	107	Background	AT4G11420	Symbols: EIF3A, ATEIF3A-1, EIF3A-1, ATTIF3A1, T	98
Dark-1	107	Background	AT4G11420	Symbols: EIF3A, ATEIF3A-1, EIF3A-1, ATTIF3A1, T	98
Dark-1	108	Background	AT5G16390	Symbols: CAC1, CAC1A, BCCP, BCCP1 | chloropla	98
Dark-1	108	Background	AT5G16390	Symbols: CAC1, CAC1A, BCCP, BCCP1 | chloropla	98
Dark-1	108	Background	AT5G16390	Symbols: CAC1, CAC1A, BCCP, BCCP1 | chloropla	98
Dark-1	109	Background	AT2G18330	Symbols: | AAA-type ATPase family protein | ch	97
Dark-1	109	Background	AT2G18330	Symbols: | AAA-type ATPase family protein | ch	97
Dark-1	110	Background	AT3G07050	Symbols: | GTP-binding family protein | chr3:22	97
Dark-1	110	Background	AT3G07050	Symbols: | GTP-binding family protein | chr3:22	97
Dark-1	111	Background	AT5G08610	Symbols: | P-loop containing nucleoside triphos	93
Dark-1	111	Background	AT5G08610	Symbols: | P-loop containing nucleoside triphos	93
Dark-1	111	Background	AT5G08610	Symbols: | P-loop containing nucleoside triphos	93
Dark-1	112	Background	AT5G36230	Symbols: | ARM repeat superfamily protein | ch	92
Dark-1	112	Background	AT5G36230	Symbols: | ARM repeat superfamily protein | ch	92
Dark-1	112	Background	AT5G36230	Symbols: | ARM repeat superfamily protein | ch	92
Dark-1	112	Background	AT5G36230	Symbols: | ARM repeat superfamily protein | ch	92
Dark-1	113	Background	AT5G18700	Symbols: RUK, EMB3013 | Protein kinase family	90
Dark-1	113	Background	AT5G18700	Symbols: RUK, EMB3013 | Protein kinase family	90
Dark-1	114	Background	AT3G54110	Symbols: ATPUMP1, UCP, PUMP1, ATUCP1, UCP	90
Dark-1	114	Background	AT3G54110	Symbols: ATPUMP1, UCP, PUMP1, ATUCP1, UCP	90
Dark-1	115	Background	AT1G56110	Symbols: NOP56 | homolog of nucleolar protein	84
Dark-1	115	Background	AT1G56110	Symbols: NOP56 | homolog of nucleolar protein	84
Dark-1	116	Background	AT1G29150	Symbols: ATS9, RPN6 | non-ATPase subunit 9 | c	83
Dark-1	116	Background	AT1G29150	Symbols: ATS9, RPN6 | non-ATPase subunit 9 | c	83
Dark-1	116	Background	AT1G29150	Symbols: ATS9, RPN6 | non-ATPase subunit 9 | c	83
Dark-1	117	Background	AT3G56130	Symbols: | biotin/lipoyl attachment domain-cor	80
Dark-1	117	Background	AT3G56130	Symbols: | biotin/lipoyl attachment domain-cor	80
Dark-1	118	Background	AT3G44110	Symbols: ATJ3, ATJ | DNAJ homologue 3 | chr3:1	79
Dark-1	118	Background	AT3G44110	Symbols: ATJ3, ATJ | DNAJ homologue 3 | chr3:1	79
Dark-1	119	Background	AT5G17020	Symbols: XPO1A, ATCRM1, ATXPO1, XPO1, HIT2	78
Dark-1	119	Background	AT5G17020	Symbols: XPO1A, ATCRM1, ATXPO1, XPO1, HIT2	78
Dark-1	120	Background	AT3G06720	Symbols: AT-IMP, ATKAP ALPHA, AIMP ALPHA, II	78
Dark-1	120	Background	AT3G06720	Symbols: AT-IMP, ATKAP ALPHA, AIMP ALPHA, II	78
Dark-1	121	Background	AT5G22060	Symbols: ATJ2, J2 | DNAJ homologue 2 | chr5:73	78
Dark-1	121	Background	AT5G22060	Symbols: ATJ2, J2 | DNAJ homologue 2 | chr5:73	78
Dark-1	122	Background	AT3G20050	Symbols: ATTCP-1, TCP-1 | T-complex protein 1	77
Dark-1	122	Background	AT3G20050	Symbols: ATTCP-1, TCP-1 | T-complex protein 1	77
Dark-1	122	Background	AT3G20050	Symbols: ATTCP-1, TCP-1 | T-complex protein 1	77
Dark-1	123	Background	AT5G27770	Symbols: | Ribosomal L22e protein family | chr5	75
Dark-1	123	Background	AT5G27770	Symbols: | Ribosomal L22e protein family | chr5	75
Dark-1	124	Background	AT2G36160	Symbols: | Ribosomal protein S11 family protein	74
Dark-1	124	Background	AT2G36160	Symbols: | Ribosomal protein S11 family protein	74
Dark-1	125	Background	AT2G01250	Symbols: | Ribosomal protein L30/L7 family pro	67
Dark-1	125	Background	AT2G01250	Symbols: | Ribosomal protein L30/L7 family pro	67
Dark-1	126	Background	AT1G10490	Symbols: | Domain of unknown function (DUF1	64
Dark-1	126	Background	AT1G10490	Symbols: | Domain of unknown function (DUF1	64

Dark-1	127	Background	AT1G71820	Symbols: SEC6 | SEC6 | chr1:27010022-2701674	63
Dark-1	127	Background	AT1G71820	Symbols: SEC6 | SEC6 | chr1:27010022-2701674	63
Dark-1	128	Background	AT2G40730	Symbols: | Protein kinase family protein with AI	60
Dark-1	128	Background	AT2G40730	Symbols: | Protein kinase family protein with AI	60
Dark-1	129	Background	AT1G04810	Symbols: | 26S proteasome regulatory complex	60
Dark-1	129	Background	AT1G04810	Symbols: | 26S proteasome regulatory complex	60
Dark-1	130	Background	AT2G07360	Symbols: | SH3 domain-containing protein | chr	58
Dark-1	130	Background	AT2G07360	Symbols: | SH3 domain-containing protein | chr	58
Dark-1	131	Table 1 Filtere	AT1G55150	Symbols: | DEA(D/H)-box RNA helicase family p	53
Dark-1	131	Table 1 Filtere	AT1G55150	Symbols: | DEA(D/H)-box RNA helicase family p	53
Dark-1	132	Background	AT4G25630	Symbols: FIB2, ATFIB2 | fibrillarin 2 | chr4:1307	51
Dark-1	132	Background	AT4G25630	Symbols: FIB2, ATFIB2 | fibrillarin 2 | chr4:1307	51
Dark-1	133	Background	AT5G16130	Symbols: | Ribosomal protein S7e family protei	48
Dark-1	133	Background	AT5G16130	Symbols: | Ribosomal protein S7e family protei	48
Dark-1	134	Background	AT1G79340	Symbols: AtMC4, MC4 | metacaspase 4 | chr1:2	40
Dark-1	134	Background	AT1G79340	Symbols: AtMC4, MC4 | metacaspase 4 | chr1:2	40
Dark-2	135	Background	prot_acc	prot_desc	prot_score
Dark-2	136	Background	AT5G44340	Symbols: TUB4 | tubulin beta chain 4 | chr5:178	1584
Dark-2	136	Background	AT5G44340	Symbols: TUB4 | tubulin beta chain 4 | chr5:178	1584
Dark-2	136	Background	AT5G44340	Symbols: TUB4 | tubulin beta chain 4 | chr5:178	1584
Dark-2	136	Background	AT5G44340	Symbols: TUB4 | tubulin beta chain 4 | chr5:178	1584
Dark-2	136	Background	AT5G44340	Symbols: TUB4 | tubulin beta chain 4 | chr5:178	1584
Dark-2	136	Background	AT5G44340	Symbols: TUB4 | tubulin beta chain 4 | chr5:178	1584
Dark-2	136	Background	AT5G44340	Symbols: TUB4 | tubulin beta chain 4 | chr5:178	1584
Dark-2	136	Background	AT5G44340	Symbols: TUB4 | tubulin beta chain 4 | chr5:178	1584
Dark-2	136	Background	AT5G44340	Symbols: TUB4 | tubulin beta chain 4 | chr5:178	1584
Dark-2	136	Background	AT5G44340	Symbols: TUB4 | tubulin beta chain 4 | chr5:178	1584
Dark-2	136	Background	AT5G44340	Symbols: TUB4 | tubulin beta chain 4 | chr5:178	1584
Dark-2	136	Background	AT5G44340	Symbols: TUB4 | tubulin beta chain 4 | chr5:178	1584
Dark-2	136	Background	AT5G44340	Symbols: TUB4 | tubulin beta chain 4 | chr5:178	1584
Dark-2	136	Background	AT5G44340	Symbols: TUB4 | tubulin beta chain 4 | chr5:178	1584
Dark-2	136	Background	AT5G44340	Symbols: TUB4 | tubulin beta chain 4 | chr5:178	1584
Dark-2	136	Background	AT5G44340	Symbols: TUB4 | tubulin beta chain 4 | chr5:178	1584
Dark-2	136	Background	AT5G44340	Symbols: TUB4 | tubulin beta chain 4 | chr5:178	1584
Dark-2	136	Background	AT5G44340	Symbols: TUB4 | tubulin beta chain 4 | chr5:178	1584
Dark-2	136	Background	AT5G44340	Symbols: TUB4 | tubulin beta chain 4 | chr5:178	1584
Dark-2	136	Background	AT5G44340	Symbols: TUB4 | tubulin beta chain 4 | chr5:178	1584
Dark-2	136	Background	AT5G44340	Symbols: TUB4 | tubulin beta chain 4 | chr5:178	1584
Dark-2	136	Background	AT5G44340	Symbols: TUB4 | tubulin beta chain 4 | chr5:178	1584
Dark-2	137	Background	AT5G62690	Symbols: TUB2 | tubulin beta chain 2 | chr5:251	1473
Dark-2	137	Background	AT5G62690	Symbols: TUB2 | tubulin beta chain 2 | chr5:251	1473
Dark-2	137	Background	AT5G62690	Symbols: TUB2 | tubulin beta chain 2 | chr5:251	1473
Dark-2	137	Background	AT5G62690	Symbols: TUB2 | tubulin beta chain 2 | chr5:251	1473
Dark-2	137	Background	AT5G62690	Symbols: TUB2 | tubulin beta chain 2 | chr5:251	1473
Dark-2	137	Background	AT5G62690	Symbols: TUB2 | tubulin beta chain 2 | chr5:251	1473
Dark-2	137	Background	AT5G62690	Symbols: TUB2 | tubulin beta chain 2 | chr5:251	1473
Dark-2	137	Background	AT5G62690	Symbols: TUB2 | tubulin beta chain 2 | chr5:251	1473
Dark-2	137	Background	AT5G62690	Symbols: TUB2 | tubulin beta chain 2 | chr5:251	1473
Dark-2	137	Background	AT5G62690	Symbols: TUB2 | tubulin beta chain 2 | chr5:251	1473
Dark-2	137	Background	AT5G62690	Symbols: TUB2 | tubulin beta chain 2 | chr5:251	1473
Dark-2	137	Background	AT5G62690	Symbols: TUB2 | tubulin beta chain 2 | chr5:251	1473
Dark-2	137	Background	AT5G62690	Symbols: TUB2 | tubulin beta chain 2 | chr5:251	1473
Dark-2	137	Background	AT5G62690	Symbols: TUB2 | tubulin beta chain 2 | chr5:251	1473
Dark-2	137	Background	AT5G62690	Symbols: TUB2 | tubulin beta chain 2 | chr5:251	1473
Dark-2	137	Background	AT5G62690	Symbols: TUB2 | tubulin beta chain 2 | chr5:251	1473
Dark-2	137	Background	AT5G62690	Symbols: TUB2 | tubulin beta chain 2 | chr5:251	1473
Dark-2	137	Background	AT5G62690	Symbols: TUB2 | tubulin beta chain 2 | chr5:251	1473
Dark-2	137	Background	AT5G62690	Symbols: TUB2 | tubulin beta chain 2 | chr5:251	1473

Dark-2	137	Background	AT5G62690	Symbols: TUB2 | tubulin beta chain 2 | chr5:251	1473
Dark-2	137	Background	AT5G62690	Symbols: TUB2 | tubulin beta chain 2 | chr5:251	1473
Dark-2	137	Background	AT5G62690	Symbols: TUB2 | tubulin beta chain 2 | chr5:251	1473
Dark-2	137	Background	AT5G62690	Symbols: TUB2 | tubulin beta chain 2 | chr5:251	1473
Dark-2	138	Background	AT2G29550	Symbols: TUB7 | tubulin beta-7 chain | chr2:126	1445
Dark-2	138	Background	AT2G29550	Symbols: TUB7 | tubulin beta-7 chain | chr2:126	1445
Dark-2	138	Background	AT2G29550	Symbols: TUB7 | tubulin beta-7 chain | chr2:126	1445
Dark-2	138	Background	AT2G29550	Symbols: TUB7 | tubulin beta-7 chain | chr2:126	1445
Dark-2	138	Background	AT2G29550	Symbols: TUB7 | tubulin beta-7 chain | chr2:126	1445
Dark-2	138	Background	AT2G29550	Symbols: TUB7 | tubulin beta-7 chain | chr2:126	1445
Dark-2	138	Background	AT2G29550	Symbols: TUB7 | tubulin beta-7 chain | chr2:126	1445
Dark-2	138	Background	AT2G29550	Symbols: TUB7 | tubulin beta-7 chain | chr2:126	1445
Dark-2	138	Background	AT2G29550	Symbols: TUB7 | tubulin beta-7 chain | chr2:126	1445
Dark-2	138	Background	AT2G29550	Symbols: TUB7 | tubulin beta-7 chain | chr2:126	1445
Dark-2	138	Background	AT2G29550	Symbols: TUB7 | tubulin beta-7 chain | chr2:126	1445
Dark-2	138	Background	AT2G29550	Symbols: TUB7 | tubulin beta-7 chain | chr2:126	1445
Dark-2	138	Background	AT2G29550	Symbols: TUB7 | tubulin beta-7 chain | chr2:126	1445
Dark-2	138	Background	AT2G29550	Symbols: TUB7 | tubulin beta-7 chain | chr2:126	1445
Dark-2	138	Background	AT2G29550	Symbols: TUB7 | tubulin beta-7 chain | chr2:126	1445
Dark-2	138	Background	AT2G29550	Symbols: TUB7 | tubulin beta-7 chain | chr2:126	1445
Dark-2	138	Background	AT2G29550	Symbols: TUB7 | tubulin beta-7 chain | chr2:126	1445
Dark-2	138	Background	AT2G29550	Symbols: TUB7 | tubulin beta-7 chain | chr2:126	1445
Dark-2	138	Background	AT2G29550	Symbols: TUB7 | tubulin beta-7 chain | chr2:126	1445
Dark-2	138	Background	AT2G29550	Symbols: TUB7 | tubulin beta-7 chain | chr2:126	1445
Dark-2	138	Background	AT2G29550	Symbols: TUB7 | tubulin beta-7 chain | chr2:126	1445
Dark-2	139	Background	AT4G20890	Symbols: TUB9 | tubulin beta-9 chain | chr4:111	1424
Dark-2	139	Background	AT4G20890	Symbols: TUB9 | tubulin beta-9 chain | chr4:111	1424
Dark-2	139	Background	AT4G20890	Symbols: TUB9 | tubulin beta-9 chain | chr4:111	1424
Dark-2	139	Background	AT4G20890	Symbols: TUB9 | tubulin beta-9 chain | chr4:111	1424
Dark-2	139	Background	AT4G20890	Symbols: TUB9 | tubulin beta-9 chain | chr4:111	1424
Dark-2	139	Background	AT4G20890	Symbols: TUB9 | tubulin beta-9 chain | chr4:111	1424
Dark-2	139	Background	AT4G20890	Symbols: TUB9 | tubulin beta-9 chain | chr4:111	1424
Dark-2	139	Background	AT4G20890	Symbols: TUB9 | tubulin beta-9 chain | chr4:111	1424
Dark-2	139	Background	AT4G20890	Symbols: TUB9 | tubulin beta-9 chain | chr4:111	1424
Dark-2	139	Background	AT4G20890	Symbols: TUB9 | tubulin beta-9 chain | chr4:111	1424
Dark-2	139	Background	AT4G20890	Symbols: TUB9 | tubulin beta-9 chain | chr4:111	1424
Dark-2	139	Background	AT4G20890	Symbols: TUB9 | tubulin beta-9 chain | chr4:111	1424
Dark-2	139	Background	AT4G20890	Symbols: TUB9 | tubulin beta-9 chain | chr4:111	1424
Dark-2	139	Background	AT4G20890	Symbols: TUB9 | tubulin beta-9 chain | chr4:111	1424
Dark-2	139	Background	AT4G20890	Symbols: TUB9 | tubulin beta-9 chain | chr4:111	1424
Dark-2	139	Background	AT4G20890	Symbols: TUB9 | tubulin beta-9 chain | chr4:111	1424
Dark-2	139	Background	AT4G20890	Symbols: TUB9 | tubulin beta-9 chain | chr4:111	1424
Dark-2	139	Background	AT4G20890	Symbols: TUB9 | tubulin beta-9 chain | chr4:111	1424
Dark-2	139	Background	AT4G20890	Symbols: TUB9 | tubulin beta-9 chain | chr4:111	1424
Dark-2	140	Background	AT1G04820	Symbols: TUA4, TOR2 | tubulin alpha-4 chain | c	1321
Dark-2	140	Background	AT1G04820	Symbols: TUA4, TOR2 | tubulin alpha-4 chain | c	1321
Dark-2	140	Background	AT1G04820	Symbols: TUA4, TOR2 | tubulin alpha-4 chain | c	1321
Dark-2	140	Background	AT1G04820	Symbols: TUA4, TOR2 | tubulin alpha-4 chain | c	1321
Dark-2	140	Background	AT1G04820	Symbols: TUA4, TOR2 | tubulin alpha-4 chain | c	1321
Dark-2	140	Background	AT1G04820	Symbols: TUA4, TOR2 | tubulin alpha-4 chain | c	1321
Dark-2	140	Background	AT1G04820	Symbols: TUA4, TOR2 | tubulin alpha-4 chain | c	1321
Dark-2	140	Background	AT1G04820	Symbols: TUA4, TOR2 | tubulin alpha-4 chain | c	1321
Dark-2	140	Background	AT1G04820	Symbols: TUA4, TOR2 | tubulin alpha-4 chain | c	1321
Dark-2	140	Background	AT1G04820	Symbols: TUA4, TOR2 | tubulin alpha-4 chain | c	1321
Dark-2	140	Background	AT1G04820	Symbols: TUA4, TOR2 | tubulin alpha-4 chain | c	1321
Dark-2	140	Background	AT1G04820	Symbols: TUA4, TOR2 | tubulin alpha-4 chain | c	1321
Dark-2	140	Background	AT1G04820	Symbols: TUA4, TOR2 | tubulin alpha-4 chain | c	1321
Dark-2	140	Background	AT1G04820	Symbols: TUA4, TOR2 | tubulin alpha-4 chain | c	1321

Dark-2	140	Background	AT1G04820	Symbols: TUA4, TOR2 | tubulin alpha-4 chain | c	1321
Dark-2	140	Background	AT1G04820	Symbols: TUA4, TOR2 | tubulin alpha-4 chain | c	1321
Dark-2	140	Background	AT1G04820	Symbols: TUA4, TOR2 | tubulin alpha-4 chain | c	1321
Dark-2	140	Background	AT1G04820	Symbols: TUA4, TOR2 | tubulin alpha-4 chain | c	1321
Dark-2	140	Background	AT1G04820	Symbols: TUA4, TOR2 | tubulin alpha-4 chain | c	1321
Dark-2	140	Background	AT1G04820	Symbols: TUA4, TOR2 | tubulin alpha-4 chain | c	1321
Dark-2	141	Background	AT4G14960	Symbols: TUA6 | Tubulin/FtsZ family protein | cl	1318
Dark-2	141	Background	AT4G14960	Symbols: TUA6 | Tubulin/FtsZ family protein | cl	1318
Dark-2	141	Background	AT4G14960	Symbols: TUA6 | Tubulin/FtsZ family protein | cl	1318
Dark-2	141	Background	AT4G14960	Symbols: TUA6 | Tubulin/FtsZ family protein | cl	1318
Dark-2	141	Background	AT4G14960	Symbols: TUA6 | Tubulin/FtsZ family protein | cl	1318
Dark-2	141	Background	AT4G14960	Symbols: TUA6 | Tubulin/FtsZ family protein | cl	1318
Dark-2	141	Background	AT4G14960	Symbols: TUA6 | Tubulin/FtsZ family protein | cl	1318
Dark-2	141	Background	AT4G14960	Symbols: TUA6 | Tubulin/FtsZ family protein | cl	1318
Dark-2	141	Background	AT4G14960	Symbols: TUA6 | Tubulin/FtsZ family protein | cl	1318
Dark-2	141	Background	AT4G14960	Symbols: TUA6 | Tubulin/FtsZ family protein | cl	1318
Dark-2	141	Background	AT4G14960	Symbols: TUA6 | Tubulin/FtsZ family protein | cl	1318
Dark-2	141	Background	AT4G14960	Symbols: TUA6 | Tubulin/FtsZ family protein | cl	1318
Dark-2	141	Background	AT4G14960	Symbols: TUA6 | Tubulin/FtsZ family protein | cl	1318
Dark-2	141	Background	AT4G14960	Symbols: TUA6 | Tubulin/FtsZ family protein | cl	1318
Dark-2	141	Background	AT4G14960	Symbols: TUA6 | Tubulin/FtsZ family protein | cl	1318
Dark-2	141	Background	AT4G14960	Symbols: TUA6 | Tubulin/FtsZ family protein | cl	1318
Dark-2	141	Background	AT4G14960	Symbols: TUA6 | Tubulin/FtsZ family protein | cl	1318
Dark-2	141	Background	AT4G14960	Symbols: TUA6 | Tubulin/FtsZ family protein | cl	1318
Dark-2	141	Background	AT4G14960	Symbols: TUA6 | Tubulin/FtsZ family protein | cl	1318
Dark-2	141	Background	AT4G14960	Symbols: TUA6 | Tubulin/FtsZ family protein | cl	1318
Dark-2	142	Background	AT1G54270	Symbols: EIF4A-2 | eif4a-2 | chr1:20260495-202	1195
Dark-2	142	Background	AT1G54270	Symbols: EIF4A-2 | eif4a-2 | chr1:20260495-202	1195
Dark-2	142	Background	AT1G54270	Symbols: EIF4A-2 | eif4a-2 | chr1:20260495-202	1195
Dark-2	142	Background	AT1G54270	Symbols: EIF4A-2 | eif4a-2 | chr1:20260495-202	1195
Dark-2	142	Background	AT1G54270	Symbols: EIF4A-2 | eif4a-2 | chr1:20260495-202	1195
Dark-2	142	Background	AT1G54270	Symbols: EIF4A-2 | eif4a-2 | chr1:20260495-202	1195
Dark-2	142	Background	AT1G54270	Symbols: EIF4A-2 | eif4a-2 | chr1:20260495-202	1195
Dark-2	142	Background	AT1G54270	Symbols: EIF4A-2 | eif4a-2 | chr1:20260495-202	1195
Dark-2	142	Background	AT1G54270	Symbols: EIF4A-2 | eif4a-2 | chr1:20260495-202	1195
Dark-2	142	Background	AT1G54270	Symbols: EIF4A-2 | eif4a-2 | chr1:20260495-202	1195
Dark-2	142	Background	AT1G54270	Symbols: EIF4A-2 | eif4a-2 | chr1:20260495-202	1195
Dark-2	142	Background	AT1G54270	Symbols: EIF4A-2 | eif4a-2 | chr1:20260495-202	1195
Dark-2	142	Background	AT1G54270	Symbols: EIF4A-2 | eif4a-2 | chr1:20260495-202	1195
Dark-2	142	Background	AT1G54270	Symbols: EIF4A-2 | eif4a-2 | chr1:20260495-202	1195
Dark-2	142	Background	AT1G54270	Symbols: EIF4A-2 | eif4a-2 | chr1:20260495-202	1195
Dark-2	142	Background	AT1G54270	Symbols: EIF4A-2 | eif4a-2 | chr1:20260495-202	1195
Dark-2	142	Background	AT1G54270	Symbols: EIF4A-2 | eif4a-2 | chr1:20260495-202	1195
Dark-2	142	Background	AT1G54270	Symbols: EIF4A-2 | eif4a-2 | chr1:20260495-202	1195
Dark-2	142	Background	AT1G54270	Symbols: EIF4A-2 | eif4a-2 | chr1:20260495-202	1195
Dark-2	142	Background	AT1G54270	Symbols: EIF4A-2 | eif4a-2 | chr1:20260495-202	1195
Dark-2	142	Background	AT1G54270	Symbols: EIF4A-2 | eif4a-2 | chr1:20260495-202	1195
Dark-2	143	Background	AT3G13920	Symbols: EIF4A1, RH4, TIF4A1 | eukaryotic trans	1088
Dark-2	143	Background	AT3G13920	Symbols: EIF4A1, RH4, TIF4A1 | eukaryotic trans	1088
Dark-2	143	Background	AT3G13920	Symbols: EIF4A1, RH4, TIF4A1 | eukaryotic trans	1088
Dark-2	143	Background	AT3G13920	Symbols: EIF4A1, RH4, TIF4A1 | eukaryotic trans	1088
Dark-2	143	Background	AT3G13920	Symbols: EIF4A1, RH4, TIF4A1 | eukaryotic trans	1088
Dark-2	143	Background	AT3G13920	Symbols: EIF4A1, RH4, TIF4A1 | eukaryotic trans	1088
Dark-2	143	Background	AT3G13920	Symbols: EIF4A1, RH4, TIF4A1 | eukaryotic trans	1088
Dark-2	143	Background	AT3G13920	Symbols: EIF4A1, RH4, TIF4A1 | eukaryotic trans	1088
Dark-2	143	Background	AT3G13920	Symbols: EIF4A1, RH4, TIF4A1 | eukaryotic trans	1088
Dark-2	143	Background	AT3G13920	Symbols: EIF4A1, RH4, TIF4A1 | eukaryotic trans	1088
Dark-2	143	Background	AT3G13920	Symbols: EIF4A1, RH4, TIF4A1 | eukaryotic trans	1088

Dark-2	143	Background	AT3G13920	Symbols: EIF4A1, RH4, TIF4A1 | eukaryotic trans	1088
Dark-2	143	Background	AT3G13920	Symbols: EIF4A1, RH4, TIF4A1 | eukaryotic trans	1088
Dark-2	143	Background	AT3G13920	Symbols: EIF4A1, RH4, TIF4A1 | eukaryotic trans	1088
Dark-2	143	Background	AT3G13920	Symbols: EIF4A1, RH4, TIF4A1 | eukaryotic trans	1088
Dark-2	143	Background	AT3G13920	Symbols: EIF4A1, RH4, TIF4A1 | eukaryotic trans	1088
Dark-2	143	Background	AT3G13920	Symbols: EIF4A1, RH4, TIF4A1 | eukaryotic trans	1088
Dark-2	143	Background	AT3G13920	Symbols: EIF4A1, RH4, TIF4A1 | eukaryotic trans	1088
Dark-2	143	Background	AT3G13920	Symbols: EIF4A1, RH4, TIF4A1 | eukaryotic trans	1088
Dark-2	143	Background	AT3G13920	Symbols: EIF4A1, RH4, TIF4A1 | eukaryotic trans	1088
Dark-2	143	Background	AT3G13920	Symbols: EIF4A1, RH4, TIF4A1 | eukaryotic trans	1088
Dark-2	144	Background	AT5G02500	Symbols: HSC70-1, HSP70-1, AT-HSC70-1, HSC70	1078
Dark-2	144	Background	AT5G02500	Symbols: HSC70-1, HSP70-1, AT-HSC70-1, HSC70	1078
Dark-2	144	Background	AT5G02500	Symbols: HSC70-1, HSP70-1, AT-HSC70-1, HSC70	1078
Dark-2	144	Background	AT5G02500	Symbols: HSC70-1, HSP70-1, AT-HSC70-1, HSC70	1078
Dark-2	144	Background	AT5G02500	Symbols: HSC70-1, HSP70-1, AT-HSC70-1, HSC70	1078
Dark-2	144	Background	AT5G02500	Symbols: HSC70-1, HSP70-1, AT-HSC70-1, HSC70	1078
Dark-2	144	Background	AT5G02500	Symbols: HSC70-1, HSP70-1, AT-HSC70-1, HSC70	1078
Dark-2	144	Background	AT5G02500	Symbols: HSC70-1, HSP70-1, AT-HSC70-1, HSC70	1078
Dark-2	144	Background	AT5G02500	Symbols: HSC70-1, HSP70-1, AT-HSC70-1, HSC70	1078
Dark-2	144	Background	AT5G02500	Symbols: HSC70-1, HSP70-1, AT-HSC70-1, HSC70	1078
Dark-2	144	Background	AT5G02500	Symbols: HSC70-1, HSP70-1, AT-HSC70-1, HSC70	1078
Dark-2	144	Background	AT5G02500	Symbols: HSC70-1, HSP70-1, AT-HSC70-1, HSC70	1078
Dark-2	144	Background	AT5G02500	Symbols: HSC70-1, HSP70-1, AT-HSC70-1, HSC70	1078
Dark-2	144	Background	AT5G02500	Symbols: HSC70-1, HSP70-1, AT-HSC70-1, HSC70	1078
Dark-2	144	Background	AT5G02500	Symbols: HSC70-1, HSP70-1, AT-HSC70-1, HSC70	1078
Dark-2	144	Background	AT5G02500	Symbols: HSC70-1, HSP70-1, AT-HSC70-1, HSC70	1078
Dark-2	144	Background	AT5G02500	Symbols: HSC70-1, HSP70-1, AT-HSC70-1, HSC70	1078
Dark-2	144	Background	AT5G02500	Symbols: HSC70-1, HSP70-1, AT-HSC70-1, HSC70	1078
Dark-2	144	Background	AT5G02500	Symbols: HSC70-1, HSP70-1, AT-HSC70-1, HSC70	1078
Dark-2	144	Background	AT5G02500	Symbols: HSC70-1, HSP70-1, AT-HSC70-1, HSC70	1078
Dark-2	144	Background	AT5G02500	Symbols: HSC70-1, HSP70-1, AT-HSC70-1, HSC70	1078
Dark-2	144	Background	AT5G02500	Symbols: HSC70-1, HSP70-1, AT-HSC70-1, HSC70	1078
Dark-2	145	Background	AT1G67120	Symbols: | ATPases;nucleotide binding;ATP binc	1036
Dark-2	145	Background	AT1G67120	Symbols: | ATPases;nucleotide binding;ATP binc	1036
Dark-2	145	Background	AT1G67120	Symbols: | ATPases;nucleotide binding;ATP binc	1036
Dark-2	145	Background	AT1G67120	Symbols: | ATPases;nucleotide binding;ATP binc	1036
Dark-2	145	Background	AT1G67120	Symbols: | ATPases;nucleotide binding;ATP binc	1036
Dark-2	145	Background	AT1G67120	Symbols: | ATPases;nucleotide binding;ATP binc	1036
Dark-2	145	Background	AT1G67120	Symbols: | ATPases;nucleotide binding;ATP binc	1036
Dark-2	145	Background	AT1G67120	Symbols: | ATPases;nucleotide binding;ATP binc	1036
Dark-2	145	Background	AT1G67120	Symbols: | ATPases;nucleotide binding;ATP binc	1036
Dark-2	145	Background	AT1G67120	Symbols: | ATPases;nucleotide binding;ATP binc	1036
Dark-2	145	Background	AT1G67120	Symbols: | ATPases;nucleotide binding;ATP binc	1036
Dark-2	145	Background	AT1G67120	Symbols: | ATPases;nucleotide binding;ATP binc	1036
Dark-2	145	Background	AT1G67120	Symbols: | ATPases;nucleotide binding;ATP binc	1036
Dark-2	145	Background	AT1G67120	Symbols: | ATPases;nucleotide binding;ATP binc	1036
Dark-2	145	Background	AT1G67120	Symbols: | ATPases;nucleotide binding;ATP binc	1036
Dark-2	145	Background	AT1G67120	Symbols: | ATPases;nucleotide binding;ATP binc	1036
Dark-2	145	Background	AT1G67120	Symbols: | ATPases;nucleotide binding;ATP binc	1036
Dark-2	145	Background	AT1G67120	Symbols: | ATPases;nucleotide binding;ATP binc	1036
Dark-2	145	Background	AT1G67120	Symbols: | ATPases;nucleotide binding;ATP binc	1036
Dark-2	145	Background	AT1G67120	Symbols: | ATPases;nucleotide binding;ATP binc	1036
Dark-2	145	Background	AT1G67120	Symbols: | ATPases;nucleotide binding;ATP binc	1036
Dark-2	145	Background	AT1G67120	Symbols: | ATPases;nucleotide binding;ATP binc	1036
Dark-2	146	Background	AT3G09440	Symbols: | Heat shock protein 70 (Hsp 70) famil	1003
Dark-2	146	Background	AT3G09440	Symbols: | Heat shock protein 70 (Hsp 70) famil	1003
Dark-2	146	Background	AT3G09440	Symbols: | Heat shock protein 70 (Hsp 70) famil	1003
Dark-2	146	Background	AT3G09440	Symbols: | Heat shock protein 70 (Hsp 70) famil	1003

Dark-2	146	Background	AT3G09440	Symbols: | Heat shock protein 70 (Hsp 70) famil	1003
Dark-2	146	Background	AT3G09440	Symbols: | Heat shock protein 70 (Hsp 70) famil	1003
Dark-2	146	Background	AT3G09440	Symbols: | Heat shock protein 70 (Hsp 70) famil	1003
Dark-2	146	Background	AT3G09440	Symbols: | Heat shock protein 70 (Hsp 70) famil	1003
Dark-2	146	Background	AT3G09440	Symbols: | Heat shock protein 70 (Hsp 70) famil	1003
Dark-2	146	Background	AT3G09440	Symbols: | Heat shock protein 70 (Hsp 70) famil	1003
Dark-2	146	Background	AT3G09440	Symbols: | Heat shock protein 70 (Hsp 70) famil	1003
Dark-2	146	Background	AT3G09440	Symbols: | Heat shock protein 70 (Hsp 70) famil	1003
Dark-2	146	Background	AT3G09440	Symbols: | Heat shock protein 70 (Hsp 70) famil	1003
Dark-2	146	Background	AT3G09440	Symbols: | Heat shock protein 70 (Hsp 70) famil	1003
Dark-2	146	Background	AT3G09440	Symbols: | Heat shock protein 70 (Hsp 70) famil	1003
Dark-2	146	Background	AT3G09440	Symbols: | Heat shock protein 70 (Hsp 70) famil	1003
Dark-2	146	Background	AT3G09440	Symbols: | Heat shock protein 70 (Hsp 70) famil	1003
Dark-2	146	Background	AT3G09440	Symbols: | Heat shock protein 70 (Hsp 70) famil	1003
Dark-2	146	Background	AT3G09440	Symbols: | Heat shock protein 70 (Hsp 70) famil	1003
Dark-2	146	Background	AT3G09440	Symbols: | Heat shock protein 70 (Hsp 70) famil	1003
Dark-2	147	Background	AT5G12250	Symbols: TUB6 | beta-6 tubulin | chr5:3961317-	915
Dark-2	147	Background	AT5G12250	Symbols: TUB6 | beta-6 tubulin | chr5:3961317-	915
Dark-2	147	Background	AT5G12250	Symbols: TUB6 | beta-6 tubulin | chr5:3961317-	915
Dark-2	147	Background	AT5G12250	Symbols: TUB6 | beta-6 tubulin | chr5:3961317-	915
Dark-2	147	Background	AT5G12250	Symbols: TUB6 | beta-6 tubulin | chr5:3961317-	915
Dark-2	147	Background	AT5G12250	Symbols: TUB6 | beta-6 tubulin | chr5:3961317-	915
Dark-2	147	Background	AT5G12250	Symbols: TUB6 | beta-6 tubulin | chr5:3961317-	915
Dark-2	147	Background	AT5G12250	Symbols: TUB6 | beta-6 tubulin | chr5:3961317-	915
Dark-2	147	Background	AT5G12250	Symbols: TUB6 | beta-6 tubulin | chr5:3961317-	915
Dark-2	147	Background	AT5G12250	Symbols: TUB6 | beta-6 tubulin | chr5:3961317-	915
Dark-2	147	Background	AT5G12250	Symbols: TUB6 | beta-6 tubulin | chr5:3961317-	915
Dark-2	147	Background	AT5G12250	Symbols: TUB6 | beta-6 tubulin | chr5:3961317-	915
Dark-2	147	Background	AT5G12250	Symbols: TUB6 | beta-6 tubulin | chr5:3961317-	915
Dark-2	147	Background	AT5G12250	Symbols: TUB6 | beta-6 tubulin | chr5:3961317-	915
Dark-2	147	Background	AT5G12250	Symbols: TUB6 | beta-6 tubulin | chr5:3961317-	915
Dark-2	147	Background	AT5G12250	Symbols: TUB6 | beta-6 tubulin | chr5:3961317-	915
Dark-2	148	Background	AT1G64790	Symbols: ILA | ILITYHIA | chr1:24065232-24081[illegible]	872
Dark-2	148	Background	AT1G64790	Symbols: ILA | ILITYHIA | chr1:24065232-24081[illegible]	872
Dark-2	148	Background	AT1G64790	Symbols: ILA | ILITYHIA | chr1:24065232-24081[illegible]	872
Dark-2	148	Background	AT1G64790	Symbols: ILA | ILITYHIA | chr1:24065232-24081[illegible]	872
Dark-2	148	Background	AT1G64790	Symbols: ILA | ILITYHIA | chr1:24065232-24081[illegible]	872
Dark-2	148	Background	AT1G64790	Symbols: ILA | ILITYHIA | chr1:24065232-24081[illegible]	872
Dark-2	148	Background	AT1G64790	Symbols: ILA | ILITYHIA | chr1:24065232-24081[illegible]	872
Dark-2	148	Background	AT1G64790	Symbols: ILA | ILITYHIA | chr1:24065232-24081[illegible]	872
Dark-2	148	Background	AT1G64790	Symbols: ILA | ILITYHIA | chr1:24065232-24081[illegible]	872
Dark-2	148	Background	AT1G64790	Symbols: ILA | ILITYHIA | chr1:24065232-24081[illegible]	872
Dark-2	148	Background	AT1G64790	Symbols: ILA | ILITYHIA | chr1:24065232-24081[illegible]	872
Dark-2	148	Background	AT1G64790	Symbols: ILA | ILITYHIA | chr1:24065232-24081[illegible]	872
Dark-2	148	Background	AT1G64790	Symbols: ILA | ILITYHIA | chr1:24065232-24081[illegible]	872
Dark-2	148	Background	AT1G64790	Symbols: ILA | ILITYHIA | chr1:24065232-24081[illegible]	872
Dark-2	148	Background	AT1G64790	Symbols: ILA | ILITYHIA | chr1:24065232-24081[illegible]	872
Dark-2	148	Background	AT1G64790	Symbols: ILA | ILITYHIA | chr1:24065232-24081[illegible]	872
Dark-2	149	Background	AT1G75780	Symbols: TUB1 | tubulin beta-1 chain | chr1:284	857
Dark-2	149	Background	AT1G75780	Symbols: TUB1 | tubulin beta-1 chain | chr1:284	857
Dark-2	149	Background	AT1G75780	Symbols: TUB1 | tubulin beta-1 chain | chr1:284	857
Dark-2	149	Background	AT1G75780	Symbols: TUB1 | tubulin beta-1 chain | chr1:284	857
Dark-2	149	Background	AT1G75780	Symbols: TUB1 | tubulin beta-1 chain | chr1:284	857
Dark-2	149	Background	AT1G75780	Symbols: TUB1 | tubulin beta-1 chain | chr1:284	857
Dark-2	149	Background	AT1G75780	Symbols: TUB1 | tubulin beta-1 chain | chr1:284	857
Dark-2	149	Background	AT1G75780	Symbols: TUB1 | tubulin beta-1 chain | chr1:284	857
Dark-2	149	Background	AT1G75780	Symbols: TUB1 | tubulin beta-1 chain | chr1:284	857
Dark-2	149	Background	AT1G75780	Symbols: TUB1 | tubulin beta-1 chain | chr1:284	857

Dark-2	149	Background	AT1G75780	Symbols: TUB1 | tubulin beta-1 chain | chr1:284	857
Dark-2	149	Background	AT1G75780	Symbols: TUB1 | tubulin beta-1 chain | chr1:284	857
Dark-2	149	Background	AT1G75780	Symbols: TUB1 | tubulin beta-1 chain | chr1:284	857
Dark-2	150	Background	AT2G02560	Symbols: CAND1, ATCAND1, ETA2, TIP120, HVE	854
Dark-2	150	Background	AT2G02560	Symbols: CAND1, ATCAND1, ETA2, TIP120, HVE	854
Dark-2	150	Background	AT2G02560	Symbols: CAND1, ATCAND1, ETA2, TIP120, HVE	854
Dark-2	150	Background	AT2G02560	Symbols: CAND1, ATCAND1, ETA2, TIP120, HVE	854
Dark-2	150	Background	AT2G02560	Symbols: CAND1, ATCAND1, ETA2, TIP120, HVE	854
Dark-2	150	Background	AT2G02560	Symbols: CAND1, ATCAND1, ETA2, TIP120, HVE	854
Dark-2	150	Background	AT2G02560	Symbols: CAND1, ATCAND1, ETA2, TIP120, HVE	854
Dark-2	150	Background	AT2G02560	Symbols: CAND1, ATCAND1, ETA2, TIP120, HVE	854
Dark-2	150	Background	AT2G02560	Symbols: CAND1, ATCAND1, ETA2, TIP120, HVE	854
Dark-2	150	Background	AT2G02560	Symbols: CAND1, ATCAND1, ETA2, TIP120, HVE	854
Dark-2	150	Background	AT2G02560	Symbols: CAND1, ATCAND1, ETA2, TIP120, HVE	854
Dark-2	150	Background	AT2G02560	Symbols: CAND1, ATCAND1, ETA2, TIP120, HVE	854
Dark-2	150	Background	AT2G02560	Symbols: CAND1, ATCAND1, ETA2, TIP120, HVE	854
Dark-2	150	Background	AT2G02560	Symbols: CAND1, ATCAND1, ETA2, TIP120, HVE	854
Dark-2	150	Background	AT2G02560	Symbols: CAND1, ATCAND1, ETA2, TIP120, HVE	854
Dark-2	150	Background	AT2G02560	Symbols: CAND1, ATCAND1, ETA2, TIP120, HVE	854
Dark-2	150	Background	AT2G02560	Symbols: CAND1, ATCAND1, ETA2, TIP120, HVE	854
Dark-2	150	Background	AT2G02560	Symbols: CAND1, ATCAND1, ETA2, TIP120, HVE	854
Dark-2	151	Background	AT4G20360	Symbols: ATRAB8D, ATRABE1B, RABE1b | RAB G	837
Dark-2	151	Background	AT4G20360	Symbols: ATRAB8D, ATRABE1B, RABE1b | RAB G	837
Dark-2	151	Background	AT4G20360	Symbols: ATRAB8D, ATRABE1B, RABE1b | RAB G	837
Dark-2	151	Background	AT4G20360	Symbols: ATRAB8D, ATRABE1B, RABE1b | RAB G	837
Dark-2	151	Background	AT4G20360	Symbols: ATRAB8D, ATRABE1B, RABE1b | RAB G	837
Dark-2	151	Background	AT4G20360	Symbols: ATRAB8D, ATRABE1B, RABE1b | RAB G	837
Dark-2	151	Background	AT4G20360	Symbols: ATRAB8D, ATRABE1B, RABE1b | RAB G	837
Dark-2	151	Background	AT4G20360	Symbols: ATRAB8D, ATRABE1B, RABE1b | RAB G	837
Dark-2	151	Background	AT4G20360	Symbols: ATRAB8D, ATRABE1B, RABE1b | RAB G	837
Dark-2	151	Background	AT4G20360	Symbols: ATRAB8D, ATRABE1B, RABE1b | RAB G	837
Dark-2	151	Background	AT4G20360	Symbols: ATRAB8D, ATRABE1B, RABE1b | RAB G	837
Dark-2	151	Background	AT4G20360	Symbols: ATRAB8D, ATRABE1B, RABE1b | RAB G	837
Dark-2	151	Background	AT4G20360	Symbols: ATRAB8D, ATRABE1B, RABE1b | RAB G	837
Dark-2	151	Background	AT4G20360	Symbols: ATRAB8D, ATRABE1B, RABE1b | RAB G	837
Dark-2	152	Background	AT5G19770	Symbols: TUA3 | tubulin alpha-3 | chr5:6682761	822
Dark-2	152	Background	AT5G19770	Symbols: TUA3 | tubulin alpha-3 | chr5:6682761	822
Dark-2	152	Background	AT5G19770	Symbols: TUA3 | tubulin alpha-3 | chr5:6682761	822
Dark-2	152	Background	AT5G19770	Symbols: TUA3 | tubulin alpha-3 | chr5:6682761	822
Dark-2	152	Background	AT5G19770	Symbols: TUA3 | tubulin alpha-3 | chr5:6682761	822
Dark-2	152	Background	AT5G19770	Symbols: TUA3 | tubulin alpha-3 | chr5:6682761	822
Dark-2	152	Background	AT5G19770	Symbols: TUA3 | tubulin alpha-3 | chr5:6682761	822
Dark-2	152	Background	AT5G19770	Symbols: TUA3 | tubulin alpha-3 | chr5:6682761	822
Dark-2	152	Background	AT5G19770	Symbols: TUA3 | tubulin alpha-3 | chr5:6682761	822
Dark-2	152	Background	AT5G19770	Symbols: TUA3 | tubulin alpha-3 | chr5:6682761	822
Dark-2	152	Background	AT5G19770	Symbols: TUA3 | tubulin alpha-3 | chr5:6682761	822
Dark-2	152	Background	AT5G19770	Symbols: TUA3 | tubulin alpha-3 | chr5:6682761	822
Dark-2	152	Background	AT5G19770	Symbols: TUA3 | tubulin alpha-3 | chr5:6682761	822
Dark-2	152	Background	AT5G19770	Symbols: TUA3 | tubulin alpha-3 | chr5:6682761	822
Dark-2	152	Background	AT5G19770	Symbols: TUA3 | tubulin alpha-3 | chr5:6682761	822
Dark-2	152	Background	AT5G19770	Symbols: TUA3 | tubulin alpha-3 | chr5:6682761	822
Dark-2	153	Background	AT2G20580	Symbols: RPN1A, ATRPN1A | 26S proteasome re	754
Dark-2	153	Background	AT2G20580	Symbols: RPN1A, ATRPN1A | 26S proteasome re	754
Dark-2	153	Background	AT2G20580	Symbols: RPN1A, ATRPN1A | 26S proteasome re	754
Dark-2	153	Background	AT2G20580	Symbols: RPN1A, ATRPN1A | 26S proteasome re	754
Dark-2	153	Background	AT2G20580	Symbols: RPN1A, ATRPN1A | 26S proteasome re	754
Dark-2	153	Background	AT2G20580	Symbols: RPN1A, ATRPN1A | 26S proteasome re	754

Dark-2	153	Background	AT2G20580	Symbols: RPN1A, ATRPN1A | 26S proteasome re	754
Dark-2	153	Background	AT2G20580	Symbols: RPN1A, ATRPN1A | 26S proteasome re	754
Dark-2	153	Background	AT2G20580	Symbols: RPN1A, ATRPN1A | 26S proteasome re	754
Dark-2	153	Background	AT2G20580	Symbols: RPN1A, ATRPN1A | 26S proteasome re	754
Dark-2	153	Background	AT2G20580	Symbols: RPN1A, ATRPN1A | 26S proteasome re	754
Dark-2	153	Background	AT2G20580	Symbols: RPN1A, ATRPN1A | 26S proteasome re	754
Dark-2	153	Background	AT2G20580	Symbols: RPN1A, ATRPN1A | 26S proteasome re	754
Dark-2	153	Background	AT2G20580	Symbols: RPN1A, ATRPN1A | 26S proteasome re	754
Dark-2	153	Background	AT2G20580	Symbols: RPN1A, ATRPN1A | 26S proteasome re	754
Dark-2	153	Background	AT2G20580	Symbols: RPN1A, ATRPN1A | 26S proteasome re	754
Dark-2	153	Background	AT2G20580	Symbols: RPN1A, ATRPN1A | 26S proteasome re	754
Dark-2	154	Background	AT1G13440	Symbols: GAPC-2, GAPC2 | glyceraldehyde-3-ph	744
Dark-2	154	Background	AT1G13440	Symbols: GAPC-2, GAPC2 | glyceraldehyde-3-ph	744
Dark-2	154	Background	AT1G13440	Symbols: GAPC-2, GAPC2 | glyceraldehyde-3-ph	744
Dark-2	154	Background	AT1G13440	Symbols: GAPC-2, GAPC2 | glyceraldehyde-3-ph	744
Dark-2	154	Background	AT1G13440	Symbols: GAPC-2, GAPC2 | glyceraldehyde-3-ph	744
Dark-2	154	Background	AT1G13440	Symbols: GAPC-2, GAPC2 | glyceraldehyde-3-ph	744
Dark-2	154	Background	AT1G13440	Symbols: GAPC-2, GAPC2 | glyceraldehyde-3-ph	744
Dark-2	154	Background	AT1G13440	Symbols: GAPC-2, GAPC2 | glyceraldehyde-3-ph	744
Dark-2	154	Background	AT1G13440	Symbols: GAPC-2, GAPC2 | glyceraldehyde-3-ph	744
Dark-2	154	Background	AT1G13440	Symbols: GAPC-2, GAPC2 | glyceraldehyde-3-ph	744
Dark-2	154	Background	AT1G13440	Symbols: GAPC-2, GAPC2 | glyceraldehyde-3-ph	744
Dark-2	154	Background	AT1G13440	Symbols: GAPC-2, GAPC2 | glyceraldehyde-3-ph	744
Dark-2	155	Background	AT1G07920	Symbols: | GTP binding Elongation factor Tu fan	736
Dark-2	155	Background	AT1G07920	Symbols: | GTP binding Elongation factor Tu fan	736
Dark-2	155	Background	AT1G07920	Symbols: | GTP binding Elongation factor Tu fan	736
Dark-2	155	Background	AT1G07920	Symbols: | GTP binding Elongation factor Tu fan	736
Dark-2	155	Background	AT1G07920	Symbols: | GTP binding Elongation factor Tu fan	736
Dark-2	155	Background	AT1G07920	Symbols: | GTP binding Elongation factor Tu fan	736
Dark-2	155	Background	AT1G07920	Symbols: | GTP binding Elongation factor Tu fan	736
Dark-2	155	Background	AT1G07920	Symbols: | GTP binding Elongation factor Tu fan	736
Dark-2	155	Background	AT1G07920	Symbols: | GTP binding Elongation factor Tu fan	736
Dark-2	155	Background	AT1G07920	Symbols: | GTP binding Elongation factor Tu fan	736
Dark-2	155	Background	AT1G07920	Symbols: | GTP binding Elongation factor Tu fan	736
Dark-2	155	Background	AT1G07920	Symbols: | GTP binding Elongation factor Tu fan	736
Dark-2	155	Background	AT1G07920	Symbols: | GTP binding Elongation factor Tu fan	736
Dark-2	156	Background	AT1G72730	Symbols: | DEA(D/H)-box RNA helicase family p	727
Dark-2	156	Background	AT1G72730	Symbols: | DEA(D/H)-box RNA helicase family p	727
Dark-2	156	Background	AT1G72730	Symbols: | DEA(D/H)-box RNA helicase family p	727
Dark-2	156	Background	AT1G72730	Symbols: | DEA(D/H)-box RNA helicase family p	727
Dark-2	156	Background	AT1G72730	Symbols: | DEA(D/H)-box RNA helicase family p	727
Dark-2	156	Background	AT1G72730	Symbols: | DEA(D/H)-box RNA helicase family p	727
Dark-2	156	Background	AT1G72730	Symbols: | DEA(D/H)-box RNA helicase family p	727
Dark-2	156	Background	AT1G72730	Symbols: | DEA(D/H)-box RNA helicase family p	727
Dark-2	156	Background	AT1G72730	Symbols: | DEA(D/H)-box RNA helicase family p	727
Dark-2	156	Background	AT1G72730	Symbols: | DEA(D/H)-box RNA helicase family p	727
Dark-2	156	Background	AT1G72730	Symbols: | DEA(D/H)-box RNA helicase family p	727
Dark-2	156	Background	AT1G72730	Symbols: | DEA(D/H)-box RNA helicase family p	727
Dark-2	156	Background	AT1G72730	Symbols: | DEA(D/H)-box RNA helicase family p	727
Dark-2	156	Background	AT1G72730	Symbols: | DEA(D/H)-box RNA helicase family p	727
Dark-2	157	Background	AT3G05530	Symbols: RPT5A, ATS6A.2 | regulatory particle tr	700
Dark-2	157	Background	AT3G05530	Symbols: RPT5A, ATS6A.2 | regulatory particle tr	700
Dark-2	157	Background	AT3G05530	Symbols: RPT5A, ATS6A.2 | regulatory particle tr	700
Dark-2	157	Background	AT3G05530	Symbols: RPT5A, ATS6A.2 | regulatory particle tr	700
Dark-2	157	Background	AT3G05530	Symbols: RPT5A, ATS6A.2 | regulatory particle tr	700
Dark-2	157	Background	AT3G05530	Symbols: RPT5A, ATS6A.2 | regulatory particle tr	700
Dark-2	157	Background	AT3G05530	Symbols: RPT5A, ATS6A.2 | regulatory particle tr	700
Dark-2	157	Background	AT3G05530	Symbols: RPT5A, ATS6A.2 | regulatory particle tr	700

Dark-2	157	Background	AT3G05530	Symbols: RPT5A, ATS6A.2 | regulatory particle tr	700
Dark-2	157	Background	AT3G05530	Symbols: RPT5A, ATS6A.2 | regulatory particle tr	700
Dark-2	157	Background	AT3G05530	Symbols: RPT5A, ATS6A.2 | regulatory particle tr	700
Dark-2	157	Background	AT3G05530	Symbols: RPT5A, ATS6A.2 | regulatory particle tr	700
Dark-2	157	Background	AT3G05530	Symbols: RPT5A, ATS6A.2 | regulatory particle tr	700
Dark-2	157	Background	AT3G05530	Symbols: RPT5A, ATS6A.2 | regulatory particle tr	700
Dark-2	157	Background	AT3G05530	Symbols: RPT5A, ATS6A.2 | regulatory particle tr	700
Dark-2	157	Background	AT3G05530	Symbols: RPT5A, ATS6A.2 | regulatory particle tr	700
Dark-2	158	Background	AT3G12580	Symbols: HSP70, ATHSP70 | heat shock protein	674
Dark-2	158	Background	AT3G12580	Symbols: HSP70, ATHSP70 | heat shock protein	674
Dark-2	158	Background	AT3G12580	Symbols: HSP70, ATHSP70 | heat shock protein	674
Dark-2	158	Background	AT3G12580	Symbols: HSP70, ATHSP70 | heat shock protein	674
Dark-2	158	Background	AT3G12580	Symbols: HSP70, ATHSP70 | heat shock protein	674
Dark-2	158	Background	AT3G12580	Symbols: HSP70, ATHSP70 | heat shock protein	674
Dark-2	158	Background	AT3G12580	Symbols: HSP70, ATHSP70 | heat shock protein	674
Dark-2	158	Background	AT3G12580	Symbols: HSP70, ATHSP70 | heat shock protein	674
Dark-2	158	Background	AT3G12580	Symbols: HSP70, ATHSP70 | heat shock protein	674
Dark-2	158	Background	AT3G12580	Symbols: HSP70, ATHSP70 | heat shock protein	674
Dark-2	158	Background	AT3G12580	Symbols: HSP70, ATHSP70 | heat shock protein	674
Dark-2	158	Background	AT3G12580	Symbols: HSP70, ATHSP70 | heat shock protein	674
Dark-2	159	Background	AT5G02490	Symbols: | Heat shock protein 70 (Hsp 70) famil	659
Dark-2	159	Background	AT5G02490	Symbols: | Heat shock protein 70 (Hsp 70) famil	659
Dark-2	159	Background	AT5G02490	Symbols: | Heat shock protein 70 (Hsp 70) famil	659
Dark-2	159	Background	AT5G02490	Symbols: | Heat shock protein 70 (Hsp 70) famil	659
Dark-2	159	Background	AT5G02490	Symbols: | Heat shock protein 70 (Hsp 70) famil	659
Dark-2	159	Background	AT5G02490	Symbols: | Heat shock protein 70 (Hsp 70) famil	659
Dark-2	159	Background	AT5G02490	Symbols: | Heat shock protein 70 (Hsp 70) famil	659
Dark-2	159	Background	AT5G02490	Symbols: | Heat shock protein 70 (Hsp 70) famil	659
Dark-2	159	Background	AT5G02490	Symbols: | Heat shock protein 70 (Hsp 70) famil	659
Dark-2	159	Background	AT5G02490	Symbols: | Heat shock protein 70 (Hsp 70) famil	659
Dark-2	159	Background	AT5G02490	Symbols: | Heat shock protein 70 (Hsp 70) famil	659
Dark-2	159	Background	AT5G02490	Symbols: | Heat shock protein 70 (Hsp 70) famil	659
Dark-2	160	Background	AT5G03290	Symbols: IDH-V | isocitrate dehydrogenase V | c	655
Dark-2	160	Background	AT5G03290	Symbols: IDH-V | isocitrate dehydrogenase V | c	655
Dark-2	160	Background	AT5G03290	Symbols: IDH-V | isocitrate dehydrogenase V | c	655
Dark-2	160	Background	AT5G03290	Symbols: IDH-V | isocitrate dehydrogenase V | c	655
Dark-2	160	Background	AT5G03290	Symbols: IDH-V | isocitrate dehydrogenase V | c	655
Dark-2	160	Background	AT5G03290	Symbols: IDH-V | isocitrate dehydrogenase V | c	655
Dark-2	160	Background	AT5G03290	Symbols: IDH-V | isocitrate dehydrogenase V | c	655
Dark-2	160	Background	AT5G03290	Symbols: IDH-V | isocitrate dehydrogenase V | c	655
Dark-2	160	Background	AT5G03290	Symbols: IDH-V | isocitrate dehydrogenase V | c	655
Dark-2	160	Background	AT5G03290	Symbols: IDH-V | isocitrate dehydrogenase V | c	655
Dark-2	160	Background	AT5G03290	Symbols: IDH-V | isocitrate dehydrogenase V | c	655
Dark-2	161	Background	AT3G08580	Symbols: AAC1 | ADP/ATP carrier 1 | chr3:2605	648
Dark-2	161	Background	AT3G08580	Symbols: AAC1 | ADP/ATP carrier 1 | chr3:2605	648
Dark-2	161	Background	AT3G08580	Symbols: AAC1 | ADP/ATP carrier 1 | chr3:2605	648
Dark-2	161	Background	AT3G08580	Symbols: AAC1 | ADP/ATP carrier 1 | chr3:2605	648
Dark-2	161	Background	AT3G08580	Symbols: AAC1 | ADP/ATP carrier 1 | chr3:2605	648
Dark-2	161	Background	AT3G08580	Symbols: AAC1 | ADP/ATP carrier 1 | chr3:2605	648
Dark-2	161	Background	AT3G08580	Symbols: AAC1 | ADP/ATP carrier 1 | chr3:2605	648
Dark-2	161	Background	AT3G08580	Symbols: AAC1 | ADP/ATP carrier 1 | chr3:2605	648
Dark-2	161	Background	AT3G08580	Symbols: AAC1 | ADP/ATP carrier 1 | chr3:2605	648
Dark-2	161	Background	AT3G08580	Symbols: AAC1 | ADP/ATP carrier 1 | chr3:2605	648
Dark-2	162	Background	AT5G13490	Symbols: AAC2 | ADP/ATP carrier 2 | chr5:4336	618
Dark-2	162	Background	AT5G13490	Symbols: AAC2 | ADP/ATP carrier 2 | chr5:4336	618
Dark-2	162	Background	AT5G13490	Symbols: AAC2 | ADP/ATP carrier 2 | chr5:4336	618
Dark-2	162	Background	AT5G13490	Symbols: AAC2 | ADP/ATP carrier 2 | chr5:4336	618
Dark-2	162	Background	AT5G13490	Symbols: AAC2 | ADP/ATP carrier 2 | chr5:4336	618

Dark-2	162	Background	AT5G13490	Symbols: AAC2 | ADP/ATP carrier 2 | chr5:4336(	618
Dark-2	162	Background	AT5G13490	Symbols: AAC2 | ADP/ATP carrier 2 | chr5:4336(	618
Dark-2	162	Background	AT5G13490	Symbols: AAC2 | ADP/ATP carrier 2 | chr5:4336(	618
Dark-2	162	Background	AT5G13490	Symbols: AAC2 | ADP/ATP carrier 2 | chr5:4336(	618
Dark-2	162	Background	AT5G13490	Symbols: AAC2 | ADP/ATP carrier 2 | chr5:4336(	618
Dark-2	162	Background	AT5G13490	Symbols: AAC2 | ADP/ATP carrier 2 | chr5:4336(	618
Dark-2	162	Background	AT5G13490	Symbols: AAC2 | ADP/ATP carrier 2 | chr5:4336(	618
Dark-2	162	Background	AT5G13490	Symbols: AAC2 | ADP/ATP carrier 2 | chr5:4336(	618
Dark-2	163	Background	AT5G09810	Symbols: ACT7 | actin 7 | chr5:3052809-305422	617
Dark-2	163	Background	AT5G09810	Symbols: ACT7 | actin 7 | chr5:3052809-305422	617
Dark-2	163	Background	AT5G09810	Symbols: ACT7 | actin 7 | chr5:3052809-305422	617
Dark-2	163	Background	AT5G09810	Symbols: ACT7 | actin 7 | chr5:3052809-305422	617
Dark-2	163	Background	AT5G09810	Symbols: ACT7 | actin 7 | chr5:3052809-305422	617
Dark-2	163	Background	AT5G09810	Symbols: ACT7 | actin 7 | chr5:3052809-305422	617
Dark-2	163	Background	AT5G09810	Symbols: ACT7 | actin 7 | chr5:3052809-305422	617
Dark-2	163	Background	AT5G09810	Symbols: ACT7 | actin 7 | chr5:3052809-305422	617
Dark-2	163	Background	AT5G09810	Symbols: ACT7 | actin 7 | chr5:3052809-305422	617
Dark-2	163	Background	AT5G09810	Symbols: ACT7 | actin 7 | chr5:3052809-305422	617
Dark-2	163	Background	AT5G09810	Symbols: ACT7 | actin 7 | chr5:3052809-305422	617
Dark-2	163	Background	AT5G09810	Symbols: ACT7 | actin 7 | chr5:3052809-305422	617
Dark-2	163	Background	AT5G09810	Symbols: ACT7 | actin 7 | chr5:3052809-305422	617
Dark-2	164	Background	AT1G69840	Symbols: | SPFH/Band 7/PHB domain-containin	617
Dark-2	164	Background	AT1G69840	Symbols: | SPFH/Band 7/PHB domain-containin	617
Dark-2	164	Background	AT1G69840	Symbols: | SPFH/Band 7/PHB domain-containin	617
Dark-2	164	Background	AT1G69840	Symbols: | SPFH/Band 7/PHB domain-containin	617
Dark-2	164	Background	AT1G69840	Symbols: | SPFH/Band 7/PHB domain-containin	617
Dark-2	164	Background	AT1G69840	Symbols: | SPFH/Band 7/PHB domain-containin	617
Dark-2	164	Background	AT1G69840	Symbols: | SPFH/Band 7/PHB domain-containin	617
Dark-2	164	Background	AT1G69840	Symbols: | SPFH/Band 7/PHB domain-containin	617
Dark-2	164	Background	AT1G69840	Symbols: | SPFH/Band 7/PHB domain-containin	617
Dark-2	164	Background	AT1G69840	Symbols: | SPFH/Band 7/PHB domain-containin	617
Dark-2	165	Background	AT2G44060	Symbols: | Late embryogenesis abundant prote	612
Dark-2	165	Background	AT2G44060	Symbols: | Late embryogenesis abundant prote	612
Dark-2	165	Background	AT2G44060	Symbols: | Late embryogenesis abundant prote	612
Dark-2	165	Background	AT2G44060	Symbols: | Late embryogenesis abundant prote	612
Dark-2	165	Background	AT2G44060	Symbols: | Late embryogenesis abundant prote	612
Dark-2	165	Background	AT2G44060	Symbols: | Late embryogenesis abundant prote	612
Dark-2	165	Background	AT2G44060	Symbols: | Late embryogenesis abundant prote	612
Dark-2	165	Background	AT2G44060	Symbols: | Late embryogenesis abundant prote	612
Dark-2	165	Background	AT2G44060	Symbols: | Late embryogenesis abundant prote	612
Dark-2	165	Background	AT2G44060	Symbols: | Late embryogenesis abundant prote	612
Dark-2	165	Background	AT2G44060	Symbols: | Late embryogenesis abundant prote	612
Dark-2	165	Background	AT2G44060	Symbols: | Late embryogenesis abundant prote	612
Dark-2	165	Background	AT2G44060	Symbols: | Late embryogenesis abundant prote	612
Dark-2	166	Background	AT3G48870	Symbols: ATCLPC, ATHSP93-III, HSP93-III | Clp A	611
Dark-2	166	Background	AT3G48870	Symbols: ATCLPC, ATHSP93-III, HSP93-III | Clp A	611
Dark-2	166	Background	AT3G48870	Symbols: ATCLPC, ATHSP93-III, HSP93-III | Clp A	611
Dark-2	166	Background	AT3G48870	Symbols: ATCLPC, ATHSP93-III, HSP93-III | Clp A	611
Dark-2	166	Background	AT3G48870	Symbols: ATCLPC, ATHSP93-III, HSP93-III | Clp A	611
Dark-2	166	Background	AT3G48870	Symbols: ATCLPC, ATHSP93-III, HSP93-III | Clp A	611
Dark-2	166	Background	AT3G48870	Symbols: ATCLPC, ATHSP93-III, HSP93-III | Clp A	611
Dark-2	166	Background	AT3G48870	Symbols: ATCLPC, ATHSP93-III, HSP93-III | Clp A	611
Dark-2	166	Background	AT3G48870	Symbols: ATCLPC, ATHSP93-III, HSP93-III | Clp A	611
Dark-2	166	Background	AT3G48870	Symbols: ATCLPC, ATHSP93-III, HSP93-III | Clp A	611
Dark-2	166	Background	AT3G48870	Symbols: ATCLPC, ATHSP93-III, HSP93-III | Clp A	611
Dark-2	166	Background	AT3G48870	Symbols: ATCLPC, ATHSP93-III, HSP93-III | Clp A	611
Dark-2	166	Background	AT3G48870	Symbols: ATCLPC, ATHSP93-III, HSP93-III | Clp A	611
Dark-2	166	Background	AT3G48870	Symbols: ATCLPC, ATHSP93-III, HSP93-III | Clp A	611

Dark-2	166	Background	AT3G48870	Symbols: ATCLPC, ATHSP93-III, HSP93-III | Clp A	611
Dark-2	166	Background	AT3G48870	Symbols: ATCLPC, ATHSP93-III, HSP93-III | Clp A	611
Dark-2	166	Background	AT3G48870	Symbols: ATCLPC, ATHSP93-III, HSP93-III | Clp A	611
Dark-2	166	Background	AT3G48870	Symbols: ATCLPC, ATHSP93-III, HSP93-III | Clp A	611
Dark-2	167	Background	AT1G09100	Symbols: RPT5B | 26S proteasome AAA-ATPase	597
Dark-2	167	Background	AT1G09100	Symbols: RPT5B | 26S proteasome AAA-ATPase	597
Dark-2	167	Background	AT1G09100	Symbols: RPT5B | 26S proteasome AAA-ATPase	597
Dark-2	167	Background	AT1G09100	Symbols: RPT5B | 26S proteasome AAA-ATPase	597
Dark-2	167	Background	AT1G09100	Symbols: RPT5B | 26S proteasome AAA-ATPase	597
Dark-2	167	Background	AT1G09100	Symbols: RPT5B | 26S proteasome AAA-ATPase	597
Dark-2	167	Background	AT1G09100	Symbols: RPT5B | 26S proteasome AAA-ATPase	597
Dark-2	167	Background	AT1G09100	Symbols: RPT5B | 26S proteasome AAA-ATPase	597
Dark-2	167	Background	AT1G09100	Symbols: RPT5B | 26S proteasome AAA-ATPase	597
Dark-2	167	Background	AT1G09100	Symbols: RPT5B | 26S proteasome AAA-ATPase	597
Dark-2	167	Background	AT1G09100	Symbols: RPT5B | 26S proteasome AAA-ATPase	597
Dark-2	167	Background	AT1G09100	Symbols: RPT5B | 26S proteasome AAA-ATPase	597
Dark-2	167	Background	AT1G09100	Symbols: RPT5B | 26S proteasome AAA-ATPase	597
Dark-2	168	Background	AT3G22330	Symbols: PMH2, ATRH53 | putative mitochondri	562
Dark-2	168	Background	AT3G22330	Symbols: PMH2, ATRH53 | putative mitochondri	562
Dark-2	168	Background	AT3G22330	Symbols: PMH2, ATRH53 | putative mitochondri	562
Dark-2	168	Background	AT3G22330	Symbols: PMH2, ATRH53 | putative mitochondri	562
Dark-2	168	Background	AT3G22330	Symbols: PMH2, ATRH53 | putative mitochondri	562
Dark-2	168	Background	AT3G22330	Symbols: PMH2, ATRH53 | putative mitochondri	562
Dark-2	168	Background	AT3G22330	Symbols: PMH2, ATRH53 | putative mitochondri	562
Dark-2	168	Background	AT3G22330	Symbols: PMH2, ATRH53 | putative mitochondri	562
Dark-2	168	Background	AT3G22330	Symbols: PMH2, ATRH53 | putative mitochondri	562
Dark-2	168	Background	AT3G22330	Symbols: PMH2, ATRH53 | putative mitochondri	562
Dark-2	168	Background	AT3G22330	Symbols: PMH2, ATRH53 | putative mitochondri	562
Dark-2	168	Background	AT3G22330	Symbols: PMH2, ATRH53 | putative mitochondri	562
Dark-2	168	Background	AT3G22330	Symbols: PMH2, ATRH53 | putative mitochondri	562
Dark-2	168	Background	AT3G22330	Symbols: PMH2, ATRH53 | putative mitochondri	562
Dark-2	169	Background	AT3G43300	Symbols: ATMIN7, BEN1 | HOPM interactor 7 |	555
Dark-2	169	Background	AT3G43300	Symbols: ATMIN7, BEN1 | HOPM interactor 7 |	555
Dark-2	169	Background	AT3G43300	Symbols: ATMIN7, BEN1 | HOPM interactor 7 |	555
Dark-2	169	Background	AT3G43300	Symbols: ATMIN7, BEN1 | HOPM interactor 7 |	555
Dark-2	169	Background	AT3G43300	Symbols: ATMIN7, BEN1 | HOPM interactor 7 |	555
Dark-2	169	Background	AT3G43300	Symbols: ATMIN7, BEN1 | HOPM interactor 7 |	555
Dark-2	169	Background	AT3G43300	Symbols: ATMIN7, BEN1 | HOPM interactor 7 |	555
Dark-2	169	Background	AT3G43300	Symbols: ATMIN7, BEN1 | HOPM interactor 7 |	555
Dark-2	169	Background	AT3G43300	Symbols: ATMIN7, BEN1 | HOPM interactor 7 |	555
Dark-2	169	Background	AT3G43300	Symbols: ATMIN7, BEN1 | HOPM interactor 7 |	555
Dark-2	169	Background	AT3G43300	Symbols: ATMIN7, BEN1 | HOPM interactor 7 |	555
Dark-2	170	Background	AT2G39730	Symbols: RCA | rubisco activase | chr2:1657095	549
Dark-2	170	Background	AT2G39730	Symbols: RCA | rubisco activase | chr2:1657095	549
Dark-2	170	Background	AT2G39730	Symbols: RCA | rubisco activase | chr2:1657095	549
Dark-2	170	Background	AT2G39730	Symbols: RCA | rubisco activase | chr2:1657095	549
Dark-2	170	Background	AT2G39730	Symbols: RCA | rubisco activase | chr2:1657095	549
Dark-2	170	Background	AT2G39730	Symbols: RCA | rubisco activase | chr2:1657095	549
Dark-2	171	Background	AT3G56150	Symbols: EIF3C, ATEIF3C-1, EIF3C-1, ATTIF3C1, T	541
Dark-2	171	Background	AT3G56150	Symbols: EIF3C, ATEIF3C-1, EIF3C-1, ATTIF3C1, T	541
Dark-2	171	Background	AT3G56150	Symbols: EIF3C, ATEIF3C-1, EIF3C-1, ATTIF3C1, T	541
Dark-2	171	Background	AT3G56150	Symbols: EIF3C, ATEIF3C-1, EIF3C-1, ATTIF3C1, T	541
Dark-2	171	Background	AT3G56150	Symbols: EIF3C, ATEIF3C-1, EIF3C-1, ATTIF3C1, T	541
Dark-2	171	Background	AT3G56150	Symbols: EIF3C, ATEIF3C-1, EIF3C-1, ATTIF3C1, T	541
Dark-2	171	Background	AT3G56150	Symbols: EIF3C, ATEIF3C-1, EIF3C-1, ATTIF3C1, T	541
Dark-2	171	Background	AT3G56150	Symbols: EIF3C, ATEIF3C-1, EIF3C-1, ATTIF3C1, T	541
Dark-2	171	Background	AT3G56150	Symbols: EIF3C, ATEIF3C-1, EIF3C-1, ATTIF3C1, T	541
Dark-2	171	Background	AT3G56150	Symbols: EIF3C, ATEIF3C-1, EIF3C-1, ATTIF3C1, T	541

Dark-2	171	Background	AT3G56150	Symbols: EIF3C, ATEIF3C-1, EIF3C-1, ATTIF3C1, T	541
Dark-2	171	Background	AT3G56150	Symbols: EIF3C, ATEIF3C-1, EIF3C-1, ATTIF3C1, T	541
Dark-2	172	Background	AT2G20140	Symbols: | AAA-type ATPase family protein | ch	528
Dark-2	172	Background	AT2G20140	Symbols: | AAA-type ATPase family protein | ch	528
Dark-2	172	Background	AT2G20140	Symbols: | AAA-type ATPase family protein | ch	528
Dark-2	172	Background	AT2G20140	Symbols: | AAA-type ATPase family protein | ch	528
Dark-2	172	Background	AT2G20140	Symbols: | AAA-type ATPase family protein | ch	528
Dark-2	172	Background	AT2G20140	Symbols: | AAA-type ATPase family protein | ch	528
Dark-2	172	Background	AT2G20140	Symbols: | AAA-type ATPase family protein | ch	528
Dark-2	172	Background	AT2G20140	Symbols: | AAA-type ATPase family protein | ch	528
Dark-2	172	Background	AT2G20140	Symbols: | AAA-type ATPase family protein | ch	528
Dark-2	173	Background	AT5G50920	Symbols: CLPC, ATHSP93-V, HSP93-V, DCA1, CLP	493
Dark-2	173	Background	AT5G50920	Symbols: CLPC, ATHSP93-V, HSP93-V, DCA1, CLP	493
Dark-2	173	Background	AT5G50920	Symbols: CLPC, ATHSP93-V, HSP93-V, DCA1, CLP	493
Dark-2	173	Background	AT5G50920	Symbols: CLPC, ATHSP93-V, HSP93-V, DCA1, CLP	493
Dark-2	173	Background	AT5G50920	Symbols: CLPC, ATHSP93-V, HSP93-V, DCA1, CLP	493
Dark-2	173	Background	AT5G50920	Symbols: CLPC, ATHSP93-V, HSP93-V, DCA1, CLP	493
Dark-2	173	Background	AT5G50920	Symbols: CLPC, ATHSP93-V, HSP93-V, DCA1, CLP	493
Dark-2	173	Background	AT5G50920	Symbols: CLPC, ATHSP93-V, HSP93-V, DCA1, CLP	493
Dark-2	173	Background	AT5G50920	Symbols: CLPC, ATHSP93-V, HSP93-V, DCA1, CLP	493
Dark-2	173	Background	AT5G50920	Symbols: CLPC, ATHSP93-V, HSP93-V, DCA1, CLP	493
Dark-2	173	Background	AT5G50920	Symbols: CLPC, ATHSP93-V, HSP93-V, DCA1, CLP	493
Dark-2	173	Background	AT5G50920	Symbols: CLPC, ATHSP93-V, HSP93-V, DCA1, CLP	493
Dark-2	173	Background	AT5G50920	Symbols: CLPC, ATHSP93-V, HSP93-V, DCA1, CLP	493
Dark-2	173	Background	AT5G50920	Symbols: CLPC, ATHSP93-V, HSP93-V, DCA1, CLP	493
Dark-2	173	Background	AT5G50920	Symbols: CLPC, ATHSP93-V, HSP93-V, DCA1, CLP	493
Dark-2	174	Background	AT5G35360	Symbols: CAC2 | acetyl Co-enzyme a carboxylase	492
Dark-2	174	Background	AT5G35360	Symbols: CAC2 | acetyl Co-enzyme a carboxylase	492
Dark-2	174	Background	AT5G35360	Symbols: CAC2 | acetyl Co-enzyme a carboxylase	492
Dark-2	174	Background	AT5G35360	Symbols: CAC2 | acetyl Co-enzyme a carboxylase	492
Dark-2	174	Background	AT5G35360	Symbols: CAC2 | acetyl Co-enzyme a carboxylase	492
Dark-2	174	Background	AT5G35360	Symbols: CAC2 | acetyl Co-enzyme a carboxylase	492
Dark-2	174	Background	AT5G35360	Symbols: CAC2 | acetyl Co-enzyme a carboxylase	492
Dark-2	174	Background	AT5G35360	Symbols: CAC2 | acetyl Co-enzyme a carboxylase	492
Dark-2	174	Background	AT5G35360	Symbols: CAC2 | acetyl Co-enzyme a carboxylase	492
Dark-2	174	Background	AT5G35360	Symbols: CAC2 | acetyl Co-enzyme a carboxylase	492
Dark-2	174	Background	AT5G35360	Symbols: CAC2 | acetyl Co-enzyme a carboxylase	492
Dark-2	174	Background	AT5G35360	Symbols: CAC2 | acetyl Co-enzyme a carboxylase	492
Dark-2	174	Background	AT5G35360	Symbols: CAC2 | acetyl Co-enzyme a carboxylase	492
Dark-2	174	Background	AT5G35360	Symbols: CAC2 | acetyl Co-enzyme a carboxylase	492
Dark-2	175	Background	AT4G37910	Symbols: mtHsc70-1 | mitochondrial heat shock	475
Dark-2	175	Background	AT4G37910	Symbols: mtHsc70-1 | mitochondrial heat shock	475
Dark-2	175	Background	AT4G37910	Symbols: mtHsc70-1 | mitochondrial heat shock	475
Dark-2	175	Background	AT4G37910	Symbols: mtHsc70-1 | mitochondrial heat shock	475
Dark-2	175	Background	AT4G37910	Symbols: mtHsc70-1 | mitochondrial heat shock	475
Dark-2	175	Background	AT4G37910	Symbols: mtHsc70-1 | mitochondrial heat shock	475
Dark-2	175	Background	AT4G37910	Symbols: mtHsc70-1 | mitochondrial heat shock	475
Dark-2	175	Background	AT4G37910	Symbols: mtHsc70-1 | mitochondrial heat shock	475
Dark-2	175	Background	AT4G37910	Symbols: mtHsc70-1 | mitochondrial heat shock	475
Dark-2	175	Background	AT4G37910	Symbols: mtHsc70-1 | mitochondrial heat shock	475
Dark-2	175	Background	AT4G37910	Symbols: mtHsc70-1 | mitochondrial heat shock	475
Dark-2	176	Background	AT1G56070	Symbols: LOS1 | Ribosomal protein S5/Elongatic	463
Dark-2	176	Background	AT1G56070	Symbols: LOS1 | Ribosomal protein S5/Elongatic	463
Dark-2	176	Background	AT1G56070	Symbols: LOS1 | Ribosomal protein S5/Elongatic	463
Dark-2	176	Background	AT1G56070	Symbols: LOS1 | Ribosomal protein S5/Elongatic	463
Dark-2	176	Background	AT1G56070	Symbols: LOS1 | Ribosomal protein S5/Elongatic	463
Dark-2	176	Background	AT1G56070	Symbols: LOS1 | Ribosomal protein S5/Elongatic	463
Dark-2	176	Background	AT1G56070	Symbols: LOS1 | Ribosomal protein S5/Elongatic	463

Dark-2	176	Background	AT1G56070	Symbols: LOS1 | Ribosomal protein S5/Elongatic	463
Dark-2	176	Background	AT1G56070	Symbols: LOS1 | Ribosomal protein S5/Elongatic	463
Dark-2	176	Background	AT1G56070	Symbols: LOS1 | Ribosomal protein S5/Elongatic	463
Dark-2	176	Background	AT1G56070	Symbols: LOS1 | Ribosomal protein S5/Elongatic	463
Dark-2	176	Background	AT1G56070	Symbols: LOS1 | Ribosomal protein S5/Elongatic	463
Dark-2	177	Background	AT3G06720	Symbols: AT-IMP, ATKAP ALPHA, AIMP ALPHA, II	453
Dark-2	177	Background	AT3G06720	Symbols: AT-IMP, ATKAP ALPHA, AIMP ALPHA, II	453
Dark-2	177	Background	AT3G06720	Symbols: AT-IMP, ATKAP ALPHA, AIMP ALPHA, II	453
Dark-2	177	Background	AT3G06720	Symbols: AT-IMP, ATKAP ALPHA, AIMP ALPHA, II	453
Dark-2	177	Background	AT3G06720	Symbols: AT-IMP, ATKAP ALPHA, AIMP ALPHA, II	453
Dark-2	177	Background	AT3G06720	Symbols: AT-IMP, ATKAP ALPHA, AIMP ALPHA, II	453
Dark-2	177	Background	AT3G06720	Symbols: AT-IMP, ATKAP ALPHA, AIMP ALPHA, II	453
Dark-2	178	Background	AT4G35260	Symbols: IDH1, IDH-I | isocitrate dehydrogenase	451
Dark-2	178	Background	AT4G35260	Symbols: IDH1, IDH-I | isocitrate dehydrogenase	451
Dark-2	178	Background	AT4G35260	Symbols: IDH1, IDH-I | isocitrate dehydrogenase	451
Dark-2	178	Background	AT4G35260	Symbols: IDH1, IDH-I | isocitrate dehydrogenase	451
Dark-2	178	Background	AT4G35260	Symbols: IDH1, IDH-I | isocitrate dehydrogenase	451
Dark-2	178	Background	AT4G35260	Symbols: IDH1, IDH-I | isocitrate dehydrogenase	451
Dark-2	178	Background	AT4G35260	Symbols: IDH1, IDH-I | isocitrate dehydrogenase	451
Dark-2	178	Background	AT4G35260	Symbols: IDH1, IDH-I | isocitrate dehydrogenase	451
Dark-2	178	Background	AT4G35260	Symbols: IDH1, IDH-I | isocitrate dehydrogenase	451
Dark-2	178	Background	AT4G35260	Symbols: IDH1, IDH-I | isocitrate dehydrogenase	451
Dark-2	179	Background	AT1G52670	Symbols: | Single hybrid motif superfamily prot	436
Dark-2	179	Background	AT1G52670	Symbols: | Single hybrid motif superfamily prot	436
Dark-2	179	Background	AT1G52670	Symbols: | Single hybrid motif superfamily prot	436
Dark-2	179	Background	AT1G52670	Symbols: | Single hybrid motif superfamily prot	436
Dark-2	179	Background	AT1G52670	Symbols: | Single hybrid motif superfamily prot	436
Dark-2	179	Background	AT1G52670	Symbols: | Single hybrid motif superfamily prot	436
Dark-2	179	Background	AT1G52670	Symbols: | Single hybrid motif superfamily prot	436
Dark-2	180	Table 1 Filtere	AT4G02380	Symbols: SAG21, AtLEA5 | senescence-associate	435
Dark-2	180	Table 1 Filtere	AT4G02380	Symbols: SAG21, AtLEA5 | senescence-associate	435
Dark-2	180	Table 1 Filtere	AT4G02380	Symbols: SAG21, AtLEA5 | senescence-associate	435
Dark-2	180	Table 1 Filtere	AT4G02380	Symbols: SAG21, AtLEA5 | senescence-associate	435
Dark-2	180	Table 1 Filtere	AT4G02380	Symbols: SAG21, AtLEA5 | senescence-associate	435
Dark-2	181	Background	AT3G63460	Symbols: | transducin family protein / WD-40 re	423
Dark-2	181	Background	AT3G63460	Symbols: | transducin family protein / WD-40 re	423
Dark-2	181	Background	AT3G63460	Symbols: | transducin family protein / WD-40 re	423
Dark-2	181	Background	AT3G63460	Symbols: | transducin family protein / WD-40 re	423
Dark-2	181	Background	AT3G63460	Symbols: | transducin family protein / WD-40 re	423
Dark-2	181	Background	AT3G63460	Symbols: | transducin family protein / WD-40 re	423
Dark-2	181	Background	AT3G63460	Symbols: | transducin family protein / WD-40 re	423
Dark-2	181	Background	AT3G63460	Symbols: | transducin family protein / WD-40 re	423
Dark-2	181	Background	AT3G63460	Symbols: | transducin family protein / WD-40 re	423
Dark-2	181	Background	AT3G63460	Symbols: | transducin family protein / WD-40 re	423
Dark-2	181	Background	AT3G63460	Symbols: | transducin family protein / WD-40 re	423
Dark-2	182	Background	AT5G08670	Symbols: | ATP synthase alpha/beta family prot	418
Dark-2	182	Background	AT5G08670	Symbols: | ATP synthase alpha/beta family prot	418
Dark-2	182	Background	AT5G08670	Symbols: | ATP synthase alpha/beta family prot	418
Dark-2	182	Background	AT5G08670	Symbols: | ATP synthase alpha/beta family prot	418
Dark-2	182	Background	AT5G08670	Symbols: | ATP synthase alpha/beta family prot	418
Dark-2	182	Background	AT5G08670	Symbols: | ATP synthase alpha/beta family prot	418
Dark-2	182	Background	AT5G08670	Symbols: | ATP synthase alpha/beta family prot	418
Dark-2	182	Background	AT5G08670	Symbols: | ATP synthase alpha/beta family prot	418
Dark-2	182	Background	AT5G08670	Symbols: | ATP synthase alpha/beta family prot	418
Dark-2	183	Background	AT5G62740	Symbols: HIR1, ATHIR1 | SPFH/Band 7/PHB dom	416
Dark-2	183	Background	AT5G62740	Symbols: HIR1, ATHIR1 | SPFH/Band 7/PHB dom	416
Dark-2	183	Background	AT5G62740	Symbols: HIR1, ATHIR1 | SPFH/Band 7/PHB dom	416
Dark-2	183	Background	AT5G62740	Symbols: HIR1, ATHIR1 | SPFH/Band 7/PHB dom	416

Dark-2	183	Background	AT5G62740	Symbols: HIR1, ATHIR1 | SPFH/Band 7/PHB dom	416
Dark-2	183	Background	AT5G62740	Symbols: HIR1, ATHIR1 | SPFH/Band 7/PHB dom	416
Dark-2	183	Background	AT5G62740	Symbols: HIR1, ATHIR1 | SPFH/Band 7/PHB dom	416
Dark-2	183	Background	AT5G62740	Symbols: HIR1, ATHIR1 | SPFH/Band 7/PHB dom	416
Dark-2	183	Background	AT5G62740	Symbols: HIR1, ATHIR1 | SPFH/Band 7/PHB dom	416
Dark-2	183	Background	AT5G62740	Symbols: HIR1, ATHIR1 | SPFH/Band 7/PHB dom	416
Dark-2	184	Background	AT3G22310	Symbols: PMH1, ATRH9 | putative mitochondria	407
Dark-2	184	Background	AT3G22310	Symbols: PMH1, ATRH9 | putative mitochondria	407
Dark-2	184	Background	AT3G22310	Symbols: PMH1, ATRH9 | putative mitochondria	407
Dark-2	184	Background	AT3G22310	Symbols: PMH1, ATRH9 | putative mitochondria	407
Dark-2	184	Background	AT3G22310	Symbols: PMH1, ATRH9 | putative mitochondria	407
Dark-2	184	Background	AT3G22310	Symbols: PMH1, ATRH9 | putative mitochondria	407
Dark-2	184	Background	AT3G22310	Symbols: PMH1, ATRH9 | putative mitochondria	407
Dark-2	184	Background	AT3G22310	Symbols: PMH1, ATRH9 | putative mitochondria	407
Dark-2	184	Background	AT3G22310	Symbols: PMH1, ATRH9 | putative mitochondria	407
Dark-2	184	Background	AT3G22310	Symbols: PMH1, ATRH9 | putative mitochondria	407
Dark-2	184	Background	AT3G22310	Symbols: PMH1, ATRH9 | putative mitochondria	407
Dark-2	184	Background	AT3G22310	Symbols: PMH1, ATRH9 | putative mitochondria	407
Dark-2	185	Table 1 Filter	AT3G16810	Symbols: APUM24, PUM24 | pumilio 24 | chr3:5	405
Dark-2	185	Table 1 Filter	AT3G16810	Symbols: APUM24, PUM24 | pumilio 24 | chr3:5	405
Dark-2	185	Table 1 Filter	AT3G16810	Symbols: APUM24, PUM24 | pumilio 24 | chr3:5	405
Dark-2	185	Table 1 Filter	AT3G16810	Symbols: APUM24, PUM24 | pumilio 24 | chr3:5	405
Dark-2	185	Table 1 Filter	AT3G16810	Symbols: APUM24, PUM24 | pumilio 24 | chr3:5	405
Dark-2	185	Table 1 Filter	AT3G16810	Symbols: APUM24, PUM24 | pumilio 24 | chr3:5	405
Dark-2	186	Background	AT2G46520	Symbols: | cellular apoptosis susceptibility prot	400
Dark-2	186	Background	AT2G46520	Symbols: | cellular apoptosis susceptibility prot	400
Dark-2	186	Background	AT2G46520	Symbols: | cellular apoptosis susceptibility prot	400
Dark-2	186	Background	AT2G46520	Symbols: | cellular apoptosis susceptibility prot	400
Dark-2	186	Background	AT2G46520	Symbols: | cellular apoptosis susceptibility prot	400
Dark-2	186	Background	AT2G46520	Symbols: | cellular apoptosis susceptibility prot	400
Dark-2	186	Background	AT2G46520	Symbols: | cellular apoptosis susceptibility prot	400
Dark-2	186	Background	AT2G46520	Symbols: | cellular apoptosis susceptibility prot	400
Dark-2	187	Background	AT1G43800	Symbols: | Plant stearoyl-acyl-carrier-protein de	383
Dark-2	187	Background	AT1G43800	Symbols: | Plant stearoyl-acyl-carrier-protein de	383
Dark-2	187	Background	AT1G43800	Symbols: | Plant stearoyl-acyl-carrier-protein de	383
Dark-2	187	Background	AT1G43800	Symbols: | Plant stearoyl-acyl-carrier-protein de	383
Dark-2	187	Background	AT1G43800	Symbols: | Plant stearoyl-acyl-carrier-protein de	383
Dark-2	187	Background	AT1G43800	Symbols: | Plant stearoyl-acyl-carrier-protein de	383
Dark-2	188	Background	AT2G24050	Symbols: eIFiso4G2 | MIF4G domain-containing	383
Dark-2	188	Background	AT2G24050	Symbols: eIFiso4G2 | MIF4G domain-containing	383
Dark-2	188	Background	AT2G24050	Symbols: eIFiso4G2 | MIF4G domain-containing	383
Dark-2	188	Background	AT2G24050	Symbols: eIFiso4G2 | MIF4G domain-containing	383
Dark-2	188	Background	AT2G24050	Symbols: eIFiso4G2 | MIF4G domain-containing	383
Dark-2	188	Background	AT2G24050	Symbols: eIFiso4G2 | MIF4G domain-containing	383
Dark-2	188	Background	AT2G24050	Symbols: eIFiso4G2 | MIF4G domain-containing	383
Dark-2	188	Background	AT2G24050	Symbols: eIFiso4G2 | MIF4G domain-containing	383
Dark-2	188	Background	AT2G24050	Symbols: eIFiso4G2 | MIF4G domain-containing	383
Dark-2	188	Background	AT2G24050	Symbols: eIFiso4G2 | MIF4G domain-containing	383
Dark-2	189	Table 1 Filter	AT1G59990	Symbols: | DEA(D/H)-box RNA helicase family p	377
Dark-2	189	Table 1 Filter	AT1G59990	Symbols: | DEA(D/H)-box RNA helicase family p	377
Dark-2	189	Table 1 Filter	AT1G59990	Symbols: | DEA(D/H)-box RNA helicase family p	377
Dark-2	189	Table 1 Filter	AT1G59990	Symbols: | DEA(D/H)-box RNA helicase family p	377
Dark-2	189	Table 1 Filter	AT1G59990	Symbols: | DEA(D/H)-box RNA helicase family p	377
Dark-2	189	Table 1 Filter	AT1G59990	Symbols: | DEA(D/H)-box RNA helicase family p	377
Dark-2	190	Background	AT1G79530	Symbols: GAPCP-1 | glyceraldehyde-3-phosphat	375
Dark-2	190	Background	AT1G79530	Symbols: GAPCP-1 | glyceraldehyde-3-phosphat	375
Dark-2	190	Background	AT1G79530	Symbols: GAPCP-1 | glyceraldehyde-3-phosphat	375
Dark-2	190	Background	AT1G79530	Symbols: GAPCP-1 | glyceraldehyde-3-phosphat	375

Dark-2	190	Background	AT1G79530	Symbols: GAPCP-1 | glyceraldehyde-3-phosphat	375
Dark-2	190	Background	AT1G79530	Symbols: GAPCP-1 | glyceraldehyde-3-phosphat	375
Dark-2	191	Background	AT3G09810	Symbols: IDH-VI | isocitrate dehydrogenase VI |	372
Dark-2	191	Background	AT3G09810	Symbols: IDH-VI | isocitrate dehydrogenase VI |	372
Dark-2	191	Background	AT3G09810	Symbols: IDH-VI | isocitrate dehydrogenase VI |	372
Dark-2	191	Background	AT3G09810	Symbols: IDH-VI | isocitrate dehydrogenase VI |	372
Dark-2	191	Background	AT3G09810	Symbols: IDH-VI | isocitrate dehydrogenase VI |	372
Dark-2	191	Background	AT3G09810	Symbols: IDH-VI | isocitrate dehydrogenase VI |	372
Dark-2	191	Background	AT3G09810	Symbols: IDH-VI | isocitrate dehydrogenase VI |	372
Dark-2	191	Background	AT3G09810	Symbols: IDH-VI | isocitrate dehydrogenase VI |	372
Dark-2	192	Background	AT1G10630	Symbols: ATARFA1F, ARFA1F | ADP-ribosylation	361
Dark-2	192	Background	AT1G10630	Symbols: ATARFA1F, ARFA1F | ADP-ribosylation	361
Dark-2	192	Background	AT1G10630	Symbols: ATARFA1F, ARFA1F | ADP-ribosylation	361
Dark-2	192	Background	AT1G10630	Symbols: ATARFA1F, ARFA1F | ADP-ribosylation	361
Dark-2	192	Background	AT1G10630	Symbols: ATARFA1F, ARFA1F | ADP-ribosylation	361
Dark-2	192	Background	AT1G10630	Symbols: ATARFA1F, ARFA1F | ADP-ribosylation	361
Dark-2	192	Background	AT1G10630	Symbols: ATARFA1F, ARFA1F | ADP-ribosylation	361
Dark-2	193	Background	AT5G43010	Symbols: RPT4A | regulatory particle triple-A AT	356
Dark-2	193	Background	AT5G43010	Symbols: RPT4A | regulatory particle triple-A AT	356
Dark-2	193	Background	AT5G43010	Symbols: RPT4A | regulatory particle triple-A AT	356
Dark-2	193	Background	AT5G43010	Symbols: RPT4A | regulatory particle triple-A AT	356
Dark-2	193	Background	AT5G43010	Symbols: RPT4A | regulatory particle triple-A AT	356
Dark-2	193	Background	AT5G43010	Symbols: RPT4A | regulatory particle triple-A AT	356
Dark-2	193	Background	AT5G43010	Symbols: RPT4A | regulatory particle triple-A AT	356
Dark-2	193	Background	AT5G43010	Symbols: RPT4A | regulatory particle triple-A AT	356
Dark-2	193	Background	AT5G43010	Symbols: RPT4A | regulatory particle triple-A AT	356
Dark-2	193	Background	AT5G43010	Symbols: RPT4A | regulatory particle triple-A AT	356
Dark-2	194	Background	AT1G56110	Symbols: NOP56 | homolog of nucleolar protein	355
Dark-2	194	Background	AT1G56110	Symbols: NOP56 | homolog of nucleolar protein	355
Dark-2	194	Background	AT1G56110	Symbols: NOP56 | homolog of nucleolar protein	355
Dark-2	194	Background	AT1G56110	Symbols: NOP56 | homolog of nucleolar protein	355
Dark-2	194	Background	AT1G56110	Symbols: NOP56 | homolog of nucleolar protein	355
Dark-2	194	Background	AT1G56110	Symbols: NOP56 | homolog of nucleolar protein	355
Dark-2	194	Background	AT1G56110	Symbols: NOP56 | homolog of nucleolar protein	355
Dark-2	194	Background	AT1G56110	Symbols: NOP56 | homolog of nucleolar protein	355
Dark-2	195	Background	AT3G12110	Symbols: ACT11 | actin-11 | chr3:3858116-3859	354
Dark-2	195	Background	AT3G12110	Symbols: ACT11 | actin-11 | chr3:3858116-3859	354
Dark-2	195	Background	AT3G12110	Symbols: ACT11 | actin-11 | chr3:3858116-3859	354
Dark-2	195	Background	AT3G12110	Symbols: ACT11 | actin-11 | chr3:3858116-3859	354
Dark-2	195	Background	AT3G12110	Symbols: ACT11 | actin-11 | chr3:3858116-3859	354
Dark-2	195	Background	AT3G12110	Symbols: ACT11 | actin-11 | chr3:3858116-3859	354
Dark-2	195	Background	AT3G12110	Symbols: ACT11 | actin-11 | chr3:3858116-3859	354
Dark-2	195	Background	AT3G12110	Symbols: ACT11 | actin-11 | chr3:3858116-3859	354
Dark-2	195	Background	AT3G12110	Symbols: ACT11 | actin-11 | chr3:3858116-3859	354
Dark-2	196	Background	AT5G19820	Symbols: emb2734 | ARM repeat superfamily pr	354
Dark-2	196	Background	AT5G19820	Symbols: emb2734 | ARM repeat superfamily pr	354
Dark-2	196	Background	AT5G19820	Symbols: emb2734 | ARM repeat superfamily pr	354
Dark-2	196	Background	AT5G19820	Symbols: emb2734 | ARM repeat superfamily pr	354
Dark-2	196	Background	AT5G19820	Symbols: emb2734 | ARM repeat superfamily pr	354
Dark-2	196	Background	AT5G19820	Symbols: emb2734 | ARM repeat superfamily pr	354
Dark-2	196	Background	AT5G19820	Symbols: emb2734 | ARM repeat superfamily pr	354
Dark-2	196	Background	AT5G19820	Symbols: emb2734 | ARM repeat superfamily pr	354
Dark-2	196	Background	AT5G19820	Symbols: emb2734 | ARM repeat superfamily pr	354
Dark-2	196	Background	AT5G19820	Symbols: emb2734 | ARM repeat superfamily pr	354
Dark-2	196	Background	AT5G19820	Symbols: emb2734 | ARM repeat superfamily pr	354
Dark-2	197	Background	AT1G33120	Symbols: | Ribosomal protein L6 family | chr1:1	351
Dark-2	197	Background	AT1G33120	Symbols: | Ribosomal protein L6 family | chr1:1	351
Dark-2	197	Background	AT1G33120	Symbols: | Ribosomal protein L6 family | chr1:1	351

Dark-2	197	Background	AT1G33120	Symbols: | Ribosomal protein L6 family | chr1:1	351
Dark-2	197	Background	AT1G33120	Symbols: | Ribosomal protein L6 family | chr1:1	351
Dark-2	197	Background	AT1G33120	Symbols: | Ribosomal protein L6 family | chr1:1	351
Dark-2	197	Background	AT1G33120	Symbols: | Ribosomal protein L6 family | chr1:1	351
Dark-2	197	Background	AT1G33120	Symbols: | Ribosomal protein L6 family | chr1:1	351
Dark-2	197	Background	AT1G33120	Symbols: | Ribosomal protein L6 family | chr1:1	351
Dark-2	198	Background	AT1G80270	Symbols: PPR596 | PENTATRICOPEPTIDE REPEA	343
Dark-2	198	Background	AT1G80270	Symbols: PPR596 | PENTATRICOPEPTIDE REPEA	343
Dark-2	198	Background	AT1G80270	Symbols: PPR596 | PENTATRICOPEPTIDE REPEA	343
Dark-2	198	Background	AT1G80270	Symbols: PPR596 | PENTATRICOPEPTIDE REPEA	343
Dark-2	198	Background	AT1G80270	Symbols: PPR596 | PENTATRICOPEPTIDE REPEA	343
Dark-2	198	Background	AT1G80270	Symbols: PPR596 | PENTATRICOPEPTIDE REPEA	343
Dark-2	198	Background	AT1G80270	Symbols: PPR596 | PENTATRICOPEPTIDE REPEA	343
Dark-2	199	Background	AT5G09590	Symbols: MTHSC70-2, HSC70-5 | mitochondrial	339
Dark-2	199	Background	AT5G09590	Symbols: MTHSC70-2, HSC70-5 | mitochondrial	339
Dark-2	199	Background	AT5G09590	Symbols: MTHSC70-2, HSC70-5 | mitochondrial	339
Dark-2	199	Background	AT5G09590	Symbols: MTHSC70-2, HSC70-5 | mitochondrial	339
Dark-2	199	Background	AT5G09590	Symbols: MTHSC70-2, HSC70-5 | mitochondrial	339
Dark-2	199	Background	AT5G09590	Symbols: MTHSC70-2, HSC70-5 | mitochondrial	339
Dark-2	199	Background	AT5G09590	Symbols: MTHSC70-2, HSC70-5 | mitochondrial	339
Dark-2	199	Background	AT5G09590	Symbols: MTHSC70-2, HSC70-5 | mitochondrial	339
Dark-2	199	Background	AT5G09590	Symbols: MTHSC70-2, HSC70-5 | mitochondrial	339
Dark-2	199	Background	AT5G09590	Symbols: MTHSC70-2, HSC70-5 | mitochondrial	339
Dark-2	200	Background	AT2G15860	Symbols: | unknown protein; FUNCTIONS IN: m	339
Dark-2	200	Background	AT2G15860	Symbols: | unknown protein; FUNCTIONS IN: m	339
Dark-2	200	Background	AT2G15860	Symbols: | unknown protein; FUNCTIONS IN: m	339
Dark-2	200	Background	AT2G15860	Symbols: | unknown protein; FUNCTIONS IN: m	339
Dark-2	200	Background	AT2G15860	Symbols: | unknown protein; FUNCTIONS IN: m	339
Dark-2	200	Background	AT2G15860	Symbols: | unknown protein; FUNCTIONS IN: m	339
Dark-2	200	Background	AT2G15860	Symbols: | unknown protein; FUNCTIONS IN: m	339
Dark-2	201	Background	AT2G37620	Symbols: ACT1, AAc1 | actin 1 | chr2:15779761-	335
Dark-2	201	Background	AT2G37620	Symbols: ACT1, AAc1 | actin 1 | chr2:15779761-	335
Dark-2	201	Background	AT2G37620	Symbols: ACT1, AAc1 | actin 1 | chr2:15779761-	335
Dark-2	201	Background	AT2G37620	Symbols: ACT1, AAc1 | actin 1 | chr2:15779761-	335
Dark-2	201	Background	AT2G37620	Symbols: ACT1, AAc1 | actin 1 | chr2:15779761-	335
Dark-2	201	Background	AT2G37620	Symbols: ACT1, AAc1 | actin 1 | chr2:15779761-	335
Dark-2	201	Background	AT2G37620	Symbols: ACT1, AAc1 | actin 1 | chr2:15779761-	335
Dark-2	201	Background	AT2G37620	Symbols: ACT1, AAc1 | actin 1 | chr2:15779761-	335
Dark-2	201	Background	AT2G37620	Symbols: ACT1, AAc1 | actin 1 | chr2:15779761-	335
Dark-2	201	Background	AT2G37620	Symbols: ACT1, AAc1 | actin 1 | chr2:15779761-	335
Dark-2	202	Background	AT2G21390	Symbols: | Coatomer, alpha subunit | chr2:915	327
Dark-2	202	Background	AT2G21390	Symbols: | Coatomer, alpha subunit | chr2:915	327
Dark-2	202	Background	AT2G21390	Symbols: | Coatomer, alpha subunit | chr2:915	327
Dark-2	202	Background	AT2G21390	Symbols: | Coatomer, alpha subunit | chr2:915	327
Dark-2	202	Background	AT2G21390	Symbols: | Coatomer, alpha subunit | chr2:915	327
Dark-2	202	Background	AT2G21390	Symbols: | Coatomer, alpha subunit | chr2:915	327
Dark-2	202	Background	AT2G21390	Symbols: | Coatomer, alpha subunit | chr2:915	327
Dark-2	202	Background	AT2G21390	Symbols: | Coatomer, alpha subunit | chr2:915	327
Dark-2	203	Background	AT5G36230	Symbols: | ARM repeat superfamily protein | ch	324
Dark-2	203	Background	AT5G36230	Symbols: | ARM repeat superfamily protein | ch	324
Dark-2	203	Background	AT5G36230	Symbols: | ARM repeat superfamily protein | ch	324
Dark-2	203	Background	AT5G36230	Symbols: | ARM repeat superfamily protein | ch	324
Dark-2	203	Background	AT5G36230	Symbols: | ARM repeat superfamily protein | ch	324
Dark-2	203	Background	AT5G36230	Symbols: | ARM repeat superfamily protein | ch	324
Dark-2	203	Background	AT5G36230	Symbols: | ARM repeat superfamily protein | ch	324
Dark-2	203	Background	AT5G36230	Symbols: | ARM repeat superfamily protein | ch	324
Dark-2	203	Background	AT5G36230	Symbols: | ARM repeat superfamily protein | ch	324
Dark-2	204	Background	AT2G15620	Symbols: NIR1, NIR, ATHNIR | nitrite reductase 1	323

Dark-2	204	Background	AT2G15620	Symbols: NIR1, NIR, ATHNIR | nitrite reductase 1	323
Dark-2	204	Background	AT2G15620	Symbols: NIR1, NIR, ATHNIR | nitrite reductase 1	323
Dark-2	204	Background	AT2G15620	Symbols: NIR1, NIR, ATHNIR | nitrite reductase 1	323
Dark-2	204	Background	AT2G15620	Symbols: NIR1, NIR, ATHNIR | nitrite reductase 1	323
Dark-2	204	Background	AT2G15620	Symbols: NIR1, NIR, ATHNIR | nitrite reductase 1	323
Dark-2	204	Background	AT2G15620	Symbols: NIR1, NIR, ATHNIR | nitrite reductase 1	323
Dark-2	204	Background	AT2G15620	Symbols: NIR1, NIR, ATHNIR | nitrite reductase 1	323
Dark-2	205	Background	AT4G16143	Symbols: IMPA-2 | importin alpha isoform 2 | ch	323
Dark-2	205	Background	AT4G16143	Symbols: IMPA-2 | importin alpha isoform 2 | ch	323
Dark-2	205	Background	AT4G16143	Symbols: IMPA-2 | importin alpha isoform 2 | ch	323
Dark-2	205	Background	AT4G16143	Symbols: IMPA-2 | importin alpha isoform 2 | ch	323
Dark-2	205	Background	AT4G16143	Symbols: IMPA-2 | importin alpha isoform 2 | ch	323
Dark-2	205	Background	AT4G16143	Symbols: IMPA-2 | importin alpha isoform 2 | ch	323
Dark-2	205	Background	AT4G16143	Symbols: IMPA-2 | importin alpha isoform 2 | ch	323
Dark-2	205	Background	AT4G16143	Symbols: IMPA-2 | importin alpha isoform 2 | ch	323
Dark-2	206	Background	AT1G55490	Symbols: CPN60B, LEN1 | chaperonin 60 beta | c	322
Dark-2	206	Background	AT1G55490	Symbols: CPN60B, LEN1 | chaperonin 60 beta | c	322
Dark-2	206	Background	AT1G55490	Symbols: CPN60B, LEN1 | chaperonin 60 beta | c	322
Dark-2	206	Background	AT1G55490	Symbols: CPN60B, LEN1 | chaperonin 60 beta | c	322
Dark-2	206	Background	AT1G55490	Symbols: CPN60B, LEN1 | chaperonin 60 beta | c	322
Dark-2	207	Background	AT2G07698	Symbols: | ATPase, F1 complex, alpha subunit p	321
Dark-2	207	Background	AT2G07698	Symbols: | ATPase, F1 complex, alpha subunit p	321
Dark-2	207	Background	AT2G07698	Symbols: | ATPase, F1 complex, alpha subunit p	321
Dark-2	207	Background	AT2G07698	Symbols: | ATPase, F1 complex, alpha subunit p	321
Dark-2	207	Background	AT2G07698	Symbols: | ATPase, F1 complex, alpha subunit p	321
Dark-2	207	Background	AT2G07698	Symbols: | ATPase, F1 complex, alpha subunit p	321
Dark-2	207	Background	AT2G07698	Symbols: | ATPase, F1 complex, alpha subunit p	321
Dark-2	207	Background	AT2G07698	Symbols: | ATPase, F1 complex, alpha subunit p	321
Dark-2	208	Background	AT1G29150	Symbols: ATS9, RPN6 | non-ATPase subunit 9 | c	319
Dark-2	208	Background	AT1G29150	Symbols: ATS9, RPN6 | non-ATPase subunit 9 | c	319
Dark-2	208	Background	AT1G29150	Symbols: ATS9, RPN6 | non-ATPase subunit 9 | c	319
Dark-2	208	Background	AT1G29150	Symbols: ATS9, RPN6 | non-ATPase subunit 9 | c	319
Dark-2	208	Background	AT1G29150	Symbols: ATS9, RPN6 | non-ATPase subunit 9 | c	319
Dark-2	208	Background	AT1G29150	Symbols: ATS9, RPN6 | non-ATPase subunit 9 | c	319
Dark-2	208	Background	AT1G29150	Symbols: ATS9, RPN6 | non-ATPase subunit 9 | c	319
Dark-2	208	Background	AT1G29150	Symbols: ATS9, RPN6 | non-ATPase subunit 9 | c	319
Dark-2	209	Background	AT2G33210	Symbols: HSP60-2 | heat shock protein 60-2 | ch	317
Dark-2	209	Background	AT2G33210	Symbols: HSP60-2 | heat shock protein 60-2 | ch	317
Dark-2	209	Background	AT2G33210	Symbols: HSP60-2 | heat shock protein 60-2 | ch	317
Dark-2	209	Background	AT2G33210	Symbols: HSP60-2 | heat shock protein 60-2 | ch	317
Dark-2	209	Background	AT2G33210	Symbols: HSP60-2 | heat shock protein 60-2 | ch	317
Dark-2	209	Background	AT2G33210	Symbols: HSP60-2 | heat shock protein 60-2 | ch	317
Dark-2	209	Background	AT2G33210	Symbols: HSP60-2 | heat shock protein 60-2 | ch	317
Dark-2	209	Background	AT2G33210	Symbols: HSP60-2 | heat shock protein 60-2 | ch	317
Dark-2	209	Background	AT2G33210	Symbols: HSP60-2 | heat shock protein 60-2 | ch	317
Dark-2	210	Background	AT3G44110	Symbols: ATJ3, ATJ | DNAJ homologue 3 | chr3:1	316
Dark-2	210	Background	AT3G44110	Symbols: ATJ3, ATJ | DNAJ homologue 3 | chr3:1	316
Dark-2	210	Background	AT3G44110	Symbols: ATJ3, ATJ | DNAJ homologue 3 | chr3:1	316
Dark-2	210	Background	AT3G44110	Symbols: ATJ3, ATJ | DNAJ homologue 3 | chr3:1	316
Dark-2	210	Background	AT3G44110	Symbols: ATJ3, ATJ | DNAJ homologue 3 | chr3:1	316
Dark-2	210	Background	AT3G44110	Symbols: ATJ3, ATJ | DNAJ homologue 3 | chr3:1	316
Dark-2	210	Background	AT3G44110	Symbols: ATJ3, ATJ | DNAJ homologue 3 | chr3:1	316
Dark-2	210	Background	AT3G44110	Symbols: ATJ3, ATJ | DNAJ homologue 3 | chr3:1	316
Dark-2	210	Background	AT3G44110	Symbols: ATJ3, ATJ | DNAJ homologue 3 | chr3:1	316
Dark-2	211	Background	AT3G17390	Symbols: MTO3, SAMS3, MAT4 | S-adenosylmet	315
Dark-2	211	Background	AT3G17390	Symbols: MTO3, SAMS3, MAT4 | S-adenosylmet	315
Dark-2	211	Background	AT3G17390	Symbols: MTO3, SAMS3, MAT4 | S-adenosylmet	315
Dark-2	211	Background	AT3G17390	Symbols: MTO3, SAMS3, MAT4 | S-adenosylmet	315

Dark-2	211	Background	AT3G17390	Symbols: MTO3, SAMS3, MAT4 | S-adenosylmet	315
Dark-2	211	Background	AT3G17390	Symbols: MTO3, SAMS3, MAT4 | S-adenosylmet	315
Dark-2	211	Background	AT3G17390	Symbols: MTO3, SAMS3, MAT4 | S-adenosylmet	315
Dark-2	211	Background	AT3G17390	Symbols: MTO3, SAMS3, MAT4 | S-adenosylmet	315
Dark-2	211	Background	AT3G17390	Symbols: MTO3, SAMS3, MAT4 | S-adenosylmet	315
Dark-2	211	Background	AT3G17390	Symbols: MTO3, SAMS3, MAT4 | S-adenosylmet	315
Dark-2	211	Background	AT3G17390	Symbols: MTO3, SAMS3, MAT4 | S-adenosylmet	315
Dark-2	212	Background	AT5G19990	Symbols: RPT6A, ATSUG1 | regulatory particle tr	314
Dark-2	212	Background	AT5G19990	Symbols: RPT6A, ATSUG1 | regulatory particle tr	314
Dark-2	212	Background	AT5G19990	Symbols: RPT6A, ATSUG1 | regulatory particle tr	314
Dark-2	212	Background	AT5G19990	Symbols: RPT6A, ATSUG1 | regulatory particle tr	314
Dark-2	212	Background	AT5G19990	Symbols: RPT6A, ATSUG1 | regulatory particle tr	314
Dark-2	212	Background	AT5G19990	Symbols: RPT6A, ATSUG1 | regulatory particle tr	314
Dark-2	212	Background	AT5G19990	Symbols: RPT6A, ATSUG1 | regulatory particle tr	314
Dark-2	213	Background	AT1G10490	Symbols: | Domain of unknown function (DUF1	313
Dark-2	213	Background	AT1G10490	Symbols: | Domain of unknown function (DUF1	313
Dark-2	213	Background	AT1G10490	Symbols: | Domain of unknown function (DUF1	313
Dark-2	213	Background	AT1G10490	Symbols: | Domain of unknown function (DUF1	313
Dark-2	213	Background	AT1G10490	Symbols: | Domain of unknown function (DUF1	313
Dark-2	214	Background	AT5G17020	Symbols: XPO1A, ATCRM1, ATXPO1, XPO1, HIT2	304
Dark-2	214	Background	AT5G17020	Symbols: XPO1A, ATCRM1, ATXPO1, XPO1, HIT2	304
Dark-2	214	Background	AT5G17020	Symbols: XPO1A, ATCRM1, ATXPO1, XPO1, HIT2	304
Dark-2	214	Background	AT5G17020	Symbols: XPO1A, ATCRM1, ATXPO1, XPO1, HIT2	304
Dark-2	214	Background	AT5G17020	Symbols: XPO1A, ATCRM1, ATXPO1, XPO1, HIT2	304
Dark-2	214	Background	AT5G17020	Symbols: XPO1A, ATCRM1, ATXPO1, XPO1, HIT2	304
Dark-2	215	Background	AT3G20050	Symbols: ATTCP-1, TCP-1 | T-complex protein 1	303
Dark-2	215	Background	AT3G20050	Symbols: ATTCP-1, TCP-1 | T-complex protein 1	303
Dark-2	215	Background	AT3G20050	Symbols: ATTCP-1, TCP-1 | T-complex protein 1	303
Dark-2	215	Background	AT3G20050	Symbols: ATTCP-1, TCP-1 | T-complex protein 1	303
Dark-2	215	Background	AT3G20050	Symbols: ATTCP-1, TCP-1 | T-complex protein 1	303
Dark-2	216	Background	AT1G49240	Symbols: ACT8 | actin 8 | chr1:18216539-18217	300
Dark-2	216	Background	AT1G49240	Symbols: ACT8 | actin 8 | chr1:18216539-18217	300
Dark-2	216	Background	AT1G49240	Symbols: ACT8 | actin 8 | chr1:18216539-18217	300
Dark-2	216	Background	AT1G49240	Symbols: ACT8 | actin 8 | chr1:18216539-18217	300
Dark-2	216	Background	AT1G49240	Symbols: ACT8 | actin 8 | chr1:18216539-18217	300
Dark-2	216	Background	AT1G49240	Symbols: ACT8 | actin 8 | chr1:18216539-18217	300
Dark-2	216	Background	AT1G49240	Symbols: ACT8 | actin 8 | chr1:18216539-18217	300
Dark-2	216	Background	AT1G49240	Symbols: ACT8 | actin 8 | chr1:18216539-18217	300
Dark-2	217	Background	AT5G18700	Symbols: RUK, EMB3013 | Protein kinase family	299
Dark-2	217	Background	AT5G18700	Symbols: RUK, EMB3013 | Protein kinase family	299
Dark-2	217	Background	AT5G18700	Symbols: RUK, EMB3013 | Protein kinase family	299
Dark-2	217	Background	AT5G18700	Symbols: RUK, EMB3013 | Protein kinase family	299
Dark-2	217	Background	AT5G18700	Symbols: RUK, EMB3013 | Protein kinase family	299
Dark-2	217	Background	AT5G18700	Symbols: RUK, EMB3013 | Protein kinase family	299
Dark-2	217	Background	AT5G18700	Symbols: RUK, EMB3013 | Protein kinase family	299
Dark-2	218	Background	AT3G12050	Symbols: | Aha1 domain-containing protein | ch	296
Dark-2	218	Background	AT3G12050	Symbols: | Aha1 domain-containing protein | ch	296
Dark-2	218	Background	AT3G12050	Symbols: | Aha1 domain-containing protein | ch	296
Dark-2	218	Background	AT3G12050	Symbols: | Aha1 domain-containing protein | ch	296
Dark-2	218	Background	AT3G12050	Symbols: | Aha1 domain-containing protein | ch	296
Dark-2	219	Background	AT4G24820	Symbols: | 26S proteasome, regulatory subunit	283
Dark-2	219	Background	AT4G24820	Symbols: | 26S proteasome, regulatory subunit	283
Dark-2	219	Background	AT4G24820	Symbols: | 26S proteasome, regulatory subunit	283
Dark-2	219	Background	AT4G24820	Symbols: | 26S proteasome, regulatory subunit	283
Dark-2	219	Background	AT4G24820	Symbols: | 26S proteasome, regulatory subunit	283
Dark-2	219	Background	AT4G24820	Symbols: | 26S proteasome, regulatory subunit	283
Dark-2	220	Background	AT2G16950	Symbols: TRN1, ATTRN1 | transportin 1 | chr2:7	283
Dark-2	220	Background	AT2G16950	Symbols: TRN1, ATTRN1 | transportin 1 | chr2:7	283

Dark-2	220	Background	AT2G16950	Symbols: TRN1, ATTRN1 | transportin 1 | chr2:7	283
Dark-2	220	Background	AT2G16950	Symbols: TRN1, ATTRN1 | transportin 1 | chr2:7	283
Dark-2	220	Background	AT2G16950	Symbols: TRN1, ATTRN1 | transportin 1 | chr2:7	283
Dark-2	220	Background	AT2G16950	Symbols: TRN1, ATTRN1 | transportin 1 | chr2:7	283
Dark-2	220	Background	AT2G16950	Symbols: TRN1, ATTRN1 | transportin 1 | chr2:7	283
Dark-2	220	Background	AT2G16950	Symbols: TRN1, ATTRN1 | transportin 1 | chr2:7	283
Dark-2	220	Background	AT2G16950	Symbols: TRN1, ATTRN1 | transportin 1 | chr2:7	283
Dark-2	221	Background	AT4G28470	Symbols: RPN1B, ATRPN1B | 26S proteasome re	276
Dark-2	221	Background	AT4G28470	Symbols: RPN1B, ATRPN1B | 26S proteasome re	276
Dark-2	221	Background	AT4G28470	Symbols: RPN1B, ATRPN1B | 26S proteasome re	276
Dark-2	221	Background	AT4G28470	Symbols: RPN1B, ATRPN1B | 26S proteasome re	276
Dark-2	221	Background	AT4G28470	Symbols: RPN1B, ATRPN1B | 26S proteasome re	276
Dark-2	221	Background	AT4G28470	Symbols: RPN1B, ATRPN1B | 26S proteasome re	276
Dark-2	222	Background	AT5G20890	Symbols: | TCP-1/cpn60 chaperonin family prot	271
Dark-2	222	Background	AT5G20890	Symbols: | TCP-1/cpn60 chaperonin family prot	271
Dark-2	222	Background	AT5G20890	Symbols: | TCP-1/cpn60 chaperonin family prot	271
Dark-2	222	Background	AT5G20890	Symbols: | TCP-1/cpn60 chaperonin family prot	271
Dark-2	222	Background	AT5G20890	Symbols: | TCP-1/cpn60 chaperonin family prot	271
Dark-2	222	Background	AT5G20890	Symbols: | TCP-1/cpn60 chaperonin family prot	271
Dark-2	222	Background	AT5G20890	Symbols: | TCP-1/cpn60 chaperonin family prot	271
Dark-2	222	Background	AT5G20890	Symbols: | TCP-1/cpn60 chaperonin family prot	271
Dark-2	222	Background	AT5G20890	Symbols: | TCP-1/cpn60 chaperonin family prot	271
Dark-2	223	Background	AT2G35630	Symbols: MOR1, GEM1 | ARM repeat superfami	267
Dark-2	223	Background	AT2G35630	Symbols: MOR1, GEM1 | ARM repeat superfami	267
Dark-2	223	Background	AT2G35630	Symbols: MOR1, GEM1 | ARM repeat superfami	267
Dark-2	223	Background	AT2G35630	Symbols: MOR1, GEM1 | ARM repeat superfami	267
Dark-2	223	Background	AT2G35630	Symbols: MOR1, GEM1 | ARM repeat superfami	267
Dark-2	224	Background	AT3G23990	Symbols: HSP60, HSP60-3B | heat shock protein	261
Dark-2	224	Background	AT3G23990	Symbols: HSP60, HSP60-3B | heat shock protein	261
Dark-2	224	Background	AT3G23990	Symbols: HSP60, HSP60-3B | heat shock protein	261
Dark-2	224	Background	AT3G23990	Symbols: HSP60, HSP60-3B | heat shock protein	261
Dark-2	224	Background	AT3G23990	Symbols: HSP60, HSP60-3B | heat shock protein	261
Dark-2	224	Background	AT3G23990	Symbols: HSP60, HSP60-3B | heat shock protein	261
Dark-2	224	Background	AT3G23990	Symbols: HSP60, HSP60-3B | heat shock protein	261
Dark-2	224	Background	AT3G23990	Symbols: HSP60, HSP60-3B | heat shock protein	261
Dark-2	225	Background	AT2G17130	Symbols: IDH2, IDH-II | isocitrate dehydrogenase	257
Dark-2	225	Background	AT2G17130	Symbols: IDH2, IDH-II | isocitrate dehydrogenase	257
Dark-2	225	Background	AT2G17130	Symbols: IDH2, IDH-II | isocitrate dehydrogenase	257
Dark-2	225	Background	AT2G17130	Symbols: IDH2, IDH-II | isocitrate dehydrogenase	257
Dark-2	225	Background	AT2G17130	Symbols: IDH2, IDH-II | isocitrate dehydrogenase	257
Dark-2	226	Background	AT5G23540	Symbols: | Mov34/MPN/PAD-1 family protein |	247
Dark-2	226	Background	AT5G23540	Symbols: | Mov34/MPN/PAD-1 family protein |	247
Dark-2	226	Background	AT5G23540	Symbols: | Mov34/MPN/PAD-1 family protein |	247
Dark-2	226	Background	AT5G23540	Symbols: | Mov34/MPN/PAD-1 family protein |	247
Dark-2	226	Background	AT5G23540	Symbols: | Mov34/MPN/PAD-1 family protein |	247
Dark-2	227	Background	AT5G12370	Symbols: SEC10 | exocyst complex component s	246
Dark-2	227	Background	AT5G12370	Symbols: SEC10 | exocyst complex component s	246
Dark-2	227	Background	AT5G12370	Symbols: SEC10 | exocyst complex component s	246
Dark-2	227	Background	AT5G12370	Symbols: SEC10 | exocyst complex component s	246
Dark-2	227	Background	AT5G12370	Symbols: SEC10 | exocyst complex component s	246
Dark-2	227	Background	AT5G12370	Symbols: SEC10 | exocyst complex component s	246
Dark-2	228	Background	AT3G09630	Symbols: | Ribosomal protein L4/L1 family | chr	243
Dark-2	228	Background	AT3G09630	Symbols: | Ribosomal protein L4/L1 family | chr	243
Dark-2	228	Background	AT3G09630	Symbols: | Ribosomal protein L4/L1 family | chr	243
Dark-2	228	Background	AT3G09630	Symbols: | Ribosomal protein L4/L1 family | chr	243
Dark-2	228	Background	AT3G09630	Symbols: | Ribosomal protein L4/L1 family | chr	243
Dark-2	228	Background	AT3G09630	Symbols: | Ribosomal protein L4/L1 family | chr	243
Dark-2	228	Background	AT3G09630	Symbols: | Ribosomal protein L4/L1 family | chr	243

Dark-2	229	Background	AT5G58290	Symbols: RPT3 | regulatory particle triple-A ATPa	242
Dark-2	229	Background	AT5G58290	Symbols: RPT3 | regulatory particle triple-A ATPa	242
Dark-2	229	Background	AT5G58290	Symbols: RPT3 | regulatory particle triple-A ATPa	242
Dark-2	229	Background	AT5G58290	Symbols: RPT3 | regulatory particle triple-A ATPa	242
Dark-2	229	Background	AT5G58290	Symbols: RPT3 | regulatory particle triple-A ATPa	242
Dark-2	229	Background	AT5G58290	Symbols: RPT3 | regulatory particle triple-A ATPa	242
Dark-2	229	Background	AT5G58290	Symbols: RPT3 | regulatory particle triple-A ATPa	242
Dark-2	230	Background	AT5G56010	Symbols: HSP81-3, Hsp81.3, AtHsp90-3, AtHsp90	240
Dark-2	230	Background	AT5G56010	Symbols: HSP81-3, Hsp81.3, AtHsp90-3, AtHsp90	240
Dark-2	230	Background	AT5G56010	Symbols: HSP81-3, Hsp81.3, AtHsp90-3, AtHsp90	240
Dark-2	230	Background	AT5G56010	Symbols: HSP81-3, Hsp81.3, AtHsp90-3, AtHsp90	240
Dark-2	230	Background	AT5G56010	Symbols: HSP81-3, Hsp81.3, AtHsp90-3, AtHsp90	240
Dark-2	230	Background	AT5G56010	Symbols: HSP81-3, Hsp81.3, AtHsp90-3, AtHsp90	240
Dark-2	231	Background	AT5G14040	Symbols: PHT3;1 | phosphate transporter 3;1 | c	237
Dark-2	231	Background	AT5G14040	Symbols: PHT3;1 | phosphate transporter 3;1 | c	237
Dark-2	231	Background	AT5G14040	Symbols: PHT3;1 | phosphate transporter 3;1 | c	237
Dark-2	231	Background	AT5G14040	Symbols: PHT3;1 | phosphate transporter 3;1 | c	237
Dark-2	231	Background	AT5G14040	Symbols: PHT3;1 | phosphate transporter 3;1 | c	237
Dark-2	232	Background	AT3G11250	Symbols: | Ribosomal protein L10 family protein	234
Dark-2	232	Background	AT3G11250	Symbols: | Ribosomal protein L10 family protein	234
Dark-2	232	Background	AT3G11250	Symbols: | Ribosomal protein L10 family protein	234
Dark-2	233	Background	AT3G25520	Symbols: ATL5, PGY3, OLI5, RPL5A | ribosomal p	231
Dark-2	233	Background	AT3G25520	Symbols: ATL5, PGY3, OLI5, RPL5A | ribosomal p	231
Dark-2	234	Background	AT5G28540	Symbols: BIP1 | heat shock protein 70 (Hsp 70) f	231
Dark-2	234	Background	AT5G28540	Symbols: BIP1 | heat shock protein 70 (Hsp 70) f	231
Dark-2	234	Background	AT5G28540	Symbols: BIP1 | heat shock protein 70 (Hsp 70) f	231
Dark-2	234	Background	AT5G28540	Symbols: BIP1 | heat shock protein 70 (Hsp 70) f	231
Dark-2	234	Background	AT5G28540	Symbols: BIP1 | heat shock protein 70 (Hsp 70) f	231
Dark-2	235	Background	AT2G31610	Symbols: | Ribosomal protein S3 family protein	225
Dark-2	235	Background	AT2G31610	Symbols: | Ribosomal protein S3 family protein	225
Dark-2	235	Background	AT2G31610	Symbols: | Ribosomal protein S3 family protein	225
Dark-2	236	Background	AT5G02870	Symbols: | Ribosomal protein L4/L1 family | chr	224
Dark-2	236	Background	AT5G02870	Symbols: | Ribosomal protein L4/L1 family | chr	224
Dark-2	236	Background	AT5G02870	Symbols: | Ribosomal protein L4/L1 family | chr	224
Dark-2	236	Background	AT5G02870	Symbols: | Ribosomal protein L4/L1 family | chr	224
Dark-2	236	Background	AT5G02870	Symbols: | Ribosomal protein L4/L1 family | chr	224
Dark-2	236	Background	AT5G02870	Symbols: | Ribosomal protein L4/L1 family | chr	224
Dark-2	237	Background	AT5G47690	Symbols: | binding | chr5:19317899-19327014	224
Dark-2	237	Background	AT5G47690	Symbols: | binding | chr5:19317899-19327014	224
Dark-2	237	Background	AT5G47690	Symbols: | binding | chr5:19317899-19327014	224
Dark-2	238	Background	AT1G15480	Symbols: | Tetratricopeptide repeat (TPR)-like s	221
Dark-2	238	Background	AT1G15480	Symbols: | Tetratricopeptide repeat (TPR)-like s	221
Dark-2	238	Background	AT1G15480	Symbols: | Tetratricopeptide repeat (TPR)-like s	221
Dark-2	238	Background	AT1G15480	Symbols: | Tetratricopeptide repeat (TPR)-like s	221
Dark-2	239	Background	AT5G20290	Symbols: | Ribosomal protein S8e family protein	218
Dark-2	239	Background	AT5G20290	Symbols: | Ribosomal protein S8e family protein	218
Dark-2	239	Background	AT5G20290	Symbols: | Ribosomal protein S8e family protein	218
Dark-2	239	Background	AT5G20290	Symbols: | Ribosomal protein S8e family protein	218
Dark-2	239	Background	AT5G20290	Symbols: | Ribosomal protein S8e family protein	218
Dark-2	240	Background	AT1G53750	Symbols: RPT1A | regulatory particle triple-A 1A	217
Dark-2	240	Background	AT1G53750	Symbols: RPT1A | regulatory particle triple-A 1A	217
Dark-2	240	Background	AT1G53750	Symbols: RPT1A | regulatory particle triple-A 1A	217
Dark-2	240	Background	AT1G53750	Symbols: RPT1A | regulatory particle triple-A 1A	217
Dark-2	240	Background	AT1G53750	Symbols: RPT1A | regulatory particle triple-A 1A	217
Dark-2	241	Background	AT4G11380	Symbols: | Adaptin family protein | chr4:692060	215
Dark-2	241	Background	AT4G11380	Symbols: | Adaptin family protein | chr4:692060	215
Dark-2	241	Background	AT4G11380	Symbols: | Adaptin family protein | chr4:692060	215
Dark-2	241	Background	AT4G11380	Symbols: | Adaptin family protein | chr4:692060	215

Dark-2	241	Background	AT4G11380	Symbols: | Adaptin family protein | chr4:69206	215
Dark-2	242	Background	AT2G46900	Symbols: | CONTAINS InterPro DOMAIN/s: Basi	210
Dark-2	242	Background	AT2G46900	Symbols: | CONTAINS InterPro DOMAIN/s: Basi	210
Dark-2	242	Background	AT2G46900	Symbols: | CONTAINS InterPro DOMAIN/s: Basi	210
Dark-2	243	Background	AT5G56500	Symbols: | TCP-1/cpn60 chaperonin family prot	207
Dark-2	243	Background	AT5G56500	Symbols: | TCP-1/cpn60 chaperonin family prot	207
Dark-2	243	Background	AT5G56500	Symbols: | TCP-1/cpn60 chaperonin family prot	207
Dark-2	243	Background	AT5G56500	Symbols: | TCP-1/cpn60 chaperonin family prot	207
Dark-2	243	Background	AT5G56500	Symbols: | TCP-1/cpn60 chaperonin family prot	207
Dark-2	244	Background	AT3G13160	Symbols: | Tetratricopeptide repeat (TPR)-like s	207
Dark-2	244	Background	AT3G13160	Symbols: | Tetratricopeptide repeat (TPR)-like s	207
Dark-2	245	Background	AT3G07050	Symbols: | GTP-binding family protein | chr3:22	206
Dark-2	245	Background	AT3G07050	Symbols: | GTP-binding family protein | chr3:22	206
Dark-2	246	Background	AT4G23460	Symbols: | Adaptin family protein | chr4:12243	204
Dark-2	246	Background	AT4G23460	Symbols: | Adaptin family protein | chr4:12243	204
Dark-2	246	Background	AT4G23460	Symbols: | Adaptin family protein | chr4:12243	204
Dark-2	246	Background	AT4G23460	Symbols: | Adaptin family protein | chr4:12243	204
Dark-2	246	Background	AT4G23460	Symbols: | Adaptin family protein | chr4:12243	204
Dark-2	247	Background	AT3G59020	Symbols: | ARM repeat superfamily protein | ch	203
Dark-2	247	Background	AT3G59020	Symbols: | ARM repeat superfamily protein | ch	203
Dark-2	247	Background	AT3G59020	Symbols: | ARM repeat superfamily protein | ch	203
Dark-2	247	Background	AT3G59020	Symbols: | ARM repeat superfamily protein | ch	203
Dark-2	247	Background	AT3G59020	Symbols: | ARM repeat superfamily protein | ch	203
Dark-2	248	Background	AT3G11830	Symbols: | TCP-1/cpn60 chaperonin family prot	202
Dark-2	248	Background	AT3G11830	Symbols: | TCP-1/cpn60 chaperonin family prot	202
Dark-2	248	Background	AT3G11830	Symbols: | TCP-1/cpn60 chaperonin family prot	202
Dark-2	249	Background	AT1G36180	Symbols: ACC2 | acetyl-CoA carboxylase 2 | chr1	200
Dark-2	249	Background	AT1G36180	Symbols: ACC2 | acetyl-CoA carboxylase 2 | chr1	200
Dark-2	249	Background	AT1G36180	Symbols: ACC2 | acetyl-CoA carboxylase 2 | chr1	200
Dark-2	249	Background	AT1G36180	Symbols: ACC2 | acetyl-CoA carboxylase 2 | chr1	200
Dark-2	250	Background	AT1G43170	Symbols: ARP1, emb2207, RPL3A, RP1 | ribosom	199
Dark-2	250	Background	AT1G43170	Symbols: ARP1, emb2207, RPL3A, RP1 | ribosom	199
Dark-2	250	Background	AT1G43170	Symbols: ARP1, emb2207, RPL3A, RP1 | ribosom	199
Dark-2	250	Background	AT1G43170	Symbols: ARP1, emb2207, RPL3A, RP1 | ribosom	199
Dark-2	250	Background	AT1G43170	Symbols: ARP1, emb2207, RPL3A, RP1 | ribosom	199
Dark-2	250	Background	AT1G43170	Symbols: ARP1, emb2207, RPL3A, RP1 | ribosom	199
Dark-2	250	Background	AT1G43170	Symbols: ARP1, emb2207, RPL3A, RP1 | ribosom	199
Dark-2	250	Background	AT1G43170	Symbols: ARP1, emb2207, RPL3A, RP1 | ribosom	199
Dark-2	250	Background	AT1G43170	Symbols: ARP1, emb2207, RPL3A, RP1 | ribosom	199
Dark-2	251	Background	AT1G20200	Symbols: EMB2719, HAP15 | PAM domain (PCI/	195
Dark-2	251	Background	AT1G20200	Symbols: EMB2719, HAP15 | PAM domain (PCI/	195
Dark-2	251	Background	AT1G20200	Symbols: EMB2719, HAP15 | PAM domain (PCI/	195
Dark-2	251	Background	AT1G20200	Symbols: EMB2719, HAP15 | PAM domain (PCI/	195
Dark-2	251	Background	AT1G20200	Symbols: EMB2719, HAP15 | PAM domain (PCI/	195
Dark-2	251	Background	AT1G20200	Symbols: EMB2719, HAP15 | PAM domain (PCI/	195
Dark-2	252	Background	AT5G51430	Symbols: EYE | conserved oligomeric Golgi comp	192
Dark-2	252	Background	AT5G51430	Symbols: EYE | conserved oligomeric Golgi comp	192
Dark-2	253	Background	AT4G17530	Symbols: RAB1C, ATRAB1C, ATRABD2C | RAB GT	191
Dark-2	253	Background	AT4G17530	Symbols: RAB1C, ATRAB1C, ATRABD2C | RAB GT	191
Dark-2	253	Background	AT4G17530	Symbols: RAB1C, ATRAB1C, ATRABD2C | RAB GT	191
Dark-2	253	Background	AT4G17530	Symbols: RAB1C, ATRAB1C, ATRABD2C | RAB GT	191
Dark-2	254	Background	AT2G39990	Symbols: EIF2, AteIF3f, eIF3F | eukaryotic transl	189
Dark-2	254	Background	AT2G39990	Symbols: EIF2, AteIF3f, eIF3F | eukaryotic transl	189
Dark-2	254	Background	AT2G39990	Symbols: EIF2, AteIF3f, eIF3F | eukaryotic transl	189
Dark-2	254	Background	AT2G39990	Symbols: EIF2, AteIF3f, eIF3F | eukaryotic transl	189
Dark-2	254	Background	AT2G39990	Symbols: EIF2, AteIF3f, eIF3F | eukaryotic transl	189
Dark-2	255	Background	AT1G22780	Symbols: PFL, RPS18A, PFL1 | Ribosomal protein	187
Dark-2	255	Background	AT1G22780	Symbols: PFL, RPS18A, PFL1 | Ribosomal protein	187

Dark-2	255	Background	AT1G22780	Symbols: PFL, RPS18A, PFL1 | Ribosomal protein	187
Dark-2	256	Background	AT2G37270	Symbols: ATRPS5B, RPS5B | ribosomal protein 5	187
Dark-2	256	Background	AT2G37270	Symbols: ATRPS5B, RPS5B | ribosomal protein 5	187
Dark-2	256	Background	AT2G37270	Symbols: ATRPS5B, RPS5B | ribosomal protein 5	187
Dark-2	257	Background	AT5G15090	Symbols: VDAC3, ATVDAC3 | voltage dependent	186
Dark-2	257	Background	AT5G15090	Symbols: VDAC3, ATVDAC3 | voltage dependent	186
Dark-2	257	Background	AT5G15090	Symbols: VDAC3, ATVDAC3 | voltage dependent	186
Dark-2	257	Background	AT5G15090	Symbols: VDAC3, ATVDAC3 | voltage dependent	186
Dark-2	258	Background	AT4G02930	Symbols: | GTP binding Elongation factor Tu fan	181
Dark-2	258	Background	AT4G02930	Symbols: | GTP binding Elongation factor Tu fan	181
Dark-2	258	Background	AT4G02930	Symbols: | GTP binding Elongation factor Tu fan	181
Dark-2	259	Background	AT5G19760	Symbols: | Mitochondrial substrate carrier fami	178
Dark-2	259	Background	AT5G19760	Symbols: | Mitochondrial substrate carrier fami	178
Dark-2	259	Background	AT5G19760	Symbols: | Mitochondrial substrate carrier fami	178
Dark-2	259	Background	AT5G19760	Symbols: | Mitochondrial substrate carrier fami	178
Dark-2	259	Background	AT5G19760	Symbols: | Mitochondrial substrate carrier fami	178
Dark-2	260	Background	AT3G10380	Symbols: SEC8, ATSEC8 | subunit of exocyst com	178
Dark-2	260	Background	AT3G10380	Symbols: SEC8, ATSEC8 | subunit of exocyst com	178
Dark-2	261	Background	AT3G08530	Symbols: | Clathrin, heavy chain | chr3:258717	174
Dark-2	261	Background	AT3G08530	Symbols: | Clathrin, heavy chain | chr3:258717	174
Dark-2	261	Background	AT3G08530	Symbols: | Clathrin, heavy chain | chr3:258717	174
Dark-2	261	Background	AT3G08530	Symbols: | Clathrin, heavy chain | chr3:258717	174
Dark-2	262	Background	AT4G34450	Symbols: | coatomer gamma-2 subunit, putative	174
Dark-2	262	Background	AT4G34450	Symbols: | coatomer gamma-2 subunit, putative	174
Dark-2	262	Background	AT4G34450	Symbols: | coatomer gamma-2 subunit, putative	174
Dark-2	263	Background	AT3G57290	Symbols: EIF3E, TIF3E1, ATEIF3E-1, INT-6, ATINT	171
Dark-2	263	Background	AT3G57290	Symbols: EIF3E, TIF3E1, ATEIF3E-1, INT-6, ATINT	171
Dark-2	263	Background	AT3G57290	Symbols: EIF3E, TIF3E1, ATEIF3E-1, INT-6, ATINT	171
Dark-2	263	Background	AT3G57290	Symbols: EIF3E, TIF3E1, ATEIF3E-1, INT-6, ATINT	171
Dark-2	263	Background	AT3G57290	Symbols: EIF3E, TIF3E1, ATEIF3E-1, INT-6, ATINT	171
Dark-2	264	Background	AT1G65220	Symbols: | ARM repeat superfamily protein | ch	169
Dark-2	264	Background	AT1G65220	Symbols: | ARM repeat superfamily protein | ch	169
Dark-2	264	Background	AT1G65220	Symbols: | ARM repeat superfamily protein | ch	169
Dark-2	264	Background	AT1G65220	Symbols: | ARM repeat superfamily protein | ch	169
Dark-2	264	Background	AT1G65220	Symbols: | ARM repeat superfamily protein | ch	169
Dark-2	265	Background	AT1G64520	Symbols: RPN12a | regulatory particle non-ATPa	168
Dark-2	265	Background	AT1G64520	Symbols: RPN12a | regulatory particle non-ATPa	168
Dark-2	265	Background	AT1G64520	Symbols: RPN12a | regulatory particle non-ATPa	168
Dark-2	266	Background	AT5G49910	Symbols: CPHSC70-2EAT SHOCK PROTEIN 70-2, I	167
Dark-2	266	Background	AT5G49910	Symbols: CPHSC70-2EAT SHOCK PROTEIN 70-2, I	167
Dark-2	266	Background	AT5G49910	Symbols: CPHSC70-2EAT SHOCK PROTEIN 70-2, I	167
Dark-2	266	Background	AT5G49910	Symbols: CPHSC70-2EAT SHOCK PROTEIN 70-2, I	167
Dark-2	267	Background	AT2G44120	Symbols: | Ribosomal protein L30/L7 family pro	167
Dark-2	267	Background	AT2G44120	Symbols: | Ribosomal protein L30/L7 family pro	167
Dark-2	267	Background	AT2G44120	Symbols: | Ribosomal protein L30/L7 family pro	167
Dark-2	267	Background	AT2G44120	Symbols: | Ribosomal protein L30/L7 family pro	167
Dark-2	268	Background	AT3G10690	Symbols: GYRA | DNA GYRASE A | chr3:3339612	166
Dark-2	268	Background	AT3G10690	Symbols: GYRA | DNA GYRASE A | chr3:3339612	166
Dark-2	268	Background	AT3G10690	Symbols: GYRA | DNA GYRASE A | chr3:3339612	166
Dark-2	268	Background	AT3G10690	Symbols: GYRA | DNA GYRASE A | chr3:3339612	166
Dark-2	269	Background	AT2G20450	Symbols: | Ribosomal protein L14 | chr2:88139	164
Dark-2	269	Background	AT2G20450	Symbols: | Ribosomal protein L14 | chr2:88139	164
Dark-2	270	Background	AT1G01960	Symbols: EDA10 | SEC7-like guanine nucleotide	164
Dark-2	270	Background	AT1G01960	Symbols: EDA10 | SEC7-like guanine nucleotide	164
Dark-2	270	Background	AT1G01960	Symbols: EDA10 | SEC7-like guanine nucleotide	164
Dark-2	271	Background	AT3G54110	Symbols: ATPUMP1, UCP, PUMP1, ATUCP1, UCP	162
Dark-2	271	Background	AT3G54110	Symbols: ATPUMP1, UCP, PUMP1, ATUCP1, UCP	162
Dark-2	271	Background	AT3G54110	Symbols: ATPUMP1, UCP, PUMP1, ATUCP1, UCP	162

Dark-2	271	Background	AT3G54110	Symbols: ATPUMP1, UCP, PUMP1, ATUCP1, UCP	162
Dark-2	272	Background	AT1G02080	Symbols: | transcription regulators | chr1:37333	162
Dark-2	272	Background	AT1G02080	Symbols: | transcription regulators | chr1:37333	162
Dark-2	272	Background	AT1G02080	Symbols: | transcription regulators | chr1:37333	162
Dark-2	272	Background	AT1G02080	Symbols: | transcription regulators | chr1:37333	162
Dark-2	273	Background	AT3G04840	Symbols: | Ribosomal protein S3Ae | chr3:1329	162
Dark-2	273	Background	AT3G04840	Symbols: | Ribosomal protein S3Ae | chr3:1329	162
Dark-2	273	Background	AT3G04840	Symbols: | Ribosomal protein S3Ae | chr3:1329	162
Dark-2	273	Background	AT3G04840	Symbols: | Ribosomal protein S3Ae | chr3:1329	162
Dark-2	274	Background	AT5G26742	Symbols: emb1138 | DEAD box RNA helicase (RH	161
Dark-2	274	Background	AT5G26742	Symbols: emb1138 | DEAD box RNA helicase (RH	161
Dark-2	274	Background	AT5G26742	Symbols: emb1138 | DEAD box RNA helicase (RH	161
Dark-2	274	Background	AT5G26742	Symbols: emb1138 | DEAD box RNA helicase (RH	161
Dark-2	274	Background	AT5G26742	Symbols: emb1138 | DEAD box RNA helicase (RH	161
Dark-2	274	Background	AT5G26742	Symbols: emb1138 | DEAD box RNA helicase (RH	161
Dark-2	275	Background	AT3G49870	Symbols: ATARLA1C, ARLA1C | ADP-ribosylation	161
Dark-2	275	Background	AT3G49870	Symbols: ATARLA1C, ARLA1C | ADP-ribosylation	161
Dark-2	275	Background	AT3G49870	Symbols: ATARLA1C, ARLA1C | ADP-ribosylation	161
Dark-2	276	Background	AT3G02530	Symbols: | TCP-1/cpn60 chaperonin family prot	159
Dark-2	276	Background	AT3G02530	Symbols: | TCP-1/cpn60 chaperonin family prot	159
Dark-2	276	Background	AT3G02530	Symbols: | TCP-1/cpn60 chaperonin family prot	159
Dark-2	276	Background	AT3G02530	Symbols: | TCP-1/cpn60 chaperonin family prot	159
Dark-2	277	Background	AT2G44610	Symbols: RAB6, ATRABH1B, ATRAB6A, RAB6A |	159
Dark-2	277	Background	AT2G44610	Symbols: RAB6, ATRABH1B, ATRAB6A, RAB6A |	159
Dark-2	277	Background	AT2G44610	Symbols: RAB6, ATRABH1B, ATRAB6A, RAB6A |	159
Dark-2	277	Background	AT2G44610	Symbols: RAB6, ATRABH1B, ATRAB6A, RAB6A |	159
Dark-2	278	Background	AT5G59840	Symbols: | Ras-related small GTP-binding family	157
Dark-2	278	Background	AT5G59840	Symbols: | Ras-related small GTP-binding family	157
Dark-2	278	Background	AT5G59840	Symbols: | Ras-related small GTP-binding family	157
Dark-2	278	Background	AT5G59840	Symbols: | Ras-related small GTP-binding family	157
Dark-2	279	Background	AT1G18450	Symbols: ATARP4, ARP4 | actin-related protein 4	156
Dark-2	279	Background	AT1G18450	Symbols: ATARP4, ARP4 | actin-related protein 4	156
Dark-2	280	Background	AT3G05590	Symbols: RPL18 | ribosomal protein L18 | chr3:1	155
Dark-2	280	Background	AT3G05590	Symbols: RPL18 | ribosomal protein L18 | chr3:1	155
Dark-2	280	Background	AT3G05590	Symbols: RPL18 | ribosomal protein L18 | chr3:1	155
Dark-2	281	Background	AT2G17360	Symbols: | Ribosomal protein S4 (RPS4A) family	151
Dark-2	281	Background	AT2G17360	Symbols: | Ribosomal protein S4 (RPS4A) family	151
Dark-2	281	Background	AT2G17360	Symbols: | Ribosomal protein S4 (RPS4A) family	151
Dark-2	281	Background	AT2G17360	Symbols: | Ribosomal protein S4 (RPS4A) family	151
Dark-2	281	Background	AT2G17360	Symbols: | Ribosomal protein S4 (RPS4A) family	151
Dark-2	282	Background	AT4G38630	Symbols: RPN10, MCB1, ATMCB1, MBP1 | regula	150
Dark-2	282	Background	AT4G38630	Symbols: RPN10, MCB1, ATMCB1, MBP1 | regula	150
Dark-2	283	Background	AT2G41800	Symbols: | Protein of unknown function, DUF64	150
Dark-2	283	Background	AT2G41800	Symbols: | Protein of unknown function, DUF64	150
Dark-2	283	Background	AT2G41800	Symbols: | Protein of unknown function, DUF64	150
Dark-2	283	Background	AT2G41800	Symbols: | Protein of unknown function, DUF64	150
Dark-2	284	Background	AT5G43780	Symbols: APS4 | Pseudouridine synthase/archae	149
Dark-2	284	Background	AT5G43780	Symbols: APS4 | Pseudouridine synthase/archae	149
Dark-2	284	Background	AT5G43780	Symbols: APS4 | Pseudouridine synthase/archae	149
Dark-2	284	Background	AT5G43780	Symbols: APS4 | Pseudouridine synthase/archae	149
Dark-2	285	Background	AT5G24710	Symbols: | Transducin/WD40 repeat-like superf	148
Dark-2	285	Background	AT5G24710	Symbols: | Transducin/WD40 repeat-like superf	148
Dark-2	285	Background	AT5G24710	Symbols: | Transducin/WD40 repeat-like superf	148
Dark-2	286	Background	AT5G51570	Symbols: | SPFH/Band 7/PHB domain-containin	147
Dark-2	286	Background	AT5G51570	Symbols: | SPFH/Band 7/PHB domain-containin	147
Dark-2	286	Background	AT5G51570	Symbols: | SPFH/Band 7/PHB domain-containin	147
Dark-2	287	Background	AT5G05010	Symbols: | clathrin adaptor complexes medium	147
Dark-2	287	Background	AT5G05010	Symbols: | clathrin adaptor complexes medium	147

Dark-2	288	Background	AT4G18800	Symbols: ATHSGBP, ATRAB11B, ATRABA1D, RAB	147
Dark-2	288	Background	AT4G18800	Symbols: ATHSGBP, ATRAB11B, ATRABA1D, RAB	147
Dark-2	288	Background	AT4G18800	Symbols: ATHSGBP, ATRAB11B, ATRABA1D, RAB	147
Dark-2	288	Background	AT4G18800	Symbols: ATHSGBP, ATRAB11B, ATRABA1D, RAB	147
Dark-2	288	Background	AT4G18800	Symbols: ATHSGBP, ATRAB11B, ATRABA1D, RAB	147
Dark-2	289	Background	AT5G09900	Symbols: EMB2107, RPN5A, MSA | 26S proteaso	146
Dark-2	289	Background	AT5G09900	Symbols: EMB2107, RPN5A, MSA | 26S proteaso	146
Dark-2	289	Background	AT5G09900	Symbols: EMB2107, RPN5A, MSA | 26S proteaso	146
Dark-2	289	Background	AT5G09900	Symbols: EMB2107, RPN5A, MSA | 26S proteaso	146
Dark-2	289	Background	AT5G09900	Symbols: EMB2107, RPN5A, MSA | 26S proteaso	146
Dark-2	290	Background	AT5G50850	Symbols: MAB1 | Transketolase family protein |	146
Dark-2	290	Background	AT5G50850	Symbols: MAB1 | Transketolase family protein |	146
Dark-2	290	Background	AT5G50850	Symbols: MAB1 | Transketolase family protein |	146
Dark-2	291	Background	AT5G08610	Symbols: | P-loop containing nucleoside triphos	144
Dark-2	291	Background	AT5G08610	Symbols: | P-loop containing nucleoside triphos	144
Dark-2	291	Background	AT5G08610	Symbols: | P-loop containing nucleoside triphos	144
Dark-2	291	Background	AT5G08610	Symbols: | P-loop containing nucleoside triphos	144
Dark-2	292	Background	AT4G01100	Symbols: ADNT1 | adenine nucleotide transport	143
Dark-2	292	Background	AT4G01100	Symbols: ADNT1 | adenine nucleotide transport	143
Dark-2	292	Background	AT4G01100	Symbols: ADNT1 | adenine nucleotide transport	143
Dark-2	293	Background	AT4G11420	Symbols: EIF3A, ATEIF3A-1, EIF3A-1, ATTIF3A1, T	142
Dark-2	293	Background	AT4G11420	Symbols: EIF3A, ATEIF3A-1, EIF3A-1, ATTIF3A1, T	142
Dark-2	293	Background	AT4G11420	Symbols: EIF3A, ATEIF3A-1, EIF3A-1, ATTIF3A1, T	142
Dark-2	293	Background	AT4G11420	Symbols: EIF3A, ATEIF3A-1, EIF3A-1, ATTIF3A1, T	142
Dark-2	294	Background	AT1G26630	Symbols: FBR12, ATELF5A-2, ELF5A-2 | Eukaryot	142
Dark-2	294	Background	AT1G26630	Symbols: FBR12, ATELF5A-2, ELF5A-2 | Eukaryot	142
Dark-2	295	Background	AT1G23410	Symbols: | Ribosomal protein S27a / Ubiquitin f	140
Dark-2	295	Background	AT1G23410	Symbols: | Ribosomal protein S27a / Ubiquitin f	140
Dark-2	295	Background	AT1G23410	Symbols: | Ribosomal protein S27a / Ubiquitin f	140
Dark-2	295	Background	AT1G23410	Symbols: | Ribosomal protein S27a / Ubiquitin f	140
Dark-2	296	Background	AT2G01250	Symbols: | Ribosomal protein L30/L7 family pro	139
Dark-2	296	Background	AT2G01250	Symbols: | Ribosomal protein L30/L7 family pro	139
Dark-2	296	Background	AT2G01250	Symbols: | Ribosomal protein L30/L7 family pro	139
Dark-2	296	Background	AT2G01250	Symbols: | Ribosomal protein L30/L7 family pro	139
Dark-2	296	Background	AT2G01250	Symbols: | Ribosomal protein L30/L7 family pro	139
Dark-2	297	Background	AT5G37510	Symbols: EMB1467, CI76 | NADH-ubiquinone de	138
Dark-2	297	Background	AT5G37510	Symbols: EMB1467, CI76 | NADH-ubiquinone de	138
Dark-2	298	Background	AT1G06220	Symbols: MEE5, CLO, GFA1 | Ribosomal protein	138
Dark-2	298	Background	AT1G06220	Symbols: MEE5, CLO, GFA1 | Ribosomal protein	138
Dark-2	298	Background	AT1G06220	Symbols: MEE5, CLO, GFA1 | Ribosomal protein	138
Dark-2	298	Background	AT1G06220	Symbols: MEE5, CLO, GFA1 | Ribosomal protein	138
Dark-2	298	Background	AT1G06220	Symbols: MEE5, CLO, GFA1 | Ribosomal protein	138
Dark-2	299	Background	AT2G28000	Symbols: CPN60A, CH-CPN60A, SLP | chaperonir	137
Dark-2	299	Background	AT2G28000	Symbols: CPN60A, CH-CPN60A, SLP | chaperonir	137
Dark-2	299	Background	AT2G28000	Symbols: CPN60A, CH-CPN60A, SLP | chaperonir	137
Dark-2	300	Background	AT2G19490	Symbols: | recA DNA recombination family prot	136
Dark-2	300	Background	AT2G19490	Symbols: | recA DNA recombination family prot	136
Dark-2	300	Background	AT2G19490	Symbols: | recA DNA recombination family prot	136
Dark-2	301	Background	AT1G72370	Symbols: P40, AP40, RP40, RPSAA | 40s ribosom	135
Dark-2	301	Background	AT1G72370	Symbols: P40, AP40, RP40, RPSAA | 40s ribosom	135
Dark-2	301	Background	AT1G72370	Symbols: P40, AP40, RP40, RPSAA | 40s ribosom	135
Dark-2	302	Background	AT4G27585	Symbols: | SPFH/Band 7/PHB domain-containin	133
Dark-2	302	Background	AT4G27585	Symbols: | SPFH/Band 7/PHB domain-containin	133
Dark-2	302	Background	AT4G27585	Symbols: | SPFH/Band 7/PHB domain-containin	133
Dark-2	302	Background	AT4G27585	Symbols: | SPFH/Band 7/PHB domain-containin	133
Dark-2	303	Background	AT3G52580	Symbols: | Ribosomal protein S11 family protei	133
Dark-2	303	Background	AT3G52580	Symbols: | Ribosomal protein S11 family protei	133
Dark-2	303	Background	AT3G52580	Symbols: | Ribosomal protein S11 family protei	133

Dark-2	304	Background	AT1G04480	Symbols: | Ribosomal protein L14p/L23e family	132
Dark-2	304	Background	AT1G04480	Symbols: | Ribosomal protein L14p/L23e family	132
Dark-2	305	Background	AT2G18330	Symbols: | AAA-type ATPase family protein | ch	129
Dark-2	305	Background	AT2G18330	Symbols: | AAA-type ATPase family protein | ch	129
Dark-2	306	Background	AT1G50200	Symbols: ALATS, ACD | Alanyl-tRNA synthetase |	128
Dark-2	306	Background	AT1G50200	Symbols: ALATS, ACD | Alanyl-tRNA synthetase |	128
Dark-2	307	Background	AT5G04130	Symbols: GYRB2 | DNA GYRASE B2 | chr5:11220	127
Dark-2	307	Background	AT5G04130	Symbols: GYRB2 | DNA GYRASE B2 | chr5:11220	127
Dark-2	308	Background	AT3G13300	Symbols: VCS | Transducin/WD40 repeat-like su	127
Dark-2	308	Background	AT3G13300	Symbols: VCS | Transducin/WD40 repeat-like su	127
Dark-2	308	Background	AT3G13300	Symbols: VCS | Transducin/WD40 repeat-like su	127
Dark-2	308	Background	AT3G13300	Symbols: VCS | Transducin/WD40 repeat-like su	127
Dark-2	308	Background	AT3G13300	Symbols: VCS | Transducin/WD40 repeat-like su	127
Dark-2	308	Background	AT3G13300	Symbols: VCS | Transducin/WD40 repeat-like su	127
Dark-2	309	Background	AT5G22060	Symbols: ATJ2, J2 | DNAJ homologue 2 | chr5:73	127
Dark-2	309	Background	AT5G22060	Symbols: ATJ2, J2 | DNAJ homologue 2 | chr5:73	127
Dark-2	309	Background	AT5G22060	Symbols: ATJ2, J2 | DNAJ homologue 2 | chr5:73	127
Dark-2	309	Background	AT5G22060	Symbols: ATJ2, J2 | DNAJ homologue 2 | chr5:73	127
Dark-2	310	Background	AT5G16300	Symbols: | Vps51/Vps67 family (components of	126
Dark-2	310	Background	AT5G16300	Symbols: | Vps51/Vps67 family (components of	126
Dark-2	311	Table 1 Filtere	AT2G02100	Symbols: LCR69, PDF2.2 | low-molecular-weight	126
Dark-2	311	Table 1 Filtere	AT2G02100	Symbols: LCR69, PDF2.2 | low-molecular-weight	126
Dark-2	312	Background	AT4G19490	Symbols: ATVPS54, VPS54 | VPS54 | chr4:10616	125
Dark-2	312	Background	AT4G19490	Symbols: ATVPS54, VPS54 | VPS54 | chr4:10616	125
Dark-2	313	Background	AT3G02260	Symbols: BIG, DOC1, TIR3, UMB1, ASA1, LPR1, C	125
Dark-2	313	Background	AT3G02260	Symbols: BIG, DOC1, TIR3, UMB1, ASA1, LPR1, C	125
Dark-2	314	Background	AT2G47650	Symbols: UXS4 | UDP-xylose synthase 4 | chr2:1	125
Dark-2	314	Background	AT2G47650	Symbols: UXS4 | UDP-xylose synthase 4 | chr2:1	125
Dark-2	315	Background	AT5G25780	Symbols: EIF3B-2, EIF3B, ATEIF3B-2 | eukaryotic	121
Dark-2	315	Background	AT5G25780	Symbols: EIF3B-2, EIF3B, ATEIF3B-2 | eukaryotic	121
Dark-2	315	Background	AT5G25780	Symbols: EIF3B-2, EIF3B, ATEIF3B-2 | eukaryotic	121
Dark-2	316	Background	AT3G24830	Symbols: | Ribosomal protein L13 family protei	121
Dark-2	316	Background	AT3G24830	Symbols: | Ribosomal protein L13 family protei	121
Dark-2	317	Background	AT2G20800	Symbols: NDB4 | NAD(P)H dehydrogenase B4 |	121
Dark-2	317	Background	AT2G20800	Symbols: NDB4 | NAD(P)H dehydrogenase B4 |	121
Dark-2	318	Background	AT1G80070	Symbols: SUS2, EMB33, EMB177, EMB14 | Pre-r	120
Dark-2	318	Background	AT1G80070	Symbols: SUS2, EMB33, EMB177, EMB14 | Pre-r	120
Dark-2	319	Background	AT5G22770	Symbols: alpha-ADR | alpha-adaptin | chr5:7579	120
Dark-2	319	Background	AT5G22770	Symbols: alpha-ADR | alpha-adaptin | chr5:7579	120
Dark-2	320	Background	AT1G70320	Symbols: UPL2 | ubiquitin-protein ligase 2 | chr1	119
Dark-2	320	Background	AT1G70320	Symbols: UPL2 | ubiquitin-protein ligase 2 | chr1	119
Dark-2	321	Background	AT4G02030	Symbols: | Vps51/Vps67 family (components of	119
Dark-2	321	Background	AT4G02030	Symbols: | Vps51/Vps67 family (components of	119
Dark-2	322	Table 1 Filtere	AT2G36200	Symbols: | P-loop containing nucleoside triphos	118
Dark-2	322	Table 1 Filtere	AT2G36200	Symbols: | P-loop containing nucleoside triphos	118
Dark-2	322	Table 1 Filtere	AT2G36200	Symbols: | P-loop containing nucleoside triphos	118
Dark-2	323	Background	AT5G62190	Symbols: PRH75 | DEAD box RNA helicase (PRH7	118
Dark-2	323	Background	AT5G62190	Symbols: PRH75 | DEAD box RNA helicase (PRH7	118
Dark-2	323	Background	AT5G62190	Symbols: PRH75 | DEAD box RNA helicase (PRH7	118
Dark-2	324	Background	AT1G26830	Symbols: CUL3A, ATCUL3A, ATCUL3, CUL3 | culli	116
Dark-2	324	Background	AT1G26830	Symbols: CUL3A, ATCUL3A, ATCUL3, CUL3 | culli	116
Dark-2	325	Background	AT2G42740	Symbols: RPL16A | ribosomal protein large subu	116
Dark-2	325	Background	AT2G42740	Symbols: RPL16A | ribosomal protein large subu	116
Dark-2	325	Background	AT2G42740	Symbols: RPL16A | ribosomal protein large subu	116
Dark-2	326	Background	AT3G14990	Symbols: | Class I glutamine amidotransferase-l	115
Dark-2	326	Background	AT3G14990	Symbols: | Class I glutamine amidotransferase-l	115
Dark-2	326	Background	AT3G14990	Symbols: | Class I glutamine amidotransferase-l	115
Dark-2	327	Background	AT1G14320	Symbols: SAC52, RPL10, RPL10A | Ribosomal pro	115

Dark-2	327	Background	AT1G14320	Symbols: SAC52, RPL10, RPL10A | Ribosomal pro	115
Dark-2	328	Background	AT3G58660	Symbols: | Ribosomal protein L1p/L10e family |	114
Dark-2	328	Background	AT3G58660	Symbols: | Ribosomal protein L1p/L10e family |	114
Dark-2	329	Background	AT1G07705	Symbols: | NOT2 / NOT3 / NOT5 family | chr1:2	112
Dark-2	329	Background	AT1G07705	Symbols: | NOT2 / NOT3 / NOT5 family | chr1:2	112
Dark-2	330	Background	AT2G27530	Symbols: PGY1 | Ribosomal protein L1p/L10e fa	112
Dark-2	330	Background	AT2G27530	Symbols: PGY1 | Ribosomal protein L1p/L10e fa	112
Dark-2	331	Background	AT1G24510	Symbols: | TCP-1/cpn60 chaperonin family prot	110
Dark-2	331	Background	AT1G24510	Symbols: | TCP-1/cpn60 chaperonin family prot	110
Dark-2	332	Background	AT4G12420	Symbols: SKU5 | Cupredoxin superfamily proteir	110
Dark-2	332	Background	AT4G12420	Symbols: SKU5 | Cupredoxin superfamily proteir	110
Dark-2	332	Background	AT4G12420	Symbols: SKU5 | Cupredoxin superfamily proteir	110
Dark-2	332	Background	AT4G12420	Symbols: SKU5 | Cupredoxin superfamily proteir	110
Dark-2	333	Background	AT5G05780	Symbols: RPN8A, AE3, ATHMOV34 | RP non-ATP	107
Dark-2	333	Background	AT5G05780	Symbols: RPN8A, AE3, ATHMOV34 | RP non-ATP	107
Dark-2	333	Background	AT5G05780	Symbols: RPN8A, AE3, ATHMOV34 | RP non-ATP	107
Dark-2	333	Background	AT5G05780	Symbols: RPN8A, AE3, ATHMOV34 | RP non-ATP	107
Dark-2	334	Background	AT5G52470	Symbols: FIB1, FBR1, ATFIB1, ATFBR1, SKIP7 | fib	107
Dark-2	334	Background	AT5G52470	Symbols: FIB1, FBR1, ATFIB1, ATFBR1, SKIP7 | fib	107
Dark-2	334	Background	AT5G52470	Symbols: FIB1, FBR1, ATFIB1, ATFBR1, SKIP7 | fib	107
Dark-2	335	Background	AT1G71270	Symbols: POK, TTD8, ATVPS52 | Vps52 / Sac2 fa	107
Dark-2	335	Background	AT1G71270	Symbols: POK, TTD8, ATVPS52 | Vps52 / Sac2 fa	107
Dark-2	335	Background	AT1G71270	Symbols: POK, TTD8, ATVPS52 | Vps52 / Sac2 fa	107
Dark-2	336	Background	AT3G05560	Symbols: | Ribosomal L22e protein family | chr3	106
Dark-2	336	Background	AT3G05560	Symbols: | Ribosomal L22e protein family | chr3	106
Dark-2	337	Background	AT5G26360	Symbols: | TCP-1/cpn60 chaperonin family prot	106
Dark-2	337	Background	AT5G26360	Symbols: | TCP-1/cpn60 chaperonin family prot	106
Dark-2	338	Background	AT1G48900	Symbols: | Signal recognition particle, SRP54 su	103
Dark-2	338	Background	AT1G48900	Symbols: | Signal recognition particle, SRP54 su	103
Dark-2	339	Background	AT4G27640	Symbols: | ARM repeat superfamily protein | ch	101
Dark-2	339	Background	AT4G27640	Symbols: | ARM repeat superfamily protein | ch	101
Dark-2	340	Background	AT2G42520	Symbols: | P-loop containing nucleoside triphos	99
Dark-2	340	Background	AT2G42520	Symbols: | P-loop containing nucleoside triphos	99
Dark-2	341	Background	AT1G24300	Symbols: | GYF domain-containing protein | chr	96
Dark-2	341	Background	AT1G24300	Symbols: | GYF domain-containing protein | chr	96
Dark-2	341	Background	AT1G24300	Symbols: | GYF domain-containing protein | chr	96
Dark-2	342	Background	AT5G60730	Symbols: | Anion-transporting ATPase | chr5:24	96
Dark-2	342	Background	AT5G60730	Symbols: | Anion-transporting ATPase | chr5:24	96
Dark-2	343	Background	AT3G42170	Symbols: | BED zinc finger ;hAT family dimerisat	95
Dark-2	343	Background	AT3G42170	Symbols: | BED zinc finger ;hAT family dimerisat	95
Dark-2	344	Background	AT1G50500	Symbols: HIT1, ATVPS53, VPS53 | Membrane tra	94
Dark-2	344	Background	AT1G50500	Symbols: HIT1, ATVPS53, VPS53 | Membrane tra	94
Dark-2	344	Background	AT1G50500	Symbols: HIT1, ATVPS53, VPS53 | Membrane tra	94
Dark-2	345	Background	AT1G01470	Symbols: LEA14, LSR3 | Late embryogenesis abu	94
Dark-2	345	Background	AT1G01470	Symbols: LEA14, LSR3 | Late embryogenesis abu	94
Dark-2	346	Background	AT1G79340	Symbols: AtMC4, MC4 | metacaspase 4 | chr1:2	93
Dark-2	346	Background	AT1G79340	Symbols: AtMC4, MC4 | metacaspase 4 | chr1:2	93
Dark-2	347	Background	AT2G05830	Symbols: | NagB/RpiA/CoA transferase-like supe	93
Dark-2	347	Background	AT2G05830	Symbols: | NagB/RpiA/CoA transferase-like supe	93
Dark-2	348	Background	ATCG00480	Symbols: ATPB, PB | ATP synthase subunit beta	92
Dark-2	348	Background	ATCG00480	Symbols: ATPB, PB | ATP synthase subunit beta	92
Dark-2	349	Background	AT1G51380	Symbols: | DEA(D/H)-box RNA helicase family p	92
Dark-2	349	Background	AT1G51380	Symbols: | DEA(D/H)-box RNA helicase family p	92
Dark-2	350	Background	AT5G45620	Symbols: | Proteasome component (PCI) domai	92
Dark-2	350	Background	AT5G45620	Symbols: | Proteasome component (PCI) domai	92
Dark-2	351	Background	AT3G56130	Symbols: | biotin/lipoyl attachment domain-cor	90
Dark-2	351	Background	AT3G56130	Symbols: | biotin/lipoyl attachment domain-cor	90
Dark-2	352	Background	AT3G61650	Symbols: TUBG1 | gamma-tubulin | chr3:22812(	90

Dark-2	352	Background	AT3G61650	Symbols: TUBG1 | gamma-tubulin | chr3:22812(	90
Dark-2	352	Background	AT3G61650	Symbols: TUBG1 | gamma-tubulin | chr3:22812(	90
Dark-2	353	Background	AT1G27400	Symbols: | Ribosomal protein L22p/L17e family	89
Dark-2	353	Background	AT1G27400	Symbols: | Ribosomal protein L22p/L17e family	89
Dark-2	354	Background	AT4G31700	Symbols: RPS6, RPS6A | ribosomal protein S6 | c	89
Dark-2	354	Background	AT4G31700	Symbols: RPS6, RPS6A | ribosomal protein S6 | c	89
Dark-2	355	Background	AT3G06400	Symbols: CHR11 | chromatin-remodeling protei	86
Dark-2	355	Background	AT3G06400	Symbols: CHR11 | chromatin-remodeling protei	86
Dark-2	356	Background	AT3G19820	Symbols: DWF1, DIM, EVE1, DIM1, CBB1 | cell el	85
Dark-2	356	Background	AT3G19820	Symbols: DWF1, DIM, EVE1, DIM1, CBB1 | cell el	85
Dark-2	357	Background	AT5G12470	Symbols: | Protein of unknown function (DUF34	84
Dark-2	357	Background	AT5G12470	Symbols: | Protein of unknown function (DUF34	84
Dark-2	357	Background	AT5G12470	Symbols: | Protein of unknown function (DUF34	84
Dark-2	358	Background	AT4G19006	Symbols: | Proteasome component (PCI) domai	84
Dark-2	358	Background	AT4G19006	Symbols: | Proteasome component (PCI) domai	84
Dark-2	359	Background	AT4G25630	Symbols: FIB2, ATFIB2 | fibrillarin 2 | chr4:13074	84
Dark-2	359	Background	AT4G25630	Symbols: FIB2, ATFIB2 | fibrillarin 2 | chr4:13074	84
Dark-2	359	Background	AT4G25630	Symbols: FIB2, ATFIB2 | fibrillarin 2 | chr4:13074	84
Dark-2	360	Background	AT3G18190	Symbols: | TCP-1/cpn60 chaperonin family prot	82
Dark-2	360	Background	AT3G18190	Symbols: | TCP-1/cpn60 chaperonin family prot	82
Dark-2	360	Background	AT3G18190	Symbols: | TCP-1/cpn60 chaperonin family prot	82
Dark-2	361	Background	AT2G40510	Symbols: | Ribosomal protein S26e family prote	81
Dark-2	361	Background	AT2G40510	Symbols: | Ribosomal protein S26e family prote	81
Dark-2	362	Background	AT5G27770	Symbols: | Ribosomal L22e protein family | chr5	81
Dark-2	362	Background	AT5G27770	Symbols: | Ribosomal L22e protein family | chr5	81
Dark-2	363	Background	AT1G07890	Symbols: APX1, MEE6, CS1, ATAPX1, ATAPX01 |	81
Dark-2	363	Background	AT1G07890	Symbols: APX1, MEE6, CS1, ATAPX1, ATAPX01 |	81
Dark-2	364	Background	AT1G71380	Symbols: ATGH9B3, ATCEL3, CEL3 | cellulase 3 |	80
Dark-2	364	Background	AT1G71380	Symbols: ATGH9B3, ATCEL3, CEL3 | cellulase 3 |	80
Dark-2	364	Background	AT1G71380	Symbols: ATGH9B3, ATCEL3, CEL3 | cellulase 3 |	80
Dark-2	364	Background	AT1G71380	Symbols: ATGH9B3, ATCEL3, CEL3 | cellulase 3 |	80
Dark-2	365	Background	AT3G60770	Symbols: | Ribosomal protein S13/S15 | chr3:22	80
Dark-2	365	Background	AT3G60770	Symbols: | Ribosomal protein S13/S15 | chr3:22	80
Dark-2	365	Background	AT3G60770	Symbols: | Ribosomal protein S13/S15 | chr3:22	80
Dark-2	366	Background	AT2G43460	Symbols: | Ribosomal L38e protein family | chr2	76
Dark-2	366	Background	AT2G43460	Symbols: | Ribosomal L38e protein family | chr2	76
Dark-2	367	Background	AT3G11400	Symbols: EIF3G1, ATEIF3G1 | eukaryotic translat	75
Dark-2	367	Background	AT3G11400	Symbols: EIF3G1, ATEIF3G1 | eukaryotic translat	75
Dark-2	368	Background	AT1G80670	Symbols: | Transducin/WD40 repeat-like superf	75
Dark-2	368	Background	AT1G80670	Symbols: | Transducin/WD40 repeat-like superf	75
Dark-2	369	Background	AT5G42080	Symbols: ADL1, ADL1A, AG68, DRP1A, RSW9, DL	74
Dark-2	369	Background	AT5G42080	Symbols: ADL1, ADL1A, AG68, DRP1A, RSW9, DL	74
Dark-2	370	Background	AT2G32730	Symbols: | 26S proteasome regulatory complex	74
Dark-2	370	Background	AT2G32730	Symbols: | 26S proteasome regulatory complex	74
Dark-2	370	Background	AT2G32730	Symbols: | 26S proteasome regulatory complex	74
Dark-2	371	Background	AT1G50920	Symbols: | Nucleolar GTP-binding protein | chr1	73
Dark-2	371	Background	AT1G50920	Symbols: | Nucleolar GTP-binding protein | chr1	73
Dark-2	372	Background	AT3G01540	Symbols: DRH1, ATDRH1 | DEAD box RNA helica	73
Dark-2	372	Background	AT3G01540	Symbols: DRH1, ATDRH1 | DEAD box RNA helica	73
Dark-2	373	Background	AT2G37190	Symbols: | Ribosomal protein L11 family protei	73
Dark-2	373	Background	AT2G37190	Symbols: | Ribosomal protein L11 family protei	73
Dark-2	374	Background	AT1G12900	Symbols: GAPA-2 | glyceraldehyde 3-phosphate	72
Dark-2	374	Background	AT1G12900	Symbols: GAPA-2 | glyceraldehyde 3-phosphate	72
Dark-2	375	Background	AT3G46830	Symbols: ATRAB11A, ATRABA2C, ATRAB-A2C, RA	72
Dark-2	375	Background	AT3G46830	Symbols: ATRAB11A, ATRABA2C, ATRAB-A2C, RA	72
Dark-2	375	Background	AT3G46830	Symbols: ATRAB11A, ATRABA2C, ATRAB-A2C, RA	72
Dark-2	376	Background	AT1G61990	Symbols: | Mitochondrial transcription termina	72
Dark-2	376	Background	AT1G61990	Symbols: | Mitochondrial transcription termina	72

Dark-2	377	Background	AT5G53480	Symbols: | ARM repeat superfamily protein | ch	72
Dark-2	377	Background	AT5G53480	Symbols: | ARM repeat superfamily protein | ch	72
Dark-2	378	Background	AT5G18230	Symbols: | transcription regulator NOT2/NOT3/	69
Dark-2	378	Background	AT5G18230	Symbols: | transcription regulator NOT2/NOT3/	69
Dark-2	379	Background	AT5G15200	Symbols: | Ribosomal protein S4 | chr5:493512	68
Dark-2	379	Background	AT5G15200	Symbols: | Ribosomal protein S4 | chr5:493512	68
Dark-2	379	Background	AT5G15200	Symbols: | Ribosomal protein S4 | chr5:493512	68
Dark-2	380	Background	AT3G60860	Symbols: | SEC7-like guanine nucleotide exchan	68
Dark-2	380	Background	AT3G60860	Symbols: | SEC7-like guanine nucleotide exchan	68
Dark-2	381	Background	AT1G04810	Symbols: | 26S proteasome regulatory complex	68
Dark-2	381	Background	AT1G04810	Symbols: | 26S proteasome regulatory complex	68
Dark-2	381	Background	AT1G04810	Symbols: | 26S proteasome regulatory complex	68
Dark-2	382	Background	AT2G46280	Symbols: TRIP-1, TIF3I1 | TGF-beta receptor inte	67
Dark-2	382	Background	AT2G46280	Symbols: TRIP-1, TIF3I1 | TGF-beta receptor inte	67
Dark-2	383	Background	AT5G16390	Symbols: CAC1, CAC1A, BCCP, BCCP1 | chloropla	67
Dark-2	383	Background	AT5G16390	Symbols: CAC1, CAC1A, BCCP, BCCP1 | chloropla	67
Dark-2	383	Background	AT5G16390	Symbols: CAC1, CAC1A, BCCP, BCCP1 | chloropla	67
Dark-2	384	Background	AT2G03510	Symbols: | SPFH/Band 7/PHB domain-containin	66
Dark-2	384	Background	AT2G03510	Symbols: | SPFH/Band 7/PHB domain-containin	66
Dark-2	384	Background	AT2G03510	Symbols: | SPFH/Band 7/PHB domain-containin	66
Dark-2	384	Background	AT2G03510	Symbols: | SPFH/Band 7/PHB domain-containin	66
Dark-2	385	Background	AT3G21540	Symbols: | transducin family protein / WD-40 re	66
Dark-2	385	Background	AT3G21540	Symbols: | transducin family protein / WD-40 re	66
Dark-2	386	Background	AT2G40730	Symbols: | Protein kinase family protein with AI	64
Dark-2	386	Background	AT2G40730	Symbols: | Protein kinase family protein with AI	64
Dark-2	387	Background	AT2G47640	Symbols: | Small nuclear ribonucleoprotein fam	62
Dark-2	387	Background	AT2G47640	Symbols: | Small nuclear ribonucleoprotein fam	62
Dark-2	388	Table 1 Filtere	AT1G55150	Symbols: | DEA(D/H)-box RNA helicase family p	61
Dark-2	388	Table 1 Filtere	AT1G55150	Symbols: | DEA(D/H)-box RNA helicase family p	61
Dark-2	389	Background	AT3G15000	Symbols: | cobalt ion binding | chr3:5050321-5	60
Dark-2	389	Background	AT3G15000	Symbols: | cobalt ion binding | chr3:5050321-5	60
Dark-2	390	Background	AT5G25754	Symbols: | RNA polymerase I-associated factor	60
Dark-2	390	Background	AT5G25754	Symbols: | RNA polymerase I-associated factor	60
Dark-2	391	Background	AT5G08620	Symbols: STRS2, ATRH25 | DEA(D/H)-box RNA h	58
Dark-2	391	Background	AT5G08620	Symbols: STRS2, ATRH25 | DEA(D/H)-box RNA h	58
Dark-2	392	Background	AT5G60790	Symbols: ATGCN1, GCN1 | ABC transporter fami	58
Dark-2	392	Background	AT5G60790	Symbols: ATGCN1, GCN1 | ABC transporter fami	58
Dark-2	393	Background	AT4G15000	Symbols: | Ribosomal L27e protein family | chr4	54
Dark-2	393	Background	AT4G15000	Symbols: | Ribosomal L27e protein family | chr4	54
Dark-2	394	Background	AT1G09590	Symbols: | Translation protein SH3-like family p	52
Dark-2	394	Background	AT1G09590	Symbols: | Translation protein SH3-like family p	52
Dark-2	395	Background	AT2G21580	Symbols: | Ribosomal protein S25 family protei	52
Dark-2	395	Background	AT2G21580	Symbols: | Ribosomal protein S25 family protei	52
Dark-2	396	Background	AT5G16130	Symbols: | Ribosomal protein S7e family protei	48
Dark-2	396	Background	AT5G16130	Symbols: | Ribosomal protein S7e family protei	48
Dark-2	397	Background	AT5G42220	Symbols: | Ubiquitin-like superfamily protein |	47
Dark-2	398	Background	AT4G39200	Symbols: | Ribosomal protein S25 family protei	41
Dark-2	398	Background	AT4G39200	Symbols: | Ribosomal protein S25 family protei	41
Dark-2	399	Background	AT2G34357	Symbols: | ARM repeat superfamily protein | ch	33
Dark-2	399	Background	AT2G34357	Symbols: | ARM repeat superfamily protein | ch	33
Light-1	400	Background	prot_acc	prot_desc	prot_score
Light-1	401	Background	AT5G44340	Symbols: TUB4 | tubulin beta chain 4 | chr5:178	1928
Light-1	401	Background	AT5G44340	Symbols: TUB4 | tubulin beta chain 4 | chr5:178	1928
Light-1	401	Background	AT5G44340	Symbols: TUB4 | tubulin beta chain 4 | chr5:178	1928
Light-1	401	Background	AT5G44340	Symbols: TUB4 | tubulin beta chain 4 | chr5:178	1928
Light-1	401	Background	AT5G44340	Symbols: TUB4 | tubulin beta chain 4 | chr5:178	1928
Light-1	401	Background	AT5G44340	Symbols: TUB4 | tubulin beta chain 4 | chr5:178	1928
Light-1	401	Background	AT5G44340	Symbols: TUB4 | tubulin beta chain 4 | chr5:178	1928

Light-1	401	Background	AT5G44340	Symbols: TUB4 | tubulin beta chain 4 | chr5:178	1928
Light-1	401	Background	AT5G44340	Symbols: TUB4 | tubulin beta chain 4 | chr5:178	1928
Light-1	401	Background	AT5G44340	Symbols: TUB4 | tubulin beta chain 4 | chr5:178	1928
Light-1	401	Background	AT5G44340	Symbols: TUB4 | tubulin beta chain 4 | chr5:178	1928
Light-1	401	Background	AT5G44340	Symbols: TUB4 | tubulin beta chain 4 | chr5:178	1928
Light-1	401	Background	AT5G44340	Symbols: TUB4 | tubulin beta chain 4 | chr5:178	1928
Light-1	401	Background	AT5G44340	Symbols: TUB4 | tubulin beta chain 4 | chr5:178	1928
Light-1	401	Background	AT5G44340	Symbols: TUB4 | tubulin beta chain 4 | chr5:178	1928
Light-1	401	Background	AT5G44340	Symbols: TUB4 | tubulin beta chain 4 | chr5:178	1928
Light-1	401	Background	AT5G44340	Symbols: TUB4 | tubulin beta chain 4 | chr5:178	1928
Light-1	401	Background	AT5G44340	Symbols: TUB4 | tubulin beta chain 4 | chr5:178	1928
Light-1	401	Background	AT5G44340	Symbols: TUB4 | tubulin beta chain 4 | chr5:178	1928
Light-1	401	Background	AT5G44340	Symbols: TUB4 | tubulin beta chain 4 | chr5:178	1928
Light-1	401	Background	AT5G44340	Symbols: TUB4 | tubulin beta chain 4 | chr5:178	1928
Light-1	401	Background	AT5G44340	Symbols: TUB4 | tubulin beta chain 4 | chr5:178	1928
Light-1	401	Background	AT5G44340	Symbols: TUB4 | tubulin beta chain 4 | chr5:178	1928
Light-1	401	Background	AT5G44340	Symbols: TUB4 | tubulin beta chain 4 | chr5:178	1928
Light-1	401	Background	AT5G44340	Symbols: TUB4 | tubulin beta chain 4 | chr5:178	1928
Light-1	402	Background	AT4G20890	Symbols: TUB9 | tubulin beta-9 chain | chr4:111	1854
Light-1	402	Background	AT4G20890	Symbols: TUB9 | tubulin beta-9 chain | chr4:111	1854
Light-1	402	Background	AT4G20890	Symbols: TUB9 | tubulin beta-9 chain | chr4:111	1854
Light-1	402	Background	AT4G20890	Symbols: TUB9 | tubulin beta-9 chain | chr4:111	1854
Light-1	402	Background	AT4G20890	Symbols: TUB9 | tubulin beta-9 chain | chr4:111	1854
Light-1	402	Background	AT4G20890	Symbols: TUB9 | tubulin beta-9 chain | chr4:111	1854
Light-1	402	Background	AT4G20890	Symbols: TUB9 | tubulin beta-9 chain | chr4:111	1854
Light-1	402	Background	AT4G20890	Symbols: TUB9 | tubulin beta-9 chain | chr4:111	1854
Light-1	402	Background	AT4G20890	Symbols: TUB9 | tubulin beta-9 chain | chr4:111	1854
Light-1	402	Background	AT4G20890	Symbols: TUB9 | tubulin beta-9 chain | chr4:111	1854
Light-1	402	Background	AT4G20890	Symbols: TUB9 | tubulin beta-9 chain | chr4:111	1854
Light-1	402	Background	AT4G20890	Symbols: TUB9 | tubulin beta-9 chain | chr4:111	1854
Light-1	402	Background	AT4G20890	Symbols: TUB9 | tubulin beta-9 chain | chr4:111	1854
Light-1	402	Background	AT4G20890	Symbols: TUB9 | tubulin beta-9 chain | chr4:111	1854
Light-1	402	Background	AT4G20890	Symbols: TUB9 | tubulin beta-9 chain | chr4:111	1854
Light-1	402	Background	AT4G20890	Symbols: TUB9 | tubulin beta-9 chain | chr4:111	1854
Light-1	402	Background	AT4G20890	Symbols: TUB9 | tubulin beta-9 chain | chr4:111	1854
Light-1	402	Background	AT4G20890	Symbols: TUB9 | tubulin beta-9 chain | chr4:111	1854
Light-1	402	Background	AT4G20890	Symbols: TUB9 | tubulin beta-9 chain | chr4:111	1854
Light-1	402	Background	AT4G20890	Symbols: TUB9 | tubulin beta-9 chain | chr4:111	1854
Light-1	402	Background	AT4G20890	Symbols: TUB9 | tubulin beta-9 chain | chr4:111	1854
Light-1	402	Background	AT4G20890	Symbols: TUB9 | tubulin beta-9 chain | chr4:111	1854
Light-1	402	Background	AT4G20890	Symbols: TUB9 | tubulin beta-9 chain | chr4:111	1854
Light-1	403	Background	AT5G62690	Symbols: TUB2 | tubulin beta chain 2 | chr5:251	1840
Light-1	403	Background	AT5G62690	Symbols: TUB2 | tubulin beta chain 2 | chr5:251	1840
Light-1	403	Background	AT5G62690	Symbols: TUB2 | tubulin beta chain 2 | chr5:251	1840
Light-1	403	Background	AT5G62690	Symbols: TUB2 | tubulin beta chain 2 | chr5:251	1840
Light-1	403	Background	AT5G62690	Symbols: TUB2 | tubulin beta chain 2 | chr5:251	1840
Light-1	403	Background	AT5G62690	Symbols: TUB2 | tubulin beta chain 2 | chr5:251	1840
Light-1	403	Background	AT5G62690	Symbols: TUB2 | tubulin beta chain 2 | chr5:251	1840
Light-1	403	Background	AT5G62690	Symbols: TUB2 | tubulin beta chain 2 | chr5:251	1840
Light-1	403	Background	AT5G62690	Symbols: TUB2 | tubulin beta chain 2 | chr5:251	1840
Light-1	403	Background	AT5G62690	Symbols: TUB2 | tubulin beta chain 2 | chr5:251	1840
Light-1	403	Background	AT5G62690	Symbols: TUB2 | tubulin beta chain 2 | chr5:251	1840
Light-1	403	Background	AT5G62690	Symbols: TUB2 | tubulin beta chain 2 | chr5:251	1840
Light-1	403	Background	AT5G62690	Symbols: TUB2 | tubulin beta chain 2 | chr5:251	1840
Light-1	403	Background	AT5G62690	Symbols: TUB2 | tubulin beta chain 2 | chr5:251	1840
Light-1	403	Background	AT5G62690	Symbols: TUB2 | tubulin beta chain 2 | chr5:251	1840
Light-1	403	Background	AT5G62690	Symbols: TUB2 | tubulin beta chain 2 | chr5:251	1840
Light-1	403	Background	AT5G62690	Symbols: TUB2 | tubulin beta chain 2 | chr5:251	1840

Light-1	403	Background	AT5G62690	Symbols: TUB2 | tubulin beta chain 2 | chr5:251	1840
Light-1	403	Background	AT5G62690	Symbols: TUB2 | tubulin beta chain 2 | chr5:251	1840
Light-1	403	Background	AT5G62690	Symbols: TUB2 | tubulin beta chain 2 | chr5:251	1840
Light-1	403	Background	AT5G62690	Symbols: TUB2 | tubulin beta chain 2 | chr5:251	1840
Light-1	403	Background	AT5G62690	Symbols: TUB2 | tubulin beta chain 2 | chr5:251	1840
Light-1	403	Background	AT5G62690	Symbols: TUB2 | tubulin beta chain 2 | chr5:251	1840
Light-1	403	Background	AT5G62690	Symbols: TUB2 | tubulin beta chain 2 | chr5:251	1840
Light-1	403	Background	AT5G62690	Symbols: TUB2 | tubulin beta chain 2 | chr5:251	1840
Light-1	404	Background	AT2G29550	Symbols: TUB7 | tubulin beta-7 chain | chr2:126	1784
Light-1	404	Background	AT2G29550	Symbols: TUB7 | tubulin beta-7 chain | chr2:126	1784
Light-1	404	Background	AT2G29550	Symbols: TUB7 | tubulin beta-7 chain | chr2:126	1784
Light-1	404	Background	AT2G29550	Symbols: TUB7 | tubulin beta-7 chain | chr2:126	1784
Light-1	404	Background	AT2G29550	Symbols: TUB7 | tubulin beta-7 chain | chr2:126	1784
Light-1	404	Background	AT2G29550	Symbols: TUB7 | tubulin beta-7 chain | chr2:126	1784
Light-1	404	Background	AT2G29550	Symbols: TUB7 | tubulin beta-7 chain | chr2:126	1784
Light-1	404	Background	AT2G29550	Symbols: TUB7 | tubulin beta-7 chain | chr2:126	1784
Light-1	404	Background	AT2G29550	Symbols: TUB7 | tubulin beta-7 chain | chr2:126	1784
Light-1	404	Background	AT2G29550	Symbols: TUB7 | tubulin beta-7 chain | chr2:126	1784
Light-1	404	Background	AT2G29550	Symbols: TUB7 | tubulin beta-7 chain | chr2:126	1784
Light-1	404	Background	AT2G29550	Symbols: TUB7 | tubulin beta-7 chain | chr2:126	1784
Light-1	404	Background	AT2G29550	Symbols: TUB7 | tubulin beta-7 chain | chr2:126	1784
Light-1	404	Background	AT2G29550	Symbols: TUB7 | tubulin beta-7 chain | chr2:126	1784
Light-1	404	Background	AT2G29550	Symbols: TUB7 | tubulin beta-7 chain | chr2:126	1784
Light-1	404	Background	AT2G29550	Symbols: TUB7 | tubulin beta-7 chain | chr2:126	1784
Light-1	404	Background	AT2G29550	Symbols: TUB7 | tubulin beta-7 chain | chr2:126	1784
Light-1	404	Background	AT2G29550	Symbols: TUB7 | tubulin beta-7 chain | chr2:126	1784
Light-1	404	Background	AT2G29550	Symbols: TUB7 | tubulin beta-7 chain | chr2:126	1784
Light-1	404	Background	AT2G29550	Symbols: TUB7 | tubulin beta-7 chain | chr2:126	1784
Light-1	404	Background	AT2G29550	Symbols: TUB7 | tubulin beta-7 chain | chr2:126	1784
Light-1	404	Background	AT2G29550	Symbols: TUB7 | tubulin beta-7 chain | chr2:126	1784
Light-1	405	Background	AT5G23860	Symbols: TUB8 | tubulin beta 8 | chr5:8042962-	1695
Light-1	405	Background	AT5G23860	Symbols: TUB8 | tubulin beta 8 | chr5:8042962-	1695
Light-1	405	Background	AT5G23860	Symbols: TUB8 | tubulin beta 8 | chr5:8042962-	1695
Light-1	405	Background	AT5G23860	Symbols: TUB8 | tubulin beta 8 | chr5:8042962-	1695
Light-1	405	Background	AT5G23860	Symbols: TUB8 | tubulin beta 8 | chr5:8042962-	1695
Light-1	405	Background	AT5G23860	Symbols: TUB8 | tubulin beta 8 | chr5:8042962-	1695
Light-1	405	Background	AT5G23860	Symbols: TUB8 | tubulin beta 8 | chr5:8042962-	1695
Light-1	405	Background	AT5G23860	Symbols: TUB8 | tubulin beta 8 | chr5:8042962-	1695
Light-1	405	Background	AT5G23860	Symbols: TUB8 | tubulin beta 8 | chr5:8042962-	1695
Light-1	405	Background	AT5G23860	Symbols: TUB8 | tubulin beta 8 | chr5:8042962-	1695
Light-1	405	Background	AT5G23860	Symbols: TUB8 | tubulin beta 8 | chr5:8042962-	1695
Light-1	405	Background	AT5G23860	Symbols: TUB8 | tubulin beta 8 | chr5:8042962-	1695
Light-1	405	Background	AT5G23860	Symbols: TUB8 | tubulin beta 8 | chr5:8042962-	1695
Light-1	405	Background	AT5G23860	Symbols: TUB8 | tubulin beta 8 | chr5:8042962-	1695
Light-1	405	Background	AT5G23860	Symbols: TUB8 | tubulin beta 8 | chr5:8042962-	1695
Light-1	405	Background	AT5G23860	Symbols: TUB8 | tubulin beta 8 | chr5:8042962-	1695
Light-1	405	Background	AT5G23860	Symbols: TUB8 | tubulin beta 8 | chr5:8042962-	1695
Light-1	405	Background	AT5G23860	Symbols: TUB8 | tubulin beta 8 | chr5:8042962-	1695
Light-1	405	Background	AT5G23860	Symbols: TUB8 | tubulin beta 8 | chr5:8042962-	1695
Light-1	405	Background	AT5G23860	Symbols: TUB8 | tubulin beta 8 | chr5:8042962-	1695
Light-1	405	Background	AT5G23860	Symbols: TUB8 | tubulin beta 8 | chr5:8042962-	1695
Light-1	405	Background	AT5G23860	Symbols: TUB8 | tubulin beta 8 | chr5:8042962-	1695
Light-1	405	Background	AT5G23860	Symbols: TUB8 | tubulin beta 8 | chr5:8042962-	1695
Light-1	406	Background	AT4G14960	Symbols: TUA6 | Tubulin/FtsZ family protein | cl	1625
Light-1	406	Background	AT4G14960	Symbols: TUA6 | Tubulin/FtsZ family protein | cl	1625
Light-1	406	Background	AT4G14960	Symbols: TUA6 | Tubulin/FtsZ family protein | cl	1625
Light-1	406	Background	AT4G14960	Symbols: TUA6 | Tubulin/FtsZ family protein | cl	1625

Light-1	406	Background	AT4G14960	Symbols: TUA6 | Tubulin/FtsZ family protein | cl	1625
Light-1	406	Background	AT4G14960	Symbols: TUA6 | Tubulin/FtsZ family protein | cl	1625
Light-1	406	Background	AT4G14960	Symbols: TUA6 | Tubulin/FtsZ family protein | cl	1625
Light-1	406	Background	AT4G14960	Symbols: TUA6 | Tubulin/FtsZ family protein | cl	1625
Light-1	406	Background	AT4G14960	Symbols: TUA6 | Tubulin/FtsZ family protein | cl	1625
Light-1	406	Background	AT4G14960	Symbols: TUA6 | Tubulin/FtsZ family protein | cl	1625
Light-1	406	Background	AT4G14960	Symbols: TUA6 | Tubulin/FtsZ family protein | cl	1625
Light-1	406	Background	AT4G14960	Symbols: TUA6 | Tubulin/FtsZ family protein | cl	1625
Light-1	406	Background	AT4G14960	Symbols: TUA6 | Tubulin/FtsZ family protein | cl	1625
Light-1	406	Background	AT4G14960	Symbols: TUA6 | Tubulin/FtsZ family protein | cl	1625
Light-1	406	Background	AT4G14960	Symbols: TUA6 | Tubulin/FtsZ family protein | cl	1625
Light-1	406	Background	AT4G14960	Symbols: TUA6 | Tubulin/FtsZ family protein | cl	1625
Light-1	406	Background	AT4G14960	Symbols: TUA6 | Tubulin/FtsZ family protein | cl	1625
Light-1	406	Background	AT4G14960	Symbols: TUA6 | Tubulin/FtsZ family protein | cl	1625
Light-1	406	Background	AT4G14960	Symbols: TUA6 | Tubulin/FtsZ family protein | cl	1625
Light-1	406	Background	AT4G14960	Symbols: TUA6 | Tubulin/FtsZ family protein | cl	1625
Light-1	406	Background	AT4G14960	Symbols: TUA6 | Tubulin/FtsZ family protein | cl	1625
Light-1	407	Background	AT1G04820	Symbols: TUA4, TOR2 | tubulin alpha-4 chain | c	1620
Light-1	407	Background	AT1G04820	Symbols: TUA4, TOR2 | tubulin alpha-4 chain | c	1620
Light-1	407	Background	AT1G04820	Symbols: TUA4, TOR2 | tubulin alpha-4 chain | c	1620
Light-1	407	Background	AT1G04820	Symbols: TUA4, TOR2 | tubulin alpha-4 chain | c	1620
Light-1	407	Background	AT1G04820	Symbols: TUA4, TOR2 | tubulin alpha-4 chain | c	1620
Light-1	407	Background	AT1G04820	Symbols: TUA4, TOR2 | tubulin alpha-4 chain | c	1620
Light-1	407	Background	AT1G04820	Symbols: TUA4, TOR2 | tubulin alpha-4 chain | c	1620
Light-1	407	Background	AT1G04820	Symbols: TUA4, TOR2 | tubulin alpha-4 chain | c	1620
Light-1	407	Background	AT1G04820	Symbols: TUA4, TOR2 | tubulin alpha-4 chain | c	1620
Light-1	407	Background	AT1G04820	Symbols: TUA4, TOR2 | tubulin alpha-4 chain | c	1620
Light-1	407	Background	AT1G04820	Symbols: TUA4, TOR2 | tubulin alpha-4 chain | c	1620
Light-1	407	Background	AT1G04820	Symbols: TUA4, TOR2 | tubulin alpha-4 chain | c	1620
Light-1	407	Background	AT1G04820	Symbols: TUA4, TOR2 | tubulin alpha-4 chain | c	1620
Light-1	407	Background	AT1G04820	Symbols: TUA4, TOR2 | tubulin alpha-4 chain | c	1620
Light-1	407	Background	AT1G04820	Symbols: TUA4, TOR2 | tubulin alpha-4 chain | c	1620
Light-1	407	Background	AT1G04820	Symbols: TUA4, TOR2 | tubulin alpha-4 chain | c	1620
Light-1	407	Background	AT1G04820	Symbols: TUA4, TOR2 | tubulin alpha-4 chain | c	1620
Light-1	407	Background	AT1G04820	Symbols: TUA4, TOR2 | tubulin alpha-4 chain | c	1620
Light-1	407	Background	AT1G04820	Symbols: TUA4, TOR2 | tubulin alpha-4 chain | c	1620
Light-1	407	Background	AT1G04820	Symbols: TUA4, TOR2 | tubulin alpha-4 chain | c	1620
Light-1	407	Background	AT1G04820	Symbols: TUA4, TOR2 | tubulin alpha-4 chain | c	1620
Light-1	408	Background	AT1G54270	Symbols: EIF4A-2 | eif4a-2 | chr1:20260495-202	1369
Light-1	408	Background	AT1G54270	Symbols: EIF4A-2 | eif4a-2 | chr1:20260495-202	1369
Light-1	408	Background	AT1G54270	Symbols: EIF4A-2 | eif4a-2 | chr1:20260495-202	1369
Light-1	408	Background	AT1G54270	Symbols: EIF4A-2 | eif4a-2 | chr1:20260495-202	1369
Light-1	408	Background	AT1G54270	Symbols: EIF4A-2 | eif4a-2 | chr1:20260495-202	1369
Light-1	408	Background	AT1G54270	Symbols: EIF4A-2 | eif4a-2 | chr1:20260495-202	1369
Light-1	408	Background	AT1G54270	Symbols: EIF4A-2 | eif4a-2 | chr1:20260495-202	1369
Light-1	408	Background	AT1G54270	Symbols: EIF4A-2 | eif4a-2 | chr1:20260495-202	1369
Light-1	408	Background	AT1G54270	Symbols: EIF4A-2 | eif4a-2 | chr1:20260495-202	1369
Light-1	408	Background	AT1G54270	Symbols: EIF4A-2 | eif4a-2 | chr1:20260495-202	1369
Light-1	408	Background	AT1G54270	Symbols: EIF4A-2 | eif4a-2 | chr1:20260495-202	1369
Light-1	408	Background	AT1G54270	Symbols: EIF4A-2 | eif4a-2 | chr1:20260495-202	1369
Light-1	408	Background	AT1G54270	Symbols: EIF4A-2 | eif4a-2 | chr1:20260495-202	1369
Light-1	408	Background	AT1G54270	Symbols: EIF4A-2 | eif4a-2 | chr1:20260495-202	1369
Light-1	408	Background	AT1G54270	Symbols: EIF4A-2 | eif4a-2 | chr1:20260495-202	1369
Light-1	408	Background	AT1G54270	Symbols: EIF4A-2 | eif4a-2 | chr1:20260495-202	1369
Light-1	408	Background	AT1G54270	Symbols: EIF4A-2 | eif4a-2 | chr1:20260495-202	1369
Light-1	409	Background	AT3G13920	Symbols: EIF4A1, RH4, TIF4A1 | eukaryotic trans	1349
Light-1	409	Background	AT3G13920	Symbols: EIF4A1, RH4, TIF4A1 | eukaryotic trans	1349
Light-1	409	Background	AT3G13920	Symbols: EIF4A1, RH4, TIF4A1 | eukaryotic trans	1349

Light-1	409	Background	AT3G13920	Symbols: EIF4A1, RH4, TIF4A1 | eukaryotic trans	1349
Light-1	409	Background	AT3G13920	Symbols: EIF4A1, RH4, TIF4A1 | eukaryotic trans	1349
Light-1	409	Background	AT3G13920	Symbols: EIF4A1, RH4, TIF4A1 | eukaryotic trans	1349
Light-1	409	Background	AT3G13920	Symbols: EIF4A1, RH4, TIF4A1 | eukaryotic trans	1349
Light-1	409	Background	AT3G13920	Symbols: EIF4A1, RH4, TIF4A1 | eukaryotic trans	1349
Light-1	409	Background	AT3G13920	Symbols: EIF4A1, RH4, TIF4A1 | eukaryotic trans	1349
Light-1	409	Background	AT3G13920	Symbols: EIF4A1, RH4, TIF4A1 | eukaryotic trans	1349
Light-1	409	Background	AT3G13920	Symbols: EIF4A1, RH4, TIF4A1 | eukaryotic trans	1349
Light-1	409	Background	AT3G13920	Symbols: EIF4A1, RH4, TIF4A1 | eukaryotic trans	1349
Light-1	409	Background	AT3G13920	Symbols: EIF4A1, RH4, TIF4A1 | eukaryotic trans	1349
Light-1	409	Background	AT3G13920	Symbols: EIF4A1, RH4, TIF4A1 | eukaryotic trans	1349
Light-1	409	Background	AT3G13920	Symbols: EIF4A1, RH4, TIF4A1 | eukaryotic trans	1349
Light-1	409	Background	AT3G13920	Symbols: EIF4A1, RH4, TIF4A1 | eukaryotic trans	1349
Light-1	409	Background	AT3G13920	Symbols: EIF4A1, RH4, TIF4A1 | eukaryotic trans	1349
Light-1	410	Background	AT5G19770	Symbols: TUA3 | tubulin alpha-3 | chr5:6682761	1257
Light-1	410	Background	AT5G19770	Symbols: TUA3 | tubulin alpha-3 | chr5:6682761	1257
Light-1	410	Background	AT5G19770	Symbols: TUA3 | tubulin alpha-3 | chr5:6682761	1257
Light-1	410	Background	AT5G19770	Symbols: TUA3 | tubulin alpha-3 | chr5:6682761	1257
Light-1	410	Background	AT5G19770	Symbols: TUA3 | tubulin alpha-3 | chr5:6682761	1257
Light-1	410	Background	AT5G19770	Symbols: TUA3 | tubulin alpha-3 | chr5:6682761	1257
Light-1	410	Background	AT5G19770	Symbols: TUA3 | tubulin alpha-3 | chr5:6682761	1257
Light-1	410	Background	AT5G19770	Symbols: TUA3 | tubulin alpha-3 | chr5:6682761	1257
Light-1	410	Background	AT5G19770	Symbols: TUA3 | tubulin alpha-3 | chr5:6682761	1257
Light-1	410	Background	AT5G19770	Symbols: TUA3 | tubulin alpha-3 | chr5:6682761	1257
Light-1	410	Background	AT5G19770	Symbols: TUA3 | tubulin alpha-3 | chr5:6682761	1257
Light-1	410	Background	AT5G19770	Symbols: TUA3 | tubulin alpha-3 | chr5:6682761	1257
Light-1	410	Background	AT5G19770	Symbols: TUA3 | tubulin alpha-3 | chr5:6682761	1257
Light-1	410	Background	AT5G19770	Symbols: TUA3 | tubulin alpha-3 | chr5:6682761	1257
Light-1	410	Background	AT5G19770	Symbols: TUA3 | tubulin alpha-3 | chr5:6682761	1257
Light-1	410	Background	AT5G19770	Symbols: TUA3 | tubulin alpha-3 | chr5:6682761	1257
Light-1	410	Background	AT5G19770	Symbols: TUA3 | tubulin alpha-3 | chr5:6682761	1257
Light-1	410	Background	AT5G19770	Symbols: TUA3 | tubulin alpha-3 | chr5:6682761	1257
Light-1	411	Background	AT4G20360	Symbols: ATRAB8D, ATRABE1B, RABE1b | RAB G	1111
Light-1	411	Background	AT4G20360	Symbols: ATRAB8D, ATRABE1B, RABE1b | RAB G	1111
Light-1	411	Background	AT4G20360	Symbols: ATRAB8D, ATRABE1B, RABE1b | RAB G	1111
Light-1	411	Background	AT4G20360	Symbols: ATRAB8D, ATRABE1B, RABE1b | RAB G	1111
Light-1	411	Background	AT4G20360	Symbols: ATRAB8D, ATRABE1B, RABE1b | RAB G	1111
Light-1	411	Background	AT4G20360	Symbols: ATRAB8D, ATRABE1B, RABE1b | RAB G	1111
Light-1	411	Background	AT4G20360	Symbols: ATRAB8D, ATRABE1B, RABE1b | RAB G	1111
Light-1	411	Background	AT4G20360	Symbols: ATRAB8D, ATRABE1B, RABE1b | RAB G	1111
Light-1	411	Background	AT4G20360	Symbols: ATRAB8D, ATRABE1B, RABE1b | RAB G	1111
Light-1	411	Background	AT4G20360	Symbols: ATRAB8D, ATRABE1B, RABE1b | RAB G	1111
Light-1	411	Background	AT4G20360	Symbols: ATRAB8D, ATRABE1B, RABE1b | RAB G	1111
Light-1	411	Background	AT4G20360	Symbols: ATRAB8D, ATRABE1B, RABE1b | RAB G	1111
Light-1	411	Background	AT4G20360	Symbols: ATRAB8D, ATRABE1B, RABE1b | RAB G	1111
Light-1	411	Background	AT4G20360	Symbols: ATRAB8D, ATRABE1B, RABE1b | RAB G	1111
Light-1	412	Background	AT3G48870	Symbols: ATCLPC, ATHSP93-III, HSP93-III | Clp A	1043
Light-1	412	Background	AT3G48870	Symbols: ATCLPC, ATHSP93-III, HSP93-III | Clp A	1043
Light-1	412	Background	AT3G48870	Symbols: ATCLPC, ATHSP93-III, HSP93-III | Clp A	1043
Light-1	412	Background	AT3G48870	Symbols: ATCLPC, ATHSP93-III, HSP93-III | Clp A	1043
Light-1	412	Background	AT3G48870	Symbols: ATCLPC, ATHSP93-III, HSP93-III | Clp A	1043
Light-1	412	Background	AT3G48870	Symbols: ATCLPC, ATHSP93-III, HSP93-III | Clp A	1043
Light-1	412	Background	AT3G48870	Symbols: ATCLPC, ATHSP93-III, HSP93-III | Clp A	1043
Light-1	412	Background	AT3G48870	Symbols: ATCLPC, ATHSP93-III, HSP93-III | Clp A	1043
Light-1	412	Background	AT3G48870	Symbols: ATCLPC, ATHSP93-III, HSP93-III | Clp A	1043
Light-1	412	Background	AT3G48870	Symbols: ATCLPC, ATHSP93-III, HSP93-III | Clp A	1043
Light-1	412	Background	AT3G48870	Symbols: ATCLPC, ATHSP93-III, HSP93-III | Clp A	1043
Light-1	412	Background	AT3G48870	Symbols: ATCLPC, ATHSP93-III, HSP93-III | Clp A	1043

Light-1	412	Background	AT3G48870	Symbols: ATCLPC, ATHSP93-III, HSP93-III | Clp A	1043
Light-1	412	Background	AT3G48870	Symbols: ATCLPC, ATHSP93-III, HSP93-III | Clp A	1043
Light-1	412	Background	AT3G48870	Symbols: ATCLPC, ATHSP93-III, HSP93-III | Clp A	1043
Light-1	412	Background	AT3G48870	Symbols: ATCLPC, ATHSP93-III, HSP93-III | Clp A	1043
Light-1	412	Background	AT3G48870	Symbols: ATCLPC, ATHSP93-III, HSP93-III | Clp A	1043
Light-1	412	Background	AT3G48870	Symbols: ATCLPC, ATHSP93-III, HSP93-III | Clp A	1043
Light-1	412	Background	AT3G48870	Symbols: ATCLPC, ATHSP93-III, HSP93-III | Clp A	1043
Light-1	412	Background	AT3G48870	Symbols: ATCLPC, ATHSP93-III, HSP93-III | Clp A	1043
Light-1	412	Background	AT3G48870	Symbols: ATCLPC, ATHSP93-III, HSP93-III | Clp A	1043
Light-1	412	Background	AT3G48870	Symbols: ATCLPC, ATHSP93-III, HSP93-III | Clp A	1043
Light-1	412	Background	AT3G48870	Symbols: ATCLPC, ATHSP93-III, HSP93-III | Clp A	1043
Light-1	412	Background	AT3G48870	Symbols: ATCLPC, ATHSP93-III, HSP93-III | Clp A	1043
Light-1	412	Background	AT3G48870	Symbols: ATCLPC, ATHSP93-III, HSP93-III | Clp A	1043
Light-1	412	Background	AT3G48870	Symbols: ATCLPC, ATHSP93-III, HSP93-III | Clp A	1043
Light-1	412	Background	AT3G48870	Symbols: ATCLPC, ATHSP93-III, HSP93-III | Clp A	1043
Light-1	412	Background	AT3G48870	Symbols: ATCLPC, ATHSP93-III, HSP93-III | Clp A	1043
Light-1	412	Background	AT3G48870	Symbols: ATCLPC, ATHSP93-III, HSP93-III | Clp A	1043
Light-1	413	Background	AT5G12250	Symbols: TUB6 | beta-6 tubulin | chr5:3961317-	1042
Light-1	413	Background	AT5G12250	Symbols: TUB6 | beta-6 tubulin | chr5:3961317-	1042
Light-1	413	Background	AT5G12250	Symbols: TUB6 | beta-6 tubulin | chr5:3961317-	1042
Light-1	413	Background	AT5G12250	Symbols: TUB6 | beta-6 tubulin | chr5:3961317-	1042
Light-1	413	Background	AT5G12250	Symbols: TUB6 | beta-6 tubulin | chr5:3961317-	1042
Light-1	413	Background	AT5G12250	Symbols: TUB6 | beta-6 tubulin | chr5:3961317-	1042
Light-1	413	Background	AT5G12250	Symbols: TUB6 | beta-6 tubulin | chr5:3961317-	1042
Light-1	413	Background	AT5G12250	Symbols: TUB6 | beta-6 tubulin | chr5:3961317-	1042
Light-1	413	Background	AT5G12250	Symbols: TUB6 | beta-6 tubulin | chr5:3961317-	1042
Light-1	413	Background	AT5G12250	Symbols: TUB6 | beta-6 tubulin | chr5:3961317-	1042
Light-1	413	Background	AT5G12250	Symbols: TUB6 | beta-6 tubulin | chr5:3961317-	1042
Light-1	413	Background	AT5G12250	Symbols: TUB6 | beta-6 tubulin | chr5:3961317-	1042
Light-1	413	Background	AT5G12250	Symbols: TUB6 | beta-6 tubulin | chr5:3961317-	1042
Light-1	413	Background	AT5G12250	Symbols: TUB6 | beta-6 tubulin | chr5:3961317-	1042
Light-1	413	Background	AT5G12250	Symbols: TUB6 | beta-6 tubulin | chr5:3961317-	1042
Light-1	413	Background	AT5G12250	Symbols: TUB6 | beta-6 tubulin | chr5:3961317-	1042
Light-1	413	Background	AT5G12250	Symbols: TUB6 | beta-6 tubulin | chr5:3961317-	1042
Light-1	413	Background	AT5G12250	Symbols: TUB6 | beta-6 tubulin | chr5:3961317-	1042
Light-1	413	Background	AT5G12250	Symbols: TUB6 | beta-6 tubulin | chr5:3961317-	1042
Light-1	414	Background	AT5G02500	Symbols: HSC70-1, HSP70-1, AT-HSC70-1, HSC70	1015
Light-1	414	Background	AT5G02500	Symbols: HSC70-1, HSP70-1, AT-HSC70-1, HSC70	1015
Light-1	414	Background	AT5G02500	Symbols: HSC70-1, HSP70-1, AT-HSC70-1, HSC70	1015
Light-1	414	Background	AT5G02500	Symbols: HSC70-1, HSP70-1, AT-HSC70-1, HSC70	1015
Light-1	414	Background	AT5G02500	Symbols: HSC70-1, HSP70-1, AT-HSC70-1, HSC70	1015
Light-1	414	Background	AT5G02500	Symbols: HSC70-1, HSP70-1, AT-HSC70-1, HSC70	1015
Light-1	414	Background	AT5G02500	Symbols: HSC70-1, HSP70-1, AT-HSC70-1, HSC70	1015
Light-1	414	Background	AT5G02500	Symbols: HSC70-1, HSP70-1, AT-HSC70-1, HSC70	1015
Light-1	414	Background	AT5G02500	Symbols: HSC70-1, HSP70-1, AT-HSC70-1, HSC70	1015
Light-1	414	Background	AT5G02500	Symbols: HSC70-1, HSP70-1, AT-HSC70-1, HSC70	1015
Light-1	414	Background	AT5G02500	Symbols: HSC70-1, HSP70-1, AT-HSC70-1, HSC70	1015
Light-1	414	Background	AT5G02500	Symbols: HSC70-1, HSP70-1, AT-HSC70-1, HSC70	1015
Light-1	414	Background	AT5G02500	Symbols: HSC70-1, HSP70-1, AT-HSC70-1, HSC70	1015
Light-1	414	Background	AT5G02500	Symbols: HSC70-1, HSP70-1, AT-HSC70-1, HSC70	1015
Light-1	414	Background	AT5G02500	Symbols: HSC70-1, HSP70-1, AT-HSC70-1, HSC70	1015
Light-1	414	Background	AT5G02500	Symbols: HSC70-1, HSP70-1, AT-HSC70-1, HSC70	1015
Light-1	414	Background	AT5G02500	Symbols: HSC70-1, HSP70-1, AT-HSC70-1, HSC70	1015
Light-1	414	Background	AT5G02500	Symbols: HSC70-1, HSP70-1, AT-HSC70-1, HSC70	1015
Light-1	414	Background	AT5G02500	Symbols: HSC70-1, HSP70-1, AT-HSC70-1, HSC70	1015
Light-1	414	Background	AT5G02500	Symbols: HSC70-1, HSP70-1, AT-HSC70-1, HSC70	1015
Light-1	415	Background	AT1G75780	Symbols: TUB1 | tubulin beta-1 chain | chr1:284	978
Light-1	415	Background	AT1G75780	Symbols: TUB1 | tubulin beta-1 chain | chr1:284	978

Light-1	415	Background	AT1G75780	Symbols: TUB1 | tubulin beta-1 chain | chr1:284	978
Light-1	415	Background	AT1G75780	Symbols: TUB1 | tubulin beta-1 chain | chr1:284	978
Light-1	415	Background	AT1G75780	Symbols: TUB1 | tubulin beta-1 chain | chr1:284	978
Light-1	415	Background	AT1G75780	Symbols: TUB1 | tubulin beta-1 chain | chr1:284	978
Light-1	415	Background	AT1G75780	Symbols: TUB1 | tubulin beta-1 chain | chr1:284	978
Light-1	415	Background	AT1G75780	Symbols: TUB1 | tubulin beta-1 chain | chr1:284	978
Light-1	415	Background	AT1G75780	Symbols: TUB1 | tubulin beta-1 chain | chr1:284	978
Light-1	415	Background	AT1G75780	Symbols: TUB1 | tubulin beta-1 chain | chr1:284	978
Light-1	415	Background	AT1G75780	Symbols: TUB1 | tubulin beta-1 chain | chr1:284	978
Light-1	415	Background	AT1G75780	Symbols: TUB1 | tubulin beta-1 chain | chr1:284	978
Light-1	415	Background	AT1G75780	Symbols: TUB1 | tubulin beta-1 chain | chr1:284	978
Light-1	415	Background	AT1G75780	Symbols: TUB1 | tubulin beta-1 chain | chr1:284	978
Light-1	415	Background	AT1G75780	Symbols: TUB1 | tubulin beta-1 chain | chr1:284	978
Light-1	415	Background	AT1G75780	Symbols: TUB1 | tubulin beta-1 chain | chr1:284	978
Light-1	416	Background	AT3G09440	Symbols: | Heat shock protein 70 (Hsp 70) famil	935
Light-1	416	Background	AT3G09440	Symbols: | Heat shock protein 70 (Hsp 70) famil	935
Light-1	416	Background	AT3G09440	Symbols: | Heat shock protein 70 (Hsp 70) famil	935
Light-1	416	Background	AT3G09440	Symbols: | Heat shock protein 70 (Hsp 70) famil	935
Light-1	416	Background	AT3G09440	Symbols: | Heat shock protein 70 (Hsp 70) famil	935
Light-1	416	Background	AT3G09440	Symbols: | Heat shock protein 70 (Hsp 70) famil	935
Light-1	416	Background	AT3G09440	Symbols: | Heat shock protein 70 (Hsp 70) famil	935
Light-1	416	Background	AT3G09440	Symbols: | Heat shock protein 70 (Hsp 70) famil	935
Light-1	416	Background	AT3G09440	Symbols: | Heat shock protein 70 (Hsp 70) famil	935
Light-1	416	Background	AT3G09440	Symbols: | Heat shock protein 70 (Hsp 70) famil	935
Light-1	416	Background	AT3G09440	Symbols: | Heat shock protein 70 (Hsp 70) famil	935
Light-1	416	Background	AT3G09440	Symbols: | Heat shock protein 70 (Hsp 70) famil	935
Light-1	416	Background	AT3G09440	Symbols: | Heat shock protein 70 (Hsp 70) famil	935
Light-1	416	Background	AT3G09440	Symbols: | Heat shock protein 70 (Hsp 70) famil	935
Light-1	416	Background	AT3G09440	Symbols: | Heat shock protein 70 (Hsp 70) famil	935
Light-1	416	Background	AT3G09440	Symbols: | Heat shock protein 70 (Hsp 70) famil	935
Light-1	416	Background	AT3G09440	Symbols: | Heat shock protein 70 (Hsp 70) famil	935
Light-1	416	Background	AT3G09440	Symbols: | Heat shock protein 70 (Hsp 70) famil	935
Light-1	417	Background	AT2G02560	Symbols: CAND1, ATCAND1, ETA2, TIP120, HVE	843
Light-1	417	Background	AT2G02560	Symbols: CAND1, ATCAND1, ETA2, TIP120, HVE	843
Light-1	417	Background	AT2G02560	Symbols: CAND1, ATCAND1, ETA2, TIP120, HVE	843
Light-1	417	Background	AT2G02560	Symbols: CAND1, ATCAND1, ETA2, TIP120, HVE	843
Light-1	417	Background	AT2G02560	Symbols: CAND1, ATCAND1, ETA2, TIP120, HVE	843
Light-1	417	Background	AT2G02560	Symbols: CAND1, ATCAND1, ETA2, TIP120, HVE	843
Light-1	417	Background	AT2G02560	Symbols: CAND1, ATCAND1, ETA2, TIP120, HVE	843
Light-1	417	Background	AT2G02560	Symbols: CAND1, ATCAND1, ETA2, TIP120, HVE	843
Light-1	417	Background	AT2G02560	Symbols: CAND1, ATCAND1, ETA2, TIP120, HVE	843
Light-1	417	Background	AT2G02560	Symbols: CAND1, ATCAND1, ETA2, TIP120, HVE	843
Light-1	417	Background	AT2G02560	Symbols: CAND1, ATCAND1, ETA2, TIP120, HVE	843
Light-1	417	Background	AT2G02560	Symbols: CAND1, ATCAND1, ETA2, TIP120, HVE	843
Light-1	417	Background	AT2G02560	Symbols: CAND1, ATCAND1, ETA2, TIP120, HVE	843
Light-1	417	Background	AT2G02560	Symbols: CAND1, ATCAND1, ETA2, TIP120, HVE	843
Light-1	417	Background	AT2G02560	Symbols: CAND1, ATCAND1, ETA2, TIP120, HVE	843
Light-1	417	Background	AT2G02560	Symbols: CAND1, ATCAND1, ETA2, TIP120, HVE	843
Light-1	417	Background	AT2G02560	Symbols: CAND1, ATCAND1, ETA2, TIP120, HVE	843
Light-1	417	Background	AT2G02560	Symbols: CAND1, ATCAND1, ETA2, TIP120, HVE	843
Light-1	418	Background	AT5G50920	Symbols: CLPC, ATHSP93-V, HSP93-V, DCA1, CLP	780
Light-1	418	Background	AT5G50920	Symbols: CLPC, ATHSP93-V, HSP93-V, DCA1, CLP	780
Light-1	418	Background	AT5G50920	Symbols: CLPC, ATHSP93-V, HSP93-V, DCA1, CLP	780
Light-1	418	Background	AT5G50920	Symbols: CLPC, ATHSP93-V, HSP93-V, DCA1, CLP	780
Light-1	418	Background	AT5G50920	Symbols: CLPC, ATHSP93-V, HSP93-V, DCA1, CLP	780
Light-1	418	Background	AT5G50920	Symbols: CLPC, ATHSP93-V, HSP93-V, DCA1, CLP	780
Light-1	418	Background	AT5G50920	Symbols: CLPC, ATHSP93-V, HSP93-V, DCA1, CLP	780
Light-1	418	Background	AT5G50920	Symbols: CLPC, ATHSP93-V, HSP93-V, DCA1, CLP	780

Light-1	418	Background	AT5G50920	Symbols: CLPC, ATHSP93-V, HSP93-V, DCA1, CLP	780
Light-1	418	Background	AT5G50920	Symbols: CLPC, ATHSP93-V, HSP93-V, DCA1, CLP	780
Light-1	418	Background	AT5G50920	Symbols: CLPC, ATHSP93-V, HSP93-V, DCA1, CLP	780
Light-1	418	Background	AT5G50920	Symbols: CLPC, ATHSP93-V, HSP93-V, DCA1, CLP	780
Light-1	418	Background	AT5G50920	Symbols: CLPC, ATHSP93-V, HSP93-V, DCA1, CLP	780
Light-1	418	Background	AT5G50920	Symbols: CLPC, ATHSP93-V, HSP93-V, DCA1, CLP	780
Light-1	418	Background	AT5G50920	Symbols: CLPC, ATHSP93-V, HSP93-V, DCA1, CLP	780
Light-1	418	Background	AT5G50920	Symbols: CLPC, ATHSP93-V, HSP93-V, DCA1, CLP	780
Light-1	418	Background	AT5G50920	Symbols: CLPC, ATHSP93-V, HSP93-V, DCA1, CLP	780
Light-1	418	Background	AT5G50920	Symbols: CLPC, ATHSP93-V, HSP93-V, DCA1, CLP	780
Light-1	418	Background	AT5G50920	Symbols: CLPC, ATHSP93-V, HSP93-V, DCA1, CLP	780
Light-1	418	Background	AT5G50920	Symbols: CLPC, ATHSP93-V, HSP93-V, DCA1, CLP	780
Light-1	418	Background	AT5G50920	Symbols: CLPC, ATHSP93-V, HSP93-V, DCA1, CLP	780
Light-1	419	Background	AT2G20580	Symbols: RPN1A, ATRPN1A | 26S proteasome re	700
Light-1	419	Background	AT2G20580	Symbols: RPN1A, ATRPN1A | 26S proteasome re	700
Light-1	419	Background	AT2G20580	Symbols: RPN1A, ATRPN1A | 26S proteasome re	700
Light-1	419	Background	AT2G20580	Symbols: RPN1A, ATRPN1A | 26S proteasome re	700
Light-1	419	Background	AT2G20580	Symbols: RPN1A, ATRPN1A | 26S proteasome re	700
Light-1	419	Background	AT2G20580	Symbols: RPN1A, ATRPN1A | 26S proteasome re	700
Light-1	419	Background	AT2G20580	Symbols: RPN1A, ATRPN1A | 26S proteasome re	700
Light-1	419	Background	AT2G20580	Symbols: RPN1A, ATRPN1A | 26S proteasome re	700
Light-1	419	Background	AT2G20580	Symbols: RPN1A, ATRPN1A | 26S proteasome re	700
Light-1	419	Background	AT2G20580	Symbols: RPN1A, ATRPN1A | 26S proteasome re	700
Light-1	419	Background	AT2G20580	Symbols: RPN1A, ATRPN1A | 26S proteasome re	700
Light-1	419	Background	AT2G20580	Symbols: RPN1A, ATRPN1A | 26S proteasome re	700
Light-1	419	Background	AT2G20580	Symbols: RPN1A, ATRPN1A | 26S proteasome re	700
Light-1	419	Background	AT2G20580	Symbols: RPN1A, ATRPN1A | 26S proteasome re	700
Light-1	419	Background	AT2G20580	Symbols: RPN1A, ATRPN1A | 26S proteasome re	700
Light-1	420	Background	AT1G07920	Symbols: | GTP binding Elongation factor Tu fan	698
Light-1	420	Background	AT1G07920	Symbols: | GTP binding Elongation factor Tu fan	698
Light-1	420	Background	AT1G07920	Symbols: | GTP binding Elongation factor Tu fan	698
Light-1	420	Background	AT1G07920	Symbols: | GTP binding Elongation factor Tu fan	698
Light-1	420	Background	AT1G07920	Symbols: | GTP binding Elongation factor Tu fan	698
Light-1	420	Background	AT1G07920	Symbols: | GTP binding Elongation factor Tu fan	698
Light-1	420	Background	AT1G07920	Symbols: | GTP binding Elongation factor Tu fan	698
Light-1	420	Background	AT1G07920	Symbols: | GTP binding Elongation factor Tu fan	698
Light-1	420	Background	AT1G07920	Symbols: | GTP binding Elongation factor Tu fan	698
Light-1	420	Background	AT1G07920	Symbols: | GTP binding Elongation factor Tu fan	698
Light-1	420	Background	AT1G07920	Symbols: | GTP binding Elongation factor Tu fan	698
Light-1	421	Background	AT5G02490	Symbols: | Heat shock protein 70 (Hsp 70) famil	692
Light-1	421	Background	AT5G02490	Symbols: | Heat shock protein 70 (Hsp 70) famil	692
Light-1	421	Background	AT5G02490	Symbols: | Heat shock protein 70 (Hsp 70) famil	692
Light-1	421	Background	AT5G02490	Symbols: | Heat shock protein 70 (Hsp 70) famil	692
Light-1	421	Background	AT5G02490	Symbols: | Heat shock protein 70 (Hsp 70) famil	692
Light-1	421	Background	AT5G02490	Symbols: | Heat shock protein 70 (Hsp 70) famil	692
Light-1	421	Background	AT5G02490	Symbols: | Heat shock protein 70 (Hsp 70) famil	692
Light-1	421	Background	AT5G02490	Symbols: | Heat shock protein 70 (Hsp 70) famil	692
Light-1	421	Background	AT5G02490	Symbols: | Heat shock protein 70 (Hsp 70) famil	692
Light-1	421	Background	AT5G02490	Symbols: | Heat shock protein 70 (Hsp 70) famil	692
Light-1	421	Background	AT5G02490	Symbols: | Heat shock protein 70 (Hsp 70) famil	692
Light-1	421	Background	AT5G02490	Symbols: | Heat shock protein 70 (Hsp 70) famil	692
Light-1	421	Background	AT5G02490	Symbols: | Heat shock protein 70 (Hsp 70) famil	692
Light-1	422	Background	AT5G03290	Symbols: IDH-V | isocitrate dehydrogenase V | c	684
Light-1	422	Background	AT5G03290	Symbols: IDH-V | isocitrate dehydrogenase V | c	684
Light-1	422	Background	AT5G03290	Symbols: IDH-V | isocitrate dehydrogenase V | c	684
Light-1	422	Background	AT5G03290	Symbols: IDH-V | isocitrate dehydrogenase V | c	684
Light-1	422	Background	AT5G03290	Symbols: IDH-V | isocitrate dehydrogenase V | c	684
Light-1	422	Background	AT5G03290	Symbols: IDH-V | isocitrate dehydrogenase V | c	684

Light-1	422	Background	AT5G03290	Symbols: IDH-V | isocitrate dehydrogenase V | c	684
Light-1	422	Background	AT5G03290	Symbols: IDH-V | isocitrate dehydrogenase V | c	684
Light-1	422	Background	AT5G03290	Symbols: IDH-V | isocitrate dehydrogenase V | c	684
Light-1	422	Background	AT5G03290	Symbols: IDH-V | isocitrate dehydrogenase V | c	684
Light-1	422	Background	AT5G03290	Symbols: IDH-V | isocitrate dehydrogenase V | c	684
Light-1	423	Background	AT3G12580	Symbols: HSP70, ATHSP70 | heat shock protein	684
Light-1	423	Background	AT3G12580	Symbols: HSP70, ATHSP70 | heat shock protein	684
Light-1	423	Background	AT3G12580	Symbols: HSP70, ATHSP70 | heat shock protein	684
Light-1	423	Background	AT3G12580	Symbols: HSP70, ATHSP70 | heat shock protein	684
Light-1	423	Background	AT3G12580	Symbols: HSP70, ATHSP70 | heat shock protein	684
Light-1	423	Background	AT3G12580	Symbols: HSP70, ATHSP70 | heat shock protein	684
Light-1	423	Background	AT3G12580	Symbols: HSP70, ATHSP70 | heat shock protein	684
Light-1	423	Background	AT3G12580	Symbols: HSP70, ATHSP70 | heat shock protein	684
Light-1	423	Background	AT3G12580	Symbols: HSP70, ATHSP70 | heat shock protein	684
Light-1	423	Background	AT3G12580	Symbols: HSP70, ATHSP70 | heat shock protein	684
Light-1	423	Background	AT3G12580	Symbols: HSP70, ATHSP70 | heat shock protein	684
Light-1	423	Background	AT3G12580	Symbols: HSP70, ATHSP70 | heat shock protein	684
Light-1	423	Background	AT3G12580	Symbols: HSP70, ATHSP70 | heat shock protein	684
Light-1	423	Background	AT3G12580	Symbols: HSP70, ATHSP70 | heat shock protein	684
Light-1	423	Background	AT3G12580	Symbols: HSP70, ATHSP70 | heat shock protein	684
Light-1	423	Background	AT3G12580	Symbols: HSP70, ATHSP70 | heat shock protein	684
Light-1	424	Background	AT1G67120	Symbols: | ATPases;nucleotide binding;ATP binc	628
Light-1	424	Background	AT1G67120	Symbols: | ATPases;nucleotide binding;ATP binc	628
Light-1	424	Background	AT1G67120	Symbols: | ATPases;nucleotide binding;ATP binc	628
Light-1	424	Background	AT1G67120	Symbols: | ATPases;nucleotide binding;ATP binc	628
Light-1	424	Background	AT1G67120	Symbols: | ATPases;nucleotide binding;ATP binc	628
Light-1	424	Background	AT1G67120	Symbols: | ATPases;nucleotide binding;ATP binc	628
Light-1	424	Background	AT1G67120	Symbols: | ATPases;nucleotide binding;ATP binc	628
Light-1	424	Background	AT1G67120	Symbols: | ATPases;nucleotide binding;ATP binc	628
Light-1	424	Background	AT1G67120	Symbols: | ATPases;nucleotide binding;ATP binc	628
Light-1	424	Background	AT1G67120	Symbols: | ATPases;nucleotide binding;ATP binc	628
Light-1	424	Background	AT1G67120	Symbols: | ATPases;nucleotide binding;ATP binc	628
Light-1	424	Background	AT1G67120	Symbols: | ATPases;nucleotide binding;ATP binc	628
Light-1	424	Background	AT1G67120	Symbols: | ATPases;nucleotide binding;ATP binc	628
Light-1	424	Background	AT1G67120	Symbols: | ATPases;nucleotide binding;ATP binc	628
Light-1	424	Background	AT1G67120	Symbols: | ATPases;nucleotide binding;ATP binc	628
Light-1	424	Background	AT1G67120	Symbols: | ATPases;nucleotide binding;ATP binc	628
Light-1	425	Background	AT1G64790	Symbols: ILA | ILITYHIA | chr1:24065232-240819	577
Light-1	425	Background	AT1G64790	Symbols: ILA | ILITYHIA | chr1:24065232-240819	577
Light-1	425	Background	AT1G64790	Symbols: ILA | ILITYHIA | chr1:24065232-240819	577
Light-1	425	Background	AT1G64790	Symbols: ILA | ILITYHIA | chr1:24065232-240819	577
Light-1	425	Background	AT1G64790	Symbols: ILA | ILITYHIA | chr1:24065232-240819	577
Light-1	425	Background	AT1G64790	Symbols: ILA | ILITYHIA | chr1:24065232-240819	577
Light-1	425	Background	AT1G64790	Symbols: ILA | ILITYHIA | chr1:24065232-240819	577
Light-1	425	Background	AT1G64790	Symbols: ILA | ILITYHIA | chr1:24065232-240819	577
Light-1	425	Background	AT1G64790	Symbols: ILA | ILITYHIA | chr1:24065232-240819	577
Light-1	425	Background	AT1G64790	Symbols: ILA | ILITYHIA | chr1:24065232-240819	577
Light-1	426	Background	AT5G43010	Symbols: RPT4A | regulatory particle triple-A AT	574
Light-1	426	Background	AT5G43010	Symbols: RPT4A | regulatory particle triple-A AT	574
Light-1	426	Background	AT5G43010	Symbols: RPT4A | regulatory particle triple-A AT	574
Light-1	426	Background	AT5G43010	Symbols: RPT4A | regulatory particle triple-A AT	574
Light-1	426	Background	AT5G43010	Symbols: RPT4A | regulatory particle triple-A AT	574
Light-1	426	Background	AT5G43010	Symbols: RPT4A | regulatory particle triple-A AT	574
Light-1	426	Background	AT5G43010	Symbols: RPT4A | regulatory particle triple-A AT	574
Light-1	426	Background	AT5G43010	Symbols: RPT4A | regulatory particle triple-A AT	574
Light-1	426	Background	AT5G43010	Symbols: RPT4A | regulatory particle triple-A AT	574
Light-1	426	Background	AT5G43010	Symbols: RPT4A | regulatory particle triple-A AT	574
Light-1	426	Background	AT5G43010	Symbols: RPT4A | regulatory particle triple-A AT	574

Light-1	427	Background	AT2G39730	Symbols: RCA | rubisco activase | chr2:1657095	559
Light-1	427	Background	AT2G39730	Symbols: RCA | rubisco activase | chr2:1657095	559
Light-1	427	Background	AT2G39730	Symbols: RCA | rubisco activase | chr2:1657095	559
Light-1	427	Background	AT2G39730	Symbols: RCA | rubisco activase | chr2:1657095	559
Light-1	427	Background	AT2G39730	Symbols: RCA | rubisco activase | chr2:1657095	559
Light-1	427	Background	AT2G39730	Symbols: RCA | rubisco activase | chr2:1657095	559
Light-1	427	Background	AT2G39730	Symbols: RCA | rubisco activase | chr2:1657095	559
Light-1	427	Background	AT2G39730	Symbols: RCA | rubisco activase | chr2:1657095	559
Light-1	428	Background	AT5G19820	Symbols: emb2734 | ARM repeat superfamily pr	540
Light-1	428	Background	AT5G19820	Symbols: emb2734 | ARM repeat superfamily pr	540
Light-1	428	Background	AT5G19820	Symbols: emb2734 | ARM repeat superfamily pr	540
Light-1	428	Background	AT5G19820	Symbols: emb2734 | ARM repeat superfamily pr	540
Light-1	428	Background	AT5G19820	Symbols: emb2734 | ARM repeat superfamily pr	540
Light-1	428	Background	AT5G19820	Symbols: emb2734 | ARM repeat superfamily pr	540
Light-1	428	Background	AT5G19820	Symbols: emb2734 | ARM repeat superfamily pr	540
Light-1	428	Background	AT5G19820	Symbols: emb2734 | ARM repeat superfamily pr	540
Light-1	428	Background	AT5G19820	Symbols: emb2734 | ARM repeat superfamily pr	540
Light-1	428	Background	AT5G19820	Symbols: emb2734 | ARM repeat superfamily pr	540
Light-1	428	Background	AT5G19820	Symbols: emb2734 | ARM repeat superfamily pr	540
Light-1	428	Background	AT5G19820	Symbols: emb2734 | ARM repeat superfamily pr	540
Light-1	428	Background	AT5G19820	Symbols: emb2734 | ARM repeat superfamily pr	540
Light-1	429	Background	AT1G09100	Symbols: RPT5B | 26S proteasome AAA-ATPase	534
Light-1	429	Background	AT1G09100	Symbols: RPT5B | 26S proteasome AAA-ATPase	534
Light-1	429	Background	AT1G09100	Symbols: RPT5B | 26S proteasome AAA-ATPase	534
Light-1	429	Background	AT1G09100	Symbols: RPT5B | 26S proteasome AAA-ATPase	534
Light-1	429	Background	AT1G09100	Symbols: RPT5B | 26S proteasome AAA-ATPase	534
Light-1	429	Background	AT1G09100	Symbols: RPT5B | 26S proteasome AAA-ATPase	534
Light-1	429	Background	AT1G09100	Symbols: RPT5B | 26S proteasome AAA-ATPase	534
Light-1	429	Background	AT1G09100	Symbols: RPT5B | 26S proteasome AAA-ATPase	534
Light-1	429	Background	AT1G09100	Symbols: RPT5B | 26S proteasome AAA-ATPase	534
Light-1	430	Background	AT4G37910	Symbols: mtHsc70-1 | mitochondrial heat shock	520
Light-1	430	Background	AT4G37910	Symbols: mtHsc70-1 | mitochondrial heat shock	520
Light-1	430	Background	AT4G37910	Symbols: mtHsc70-1 | mitochondrial heat shock	520
Light-1	430	Background	AT4G37910	Symbols: mtHsc70-1 | mitochondrial heat shock	520
Light-1	430	Background	AT4G37910	Symbols: mtHsc70-1 | mitochondrial heat shock	520
Light-1	430	Background	AT4G37910	Symbols: mtHsc70-1 | mitochondrial heat shock	520
Light-1	430	Background	AT4G37910	Symbols: mtHsc70-1 | mitochondrial heat shock	520
Light-1	430	Background	AT4G37910	Symbols: mtHsc70-1 | mitochondrial heat shock	520
Light-1	430	Background	AT4G37910	Symbols: mtHsc70-1 | mitochondrial heat shock	520
Light-1	430	Background	AT4G37910	Symbols: mtHsc70-1 | mitochondrial heat shock	520
Light-1	430	Background	AT4G37910	Symbols: mtHsc70-1 | mitochondrial heat shock	520
Light-1	431	Background	AT3G09810	Symbols: IDH-VI | isocitrate dehydrogenase VI |	512
Light-1	431	Background	AT3G09810	Symbols: IDH-VI | isocitrate dehydrogenase VI |	512
Light-1	431	Background	AT3G09810	Symbols: IDH-VI | isocitrate dehydrogenase VI |	512
Light-1	431	Background	AT3G09810	Symbols: IDH-VI | isocitrate dehydrogenase VI |	512
Light-1	431	Background	AT3G09810	Symbols: IDH-VI | isocitrate dehydrogenase VI |	512
Light-1	431	Background	AT3G09810	Symbols: IDH-VI | isocitrate dehydrogenase VI |	512
Light-1	431	Background	AT3G09810	Symbols: IDH-VI | isocitrate dehydrogenase VI |	512
Light-1	431	Background	AT3G09810	Symbols: IDH-VI | isocitrate dehydrogenase VI |	512
Light-1	431	Background	AT3G09810	Symbols: IDH-VI | isocitrate dehydrogenase VI |	512
Light-1	431	Background	AT3G09810	Symbols: IDH-VI | isocitrate dehydrogenase VI |	512
Light-1	431	Background	AT3G09810	Symbols: IDH-VI | isocitrate dehydrogenase VI |	512
Light-1	432	Background	AT3G56150	Symbols: EIF3C, ATEIF3C-1, EIF3C-1, ATTIF3C1, T	503
Light-1	432	Background	AT3G56150	Symbols: EIF3C, ATEIF3C-1, EIF3C-1, ATTIF3C1, T	503
Light-1	432	Background	AT3G56150	Symbols: EIF3C, ATEIF3C-1, EIF3C-1, ATTIF3C1, T	503
Light-1	432	Background	AT3G56150	Symbols: EIF3C, ATEIF3C-1, EIF3C-1, ATTIF3C1, T	503
Light-1	432	Background	AT3G56150	Symbols: EIF3C, ATEIF3C-1, EIF3C-1, ATTIF3C1, T	503
Light-1	432	Background	AT3G56150	Symbols: EIF3C, ATEIF3C-1, EIF3C-1, ATTIF3C1, T	503

Light-1	432	Background	AT3G56150	Symbols: EIF3C, ATEIF3C-1, EIF3C-1, ATTIF3C1, T	503
Light-1	432	Background	AT3G56150	Symbols: EIF3C, ATEIF3C-1, EIF3C-1, ATTIF3C1, T	503
Light-1	432	Background	AT3G56150	Symbols: EIF3C, ATEIF3C-1, EIF3C-1, ATTIF3C1, T	503
Light-1	432	Background	AT3G56150	Symbols: EIF3C, ATEIF3C-1, EIF3C-1, ATTIF3C1, T	503
Light-1	433	Background	AT3G22330	Symbols: PMH2, ATRH53 | putative mitochondri	499
Light-1	433	Background	AT3G22330	Symbols: PMH2, ATRH53 | putative mitochondri	499
Light-1	433	Background	AT3G22330	Symbols: PMH2, ATRH53 | putative mitochondri	499
Light-1	433	Background	AT3G22330	Symbols: PMH2, ATRH53 | putative mitochondri	499
Light-1	433	Background	AT3G22330	Symbols: PMH2, ATRH53 | putative mitochondri	499
Light-1	433	Background	AT3G22330	Symbols: PMH2, ATRH53 | putative mitochondri	499
Light-1	433	Background	AT3G22330	Symbols: PMH2, ATRH53 | putative mitochondri	499
Light-1	433	Background	AT3G22330	Symbols: PMH2, ATRH53 | putative mitochondri	499
Light-1	433	Background	AT3G22330	Symbols: PMH2, ATRH53 | putative mitochondri	499
Light-1	433	Background	AT3G22330	Symbols: PMH2, ATRH53 | putative mitochondri	499
Light-1	433	Background	AT3G22330	Symbols: PMH2, ATRH53 | putative mitochondri	499
Light-1	433	Background	AT3G22330	Symbols: PMH2, ATRH53 | putative mitochondri	499
Light-1	434	Background	AT1G13440	Symbols: GAPC-2, GAPC2 | glyceraldehyde-3-ph	498
Light-1	434	Background	AT1G13440	Symbols: GAPC-2, GAPC2 | glyceraldehyde-3-ph	498
Light-1	434	Background	AT1G13440	Symbols: GAPC-2, GAPC2 | glyceraldehyde-3-ph	498
Light-1	434	Background	AT1G13440	Symbols: GAPC-2, GAPC2 | glyceraldehyde-3-ph	498
Light-1	434	Background	AT1G13440	Symbols: GAPC-2, GAPC2 | glyceraldehyde-3-ph	498
Light-1	434	Background	AT1G13440	Symbols: GAPC-2, GAPC2 | glyceraldehyde-3-ph	498
Light-1	434	Background	AT1G13440	Symbols: GAPC-2, GAPC2 | glyceraldehyde-3-ph	498
Light-1	434	Background	AT1G13440	Symbols: GAPC-2, GAPC2 | glyceraldehyde-3-ph	498
Light-1	434	Background	AT1G13440	Symbols: GAPC-2, GAPC2 | glyceraldehyde-3-ph	498
Light-1	434	Background	AT1G13440	Symbols: GAPC-2, GAPC2 | glyceraldehyde-3-ph	498
Light-1	435	Background	AT5G09590	Symbols: MTHSC70-2, HSC70-5 | mitochondrial	486
Light-1	435	Background	AT5G09590	Symbols: MTHSC70-2, HSC70-5 | mitochondrial	486
Light-1	435	Background	AT5G09590	Symbols: MTHSC70-2, HSC70-5 | mitochondrial	486
Light-1	435	Background	AT5G09590	Symbols: MTHSC70-2, HSC70-5 | mitochondrial	486
Light-1	435	Background	AT5G09590	Symbols: MTHSC70-2, HSC70-5 | mitochondrial	486
Light-1	435	Background	AT5G09590	Symbols: MTHSC70-2, HSC70-5 | mitochondrial	486
Light-1	435	Background	AT5G09590	Symbols: MTHSC70-2, HSC70-5 | mitochondrial	486
Light-1	435	Background	AT5G09590	Symbols: MTHSC70-2, HSC70-5 | mitochondrial	486
Light-1	435	Background	AT5G09590	Symbols: MTHSC70-2, HSC70-5 | mitochondrial	486
Light-1	436	Background	AT5G35360	Symbols: CAC2 | acetyl Co-enzyme a carboxylas	480
Light-1	436	Background	AT5G35360	Symbols: CAC2 | acetyl Co-enzyme a carboxylas	480
Light-1	436	Background	AT5G35360	Symbols: CAC2 | acetyl Co-enzyme a carboxylas	480
Light-1	436	Background	AT5G35360	Symbols: CAC2 | acetyl Co-enzyme a carboxylas	480
Light-1	436	Background	AT5G35360	Symbols: CAC2 | acetyl Co-enzyme a carboxylas	480
Light-1	436	Background	AT5G35360	Symbols: CAC2 | acetyl Co-enzyme a carboxylas	480
Light-1	436	Background	AT5G35360	Symbols: CAC2 | acetyl Co-enzyme a carboxylas	480
Light-1	436	Background	AT5G35360	Symbols: CAC2 | acetyl Co-enzyme a carboxylas	480
Light-1	436	Background	AT5G35360	Symbols: CAC2 | acetyl Co-enzyme a carboxylas	480
Light-1	436	Background	AT5G35360	Symbols: CAC2 | acetyl Co-enzyme a carboxylas	480
Light-1	436	Background	AT5G35360	Symbols: CAC2 | acetyl Co-enzyme a carboxylas	480
Light-1	437	Background	AT1G56070	Symbols: LOS1 | Ribosomal protein S5/Elongatic	474
Light-1	437	Background	AT1G56070	Symbols: LOS1 | Ribosomal protein S5/Elongatic	474
Light-1	437	Background	AT1G56070	Symbols: LOS1 | Ribosomal protein S5/Elongatic	474
Light-1	437	Background	AT1G56070	Symbols: LOS1 | Ribosomal protein S5/Elongatic	474
Light-1	437	Background	AT1G56070	Symbols: LOS1 | Ribosomal protein S5/Elongatic	474
Light-1	437	Background	AT1G56070	Symbols: LOS1 | Ribosomal protein S5/Elongatic	474
Light-1	437	Background	AT1G56070	Symbols: LOS1 | Ribosomal protein S5/Elongatic	474
Light-1	437	Background	AT1G56070	Symbols: LOS1 | Ribosomal protein S5/Elongatic	474
Light-1	437	Background	AT1G56070	Symbols: LOS1 | Ribosomal protein S5/Elongatic	474
Light-1	438	Background	AT2G24050	Symbols: eIFiso4G2 | MIF4G domain-containing	468
Light-1	438	Background	AT2G24050	Symbols: eIFiso4G2 | MIF4G domain-containing	468
Light-1	438	Background	AT2G24050	Symbols: eIFiso4G2 | MIF4G domain-containing	468

Light-1	438	Background	AT2G24050	Symbols: eIFiso4G2 | MIF4G domain-containing	468
Light-1	438	Background	AT2G24050	Symbols: eIFiso4G2 | MIF4G domain-containing	468
Light-1	438	Background	AT2G24050	Symbols: eIFiso4G2 | MIF4G domain-containing	468
Light-1	438	Background	AT2G24050	Symbols: eIFiso4G2 | MIF4G domain-containing	468
Light-1	438	Background	AT2G24050	Symbols: eIFiso4G2 | MIF4G domain-containing	468
Light-1	439	Table 1 Filtere	AT1G59990	Symbols: | DEA(D/H)-box RNA helicase family p	468
Light-1	439	Table 1 Filtere	AT1G59990	Symbols: | DEA(D/H)-box RNA helicase family p	468
Light-1	439	Table 1 Filtere	AT1G59990	Symbols: | DEA(D/H)-box RNA helicase family p	468
Light-1	439	Table 1 Filtere	AT1G59990	Symbols: | DEA(D/H)-box RNA helicase family p	468
Light-1	439	Table 1 Filtere	AT1G59990	Symbols: | DEA(D/H)-box RNA helicase family p	468
Light-1	439	Table 1 Filtere	AT1G59990	Symbols: | DEA(D/H)-box RNA helicase family p	468
Light-1	440	Background	AT2G20140	Symbols: | AAA-type ATPase family protein | ch	459
Light-1	440	Background	AT2G20140	Symbols: | AAA-type ATPase family protein | ch	459
Light-1	440	Background	AT2G20140	Symbols: | AAA-type ATPase family protein | ch	459
Light-1	440	Background	AT2G20140	Symbols: | AAA-type ATPase family protein | ch	459
Light-1	440	Background	AT2G20140	Symbols: | AAA-type ATPase family protein | ch	459
Light-1	440	Background	AT2G20140	Symbols: | AAA-type ATPase family protein | ch	459
Light-1	440	Background	AT2G20140	Symbols: | AAA-type ATPase family protein | ch	459
Light-1	440	Background	AT2G20140	Symbols: | AAA-type ATPase family protein | ch	459
Light-1	440	Background	AT2G20140	Symbols: | AAA-type ATPase family protein | ch	459
Light-1	441	Background	AT2G28000	Symbols: CPN60A, CH-CPN60A, SLP | chaperonir	430
Light-1	441	Background	AT2G28000	Symbols: CPN60A, CH-CPN60A, SLP | chaperonir	430
Light-1	441	Background	AT2G28000	Symbols: CPN60A, CH-CPN60A, SLP | chaperonir	430
Light-1	441	Background	AT2G28000	Symbols: CPN60A, CH-CPN60A, SLP | chaperonir	430
Light-1	441	Background	AT2G28000	Symbols: CPN60A, CH-CPN60A, SLP | chaperonir	430
Light-1	441	Background	AT2G28000	Symbols: CPN60A, CH-CPN60A, SLP | chaperonir	430
Light-1	441	Background	AT2G28000	Symbols: CPN60A, CH-CPN60A, SLP | chaperonir	430
Light-1	441	Background	AT2G28000	Symbols: CPN60A, CH-CPN60A, SLP | chaperonir	430
Light-1	441	Background	AT2G28000	Symbols: CPN60A, CH-CPN60A, SLP | chaperonir	430
Light-1	442	Background	AT5G09810	Symbols: ACT7 | actin 7 | chr5:3052809-305422	429
Light-1	442	Background	AT5G09810	Symbols: ACT7 | actin 7 | chr5:3052809-305422	429
Light-1	442	Background	AT5G09810	Symbols: ACT7 | actin 7 | chr5:3052809-305422	429
Light-1	442	Background	AT5G09810	Symbols: ACT7 | actin 7 | chr5:3052809-305422	429
Light-1	442	Background	AT5G09810	Symbols: ACT7 | actin 7 | chr5:3052809-305422	429
Light-1	442	Background	AT5G09810	Symbols: ACT7 | actin 7 | chr5:3052809-305422	429
Light-1	442	Background	AT5G09810	Symbols: ACT7 | actin 7 | chr5:3052809-305422	429
Light-1	442	Background	AT5G09810	Symbols: ACT7 | actin 7 | chr5:3052809-305422	429
Light-1	443	Background	AT4G35260	Symbols: IDH1, IDH-I | isocitrate dehydrogenase	428
Light-1	443	Background	AT4G35260	Symbols: IDH1, IDH-I | isocitrate dehydrogenase	428
Light-1	443	Background	AT4G35260	Symbols: IDH1, IDH-I | isocitrate dehydrogenase	428
Light-1	443	Background	AT4G35260	Symbols: IDH1, IDH-I | isocitrate dehydrogenase	428
Light-1	443	Background	AT4G35260	Symbols: IDH1, IDH-I | isocitrate dehydrogenase	428
Light-1	443	Background	AT4G35260	Symbols: IDH1, IDH-I | isocitrate dehydrogenase	428
Light-1	443	Background	AT4G35260	Symbols: IDH1, IDH-I | isocitrate dehydrogenase	428
Light-1	443	Background	AT4G35260	Symbols: IDH1, IDH-I | isocitrate dehydrogenase	428
Light-1	444	Background	AT2G44060	Symbols: | Late embryogenesis abundant prote	424
Light-1	444	Background	AT2G44060	Symbols: | Late embryogenesis abundant prote	424
Light-1	444	Background	AT2G44060	Symbols: | Late embryogenesis abundant prote	424
Light-1	444	Background	AT2G44060	Symbols: | Late embryogenesis abundant prote	424
Light-1	444	Background	AT2G44060	Symbols: | Late embryogenesis abundant prote	424
Light-1	444	Background	AT2G44060	Symbols: | Late embryogenesis abundant prote	424
Light-1	444	Background	AT2G44060	Symbols: | Late embryogenesis abundant prote	424
Light-1	444	Background	AT2G44060	Symbols: | Late embryogenesis abundant prote	424
Light-1	444	Background	AT2G44060	Symbols: | Late embryogenesis abundant prote	424
Light-1	445	Background	AT3G22310	Symbols: PMH1, ATRH9 | putative mitochondria	411
Light-1	445	Background	AT3G22310	Symbols: PMH1, ATRH9 | putative mitochondria	411
Light-1	445	Background	AT3G22310	Symbols: PMH1, ATRH9 | putative mitochondria	411
Light-1	445	Background	AT3G22310	Symbols: PMH1, ATRH9 | putative mitochondria	411

Light-1	445	Background	AT3G22310	Symbols: PMH1, ATRH9 | putative mitochondria	411
Light-1	445	Background	AT3G22310	Symbols: PMH1, ATRH9 | putative mitochondria	411
Light-1	445	Background	AT3G22310	Symbols: PMH1, ATRH9 | putative mitochondria	411
Light-1	445	Background	AT3G22310	Symbols: PMH1, ATRH9 | putative mitochondria	411
Light-1	445	Background	AT3G22310	Symbols: PMH1, ATRH9 | putative mitochondria	411
Light-1	445	Background	AT3G22310	Symbols: PMH1, ATRH9 | putative mitochondria	411
Light-1	445	Background	AT3G22310	Symbols: PMH1, ATRH9 | putative mitochondria	411
Light-1	446	Background	AT5G23540	Symbols: | Mov34/MPN/PAD-1 family protein |	399
Light-1	446	Background	AT5G23540	Symbols: | Mov34/MPN/PAD-1 family protein |	399
Light-1	446	Background	AT5G23540	Symbols: | Mov34/MPN/PAD-1 family protein |	399
Light-1	446	Background	AT5G23540	Symbols: | Mov34/MPN/PAD-1 family protein |	399
Light-1	446	Background	AT5G23540	Symbols: | Mov34/MPN/PAD-1 family protein |	399
Light-1	446	Background	AT5G23540	Symbols: | Mov34/MPN/PAD-1 family protein |	399
Light-1	446	Background	AT5G23540	Symbols: | Mov34/MPN/PAD-1 family protein |	399
Light-1	447	Table 1 Filtere	AT4G02380	Symbols: SAG21, AtLEA5 | senescence-associate	390
Light-1	447	Table 1 Filtere	AT4G02380	Symbols: SAG21, AtLEA5 | senescence-associate	390
Light-1	447	Table 1 Filtere	AT4G02380	Symbols: SAG21, AtLEA5 | senescence-associate	390
Light-1	447	Table 1 Filtere	AT4G02380	Symbols: SAG21, AtLEA5 | senescence-associate	390
Light-1	447	Table 1 Filtere	AT4G02380	Symbols: SAG21, AtLEA5 | senescence-associate	390
Light-1	448	Background	AT3G52140	Symbols: | tetratricopeptide repeat (TPR)-conta	389
Light-1	448	Background	AT3G52140	Symbols: | tetratricopeptide repeat (TPR)-conta	389
Light-1	448	Background	AT3G52140	Symbols: | tetratricopeptide repeat (TPR)-conta	389
Light-1	448	Background	AT3G52140	Symbols: | tetratricopeptide repeat (TPR)-conta	389
Light-1	448	Background	AT3G52140	Symbols: | tetratricopeptide repeat (TPR)-conta	389
Light-1	448	Background	AT3G52140	Symbols: | tetratricopeptide repeat (TPR)-conta	389
Light-1	449	Background	AT5G19990	Symbols: RPT6A, ATSUG1 | regulatory particle tr	383
Light-1	449	Background	AT5G19990	Symbols: RPT6A, ATSUG1 | regulatory particle tr	383
Light-1	449	Background	AT5G19990	Symbols: RPT6A, ATSUG1 | regulatory particle tr	383
Light-1	449	Background	AT5G19990	Symbols: RPT6A, ATSUG1 | regulatory particle tr	383
Light-1	449	Background	AT5G19990	Symbols: RPT6A, ATSUG1 | regulatory particle tr	383
Light-1	450	Background	AT5G24710	Symbols: | Transducin/WD40 repeat-like superf	376
Light-1	450	Background	AT5G24710	Symbols: | Transducin/WD40 repeat-like superf	376
Light-1	450	Background	AT5G24710	Symbols: | Transducin/WD40 repeat-like superf	376
Light-1	450	Background	AT5G24710	Symbols: | Transducin/WD40 repeat-like superf	376
Light-1	451	Background	AT3G43300	Symbols: ATMIN7, BEN1 | HOPM interactor 7 | (	352
Light-1	451	Background	AT3G43300	Symbols: ATMIN7, BEN1 | HOPM interactor 7 | (	352
Light-1	451	Background	AT3G43300	Symbols: ATMIN7, BEN1 | HOPM interactor 7 | (	352
Light-1	451	Background	AT3G43300	Symbols: ATMIN7, BEN1 | HOPM interactor 7 | (	352
Light-1	451	Background	AT3G43300	Symbols: ATMIN7, BEN1 | HOPM interactor 7 | (	352
Light-1	451	Background	AT3G43300	Symbols: ATMIN7, BEN1 | HOPM interactor 7 | (	352
Light-1	451	Background	AT3G43300	Symbols: ATMIN7, BEN1 | HOPM interactor 7 | (	352
Light-1	451	Background	AT3G43300	Symbols: ATMIN7, BEN1 | HOPM interactor 7 | (	352
Light-1	451	Background	AT3G43300	Symbols: ATMIN7, BEN1 | HOPM interactor 7 | (	352
Light-1	451	Background	AT3G43300	Symbols: ATMIN7, BEN1 | HOPM interactor 7 | (	352
Light-1	452	Background	AT3G08580	Symbols: AAC1 | ADP/ATP carrier 1 | chr3:2605	351
Light-1	452	Background	AT3G08580	Symbols: AAC1 | ADP/ATP carrier 1 | chr3:2605	351
Light-1	452	Background	AT3G08580	Symbols: AAC1 | ADP/ATP carrier 1 | chr3:2605	351
Light-1	452	Background	AT3G08580	Symbols: AAC1 | ADP/ATP carrier 1 | chr3:2605	351
Light-1	452	Background	AT3G08580	Symbols: AAC1 | ADP/ATP carrier 1 | chr3:2605	351
Light-1	452	Background	AT3G08580	Symbols: AAC1 | ADP/ATP carrier 1 | chr3:2605	351
Light-1	452	Background	AT3G08580	Symbols: AAC1 | ADP/ATP carrier 1 | chr3:2605	351
Light-1	452	Background	AT3G08580	Symbols: AAC1 | ADP/ATP carrier 1 | chr3:2605	351
Light-1	453	Background	AT2G15860	Symbols: | unknown protein; FUNCTIONS IN: m	331
Light-1	453	Background	AT2G15860	Symbols: | unknown protein; FUNCTIONS IN: m	331
Light-1	453	Background	AT2G15860	Symbols: | unknown protein; FUNCTIONS IN: m	331
Light-1	453	Background	AT2G15860	Symbols: | unknown protein; FUNCTIONS IN: m	331
Light-1	453	Background	AT2G15860	Symbols: | unknown protein; FUNCTIONS IN: m	331
Light-1	453	Background	AT2G15860	Symbols: | unknown protein; FUNCTIONS IN: m	331

Light-1	454	Background	AT1G52670	Symbols: | Single hybrid motif superfamily prot	328
Light-1	454	Background	AT1G52670	Symbols: | Single hybrid motif superfamily prot	328
Light-1	454	Background	AT1G52670	Symbols: | Single hybrid motif superfamily prot	328
Light-1	454	Background	AT1G52670	Symbols: | Single hybrid motif superfamily prot	328
Light-1	455	Background	AT4G02930	Symbols: | GTP binding Elongation factor Tu fan	324
Light-1	455	Background	AT4G02930	Symbols: | GTP binding Elongation factor Tu fan	324
Light-1	455	Background	AT4G02930	Symbols: | GTP binding Elongation factor Tu fan	324
Light-1	455	Background	AT4G02930	Symbols: | GTP binding Elongation factor Tu fan	324
Light-1	455	Background	AT4G02930	Symbols: | GTP binding Elongation factor Tu fan	324
Light-1	456	Background	AT5G28540	Symbols: BIP1 | heat shock protein 70 (Hsp 70) f	323
Light-1	456	Background	AT5G28540	Symbols: BIP1 | heat shock protein 70 (Hsp 70) f	323
Light-1	456	Background	AT5G28540	Symbols: BIP1 | heat shock protein 70 (Hsp 70) f	323
Light-1	456	Background	AT5G28540	Symbols: BIP1 | heat shock protein 70 (Hsp 70) f	323
Light-1	456	Background	AT5G28540	Symbols: BIP1 | heat shock protein 70 (Hsp 70) f	323
Light-1	456	Background	AT5G28540	Symbols: BIP1 | heat shock protein 70 (Hsp 70) f	323
Light-1	456	Background	AT5G28540	Symbols: BIP1 | heat shock protein 70 (Hsp 70) f	323
Light-1	457	Background	AT4G16143	Symbols: IMPA-2 | importin alpha isoform 2 | ch	322
Light-1	457	Background	AT4G16143	Symbols: IMPA-2 | importin alpha isoform 2 | ch	322
Light-1	457	Background	AT4G16143	Symbols: IMPA-2 | importin alpha isoform 2 | ch	322
Light-1	457	Background	AT4G16143	Symbols: IMPA-2 | importin alpha isoform 2 | ch	322
Light-1	457	Background	AT4G16143	Symbols: IMPA-2 | importin alpha isoform 2 | ch	322
Light-1	457	Background	AT4G16143	Symbols: IMPA-2 | importin alpha isoform 2 | ch	322
Light-1	457	Background	AT4G16143	Symbols: IMPA-2 | importin alpha isoform 2 | ch	322
Light-1	457	Background	AT4G16143	Symbols: IMPA-2 | importin alpha isoform 2 | ch	322
Light-1	457	Background	AT4G16143	Symbols: IMPA-2 | importin alpha isoform 2 | ch	322
Light-1	457	Background	AT4G16143	Symbols: IMPA-2 | importin alpha isoform 2 | ch	322
Light-1	458	Background	AT3G17390	Symbols: MTO3, SAMS3, MAT4 | S-adenosylmet	321
Light-1	458	Background	AT3G17390	Symbols: MTO3, SAMS3, MAT4 | S-adenosylmet	321
Light-1	458	Background	AT3G17390	Symbols: MTO3, SAMS3, MAT4 | S-adenosylmet	321
Light-1	458	Background	AT3G17390	Symbols: MTO3, SAMS3, MAT4 | S-adenosylmet	321
Light-1	458	Background	AT3G17390	Symbols: MTO3, SAMS3, MAT4 | S-adenosylmet	321
Light-1	458	Background	AT3G17390	Symbols: MTO3, SAMS3, MAT4 | S-adenosylmet	321
Light-1	458	Background	AT3G17390	Symbols: MTO3, SAMS3, MAT4 | S-adenosylmet	321
Light-1	458	Background	AT3G17390	Symbols: MTO3, SAMS3, MAT4 | S-adenosylmet	321
Light-1	459	Background	AT1G56110	Symbols: NOP56 | homolog of nucleolar protein	320
Light-1	459	Background	AT1G56110	Symbols: NOP56 | homolog of nucleolar protein	320
Light-1	459	Background	AT1G56110	Symbols: NOP56 | homolog of nucleolar protein	320
Light-1	459	Background	AT1G56110	Symbols: NOP56 | homolog of nucleolar protein	320
Light-1	459	Background	AT1G56110	Symbols: NOP56 | homolog of nucleolar protein	320
Light-1	459	Background	AT1G56110	Symbols: NOP56 | homolog of nucleolar protein	320
Light-1	459	Background	AT1G56110	Symbols: NOP56 | homolog of nucleolar protein	320
Light-1	459	Background	AT1G56110	Symbols: NOP56 | homolog of nucleolar protein	320
Light-1	460	Background	AT3G06720	Symbols: AT-IMP, ATKAP ALPHA, AIMP ALPHA, II	317
Light-1	460	Background	AT3G06720	Symbols: AT-IMP, ATKAP ALPHA, AIMP ALPHA, II	317
Light-1	460	Background	AT3G06720	Symbols: AT-IMP, ATKAP ALPHA, AIMP ALPHA, II	317
Light-1	460	Background	AT3G06720	Symbols: AT-IMP, ATKAP ALPHA, AIMP ALPHA, II	317
Light-1	460	Background	AT3G06720	Symbols: AT-IMP, ATKAP ALPHA, AIMP ALPHA, II	317
Light-1	460	Background	AT3G06720	Symbols: AT-IMP, ATKAP ALPHA, AIMP ALPHA, II	317
Light-1	460	Background	AT3G06720	Symbols: AT-IMP, ATKAP ALPHA, AIMP ALPHA, II	317
Light-1	461	Background	AT2G07698	Symbols: | ATPase, F1 complex, alpha subunit p	310
Light-1	461	Background	AT2G07698	Symbols: | ATPase, F1 complex, alpha subunit p	310
Light-1	461	Background	AT2G07698	Symbols: | ATPase, F1 complex, alpha subunit p	310
Light-1	461	Background	AT2G07698	Symbols: | ATPase, F1 complex, alpha subunit p	310
Light-1	461	Background	AT2G07698	Symbols: | ATPase, F1 complex, alpha subunit p	310
Light-1	461	Background	AT2G07698	Symbols: | ATPase, F1 complex, alpha subunit p	310
Light-1	462	Background	AT2G20800	Symbols: NDB4 | NAD(P)H dehydrogenase B4 | (	302
Light-1	462	Background	AT2G20800	Symbols: NDB4 | NAD(P)H dehydrogenase B4 | (	302
Light-1	462	Background	AT2G20800	Symbols: NDB4 | NAD(P)H dehydrogenase B4 | (	302

Light-1	462	Background	AT2G20800	Symbols: NDB4 | NAD(P)H dehydrogenase B4 |	302
Light-1	462	Background	AT2G20800	Symbols: NDB4 | NAD(P)H dehydrogenase B4 |	302
Light-1	462	Background	AT2G20800	Symbols: NDB4 | NAD(P)H dehydrogenase B4 |	302
Light-1	463	Background	AT2G37620	Symbols: ACT1, AAc1 | actin 1 | chr2:15779761-	301
Light-1	463	Background	AT2G37620	Symbols: ACT1, AAc1 | actin 1 | chr2:15779761-	301
Light-1	463	Background	AT2G37620	Symbols: ACT1, AAc1 | actin 1 | chr2:15779761-	301
Light-1	463	Background	AT2G37620	Symbols: ACT1, AAc1 | actin 1 | chr2:15779761-	301
Light-1	463	Background	AT2G37620	Symbols: ACT1, AAc1 | actin 1 | chr2:15779761-	301
Light-1	463	Background	AT2G37620	Symbols: ACT1, AAc1 | actin 1 | chr2:15779761-	301
Light-1	464	Background	AT1G53750	Symbols: RPT1A | regulatory particle triple-A 1A	295
Light-1	464	Background	AT1G53750	Symbols: RPT1A | regulatory particle triple-A 1A	295
Light-1	464	Background	AT1G53750	Symbols: RPT1A | regulatory particle triple-A 1A	295
Light-1	464	Background	AT1G53750	Symbols: RPT1A | regulatory particle triple-A 1A	295
Light-1	464	Background	AT1G53750	Symbols: RPT1A | regulatory particle triple-A 1A	295
Light-1	465	Background	AT2G21390	Symbols: | Coatomer, alpha subunit | chr2:915	292
Light-1	465	Background	AT2G21390	Symbols: | Coatomer, alpha subunit | chr2:915	292
Light-1	465	Background	AT2G21390	Symbols: | Coatomer, alpha subunit | chr2:915	292
Light-1	465	Background	AT2G21390	Symbols: | Coatomer, alpha subunit | chr2:915	292
Light-1	465	Background	AT2G21390	Symbols: | Coatomer, alpha subunit | chr2:915	292
Light-1	465	Background	AT2G21390	Symbols: | Coatomer, alpha subunit | chr2:915	292
Light-1	465	Background	AT2G21390	Symbols: | Coatomer, alpha subunit | chr2:915	292
Light-1	465	Background	AT2G21390	Symbols: | Coatomer, alpha subunit | chr2:915	292
Light-1	466	Background	AT2G16950	Symbols: TRN1, ATTRN1 | transportin 1 | chr2:7	291
Light-1	466	Background	AT2G16950	Symbols: TRN1, ATTRN1 | transportin 1 | chr2:7	291
Light-1	466	Background	AT2G16950	Symbols: TRN1, ATTRN1 | transportin 1 | chr2:7	291
Light-1	466	Background	AT2G16950	Symbols: TRN1, ATTRN1 | transportin 1 | chr2:7	291
Light-1	466	Background	AT2G16950	Symbols: TRN1, ATTRN1 | transportin 1 | chr2:7	291
Light-1	466	Background	AT2G16950	Symbols: TRN1, ATTRN1 | transportin 1 | chr2:7	291
Light-1	466	Background	AT2G16950	Symbols: TRN1, ATTRN1 | transportin 1 | chr2:7	291
Light-1	466	Background	AT2G16950	Symbols: TRN1, ATTRN1 | transportin 1 | chr2:7	291
Light-1	466	Background	AT2G16950	Symbols: TRN1, ATTRN1 | transportin 1 | chr2:7	291
Light-1	467	Background	AT3G63460	Symbols: | transducin family protein / WD-40 re	287
Light-1	467	Background	AT3G63460	Symbols: | transducin family protein / WD-40 re	287
Light-1	467	Background	AT3G63460	Symbols: | transducin family protein / WD-40 re	287
Light-1	467	Background	AT3G63460	Symbols: | transducin family protein / WD-40 re	287
Light-1	467	Background	AT3G63460	Symbols: | transducin family protein / WD-40 re	287
Light-1	467	Background	AT3G63460	Symbols: | transducin family protein / WD-40 re	287
Light-1	467	Background	AT3G63460	Symbols: | transducin family protein / WD-40 re	287
Light-1	468	Background	AT1G55490	Symbols: CPN60B, LEN1 | chaperonin 60 beta |	285
Light-1	468	Background	AT1G55490	Symbols: CPN60B, LEN1 | chaperonin 60 beta |	285
Light-1	468	Background	AT1G55490	Symbols: CPN60B, LEN1 | chaperonin 60 beta |	285
Light-1	468	Background	AT1G55490	Symbols: CPN60B, LEN1 | chaperonin 60 beta |	285
Light-1	468	Background	AT1G55490	Symbols: CPN60B, LEN1 | chaperonin 60 beta |	285
Light-1	468	Background	AT1G55490	Symbols: CPN60B, LEN1 | chaperonin 60 beta |	285
Light-1	469	Background	AT2G17130	Symbols: IDH2, IDH-II | isocitrate dehydrogenase	280
Light-1	469	Background	AT2G17130	Symbols: IDH2, IDH-II | isocitrate dehydrogenase	280
Light-1	469	Background	AT2G17130	Symbols: IDH2, IDH-II | isocitrate dehydrogenase	280
Light-1	469	Background	AT2G17130	Symbols: IDH2, IDH-II | isocitrate dehydrogenase	280
Light-1	469	Background	AT2G17130	Symbols: IDH2, IDH-II | isocitrate dehydrogenase	280
Light-1	469	Background	AT2G17130	Symbols: IDH2, IDH-II | isocitrate dehydrogenase	280
Light-1	470	Background	AT1G43800	Symbols: | Plant stearoyl-acyl-carrier-protein de	275
Light-1	470	Background	AT1G43800	Symbols: | Plant stearoyl-acyl-carrier-protein de	275
Light-1	470	Background	AT1G43800	Symbols: | Plant stearoyl-acyl-carrier-protein de	275
Light-1	470	Background	AT1G43800	Symbols: | Plant stearoyl-acyl-carrier-protein de	275
Light-1	470	Background	AT1G43800	Symbols: | Plant stearoyl-acyl-carrier-protein de	275
Light-1	471	Background	AT3G07050	Symbols: | GTP-binding family protein | chr3:22	267
Light-1	471	Background	AT3G07050	Symbols: | GTP-binding family protein | chr3:22	267
Light-1	471	Background	AT3G07050	Symbols: | GTP-binding family protein | chr3:22	267

Light-1	471	Background	AT3G07050	Symbols: | GTP-binding family protein | chr3:22	267
Light-1	472	Background	AT3G11130	Symbols: | Clathrin, heavy chain | chr3:3482575	264
Light-1	472	Background	AT3G11130	Symbols: | Clathrin, heavy chain | chr3:3482575	264
Light-1	472	Background	AT3G11130	Symbols: | Clathrin, heavy chain | chr3:3482575	264
Light-1	472	Background	AT3G11130	Symbols: | Clathrin, heavy chain | chr3:3482575	264
Light-1	472	Background	AT3G11130	Symbols: | Clathrin, heavy chain | chr3:3482575	264
Light-1	472	Background	AT3G11130	Symbols: | Clathrin, heavy chain | chr3:3482575	264
Light-1	472	Background	AT3G11130	Symbols: | Clathrin, heavy chain | chr3:3482575	264
Light-1	473	Background	AT5G08670	Symbols: | ATP synthase alpha/beta family prot	264
Light-1	473	Background	AT5G08670	Symbols: | ATP synthase alpha/beta family prot	264
Light-1	473	Background	AT5G08670	Symbols: | ATP synthase alpha/beta family prot	264
Light-1	473	Background	AT5G08670	Symbols: | ATP synthase alpha/beta family prot	264
Light-1	473	Background	AT5G08670	Symbols: | ATP synthase alpha/beta family prot	264
Light-1	473	Background	AT5G08670	Symbols: | ATP synthase alpha/beta family prot	264
Light-1	473	Background	AT5G08670	Symbols: | ATP synthase alpha/beta family prot	264
Light-1	473	Background	AT5G08670	Symbols: | ATP synthase alpha/beta family prot	264
Light-1	474	Background	AT1G22780	Symbols: PFL, RPS18A, PFL1 | Ribosomal protein	260
Light-1	474	Background	AT1G22780	Symbols: PFL, RPS18A, PFL1 | Ribosomal protein	260
Light-1	475	Background	AT3G02530	Symbols: | TCP-1/cpn60 chaperonin family prot	259
Light-1	475	Background	AT3G02530	Symbols: | TCP-1/cpn60 chaperonin family prot	259
Light-1	475	Background	AT3G02530	Symbols: | TCP-1/cpn60 chaperonin family prot	259
Light-1	475	Background	AT3G02530	Symbols: | TCP-1/cpn60 chaperonin family prot	259
Light-1	476	Background	AT1G49240	Symbols: ACT8 | actin 8 | chr1:18216539-18217	257
Light-1	476	Background	AT1G49240	Symbols: ACT8 | actin 8 | chr1:18216539-18217	257
Light-1	476	Background	AT1G49240	Symbols: ACT8 | actin 8 | chr1:18216539-18217	257
Light-1	476	Background	AT1G49240	Symbols: ACT8 | actin 8 | chr1:18216539-18217	257
Light-1	476	Background	AT1G49240	Symbols: ACT8 | actin 8 | chr1:18216539-18217	257
Light-1	477	Background	AT2G37270	Symbols: ATRPS5B, RPS5B | ribosomal protein 5	252
Light-1	477	Background	AT2G37270	Symbols: ATRPS5B, RPS5B | ribosomal protein 5	252
Light-1	477	Background	AT2G37270	Symbols: ATRPS5B, RPS5B | ribosomal protein 5	252
Light-1	477	Background	AT2G37270	Symbols: ATRPS5B, RPS5B | ribosomal protein 5	252
Light-1	477	Background	AT2G37270	Symbols: ATRPS5B, RPS5B | ribosomal protein 5	252
Light-1	478	Background	AT3G08530	Symbols: | Clathrin, heavy chain | chr3:2587171	248
Light-1	478	Background	AT3G08530	Symbols: | Clathrin, heavy chain | chr3:2587171	248
Light-1	478	Background	AT3G08530	Symbols: | Clathrin, heavy chain | chr3:2587171	248
Light-1	478	Background	AT3G08530	Symbols: | Clathrin, heavy chain | chr3:2587171	248
Light-1	478	Background	AT3G08530	Symbols: | Clathrin, heavy chain | chr3:2587171	248
Light-1	478	Background	AT3G08530	Symbols: | Clathrin, heavy chain | chr3:2587171	248
Light-1	479	Background	AT3G57290	Symbols: EIF3E, TIF3E1, ATEIF3E-1, INT-6, ATINT	246
Light-1	479	Background	AT3G57290	Symbols: EIF3E, TIF3E1, ATEIF3E-1, INT-6, ATINT	246
Light-1	479	Background	AT3G57290	Symbols: EIF3E, TIF3E1, ATEIF3E-1, INT-6, ATINT	246
Light-1	480	Background	AT5G56500	Symbols: | TCP-1/cpn60 chaperonin family prot	246
Light-1	480	Background	AT5G56500	Symbols: | TCP-1/cpn60 chaperonin family prot	246
Light-1	480	Background	AT5G56500	Symbols: | TCP-1/cpn60 chaperonin family prot	246
Light-1	480	Background	AT5G56500	Symbols: | TCP-1/cpn60 chaperonin family prot	246
Light-1	480	Background	AT5G56500	Symbols: | TCP-1/cpn60 chaperonin family prot	246
Light-1	481	Background	AT5G26860	Symbols: LON_ARA_ARA, LON1 | lon protease 1	245
Light-1	481	Background	AT5G26860	Symbols: LON_ARA_ARA, LON1 | lon protease 1	245
Light-1	481	Background	AT5G26860	Symbols: LON_ARA_ARA, LON1 | lon protease 1	245
Light-1	481	Background	AT5G26860	Symbols: LON_ARA_ARA, LON1 | lon protease 1	245
Light-1	481	Background	AT5G26860	Symbols: LON_ARA_ARA, LON1 | lon protease 1	245
Light-1	482	Background	AT5G13490	Symbols: AAC2 | ADP/ATP carrier 2 | chr5:43360	245
Light-1	482	Background	AT5G13490	Symbols: AAC2 | ADP/ATP carrier 2 | chr5:43360	245
Light-1	482	Background	AT5G13490	Symbols: AAC2 | ADP/ATP carrier 2 | chr5:43360	245
Light-1	482	Background	AT5G13490	Symbols: AAC2 | ADP/ATP carrier 2 | chr5:43360	245
Light-1	482	Background	AT5G13490	Symbols: AAC2 | ADP/ATP carrier 2 | chr5:43360	245
Light-1	482	Background	AT5G13490	Symbols: AAC2 | ADP/ATP carrier 2 | chr5:43360	245
Light-1	482	Background	AT5G13490	Symbols: AAC2 | ADP/ATP carrier 2 | chr5:43360	245

Light-1	483	Background	AT5G18700	Symbols: RUK, EMB3013 | Protein kinase family	240
Light-1	483	Background	AT5G18700	Symbols: RUK, EMB3013 | Protein kinase family	240
Light-1	483	Background	AT5G18700	Symbols: RUK, EMB3013 | Protein kinase family	240
Light-1	483	Background	AT5G18700	Symbols: RUK, EMB3013 | Protein kinase family	240
Light-1	483	Background	AT5G18700	Symbols: RUK, EMB3013 | Protein kinase family	240
Light-1	484	Background	AT3G13300	Symbols: VCS | Transducin/WD40 repeat-like su	234
Light-1	484	Background	AT3G13300	Symbols: VCS | Transducin/WD40 repeat-like su	234
Light-1	484	Background	AT3G13300	Symbols: VCS | Transducin/WD40 repeat-like su	234
Light-1	484	Background	AT3G13300	Symbols: VCS | Transducin/WD40 repeat-like su	234
Light-1	484	Background	AT3G13300	Symbols: VCS | Transducin/WD40 repeat-like su	234
Light-1	484	Background	AT3G13300	Symbols: VCS | Transducin/WD40 repeat-like su	234
Light-1	484	Background	AT3G13300	Symbols: VCS | Transducin/WD40 repeat-like su	234
Light-1	484	Background	AT3G13300	Symbols: VCS | Transducin/WD40 repeat-like su	234
Light-1	484	Background	AT3G13300	Symbols: VCS | Transducin/WD40 repeat-like su	234
Light-1	485	Background	AT5G08610	Symbols: | P-loop containing nucleoside triphos	226
Light-1	485	Background	AT5G08610	Symbols: | P-loop containing nucleoside triphos	226
Light-1	485	Background	AT5G08610	Symbols: | P-loop containing nucleoside triphos	226
Light-1	485	Background	AT5G08610	Symbols: | P-loop containing nucleoside triphos	226
Light-1	485	Background	AT5G08610	Symbols: | P-loop containing nucleoside triphos	226
Light-1	486	Table 1 Filtere	AT2G36200	Symbols: | P-loop containing nucleoside triphos	226
Light-1	486	Table 1 Filtere	AT2G36200	Symbols: | P-loop containing nucleoside triphos	226
Light-1	486	Table 1 Filtere	AT2G36200	Symbols: | P-loop containing nucleoside triphos	226
Light-1	486	Table 1 Filtere	AT2G36200	Symbols: | P-loop containing nucleoside triphos	226
Light-1	486	Table 1 Filtere	AT2G36200	Symbols: | P-loop containing nucleoside triphos	226
Light-1	486	Table 1 Filtere	AT2G36200	Symbols: | P-loop containing nucleoside triphos	226
Light-1	487	Background	AT1G79530	Symbols: GAPCP-1 | glyceraldehyde-3-phosphat	222
Light-1	487	Background	AT1G79530	Symbols: GAPCP-1 | glyceraldehyde-3-phosphat	222
Light-1	487	Background	AT1G79530	Symbols: GAPCP-1 | glyceraldehyde-3-phosphat	222
Light-1	488	Background	AT5G26742	Symbols: emb1138 | DEAD box RNA helicase (RH	219
Light-1	488	Background	AT5G26742	Symbols: emb1138 | DEAD box RNA helicase (RH	219
Light-1	488	Background	AT5G26742	Symbols: emb1138 | DEAD box RNA helicase (RH	219
Light-1	488	Background	AT5G26742	Symbols: emb1138 | DEAD box RNA helicase (RH	219
Light-1	488	Background	AT5G26742	Symbols: emb1138 | DEAD box RNA helicase (RH	219
Light-1	488	Background	AT5G26742	Symbols: emb1138 | DEAD box RNA helicase (RH	219
Light-1	488	Background	AT5G26742	Symbols: emb1138 | DEAD box RNA helicase (RH	219
Light-1	488	Background	AT5G26742	Symbols: emb1138 | DEAD box RNA helicase (RH	219
Light-1	488	Background	AT5G26742	Symbols: emb1138 | DEAD box RNA helicase (RH	219
Light-1	489	Background	AT1G29150	Symbols: ATS9, RPN6 | non-ATPase subunit 9 | c	218
Light-1	489	Background	AT1G29150	Symbols: ATS9, RPN6 | non-ATPase subunit 9 | c	218
Light-1	489	Background	AT1G29150	Symbols: ATS9, RPN6 | non-ATPase subunit 9 | c	218
Light-1	489	Background	AT1G29150	Symbols: ATS9, RPN6 | non-ATPase subunit 9 | c	218
Light-1	490	Background	AT1G24510	Symbols: | TCP-1/cpn60 chaperonin family prot	213
Light-1	490	Background	AT1G24510	Symbols: | TCP-1/cpn60 chaperonin family prot	213
Light-1	490	Background	AT1G24510	Symbols: | TCP-1/cpn60 chaperonin family prot	213
Light-1	490	Background	AT1G24510	Symbols: | TCP-1/cpn60 chaperonin family prot	213
Light-1	491	Table 1 Filtere	AT3G16810	Symbols: APUM24, PUM24 | pumilio 24 | chr3:5	212
Light-1	491	Table 1 Filtere	AT3G16810	Symbols: APUM24, PUM24 | pumilio 24 | chr3:5	212
Light-1	491	Table 1 Filtere	AT3G16810	Symbols: APUM24, PUM24 | pumilio 24 | chr3:5	212
Light-1	492	Background	AT5G58290	Symbols: RPT3 | regulatory particle triple-A ATPa	211
Light-1	492	Background	AT5G58290	Symbols: RPT3 | regulatory particle triple-A ATPa	211
Light-1	492	Background	AT5G58290	Symbols: RPT3 | regulatory particle triple-A ATPa	211
Light-1	492	Background	AT5G58290	Symbols: RPT3 | regulatory particle triple-A ATPa	211
Light-1	492	Background	AT5G58290	Symbols: RPT3 | regulatory particle triple-A ATPa	211
Light-1	492	Background	AT5G58290	Symbols: RPT3 | regulatory particle triple-A ATPa	211
Light-1	493	Background	AT3G11830	Symbols: | TCP-1/cpn60 chaperonin family prot	206
Light-1	493	Background	AT3G11830	Symbols: | TCP-1/cpn60 chaperonin family prot	206
Light-1	493	Background	AT3G11830	Symbols: | TCP-1/cpn60 chaperonin family prot	206
Light-1	494	Background	AT1G59610	Symbols: ADL3, CF1, DRP2B, DL3 | dynamin-like	202

Light-1	494	Background	AT1G59610	Symbols: ADL3, CF1, DRP2B, DL3 | dynamin-like	202
Light-1	494	Background	AT1G59610	Symbols: ADL3, CF1, DRP2B, DL3 | dynamin-like	202
Light-1	494	Background	AT1G59610	Symbols: ADL3, CF1, DRP2B, DL3 | dynamin-like	202
Light-1	495	Background	AT5G12370	Symbols: SEC10 | exocyst complex component s	201
Light-1	495	Background	AT5G12370	Symbols: SEC10 | exocyst complex component s	201
Light-1	495	Background	AT5G12370	Symbols: SEC10 | exocyst complex component s	201
Light-1	495	Background	AT5G12370	Symbols: SEC10 | exocyst complex component s	201
Light-1	495	Background	AT5G12370	Symbols: SEC10 | exocyst complex component s	201
Light-1	495	Background	AT5G12370	Symbols: SEC10 | exocyst complex component s	201
Light-1	496	Background	AT3G57940	Symbols: | Domain of unknown function (DUF1	201
Light-1	496	Background	AT3G57940	Symbols: | Domain of unknown function (DUF1	201
Light-1	496	Background	AT3G57940	Symbols: | Domain of unknown function (DUF1	201
Light-1	496	Background	AT3G57940	Symbols: | Domain of unknown function (DUF1	201
Light-1	497	Background	AT1G01470	Symbols: LEA14, LSR3 | Late embryogenesis abu	197
Light-1	497	Background	AT1G01470	Symbols: LEA14, LSR3 | Late embryogenesis abu	197
Light-1	497	Background	AT1G01470	Symbols: LEA14, LSR3 | Late embryogenesis abu	197
Light-1	498	Background	AT2G14120	Symbols: DRP3B | dynamin related protein | chr	197
Light-1	498	Background	AT2G14120	Symbols: DRP3B | dynamin related protein | chr	197
Light-1	498	Background	AT2G14120	Symbols: DRP3B | dynamin related protein | chr	197
Light-1	498	Background	AT2G14120	Symbols: DRP3B | dynamin related protein | chr	197
Light-1	498	Background	AT2G14120	Symbols: DRP3B | dynamin related protein | chr	197
Light-1	499	Background	AT2G46520	Symbols: | cellular apoptosis susceptibility prote	196
Light-1	499	Background	AT2G46520	Symbols: | cellular apoptosis susceptibility prote	196
Light-1	499	Background	AT2G46520	Symbols: | cellular apoptosis susceptibility prote	196
Light-1	499	Background	AT2G46520	Symbols: | cellular apoptosis susceptibility prote	196
Light-1	499	Background	AT2G46520	Symbols: | cellular apoptosis susceptibility prote	196
Light-1	499	Background	AT2G46520	Symbols: | cellular apoptosis susceptibility prote	196
Light-1	500	Background	AT5G56000	Symbols: Hsp81.4, AtHsp90.4 | HEAT SHOCK PRO	196
Light-1	500	Background	AT5G56000	Symbols: Hsp81.4, AtHsp90.4 | HEAT SHOCK PRO	196
Light-1	500	Background	AT5G56000	Symbols: Hsp81.4, AtHsp90.4 | HEAT SHOCK PRO	196
Light-1	500	Background	AT5G56000	Symbols: Hsp81.4, AtHsp90.4 | HEAT SHOCK PRO	196
Light-1	500	Background	AT5G56000	Symbols: Hsp81.4, AtHsp90.4 | HEAT SHOCK PRO	196
Light-1	500	Background	AT5G56000	Symbols: Hsp81.4, AtHsp90.4 | HEAT SHOCK PRO	196
Light-1	501	Background	AT3G44110	Symbols: ATJ3, ATJ | DNAJ homologue 3 | chr3:1	193
Light-1	501	Background	AT3G44110	Symbols: ATJ3, ATJ | DNAJ homologue 3 | chr3:1	193
Light-1	501	Background	AT3G44110	Symbols: ATJ3, ATJ | DNAJ homologue 3 | chr3:1	193
Light-1	502	Background	AT4G38630	Symbols: RPN10, MCB1, ATMCB1, MBP1 | regula	192
Light-1	502	Background	AT4G38630	Symbols: RPN10, MCB1, ATMCB1, MBP1 | regula	192
Light-1	503	Background	AT4G23460	Symbols: | Adaptin family protein | chr4:122433	192
Light-1	503	Background	AT4G23460	Symbols: | Adaptin family protein | chr4:122433	192
Light-1	503	Background	AT4G23460	Symbols: | Adaptin family protein | chr4:122433	192
Light-1	504	Background	AT2G15620	Symbols: NIR1, NIR, ATHNIR | nitrite reductase 1	190
Light-1	504	Background	AT2G15620	Symbols: NIR1, NIR, ATHNIR | nitrite reductase 1	190
Light-1	504	Background	AT2G15620	Symbols: NIR1, NIR, ATHNIR | nitrite reductase 1	190
Light-1	504	Background	AT2G15620	Symbols: NIR1, NIR, ATHNIR | nitrite reductase 1	190
Light-1	504	Background	AT2G15620	Symbols: NIR1, NIR, ATHNIR | nitrite reductase 1	190
Light-1	505	Background	AT3G20050	Symbols: ATTCP-1, TCP-1 | T-complex protein 1	189
Light-1	505	Background	AT3G20050	Symbols: ATTCP-1, TCP-1 | T-complex protein 1	189
Light-1	505	Background	AT3G20050	Symbols: ATTCP-1, TCP-1 | T-complex protein 1	189
Light-1	505	Background	AT3G20050	Symbols: ATTCP-1, TCP-1 | T-complex protein 1	189
Light-1	506	Background	AT3G14990	Symbols: | Class I glutamine amidotransferase-l	189
Light-1	506	Background	AT3G14990	Symbols: | Class I glutamine amidotransferase-l	189
Light-1	506	Background	AT3G14990	Symbols: | Class I glutamine amidotransferase-l	189
Light-1	506	Background	AT3G14990	Symbols: | Class I glutamine amidotransferase-l	189
Light-1	506	Background	AT3G14990	Symbols: | Class I glutamine amidotransferase-l	189
Light-1	507	Background	AT4G27585	Symbols: | SPFH/Band 7/PHB domain-containin	188
Light-1	507	Background	AT4G27585	Symbols: | SPFH/Band 7/PHB domain-containin	188
Light-1	507	Background	AT4G27585	Symbols: | SPFH/Band 7/PHB domain-containin	188

Light-1	507	Background	AT4G27585	Symbols: | SPFH/Band 7/PHB domain-containin	188
Light-1	507	Background	AT4G27585	Symbols: | SPFH/Band 7/PHB domain-containin	188
Light-1	507	Background	AT4G27585	Symbols: | SPFH/Band 7/PHB domain-containin	188
Light-1	507	Background	AT4G27585	Symbols: | SPFH/Band 7/PHB domain-containin	188
Light-1	508	Background	AT2G18330	Symbols: | AAA-type ATPase family protein | ch	185
Light-1	508	Background	AT2G18330	Symbols: | AAA-type ATPase family protein | ch	185
Light-1	508	Background	AT2G18330	Symbols: | AAA-type ATPase family protein | ch	185
Light-1	508	Background	AT2G18330	Symbols: | AAA-type ATPase family protein | ch	185
Light-1	509	Background	AT1G62020	Symbols: | Coatomer, alpha subunit | chr1:229	181
Light-1	509	Background	AT1G62020	Symbols: | Coatomer, alpha subunit | chr1:229	181
Light-1	509	Background	AT1G62020	Symbols: | Coatomer, alpha subunit | chr1:229	181
Light-1	509	Background	AT1G62020	Symbols: | Coatomer, alpha subunit | chr1:229	181
Light-1	509	Background	AT1G62020	Symbols: | Coatomer, alpha subunit | chr1:229	181
Light-1	510	Background	AT4G11420	Symbols: EIF3A, ATEIF3A-1, EIF3A-1, ATTIF3A1, T	181
Light-1	510	Background	AT4G11420	Symbols: EIF3A, ATEIF3A-1, EIF3A-1, ATTIF3A1, T	181
Light-1	510	Background	AT4G11420	Symbols: EIF3A, ATEIF3A-1, EIF3A-1, ATTIF3A1, T	181
Light-1	510	Background	AT4G11420	Symbols: EIF3A, ATEIF3A-1, EIF3A-1, ATTIF3A1, T	181
Light-1	510	Background	AT4G11420	Symbols: EIF3A, ATEIF3A-1, EIF3A-1, ATTIF3A1, T	181
Light-1	510	Background	AT4G11420	Symbols: EIF3A, ATEIF3A-1, EIF3A-1, ATTIF3A1, T	181
Light-1	510	Background	AT4G11420	Symbols: EIF3A, ATEIF3A-1, EIF3A-1, ATTIF3A1, T	181
Light-1	511	Background	AT4G24820	Symbols: | 26S proteasome, regulatory subunit	181
Light-1	511	Background	AT4G24820	Symbols: | 26S proteasome, regulatory subunit	181
Light-1	511	Background	AT4G24820	Symbols: | 26S proteasome, regulatory subunit	181
Light-1	511	Background	AT4G24820	Symbols: | 26S proteasome, regulatory subunit	181
Light-1	512	Background	AT2G33210	Symbols: HSP60-2 | heat shock protein 60-2 | ch	175
Light-1	512	Background	AT2G33210	Symbols: HSP60-2 | heat shock protein 60-2 | ch	175
Light-1	512	Background	AT2G33210	Symbols: HSP60-2 | heat shock protein 60-2 | ch	175
Light-1	512	Background	AT2G33210	Symbols: HSP60-2 | heat shock protein 60-2 | ch	175
Light-1	513	Background	AT1G02690	Symbols: IMPA-6 | importin alpha isoform 6 | ch	175
Light-1	513	Background	AT1G02690	Symbols: IMPA-6 | importin alpha isoform 6 | ch	175
Light-1	513	Background	AT1G02690	Symbols: IMPA-6 | importin alpha isoform 6 | ch	175
Light-1	513	Background	AT1G02690	Symbols: IMPA-6 | importin alpha isoform 6 | ch	175
Light-1	513	Background	AT1G02690	Symbols: IMPA-6 | importin alpha isoform 6 | ch	175
Light-1	514	Background	AT5G47690	Symbols: | binding | chr5:19317899-19327014	172
Light-1	514	Background	AT5G47690	Symbols: | binding | chr5:19317899-19327014	172
Light-1	514	Background	AT5G47690	Symbols: | binding | chr5:19317899-19327014	172
Light-1	515	Background	AT3G05590	Symbols: RPL18 | ribosomal protein L18 | chr3:1	168
Light-1	515	Background	AT3G05590	Symbols: RPL18 | ribosomal protein L18 | chr3:1	168
Light-1	515	Background	AT3G05590	Symbols: RPL18 | ribosomal protein L18 | chr3:1	168
Light-1	516	Background	AT1G80270	Symbols: PPR596 | PENTATRICOPEPTIDE REPEA	166
Light-1	516	Background	AT1G80270	Symbols: PPR596 | PENTATRICOPEPTIDE REPEA	166
Light-1	516	Background	AT1G80270	Symbols: PPR596 | PENTATRICOPEPTIDE REPEA	166
Light-1	516	Background	AT1G80270	Symbols: PPR596 | PENTATRICOPEPTIDE REPEA	166
Light-1	516	Background	AT1G80270	Symbols: PPR596 | PENTATRICOPEPTIDE REPEA	166
Light-1	517	Background	AT5G53480	Symbols: | ARM repeat superfamily protein | ch	166
Light-1	517	Background	AT5G53480	Symbols: | ARM repeat superfamily protein | ch	166
Light-1	517	Background	AT5G53480	Symbols: | ARM repeat superfamily protein | ch	166
Light-1	517	Background	AT5G53480	Symbols: | ARM repeat superfamily protein | ch	166
Light-1	518	Background	AT4G34450	Symbols: | coatomer gamma-2 subunit, putativ	162
Light-1	518	Background	AT4G34450	Symbols: | coatomer gamma-2 subunit, putativ	162
Light-1	518	Background	AT4G34450	Symbols: | coatomer gamma-2 subunit, putativ	162
Light-1	518	Background	AT4G34450	Symbols: | coatomer gamma-2 subunit, putativ	162
Light-1	519	Background	AT5G20890	Symbols: | TCP-1/cpn60 chaperonin family prot	161
Light-1	519	Background	AT5G20890	Symbols: | TCP-1/cpn60 chaperonin family prot	161
Light-1	519	Background	AT5G20890	Symbols: | TCP-1/cpn60 chaperonin family prot	161
Light-1	519	Background	AT5G20890	Symbols: | TCP-1/cpn60 chaperonin family prot	161
Light-1	520	Background	AT3G13160	Symbols: | Tetratricopeptide repeat (TPR)-like s	160
Light-1	520	Background	AT3G13160	Symbols: | Tetratricopeptide repeat (TPR)-like s	160

Light-1	521	Background	AT4G27640	Symbols: | ARM repeat superfamily protein | ch	160
Light-1	521	Background	AT4G27640	Symbols: | ARM repeat superfamily protein | ch	160
Light-1	522	Background	AT5G62740	Symbols: HIR1, ATHIR1 | SPFH/Band 7/PHB dom	159
Light-1	522	Background	AT5G62740	Symbols: HIR1, ATHIR1 | SPFH/Band 7/PHB dom	159
Light-1	522	Background	AT5G62740	Symbols: HIR1, ATHIR1 | SPFH/Band 7/PHB dom	159
Light-1	522	Background	AT5G62740	Symbols: HIR1, ATHIR1 | SPFH/Band 7/PHB dom	159
Light-1	522	Background	AT5G62740	Symbols: HIR1, ATHIR1 | SPFH/Band 7/PHB dom	159
Light-1	523	Background	AT5G37510	Symbols: EMB1467, CI76 | NADH-ubiquinone de	156
Light-1	523	Background	AT5G37510	Symbols: EMB1467, CI76 | NADH-ubiquinone de	156
Light-1	524	Background	AT3G05060	Symbols: | NOP56-like pre RNA processing ribor	156
Light-1	524	Background	AT3G05060	Symbols: | NOP56-like pre RNA processing ribor	156
Light-1	525	Background	AT1G06220	Symbols: MEE5, CLO, GFA1 | Ribosomal protein	153
Light-1	525	Background	AT1G06220	Symbols: MEE5, CLO, GFA1 | Ribosomal protein	153
Light-1	525	Background	AT1G06220	Symbols: MEE5, CLO, GFA1 | Ribosomal protein	153
Light-1	525	Background	AT1G06220	Symbols: MEE5, CLO, GFA1 | Ribosomal protein	153
Light-1	525	Background	AT1G06220	Symbols: MEE5, CLO, GFA1 | Ribosomal protein	153
Light-1	525	Background	AT1G06220	Symbols: MEE5, CLO, GFA1 | Ribosomal protein	153
Light-1	526	Background	AT1G04270	Symbols: RPS15 | cytosolic ribosomal protein S1	153
Light-1	526	Background	AT1G04270	Symbols: RPS15 | cytosolic ribosomal protein S1	153
Light-1	527	Background	AT3G09630	Symbols: | Ribosomal protein L4/L1 family | chr	152
Light-1	527	Background	AT3G09630	Symbols: | Ribosomal protein L4/L1 family | chr	152
Light-1	527	Background	AT3G09630	Symbols: | Ribosomal protein L4/L1 family | chr	152
Light-1	528	Background	AT3G61650	Symbols: TUBG1 | gamma-tubulin | chr3:22812(	152
Light-1	528	Background	AT3G61650	Symbols: TUBG1 | gamma-tubulin | chr3:22812(	152
Light-1	529	Background	AT5G49910	Symbols: CPHSC70-2EAT SHOCK PROTEIN 70-2, I	151
Light-1	529	Background	AT5G49910	Symbols: CPHSC70-2EAT SHOCK PROTEIN 70-2, I	151
Light-1	529	Background	AT5G49910	Symbols: CPHSC70-2EAT SHOCK PROTEIN 70-2, I	151
Light-1	529	Background	AT5G49910	Symbols: CPHSC70-2EAT SHOCK PROTEIN 70-2, I	151
Light-1	529	Background	AT5G49910	Symbols: CPHSC70-2EAT SHOCK PROTEIN 70-2, I	151
Light-1	530	Background	AT5G19760	Symbols: | Mitochondrial substrate carrier fami	151
Light-1	530	Background	AT5G19760	Symbols: | Mitochondrial substrate carrier fami	151
Light-1	530	Background	AT5G19760	Symbols: | Mitochondrial substrate carrier fami	151
Light-1	531	Background	AT2G03270	Symbols: | DNA-binding protein, putative | chr2	148
Light-1	531	Background	AT2G03270	Symbols: | DNA-binding protein, putative | chr2	148
Light-1	531	Background	AT2G03270	Symbols: | DNA-binding protein, putative | chr2	148
Light-1	532	Background	AT1G69840	Symbols: | SPFH/Band 7/PHB domain-containin	148
Light-1	532	Background	AT1G69840	Symbols: | SPFH/Band 7/PHB domain-containin	148
Light-1	532	Background	AT1G69840	Symbols: | SPFH/Band 7/PHB domain-containin	148
Light-1	532	Background	AT1G69840	Symbols: | SPFH/Band 7/PHB domain-containin	148
Light-1	533	Background	AT5G17020	Symbols: XPO1A, ATCRM1, ATXPO1, XPO1, HIT2	146
Light-1	533	Background	AT5G17020	Symbols: XPO1A, ATCRM1, ATXPO1, XPO1, HIT2	146
Light-1	533	Background	AT5G17020	Symbols: XPO1A, ATCRM1, ATXPO1, XPO1, HIT2	146
Light-1	533	Background	AT5G17020	Symbols: XPO1A, ATCRM1, ATXPO1, XPO1, HIT2	146
Light-1	533	Background	AT5G17020	Symbols: XPO1A, ATCRM1, ATXPO1, XPO1, HIT2	146
Light-1	534	Background	AT1G02080	Symbols: | transcription regulators | chr1:3733:	146
Light-1	534	Background	AT1G02080	Symbols: | transcription regulators | chr1:3733:	146
Light-1	534	Background	AT1G02080	Symbols: | transcription regulators | chr1:3733:	146
Light-1	534	Background	AT1G02080	Symbols: | transcription regulators | chr1:3733:	146
Light-1	534	Background	AT1G02080	Symbols: | transcription regulators | chr1:3733:	146
Light-1	535	Background	AT1G04810	Symbols: | 26S proteasome regulatory complex	146
Light-1	535	Background	AT1G04810	Symbols: | 26S proteasome regulatory complex	146
Light-1	535	Background	AT1G04810	Symbols: | 26S proteasome regulatory complex	146
Light-1	535	Background	AT1G04810	Symbols: | 26S proteasome regulatory complex	146
Light-1	535	Background	AT1G04810	Symbols: | 26S proteasome regulatory complex	146
Light-1	536	Background	AT1G36180	Symbols: ACC2 | acetyl-CoA carboxylase 2 | chr1	143
Light-1	536	Background	AT1G36180	Symbols: ACC2 | acetyl-CoA carboxylase 2 | chr1	143
Light-1	537	Background	AT5G62190	Symbols: PRH75 | DEAD box RNA helicase (PRH7	143
Light-1	537	Background	AT5G62190	Symbols: PRH75 | DEAD box RNA helicase (PRH7	143

Light-1	537	Background	AT5G62190	Symbols: PRH75 | DEAD box RNA helicase (PRH7	143
Light-1	537	Background	AT5G62190	Symbols: PRH75 | DEAD box RNA helicase (PRH7	143
Light-1	538	Background	AT1G70320	Symbols: UPL2 | ubiquitin-protein ligase 2 | chr1	142
Light-1	538	Background	AT1G70320	Symbols: UPL2 | ubiquitin-protein ligase 2 | chr1	142
Light-1	539	Background	AT3G42170	Symbols: | BED zinc finger ;hAT family dimerisat	142
Light-1	539	Background	AT3G42170	Symbols: | BED zinc finger ;hAT family dimerisat	142
Light-1	540	Background	AT5G36230	Symbols: | ARM repeat superfamily protein | ch	142
Light-1	540	Background	AT5G36230	Symbols: | ARM repeat superfamily protein | ch	142
Light-1	540	Background	AT5G36230	Symbols: | ARM repeat superfamily protein | ch	142
Light-1	541	Background	AT3G10690	Symbols: GYRA | DNA GYRASE A | chr3:3339612	141
Light-1	541	Background	AT3G10690	Symbols: GYRA | DNA GYRASE A | chr3:3339612	141
Light-1	541	Background	AT3G10690	Symbols: GYRA | DNA GYRASE A | chr3:3339612	141
Light-1	542	Background	AT5G05010	Symbols: | clathrin adaptor complexes medium	140
Light-1	542	Background	AT5G05010	Symbols: | clathrin adaptor complexes medium	140
Light-1	543	Background	AT5G16300	Symbols: | Vps51/Vps67 family (components of	138
Light-1	543	Background	AT5G16300	Symbols: | Vps51/Vps67 family (components of	138
Light-1	543	Background	AT5G16300	Symbols: | Vps51/Vps67 family (components of	138
Light-1	544	Background	AT1G23410	Symbols: | Ribosomal protein S27a / Ubiquitin f	137
Light-1	544	Background	AT1G23410	Symbols: | Ribosomal protein S27a / Ubiquitin f	137
Light-1	544	Background	AT1G23410	Symbols: | Ribosomal protein S27a / Ubiquitin f	137
Light-1	545	Background	AT1G20200	Symbols: EMB2719, HAP15 | PAM domain (PCI/I	134
Light-1	545	Background	AT1G20200	Symbols: EMB2719, HAP15 | PAM domain (PCI/I	134
Light-1	545	Background	AT1G20200	Symbols: EMB2719, HAP15 | PAM domain (PCI/I	134
Light-1	545	Background	AT1G20200	Symbols: EMB2719, HAP15 | PAM domain (PCI/I	134
Light-1	545	Background	AT1G20200	Symbols: EMB2719, HAP15 | PAM domain (PCI/I	134
Light-1	545	Background	AT1G20200	Symbols: EMB2719, HAP15 | PAM domain (PCI/I	134
Light-1	546	Background	AT2G36620	Symbols: RPL24A | ribosomal protein L24 | chr2	133
Light-1	546	Background	AT2G36620	Symbols: RPL24A | ribosomal protein L24 | chr2	133
Light-1	547	Background	AT2G04030	Symbols: CR88, EMB1956, HSP90.5, Hsp88.1, AtI	132
Light-1	547	Background	AT2G04030	Symbols: CR88, EMB1956, HSP90.5, Hsp88.1, AtI	132
Light-1	547	Background	AT2G04030	Symbols: CR88, EMB1956, HSP90.5, Hsp88.1, AtI	132
Light-1	548	Background	AT1G23180	Symbols: | ARM repeat superfamily protein | ch	131
Light-1	548	Background	AT1G23180	Symbols: | ARM repeat superfamily protein | ch	131
Light-1	548	Background	AT1G23180	Symbols: | ARM repeat superfamily protein | ch	131
Light-1	549	Background	AT2G32730	Symbols: | 26S proteasome regulatory complex	130
Light-1	549	Background	AT2G32730	Symbols: | 26S proteasome regulatory complex	130
Light-1	549	Background	AT2G32730	Symbols: | 26S proteasome regulatory complex	130
Light-1	549	Background	AT2G32730	Symbols: | 26S proteasome regulatory complex	130
Light-1	550	Background	AT1G33120	Symbols: | Ribosomal protein L6 family | chr1:1	130
Light-1	550	Background	AT1G33120	Symbols: | Ribosomal protein L6 family | chr1:1	130
Light-1	550	Background	AT1G33120	Symbols: | Ribosomal protein L6 family | chr1:1	130
Light-1	551	Table 1 Filtere	AT2G02100	Symbols: LCR69, PDF2.2 | low-molecular-weight	127
Light-1	551	Table 1 Filtere	AT2G02100	Symbols: LCR69, PDF2.2 | low-molecular-weight	127
Light-1	552	Background	AT2G37190	Symbols: | Ribosomal protein L11 family protein	126
Light-1	552	Background	AT2G37190	Symbols: | Ribosomal protein L11 family protein	126
Light-1	553	Background	AT5G22770	Symbols: alpha-ADR | alpha-adaptin | chr5:7579	126
Light-1	553	Background	AT5G22770	Symbols: alpha-ADR | alpha-adaptin | chr5:7579	126
Light-1	553	Background	AT5G22770	Symbols: alpha-ADR | alpha-adaptin | chr5:7579	126
Light-1	553	Background	AT5G22770	Symbols: alpha-ADR | alpha-adaptin | chr5:7579	126
Light-1	554	Background	AT3G15590	Symbols: | Tetratricopeptide repeat (TPR)-like s	125
Light-1	554	Background	AT3G15590	Symbols: | Tetratricopeptide repeat (TPR)-like s	125
Light-1	555	Background	AT3G59020	Symbols: | ARM repeat superfamily protein | ch	125
Light-1	555	Background	AT3G59020	Symbols: | ARM repeat superfamily protein | ch	125
Light-1	555	Background	AT3G59020	Symbols: | ARM repeat superfamily protein | ch	125
Light-1	555	Background	AT3G59020	Symbols: | ARM repeat superfamily protein | ch	125
Light-1	556	Background	AT2G31610	Symbols: | Ribosomal protein S3 family protein	125
Light-1	556	Background	AT2G31610	Symbols: | Ribosomal protein S3 family protein	125
Light-1	556	Background	AT2G31610	Symbols: | Ribosomal protein S3 family protein	125

Light-1	557	Background	AT5G15610	Symbols: | Proteasome component (PCI) domai	123
Light-1	558	Background	AT1G10630	Symbols: ATARFA1F, ARFA1F | ADP-ribosylation	122
Light-1	558	Background	AT1G10630	Symbols: ATARFA1F, ARFA1F | ADP-ribosylation	122
Light-1	559	Background	AT2G42520	Symbols: | P-loop containing nucleoside triphos	121
Light-1	559	Background	AT2G42520	Symbols: | P-loop containing nucleoside triphos	121
Light-1	559	Background	AT2G42520	Symbols: | P-loop containing nucleoside triphos	121
Light-1	560	Background	AT3G12050	Symbols: | Aha1 domain-containing protein | ch	121
Light-1	560	Background	AT3G12050	Symbols: | Aha1 domain-containing protein | ch	121
Light-1	560	Background	AT3G12050	Symbols: | Aha1 domain-containing protein | ch	121
Light-1	561	Background	AT4G01100	Symbols: ADNT1 | adenine nucleotide transporte	121
Light-1	561	Background	AT4G01100	Symbols: ADNT1 | adenine nucleotide transporte	121
Light-1	562	Background	AT1G42970	Symbols: GAPB | glyceraldehyde-3-phosphate de	120
Light-1	562	Background	AT1G42970	Symbols: GAPB | glyceraldehyde-3-phosphate de	120
Light-1	563	Background	AT1G01960	Symbols: EDA10 | SEC7-like guanine nucleotide	118
Light-1	563	Background	AT1G01960	Symbols: EDA10 | SEC7-like guanine nucleotide	118
Light-1	563	Background	AT1G01960	Symbols: EDA10 | SEC7-like guanine nucleotide	118
Light-1	564	Background	AT5G50850	Symbols: MAB1 | Transketolase family protein |	118
Light-1	564	Background	AT5G50850	Symbols: MAB1 | Transketolase family protein |	118
Light-1	564	Background	AT5G50850	Symbols: MAB1 | Transketolase family protein |	118
Light-1	565	Background	AT3G11510	Symbols: | Ribosomal protein S11 family protein	117
Light-1	565	Background	AT3G11510	Symbols: | Ribosomal protein S11 family protein	117
Light-1	565	Background	AT3G11510	Symbols: | Ribosomal protein S11 family protein	117
Light-1	566	Background	AT2G01250	Symbols: | Ribosomal protein L30/L7 family pro	116
Light-1	566	Background	AT2G01250	Symbols: | Ribosomal protein L30/L7 family pro	116
Light-1	566	Background	AT2G01250	Symbols: | Ribosomal protein L30/L7 family pro	116
Light-1	567	Background	AT2G05830	Symbols: | NagB/RpiA/CoA transferase-like supe	114
Light-1	567	Background	AT2G05830	Symbols: | NagB/RpiA/CoA transferase-like supe	114
Light-1	568	Background	AT1G64520	Symbols: RPN12a | regulatory particle non-ATPa	114
Light-1	568	Background	AT1G64520	Symbols: RPN12a | regulatory particle non-ATPa	114
Light-1	569	Background	AT1G74470	Symbols: | Pyridine nucleotide-disulphide oxido	110
Light-1	569	Background	AT1G74470	Symbols: | Pyridine nucleotide-disulphide oxido	110
Light-1	570	Background	AT1G72440	Symbols: EDA25, SWA2 | CCAAT-binding factor |	110
Light-1	570	Background	AT1G72440	Symbols: EDA25, SWA2 | CCAAT-binding factor |	110
Light-1	571	Background	AT2G36250	Symbols: FTSZ2-1, ATFTSZ2-1 | Tubulin/FtsZ fam	109
Light-1	571	Background	AT2G36250	Symbols: FTSZ2-1, ATFTSZ2-1 | Tubulin/FtsZ fam	109
Light-1	571	Background	AT2G36250	Symbols: FTSZ2-1, ATFTSZ2-1 | Tubulin/FtsZ fam	109
Light-1	572	Background	AT5G08620	Symbols: STRS2, ATRH25 | DEA(D/H)-box RNA he	109
Light-1	572	Background	AT5G08620	Symbols: STRS2, ATRH25 | DEA(D/H)-box RNA he	109
Light-1	572	Background	AT5G08620	Symbols: STRS2, ATRH25 | DEA(D/H)-box RNA he	109
Light-1	572	Background	AT5G08620	Symbols: STRS2, ATRH25 | DEA(D/H)-box RNA he	109
Light-1	573	Background	AT1G24300	Symbols: | GYF domain-containing protein | chr	107
Light-1	573	Background	AT1G24300	Symbols: | GYF domain-containing protein | chr	107
Light-1	573	Background	AT1G24300	Symbols: | GYF domain-containing protein | chr	107
Light-1	574	Background	AT1G23280	Symbols: | MAK16 protein-related | chr1:82608	107
Light-1	574	Background	AT1G23280	Symbols: | MAK16 protein-related | chr1:82608	107
Light-1	575	Background	AT1G67930	Symbols: | Golgi transport complex protein-rela	106
Light-1	575	Background	AT1G67930	Symbols: | Golgi transport complex protein-rela	106
Light-1	576	Background	AT5G20290	Symbols: | Ribosomal protein S8e family protein	106
Light-1	576	Background	AT5G20290	Symbols: | Ribosomal protein S8e family protein	106
Light-1	577	Background	AT1G18450	Symbols: ATARP4, ARP4 | actin-related protein 4	105
Light-1	577	Background	AT1G18450	Symbols: ATARP4, ARP4 | actin-related protein 4	105
Light-1	578	Background	AT5G43780	Symbols: APS4 | Pseudouridine synthase/archae	103
Light-1	578	Background	AT5G43780	Symbols: APS4 | Pseudouridine synthase/archae	103
Light-1	578	Background	AT5G43780	Symbols: APS4 | Pseudouridine synthase/archae	103
Light-1	578	Background	AT5G43780	Symbols: APS4 | Pseudouridine synthase/archae	103
Light-1	579	Background	AT2G40730	Symbols: | Protein kinase family protein with AI	102
Light-1	579	Background	AT2G40730	Symbols: | Protein kinase family protein with AI	102
Light-1	580	Background	AT1G51710	Symbols: UBP6, ATUBP6 | ubiquitin-specific prot	101

Light-1	580	Background	AT1G51710	Symbols: UBP6, ATUBP6 | ubiquitin-specific prot	101
Light-1	580	Background	AT1G51710	Symbols: UBP6, ATUBP6 | ubiquitin-specific prot	101
Light-1	581	Background	AT3G60240	Symbols: EIF4G, CUM2 | eukaryotic translation i	101
Light-1	581	Background	AT3G60240	Symbols: EIF4G, CUM2 | eukaryotic translation i	101
Light-1	582	Background	AT5G09840	Symbols: | Putative endonuclease or glycosyl hy	101
Light-1	582	Background	AT5G09840	Symbols: | Putative endonuclease or glycosyl hy	101
Light-1	583	Background	AT1G80070	Symbols: SUS2, EMB33, EMB177, EMB14 | Pre-r	100
Light-1	583	Background	AT1G80070	Symbols: SUS2, EMB33, EMB177, EMB14 | Pre-r	100
Light-1	583	Background	AT1G80070	Symbols: SUS2, EMB33, EMB177, EMB14 | Pre-r	100
Light-1	583	Background	AT1G80070	Symbols: SUS2, EMB33, EMB177, EMB14 | Pre-r	100
Light-1	583	Background	AT1G80070	Symbols: SUS2, EMB33, EMB177, EMB14 | Pre-r	100
Light-1	584	Background	AT5G16390	Symbols: CAC1, CAC1A, BCCP, BCCP1 | chloropla	99
Light-1	584	Background	AT5G16390	Symbols: CAC1, CAC1A, BCCP, BCCP1 | chloropla	99
Light-1	584	Background	AT5G16390	Symbols: CAC1, CAC1A, BCCP, BCCP1 | chloropla	99
Light-1	585	Background	AT3G13290	Symbols: VCR | varicose-related | chr3:4297529	99
Light-1	585	Background	AT3G13290	Symbols: VCR | varicose-related | chr3:4297529	99
Light-1	585	Background	AT3G13290	Symbols: VCR | varicose-related | chr3:4297529	99
Light-1	586	Background	AT5G10470	Symbols: KCA1, KAC1 | kinesin like protein for ac	95
Light-1	586	Background	AT5G10470	Symbols: KCA1, KAC1 | kinesin like protein for ac	95
Light-1	587	Background	AT5G27640	Symbols: TIF3B1, EIF3B, ATEIF3B-1, EIF3B-1, ATT	94
Light-1	587	Background	AT5G27640	Symbols: TIF3B1, EIF3B, ATEIF3B-1, EIF3B-1, ATT	94
Light-1	587	Background	AT5G27640	Symbols: TIF3B1, EIF3B, ATEIF3B-1, EIF3B-1, ATT	94
Light-1	588	Background	AT1G72370	Symbols: P40, AP40, RP40, RPSAA | 40s ribosom	94
Light-1	588	Background	AT1G72370	Symbols: P40, AP40, RP40, RPSAA | 40s ribosom	94
Light-1	589	Background	AT5G18620	Symbols: CHR17 | chromatin remodeling factor1	93
Light-1	589	Background	AT5G18620	Symbols: CHR17 | chromatin remodeling factor1	93
Light-1	589	Background	AT5G18620	Symbols: CHR17 | chromatin remodeling factor1	93
Light-1	589	Background	AT5G18620	Symbols: CHR17 | chromatin remodeling factor1	93
Light-1	590	Background	AT1G15480	Symbols: | Tetratricopeptide repeat (TPR)-like s	93
Light-1	590	Background	AT1G15480	Symbols: | Tetratricopeptide repeat (TPR)-like s	93
Light-1	591	Background	AT3G18600	Symbols: | P-loop containing nucleoside triphos	93
Light-1	591	Background	AT3G18600	Symbols: | P-loop containing nucleoside triphos	93
Light-1	591	Background	AT3G18600	Symbols: | P-loop containing nucleoside triphos	93
Light-1	592	Background	AT5G05780	Symbols: RPN8A, AE3, ATHMOV34 | RP non-ATF	89
Light-1	592	Background	AT5G05780	Symbols: RPN8A, AE3, ATHMOV34 | RP non-ATF	89
Light-1	593	Background	AT1G58380	Symbols: XW6 | Ribosomal protein S5 family prc	88
Light-1	593	Background	AT1G58380	Symbols: XW6 | Ribosomal protein S5 family prc	88
Light-1	594	Background	AT4G31480	Symbols: | Coatomer, beta subunit | chr4:1526	88
Light-1	594	Background	AT4G31480	Symbols: | Coatomer, beta subunit | chr4:1526	88
Light-1	595	Background	AT2G40010	Symbols: | Ribosomal protein L10 family protein	87
Light-1	595	Background	AT2G40010	Symbols: | Ribosomal protein L10 family protein	87
Light-1	596	Background	AT1G52360	Symbols: | Coatomer, beta~ subunit | chr1:194	87
Light-1	596	Background	AT1G52360	Symbols: | Coatomer, beta~ subunit | chr1:194	87
Light-1	597	Background	AT5G25754	Symbols: | RNA polymerase I-associated factor	81
Light-1	597	Background	AT5G25754	Symbols: | RNA polymerase I-associated factor	81
Light-1	598	Background	AT1G71820	Symbols: SEC6 | SEC6 | chr1:27010022-2701674	78
Light-1	598	Background	AT1G71820	Symbols: SEC6 | SEC6 | chr1:27010022-2701674	78
Light-1	598	Background	AT1G71820	Symbols: SEC6 | SEC6 | chr1:27010022-2701674	78
Light-1	599	Background	AT2G46280	Symbols: TRIP-1, TIF3I1 | TGF-beta receptor inte	78
Light-1	599	Background	AT2G46280	Symbols: TRIP-1, TIF3I1 | TGF-beta receptor inte	78
Light-1	600	Background	AT5G64420	Symbols: | DNA polymerase V family | chr5:257	78
Light-1	601	Background	AT3G10270	Symbols: GYRB1 | DNA GYRASE B1 | chr3:31738	77
Light-1	601	Background	AT3G10270	Symbols: GYRB1 | DNA GYRASE B1 | chr3:31738	77
Light-1	601	Background	AT3G10270	Symbols: GYRB1 | DNA GYRASE B1 | chr3:31738	77
Light-1	602	Background	AT1G71380	Symbols: ATGH9B3, ATCEL3, CEL3 | cellulase 3 |	76
Light-1	602	Background	AT1G71380	Symbols: ATGH9B3, ATCEL3, CEL3 | cellulase 3 |	76
Light-1	603	Background	AT1G50200	Symbols: ALATS, ACD | Alanyl-tRNA synthetase |	76
Light-1	603	Background	AT1G50200	Symbols: ALATS, ACD | Alanyl-tRNA synthetase |	76

Light-1	604	Background	AT5G26360	Symbols: | TCP-1/cpn60 chaperonin family prot	74
Light-1	604	Background	AT5G26360	Symbols: | TCP-1/cpn60 chaperonin family prot	74
Light-1	604	Background	AT5G26360	Symbols: | TCP-1/cpn60 chaperonin family prot	74
Light-1	605	Background	ATMG00070	Symbols: NAD9 | NADH dehydrogenase subunit	74
Light-1	605	Background	ATMG00070	Symbols: NAD9 | NADH dehydrogenase subunit	74
Light-1	606	Background	AT1G04480	Symbols: | Ribosomal protein L14p/L23e family	74
Light-1	606	Background	AT1G04480	Symbols: | Ribosomal protein L14p/L23e family	74
Light-1	607	Background	AT5G18230	Symbols: | transcription regulator NOT2/NOT3/	74
Light-1	607	Background	AT5G18230	Symbols: | transcription regulator NOT2/NOT3/	74
Light-1	608	Background	AT3G54110	Symbols: ATPUMP1, UCP, PUMP1, ATUCP1, UCP	72
Light-1	608	Background	AT3G54110	Symbols: ATPUMP1, UCP, PUMP1, ATUCP1, UCP	72
Light-1	609	Background	AT4G02150	Symbols: MOS6, ATIMPALPHA3, IMPA-3 | ARM r	71
Light-1	609	Background	AT4G02150	Symbols: MOS6, ATIMPALPHA3, IMPA-3 | ARM r	71
Light-1	609	Background	AT4G02150	Symbols: MOS6, ATIMPALPHA3, IMPA-3 | ARM r	71
Light-1	610	Background	AT3G05560	Symbols: | Ribosomal L22e protein family | chr3	71
Light-1	610	Background	AT3G05560	Symbols: | Ribosomal L22e protein family | chr3	71
Light-1	611	Background	AT1G09270	Symbols: IMPA-4 | importin alpha isoform 4 | ch	70
Light-1	611	Background	AT1G09270	Symbols: IMPA-4 | importin alpha isoform 4 | ch	70
Light-1	612	Background	AT5G15200	Symbols: | Ribosomal protein S4 | chr5:493512	69
Light-1	612	Background	AT5G15200	Symbols: | Ribosomal protein S4 | chr5:493512	69
Light-1	613	Background	AT5G27770	Symbols: | Ribosomal L22e protein family | chr5	69
Light-1	613	Background	AT5G27770	Symbols: | Ribosomal L22e protein family | chr5	69
Light-1	614	Background	AT5G42220	Symbols: | Ubiquitin-like superfamily protein | c	69
Light-1	614	Background	AT5G42220	Symbols: | Ubiquitin-like superfamily protein | c	69
Light-1	615	Background	AT5G45550	Symbols: | Mob1/phocein family protein | chr5	67
Light-1	615	Background	AT5G45550	Symbols: | Mob1/phocein family protein | chr5	67
Light-1	616	Background	AT5G15090	Symbols: VDAC3, ATVDAC3 | voltage dependent	66
Light-1	616	Background	AT5G15090	Symbols: VDAC3, ATVDAC3 | voltage dependent	66
Light-1	617	Background	AT3G08947	Symbols: | ARM repeat superfamily protein | ch	62
Light-1	617	Background	AT3G08947	Symbols: | ARM repeat superfamily protein | ch	62
Light-1	618	Background	AT2G03510	Symbols: | SPFH/Band 7/PHB domain-containin	59
Light-1	618	Background	AT2G03510	Symbols: | SPFH/Band 7/PHB domain-containin	59
Light-1	619	Background	AT3G60770	Symbols: | Ribosomal protein S13/S15 | chr3:22	59
Light-1	619	Background	AT3G60770	Symbols: | Ribosomal protein S13/S15 | chr3:22	59
Light-1	619	Background	AT3G60770	Symbols: | Ribosomal protein S13/S15 | chr3:22	59
Light-1	620	Background	AT2G41040	Symbols: | S-adenosyl-L-methionine-dependent	57
Light-1	620	Background	AT2G41040	Symbols: | S-adenosyl-L-methionine-dependent	57
Light-1	621	Background	AT2G17360	Symbols: | Ribosomal protein S4 (RPS4A) family	53
Light-1	621	Background	AT2G17360	Symbols: | Ribosomal protein S4 (RPS4A) family	53
Light-1	622	Background	AT2G18020	Symbols: EMB2296 | Ribosomal protein L2 famil	53
Light-1	622	Background	AT2G18020	Symbols: EMB2296 | Ribosomal protein L2 famil	53
Light-1	623	Background	AT4G25630	Symbols: FIB2, ATFIB2 | fibrillarin 2 | chr4:1307	51
Light-1	623	Background	AT4G25630	Symbols: FIB2, ATFIB2 | fibrillarin 2 | chr4:1307	51
Light-1	624	Background	AT1G22730	Symbols: | MA3 domain-containing protein | ch	50
Light-1	624	Background	AT1G22730	Symbols: | MA3 domain-containing protein | ch	50
Light-1	625	Background	AT2G02090	Symbols: CHR19, CHA19, ETL1 | SNF2 domain-co	49
Light-1	625	Background	AT2G02090	Symbols: CHR19, CHA19, ETL1 | SNF2 domain-co	49
Light-1	626	Background	AT4G27090	Symbols: | Ribosomal protein L14 | chr4:13594	45
Light-1	626	Background	AT4G27090	Symbols: | Ribosomal protein L14 | chr4:13594	45
Light-1	627	Background	AT1G24290	Symbols: | AAA-type ATPase family protein | ch	42
Light-1	627	Background	AT1G24290	Symbols: | AAA-type ATPase family protein | ch	42
Light-2	628	Background	prot_acc	prot_desc	prot_score
Light-2	629	Background	AT5G44340	Symbols: TUB4 | tubulin beta chain 4 | chr5:178	1313
Light-2	629	Background	AT5G44340	Symbols: TUB4 | tubulin beta chain 4 | chr5:178	1313
Light-2	629	Background	AT5G44340	Symbols: TUB4 | tubulin beta chain 4 | chr5:178	1313
Light-2	629	Background	AT5G44340	Symbols: TUB4 | tubulin beta chain 4 | chr5:178	1313
Light-2	629	Background	AT5G44340	Symbols: TUB4 | tubulin beta chain 4 | chr5:178	1313
Light-2	629	Background	AT5G44340	Symbols: TUB4 | tubulin beta chain 4 | chr5:178	1313

Light-2	629	Background	AT5G44340	Symbols: TUB4 | tubulin beta chain 4 | chr5:178	1313
Light-2	629	Background	AT5G44340	Symbols: TUB4 | tubulin beta chain 4 | chr5:178	1313
Light-2	629	Background	AT5G44340	Symbols: TUB4 | tubulin beta chain 4 | chr5:178	1313
Light-2	629	Background	AT5G44340	Symbols: TUB4 | tubulin beta chain 4 | chr5:178	1313
Light-2	629	Background	AT5G44340	Symbols: TUB4 | tubulin beta chain 4 | chr5:178	1313
Light-2	629	Background	AT5G44340	Symbols: TUB4 | tubulin beta chain 4 | chr5:178	1313
Light-2	629	Background	AT5G44340	Symbols: TUB4 | tubulin beta chain 4 | chr5:178	1313
Light-2	629	Background	AT5G44340	Symbols: TUB4 | tubulin beta chain 4 | chr5:178	1313
Light-2	629	Background	AT5G44340	Symbols: TUB4 | tubulin beta chain 4 | chr5:178	1313
Light-2	629	Background	AT5G44340	Symbols: TUB4 | tubulin beta chain 4 | chr5:178	1313
Light-2	629	Background	AT5G44340	Symbols: TUB4 | tubulin beta chain 4 | chr5:178	1313
Light-2	629	Background	AT5G44340	Symbols: TUB4 | tubulin beta chain 4 | chr5:178	1313
Light-2	630	Background	AT5G62690	Symbols: TUB2 | tubulin beta chain 2 | chr5:251	1143
Light-2	630	Background	AT5G62690	Symbols: TUB2 | tubulin beta chain 2 | chr5:251	1143
Light-2	630	Background	AT5G62690	Symbols: TUB2 | tubulin beta chain 2 | chr5:251	1143
Light-2	630	Background	AT5G62690	Symbols: TUB2 | tubulin beta chain 2 | chr5:251	1143
Light-2	630	Background	AT5G62690	Symbols: TUB2 | tubulin beta chain 2 | chr5:251	1143
Light-2	630	Background	AT5G62690	Symbols: TUB2 | tubulin beta chain 2 | chr5:251	1143
Light-2	630	Background	AT5G62690	Symbols: TUB2 | tubulin beta chain 2 | chr5:251	1143
Light-2	630	Background	AT5G62690	Symbols: TUB2 | tubulin beta chain 2 | chr5:251	1143
Light-2	630	Background	AT5G62690	Symbols: TUB2 | tubulin beta chain 2 | chr5:251	1143
Light-2	630	Background	AT5G62690	Symbols: TUB2 | tubulin beta chain 2 | chr5:251	1143
Light-2	630	Background	AT5G62690	Symbols: TUB2 | tubulin beta chain 2 | chr5:251	1143
Light-2	630	Background	AT5G62690	Symbols: TUB2 | tubulin beta chain 2 | chr5:251	1143
Light-2	630	Background	AT5G62690	Symbols: TUB2 | tubulin beta chain 2 | chr5:251	1143
Light-2	630	Background	AT5G62690	Symbols: TUB2 | tubulin beta chain 2 | chr5:251	1143
Light-2	630	Background	AT5G62690	Symbols: TUB2 | tubulin beta chain 2 | chr5:251	1143
Light-2	631	Background	AT3G09440	Symbols: | Heat shock protein 70 (Hsp 70) famil	1091
Light-2	631	Background	AT3G09440	Symbols: | Heat shock protein 70 (Hsp 70) famil	1091
Light-2	631	Background	AT3G09440	Symbols: | Heat shock protein 70 (Hsp 70) famil	1091
Light-2	631	Background	AT3G09440	Symbols: | Heat shock protein 70 (Hsp 70) famil	1091
Light-2	631	Background	AT3G09440	Symbols: | Heat shock protein 70 (Hsp 70) famil	1091
Light-2	631	Background	AT3G09440	Symbols: | Heat shock protein 70 (Hsp 70) famil	1091
Light-2	631	Background	AT3G09440	Symbols: | Heat shock protein 70 (Hsp 70) famil	1091
Light-2	631	Background	AT3G09440	Symbols: | Heat shock protein 70 (Hsp 70) famil	1091
Light-2	631	Background	AT3G09440	Symbols: | Heat shock protein 70 (Hsp 70) famil	1091
Light-2	631	Background	AT3G09440	Symbols: | Heat shock protein 70 (Hsp 70) famil	1091
Light-2	631	Background	AT3G09440	Symbols: | Heat shock protein 70 (Hsp 70) famil	1091
Light-2	631	Background	AT3G09440	Symbols: | Heat shock protein 70 (Hsp 70) famil	1091
Light-2	631	Background	AT3G09440	Symbols: | Heat shock protein 70 (Hsp 70) famil	1091
Light-2	631	Background	AT3G09440	Symbols: | Heat shock protein 70 (Hsp 70) famil	1091
Light-2	631	Background	AT3G09440	Symbols: | Heat shock protein 70 (Hsp 70) famil	1091
Light-2	631	Background	AT3G09440	Symbols: | Heat shock protein 70 (Hsp 70) famil	1091
Light-2	631	Background	AT3G09440	Symbols: | Heat shock protein 70 (Hsp 70) famil	1091
Light-2	631	Background	AT3G09440	Symbols: | Heat shock protein 70 (Hsp 70) famil	1091
Light-2	631	Background	AT3G09440	Symbols: | Heat shock protein 70 (Hsp 70) famil	1091
Light-2	632	Background	AT5G23860	Symbols: TUB8 | tubulin beta 8 | chr5:8042962-	1077
Light-2	632	Background	AT5G23860	Symbols: TUB8 | tubulin beta 8 | chr5:8042962-	1077
Light-2	632	Background	AT5G23860	Symbols: TUB8 | tubulin beta 8 | chr5:8042962-	1077
Light-2	632	Background	AT5G23860	Symbols: TUB8 | tubulin beta 8 | chr5:8042962-	1077
Light-2	632	Background	AT5G23860	Symbols: TUB8 | tubulin beta 8 | chr5:8042962-	1077
Light-2	632	Background	AT5G23860	Symbols: TUB8 | tubulin beta 8 | chr5:8042962-	1077
Light-2	632	Background	AT5G23860	Symbols: TUB8 | tubulin beta 8 | chr5:8042962-	1077
Light-2	632	Background	AT5G23860	Symbols: TUB8 | tubulin beta 8 | chr5:8042962-	1077
Light-2	632	Background	AT5G23860	Symbols: TUB8 | tubulin beta 8 | chr5:8042962-	1077
Light-2	632	Background	AT5G23860	Symbols: TUB8 | tubulin beta 8 | chr5:8042962-	1077
Light-2	632	Background	AT5G23860	Symbols: TUB8 | tubulin beta 8 | chr5:8042962-	1077
Light-2	632	Background	AT5G23860	Symbols: TUB8 | tubulin beta 8 | chr5:8042962-	1077

Light-2	632	Background	AT5G23860	Symbols: TUB8 | tubulin beta 8 | chr5:8042962-	1077
Light-2	632	Background	AT5G23860	Symbols: TUB8 | tubulin beta 8 | chr5:8042962-	1077
Light-2	633	Background	AT5G02500	Symbols: HSC70-1, HSP70-1, AT-HSC70-1, HSC70	1044
Light-2	633	Background	AT5G02500	Symbols: HSC70-1, HSP70-1, AT-HSC70-1, HSC70	1044
Light-2	633	Background	AT5G02500	Symbols: HSC70-1, HSP70-1, AT-HSC70-1, HSC70	1044
Light-2	633	Background	AT5G02500	Symbols: HSC70-1, HSP70-1, AT-HSC70-1, HSC70	1044
Light-2	633	Background	AT5G02500	Symbols: HSC70-1, HSP70-1, AT-HSC70-1, HSC70	1044
Light-2	633	Background	AT5G02500	Symbols: HSC70-1, HSP70-1, AT-HSC70-1, HSC70	1044
Light-2	633	Background	AT5G02500	Symbols: HSC70-1, HSP70-1, AT-HSC70-1, HSC70	1044
Light-2	633	Background	AT5G02500	Symbols: HSC70-1, HSP70-1, AT-HSC70-1, HSC70	1044
Light-2	633	Background	AT5G02500	Symbols: HSC70-1, HSP70-1, AT-HSC70-1, HSC70	1044
Light-2	633	Background	AT5G02500	Symbols: HSC70-1, HSP70-1, AT-HSC70-1, HSC70	1044
Light-2	633	Background	AT5G02500	Symbols: HSC70-1, HSP70-1, AT-HSC70-1, HSC70	1044
Light-2	633	Background	AT5G02500	Symbols: HSC70-1, HSP70-1, AT-HSC70-1, HSC70	1044
Light-2	633	Background	AT5G02500	Symbols: HSC70-1, HSP70-1, AT-HSC70-1, HSC70	1044
Light-2	633	Background	AT5G02500	Symbols: HSC70-1, HSP70-1, AT-HSC70-1, HSC70	1044
Light-2	633	Background	AT5G02500	Symbols: HSC70-1, HSP70-1, AT-HSC70-1, HSC70	1044
Light-2	633	Background	AT5G02500	Symbols: HSC70-1, HSP70-1, AT-HSC70-1, HSC70	1044
Light-2	633	Background	AT5G02500	Symbols: HSC70-1, HSP70-1, AT-HSC70-1, HSC70	1044
Light-2	633	Background	AT5G02500	Symbols: HSC70-1, HSP70-1, AT-HSC70-1, HSC70	1044
Light-2	634	Background	AT2G29550	Symbols: TUB7 | tubulin beta-7 chain | chr2:126	1034
Light-2	634	Background	AT2G29550	Symbols: TUB7 | tubulin beta-7 chain | chr2:126	1034
Light-2	634	Background	AT2G29550	Symbols: TUB7 | tubulin beta-7 chain | chr2:126	1034
Light-2	634	Background	AT2G29550	Symbols: TUB7 | tubulin beta-7 chain | chr2:126	1034
Light-2	634	Background	AT2G29550	Symbols: TUB7 | tubulin beta-7 chain | chr2:126	1034
Light-2	634	Background	AT2G29550	Symbols: TUB7 | tubulin beta-7 chain | chr2:126	1034
Light-2	634	Background	AT2G29550	Symbols: TUB7 | tubulin beta-7 chain | chr2:126	1034
Light-2	634	Background	AT2G29550	Symbols: TUB7 | tubulin beta-7 chain | chr2:126	1034
Light-2	634	Background	AT2G29550	Symbols: TUB7 | tubulin beta-7 chain | chr2:126	1034
Light-2	634	Background	AT2G29550	Symbols: TUB7 | tubulin beta-7 chain | chr2:126	1034
Light-2	634	Background	AT2G29550	Symbols: TUB7 | tubulin beta-7 chain | chr2:126	1034
Light-2	634	Background	AT2G29550	Symbols: TUB7 | tubulin beta-7 chain | chr2:126	1034
Light-2	634	Background	AT2G29550	Symbols: TUB7 | tubulin beta-7 chain | chr2:126	1034
Light-2	634	Background	AT2G29550	Symbols: TUB7 | tubulin beta-7 chain | chr2:126	1034
Light-2	635	Background	AT1G04820	Symbols: TUA4, TOR2 | tubulin alpha-4 chain | c	849
Light-2	635	Background	AT1G04820	Symbols: TUA4, TOR2 | tubulin alpha-4 chain | c	849
Light-2	635	Background	AT1G04820	Symbols: TUA4, TOR2 | tubulin alpha-4 chain | c	849
Light-2	635	Background	AT1G04820	Symbols: TUA4, TOR2 | tubulin alpha-4 chain | c	849
Light-2	635	Background	AT1G04820	Symbols: TUA4, TOR2 | tubulin alpha-4 chain | c	849
Light-2	635	Background	AT1G04820	Symbols: TUA4, TOR2 | tubulin alpha-4 chain | c	849
Light-2	635	Background	AT1G04820	Symbols: TUA4, TOR2 | tubulin alpha-4 chain | c	849
Light-2	635	Background	AT1G04820	Symbols: TUA4, TOR2 | tubulin alpha-4 chain | c	849
Light-2	635	Background	AT1G04820	Symbols: TUA4, TOR2 | tubulin alpha-4 chain | c	849
Light-2	635	Background	AT1G04820	Symbols: TUA4, TOR2 | tubulin alpha-4 chain | c	849
Light-2	635	Background	AT1G04820	Symbols: TUA4, TOR2 | tubulin alpha-4 chain | c	849
Light-2	635	Background	AT1G04820	Symbols: TUA4, TOR2 | tubulin alpha-4 chain | c	849
Light-2	635	Background	AT1G04820	Symbols: TUA4, TOR2 | tubulin alpha-4 chain | c	849
Light-2	635	Background	AT1G04820	Symbols: TUA4, TOR2 | tubulin alpha-4 chain | c	849
Light-2	636	Background	AT3G13920	Symbols: EIF4A1, RH4, TIF4A1 | eukaryotic trans	832
Light-2	636	Background	AT3G13920	Symbols: EIF4A1, RH4, TIF4A1 | eukaryotic trans	832
Light-2	636	Background	AT3G13920	Symbols: EIF4A1, RH4, TIF4A1 | eukaryotic trans	832
Light-2	636	Background	AT3G13920	Symbols: EIF4A1, RH4, TIF4A1 | eukaryotic trans	832
Light-2	636	Background	AT3G13920	Symbols: EIF4A1, RH4, TIF4A1 | eukaryotic trans	832
Light-2	636	Background	AT3G13920	Symbols: EIF4A1, RH4, TIF4A1 | eukaryotic trans	832
Light-2	636	Background	AT3G13920	Symbols: EIF4A1, RH4, TIF4A1 | eukaryotic trans	832
Light-2	636	Background	AT3G13920	Symbols: EIF4A1, RH4, TIF4A1 | eukaryotic trans	832
Light-2	636	Background	AT3G13920	Symbols: EIF4A1, RH4, TIF4A1 | eukaryotic trans	832
Light-2	636	Background	AT3G13920	Symbols: EIF4A1, RH4, TIF4A1 | eukaryotic trans	832

Light-2	636	Background	AT3G13920	Symbols: EIF4A1, RH4, TIF4A1 | eukaryotic trans	832
Light-2	636	Background	AT3G13920	Symbols: EIF4A1, RH4, TIF4A1 | eukaryotic trans	832
Light-2	636	Background	AT3G13920	Symbols: EIF4A1, RH4, TIF4A1 | eukaryotic trans	832
Light-2	636	Background	AT3G13920	Symbols: EIF4A1, RH4, TIF4A1 | eukaryotic trans	832
Light-2	637	Background	AT1G54270	Symbols: EIF4A-2 | eif4a-2 | chr1:20260495-202	826
Light-2	637	Background	AT1G54270	Symbols: EIF4A-2 | eif4a-2 | chr1:20260495-202	826
Light-2	637	Background	AT1G54270	Symbols: EIF4A-2 | eif4a-2 | chr1:20260495-202	826
Light-2	637	Background	AT1G54270	Symbols: EIF4A-2 | eif4a-2 | chr1:20260495-202	826
Light-2	637	Background	AT1G54270	Symbols: EIF4A-2 | eif4a-2 | chr1:20260495-202	826
Light-2	637	Background	AT1G54270	Symbols: EIF4A-2 | eif4a-2 | chr1:20260495-202	826
Light-2	637	Background	AT1G54270	Symbols: EIF4A-2 | eif4a-2 | chr1:20260495-202	826
Light-2	637	Background	AT1G54270	Symbols: EIF4A-2 | eif4a-2 | chr1:20260495-202	826
Light-2	637	Background	AT1G54270	Symbols: EIF4A-2 | eif4a-2 | chr1:20260495-202	826
Light-2	637	Background	AT1G54270	Symbols: EIF4A-2 | eif4a-2 | chr1:20260495-202	826
Light-2	637	Background	AT1G54270	Symbols: EIF4A-2 | eif4a-2 | chr1:20260495-202	826
Light-2	637	Background	AT1G54270	Symbols: EIF4A-2 | eif4a-2 | chr1:20260495-202	826
Light-2	637	Background	AT1G54270	Symbols: EIF4A-2 | eif4a-2 | chr1:20260495-202	826
Light-2	638	Background	AT5G02490	Symbols: | Heat shock protein 70 (Hsp 70) famil	677
Light-2	638	Background	AT5G02490	Symbols: | Heat shock protein 70 (Hsp 70) famil	677
Light-2	638	Background	AT5G02490	Symbols: | Heat shock protein 70 (Hsp 70) famil	677
Light-2	638	Background	AT5G02490	Symbols: | Heat shock protein 70 (Hsp 70) famil	677
Light-2	638	Background	AT5G02490	Symbols: | Heat shock protein 70 (Hsp 70) famil	677
Light-2	638	Background	AT5G02490	Symbols: | Heat shock protein 70 (Hsp 70) famil	677
Light-2	638	Background	AT5G02490	Symbols: | Heat shock protein 70 (Hsp 70) famil	677
Light-2	638	Background	AT5G02490	Symbols: | Heat shock protein 70 (Hsp 70) famil	677
Light-2	638	Background	AT5G02490	Symbols: | Heat shock protein 70 (Hsp 70) famil	677
Light-2	638	Background	AT5G02490	Symbols: | Heat shock protein 70 (Hsp 70) famil	677
Light-2	638	Background	AT5G02490	Symbols: | Heat shock protein 70 (Hsp 70) famil	677
Light-2	639	Background	AT3G12580	Symbols: HSP70, ATHSP70 | heat shock protein	673
Light-2	639	Background	AT3G12580	Symbols: HSP70, ATHSP70 | heat shock protein	673
Light-2	639	Background	AT3G12580	Symbols: HSP70, ATHSP70 | heat shock protein	673
Light-2	639	Background	AT3G12580	Symbols: HSP70, ATHSP70 | heat shock protein	673
Light-2	639	Background	AT3G12580	Symbols: HSP70, ATHSP70 | heat shock protein	673
Light-2	639	Background	AT3G12580	Symbols: HSP70, ATHSP70 | heat shock protein	673
Light-2	639	Background	AT3G12580	Symbols: HSP70, ATHSP70 | heat shock protein	673
Light-2	639	Background	AT3G12580	Symbols: HSP70, ATHSP70 | heat shock protein	673
Light-2	639	Background	AT3G12580	Symbols: HSP70, ATHSP70 | heat shock protein	673
Light-2	639	Background	AT3G12580	Symbols: HSP70, ATHSP70 | heat shock protein	673
Light-2	639	Background	AT3G12580	Symbols: HSP70, ATHSP70 | heat shock protein	673
Light-2	639	Background	AT3G12580	Symbols: HSP70, ATHSP70 | heat shock protein	673
Light-2	639	Background	AT3G12580	Symbols: HSP70, ATHSP70 | heat shock protein	673
Light-2	640	Background	AT5G35360	Symbols: CAC2 | acetyl Co-enzyme a carboxylase	636
Light-2	640	Background	AT5G35360	Symbols: CAC2 | acetyl Co-enzyme a carboxylase	636
Light-2	640	Background	AT5G35360	Symbols: CAC2 | acetyl Co-enzyme a carboxylase	636
Light-2	640	Background	AT5G35360	Symbols: CAC2 | acetyl Co-enzyme a carboxylase	636
Light-2	640	Background	AT5G35360	Symbols: CAC2 | acetyl Co-enzyme a carboxylase	636
Light-2	640	Background	AT5G35360	Symbols: CAC2 | acetyl Co-enzyme a carboxylase	636
Light-2	640	Background	AT5G35360	Symbols: CAC2 | acetyl Co-enzyme a carboxylase	636
Light-2	640	Background	AT5G35360	Symbols: CAC2 | acetyl Co-enzyme a carboxylase	636
Light-2	640	Background	AT5G35360	Symbols: CAC2 | acetyl Co-enzyme a carboxylase	636
Light-2	640	Background	AT5G35360	Symbols: CAC2 | acetyl Co-enzyme a carboxylase	636
Light-2	640	Background	AT5G35360	Symbols: CAC2 | acetyl Co-enzyme a carboxylase	636
Light-2	640	Background	AT5G35360	Symbols: CAC2 | acetyl Co-enzyme a carboxylase	636
Light-2	640	Background	AT5G35360	Symbols: CAC2 | acetyl Co-enzyme a carboxylase	636
Light-2	640	Background	AT5G35360	Symbols: CAC2 | acetyl Co-enzyme a carboxylase	636
Light-2	640	Background	AT5G35360	Symbols: CAC2 | acetyl Co-enzyme a carboxylase	636
Light-2	641	Background	AT5G12250	Symbols: TUB6 | beta-6 tubulin | chr5:3961317-	540
Light-2	641	Background	AT5G12250	Symbols: TUB6 | beta-6 tubulin | chr5:3961317-	540

Light-2	641	Background	AT5G12250	Symbols: TUB6 | beta-6 tubulin | chr5:3961317-	540
Light-2	641	Background	AT5G12250	Symbols: TUB6 | beta-6 tubulin | chr5:3961317-	540
Light-2	641	Background	AT5G12250	Symbols: TUB6 | beta-6 tubulin | chr5:3961317-	540
Light-2	641	Background	AT5G12250	Symbols: TUB6 | beta-6 tubulin | chr5:3961317-	540
Light-2	641	Background	AT5G12250	Symbols: TUB6 | beta-6 tubulin | chr5:3961317-	540
Light-2	641	Background	AT5G12250	Symbols: TUB6 | beta-6 tubulin | chr5:3961317-	540
Light-2	641	Background	AT5G12250	Symbols: TUB6 | beta-6 tubulin | chr5:3961317-	540
Light-2	641	Background	AT5G12250	Symbols: TUB6 | beta-6 tubulin | chr5:3961317-	540
Light-2	642	Table 1 Filtere	AT4G02380	Symbols: SAG21, AtLEA5 | senescence-associate	525
Light-2	642	Table 1 Filtere	AT4G02380	Symbols: SAG21, AtLEA5 | senescence-associate	525
Light-2	642	Table 1 Filtere	AT4G02380	Symbols: SAG21, AtLEA5 | senescence-associate	525
Light-2	642	Table 1 Filtere	AT4G02380	Symbols: SAG21, AtLEA5 | senescence-associate	525
Light-2	642	Table 1 Filtere	AT4G02380	Symbols: SAG21, AtLEA5 | senescence-associate	525
Light-2	643	Background	AT5G19770	Symbols: TUA3 | tubulin alpha-3 | chr5:6682761	516
Light-2	643	Background	AT5G19770	Symbols: TUA3 | tubulin alpha-3 | chr5:6682761	516
Light-2	643	Background	AT5G19770	Symbols: TUA3 | tubulin alpha-3 | chr5:6682761	516
Light-2	643	Background	AT5G19770	Symbols: TUA3 | tubulin alpha-3 | chr5:6682761	516
Light-2	643	Background	AT5G19770	Symbols: TUA3 | tubulin alpha-3 | chr5:6682761	516
Light-2	643	Background	AT5G19770	Symbols: TUA3 | tubulin alpha-3 | chr5:6682761	516
Light-2	643	Background	AT5G19770	Symbols: TUA3 | tubulin alpha-3 | chr5:6682761	516
Light-2	643	Background	AT5G19770	Symbols: TUA3 | tubulin alpha-3 | chr5:6682761	516
Light-2	643	Background	AT5G19770	Symbols: TUA3 | tubulin alpha-3 | chr5:6682761	516
Light-2	644	Background	AT1G13440	Symbols: GAPC-2, GAPC2 | glyceraldehyde-3-ph	512
Light-2	644	Background	AT1G13440	Symbols: GAPC-2, GAPC2 | glyceraldehyde-3-ph	512
Light-2	644	Background	AT1G13440	Symbols: GAPC-2, GAPC2 | glyceraldehyde-3-ph	512
Light-2	644	Background	AT1G13440	Symbols: GAPC-2, GAPC2 | glyceraldehyde-3-ph	512
Light-2	644	Background	AT1G13440	Symbols: GAPC-2, GAPC2 | glyceraldehyde-3-ph	512
Light-2	644	Background	AT1G13440	Symbols: GAPC-2, GAPC2 | glyceraldehyde-3-ph	512
Light-2	644	Background	AT1G13440	Symbols: GAPC-2, GAPC2 | glyceraldehyde-3-ph	512
Light-2	644	Background	AT1G13440	Symbols: GAPC-2, GAPC2 | glyceraldehyde-3-ph	512
Light-2	645	Background	AT4G20360	Symbols: ATRAB8D, ATRABE1B, RABE1b | RAB G	511
Light-2	645	Background	AT4G20360	Symbols: ATRAB8D, ATRABE1B, RABE1b | RAB G	511
Light-2	645	Background	AT4G20360	Symbols: ATRAB8D, ATRABE1B, RABE1b | RAB G	511
Light-2	645	Background	AT4G20360	Symbols: ATRAB8D, ATRABE1B, RABE1b | RAB G	511
Light-2	645	Background	AT4G20360	Symbols: ATRAB8D, ATRABE1B, RABE1b | RAB G	511
Light-2	645	Background	AT4G20360	Symbols: ATRAB8D, ATRABE1B, RABE1b | RAB G	511
Light-2	645	Background	AT4G20360	Symbols: ATRAB8D, ATRABE1B, RABE1b | RAB G	511
Light-2	645	Background	AT4G20360	Symbols: ATRAB8D, ATRABE1B, RABE1b | RAB G	511
Light-2	645	Background	AT4G20360	Symbols: ATRAB8D, ATRABE1B, RABE1b | RAB G	511
Light-2	645	Background	AT4G20360	Symbols: ATRAB8D, ATRABE1B, RABE1b | RAB G	511
Light-2	645	Background	AT4G20360	Symbols: ATRAB8D, ATRABE1B, RABE1b | RAB G	511
Light-2	646	Background	AT1G07920	Symbols: | GTP binding Elongation factor Tu fan	430
Light-2	646	Background	AT1G07920	Symbols: | GTP binding Elongation factor Tu fan	430
Light-2	646	Background	AT1G07920	Symbols: | GTP binding Elongation factor Tu fan	430
Light-2	646	Background	AT1G07920	Symbols: | GTP binding Elongation factor Tu fan	430
Light-2	646	Background	AT1G07920	Symbols: | GTP binding Elongation factor Tu fan	430
Light-2	646	Background	AT1G07920	Symbols: | GTP binding Elongation factor Tu fan	430
Light-2	646	Background	AT1G07920	Symbols: | GTP binding Elongation factor Tu fan	430
Light-2	646	Background	AT1G07920	Symbols: | GTP binding Elongation factor Tu fan	430
Light-2	646	Background	AT1G07920	Symbols: | GTP binding Elongation factor Tu fan	430
Light-2	646	Background	AT1G07920	Symbols: | GTP binding Elongation factor Tu fan	430
Light-2	646	Background	AT1G07920	Symbols: | GTP binding Elongation factor Tu fan	430
Light-2	646	Background	AT1G07920	Symbols: | GTP binding Elongation factor Tu fan	430
Light-2	647	Background	AT4G35260	Symbols: IDH1, IDH-I | isocitrate dehydrogenase	426
Light-2	647	Background	AT4G35260	Symbols: IDH1, IDH-I | isocitrate dehydrogenase	426
Light-2	647	Background	AT4G35260	Symbols: IDH1, IDH-I | isocitrate dehydrogenase	426
Light-2	647	Background	AT4G35260	Symbols: IDH1, IDH-I | isocitrate dehydrogenase	426
Light-2	647	Background	AT4G35260	Symbols: IDH1, IDH-I | isocitrate dehydrogenase	426

Light-2	647	Background	AT4G35260	Symbols: IDH1, IDH-I | isocitrate dehydrogenase	426
Light-2	647	Background	AT4G35260	Symbols: IDH1, IDH-I | isocitrate dehydrogenase	426
Light-2	647	Background	AT4G35260	Symbols: IDH1, IDH-I | isocitrate dehydrogenase	426
Light-2	648	Background	AT5G03290	Symbols: IDH-V | isocitrate dehydrogenase V | c	403
Light-2	648	Background	AT5G03290	Symbols: IDH-V | isocitrate dehydrogenase V | c	403
Light-2	648	Background	AT5G03290	Symbols: IDH-V | isocitrate dehydrogenase V | c	403
Light-2	648	Background	AT5G03290	Symbols: IDH-V | isocitrate dehydrogenase V | c	403
Light-2	648	Background	AT5G03290	Symbols: IDH-V | isocitrate dehydrogenase V | c	403
Light-2	648	Background	AT5G03290	Symbols: IDH-V | isocitrate dehydrogenase V | c	403
Light-2	648	Background	AT5G03290	Symbols: IDH-V | isocitrate dehydrogenase V | c	403
Light-2	648	Background	AT5G03290	Symbols: IDH-V | isocitrate dehydrogenase V | c	403
Light-2	649	Background	AT5G09590	Symbols: MTHSC70-2, HSC70-5 | mitochondrial	395
Light-2	649	Background	AT5G09590	Symbols: MTHSC70-2, HSC70-5 | mitochondrial	395
Light-2	649	Background	AT5G09590	Symbols: MTHSC70-2, HSC70-5 | mitochondrial	395
Light-2	649	Background	AT5G09590	Symbols: MTHSC70-2, HSC70-5 | mitochondrial	395
Light-2	649	Background	AT5G09590	Symbols: MTHSC70-2, HSC70-5 | mitochondrial	395
Light-2	649	Background	AT5G09590	Symbols: MTHSC70-2, HSC70-5 | mitochondrial	395
Light-2	649	Background	AT5G09590	Symbols: MTHSC70-2, HSC70-5 | mitochondrial	395
Light-2	649	Background	AT5G09590	Symbols: MTHSC70-2, HSC70-5 | mitochondrial	395
Light-2	649	Background	AT5G09590	Symbols: MTHSC70-2, HSC70-5 | mitochondrial	395
Light-2	650	Background	AT4G37910	Symbols: mtHsc70-1 | mitochondrial heat shock	388
Light-2	650	Background	AT4G37910	Symbols: mtHsc70-1 | mitochondrial heat shock	388
Light-2	650	Background	AT4G37910	Symbols: mtHsc70-1 | mitochondrial heat shock	388
Light-2	650	Background	AT4G37910	Symbols: mtHsc70-1 | mitochondrial heat shock	388
Light-2	650	Background	AT4G37910	Symbols: mtHsc70-1 | mitochondrial heat shock	388
Light-2	650	Background	AT4G37910	Symbols: mtHsc70-1 | mitochondrial heat shock	388
Light-2	650	Background	AT4G37910	Symbols: mtHsc70-1 | mitochondrial heat shock	388
Light-2	650	Background	AT4G37910	Symbols: mtHsc70-1 | mitochondrial heat shock	388
Light-2	650	Background	AT4G37910	Symbols: mtHsc70-1 | mitochondrial heat shock	388
Light-2	651	Background	AT3G09810	Symbols: IDH-VI | isocitrate dehydrogenase VI |	369
Light-2	651	Background	AT3G09810	Symbols: IDH-VI | isocitrate dehydrogenase VI |	369
Light-2	651	Background	AT3G09810	Symbols: IDH-VI | isocitrate dehydrogenase VI |	369
Light-2	651	Background	AT3G09810	Symbols: IDH-VI | isocitrate dehydrogenase VI |	369
Light-2	651	Background	AT3G09810	Symbols: IDH-VI | isocitrate dehydrogenase VI |	369
Light-2	651	Background	AT3G09810	Symbols: IDH-VI | isocitrate dehydrogenase VI |	369
Light-2	651	Background	AT3G09810	Symbols: IDH-VI | isocitrate dehydrogenase VI |	369
Light-2	652	Background	AT2G02560	Symbols: CAND1, ATCAND1, ETA2, TIP120, HVE	338
Light-2	652	Background	AT2G02560	Symbols: CAND1, ATCAND1, ETA2, TIP120, HVE	338
Light-2	652	Background	AT2G02560	Symbols: CAND1, ATCAND1, ETA2, TIP120, HVE	338
Light-2	652	Background	AT2G02560	Symbols: CAND1, ATCAND1, ETA2, TIP120, HVE	338
Light-2	652	Background	AT2G02560	Symbols: CAND1, ATCAND1, ETA2, TIP120, HVE	338
Light-2	652	Background	AT2G02560	Symbols: CAND1, ATCAND1, ETA2, TIP120, HVE	338
Light-2	652	Background	AT2G02560	Symbols: CAND1, ATCAND1, ETA2, TIP120, HVE	338
Light-2	653	Background	AT2G39730	Symbols: RCA | rubisco activase | chr2:1657095	313
Light-2	653	Background	AT2G39730	Symbols: RCA | rubisco activase | chr2:1657095	313
Light-2	653	Background	AT2G39730	Symbols: RCA | rubisco activase | chr2:1657095	313
Light-2	653	Background	AT2G39730	Symbols: RCA | rubisco activase | chr2:1657095	313
Light-2	654	Background	AT1G24300	Symbols: | GYF domain-containing protein | chr	288
Light-2	654	Background	AT1G24300	Symbols: | GYF domain-containing protein | chr	288
Light-2	654	Background	AT1G24300	Symbols: | GYF domain-containing protein | chr	288
Light-2	654	Background	AT1G24300	Symbols: | GYF domain-containing protein | chr	288
Light-2	654	Background	AT1G24300	Symbols: | GYF domain-containing protein | chr	288
Light-2	654	Background	AT1G24300	Symbols: | GYF domain-containing protein | chr	288
Light-2	654	Background	AT1G24300	Symbols: | GYF domain-containing protein | chr	288
Light-2	655	Background	AT5G09810	Symbols: ACT7 | actin 7 | chr5:3052809-305422	281
Light-2	655	Background	AT5G09810	Symbols: ACT7 | actin 7 | chr5:3052809-305422	281
Light-2	655	Background	AT5G09810	Symbols: ACT7 | actin 7 | chr5:3052809-305422	281
Light-2	655	Background	AT5G09810	Symbols: ACT7 | actin 7 | chr5:3052809-305422	281

Light-2	655	Background	AT5G09810	Symbols: ACT7 | actin 7 | chr5:3052809-305422	281
Light-2	655	Background	AT5G09810	Symbols: ACT7 | actin 7 | chr5:3052809-305422	281
Light-2	655	Background	AT5G09810	Symbols: ACT7 | actin 7 | chr5:3052809-305422	281
Light-2	655	Background	AT5G09810	Symbols: ACT7 | actin 7 | chr5:3052809-305422	281
Light-2	656	Background	AT1G52670	Symbols: | Single hybrid motif superfamily prot	277
Light-2	656	Background	AT1G52670	Symbols: | Single hybrid motif superfamily prot	277
Light-2	656	Background	AT1G52670	Symbols: | Single hybrid motif superfamily prot	277
Light-2	657	Background	AT4G02930	Symbols: | GTP binding Elongation factor Tu fan	254
Light-2	657	Background	AT4G02930	Symbols: | GTP binding Elongation factor Tu fan	254
Light-2	657	Background	AT4G02930	Symbols: | GTP binding Elongation factor Tu fan	254
Light-2	657	Background	AT4G02930	Symbols: | GTP binding Elongation factor Tu fan	254
Light-2	657	Background	AT4G02930	Symbols: | GTP binding Elongation factor Tu fan	254
Light-2	658	Background	AT2G44060	Symbols: | Late embryogenesis abundant prote	248
Light-2	658	Background	AT2G44060	Symbols: | Late embryogenesis abundant prote	248
Light-2	658	Background	AT2G44060	Symbols: | Late embryogenesis abundant prote	248
Light-2	658	Background	AT2G44060	Symbols: | Late embryogenesis abundant prote	248
Light-2	658	Background	AT2G44060	Symbols: | Late embryogenesis abundant prote	248
Light-2	658	Background	AT2G44060	Symbols: | Late embryogenesis abundant prote	248
Light-2	659	Background	AT2G37270	Symbols: ATRPS5B, RPS5B | ribosomal protein 5	247
Light-2	659	Background	AT2G37270	Symbols: ATRPS5B, RPS5B | ribosomal protein 5	247
Light-2	659	Background	AT2G37270	Symbols: ATRPS5B, RPS5B | ribosomal protein 5	247
Light-2	659	Background	AT2G37270	Symbols: ATRPS5B, RPS5B | ribosomal protein 5	247
Light-2	660	Background	AT2G17130	Symbols: IDH2, IDH-II | isocitrate dehydrogenase	247
Light-2	660	Background	AT2G17130	Symbols: IDH2, IDH-II | isocitrate dehydrogenase	247
Light-2	660	Background	AT2G17130	Symbols: IDH2, IDH-II | isocitrate dehydrogenase	247
Light-2	660	Background	AT2G17130	Symbols: IDH2, IDH-II | isocitrate dehydrogenase	247
Light-2	660	Background	AT2G17130	Symbols: IDH2, IDH-II | isocitrate dehydrogenase	247
Light-2	660	Background	AT2G17130	Symbols: IDH2, IDH-II | isocitrate dehydrogenase	247
Light-2	661	Background	AT1G56070	Symbols: LOS1 | Ribosomal protein S5/Elongatic	245
Light-2	661	Background	AT1G56070	Symbols: LOS1 | Ribosomal protein S5/Elongatic	245
Light-2	661	Background	AT1G56070	Symbols: LOS1 | Ribosomal protein S5/Elongatic	245
Light-2	661	Background	AT1G56070	Symbols: LOS1 | Ribosomal protein S5/Elongatic	245
Light-2	662	Background	AT2G20140	Symbols: | AAA-type ATPase family protein | ch	241
Light-2	662	Background	AT2G20140	Symbols: | AAA-type ATPase family protein | ch	241
Light-2	662	Background	AT2G20140	Symbols: | AAA-type ATPase family protein | ch	241
Light-2	662	Background	AT2G20140	Symbols: | AAA-type ATPase family protein | ch	241
Light-2	663	Background	AT3G48870	Symbols: ATCLPC, ATHSP93-III, HSP93-III | Clp A	233
Light-2	663	Background	AT3G48870	Symbols: ATCLPC, ATHSP93-III, HSP93-III | Clp A	233
Light-2	663	Background	AT3G48870	Symbols: ATCLPC, ATHSP93-III, HSP93-III | Clp A	233
Light-2	663	Background	AT3G48870	Symbols: ATCLPC, ATHSP93-III, HSP93-III | Clp A	233
Light-2	663	Background	AT3G48870	Symbols: ATCLPC, ATHSP93-III, HSP93-III | Clp A	233
Light-2	663	Background	AT3G48870	Symbols: ATCLPC, ATHSP93-III, HSP93-III | Clp A	233
Light-2	663	Background	AT3G48870	Symbols: ATCLPC, ATHSP93-III, HSP93-III | Clp A	233
Light-2	663	Background	AT3G48870	Symbols: ATCLPC, ATHSP93-III, HSP93-III | Clp A	233
Light-2	664	Background	AT3G61650	Symbols: TUBG1 | gamma-tubulin | chr3:22812	226
Light-2	664	Background	AT3G61650	Symbols: TUBG1 | gamma-tubulin | chr3:22812	226
Light-2	664	Background	AT3G61650	Symbols: TUBG1 | gamma-tubulin | chr3:22812	226
Light-2	664	Background	AT3G61650	Symbols: TUBG1 | gamma-tubulin | chr3:22812	226
Light-2	664	Background	AT3G61650	Symbols: TUBG1 | gamma-tubulin | chr3:22812	226
Light-2	664	Background	AT3G61650	Symbols: TUBG1 | gamma-tubulin | chr3:22812	226
Light-2	665	Background	AT4G16143	Symbols: IMPA-2 | importin alpha isoform 2 | ch	209
Light-2	665	Background	AT4G16143	Symbols: IMPA-2 | importin alpha isoform 2 | ch	209
Light-2	665	Background	AT4G16143	Symbols: IMPA-2 | importin alpha isoform 2 | ch	209
Light-2	665	Background	AT4G16143	Symbols: IMPA-2 | importin alpha isoform 2 | ch	209
Light-2	665	Background	AT4G16143	Symbols: IMPA-2 | importin alpha isoform 2 | ch	209
Light-2	666	Background	AT5G20890	Symbols: | TCP-1/cpn60 chaperonin family prot	199
Light-2	666	Background	AT5G20890	Symbols: | TCP-1/cpn60 chaperonin family prot	199
Light-2	666	Background	AT5G20890	Symbols: | TCP-1/cpn60 chaperonin family prot	199

Light-2	666	Background	AT5G20890	Symbols: | TCP-1/cpn60 chaperonin family prot	199
Light-2	667	Background	AT1G72370	Symbols: P40, AP40, RP40, RPSAA | 40s ribosom	185
Light-2	667	Background	AT1G72370	Symbols: P40, AP40, RP40, RPSAA | 40s ribosom	185
Light-2	667	Background	AT1G72370	Symbols: P40, AP40, RP40, RPSAA | 40s ribosom	185
Light-2	667	Background	AT1G72370	Symbols: P40, AP40, RP40, RPSAA | 40s ribosom	185
Light-2	668	Background	AT5G28540	Symbols: BIP1 | heat shock protein 70 (Hsp 70) f	173
Light-2	668	Background	AT5G28540	Symbols: BIP1 | heat shock protein 70 (Hsp 70) f	173
Light-2	668	Background	AT5G28540	Symbols: BIP1 | heat shock protein 70 (Hsp 70) f	173
Light-2	668	Background	AT5G28540	Symbols: BIP1 | heat shock protein 70 (Hsp 70) f	173
Light-2	668	Background	AT5G28540	Symbols: BIP1 | heat shock protein 70 (Hsp 70) f	173
Light-2	669	Background	AT3G08580	Symbols: AAC1 | ADP/ATP carrier 1 | chr3:2605	171
Light-2	669	Background	AT3G08580	Symbols: AAC1 | ADP/ATP carrier 1 | chr3:2605	171
Light-2	669	Background	AT3G08580	Symbols: AAC1 | ADP/ATP carrier 1 | chr3:2605	171
Light-2	669	Background	AT3G08580	Symbols: AAC1 | ADP/ATP carrier 1 | chr3:2605	171
Light-2	669	Background	AT3G08580	Symbols: AAC1 | ADP/ATP carrier 1 | chr3:2605	171
Light-2	669	Background	AT3G08580	Symbols: AAC1 | ADP/ATP carrier 1 | chr3:2605	171
Light-2	669	Background	AT3G08580	Symbols: AAC1 | ADP/ATP carrier 1 | chr3:2605	171
Light-2	670	Background	AT2G37620	Symbols: ACT1, AAc1 | actin 1 | chr2:15779761-	169
Light-2	670	Background	AT2G37620	Symbols: ACT1, AAc1 | actin 1 | chr2:15779761-	169
Light-2	670	Background	AT2G37620	Symbols: ACT1, AAc1 | actin 1 | chr2:15779761-	169
Light-2	670	Background	AT2G37620	Symbols: ACT1, AAc1 | actin 1 | chr2:15779761-	169
Light-2	670	Background	AT2G37620	Symbols: ACT1, AAc1 | actin 1 | chr2:15779761-	169
Light-2	670	Background	AT2G37620	Symbols: ACT1, AAc1 | actin 1 | chr2:15779761-	169
Light-2	670	Background	AT2G37620	Symbols: ACT1, AAc1 | actin 1 | chr2:15779761-	169
Light-2	671	Background	AT5G13490	Symbols: AAC2 | ADP/ATP carrier 2 | chr5:4336	169
Light-2	671	Background	AT5G13490	Symbols: AAC2 | ADP/ATP carrier 2 | chr5:4336	169
Light-2	671	Background	AT5G13490	Symbols: AAC2 | ADP/ATP carrier 2 | chr5:4336	169
Light-2	671	Background	AT5G13490	Symbols: AAC2 | ADP/ATP carrier 2 | chr5:4336	169
Light-2	671	Background	AT5G13490	Symbols: AAC2 | ADP/ATP carrier 2 | chr5:4336	169
Light-2	671	Background	AT5G13490	Symbols: AAC2 | ADP/ATP carrier 2 | chr5:4336	169
Light-2	671	Background	AT5G13490	Symbols: AAC2 | ADP/ATP carrier 2 | chr5:4336	169
Light-2	672	Background	AT3G22330	Symbols: PMH2, ATRH53 | putative mitochondri	167
Light-2	672	Background	AT3G22330	Symbols: PMH2, ATRH53 | putative mitochondri	167
Light-2	672	Background	AT3G22330	Symbols: PMH2, ATRH53 | putative mitochondri	167
Light-2	672	Background	AT3G22330	Symbols: PMH2, ATRH53 | putative mitochondri	167
Light-2	672	Background	AT3G22330	Symbols: PMH2, ATRH53 | putative mitochondri	167
Light-2	672	Background	AT3G22330	Symbols: PMH2, ATRH53 | putative mitochondri	167
Light-2	673	Background	AT1G43800	Symbols: | Plant stearoyl-acyl-carrier-protein de	166
Light-2	673	Background	AT1G43800	Symbols: | Plant stearoyl-acyl-carrier-protein de	166
Light-2	673	Background	AT1G43800	Symbols: | Plant stearoyl-acyl-carrier-protein de	166
Light-2	673	Background	AT1G43800	Symbols: | Plant stearoyl-acyl-carrier-protein de	166
Light-2	674	Background	AT5G42020	Symbols: BIP, BIP2 | Heat shock protein 70 (Hsp	162
Light-2	674	Background	AT5G42020	Symbols: BIP, BIP2 | Heat shock protein 70 (Hsp	162
Light-2	674	Background	AT5G42020	Symbols: BIP, BIP2 | Heat shock protein 70 (Hsp	162
Light-2	674	Background	AT5G42020	Symbols: BIP, BIP2 | Heat shock protein 70 (Hsp	162
Light-2	674	Background	AT5G42020	Symbols: BIP, BIP2 | Heat shock protein 70 (Hsp	162
Light-2	675	Background	AT3G56130	Symbols: | biotin/lipoyl attachment domain-cor	160
Light-2	675	Background	AT3G56130	Symbols: | biotin/lipoyl attachment domain-cor	160
Light-2	675	Background	AT3G56130	Symbols: | biotin/lipoyl attachment domain-cor	160
Light-2	676	Background	AT5G08670	Symbols: | ATP synthase alpha/beta family prot	158
Light-2	676	Background	AT5G08670	Symbols: | ATP synthase alpha/beta family prot	158
Light-2	676	Background	AT5G08670	Symbols: | ATP synthase alpha/beta family prot	158
Light-2	676	Background	AT5G08670	Symbols: | ATP synthase alpha/beta family prot	158
Light-2	677	Background	AT3G23990	Symbols: HSP60, HSP60-3B | heat shock protein	154
Light-2	677	Background	AT3G23990	Symbols: HSP60, HSP60-3B | heat shock protein	154
Light-2	678	Background	AT3G17390	Symbols: MTO3, SAMS3, MAT4 | S-adenosylmet	150
Light-2	678	Background	AT3G17390	Symbols: MTO3, SAMS3, MAT4 | S-adenosylmet	150
Light-2	678	Background	AT3G17390	Symbols: MTO3, SAMS3, MAT4 | S-adenosylmet	150

Light-2	679	Background	AT5G43010	Symbols: RPT4A | regulatory particle triple-A AT	147
Light-2	679	Background	AT5G43010	Symbols: RPT4A | regulatory particle triple-A AT	147
Light-2	679	Background	AT5G43010	Symbols: RPT4A | regulatory particle triple-A AT	147
Light-2	679	Background	AT5G43010	Symbols: RPT4A | regulatory particle triple-A AT	147
Light-2	679	Background	AT5G43010	Symbols: RPT4A | regulatory particle triple-A AT	147
Light-2	680	Background	AT1G22780	Symbols: PFL, RPS18A, PFL1 | Ribosomal protein	147
Light-2	680	Background	AT1G22780	Symbols: PFL, RPS18A, PFL1 | Ribosomal protein	147
Light-2	681	Background	AT5G42950	Symbols: | GYF domain-containing protein | chr	146
Light-2	681	Background	AT5G42950	Symbols: | GYF domain-containing protein | chr	146
Light-2	682	Background	AT5G49910	Symbols: CPHSC70-2EAT SHOCK PROTEIN 70-2, I	145
Light-2	682	Background	AT5G49910	Symbols: CPHSC70-2EAT SHOCK PROTEIN 70-2, I	145
Light-2	682	Background	AT5G49910	Symbols: CPHSC70-2EAT SHOCK PROTEIN 70-2, I	145
Light-2	682	Background	AT5G49910	Symbols: CPHSC70-2EAT SHOCK PROTEIN 70-2, I	145
Light-2	682	Background	AT5G49910	Symbols: CPHSC70-2EAT SHOCK PROTEIN 70-2, I	145
Light-2	683	Background	AT1G09100	Symbols: RPT5B | 26S proteasome AAA-ATPase	145
Light-2	683	Background	AT1G09100	Symbols: RPT5B | 26S proteasome AAA-ATPase	145
Light-2	683	Background	AT1G09100	Symbols: RPT5B | 26S proteasome AAA-ATPase	145
Light-2	683	Background	AT1G09100	Symbols: RPT5B | 26S proteasome AAA-ATPase	145
Light-2	684	Background	AT5G56000	Symbols: Hsp81.4, AtHsp90.4 | HEAT SHOCK PR	139
Light-2	684	Background	AT5G56000	Symbols: Hsp81.4, AtHsp90.4 | HEAT SHOCK PR	139
Light-2	684	Background	AT5G56000	Symbols: Hsp81.4, AtHsp90.4 | HEAT SHOCK PR	139
Light-2	684	Background	AT5G56000	Symbols: Hsp81.4, AtHsp90.4 | HEAT SHOCK PR	139
Light-2	685	Background	AT2G31610	Symbols: | Ribosomal protein S3 family protein	134
Light-2	685	Background	AT2G31610	Symbols: | Ribosomal protein S3 family protein	134
Light-2	686	Background	AT5G24710	Symbols: | Transducin/WD40 repeat-like superf	133
Light-2	686	Background	AT5G24710	Symbols: | Transducin/WD40 repeat-like superf	133
Light-2	687	Background	AT2G41800	Symbols: | Protein of unknown function, DUF64	130
Light-2	687	Background	AT2G41800	Symbols: | Protein of unknown function, DUF64	130
Light-2	687	Background	AT2G41800	Symbols: | Protein of unknown function, DUF64	130
Light-2	688	Background	AT1G33120	Symbols: | Ribosomal protein L6 family | chr1:1	129
Light-2	688	Background	AT1G33120	Symbols: | Ribosomal protein L6 family | chr1:1	129
Light-2	688	Background	AT1G33120	Symbols: | Ribosomal protein L6 family | chr1:1	129
Light-2	688	Background	AT1G33120	Symbols: | Ribosomal protein L6 family | chr1:1	129
Light-2	689	Background	AT1G36180	Symbols: ACC2 | acetyl-CoA carboxylase 2 | chr1	127
Light-2	689	Background	AT1G36180	Symbols: ACC2 | acetyl-CoA carboxylase 2 | chr1	127
Light-2	689	Background	AT1G36180	Symbols: ACC2 | acetyl-CoA carboxylase 2 | chr1	127
Light-2	689	Background	AT1G36180	Symbols: ACC2 | acetyl-CoA carboxylase 2 | chr1	127
Light-2	690	Background	AT2G36620	Symbols: RPL24A | ribosomal protein L24 | chr2	126
Light-2	690	Background	AT2G36620	Symbols: RPL24A | ribosomal protein L24 | chr2	126
Light-2	691	Background	AT3G56150	Symbols: EIF3C, ATEIF3C-1, EIF3C-1, ATTIF3C1, T	125
Light-2	691	Background	AT3G56150	Symbols: EIF3C, ATEIF3C-1, EIF3C-1, ATTIF3C1, T	125
Light-2	691	Background	AT3G56150	Symbols: EIF3C, ATEIF3C-1, EIF3C-1, ATTIF3C1, T	125
Light-2	692	Background	AT5G19990	Symbols: RPT6A, ATSUG1 | regulatory particle tr	113
Light-2	692	Background	AT5G19990	Symbols: RPT6A, ATSUG1 | regulatory particle tr	113
Light-2	692	Background	AT5G19990	Symbols: RPT6A, ATSUG1 | regulatory particle tr	113
Light-2	692	Background	AT5G19990	Symbols: RPT6A, ATSUG1 | regulatory particle tr	113
Light-2	693	Background	AT3G52140	Symbols: | tetratricopeptide repeat (TPR)-conta	112
Light-2	693	Background	AT3G52140	Symbols: | tetratricopeptide repeat (TPR)-conta	112
Light-2	694	Background	AT2G24050	Symbols: eIFiso4G2 | MIF4G domain-containing	112
Light-2	694	Background	AT2G24050	Symbols: eIFiso4G2 | MIF4G domain-containing	112
Light-2	695	Background	AT3G05560	Symbols: | Ribosomal L22e protein family | chr	108
Light-2	695	Background	AT3G05560	Symbols: | Ribosomal L22e protein family | chr	108
Light-2	696	Background	AT2G20800	Symbols: NDB4 | NAD(P)H dehydrogenase B4 |	105
Light-2	696	Background	AT2G20800	Symbols: NDB4 | NAD(P)H dehydrogenase B4 |	105
Light-2	697	Background	AT1G64520	Symbols: RPN12a | regulatory particle non-ATPa	105
Light-2	697	Background	AT1G64520	Symbols: RPN12a | regulatory particle non-ATPa	105
Light-2	698	Background	AT1G29150	Symbols: ATS9, RPN6 | non-ATPase subunit 9 |	104
Light-2	698	Background	AT1G29150	Symbols: ATS9, RPN6 | non-ATPase subunit 9 |	104

Light-2	698	Background	AT1G29150	Symbols: ATS9, RPN6 | non-ATPase subunit 9 | c	104
Light-2	699	Table 1 Filtere	AT1G59990	Symbols: | DEA(D/H)-box RNA helicase family p	104
Light-2	699	Table 1 Filtere	AT1G59990	Symbols: | DEA(D/H)-box RNA helicase family p	104
Light-2	700	Background	AT2G36160	Symbols: | Ribosomal protein S11 family protei	104
Light-2	700	Background	AT2G36160	Symbols: | Ribosomal protein S11 family protei	104
Light-2	701	Background	AT3G05590	Symbols: RPL18 | ribosomal protein L18 | chr3:1	99
Light-2	701	Background	AT3G05590	Symbols: RPL18 | ribosomal protein L18 | chr3:1	99
Light-2	702	Background	AT2G20580	Symbols: RPN1A, ATRPN1A | 26S proteasome re	94
Light-2	702	Background	AT2G20580	Symbols: RPN1A, ATRPN1A | 26S proteasome re	94
Light-2	702	Background	AT2G20580	Symbols: RPN1A, ATRPN1A | 26S proteasome re	94
Light-2	703	Background	AT1G55490	Symbols: CPN60B, LEN1 | chaperonin 60 beta |	94
Light-2	703	Background	AT1G55490	Symbols: CPN60B, LEN1 | chaperonin 60 beta |	94
Light-2	704	Background	AT3G08530	Symbols: | Clathrin, heavy chain | chr3:258717	91
Light-2	704	Background	AT3G08530	Symbols: | Clathrin, heavy chain | chr3:258717	91
Light-2	705	Background	AT3G43300	Symbols: ATMIN7, BEN1 | HOPM interactor 7 |	91
Light-2	705	Background	AT3G43300	Symbols: ATMIN7, BEN1 | HOPM interactor 7 |	91
Light-2	706	Background	AT3G04840	Symbols: | Ribosomal protein S3Ae | chr3:1329	90
Light-2	706	Background	AT3G04840	Symbols: | Ribosomal protein S3Ae | chr3:1329	90
Light-2	707	Background	AT2G01250	Symbols: | Ribosomal protein L30/L7 family pro	85
Light-2	707	Background	AT2G01250	Symbols: | Ribosomal protein L30/L7 family pro	85
Light-2	707	Background	AT2G01250	Symbols: | Ribosomal protein L30/L7 family pro	85
Light-2	708	Background	AT3G12050	Symbols: | Aha1 domain-containing protein | ch	85
Light-2	708	Background	AT3G12050	Symbols: | Aha1 domain-containing protein | ch	85
Light-2	709	Background	AT3G14990	Symbols: | Class I glutamine amidotransferase-l	84
Light-2	709	Background	AT3G14990	Symbols: | Class I glutamine amidotransferase-l	84
Light-2	710	Background	AT3G63460	Symbols: | transducin family protein / WD-40 re	84
Light-2	710	Background	AT3G63460	Symbols: | transducin family protein / WD-40 re	84
Light-2	710	Background	AT3G63460	Symbols: | transducin family protein / WD-40 re	84
Light-2	711	Background	AT1G64790	Symbols: ILA | ILITYHIA | chr1:24065232-24081	82
Light-2	711	Background	AT1G64790	Symbols: ILA | ILITYHIA | chr1:24065232-24081	82
Light-2	712	Background	AT5G43780	Symbols: APS4 | Pseudouridine synthase/archae	79
Light-2	712	Background	AT5G43780	Symbols: APS4 | Pseudouridine synthase/archae	79
Light-2	713	Background	AT5G27770	Symbols: | Ribosomal L22e protein family | chr	78
Light-2	713	Background	AT5G27770	Symbols: | Ribosomal L22e protein family | chr	78
Light-2	714	Background	AT5G16390	Symbols: CAC1, CAC1A, BCCP, BCCP1 | chloropla	75
Light-2	714	Background	AT5G16390	Symbols: CAC1, CAC1A, BCCP, BCCP1 | chloropla	75
Light-2	714	Background	AT5G16390	Symbols: CAC1, CAC1A, BCCP, BCCP1 | chloropla	75
Light-2	715	Background	AT3G03110	Symbols: XPO1B, CRM1B | exportin 1B | chr3:70	65
Light-2	715	Background	AT3G03110	Symbols: XPO1B, CRM1B | exportin 1B | chr3:70	65
Light-2	716	Background	AT1G69840	Symbols: | SPFH/Band 7/PHB domain-containin	63
Light-2	716	Background	AT1G69840	Symbols: | SPFH/Band 7/PHB domain-containin	63
Light-2	717	Background	AT5G58290	Symbols: RPT3 | regulatory particle triple-A ATP	60
Light-2	717	Background	AT5G58290	Symbols: RPT3 | regulatory particle triple-A ATP	60
Light-2	718	Background	AT4G34030	Symbols: MCCB | 3-methylcrotonyl-CoA carboxy	55
Light-2	718	Background	AT4G34030	Symbols: MCCB | 3-methylcrotonyl-CoA carboxy	55
Light-2	719	Background	AT2G17360	Symbols: | Ribosomal protein S4 (RPS4A) family	50
Light-2	719	Background	AT2G17360	Symbols: | Ribosomal protein S4 (RPS4A) family	50

software (version 2.5.0, Matrix Science) combined with the Mascot search engine (version 2.4.1, Matrix Science) using the Masco

hold (high confidence, p < 0.01); **prot_sequences_sig**: number of significant protein sequences above threshold (high confidence,
logical assignment of peptides to proteins. The first time a peptide match to a query appears in the report, it is shown in bold face
are the most likely assignments. Conversely, a protein that does not contain any bold red matches is an intersection of proteins li
) between the experimental and calculated masses; **pep_start**: peptide start position in protein; **pep_end:** peptide end position in
ver this value, the more significant the result); **pep_res_before**: amino acid before peptide sequence; **pep_seq**: peptide sequence

prot_mass	prot_matches	prot_sequenc	prot_cover	pep_query	pep_rank	pep_isbold	pep_isunique	pep_exp_mz
51385	38	22	68.9	853	1	1	0	530.268
51385	38	22	68.9	1153	1	1	0	570.3504
51385	38	22	68.9	1178	1	1	0	573.7987
51385	38	22	68.9	1519	1	1	0	616.2913
51385	38	22	68.9	1759	1	1	0	644.3334
51385	38	22	68.9	1787	1	1	1	648.8072
51385	38	22	68.9	2119	1	1	0	692.8538
51385	38	22	68.9	2326	1	1	1	723.3277
51385	38	22	68.9	2329	1	1	0	723.8474
51385	38	22	68.9	2752	1	1	0	819.9279
51385	38	22	68.9	2835	1	1	0	840.4016
51385	38	22	68.9	2869	1	1	0	848.9189
51385	38	22	68.9	3055	1	1	1	889.9352
51385	38	22	68.9	3130	1	1	0	911.9653
51385	38	22	68.9	3247	1	1	1	936.9371
51385	38	22	68.9	3427	1	1	0	976.4478
51385	38	22	68.9	3461	1	1	0	987.0018
51385	38	22	68.9	3705	1	1	0	701.0359
51385	38	22	68.9	3743	1	1	0	709.3574
51385	38	22	68.9	4802	1	1	0	923.1117
51385	38	22	68.9	4907	1	1	1	934.7717
51385	38	22	68.9	5581	1	1	0	1133.8388
50361	36	20	63.3	580	1	1	1	488.7421
50361	36	20	63.3	750	1	1	0	514.282
50361	36	20	63.3	1153	1	0	0	570.3504
50361	36	20	63.3	1178	1	0	0	573.7987
50361	36	20	63.3	1519	1	0	0	616.2913
50361	36	20	63.3	1796	1	1	1	650.3141
50361	36	20	63.3	2241	1	1	0	709.8294
50361	36	20	63.3	2459	1	1	1	747.8315
50361	36	20	63.3	2752	1	0	0	819.9279
50361	36	20	63.3	2835	1	0	0	840.4016
50361	36	20	63.3	2869	1	0	0	848.9189
50361	36	20	63.3	3130	1	0	0	911.9653
50361	36	20	63.3	3427	1	0	0	976.4478
50361	36	20	63.3	3461	1	0	0	987.0018
50361	36	20	63.3	3705	1	0	0	701.0359
50361	36	20	63.3	3743	1	0	0	709.3574
50361	36	20	63.3	4407	1	1	0	1184.0669
50361	36	20	63.3	4792	1	1	0	922.1119
50361	36	20	63.3	4900	1	1	0	933.4519
50361	36	20	63.3	5308	1	1	1	1067.4951
51258	36	19	59.7	853	1	0	0	530.268
51258	36	19	59.7	1153	1	0	0	570.3504
51258	36	19	59.7	1178	1	0	0	573.7987
51258	36	19	59.7	1519	1	0	0	616.2913
51258	36	19	59.7	1759	1	0	0	644.3334
51258	36	19	59.7	2119	1	0	0	692.8538

51258	36	19	59.7	2281	1	1	0	716.3193
51258	36	19	59.7	2329	1	0	0	723.8474
51258	36	19	59.7	2752	1	0	0	819.9279
51258	36	19	59.7	2804	1	1	0	833.3938
51258	36	19	59.7	2869	1	0	0	848.9189
51258	36	19	59.7	3130	1	0	0	911.9653
51258	36	19	59.7	3427	1	0	0	976.4478
51258	36	19	59.7	3461	1	0	0	987.0018
51258	36	19	59.7	3705	1	0	0	701.0359
51258	36	19	59.7	3743	1	0	0	709.3574
51258	36	19	59.7	4802	1	0	0	923.1117
51258	36	19	59.7	4937	1	1	0	938.7838
51258	36	19	59.7	5581	1	0	0	1133.8388
51341	35	19	61.5	853	1	0	0	530.268
51341	35	19	61.5	1153	1	0	0	570.3504
51341	35	19	61.5	1178	1	0	0	573.7987
51341	35	19	61.5	1519	1	0	0	616.2913
51341	35	19	61.5	1759	1	0	0	644.3334
51341	35	19	61.5	1835	1	1	1	654.8245
51341	35	19	61.5	2241	1	0	0	709.8294
51341	35	19	61.5	2281	1	0	0	716.3193
51341	35	19	61.5	2329	1	0	0	723.8474
51341	35	19	61.5	2752	1	0	0	819.9279
51341	35	19	61.5	2804	1	0	0	833.3938
51341	35	19	61.5	2869	1	0	0	848.9189
51341	35	19	61.5	3331	1	1	1	950.4707
51341	35	19	61.5	3427	1	0	0	976.4478
51341	35	19	61.5	3461	1	0	0	987.0018
51341	35	19	61.5	3705	1	0	0	701.0359
51341	35	19	61.5	4739	1	1	1	913.1079
51341	35	19	61.5	4937	1	0	0	938.7838
51341	35	19	61.5	5581	1	0	0	1133.8388
71712	30	21	51.6	661	1	1	0	501.7821
71712	30	21	51.6	1453	1	1	0	608.3289
71712	30	21	51.6	1506	1	1	0	614.8175
71712	30	21	51.6	1779	1	1	0	647.8162
71712	30	21	51.6	1898	1	1	0	665.3107
71712	30	21	51.6	1999	1	1	0	679.811
71712	30	21	51.6	2127	1	1	0	693.8677
71712	30	21	51.6	2269	1	1	1	713.8857
71712	30	21	51.6	2300	1	1	0	718.8802
71712	30	21	51.6	2399	1	1	0	737.3462
71712	30	21	51.6	2575	1	1	0	770.8823
71712	30	21	51.6	2629	1	1	1	783.9082
71712	30	21	51.6	2798	1	1	0	832.4088
71712	30	21	51.6	2815	1	1	0	838.368
71712	30	21	51.6	2838	1	1	0	840.92
71712	30	21	51.6	4573	1	1	1	1280.6295
71712	30	21	51.6	4604	1	1	0	863.4107
71712	30	21	51.6	4646	1	1	0	1329.6379
71712	30	21	51.6	4795	1	1	1	922.3841
71712	30	21	51.6	5153	1	1	1	1009.5041
71712	30	21	51.6	5161	1	1	1	1017.9042
47075	25	18	55.1	489	1	1	1	475.7505
47075	25	18	55.1	518	1	1	0	479.7633
47075	25	18	55.1	719	1	1	0	509.2767
47075	25	18	55.1	1060	1	1	0	557.8452
47075	25	18	55.1	1166	1	1	0	571.7902

47075	25	18	55.1	1294	1	1	0	587.3276
47075	25	18	55.1	2184	1	1	0	701.3698
47075	25	18	55.1	2333	1	1	1	724.3795
47075	25	18	55.1	2487	1	1	0	751.943
47075	25	18	55.1	2643	1	1	1	788.4276
47075	25	18	55.1	2666	1	1	0	794.3545
47075	25	18	55.1	3059	1	1	0	595.5913
47075	25	18	55.1	3145	1	1	0	914.4724
47075	25	18	55.1	3370	1	1	0	961.0045
47075	25	18	55.1	3556	1	1	0	676.636
47075	25	18	55.1	3625	1	1	0	1030.0478
47075	25	18	55.1	5043	1	1	0	965.838
47075	25	18	55.1	5246	1	1	1	1046.5422
50191	27	16	60.2	366	1	1	0	452.2172
50191	27	16	60.2	603	1	1	0	494.2769
50191	27	16	60.2	1745	1	1	0	642.2557
50191	27	16	60.2	2403	1	1	0	737.4358
50191	27	16	60.2	2681	1	1	0	799.887
50191	27	16	60.2	2858	1	1	1	845.9465
50191	27	16	60.2	2883	1	1	0	851.4559
50191	27	16	60.2	3069	1	1	0	896.9681
50191	27	16	60.2	3216	1	1	0	929.445
50191	27	16	60.2	3286	1	1	0	943.4761
50191	27	16	60.2	3468	1	1	0	989.4454
50191	27	16	60.2	4374	1	1	0	782.6751
50191	27	16	60.2	4442	1	1	0	795.7358
50191	27	16	60.2	4462	1	1	0	1198.0994
50191	27	16	60.2	4634	1	1	0	881.0804
50191	27	16	60.2	5300	1	1	0	1065.2149
50194	26	16	60.2	366	1	0	0	452.2172
50194	26	16	60.2	603	1	0	0	494.2769
50194	26	16	60.2	1745	1	0	0	642.2557
50194	26	16	60.2	2403	1	0	0	737.4358
50194	26	16	60.2	2681	1	0	0	799.887
50194	26	16	60.2	2799	1	1	1	832.4387
50194	26	16	60.2	2883	1	0	0	851.4559
50194	26	16	60.2	3069	1	0	0	896.9681
50194	26	16	60.2	3216	1	0	0	929.445
50194	26	16	60.2	3286	1	0	0	943.4761
50194	26	16	60.2	3468	1	0	0	989.4454
50194	26	16	60.2	4374	1	0	0	782.6751
50194	26	16	60.2	4442	1	0	0	795.7358
50194	26	16	60.2	4462	1	0	0	1198.0994
50194	26	16	60.2	4634	1	0	0	881.0804
50194	26	16	60.2	5300	1	0	0	1065.2149
46960	24	18	54.1	445	1	1	0	468.2633
46960	24	18	54.1	518	1	0	0	479.7633
46960	24	18	54.1	582	1	1	1	488.7823
46960	24	18	54.1	719	1	0	0	509.2767
46960	24	18	54.1	1027	1	1	1	552.8295
46960	24	18	54.1	1060	1	0	0	557.8452
46960	24	18	54.1	1166	1	0	0	571.7902
46960	24	18	54.1	1294	1	0	0	587.3276
46960	24	18	54.1	2184	1	0	0	701.3698
46960	24	18	54.1	2487	1	0	0	751.943
46960	24	18	54.1	2666	1	0	0	794.3545
46960	24	18	54.1	3059	1	0	0	595.5913
46960	24	18	54.1	3145	1	0	0	914.4724

46960	24	18	54.1	3370	1	0	0	961.0045
46960	24	18	54.1	3556	1	0	0	676.636
46960	24	18	54.1	3625	1	0	0	1030.0478
46960	24	18	54.1	5043	1	0	0	965.838
71559	24	20	43.9	661	1	0	0	501.7821
71559	24	20	43.9	1453	1	0	0	608.3289
71559	24	20	43.9	1495	1	1	1	613.7989
71559	24	20	43.9	1506	1	0	0	614.8175
71559	24	20	43.9	1779	1	0	0	647.8162
71559	24	20	43.9	1784	1	1	1	648.3352
71559	24	20	43.9	1840	1	1	1	654.8559
71559	24	20	43.9	1999	1	0	0	679.811
71559	24	20	43.9	2127	1	0	0	693.8677
71559	24	20	43.9	2210	1	1	1	704.3851
71559	24	20	43.9	2282	1	1	1	716.3751
71559	24	20	43.9	2399	1	0	0	737.3462
71559	24	20	43.9	2529	1	1	1	508.272
71559	24	20	43.9	2815	1	0	0	838.368
71559	24	20	43.9	2819	1	1	1	839.4158
71559	24	20	43.9	2838	1	0	0	840.92
71559	24	20	43.9	4580	1	1	0	1287.6362
71559	24	20	43.9	4604	1	0	0	863.4107
71559	24	20	43.9	4646	1	0	0	1329.6379
71559	24	20	43.9	5115	1	1	1	1488.7455
58863	27	20	56.2	343	1	1	1	446.7606
58863	27	20	56.2	441	1	1	1	467.2376
58863	27	20	56.2	510	1	1	1	478.2898
58863	27	20	56.2	986	1	1	1	546.3025
58863	27	20	56.2	1244	1	1	1	581.2937
58863	27	20	56.2	1253	1	1	1	582.3243
58863	27	20	56.2	1392	1	1	1	600.324
58863	27	20	56.2	1912	1	1	1	667.3627
58863	27	20	56.2	2104	1	1	1	691.7974
58863	27	20	56.2	2154	1	1	1	697.3771
58863	27	20	56.2	2698	1	1	1	805.4427
58863	27	20	56.2	2779	1	1	1	826.4125
58863	27	20	56.2	2884	1	1	1	851.8722
58863	27	20	56.2	2926	1	1	1	860.941
58863	27	20	56.2	3458	1	1	1	986.0302
58863	27	20	56.2	3920	1	1	1	1099.057
58863	27	20	56.2	4635	1	1	1	881.1227
58863	27	20	56.2	4696	1	1	1	902.4041
58863	27	20	56.2	4847	1	1	1	928.4688
58863	27	20	56.2	5272	1	1	1	1053.2119
51237	23	15	49.9	669	1	1	1	502.7449
51237	23	15	49.9	853	1	0	0	530.268
51237	23	15	49.9	1153	1	0	0	570.3504
51237	23	15	49.9	1178	1	0	0	573.7987
51237	23	15	49.9	1519	1	0	0	616.2913
51237	23	15	49.9	1773	1	1	1	647.3247
51237	23	15	49.9	2119	1	0	0	692.8538
51237	23	15	49.9	2281	1	0	0	716.3193
51237	23	15	49.9	2329	1	0	0	723.8474
51237	23	15	49.9	2752	1	0	0	819.9279
51237	23	15	49.9	2869	1	0	0	848.9189
51237	23	15	49.9	3431	1	1	0	653.0057
51237	23	15	49.9	4802	1	0	0	923.1117
51237	23	15	49.9	4958	1	1	1	943.4609

51237	23	15	49.9	5581	1	0	0	1133.8388
71741	18	14	32.3	661	1	0	0	501.7821
71741	18	14	32.3	1048	1	1	1	556.2539
71741	18	14	32.3	1385	1	1	1	599.351
71741	18	14	32.3	1506	1	0	0	614.8175
71741	18	14	32.3	1779	1	0	0	647.8162
71741	18	14	32.3	1999	1	0	0	679.811
71741	18	14	32.3	2300	1	0	0	718.8802
71741	18	14	32.3	2399	1	0	0	737.3462
71741	18	14	32.3	2798	1	0	0	832.4088
71741	18	14	32.3	2815	1	0	0	838.368
71741	18	14	32.3	2838	1	0	0	840.92
71741	18	14	32.3	4580	1	0	0	1287.6362
71741	18	14	32.3	4604	1	0	0	863.4107
71741	18	14	32.3	5260	1	1	1	1050.8348
47083	17	13	35.3	80	1	1	1	381.7288
47083	17	13	35.3	445	1	0	0	468.2633
47083	17	13	35.3	518	1	0	0	479.7633
47083	17	13	35.3	719	1	0	0	509.2767
47083	17	13	35.3	1060	1	0	0	557.8452
47083	17	13	35.3	1294	1	0	0	587.3276
47083	17	13	35.3	2184	1	0	0	701.3698
47083	17	13	35.3	2666	1	0	0	794.3545
47083	17	13	35.3	3059	1	0	0	595.5913
47083	17	13	35.3	3145	1	0	0	914.4724
47083	17	13	35.3	3370	1	0	0	961.0045
47083	17	13	35.3	3556	1	0	0	676.636
47083	17	13	35.3	5043	1	0	0	965.838
50250	18	15	55.8	366	1	0	0	452.2172
50250	18	15	55.8	603	1	0	0	494.2769
50250	18	15	55.8	1745	1	0	0	642.2557
50250	18	15	55.8	2550	1	1	1	766.4565
50250	18	15	55.8	2659	1	1	1	792.879
50250	18	15	55.8	2860	1	1	1	846.4379
50250	18	15	55.8	2883	1	0	0	851.4559
50250	18	15	55.8	2981	1	1	1	584.3112
50250	18	15	55.8	3280	1	1	0	942.9509
50250	18	15	55.8	3286	1	0	0	943.4761
50250	18	15	55.8	4374	1	0	0	782.6751
50250	18	15	55.8	4442	1	0	0	795.7358
50250	18	15	55.8	4479	1	0	0	1205.1077
50250	18	15	55.8	4684	1	1	1	899.0957
50250	18	15	55.8	5300	1	0	0	1065.2149
73315	14	12	25.8	1582	1	1	1	624.8452
73315	14	12	25.8	1583	1	1	1	625.286
73315	14	12	25.8	1603	1	1	0	628.813
73315	14	12	25.8	1776	1	1	1	647.794
73315	14	12	25.8	1891	1	1	1	664.3221
73315	14	12	25.8	2019	1	1	1	681.3745
73315	14	12	25.8	2288	1	1	0	717.3689
73315	14	12	25.8	2418	1	1	1	739.3852
73315	14	12	25.8	3111	1	1	1	907.4811
73315	14	12	25.8	3129	1	1	1	911.9539
73315	14	12	25.8	3178	1	1	1	920.4485
73315	14	12	25.8	5187	1	1	1	1026.828
37004	12	10	42.9	102	1	1	1	387.7213
37004	12	10	42.9	1074	1	1	1	560.2644
37004	12	10	42.9	1159	1	1	1	570.8256

37004	12	10	42.9	2291	1	1	1	717.8822
37004	12	10	42.9	2470	1	1	1	749.9274
37004	12	10	42.9	2818	1	1	1	559.6625
37004	12	10	42.9	2997	1	1	1	881.3965
37004	12	10	42.9	3570	1	1	1	678.3622
37004	12	10	42.9	3864	1	1	1	1086.5077
37004	12	10	42.9	4068	1	1	1	1125.5383
136636	14	12	12.4	245	1	1	1	424.753
136636	14	12	12.4	566	1	1	1	486.7714
136636	14	12	12.4	611	1	1	1	494.7845
136636	14	12	12.4	1202	1	1	1	576.8037
136636	14	12	12.4	1674	1	1	1	635.3619
136636	14	12	12.4	1696	1	1	1	638.8286
136636	14	12	12.4	2205	1	1	1	469.6082
136636	14	12	12.4	2393	1	1	1	734.8947
136636	14	12	12.4	2472	1	1	1	750.3819
136636	14	12	12.4	2631	1	1	1	784.9199
136636	14	12	12.4	3447	1	1	1	983.0143
136636	14	12	12.4	4038	1	1	1	1121.1202
41112	11	9	31	129	1	1	1	397.2262
41112	11	9	31	177	1	1	0	408.7504
41112	11	9	31	1301	1	1	0	587.8106
41112	11	9	31	2423	1	1	0	740.3933
41112	11	9	31	2509	1	1	1	758.3613
41112	11	9	31	2843	1	1	1	841.9199
41112	11	9	31	3150	1	1	1	915.5041
41112	11	9	31	3195	1	1	1	923.9311
41112	11	9	31	3837	1	1	0	720.0324
41937	12	11	43.2	144	1	1	0	400.7712
41937	12	11	43.2	579	1	1	0	488.7275
41937	12	11	43.2	1251	1	1	0	582.3021
41937	12	11	43.2	1360	1	1	0	596.7681
41937	12	11	43.2	1387	1	1	0	599.8561
41937	12	11	43.2	2405	1	1	1	738.3409
41937	12	11	43.2	3050	1	1	0	887.9513
41937	12	11	43.2	3212	1	1	1	928.4695
41937	12	11	43.2	3926	1	1	1	1100.0402
41937	12	11	43.2	5262	1	1	0	1051.2183
51883	13	9	27.3	999	1	1	1	548.3262
51883	13	9	27.3	1226	1	1	1	579.3293
51883	13	9	27.3	2137	1	1	1	695.3387
51883	13	9	27.3	2295	1	1	1	718.3387
51883	13	9	27.3	2842	1	1	1	841.4774
51883	13	9	27.3	3039	1	1	0	590.2672
51883	13	9	27.3	3146	1	1	1	914.986
51883	13	9	27.3	3344	1	1	1	954.029
51883	13	9	27.3	3768	1	1	1	1068.0728
105902	16	15	19.7	139	1	1	1	399.7637
105902	16	15	19.7	567	1	1	1	486.7763
105902	16	15	19.7	753	1	1	0	515.2877
105902	16	15	19.7	781	1	1	0	519.7675
105902	16	15	19.7	828	1	1	1	525.77
105902	16	15	19.7	1067	1	1	0	558.7985
105902	16	15	19.7	1230	1	1	0	579.8212
105902	16	15	19.7	1233	1	1	0	580.3006
105902	16	15	19.7	2167	1	1	0	699.3721
105902	16	15	19.7	2222	1	1	1	706.3294
105902	16	15	19.7	2416	1	1	0	738.8953

105902	16	15	19.7	2502	1	1	0	756.8659
105902	16	15	19.7	3308	1	1	1	946.5273
105902	16	15	19.7	4072	1	1	1	751.3725
105902	16	15	19.7	4546	1	1	0	836.7989
10285	10	5	54.6	412	1	1	1	460.7462
10285	10	5	54.6	464	1	1	1	471.2583
10285	10	5	54.6	2310	1	1	1	720.3467
10285	10	5	54.6	2510	1	1	1	758.3674
10285	10	5	54.6	3783	1	1	1	1071.5077
49813	16	11	35.2	396	1	1	1	457.787
49813	16	11	35.2	685	1	1	1	336.5221
49813	16	11	35.2	739	1	1	1	513.3082
49813	16	11	35.2	741	1	1	1	513.748
49813	16	11	35.2	1079	1	1	1	560.8029
49813	16	11	35.2	1793	1	1	1	649.877
49813	16	11	35.2	2346	1	1	1	726.3397
49813	16	11	35.2	2744	1	1	1	545.6383
49813	16	11	35.2	4326	1	1	1	771.3808
49813	16	11	35.2	4565	1	1	1	847.1118
49813	16	11	35.2	5108	1	1	1	991.812
36185	12	8	31.4	468	1	1	1	472.2582
36185	12	8	31.4	898	1	1	1	535.8267
36185	12	8	31.4	1816	1	1	1	652.3788
36185	12	8	31.4	1842	1	1	1	655.8136
36185	12	8	31.4	2095	1	1	1	690.8264
36185	12	8	31.4	2507	1	1	1	505.284
36185	12	8	31.4	2984	1	1	1	877.4324
36185	12	8	31.4	4342	1	1	1	774.4093
29784	8	5	37.2	1558	1	1	1	621.8285
29784	8	5	37.2	3392	1	1	1	968.0192
29784	8	5	37.2	4495	1	1	1	1216.122
29784	8	5	37.2	4531	1	1	1	1242.6153
29784	8	5	37.2	5083	1	1	1	982.8304
65547	13	12	28.2	524	1	1	1	481.2133
65547	13	12	28.2	1116	1	1	1	566.2389
65547	13	12	28.2	1232	1	1	0	580.2545
65547	13	12	28.2	1347	1	1	0	594.3549
65547	13	12	28.2	1446	1	1	1	607.775
65547	13	12	28.2	1628	1	1	1	631.3428
65547	13	12	28.2	2475	1	1	1	750.8462
65547	13	12	28.2	2589	1	1	1	773.8957
65547	13	12	28.2	2682	1	1	1	533.6144
65547	13	12	28.2	3119	1	1	1	908.4595
65547	13	12	28.2	4480	1	1	0	1205.5711
65547	13	12	28.2	4601	1	1	1	862.1195
40949	8	7	25.4	177	1	0	0	408.7504
40949	8	7	25.4	1301	1	0	0	587.8106
40949	8	7	25.4	2423	1	0	0	740.3933
40949	8	7	25.4	2509	1	0	1	758.3613
40949	8	7	25.4	2934	1	1	1	863.8911
40949	8	7	25.4	3087	1	1	1	900.9719
40949	8	7	25.4	3837	1	0	0	720.0324
41563	9	6	18.9	96	1	1	0	386.2272
41563	9	6	18.9	406	1	1	0	459.2945
41563	9	6	18.9	1426	1	1	1	604.3196
41563	9	6	18.9	2014	1	1	1	680.8599
41563	9	6	18.9	2331	1	1	0	723.8744
41563	9	6	18.9	3600	1	1	1	1024.5117

166282	11	11	10.2	453	1	1	1	469.7503
166282	11	11	10.2	1150	1	1	0	570.2931
166282	11	11	10.2	1297	1	1	1	587.3398
166282	11	11	10.2	1304	1	1	1	588.33
166282	11	11	10.2	1855	1	1	1	658.3528
166282	11	11	10.2	1983	1	1	1	677.8331
166282	11	11	10.2	3252	1	1	1	937.4266
166282	11	11	10.2	3430	1	1	1	652.9962
166282	11	11	10.2	3486	1	1	1	993.9756
166282	11	11	10.2	3851	1	1	1	723.3322
166282	11	11	10.2	4429	1	1	0	793.4133
73174	10	10	17.2	268	1	1	1	429.2637
73174	10	10	17.2	481	1	1	1	474.256
73174	10	10	17.2	1114	1	1	1	565.788
73174	10	10	17.2	1355	1	1	1	595.8429
73174	10	10	17.2	1532	1	1	1	618.2912
73174	10	10	17.2	1603	1	0	0	628.813
73174	10	10	17.2	2239	1	1	1	709.3668
73174	10	10	17.2	2288	1	0	0	717.3689
73174	10	10	17.2	2703	1	1	1	538.63
73174	10	10	17.2	3128	1	1	1	911.4579
40001	8	8	31.3	1368	1	1	0	597.3351
40001	8	8	31.3	1458	1	1	0	609.3064
40001	8	8	31.3	1738	1	1	1	640.8188
40001	8	8	31.3	2256	1	1	0	712.3353
40001	8	8	31.3	2430	1	1	1	742.4107
40001	8	8	31.3	2663	1	1	1	793.941
40001	8	8	31.3	4180	1	1	1	1138.5902
40001	8	8	31.3	4532	1	1	0	829.4093
47236	7	6	18.4	1231	1	1	0	579.829
47236	7	6	18.4	1264	1	1	0	584.2725
47236	7	6	18.4	1377	1	1	0	598.283
47236	7	6	18.4	1783	1	1	0	648.3247
47236	7	6	18.4	3287	1	1	0	943.9538
47236	7	6	18.4	3481	1	1	1	992.4687
52347	4	4	17.3	2870	1	1	1	849.3856
52347	4	4	17.3	3804	1	1	1	1075.5688
52347	4	4	17.3	4376	1	1	1	783.0671
52347	4	4	17.3	4951	1	1	1	941.8224
77064	8	8	13.5	299	1	1	0	435.7737
77064	8	8	13.5	397	1	1	0	458.2482
77064	8	8	13.5	696	1	1	1	505.8033
77064	8	8	13.5	2102	1	1	0	691.3322
77064	8	8	13.5	2378	1	1	0	731.3889
77064	8	8	13.5	2650	1	1	0	790.4092
77064	8	8	13.5	2983	1	1	1	876.4508
77064	8	8	13.5	3198	1	1	0	617.0069
42056	7	7	27.3	144	1	0	0	400.7712
42056	7	7	27.3	579	1	0	0	488.7275
42056	7	7	27.3	1251	1	0	0	582.3021
42056	7	7	27.3	1387	1	0	0	599.8561
42056	7	7	27.3	3066	1	1	1	895.9492
42056	7	7	27.3	3456	1	0	1	657.0332
42056	7	7	27.3	5262	1	0	0	1051.2183
98766	7	7	11.1	1218	1	1	1	578.7748
98766	7	7	11.1	1662	1	1	1	634.3086
98766	7	7	11.1	1920	1	1	1	668.841
98766	7	7	11.1	2193	1	1	0	702.4367

98766	7	7	11.1	2917	1	1	1	858.5213
98766	7	7	11.1	3616	1	1	1	1027.5264
98766	7	7	11.1	3727	1	1	1	704.689
31671	6	6	26.2	594	1	1	1	492.2529
31671	6	6	26.2	1378	1	1	1	598.2955
31671	6	6	26.2	1848	1	1	1	657.3209
31671	6	6	26.2	2387	1	1	1	733.393
31671	6	6	26.2	2697	1	1	1	805.4064
31671	6	6	26.2	3425	1	1	1	975.4651
103283	4	4	5.6	727	1	1	1	510.2595
103283	4	4	5.6	1748	1	1	1	642.315
103283	4	4	5.6	2382	1	1	1	731.8884
103283	4	4	5.6	3165	1	1	1	918.9736
120769	6	6	7	1524	1	1	1	617.316
120769	6	6	7	1614	1	1	1	629.8552
120769	6	6	7	1708	1	1	1	639.3404
120769	6	6	7	1741	1	1	1	641.3161
120769	6	6	7	2608	1	1	1	778.4381
120769	6	6	7	2614	1	1	1	780.3881
27673	4	3	16.8	1762	1	1	1	644.8374
27673	4	3	16.8	2255	1	1	1	712.3347
27673	4	3	16.8	3408	1	1	1	971.9988
49607	5	4	13.2	1445	1	1	1	607.3429
49607	5	4	13.2	2667	1	1	1	794.8994
49607	5	4	13.2	3039	1	0	0	590.2672
49607	5	4	13.2	3760	1	1	1	1065.0404
73187	4	3	6.1	1320	1	1	1	591.3666
73187	4	3	6.1	1528	1	1	1	617.3513
73187	4	3	6.1	3275	1	1	1	941.5558
64169	5	5	12	1236	1	1	0	580.798
64169	5	5	12	1393	1	1	1	600.3354
64169	5	5	12	1740	1	1	0	640.8872
64169	5	5	12	2876	1	1	0	850.9528
64169	5	5	12	3333	1	1	0	950.5214
39964	5	5	18.8	1368	1	0	0	597.3351
39964	5	5	18.8	1458	1	0	0	609.3064
39964	5	5	18.8	2256	1	0	0	712.3353
39964	5	5	18.8	2395	1	1	1	735.4067
39964	5	5	18.8	4532	1	0	0	829.4093
109501	3	3	5.6	2970	1	1	1	874.4514
109501	3	3	5.6	3361	1	1	1	957.9862
109501	3	3	5.6	4512	1	1	1	1231.1436
23090	4	4	23.2	645	1	1	0	499.7851
23090	4	4	23.2	1367	1	1	0	398.544
23090	4	4	23.2	2192	1	1	1	702.4038
23090	4	4	23.2	2734	1	1	0	816.4186
32499	4	4	21.1	460	1	1	1	470.795
32499	4	4	21.1	3239	1	1	1	933.0275
32499	4	4	21.1	3664	1	1	1	694.6491
32499	4	4	21.1	3911	1	1	1	1097.0179
63627	5	5	12.1	1236	1	0	0	580.798
63627	5	5	12.1	1514	1	1	1	615.3529
63627	5	5	12.1	1740	1	0	0	640.8872
63627	5	5	12.1	2876	1	0	0	850.9528
63627	5	5	12.1	3333	1	0	0	950.5214
94743	4	4	7.7	2352	1	1	1	726.8488
94743	4	4	7.7	2524	1	1	1	759.8389
94743	4	4	7.7	3085	1	1	1	900.4693

94743	4	4	7.7	4099	1	1	1	1129.1045
63798	7	7	16.9	1232	1	0	0	580.2545
63798	7	7	16.9	1347	1	0	0	594.3549
63798	7	7	16.9	1688	1	1	1	637.8403
63798	7	7	16.9	2431	1	1	1	495.5685
63798	7	7	16.9	2570	1	1	1	513.2853
63798	7	7	16.9	3076	1	1	1	898.8766
63798	7	7	16.9	4480	1	0	0	1205.5711
137407	4	4	4.8	2469	1	1	1	748.9221
137407	4	4	4.8	2583	1	1	1	772.8988
137407	4	4	4.8	2709	1	1	1	809.9285
137407	4	4	4.8	3358	1	1	1	957.077
49487	3	2	6.1	1541	1	1	1	619.3299
49487	3	2	6.1	2982	1	1	1	876.4356
100826	4	3	4.8	1801	1	1	1	651.3293
100826	4	3	4.8	2582	1	1	1	515.2837
100826	4	3	4.8	3590	1	1	1	1023.0549
41834	6	6	15.3	96	1	0	0	386.2272
41834	6	6	15.3	406	1	0	0	459.2945
41834	6	6	15.3	494	1	1	1	476.248
41834	6	6	15.3	1505	1	1	1	614.8141
41834	6	6	15.3	2014	1	0	1	680.8599
41834	6	6	15.3	2331	1	0	0	723.8744
22118	6	5	34.5	352	1	1	1	449.7531
22118	6	5	34.5	1282	1	1	1	585.8055
22118	6	5	34.5	2652	1	1	1	791.9304
22118	6	5	34.5	3068	1	1	1	598.305
22118	6	5	34.5	3240	1	1	1	933.4741
44356	5	5	19.2	14	1	1	1	355.7009
44356	5	5	19.2	339	1	1	1	443.243
44356	5	5	19.2	1730	1	1	1	639.8163
44356	5	5	19.2	3930	1	1	1	1101.5532
44356	5	5	19.2	5506	1	1	1	1125.1973
59765	3	3	7.7	2266	1	1	1	713.3907
59765	3	3	7.7	2455	1	1	1	746.8905
59765	3	3	7.7	3235	1	1	1	932.9756
86221	3	3	5.5	746	1	1	1	513.8011
86221	3	3	5.5	1588	1	1	1	625.8224
86221	3	3	5.5	4316	1	1	1	1154.5809
72129	3	2	5.1	3046	1	1	1	886.0202
72129	3	2	5.1	3378	1	1	1	963.0168
53612	5	5	15.8	786	1	1	1	520.3113
53612	5	5	15.8	1581	1	1	1	624.8201
53612	5	5	15.8	1881	1	1	1	662.3357
53612	5	5	15.8	4338	1	1	1	1160.6032
53612	5	5	15.8	4642	1	1	1	885.1405
615675	5	5	1.5	2248	1	1	1	710.8935
615675	5	5	1.5	2580	1	1	1	771.4275
615675	5	5	1.5	2715	1	1	1	810.4536
615675	5	5	1.5	3610	1	1	1	1027.5091
615675	5	5	1.5	4873	1	1	1	931.1344
44425	3	3	12.9	584	1	1	1	489.7662
44425	3	3	12.9	2548	1	1	1	766.366
44425	3	3	12.9	5217	1	1	1	1037.1788
40630	5	5	15.9	654	1	1	1	500.7922
40630	5	5	15.9	1677	1	1	1	636.3565
40630	5	5	15.9	1752	1	1	1	643.3628
40630	5	5	15.9	2169	1	1	1	466.8966

40630	5	5	15.9	2236	1	1	1	708.3853
55711	2	2	7.2	2615	1	1	1	780.9093
55711	2	2	7.2	4425	1	1	1	1188.5612
44960	5	5	16.8	1231	1	0	0	579.829
44960	5	5	16.8	1246	1	1	1	581.7968
44960	5	5	16.8	1701	1	1	0	638.849
44960	5	5	16.8	2168	1	1	0	699.828
44960	5	5	16.8	3984	1	1	0	741.7413
67813	5	5	9.3	823	1	1	1	525.267
67813	5	5	9.3	1302	1	1	1	587.8209
67813	5	5	9.3	1499	1	1	1	613.8586
67813	5	5	9.3	2045	1	1	0	684.8177
67813	5	5	9.3	2985	1	1	1	877.4851
34169	5	4	32.2	2227	1	1	0	706.4239
34169	5	4	32.2	5132	1	1	1	997.5598
34169	5	4	32.2	5284	1	1	0	1058.8863
34169	5	4	32.2	5723	1	1	0	1184.3097
25092	3	3	19.8	2616	1	1	1	781.3654
25092	3	3	19.8	2784	1	1	1	829.3752
25092	3	3	19.8	2887	1	1	1	852.9487
40331	3	3	11.7	1352	1	1	1	595.3246
40331	3	3	11.7	1976	1	1	1	676.8248
40331	3	3	11.7	3134	1	1	1	912.479
286993	5	5	3.3	2022	1	1	1	681.8763
286993	5	5	3.3	3000	1	1	1	881.4818
286993	5	5	3.3	3602	1	1	1	1025.0561
286993	5	5	3.3	3914	1	1	1	1097.6201
286993	5	5	3.3	4036	1	1	1	747.3963
80376	5	5	8.3	20	1	1	1	357.729
80376	5	5	8.3	1607	1	1	0	628.8514
80376	5	5	8.3	1767	1	1	0	646.3248
80376	5	5	8.3	2542	1	1	1	764.4033
80376	5	5	8.3	2654	1	1	1	528.6017
83408	4	4	5.8	623	1	1	1	495.7846
83408	4	4	5.8	791	1	1	1	521.7508
83408	4	4	5.8	1606	1	1	1	628.8296
83408	4	4	5.8	2806	1	1	1	833.4284
44816	7	7	17.7	135	1	1	1	398.2215
44816	7	7	17.7	451	1	1	1	469.2825
44816	7	7	17.7	598	1	1	1	492.2739
44816	7	7	17.7	765	1	1	1	517.2859
44816	7	7	17.7	1591	1	1	1	626.7977
44816	7	7	17.7	1873	1	1	1	660.8383
44816	7	7	17.7	2678	1	1	1	532.9035
45894	6	4	14	176	1	1	1	408.7504
45894	6	4	14	1231	1	0	0	579.829
45894	6	4	14	3541	1	1	1	671.9987
45894	6	4	14	3561	1	1	1	1015.0344
44974	3	2	8.1	2091	1	1	1	690.3616
44974	3	2	8.1	3835	1	1	1	1079.5026
38472	2	2	8.5	2268	1	1	1	713.8838
38472	2	2	8.5	2996	1	1	1	880.9543
31696	3	3	10.8	486	1	1	1	475.2763
31696	3	3	10.8	1468	1	1	1	610.3319
31696	3	3	10.8	2214	1	1	1	704.8826
90209	2	2	3.2	1383	1	1	1	599.2982
90209	2	2	3.2	2560	1	1	1	768.368
65220	4	3	10.3	2939	1	1	1	865.9222

65220	4	3	10.3	3847	1	1	1	722.7046
65220	4	3	10.3	4637	1	1	1	881.4528
44788	4	4	11.6	369	1	1	1	453.2289
44788	4	4	11.6	770	1	1	1	517.7683
44788	4	4	11.6	2323	1	1	1	482.2241
44788	4	4	11.6	3384	1	1	1	965.4058
30060	3	3	16.4	1442	1	1	1	607.2845
30060	3	3	16.4	2714	1	1	1	810.4493
30060	3	3	16.4	3622	1	1	1	1029.4876
17591	4	3	24.3	785	1	1	1	520.2959
17591	4	3	24.3	855	1	1	1	530.7824
17591	4	3	24.3	3703	1	1	1	1051.0326
40463	3	2	5.9	630	1	1	1	497.2806
40463	3	2	5.9	2645	1	1	1	789.3995
194402	4	4	3.8	710	1	1	1	508.2739
194402	4	4	3.8	2297	1	1	1	718.3744
194402	4	4	3.8	3586	1	1	1	1020.5619
194402	4	4	3.8	4649	1	1	1	887.8182
264216	2	2	1.2	1650	1	1	1	633.2961
264216	2	2	1.2	3416	1	1	1	973.0233
20970	2	2	13.4	1318	1	1	1	591.3533
20970	2	2	13.4	2597	1	1	1	775.8847
47389	4	4	14.6	1231	1	0	0	579.829
47389	4	4	14.6	2426	1	1	1	493.9595
47389	4	4	14.6	3398	1	1	1	969.9751
47389	4	4	14.6	3673	1	1	1	696.673
20667	3	3	16	979	1	1	1	545.3187
20667	3	3	16	1023	1	1	1	552.3099
20667	3	3	16	3717	1	1	1	703.0358
19009	2	2	14.6	1610	1	1	1	629.3735
19009	2	2	14.6	2120	1	1	1	692.8591
67582	2	2	4	1624	1	1	1	630.8453
67582	2	2	4	1903	1	1	1	665.8608
20989	3	3	19.2	590	1	1	1	491.2627
20989	3	3	19.2	2004	1	1	1	679.8668
20989	3	3	19.2	2557	1	1	1	766.9186
38757	2	2	9.5	2336	1	1	1	483.9074
38757	2	2	9.5	3754	1	1	1	1064.5179
82214	2	2	3.9	1940	1	1	1	671.3855
82214	2	2	3.9	2736	1	1	1	816.4415
52003	3	2	8.2	2814	1	1	1	558.9713
52003	3	2	8.2	4528	1	1	1	827.7833
61584	3	2	6.4	1661	1	1	1	634.2915
61584	3	2	6.4	4541	1	1	1	1250.691
73869	3	3	5.4	1217	1	1	1	578.3272
73869	3	3	5.4	1603	1	0	0	628.813
73869	3	3	5.4	2460	1	1	1	747.8647
55890	3	3	8.6	529	1	1	1	481.7687
55890	3	3	8.6	2253	1	1	1	475.2101
55890	3	3	8.6	4453	1	1	1	797.7518
119913	2	2	2.2	375	1	1	0	453.756
119913	2	2	2.2	2893	1	1	1	852.998
43168	3	3	8.1	82	1	1	1	383.2162
43168	3	3	8.1	1422	1	1	1	603.807
43168	3	3	8.1	2357	1	1	1	727.3806
14066	2	2	18.5	1237	1	1	1	580.8171
14066	2	2	18.5	1262	1	1	0	583.8056
69890	2	2	3.9	982	1	1	1	545.7903

69890	2	2	3.9	2905	1	1	1	569.599
48286	2	2	4.9	62	1	1	1	374.7213
48286	2	2	4.9	2953	1	1	1	869.4269
125058	2	2	3.7	1263	1	1	1	583.8065
125058	2	2	3.7	5304	1	1	1	1066.5441
29898	4	4	12.6	474	1	1	1	472.7743
29898	4	4	12.6	1492	1	1	1	613.3701
29898	4	4	12.6	2037	1	1	1	683.8425
29898	4	4	12.6	2394	1	1	1	490.2936
65977	3	3	6.1	1315	1	1	1	591.2935
65977	3	3	6.1	1763	1	1	1	644.861
65977	3	3	6.1	2567	1	1	1	768.9842
114571	2	2	2.1	416	1	1	1	462.2565
114571	2	2	2.1	2527	1	1	1	760.399
29708	3	3	15.7	517	1	1	1	479.2842
29708	3	3	15.7	1176	1	1	1	573.3023
29708	3	3	15.7	5030	1	1	1	1442.2215
71322	2	2	3.9	672	1	1	1	502.798
71322	2	2	3.9	3273	1	1	1	941.4741
66087	2	2	3.6	2845	1	1	1	842.4798
66087	2	2	3.6	4345	1	1	1	775.1002
94470	3	3	4.7	1307	1	1	1	589.314
94470	3	3	4.7	2433	1	1	1	742.958
94470	3	3	4.7	3219	1	1	1	620.3168
47357	4	4	11.4	459	1	1	1	470.7769
47357	4	4	11.4	1665	1	1	1	634.8093
47357	4	4	11.4	1841	1	1	1	655.3789
47357	4	4	11.4	3423	1	1	1	975.4364
153955	2	2	2.1	1786	1	1	1	648.4241
153955	2	2	2.1	3410	1	1	1	648.3702
32983	2	2	7.5	850	1	1	1	529.322
32983	2	2	7.5	1600	1	1	1	627.8508
58865	2	2	5.6	1548	1	1	1	620.8193
58865	2	2	5.6	3414	1	1	1	972.997
47005	3	3	9.1	491	1	1	1	475.7685
47005	3	3	9.1	2591	1	1	1	774.9477
47005	3	3	9.1	3432	1	1	1	654.6627
29729	2	2	12.8	890	1	1	1	534.755
29729	2	2	12.8	5003	1	1	1	951.505
47042	2	2	9.5	876	1	1	0	533.3026
47042	2	2	9.5	5148	1	1	1	1007.7667
123905	2	2	1.9	795	1	1	1	522.2847
123905	2	2	1.9	1939	1	1	1	671.3379
59235	2	2	4.7	2050	1	1	1	685.3189
59235	2	2	4.7	2706	1	1	1	807.9604
47036	2	2	5	876	1	0	0	533.3026
47036	2	2	5	1886	1	1	1	662.8522
59477	3	3	11	1379	1	1	1	598.3266
59477	3	3	11	3213	1	1	1	928.5349
59477	3	3	11	5314	1	1	1	1068.2237
14095	2	2	18.5	1179	1	1	1	573.8088
14095	2	2	18.5	1262	1	0	0	583.8056
16304	2	2	16	835	1	1	1	527.7864
16304	2	2	16	2322	1	1	1	722.3694
28210	2	2	5	71	1	1	1	378.2604
28210	2	2	5	104	1	1	1	387.7655
116744	2	2	3.3	2115	1	1	1	692.3507
116744	2	2	3.3	3969	1	1	1	740.1071

86361	2	2	2.9	187	1	1	1	412.2268
86361	2	2	2.9	2778	1	1	1	826.0054
86221	2	2	2.8	762	1	1	1	516.8135
86221	2	2	2.8	2558	1	1	1	767.9348
109254	2	2	2.4	905	1	1	1	536.8291
109254	2	2	2.4	2702	1	1	1	538.626
130510	2	2	2.2	706	1	1	1	507.2906
130510	2	2	2.2	2892	1	1	1	852.9644
55656	2	2	4.4	1774	1	1	0	647.338
55656	2	2	4.4	2045	1	0	1	684.8177
33803	2	2	6.6	1129	1	1	1	567.2611
33803	2	2	6.6	1550	1	1	1	620.8485
22104	2	2	8.9	490	1	1	1	475.7684
22104	2	2	8.9	865	1	1	1	532.3186
45968	2	2	5.3	923	1	1	1	538.2905
45968	2	2	5.3	1980	1	1	1	677.4211
prot_mass	prot_matches	prot_sequenc	prot_cover	pep_query	pep_rank	pep_isbold	pep_isunique	pep_exp_mz
50361	39	22	69.8	699	1	1	1	488.7414
50361	39	22	69.8	896	1	1	0	514.2821
50361	39	22	69.8	1088	1	1	0	359.8492
50361	39	22	69.8	1327	1	1	0	570.3503
50361	39	22	69.8	1355	1	1	0	573.7986
50361	39	22	69.8	1645	1	1	0	608.2958
50361	39	22	69.8	1846	1	1	0	634.397
50361	39	22	69.8	1979	1	1	1	650.3146
50361	39	22	69.8	2342	1	1	0	701.8322
50361	39	22	69.8	2977	1	1	0	819.927
50361	39	22	69.8	3080	1	1	0	840.4014
50361	39	22	69.8	3137	1	1	0	848.9192
50361	39	22	69.8	3499	1	1	0	911.9646
50361	39	22	69.8	3906	1	1	0	976.4474
50361	39	22	69.8	3962	1	1	0	987.001
50361	39	22	69.8	4249	1	1	0	701.0352
50361	39	22	69.8	4295	1	1	0	709.3574
50361	39	22	69.8	4965	1	1	0	1184.0666
50361	39	22	69.8	5472	1	1	0	922.112
50361	39	22	69.8	5553	1	1	0	933.4537
50361	39	22	69.8	5978	1	1	1	800.8735
50361	39	22	69.8	6100	1	1	1	1109.5067
51385	36	23	63.8	1016	1	1	0	530.2681
51385	36	23	63.8	1088	1	0	0	359.8492
51385	36	23	63.8	1327	1	0	0	570.3503
51385	36	23	63.8	1355	1	0	0	573.7986
51385	36	23	63.8	1645	1	0	0	608.2958
51385	36	23	63.8	1846	1	0	0	634.397
51385	36	23	63.8	1934	1	1	0	644.3329
51385	36	23	63.8	1967	1	1	1	648.8071
51385	36	23	63.8	2401	1	1	0	708.8486
51385	36	23	63.8	2489	1	1	1	723.3266
51385	36	23	63.8	2532	1	1	0	731.8453
51385	36	23	63.8	2977	1	0	0	819.927
51385	36	23	63.8	3080	1	0	0	840.4014
51385	36	23	63.8	3137	1	0	0	848.9192
51385	36	23	63.8	3368	1	1	1	889.9346
51385	36	23	63.8	3499	1	0	0	911.9646
51385	36	23	63.8	3652	1	1	1	936.9383
51385	36	23	63.8	3906	1	0	0	976.4474
51385	36	23	63.8	3962	1	0	0	987.001

51385	36	23	63.8	4249	1	0	0	701.0352
51385	36	23	63.8	4295	1	0	0	709.3574
51385	36	23	63.8	5477	1	1	0	923.1111
51385	36	23	63.8	5564	1	1	1	934.7713
51341	36	21	59.7	1016	1	0	0	530.2681
51341	36	21	59.7	1088	1	0	0	359.8492
51341	36	21	59.7	1327	1	0	0	570.3503
51341	36	21	59.7	1355	1	0	0	573.7986
51341	36	21	59.7	1645	1	0	0	608.2958
51341	36	21	59.7	1846	1	0	0	634.397
51341	36	21	59.7	1934	1	0	0	644.3329
51341	36	21	59.7	2011	1	1	1	654.8244
51341	36	21	59.7	2342	1	0	0	701.8322
51341	36	21	59.7	2442	1	1	0	716.3194
51341	36	21	59.7	2532	1	0	0	731.8453
51341	36	21	59.7	2977	1	0	0	819.927
51341	36	21	59.7	3039	1	1	0	833.3937
51341	36	21	59.7	3137	1	0	0	848.9192
51341	36	21	59.7	3395	1	1	1	895.4434
51341	36	21	59.7	3761	1	1	1	950.4709
51341	36	21	59.7	3906	1	0	0	976.4474
51341	36	21	59.7	3962	1	0	0	987.001
51341	36	21	59.7	4249	1	0	0	701.0352
51341	36	21	59.7	5409	1	1	1	913.1083
51341	36	21	59.7	5591	1	1	0	938.783
50310	35	19	54.5	896	1	0	0	514.2821
50310	35	19	54.5	1088	1	0	0	359.8492
50310	35	19	54.5	1327	1	0	0	570.3503
50310	35	19	54.5	1355	1	0	0	573.7986
50310	35	19	54.5	1645	1	0	0	608.2958
50310	35	19	54.5	1846	1	0	0	634.397
50310	35	19	54.5	1974	1	1	1	649.8221
50310	35	19	54.5	2342	1	0	0	701.8322
50310	35	19	54.5	2977	1	0	0	819.927
50310	35	19	54.5	3080	1	0	0	840.4014
50310	35	19	54.5	3137	1	0	0	848.9192
50310	35	19	54.5	3499	1	0	0	911.9646
50310	35	19	54.5	3906	1	0	0	976.4474
50310	35	19	54.5	3962	1	0	0	987.001
50310	35	19	54.5	4249	1	0	0	701.0352
50310	35	19	54.5	4295	1	0	0	709.3574
50310	35	19	54.5	4965	1	0	0	1184.0666
50310	35	19	54.5	5472	1	0	0	922.112
50310	35	19	54.5	5553	1	0	0	933.4537
50194	33	20	74	433	1	1	0	452.2174
50194	33	20	74	736	1	1	0	494.2774
50194	33	20	74	1296	1	1	0	566.7869
50194	33	20	74	1916	1	1	0	642.2554
50194	33	20	74	2316	1	1	0	698.8502
50194	33	20	74	2322	1	1	0	466.2575
50194	33	20	74	2555	1	1	0	737.4354
50194	33	20	74	2873	1	1	0	799.8868
50194	33	20	74	3032	1	1	1	832.4405
50194	33	20	74	3153	1	1	0	851.4556
50194	33	20	74	3401	1	1	0	896.9676
50194	33	20	74	3598	1	1	0	929.4446
50194	33	20	74	3707	1	1	0	943.4762
50194	33	20	74	3971	1	1	0	989.4444

50194	33	20	74	4934	1	1	0	1173.5076
50194	33	20	74	5000	1	1	0	795.7362
50194	33	20	74	5023	1	1	0	1198.0992
50194	33	20	74	5275	1	1	0	1321.1161
50194	33	20	74	6089	1	1	0	1107.9135
50194	33	20	74	6704	1	1	0	1345.4228
50191	33	20	74	433	1	0	0	452.2174
50191	33	20	74	736	1	0	0	494.2774
50191	33	20	74	1296	1	0	0	566.7869
50191	33	20	74	1916	1	0	0	642.2554
50191	33	20	74	2316	1	0	0	698.8502
50191	33	20	74	2322	1	0	0	466.2575
50191	33	20	74	2555	1	0	0	737.4354
50191	33	20	74	2873	1	0	0	799.8868
50191	33	20	74	3116	1	1	1	845.9453
50191	33	20	74	3153	1	0	0	851.4556
50191	33	20	74	3401	1	0	0	896.9676
50191	33	20	74	3598	1	0	0	929.4446
50191	33	20	74	3707	1	0	0	943.4762
50191	33	20	74	3971	1	0	0	989.4444
50191	33	20	74	4934	1	0	0	1173.5076
50191	33	20	74	5000	1	0	0	795.7362
50191	33	20	74	5023	1	0	0	1198.0992
50191	33	20	74	5275	1	0	0	1321.1161
50191	33	20	74	6089	1	0	0	1107.9135
50191	33	20	74	6704	1	0	0	1345.4228
47075	28	21	62.1	585	1	1	1	475.7504
47075	28	21	62.1	620	1	1	0	479.7637
47075	28	21	62.1	804	1	1	0	502.7745
47075	28	21	62.1	861	1	1	0	509.2767
47075	28	21	62.1	979	1	1	0	524.2611
47075	28	21	62.1	1231	1	1	0	557.8451
47075	28	21	62.1	1342	1	1	0	571.7901
47075	28	21	62.1	1479	1	1	0	587.3273
47075	28	21	62.1	2340	1	1	0	701.3696
47075	28	21	62.1	2493	1	1	1	724.3793
47075	28	21	62.1	2649	1	1	0	751.942
47075	28	21	62.1	2824	1	1	1	788.4272
47075	28	21	62.1	2853	1	1	0	794.3544
47075	28	21	62.1	2858	1	1	0	795.4325
47075	28	21	62.1	3513	1	1	0	914.4722
47075	28	21	62.1	3814	1	1	0	961.0053
47075	28	21	62.1	3817	1	1	0	961.5237
47075	28	21	62.1	4195	1	1	0	1038.0453
47075	28	21	62.1	5347	1	1	0	1350.2014
47075	28	21	62.1	5675	1	1	0	1448.2541
47075	28	21	62.1	5909	1	1	1	1569.3109
46960	29	22	69.2	535	1	1	0	468.263
46960	29	22	69.2	620	1	0	0	479.7637
46960	29	22	69.2	700	1	1	1	488.7812
46960	29	22	69.2	804	1	0	0	502.7745
46960	29	22	69.2	861	1	0	0	509.2767
46960	29	22	69.2	979	1	0	0	524.2611
46960	29	22	69.2	1195	1	1	1	552.8296
46960	29	22	69.2	1231	1	0	0	557.8451
46960	29	22	69.2	1342	1	0	0	571.7901
46960	29	22	69.2	1479	1	0	0	587.3273
46960	29	22	69.2	2340	1	0	0	701.3696

46960	29	22	69.2	2649	1	0	0	751.942
46960	29	22	69.2	2853	1	0	0	794.3544
46960	29	22	69.2	2858	1	0	0	795.4325
46960	29	22	69.2	3513	1	0	0	914.4722
46960	29	22	69.2	3814	1	0	0	961.0053
46960	29	22	69.2	3817	1	0	0	961.5237
46960	29	22	69.2	4195	1	0	0	1038.0453
46960	29	22	69.2	5347	1	0	0	1350.2014
46960	29	22	69.2	5675	1	0	0	1448.2541
46960	29	22	69.2	6398	1	1	1	1307.2602
71712	29	22	51.5	1696	1	1	0	614.817
71712	29	22	51.5	1709	1	1	0	616.3265
71712	29	22	51.5	1960	1	1	0	647.8159
71712	29	22	51.5	2003	1	1	1	653.866
71712	29	22	51.5	2082	1	1	0	665.3104
71712	29	22	51.5	2232	1	1	0	687.8081
71712	29	22	51.5	2279	1	1	0	693.8682
71712	29	22	51.5	2434	1	1	1	713.8857
71712	29	22	51.5	2460	1	1	0	718.88
71712	29	22	51.5	2551	1	1	0	737.3461
71712	29	22	51.5	2790	1	1	0	519.589
71712	29	22	51.5	2806	1	1	1	783.9076
71712	29	22	51.5	3030	1	1	0	832.4088
71712	29	22	51.5	3058	1	1	0	838.3683
71712	29	22	51.5	3086	1	1	0	840.9196
71712	29	22	51.5	5194	1	1	1	1280.6297
71712	29	22	51.5	5236	1	1	0	863.4107
71712	29	22	51.5	5287	1	1	0	1329.6356
71712	29	22	51.5	5791	1	1	1	1007.4721
71712	29	22	51.5	5794	1	1	1	1009.1668
71712	29	22	51.5	5808	1	1	1	1526.3534
71712	29	22	51.5	5920	1	1	1	1050.17
615675	25	22	6.1	243	1	1	1	417.2601
615675	25	22	6.1	473	1	1	1	458.2972
615675	25	22	6.1	927	1	1	1	518.2953
615675	25	22	6.1	1778	1	1	1	625.3521
615675	25	22	6.1	1833	1	1	1	632.8313
615675	25	22	6.1	2410	1	1	1	710.8933
615675	25	22	6.1	2470	1	1	1	720.8813
615675	25	22	6.1	2568	1	1	1	739.8438
615675	25	22	6.1	2754	1	1	1	771.4265
615675	25	22	6.1	2932	1	1	1	810.4535
615675	25	22	6.1	3179	1	1	1	854.4695
615675	25	22	6.1	3230	1	1	1	864.9768
615675	25	22	6.1	3710	1	1	1	943.5537
615675	25	22	6.1	3841	1	1	1	643.9946
615675	25	22	6.1	4079	1	1	1	1015.5493
615675	25	22	6.1	4144	1	1	1	1027.5086
615675	25	22	6.1	4174	1	1	1	689.7021
615675	25	22	6.1	4229	1	1	1	1047.0504
615675	25	22	6.1	4263	1	1	1	1055.5943
615675	25	22	6.1	4411	1	1	1	1083.0258
615675	25	22	6.1	4488	1	1	1	1100.0373
615675	25	22	6.1	5175	1	1	1	844.4467
71559	24	20	47	1686	1	1	1	613.7985
71559	24	20	47	1696	1	0	0	614.817
71559	24	20	47	1709	1	0	0	616.3265
71559	24	20	47	1960	1	0	0	647.8159

71559	24	20	47	1965	1	1	1	648.3353
71559	24	20	47	2015	1	1	1	654.8559
71559	24	20	47	2232	1	0	0	687.8081
71559	24	20	47	2279	1	0	0	693.8682
71559	24	20	47	2368	1	1	1	704.3851
71559	24	20	47	2443	1	1	1	716.376
71559	24	20	47	2551	1	0	0	737.3461
71559	24	20	47	2696	1	1	1	761.9042
71559	24	20	47	3058	1	0	0	838.3683
71559	24	20	47	3065	1	1	1	839.4159
71559	24	20	47	3086	1	0	0	840.9196
71559	24	20	47	5214	1	0	0	1287.6359
71559	24	20	47	5236	1	0	0	863.4107
71559	24	20	47	5287	1	0	0	1329.6356
71559	24	20	47	5830	1	1	1	1022.5838
71559	24	20	47	5969	1	1	1	1065.8271
51237	24	16	44.8	803	1	1	1	502.7454
51237	24	16	44.8	1016	1	0	0	530.2681
51237	24	16	44.8	1088	1	0	0	359.8492
51237	24	16	44.8	1327	1	0	0	570.3503
51237	24	16	44.8	1355	1	0	0	573.7986
51237	24	16	44.8	1645	1	0	0	608.2958
51237	24	16	44.8	1846	1	0	0	634.397
51237	24	16	44.8	1952	1	1	1	647.3254
51237	24	16	44.8	2401	1	0	0	708.8486
51237	24	16	44.8	2442	1	0	0	716.3194
51237	24	16	44.8	2532	1	0	0	731.8453
51237	24	16	44.8	2977	1	0	0	819.927
51237	24	16	44.8	3137	1	0	0	848.9192
51237	24	16	44.8	3916	1	1	0	979.003
51237	24	16	44.8	5477	1	0	0	923.1111
51237	24	16	44.8	5608	1	1	1	943.4556
286993	19	16	10	611	1	1	1	478.7582
286993	19	16	10	1056	1	1	1	536.2754
286993	19	16	10	1611	1	1	1	603.4153
286993	19	16	10	2186	1	1	1	680.8828
286993	19	16	10	2193	1	1	1	681.8761
286993	19	16	10	2491	1	1	1	723.4035
286993	19	16	10	2497	1	1	1	724.903
286993	19	16	10	2868	1	1	1	798.4789
286993	19	16	10	3313	1	1	1	881.4819
286993	19	16	10	4131	1	1	1	1025.0565
286993	19	16	10	4242	1	1	1	1049.598
286993	19	16	10	4479	1	1	1	1097.62
286993	19	16	10	4658	1	1	1	746.7787
286993	19	16	10	4666	1	1	1	1120.5908
286993	19	16	10	5190	1	1	1	1278.681
286993	19	16	10	5916	1	1	1	785.9418
50755	22	13	35.6	1016	1	0	0	530.2681
50755	22	13	35.6	1088	1	0	0	359.8492
50755	22	13	35.6	1327	1	0	0	570.3503
50755	22	13	35.6	1355	1	0	0	573.7986
50755	22	13	35.6	1645	1	0	0	608.2958
50755	22	13	35.6	1846	1	0	0	634.397
50755	22	13	35.6	2252	1	1	1	690.824
50755	22	13	35.6	2401	1	0	0	708.8486
50755	22	13	35.6	2977	1	0	0	819.927
50755	22	13	35.6	3080	1	0	0	840.4014

50755	22	13	35.6	3137	1	0	0	848.9192
50755	22	13	35.6	3916	1	0	0	979.003
50755	22	13	35.6	5591	1	0	0	938.783
136636	23	19	23.5	283	1	1	1	424.7529
136636	23	19	23.5	592	1	1	1	476.2483
136636	23	19	23.5	681	1	1	1	486.7717
136636	23	19	23.5	743	1	1	1	494.7841
136636	23	19	23.5	1102	1	1	1	541.3526
136636	23	19	23.5	1384	1	1	1	576.8039
136636	23	19	23.5	1853	1	1	1	635.3615
136636	23	19	23.5	1949	1	1	1	646.826
136636	23	19	23.5	2542	1	1	1	734.8949
136636	23	19	23.5	2580	1	1	1	742.3844
136636	23	19	23.5	2814	1	1	1	784.9199
136636	23	19	23.5	3832	1	1	1	643.0317
136636	23	19	23.5	3893	1	1	1	650.0237
136636	23	19	23.5	3942	1	1	1	983.014
136636	23	19	23.5	4043	1	1	1	1004.0656
136636	23	19	23.5	4671	1	1	1	1121.12
136636	23	19	23.5	5141	1	1	1	834.452
136636	23	19	23.5	5290	1	1	1	887.4531
136636	23	19	23.5	6125	1	1	1	1113.2388
51883	20	14	45.6	1162	1	1	1	548.326
51883	20	14	45.6	1286	1	1	1	565.3266
51883	20	14	45.6	1404	1	1	1	579.3291
51883	20	14	45.6	2289	1	1	1	695.3397
51883	20	14	45.6	3091	1	1	1	841.4777
51883	20	14	45.6	3337	1	1	0	590.2668
51883	20	14	45.6	3464	1	1	1	905.9432
51883	20	14	45.6	3516	1	1	1	610.3261
51883	20	14	45.6	3778	1	1	1	954.029
51883	20	14	45.6	4173	1	1	1	1034.0075
51883	20	14	45.6	4272	1	1	1	1057.5285
51883	20	14	45.6	4320	1	1	1	1068.0727
51883	20	14	45.6	5220	1	1	1	860.4335
51883	20	14	45.6	6254	1	1	1	1145.9186
50250	24	16	49.8	433	1	0	0	452.2174
50250	24	16	49.8	736	1	0	0	494.2774
50250	24	16	49.8	1296	1	0	0	566.7869
50250	24	16	49.8	1916	1	0	0	642.2554
50250	24	16	49.8	2316	1	0	0	698.8502
50250	24	16	49.8	2322	1	0	0	466.2575
50250	24	16	49.8	2721	1	1	1	766.4559
50250	24	16	49.8	2845	1	1	1	792.8793
50250	24	16	49.8	3122	1	1	1	846.4381
50250	24	16	49.8	3153	1	0	0	851.4556
50250	24	16	49.8	3283	1	1	1	875.9642
50250	24	16	49.8	3707	1	0	0	943.4762
50250	24	16	49.8	4934	1	0	0	1173.5076
50250	24	16	49.8	5000	1	0	0	795.7362
50250	24	16	49.8	5050	1	0	0	1205.1053
50250	24	16	49.8	6089	1	0	0	1107.9135
98766	22	17	28.7	937	1	1	0	519.2693
98766	22	17	28.7	1395	1	1	1	578.7742
98766	22	17	28.7	1800	1	1	0	628.3411
98766	22	17	28.7	1843	1	1	1	634.3083
98766	22	17	28.7	1881	1	1	0	639.3143
98766	22	17	28.7	2110	1	1	1	668.8406

98766	22	17	28.7	2352	1	1	0	702.4366
98766	22	17	28.7	2659	1	1	1	503.5729
98766	22	17	28.7	2805	1	1	0	783.4575
98766	22	17	28.7	3193	1	1	1	858.5209
98766	22	17	28.7	3965	1	1	1	659.019
98766	22	17	28.7	4147	1	1	1	1027.5264
98766	22	17	28.7	4269	1	1	1	1056.5295
98766	22	17	28.7	5093	1	1	1	815.4729
98766	22	17	28.7	5109	1	1	1	822.7196
98766	22	17	28.7	5313	1	1	1	893.0995
98766	22	17	28.7	5775	1	1	1	998.1536
37004	17	12	55.3	240	1	1	1	417.2355
37004	17	12	55.3	943	1	1	1	519.7767
37004	17	12	55.3	1333	1	1	1	570.8258
37004	17	12	55.3	2451	1	1	1	717.8827
37004	17	12	55.3	2613	1	1	1	749.927
37004	17	12	55.3	3063	1	1	1	559.6626
37004	17	12	55.3	3280	1	1	1	875.9087
37004	17	12	55.3	3308	1	1	1	881.3974
37004	17	12	55.3	4083	1	1	1	1017.0393
37004	17	12	55.3	4424	1	1	1	1086.5073
37004	17	12	55.3	4702	1	1	1	1125.54
37004	17	12	55.3	5705	1	1	1	975.1256
49813	22	13	38.1	110	1	1	1	390.7101
49813	22	13	38.1	468	1	1	1	457.7869
49813	22	13	38.1	887	1	1	1	513.3081
49813	22	13	38.1	888	1	1	1	513.748
49813	22	13	38.1	1250	1	1	1	560.8029
49813	22	13	38.1	1976	1	1	1	649.8771
49813	22	13	38.1	2506	1	1	1	726.3399
49813	22	13	38.1	2969	1	1	1	545.6382
49813	22	13	38.1	2974	1	1	1	819.4408
49813	22	13	38.1	3317	1	1	1	882.4651
49813	22	13	38.1	4887	1	1	1	771.3808
49813	22	13	38.1	5148	1	1	1	836.114
49813	22	13	38.1	5756	1	1	1	1487.211
47083	18	14	41.5	535	1	0	0	468.263
47083	18	14	41.5	620	1	0	0	479.7637
47083	18	14	41.5	804	1	0	0	502.7745
47083	18	14	41.5	861	1	0	0	509.2767
47083	18	14	41.5	979	1	0	0	524.2611
47083	18	14	41.5	1231	1	0	0	557.8451
47083	18	14	41.5	1479	1	0	0	587.3273
47083	18	14	41.5	2340	1	0	0	701.3696
47083	18	14	41.5	2853	1	0	0	794.3544
47083	18	14	41.5	3513	1	0	0	914.4722
47083	18	14	41.5	3814	1	0	0	961.0053
47083	18	14	41.5	4165	1	1	1	1031.0369
47083	18	14	41.5	5347	1	0	0	1350.2014
47083	18	14	41.5	5675	1	0	0	1448.2541
47735	19	16	59.9	93	1	1	1	383.7504
47735	19	16	59.9	327	1	1	0	433.764
47735	19	16	59.9	1288	1	1	0	565.797
47735	19	16	59.9	1410	1	1	0	579.8293
47735	19	16	59.9	1567	1	1	0	598.2831
47735	19	16	59.9	1964	1	1	0	648.3248
47735	19	16	59.9	1999	1	1	0	653.3361
47735	19	16	59.9	2988	1	1	0	822.9419

47735	19	16	59.9	3625	1	1	0	622.3452
47735	19	16	59.9	3711	1	1	0	943.9539
47735	19	16	59.9	3983	1	1	1	992.4695
47735	19	16	59.9	4082	1	1	1	678.3586
47735	19	16	59.9	4726	1	1	0	753.0273
47735	19	16	59.9	4768	1	1	0	757.01
47735	19	16	59.9	5387	1	1	1	907.5128
47735	19	16	59.9	6379	1	1	1	1277.9342
71456	17	14	31.7	1696	1	0	0	614.817
71456	17	14	31.7	1960	1	0	0	647.8159
71456	17	14	31.7	2082	1	0	0	665.3104
71456	17	14	31.7	2232	1	0	0	687.8081
71456	17	14	31.7	2460	1	0	0	718.88
71456	17	14	31.7	2551	1	0	0	737.3461
71456	17	14	31.7	2790	1	0	0	519.589
71456	17	14	31.7	3040	1	1	1	833.3978
71456	17	14	31.7	3058	1	0	0	838.3683
71456	17	14	31.7	3086	1	0	0	840.9196
71456	17	14	31.7	5214	1	0	0	1287.6359
71456	17	14	31.7	5236	1	0	0	863.4107
71741	15	12	27.1	1577	1	1	1	599.3507
71741	15	12	27.1	1696	1	0	0	614.817
71741	15	12	27.1	1900	1	1	1	639.8507
71741	15	12	27.1	1960	1	0	0	647.8159
71741	15	12	27.1	2232	1	0	0	687.8081
71741	15	12	27.1	2460	1	0	0	718.88
71741	15	12	27.1	2551	1	0	0	737.3461
71741	15	12	27.1	3030	1	0	0	832.4088
71741	15	12	27.1	3058	1	0	0	838.3683
71741	15	12	27.1	3086	1	0	0	840.9196
71741	15	12	27.1	5214	1	0	0	1287.6359
71741	15	12	27.1	5236	1	0	0	863.4107
41112	14	11	36.4	142	1	1	1	397.2265
41112	14	11	36.4	188	1	1	0	408.7505
41112	14	11	36.4	1486	1	1	0	587.8105
41112	14	11	36.4	1674	1	1	0	611.8189
41112	14	11	36.4	2571	1	1	0	740.3928
41112	14	11	36.4	2673	1	1	1	758.3623
41112	14	11	36.4	3092	1	1	1	841.9206
41112	14	11	36.4	3520	1	1	1	915.5025
41112	14	11	36.4	3570	1	1	1	923.9315
41112	14	11	36.4	4393	1	1	0	719.6976
41112	14	11	36.4	4547	1	1	1	1107.0611
41563	13	10	33.6	94	1	1	1	384.2007
41563	13	10	33.6	98	1	1	0	386.2273
41563	13	10	33.6	482	1	1	0	459.2948
41563	13	10	33.6	1619	1	1	1	604.3192
41563	13	10	33.6	1633	1	1	1	606.3072
41563	13	10	33.6	2185	1	1	0	680.8596
41563	13	10	33.6	2371	1	1	1	470.2451
41563	13	10	33.6	2492	1	1	0	723.8742
41563	13	10	33.6	4126	1	1	1	1024.5135
41563	13	10	33.6	5473	1	1	0	1382.6892
41834	14	13	40	98	1	0	0	386.2273
41834	14	13	40	482	1	0	0	459.2948
41834	14	13	40	591	1	1	1	476.2482
41834	14	13	40	908	1	1	1	516.7596
41834	14	13	40	1510	1	1	1	591.811

41834	14	13	40	1551	1	1	0	596.3212
41834	14	13	40	1694	1	1	1	614.8137
41834	14	13	40	2185	1	0	1	680.8596
41834	14	13	40	2344	1	1	1	701.8748
41834	14	13	40	2492	1	0	0	723.8742
41834	14	13	40	2543	1	1	1	735.3817
41834	14	13	40	4139	1	1	1	1025.9984
41834	14	13	40	5473	1	0	0	1382.6892
41937	16	13	54.9	158	1	1	0	400.7718
41937	16	13	54.9	698	1	1	0	488.7276
41937	16	13	54.9	1436	1	1	0	582.3023
41937	16	13	54.9	1555	1	1	0	596.7684
41937	16	13	54.9	2557	1	1	1	738.3413
41937	16	13	54.9	2675	1	1	0	758.3777
41937	16	13	54.9	3355	1	1	0	887.9518
41937	16	13	54.9	3596	1	1	1	928.469
41937	16	13	54.9	3895	1	1	0	974.94
41937	16	13	54.9	3910	1	1	0	977.5349
41937	16	13	54.9	4489	1	1	1	1100.0404
41937	16	13	54.9	5927	1	1	0	1051.2186
41937	16	13	54.9	5965	1	1	1	1065.5103
31671	15	10	44.8	307	1	1	0	429.2456
31671	15	10	44.8	348	1	1	0	436.2541
31671	15	10	44.8	720	1	1	1	492.2524
31671	15	10	44.8	1568	1	1	1	598.2951
31671	15	10	44.8	2028	1	1	1	657.3207
31671	15	10	44.8	2465	1	1	1	719.3836
31671	15	10	44.8	2537	1	1	1	733.3932
31671	15	10	44.8	2900	1	1	1	805.4065
31671	15	10	44.8	3803	1	1	1	959.4702
31671	15	10	44.8	5494	1	1	1	694.104
36185	16	13	49.5	562	1	1	1	472.2581
36185	16	13	49.5	1053	1	1	1	535.8266
36185	16	13	49.5	1772	1	1	1	416.9029
36185	16	13	49.5	1981	1	1	1	650.8484
36185	16	13	49.5	1996	1	1	1	652.3788
36185	16	13	49.5	2019	1	1	1	655.8137
36185	16	13	49.5	2253	1	1	1	690.8266
36185	16	13	49.5	2608	1	1	1	747.8806
36185	16	13	49.5	2672	1	1	1	505.2835
36185	16	13	49.5	2742	1	1	1	512.6553
36185	16	13	49.5	3342	1	1	1	885.4294
36185	16	13	49.5	4371	1	1	1	717.7313
36185	16	13	49.5	4905	1	1	1	1161.1086
105902	19	18	27.2	151	1	1	1	399.764
105902	19	18	27.2	682	1	1	1	486.7772
105902	19	18	27.2	901	1	1	0	515.2877
105902	19	18	27.2	941	1	1	0	519.7677
105902	19	18	27.2	993	1	1	1	525.7702
105902	19	18	27.2	1237	1	1	0	558.7982
105902	19	18	27.2	1409	1	1	0	579.8216
105902	19	18	27.2	1413	1	1	0	580.3004
105902	19	18	27.2	2265	1	1	0	691.8853
105902	19	18	27.2	2324	1	1	0	699.3723
105902	19	18	27.2	2565	1	1	0	738.8949
105902	19	18	27.2	2666	1	1	0	756.8654
105902	19	18	27.2	3733	1	1	1	946.5266
105902	19	18	27.2	3923	1	1	0	653.6862

105902	19	18	27.2	3986	1	1	0	993.482
105902	19	18	27.2	4087	1	1	0	678.3803
105902	19	18	27.2	5150	1	1	0	836.799
105902	19	18	27.2	6370	1	1	0	954.7689
47236	16	13	48	327	1	0	0	433.764
47236	16	13	48	1288	1	0	0	565.797
47236	16	13	48	1410	1	0	0	579.8293
47236	16	13	48	1567	1	0	0	598.2831
47236	16	13	48	1964	1	0	0	648.3248
47236	16	13	48	1999	1	0	0	653.3361
47236	16	13	48	2988	1	0	0	822.9419
47236	16	13	48	3625	1	0	0	622.3452
47236	16	13	48	3711	1	0	0	943.9539
47236	16	13	48	3983	1	0	1	992.4695
47236	16	13	48	4726	1	0	0	753.0273
47236	16	13	48	4768	1	0	0	757.01
47236	16	13	48	6380	1	1	1	1278.2726
65547	17	14	30.5	143	1	1	1	397.2324
65547	17	14	30.5	1289	1	1	1	566.239
65547	17	14	30.5	1412	1	1	0	580.2546
65547	17	14	30.5	1538	1	1	0	594.3551
65547	17	14	30.5	1642	1	1	1	607.7773
65547	17	14	30.5	1822	1	1	1	631.3422
65547	17	14	30.5	2616	1	1	1	750.8459
65547	17	14	30.5	2875	1	1	1	533.614
65547	17	14	30.5	3210	1	1	0	860.8774
65547	17	14	30.5	3479	1	1	1	908.4598
65547	17	14	30.5	3726	1	1	1	945.9748
65547	17	14	30.5	5054	1	1	0	1205.571
65547	17	14	30.5	5076	1	1	1	1214.6242
65547	17	14	30.5	5231	1	1	1	862.1197
196643	13	11	9.1	241	1	1	1	417.2477
196643	13	11	9.1	815	1	1	1	503.7807
196643	13	11	9.1	1055	1	1	1	536.2681
196643	13	11	9.1	1183	1	1	1	551.8034
196643	13	11	9.1	1911	1	1	1	640.9
196643	13	11	9.1	2042	1	1	1	659.3615
196643	13	11	9.1	2447	1	1	1	717.3645
196643	13	11	9.1	3166	1	1	1	853.4322
196643	13	11	9.1	4923	1	1	1	1169.083
196643	13	11	9.1	5059	1	1	1	1206.6101
196643	13	11	9.1	6211	1	1	1	1128.177
52347	7	6	22.8	1388	1	1	1	576.8605
52347	7	6	22.8	3141	1	1	1	849.3845
52347	7	6	22.8	3411	1	1	1	898.9054
52347	7	6	22.8	4367	1	1	1	1075.5689
52347	7	6	22.8	4936	1	1	1	1174.097
52347	7	6	22.8	5600	1	1	1	941.8219
103283	13	12	16.7	869	1	1	1	510.2588
103283	13	12	16.7	987	1	1	1	524.775
103283	13	12	16.7	1535	1	1	1	594.3223
103283	13	12	16.7	1886	1	1	1	639.3581
103283	13	12	16.7	1917	1	1	1	642.3151
103283	13	12	16.7	1995	1	1	1	652.3614
103283	13	12	16.7	2423	1	1	1	712.8855
103283	13	12	16.7	2484	1	1	1	722.3947
103283	13	12	16.7	2534	1	1	1	731.8877
103283	13	12	16.7	2989	1	1	1	822.9748

103283	13	12	16.7	3538	1	1	1	918.9739
103283	13	12	16.7	5130	1	1	1	829.0773
49487	11	9	31.2	685	1	1	1	486.79
49487	11	9	31.2	1543	1	1	1	595.824
49487	11	9	31.2	1730	1	1	1	619.3298
49487	11	9	31.2	1935	1	1	1	644.3573
49487	11	9	31.2	3285	1	1	1	876.4351
49487	11	9	31.2	4345	1	1	1	715.0172
49487	11	9	31.2	4352	1	1	1	1073.0568
49487	11	9	31.2	4471	1	1	1	1095.5724
49487	11	9	31.2	4512	1	1	1	1102.5718
103616	16	16	25.7	901	1	0	0	515.2877
103616	16	16	25.7	941	1	0	0	519.7677
103616	16	16	25.7	1237	1	0	0	558.7982
103616	16	16	25.7	1409	1	0	0	579.8216
103616	16	16	25.7	1413	1	0	0	580.3004
103616	16	16	25.7	2265	1	0	0	691.8853
103616	16	16	25.7	2324	1	0	0	699.3723
103616	16	16	25.7	2430	1	1	1	713.8337
103616	16	16	25.7	2565	1	0	0	738.8949
103616	16	16	25.7	2666	1	0	0	756.8654
103616	16	16	25.7	3923	1	0	0	653.6862
103616	16	16	25.7	3986	1	0	0	993.482
103616	16	16	25.7	4087	1	0	0	678.3803
103616	16	16	25.7	5150	1	0	0	836.799
103616	16	16	25.7	6370	1	0	0	954.7689
58863	14	14	36.3	530	1	1	1	467.2378
58863	14	14	36.3	1145	1	1	1	546.3029
58863	14	14	36.3	1424	1	1	1	581.2935
58863	14	14	36.3	1439	1	1	1	582.3243
58863	14	14	36.3	1580	1	1	1	600.3242
58863	14	14	36.3	2103	1	1	1	667.3625
58863	14	14	36.3	2901	1	1	1	805.4427
58863	14	14	36.3	3157	1	1	1	851.8718
58863	14	14	36.3	3212	1	1	1	860.94
58863	14	14	36.3	3960	1	1	1	657.6892
58863	14	14	36.3	4485	1	1	1	1099.0574
58863	14	14	36.3	5286	1	1	1	886.4545
58863	14	14	36.3	5514	1	1	1	928.4688
58863	14	14	36.3	5696	1	1	1	971.1671
73315	12	11	21.6	727	1	1	0	492.7896
73315	12	11	21.6	1771	1	1	1	624.8451
73315	12	11	21.6	1773	1	1	1	625.2859
73315	12	11	21.6	1802	1	1	0	628.8126
73315	12	11	21.6	1957	1	1	1	647.7949
73315	12	11	21.6	2190	1	1	1	681.3748
73315	12	11	21.6	2448	1	1	0	717.3686
73315	12	11	21.6	2567	1	1	1	739.385
73315	12	11	21.6	3470	1	1	1	907.4807
73315	12	11	21.6	3498	1	1	1	911.9534
73315	12	11	21.6	3555	1	1	1	920.4495
94743	13	12	19.8	56	1	1	1	372.6948
94743	13	12	19.8	90	1	1	1	383.2107
94743	13	12	19.8	442	1	1	1	453.7377
94743	13	12	19.8	952	1	1	1	520.3343
94743	13	12	19.8	1779	1	1	1	625.3661
94743	13	12	19.8	2509	1	1	1	726.8488
94743	13	12	19.8	2689	1	1	1	759.8401

94743	13	12	19.8	2809	1	1	1	784.3691
94743	13	12	19.8	3427	1	1	1	900.4672
94743	13	12	19.8	4727	1	1	1	1129.105
94743	13	12	19.8	5029	1	1	1	800.3915
94743	13	12	19.8	5717	1	1	1	978.1487
59235	8	7	21.2	1789	1	1	1	626.8845
59235	8	7	21.2	2213	1	1	0	685.3193
59235	8	7	21.2	2774	1	1	1	775.4323
59235	8	7	21.2	2916	1	1	0	807.9607
59235	8	7	21.2	3045	1	1	0	835.4178
59235	8	7	21.2	4044	1	1	1	669.7419
59235	8	7	21.2	5831	1	1	1	1023.1322
40001	13	10	33.2	1474	1	1	1	586.8018
40001	13	10	33.2	1561	1	1	0	597.335
40001	13	10	33.2	1653	1	1	0	609.306
40001	13	10	33.2	1907	1	1	1	640.8188
40001	13	10	33.2	2418	1	1	0	712.3355
40001	13	10	33.2	2581	1	1	1	742.4144
40001	13	10	33.2	2851	1	1	1	793.9411
40001	13	10	33.2	3434	1	1	1	902.4697
40001	13	10	33.2	4085	1	1	1	678.3626
40001	13	10	33.2	4796	1	1	1	1138.5913
29784	8	7	53.3	1749	1	1	1	621.8284
29784	8	7	53.3	3857	1	1	1	968.0204
29784	8	7	53.3	5079	1	1	1	1216.1233
29784	8	7	53.3	5129	1	1	1	1242.6153
29784	8	7	53.3	5589	1	1	1	938.5068
29784	8	7	53.3	5736	1	1	1	982.8298
29784	8	7	53.3	6437	1	1	1	1026.2778
10285	8	5	54.6	489	1	1	1	460.7468
10285	8	5	54.6	553	1	1	1	471.2582
10285	8	5	54.6	2468	1	1	1	720.3466
10285	8	5	54.6	2674	1	1	1	758.3673
10285	8	5	54.6	4392	1	1	1	1078.5152
120769	12	11	13.8	1176	1	1	1	550.8374
120769	12	11	13.8	1712	1	1	1	617.3159
120769	12	11	13.8	1769	1	1	1	624.798
120769	12	11	13.8	1814	1	1	1	629.8554
120769	12	11	13.8	1883	1	1	1	639.3401
120769	12	11	13.8	1912	1	1	1	641.3162
120769	12	11	13.8	2198	1	1	1	682.8433
120769	12	11	13.8	2363	1	1	1	703.792
120769	12	11	13.8	2788	1	1	1	778.4375
120769	12	11	13.8	4509	1	1	1	735.0511
120769	12	11	13.8	5133	1	1	1	830.4464
59765	13	9	22.8	696	1	1	0	488.2844
59765	13	9	22.8	1480	1	1	1	587.3348
59765	13	9	22.8	1897	1	1	1	639.8215
59765	13	9	22.8	2302	1	1	1	697.4112
59765	13	9	22.8	2428	1	1	1	713.3905
59765	13	9	22.8	2519	1	1	1	729.4236
59765	13	9	22.8	2599	1	1	1	746.8908
59765	13	9	22.8	3623	1	1	1	932.9753
59765	13	9	22.8	5330	1	1	1	896.7966
31696	12	10	44.4	307	1	0	0	429.2456
31696	12	10	44.4	348	1	0	0	436.2541
31696	12	10	44.4	582	1	1	1	475.2769
31696	12	10	44.4	1074	1	1	1	537.7745

31696	12	10	44.4	1664	1	1	1	610.3311
31696	12	10	44.4	2306	1	1	1	697.8798
31696	12	10	44.4	2373	1	1	1	704.8827
31696	12	10	44.4	2823	1	1	1	788.4223
31696	12	10	44.4	3948	1	1	1	984.4434
31696	12	10	44.4	5407	1	1	1	912.7852
63798	13	12	27.2	27	1	1	1	360.2262
63798	13	12	27.2	413	1	1	1	449.292
63798	13	12	27.2	1412	1	0	0	580.2546
63798	13	12	27.2	1518	1	1	1	592.7719
63798	13	12	27.2	1538	1	0	0	594.3551
63798	13	12	27.2	2582	1	1	1	495.5682
63798	13	12	27.2	2749	1	1	1	513.2858
63798	13	12	27.2	2767	1	1	1	774.9043
63798	13	12	27.2	3210	1	0	0	860.8774
63798	13	12	27.2	3410	1	1	1	898.8766
63798	13	12	27.2	3768	1	0	1	952.9825
63798	13	12	27.2	5054	1	0	0	1205.571
72129	7	6	14.8	1948	1	1	1	646.379
72129	7	6	14.8	2486	1	1	1	722.406
72129	7	6	14.8	3345	1	1	1	886.0199
72129	7	6	14.8	3740	1	1	1	947.4667
72129	7	6	14.8	3826	1	1	1	963.0166
72129	7	6	14.8	5170	1	1	1	843.0563
109501	9	8	10.7	628	1	1	1	480.7974
109501	9	8	10.7	818	1	1	1	503.7882
109501	9	8	10.7	932	1	1	1	518.782
109501	9	8	10.7	1305	1	1	1	567.3317
109501	9	8	10.7	2585	1	1	1	743.9272
109501	9	8	10.7	3273	1	1	1	874.4511
109501	9	8	10.7	3795	1	1	1	957.9873
109501	9	8	10.7	5107	1	1	1	1231.1429
44356	7	6	24.6	883	1	1	1	512.7433
44356	7	6	24.6	1086	1	1	1	539.2657
44356	7	6	24.6	1896	1	1	1	639.8161
44356	7	6	24.6	3851	1	1	1	967.4698
44356	7	6	24.6	4497	1	1	1	1101.5516
44356	7	6	24.6	6201	1	1	1	1125.5311
83408	10	10	16.9	753	1	1	1	495.7842
83408	10	10	16.9	956	1	1	1	521.751
83408	10	10	16.9	1723	1	1	1	618.8243
83408	10	10	16.9	1808	1	1	1	628.8295
83408	10	10	16.9	2075	1	1	1	442.565
83408	10	10	16.9	2547	1	1	1	735.9007
83408	10	10	16.9	2990	1	1	1	823.4196
83408	10	10	16.9	2999	1	1	1	825.4008
83408	10	10	16.9	3041	1	1	1	833.4274
83408	10	10	16.9	3200	1	1	1	572.9799
65220	9	6	17	1356	1	1	1	573.8037
65220	9	6	17	1731	1	1	1	619.7949
65220	9	6	17	3016	1	1	1	829.4138
65220	9	6	17	3231	1	1	1	865.9202
65220	9	6	17	4413	1	1	1	1083.5525
65220	9	6	17	5323	1	1	1	894.1204
44974	7	6	25.1	2247	1	1	1	690.3619
44974	7	6	25.1	2339	1	1	1	701.3682
44974	7	6	25.1	2765	1	1	1	773.8977
44974	7	6	25.1	2927	1	1	1	809.9345

44974	7	6	25.1	4395	1	1	1	1079.4994
44974	7	6	25.1	5801	1	1	1	1014.1701
40949	9	8	25.9	188	1	0	0	408.7505
40949	9	8	25.9	1486	1	0	0	587.8105
40949	9	8	25.9	1674	1	0	0	611.8189
40949	9	8	25.9	2571	1	0	0	740.3928
40949	9	8	25.9	2673	1	0	1	758.3623
40949	9	8	25.9	3429	1	1	1	900.9721
40949	9	8	25.9	3501	1	1	1	608.3263
40949	9	8	25.9	4393	1	0	0	719.6976
20667	8	7	53.6	1139	1	1	1	545.318
20667	8	7	53.6	1189	1	1	1	552.3098
20667	8	7	53.6	2803	1	1	1	783.3834
20667	8	7	53.6	2993	1	1	1	823.8878
20667	8	7	53.6	4257	1	1	1	702.7014
20667	8	7	53.6	4378	1	1	1	718.3514
20667	8	7	53.6	4907	1	1	1	1162.124
44960	11	10	30.6	50	1	1	0	369.7472
44960	11	10	30.6	524	1	1	0	466.2765
44960	11	10	30.6	1410	1	0	0	579.8293
44960	11	10	30.6	1864	1	1	0	637.3246
44960	11	10	30.6	1879	1	1	0	638.8489
44960	11	10	30.6	2025	1	1	0	656.7965
44960	11	10	30.6	2298	1	1	0	696.8827
44960	11	10	30.6	2326	1	1	0	699.8286
44960	11	10	30.6	3864	1	1	1	968.9875
44960	11	10	30.6	4585	1	1	0	741.7413
58865	9	8	22.4	632	1	1	1	481.2713
58865	9	8	22.4	1737	1	1	1	620.8196
58865	9	8	22.4	2320	1	1	1	466.2504
58865	9	8	22.4	2349	1	1	1	702.3623
58865	9	8	22.4	2882	1	1	1	801.858
58865	9	8	22.4	3203	1	1	1	859.4498
58865	9	8	22.4	3837	1	1	1	964.9996
58865	9	8	22.4	5716	1	1	1	978.1474
41876	12	9	35	158	1	0	0	400.7718
41876	12	9	35	698	1	0	0	488.7276
41876	12	9	35	852	1	1	0	507.7442
41876	12	9	35	1436	1	0	0	582.3023
41876	12	9	35	2675	1	0	0	758.3777
41876	12	9	35	3895	1	0	0	974.94
41876	12	9	35	3910	1	0	0	977.5349
41876	12	9	35	4545	1	1	0	1107.0481
41876	12	9	35	5927	1	0	0	1051.2186
125058	12	11	18.5	782	1	1	1	499.3238
125058	12	11	18.5	1455	1	1	1	583.8061
125058	12	11	18.5	1991	1	1	1	651.9041
125058	12	11	18.5	4422	1	1	1	1085.6006
125058	12	11	18.5	4439	1	1	1	1089.0721
125058	12	11	18.5	4490	1	1	1	733.7071
125058	12	11	18.5	4988	1	1	1	1190.5692
125058	12	11	18.5	5145	1	1	1	1252.6302
125058	12	11	18.5	5240	1	1	1	864.4177
125058	12	11	18.5	5518	1	1	1	696.8503
125058	12	11	18.5	5554	1	1	1	933.8481
22118	10	9	57.7	414	1	1	1	449.7533
22118	10	9	57.7	1468	1	1	1	585.8052
22118	10	9	57.7	1740	1	1	1	620.8558

22118	10	9	57.7	2235	1	1	1	688.3839
22118	10	9	57.7	2839	1	1	1	791.9307
22118	10	9	57.7	3400	1	1	1	598.3051
22118	10	9	57.7	3629	1	1	1	933.4738
22118	10	9	57.7	3638	1	1	1	623.335
22118	10	9	57.7	3796	1	1	1	639.0106
67582	8	7	17.6	1419	1	1	1	580.8332
67582	8	7	17.6	1516	1	1	1	592.3192
67582	8	7	17.6	1621	1	1	1	604.3375
67582	8	7	17.6	1820	1	1	1	630.8452
67582	8	7	17.6	2091	1	1	1	665.86
67582	8	7	17.6	2715	1	1	1	764.9321
67582	8	7	17.6	6322	1	1	1	1204.5782
73174	11	10	18.2	308	1	1	1	429.2633
73174	11	10	18.2	727	1	0	0	492.7896
73174	11	10	18.2	1287	1	1	1	565.7876
73174	11	10	18.2	1545	1	1	1	595.8425
73174	11	10	18.2	1720	1	1	1	618.2908
73174	11	10	18.2	1802	1	0	0	628.8126
73174	11	10	18.2	2403	1	1	1	709.3671
73174	11	10	18.2	2448	1	0	0	717.3686
73174	11	10	18.2	2911	1	1	1	538.6291
73174	11	10	18.2	5101	1	1	1	1228.7036
55711	7	7	22.5	1264	1	1	1	562.2932
55711	7	7	22.5	1830	1	1	1	421.8862
55711	7	7	22.5	2036	1	1	1	658.8381
55711	7	7	22.5	2797	1	1	1	780.9084
55711	7	7	22.5	4273	1	1	1	1058.5105
55711	7	7	22.5	4983	1	1	1	1188.5606
55711	7	7	22.5	5096	1	1	1	817.3916
42056	11	10	39.8	158	1	0	0	400.7718
42056	11	10	39.8	698	1	0	0	488.7276
42056	11	10	39.8	1294	1	1	0	566.767
42056	11	10	39.8	1436	1	0	0	582.3023
42056	11	10	39.8	2675	1	0	0	758.3777
42056	11	10	39.8	3396	1	1	1	895.9478
42056	11	10	39.8	3953	1	0	1	657.0319
42056	11	10	39.8	3966	1	1	0	659.2991
42056	11	10	39.8	4545	1	0	0	1107.0481
42056	11	10	39.8	5927	1	0	0	1051.2186
137407	9	8	8.1	739	1	1	1	494.7458
137407	9	8	8.1	924	1	1	1	518.2829
137407	9	8	8.1	1605	1	1	1	603.2808
137407	9	8	8.1	1775	1	1	1	625.3124
137407	9	8	8.1	2272	1	1	1	692.3793
137407	9	8	8.1	2612	1	1	1	748.922
137407	9	8	8.1	2925	1	1	1	809.9277
137407	9	8	8.1	3792	1	1	1	957.0774
47357	9	9	28	547	1	1	1	470.7765
47357	9	9	28	1849	1	1	1	634.809
47357	9	9	28	2018	1	1	0	655.3793
47357	9	9	28	2183	1	1	0	680.3979
47357	9	9	28	2226	1	1	1	686.8794
47357	9	9	28	3008	1	1	0	827.4098
47357	9	9	28	3061	1	1	1	838.9143
47357	9	9	28	3774	1	1	1	953.5119
47357	9	9	28	3898	1	1	1	975.4372
65977	9	8	17.4	255	1	1	1	420.2368

65977	9	8	17.4	1504	1	1	1	591.2936
65977	9	8	17.4	1938	1	1	1	644.8587
65977	9	8	17.4	2376	1	1	1	705.3523
65977	9	8	17.4	2717	1	1	1	765.4109
65977	9	8	17.4	2745	1	1	1	512.9919
65977	9	8	17.4	2867	1	1	1	532.2948
65977	9	8	17.4	3496	1	1	1	910.9609
59440	8	8	22.2	458	1	1	1	456.2521
59440	8	8	22.2	1845	1	1	1	634.3714
59440	8	8	22.2	2213	1	0	0	685.3193
59440	8	8	22.2	2829	1	1	1	526.6225
59440	8	8	22.2	2916	1	0	0	807.9607
59440	8	8	22.2	3045	1	0	0	835.4178
59440	8	8	22.2	4617	1	1	1	743.758
59440	8	8	22.2	5137	1	1	1	831.4548
64169	6	5	12.3	1514	1	1	0	591.8583
64169	6	5	12.3	1582	1	1	1	600.3352
64169	6	5	12.3	1910	1	1	0	640.8874
64169	6	5	12.3	2783	1	1	0	776.8809
64169	6	5	12.3	5205	1	1	1	1285.2104
86221	9	8	12.4	77	1	1	1	378.2244
86221	9	8	12.4	892	1	1	1	513.8008
86221	9	8	12.4	962	1	1	1	522.2905
86221	9	8	12.4	1649	1	1	1	608.816
86221	9	8	12.4	1746	1	1	1	414.8763
86221	9	8	12.4	1780	1	1	1	625.8221
86221	9	8	12.4	2093	1	1	1	665.8822
86221	9	8	12.4	4878	1	1	1	1154.5816
47005	9	8	26.7	589	1	1	1	475.7692
47005	9	8	26.7	1660	1	1	1	406.5847
47005	9	8	26.7	1950	1	1	1	431.558
47005	9	8	26.7	2603	1	1	1	747.3686
47005	9	8	26.7	2769	1	1	1	774.9479
47005	9	8	26.7	3928	1	1	1	654.6624
47005	9	8	26.7	4239	1	1	1	699.3958
47005	9	8	26.7	4897	1	1	1	1159.5237
62339	9	9	19.7	122	1	1	0	393.2604
62339	9	9	19.7	218	1	1	0	413.755
62339	9	9	19.7	858	1	1	0	508.7665
62339	9	9	19.7	1572	1	1	0	598.8716
62339	9	9	19.7	1842	1	1	0	634.2915
62339	9	9	19.7	2398	1	1	1	708.3952
62339	9	9	19.7	2702	1	1	0	762.884
62339	9	9	19.7	3023	1	1	0	831.4047
62339	9	9	19.7	5253	1	1	1	873.1451
47042	9	9	37.6	256	1	1	1	420.763
47042	9	9	37.6	1037	1	1	0	533.3025
47042	9	9	37.6	2215	1	1	1	685.8473
47042	9	9	37.6	2407	1	1	1	710.3573
47042	9	9	37.6	2480	1	1	0	722.3346
47042	9	9	37.6	4403	1	1	1	721.3118
47042	9	9	37.6	5390	1	1	1	907.7922
47042	9	9	37.6	5792	1	1	1	1007.7668
47042	9	9	37.6	6252	1	1	1	858.9364
43168	11	11	38.4	91	1	1	1	383.2162
43168	11	11	38.4	354	1	1	1	437.2618
43168	11	11	38.4	705	1	1	1	490.2403
43168	11	11	38.4	1615	1	1	1	603.8068

43168	11	11	38.4	1970	1	1	1	432.9306
43168	11	11	38.4	2511	1	1	1	727.3805
43168	11	11	38.4	2620	1	1	1	751.8641
43168	11	11	38.4	3028	1	1	1	831.9226
43168	11	11	38.4	3457	1	1	1	905.4186
43168	11	11	38.4	4992	1	1	1	795.0952
43168	11	11	38.4	5283	1	1	1	883.7834
47389	8	7	25.5	1410	1	0	0	579.8293
47389	8	7	25.5	2129	1	1	1	671.3181
47389	8	7	25.5	2574	1	1	1	493.9593
47389	8	7	25.5	3360	1	1	1	592.6292
47389	8	7	25.5	3745	1	1	1	948.4562
47389	8	7	25.5	3868	1	1	1	970.4803
47389	8	7	25.5	5081	1	1	1	811.7602
116744	5	5	7.5	517	1	1	1	465.2926
116744	5	5	7.5	2120	1	1	1	669.8689
116744	5	5	7.5	2270	1	1	1	692.3498
116744	5	5	7.5	4557	1	1	1	739.7753
116744	5	5	7.5	4871	1	1	1	1152.0987
123905	6	6	6.8	961	1	1	1	522.2854
123905	6	6	6.8	2131	1	1	1	671.3378
123905	6	6	6.8	2197	1	1	1	682.4027
123905	6	6	6.8	3048	1	1	1	836.4709
123905	6	6	6.8	5098	1	1	1	1225.6447
123905	6	6	6.8	5386	1	1	1	907.4945
59477	5	5	18.2	1569	1	1	1	598.3265
59477	5	5	18.2	3430	1	1	1	900.9997
59477	5	5	18.2	3595	1	1	1	928.0329
59477	5	5	18.2	4584	1	1	1	1112.0987
59477	5	5	18.2	5984	1	1	1	1068.2241
42064	11	9	31.6	158	1	0	0	400.7718
42064	11	9	31.6	698	1	0	0	488.7276
42064	11	9	31.6	852	1	0	0	507.7442
42064	11	9	31.6	1436	1	0	0	582.3023
42064	11	9	31.6	1555	1	0	0	596.7684
42064	11	9	31.6	2675	1	0	0	758.3777
42064	11	9	31.6	3355	1	0	0	887.9518
42064	11	9	31.6	3855	1	1	0	645.6233
153955	7	7	7.5	1946	1	1	1	646.3483
153955	7	7	7.5	1966	1	1	1	648.4242
153955	7	7	7.5	2236	1	1	1	688.4134
153955	7	7	7.5	3875	1	1	1	648.3701
153955	7	7	7.5	4078	1	1	1	1015.07
153955	7	7	7.5	4191	1	1	1	1037.567
153955	7	7	7.5	5272	1	1	1	879.1386
40331	5	5	22.2	1541	1	1	1	595.3243
40331	5	5	22.2	2136	1	1	1	672.3616
40331	5	5	22.2	2369	1	1	1	704.8406
40331	5	5	22.2	3504	1	1	1	912.48
40331	5	5	22.2	5125	1	1	1	1240.5409
44425	7	6	22.5	702	1	1	1	489.7667
44425	7	6	22.5	874	1	1	1	511.2872
44425	7	6	22.5	2164	1	1	1	677.4026
44425	7	6	22.5	2719	1	1	1	766.3647
44425	7	6	22.5	3093	1	1	1	841.9223
44425	7	6	22.5	5878	1	1	1	1036.8457
100826	10	9	16.9	1212	1	1	1	555.274
100826	10	9	16.9	1362	1	1	1	574.8013

100826	10	9	16.9	1983	1	1	1	651.3286
100826	10	9	16.9	2600	1	1	1	746.9033
100826	10	9	16.9	2759	1	1	1	515.2841
100826	10	9	16.9	4120	1	1	1	1023.0552
100826	10	9	16.9	5154	1	1	1	837.682
100826	10	9	16.9	5662	1	1	1	962.844
100826	10	9	16.9	5887	1	1	1	779.1661
98576	7	6	8.6	937	1	0	0	519.2693
98576	7	6	8.6	1800	1	0	0	628.3411
98576	7	6	8.6	1881	1	0	0	639.3143
98576	7	6	8.6	2352	1	0	0	702.4366
98576	7	6	8.6	2805	1	0	0	783.4575
98576	7	6	8.6	3155	1	1	1	851.5133
57763	10	9	25.6	14	1	1	1	356.7213
57763	10	9	25.6	1476	1	1	1	587.3002
57763	10	9	25.6	2054	1	1	1	440.8809
57763	10	9	25.6	2327	1	1	1	699.8429
57763	10	9	25.6	2710	1	1	1	763.8906
57763	10	9	25.6	3836	1	1	1	643.6541
57763	10	9	25.6	4789	1	1	1	1136.5868
57763	10	9	25.6	5165	1	1	1	841.4655
57763	10	9	25.6	6086	1	1	1	1107.5528
219009	5	5	3.6	1184	1	1	1	551.8349
219009	5	5	3.6	2024	1	1	1	656.3978
219009	5	5	3.6	2762	1	1	1	772.9028
219009	5	5	3.6	3225	1	1	1	863.428
219009	5	5	3.6	4921	1	1	1	778.4284
61584	8	8	15.3	122	1	0	0	393.2604
61584	8	8	15.3	218	1	0	0	413.755
61584	8	8	15.3	858	1	0	0	508.7665
61584	8	8	15.3	1572	1	0	0	598.8716
61584	8	8	15.3	1842	1	0	0	634.2915
61584	8	8	15.3	2406	1	1	1	709.8504
61584	8	8	15.3	2702	1	0	0	762.884
61584	8	8	15.3	3023	1	0	0	831.4047
39964	6	5	18	1561	1	0	0	597.335
39964	6	5	18	1653	1	0	0	609.306
39964	6	5	18	2418	1	0	0	712.3355
39964	6	5	18	2544	1	1	1	735.4066
39964	6	5	18	4652	1	1	1	1118.5751
34389	5	5	26.9	259	1	1	1	421.2662
34389	5	5	26.9	2071	1	1	1	662.8954
34389	5	5	26.9	2877	1	1	1	800.4107
34389	5	5	26.9	3074	1	1	1	839.9171
34389	5	5	26.9	6059	1	1	1	1101.5703
90209	7	6	10.3	1515	1	1	1	592.303
90209	7	6	10.3	1575	1	1	1	599.2982
90209	7	6	10.3	2021	1	1	1	655.8255
90209	7	6	10.3	2598	1	1	1	746.4067
90209	7	6	10.3	2738	1	1	1	768.3675
90209	7	6	10.3	5045	1	1	1	1204.1301
44788	7	7	20.4	554	1	1	0	471.28
44788	7	7	20.4	922	1	1	1	517.7687
44788	7	7	20.4	2487	1	1	0	482.2237
44788	7	7	20.4	2816	1	1	0	524.9218
44788	7	7	20.4	2906	1	1	0	807.4055
44788	7	7	20.4	3839	1	1	1	965.4065
44788	7	7	20.4	4427	1	1	0	1086.5388

45894	7	7	23	759	1	1	1	496.7741
45894	7	7	23	1410	1	0	0	579.8293
45894	7	7	23	1856	1	1	1	424.5668
45894	7	7	23	1940	1	1	1	645.3055
45894	7	7	23	2411	1	1	1	474.2806
45894	7	7	23	4076	1	1	1	1015.0332
45894	7	7	23	4185	1	1	1	1035.4696
80287	7	6	11.9	1809	1	1	0	628.8516
80287	7	6	11.9	1944	1	1	0	646.3225
80287	7	6	11.9	2056	1	1	0	660.8327
80287	7	6	11.9	2713	1	1	1	764.4039
80287	7	6	11.9	2841	1	1	1	528.6018
80287	7	6	11.9	4664	1	1	1	1120.5792
40463	7	5	16.3	336	1	1	1	435.2531
40463	7	5	16.3	764	1	1	1	497.2813
40463	7	5	16.3	1173	1	1	1	550.7581
40463	7	5	16.3	2828	1	1	1	789.3995
40463	7	5	16.3	4334	1	1	1	1069.5954
34426	5	3	16.1	973	1	1	0	523.2981
34426	5	3	16.1	2390	1	1	0	706.4233
34426	5	3	16.1	5773	1	1	1	997.5596
34394	3	2	8.6	669	1	1	1	485.3078
34394	3	2	8.6	3146	1	1	1	849.9857
73869	5	5	9.7	1394	1	1	1	578.3275
73869	5	5	9.7	1802	1	0	0	628.8126
73869	5	5	9.7	2188	1	1	1	681.3236
73869	5	5	9.7	2607	1	1	1	747.8647
73869	5	5	9.7	4142	1	1	1	1027.0096
27673	3	3	16.8	1937	1	1	1	644.8377
27673	3	3	16.8	2419	1	1	1	712.3385
27673	3	3	16.8	3874	1	1	1	971.9985
44808	6	6	17.9	554	1	0	0	471.28
44808	6	6	17.9	2487	1	0	0	482.2237
44808	6	6	17.9	2816	1	0	0	524.9218
44808	6	6	17.9	2906	1	0	0	807.4055
44808	6	6	17.9	3013	1	1	1	827.9534
44808	6	6	17.9	4427	1	0	0	1086.5388
182693	3	3	2.5	1228	1	1	1	557.3293
182693	3	3	2.5	2478	1	1	1	721.922
182693	3	3	2.5	3480	1	1	1	908.4739
67832	4	4	7.6	1277	1	1	1	563.8575
67832	4	4	7.6	1725	1	1	1	618.8506
67832	4	4	7.6	2078	1	1	1	663.8562
67832	4	4	7.6	2375	1	1	1	704.8953
25092	5	5	29.3	654	1	1	0	483.2505
25092	5	5	29.3	1763	1	1	0	623.3445
25092	5	5	29.3	2798	1	1	1	781.365
25092	5	5	29.3	3014	1	1	1	829.3756
25092	5	5	29.3	3161	1	1	1	852.9483
48286	5	5	13.6	381	1	1	1	441.7113
48286	5	5	13.6	1741	1	1	1	621.3302
48286	5	5	13.6	1839	1	1	1	633.8397
48286	5	5	13.6	2364	1	1	1	704.3096
48286	5	5	13.6	3250	1	1	1	869.4271
100050	5	5	8.8	1661	1	1	0	609.8398
100050	5	5	8.8	2227	1	1	0	686.8973
100050	5	5	8.8	2919	1	1	0	808.4564
100050	5	5	8.8	4913	1	1	0	776.4274

100050	5	5	8.8	5162	1	1	1	841.4386
71719	4	3	8.5	1716	1	1	1	617.7867
71719	4	3	8.5	4096	1	1	1	1018.4944
71719	4	3	8.5	5314	1	1	1	1339.6332
63627	5	5	12.4	1514	1	0	0	591.8583
63627	5	5	12.4	1702	1	1	1	615.3529
63627	5	5	12.4	1910	1	0	0	640.8874
63627	5	5	12.4	2783	1	0	0	776.8809
63627	5	5	12.4	5242	1	1	1	865.8129
45355	3	2	7.4	2374	1	1	1	704.8867
45355	3	2	7.4	3587	1	1	1	927.0352
66087	3	2	4.8	2758	1	1	1	772.3875
66087	3	2	4.8	3100	1	1	1	842.4803
99776	5	5	8.4	1661	1	0	0	609.8398
99776	5	5	8.4	2227	1	0	0	686.8973
99776	5	5	8.4	2919	1	0	0	808.4564
99776	5	5	8.4	3632	1	1	1	933.5587
99776	5	5	8.4	4913	1	0	0	776.4274
118478	5	5	7.6	443	1	1	0	453.7558
118478	5	5	7.6	1801	1	1	1	628.3578
118478	5	5	7.6	3163	1	1	1	852.9985
118478	5	5	7.6	4467	1	1	1	729.7276
118478	5	5	7.6	5699	1	1	1	974.2085
60195	5	3	13.3	4813	1	1	1	761.0587
60195	5	3	13.3	4895	1	1	1	772.7418
60195	5	3	13.3	5907	1	1	1	1045.8528
264216	5	4	2.7	1834	1	1	1	633.2959
264216	5	4	2.7	2370	1	1	1	704.8469
264216	5	4	2.7	3881	1	1	1	973.0227
264216	5	4	2.7	4922	1	1	1	778.7529
44816	11	9	24.7	373	1	1	1	440.7303
44816	11	9	24.7	541	1	1	1	469.2824
44816	11	9	24.7	724	1	1	1	492.2748
44816	11	9	24.7	916	1	1	1	517.2858
44816	11	9	24.7	1777	1	1	1	625.3209
44816	11	9	24.7	1786	1	1	1	626.7977
44816	11	9	24.7	2057	1	1	1	660.8366
44816	11	9	24.7	2869	1	1	1	798.851
44816	11	9	24.7	3339	1	1	1	590.3039
55890	6	6	17.8	639	1	1	1	481.769
55890	6	6	17.8	950	1	1	1	520.3106
55890	6	6	17.8	2258	1	1	1	691.3636
55890	6	6	17.8	2772	1	1	1	517.2879
55890	6	6	17.8	4405	1	1	1	721.642
55890	6	6	17.8	5011	1	1	1	797.4166
92772	3	2	3.8	2278	1	1	1	693.4033
92772	3	2	3.8	4175	1	1	1	1034.0533
22532	4	4	28.2	1061	1	1	0	536.3239
22532	4	4	28.2	2035	1	1	0	658.8335
22532	4	4	28.2	3180	1	1	1	854.9434
22532	4	4	28.2	4390	1	1	1	1078.507
31956	5	5	27	1292	1	1	1	566.3241
31956	5	5	27	1554	1	1	1	596.3524
31956	5	5	27	1837	1	1	1	633.3272
31956	5	5	27	3662	1	1	1	937.4726
31956	5	5	27	5997	1	1	1	1072.2495
17591	5	3	24.3	882	1	1	1	512.2995
17591	5	3	24.3	1019	1	1	1	530.7821

17591	5	3	24.3	4247	1	1	1	1051.0322
23090	3	3	18.8	1559	1	1	0	398.5442
23090	3	3	18.8	2356	1	1	1	702.9024
23090	3	3	18.8	2954	1	1	0	816.4192
29193	4	4	20.1	380	1	1	1	441.2947
29193	4	4	20.1	1985	1	1	1	651.8145
29193	4	4	20.1	2105	1	1	1	667.869
29193	4	4	20.1	4473	1	1	1	1096.5695
49607	4	3	9.3	1641	1	1	1	607.3431
49607	4	3	9.3	2854	1	1	1	794.8993
49607	4	3	9.3	3337	1	0	0	590.2668
32347	5	5	22.5	2229	1	1	1	687.3459
32347	5	5	22.5	2303	1	1	1	697.8316
32347	5	5	22.5	2412	1	1	1	711.3071
32347	5	5	22.5	3050	1	1	1	837.4519
32347	5	5	22.5	3275	1	1	1	874.9625
117103	3	2	3	2182	1	1	1	679.8926
117103	3	2	3	4522	1	1	1	1104.0943
194402	4	4	3.9	1994	1	1	1	652.3407
194402	4	4	3.9	2459	1	1	1	718.3752
194402	4	4	3.9	4108	1	1	1	1020.5624
194402	4	4	3.9	5291	1	1	1	887.8183
99113	3	3	4.5	1761	1	1	1	622.8948
99113	3	3	4.5	1962	1	1	1	647.8628
99113	3	3	4.5	3705	1	1	1	943.0431
52003	6	5	17.7	466	1	1	1	457.2846
52003	6	5	17.7	3055	1	1	1	558.9712
52003	6	5	17.7	3492	1	1	1	607.6079
52003	6	5	17.7	4876	1	1	1	770.0444
52003	6	5	17.7	5126	1	1	1	1241.1694
47202	5	5	19	2018	1	0	0	655.3793
47202	5	5	19	2183	1	0	0	680.3979
47202	5	5	19	3008	1	0	0	827.4098
47202	5	5	19	4648	1	1	1	746.0482
47202	5	5	19	5250	1	1	1	870.1124
30915	3	3	19.5	3237	1	1	1	866.9492
30915	3	3	19.5	3890	1	1	1	973.9811
30915	3	3	19.5	4468	1	1	1	730.0898
77064	4	4	6	343	1	1	1	435.774
77064	4	4	6	1453	1	1	1	583.3162
77064	4	4	6	2256	1	1	1	691.3326
77064	4	4	6	2531	1	1	1	731.3881
27979	4	4	16.1	78	1	1	0	378.2606
27979	4	4	16.1	104	1	1	0	387.7656
27979	4	4	16.1	2654	1	1	0	753.8904
27979	4	4	16.1	2793	1	1	1	779.4264
105384	4	4	6.7	470	1	1	1	458.2609
105384	4	4	6.7	1784	1	1	1	626.3403
105384	4	4	6.7	3721	1	1	1	945.4709
105384	4	4	6.7	5964	1	1	1	1064.8576
15497	2	2	17.9	2212	1	1	1	684.8959
15497	2	2	17.9	2224	1	1	1	686.3957
196586	3	3	2.6	1599	1	1	0	601.8483
196586	3	3	2.6	2743	1	1	1	768.4797
196586	3	3	2.6	4159	1	1	1	1030.0659
32983	4	4	15.4	1011	1	1	1	529.3216
32983	4	4	15.4	1058	1	1	1	536.3059
32983	4	4	15.4	1797	1	1	1	627.851

32983	4	4	15.4	2395	1	1	1	707.8698
271827	6	5	3	339	1	1	1	435.7431
271827	6	5	3	2346	1	1	1	701.8811
271827	6	5	3	3375	1	1	1	891.5096
271827	6	5	3	5049	1	1	1	803.4378
30060	4	4	22.5	1638	1	1	1	607.2847
30060	4	4	22.5	2931	1	1	1	810.448
30060	4	4	22.5	3279	1	1	1	584.2696
30060	4	4	22.5	4153	1	1	1	1029.4864
81293	6	6	9.6	511	1	1	1	464.7768
81293	6	6	9.6	1064	1	1	1	536.8419
81293	6	6	9.6	1113	1	1	1	543.3271
81293	6	6	9.6	1811	1	1	1	629.3253
81293	6	6	9.6	2113	1	1	1	668.8896
81293	6	6	9.6	4388	1	1	1	718.7027
20619	3	3	23.4	2467	1	1	1	719.9355
20619	3	3	23.4	2987	1	1	1	822.9331
20619	3	3	23.4	3042	1	1	1	834.3935
59426	4	4	12.1	1631	1	1	1	605.8449
59426	4	4	12.1	1840	1	1	1	633.8558
59426	4	4	12.1	4798	1	1	1	1139.1599
59426	4	4	12.1	5014	1	1	1	1196.1324
23230	4	4	21.2	79	1	1	1	378.7339
23230	4	4	21.2	1431	1	1	1	581.8266
23230	4	4	21.2	2035	1	0	1	658.8335
23230	4	4	21.2	2575	1	1	1	740.8861
23990	4	4	21.8	1061	1	0	0	536.3239
23990	4	4	21.8	1445	1	1	1	582.798
23990	4	4	21.8	2035	1	0	0	658.8335
23990	4	4	21.8	2903	1	1	1	805.9248
49361	2	2	6.6	1429	1	1	1	581.814
49361	2	2	6.6	3773	1	1	1	953.5039
20970	3	3	18.2	269	1	1	1	422.2504
20970	3	3	18.2	1506	1	1	1	591.3536
20970	3	3	18.2	2775	1	1	1	775.8845
29898	6	5	18.4	85	1	1	1	380.7214
29898	6	5	18.4	567	1	1	1	472.7741
29898	6	5	18.4	651	1	1	1	482.7769
29898	6	5	18.4	1684	1	1	1	613.3708
29898	6	5	18.4	2205	1	1	1	683.8425
40903	2	2	8.3	2918	1	1	1	808.4272
40903	2	2	8.3	3361	1	1	1	888.942
40630	4	4	12.7	791	1	1	1	500.7923
40630	4	4	12.7	1857	1	1	1	636.3562
40630	4	4	12.7	1926	1	1	1	643.3628
40630	4	4	12.7	2397	1	1	1	708.3851
52150	4	4	10.7	1129	1	1	1	544.7921
52150	4	4	10.7	1747	1	1	1	621.8216
52150	4	4	10.7	2578	1	1	1	741.9377
52150	4	4	10.7	3120	1	1	1	564.6138
148890	3	3	3.1	1116	1	1	1	543.8004
148890	3	3	3.1	2706	1	1	1	763.4565
148890	3	3	3.1	3871	1	1	1	971.0328
32586	3	3	14.4	2540	1	1	1	733.8852
32586	3	3	14.4	2813	1	1	1	784.8655
32586	3	3	14.4	3997	1	1	1	994.4968
57853	3	2	6.3	3301	1	1	1	879.9962
57853	3	2	6.3	4051	1	1	1	671.0555

23969	5	5	24.8	564	1	1	0	472.7453
23969	5	5	24.8	963	1	1	1	522.3084
23969	5	5	24.8	1097	1	1	0	540.7878
23969	5	5	24.8	1629	1	1	1	605.8247
23969	5	5	24.8	2104	1	1	1	667.3795
51204	5	5	14	968	1	1	1	523.2706
51204	5	5	14	1024	1	1	1	531.7776
51204	5	5	14	1132	1	1	1	544.8268
51204	5	5	14	3114	1	1	1	845.4075
51204	5	5	14	4492	1	1	1	1100.5378
39436	3	3	11.3	1841	1	1	1	633.8746
39436	3	3	11.3	2670	1	1	1	757.4146
39436	3	3	11.3	3158	1	1	1	852.4235
94470	4	4	6.9	1760	1	1	1	622.8634
94470	4	4	6.9	2583	1	1	1	742.958
94470	4	4	6.9	3603	1	1	0	620.3172
94470	4	4	6.9	3970	1	1	1	988.9768
38472	3	3	11.1	596	1	1	1	476.7577
38472	3	3	11.1	2433	1	1	1	713.8854
38472	3	3	11.1	3306	1	1	1	880.954
114571	4	4	5.4	350	1	1	1	436.7454
114571	4	4	5.4	2692	1	1	1	760.3989
114571	4	4	5.4	2784	1	1	1	518.2824
114571	4	4	5.4	4226	1	1	1	697.3573
17358	2	2	27	4556	1	1	1	555.0277
17358	2	2	27	5234	1	1	1	1293.6444
17889	4	4	26.9	717	1	1	1	491.2922
17889	4	4	26.9	1036	1	1	1	533.2821
17889	4	4	26.9	1044	1	1	0	534.3136
17889	4	4	26.9	3316	1	1	1	882.4474
28210	5	5	22.3	78	1	0	0	378.2606
28210	5	5	22.3	104	1	0	0	387.7656
28210	5	5	22.3	1359	1	1	1	574.3089
28210	5	5	22.3	2654	1	0	0	753.8904
28210	5	5	22.3	5078	1	1	1	608.0713
82214	2	2	3.9	2132	1	1	1	671.385
82214	2	2	3.9	2955	1	1	1	816.4415
110942	5	5	7.2	1701	1	1	1	615.293
110942	5	5	7.2	1703	1	1	1	615.366
110942	5	5	7.2	2319	1	1	1	698.8695
110942	5	5	7.2	3712	1	1	1	943.9651
110942	5	5	7.2	3980	1	1	1	661.6809
62205	4	3	7.7	1812	1	1	1	629.3561
62205	4	3	7.7	1942	1	1	1	645.812
62205	4	3	7.7	4703	1	1	1	1125.6507
46359	4	3	14.7	3579	1	1	1	924.9879
46359	4	3	14.7	3686	1	1	1	627.5899
46359	4	3	14.7	5914	1	1	1	1047.5493
32499	3	3	15.4	548	1	1	1	470.7952
32499	3	3	15.4	4210	1	1	1	694.6494
32499	3	3	15.4	4476	1	1	1	1097.0177
45049	4	4	10.2	1012	1	1	1	529.3343
45049	4	4	10.2	1136	1	1	1	545.2792
45049	4	4	10.2	1282	1	1	1	564.8043
45049	4	4	10.2	1291	1	1	1	566.2853
16285	3	3	23.3	1001	1	1	1	527.7849
16285	3	3	23.3	1391	1	1	1	577.8296
16285	3	3	23.3	2482	1	1	1	722.3686

15188	2	2	21.4	420	1	1	1	450.7609
15188	2	2	21.4	4037	1	1	1	1003.4876
71322	2	2	3.9	806	1	1	1	502.7981
71322	2	2	3.9	3691	1	1	1	941.4736
111275	2	2	3.5	2053	1	1	1	660.8165
111275	2	2	3.5	4956	1	1	1	787.0898
81345	2	2	5.3	2665	1	1	1	504.6192
81345	2	2	5.3	5262	1	1	1	876.7772
146599	6	6	6.8	1215	1	1	1	555.7563
146599	6	6	6.8	1397	1	1	1	578.8179
146599	6	6	6.8	1721	1	1	1	618.3225
146599	6	6	6.8	2074	1	1	1	663.3386
146599	6	6	6.8	2983	1	1	1	547.9241
146599	6	6	6.8	5829	1	1	1	1022.5644
47036	4	4	13.8	256	1	0	1	420.763
47036	4	4	13.8	1037	1	0	0	533.3025
47036	4	4	13.8	2480	1	0	0	722.3346
47036	4	4	13.8	6153	1	1	1	837.9299
119640	2	2	2.1	1328	1	1	1	570.3609
119640	2	2	2.1	2399	1	1	1	708.3986
8974	3	2	33.8	5259	1	1	1	876.3572
8974	3	2	33.8	5681	1	1	1	968.0789
114479	2	2	2.5	1207	1	1	1	553.8865
114479	2	2	2.5	3291	1	1	1	877.4907
574541	3	2	0.6	1810	1	1	1	628.8822
574541	3	2	0.6	4021	1	1	1	667.0459
50085	2	2	5.6	1603	1	1	1	602.3452
50085	2	2	5.6	2947	1	1	1	814.4113
82296	3	3	5.9	605	1	1	1	477.803
82296	3	3	5.9	1613	1	1	1	603.793
82296	3	3	5.9	4579	1	1	1	1111.0367
23729	2	2	11.7	1757	1	1	1	622.366
23729	2	2	11.7	2297	1	1	1	696.834
65730	2	2	4.6	2353	1	1	1	702.8335
65730	2	2	4.6	2391	1	1	1	706.8782
276563	3	3	1.6	549	1	1	1	470.813
276563	3	3	1.6	4340	1	1	1	714.0595
112934	2	2	3.9	1149	1	1	1	546.8064
112934	2	2	3.9	5841	1	1	1	1025.8601
406216	2	2	0.7	1927	1	1	1	643.3924
406216	2	2	0.7	2781	1	1	1	776.4607
88746	2	2	4.4	1570	1	1	1	598.3271
88746	2	2	4.4	5143	1	1	1	835.4184
114752	4	3	5	781	1	1	1	499.3113
114752	4	3	5	4407	1	1	1	1082.0629
114752	4	3	5	5222	1	1	1	1290.6574
73187	4	3	6.1	1263	1	1	1	562.2877
73187	4	3	6.1	1715	1	1	1	617.3514
73187	4	3	6.1	3695	1	1	1	941.5562
85715	2	2	3.1	1854	1	1	1	635.3924
85715	2	2	3.1	2318	1	1	1	698.853
20989	3	3	17.6	716	1	1	1	491.2619
20989	3	3	17.6	1198	1	0	1	553.2995
20989	3	3	17.6	2728	1	1	1	766.9187
42230	3	3	15.1	2437	1	1	1	714.4031
42230	3	3	15.1	2834	1	1	1	790.4077
42230	3	3	15.1	5986	1	1	1	1068.2311
25357	2	2	10.5	1392	1	1	1	577.8662

25357	2	2	10.5	1872	1	1	1	638.3124
50224	2	2	7.4	2549	1	1	1	736.8969
50224	2	2	7.4	5046	1	1	1	803.1052
57427	2	2	4.8	1414	1	1	1	580.3761
57427	2	2	4.8	2266	1	1	1	691.892
24580	2	2	14.4	2592	1	1	1	745.4169
24580	2	2	14.4	4288	1	1	1	1062.5791
59860	2	2	5	1007	1	1	1	528.8062
59860	2	2	5	3255	1	1	1	870.9864
65767	4	4	10.9	712	1	1	1	490.7741
65767	4	4	10.9	1961	1	1	1	647.8339
65767	4	4	10.9	4001	1	1	1	663.6598
65767	4	4	10.9	5621	1	1	1	948.1675
34706	4	4	22.4	1071	1	1	1	537.319
34706	4	4	22.4	2107	1	1	1	668.3906
34706	4	4	22.4	3963	1	1	1	494.0387
34706	4	4	22.4	6051	1	1	1	822.7002
32980	3	3	13.3	1727	1	1	1	618.8712
32980	3	3	13.3	1739	1	1	0	620.8486
32980	3	3	13.3	3780	1	1	0	636.375
81286	3	3	6.2	1977	1	1	1	649.8816
81286	3	3	6.2	2429	1	1	1	713.4085
81286	3	3	6.2	4903	1	1	1	774.0747
14066	2	2	18.5	1416	1	1	1	580.8168
14066	2	2	18.5	1456	1	1	0	583.8063
60872	2	2	4	623	1	1	1	480.2746
60872	2	2	4	2800	1	1	1	782.3644
55031	2	2	4.6	2099	1	1	1	666.8321
55031	2	2	4.6	2504	1	1	0	725.8839
116128	3	2	3.4	3394	1	1	1	895.0215
116128	3	2	3.4	4515	1	1	1	735.7698
67813	2	2	3.6	1690	1	1	1	613.8583
67813	2	2	3.6	2209	1	1	0	684.8173
166282	3	3	2.3	1325	1	1	1	570.2933
166282	3	3	2.3	1482	1	1	1	587.3399
166282	3	3	2.3	3657	1	1	1	937.4276
43084	2	2	7.4	2846	1	1	1	792.9108
43084	2	2	7.4	2880	1	1	1	800.451
79562	3	2	4.6	2535	1	1	1	732.8616
79562	3	2	4.6	4978	1	1	1	791.3879
93099	3	3	4.3	1635	1	1	1	606.371
93099	3	3	4.3	1918	1	1	1	642.3224
93099	3	3	4.3	2953	1	1	1	815.9474
16590	3	2	15.2	4964	1	1	1	1182.1373
16590	3	2	15.2	5198	1	1	1	854.8044
45968	2	2	5.3	1080	1	1	1	538.2907
45968	2	2	5.3	2165	1	1	1	677.4212
39816	2	2	10.4	1421	1	1	1	580.8429
39816	2	2	10.4	5725	1	1	1	981.1838
53957	2	2	5.4	696	1	0	0	488.2844
53957	2	2	5.4	3903	1	1	1	975.5028
44614	3	3	9.4	1285	1	1	1	565.3219
44614	3	3	9.4	2658	1	1	1	754.3805
44368	3	3	11.4	2476	1	1	0	481.5901
44368	3	3	11.4	4326	1	1	1	1068.5414
29729	2	2	12.8	1048	1	1	1	534.7551
29729	2	2	12.8	5632	1	1	1	951.5047
53612	3	3	8.6	951	1	1	1	520.311

53612	3	3	8.6	2064	1	1	1	662.3344
53612	3	3	8.6	4902	1	1	1	774.0714
20056	3	3	14.2	1121	1	1	1	544.2914
20056	3	3	14.2	1306	1	1	1	567.3318
28578	2	2	10.4	1186	1	1	1	552.2816
28578	2	2	10.4	3508	1	1	1	913.527
122979	2	2	1.9	719	1	1	1	491.7898
122979	2	2	1.9	1969	1	1	1	648.8848
65637	2	2	4.6	2181	1	1	1	679.8587
65637	2	2	4.6	3143	1	1	1	849.8912
41582	3	3	7.8	1337	1	1	1	571.2849
41582	3	3	7.8	1383	1	1	1	576.8016
41582	3	3	7.8	1587	1	1	1	601.319
44164	2	2	6.5	2367	1	1	1	704.3714
44164	2	2	6.5	2476	1	0	0	481.5901
33803	3	3	12.8	1739	1	0	0	620.8486
33803	3	3	12.8	1788	1	1	1	626.8685
33803	3	3	12.8	3780	1	0	0	636.375
58195	4	3	13.8	2217	1	1	1	457.5805
58195	4	3	13.8	5888	1	1	1	1038.8956
58195	4	3	13.8	6261	1	1	1	1150.6421
15174	2	2	18	850	1	1	1	507.2912
15174	2	2	18	3144	1	1	1	849.9149
14095	2	2	18.5	1357	1	1	1	573.8096
14095	2	2	18.5	1456	1	0	0	583.8063
27829	2	2	11.2	1180	1	1	1	551.3111
27829	2	2	11.2	4207	1	1	1	694.2908
53639	4	4	15.1	946	1	1	1	519.7878
53639	4	4	15.1	1161	1	1	1	548.302
53639	4	4	15.1	5261	1	1	1	876.7298
53639	4	4	15.1	5663	1	1	1	963.1275
17142	3	3	27.8	2259	1	1	1	461.2891
17142	3	3	27.8	2296	1	1	1	696.8191
17142	3	3	27.8	3798	1	1	1	639.3473
8231	2	2	34.8	1792	1	1	1	627.3377
8231	2	2	34.8	2830	1	1	1	789.8784
32865	2	2	6.1	536	1	1	1	468.2634
32865	2	2	6.1	1284	1	1	1	565.2928
38757	2	2	8	2498	1	1	1	483.9073
38757	2	2	8	3437	1	1	1	902.9832
68472	2	2	6.7	1657	1	1	1	609.3223
68472	2	2	6.7	5839	1	1	1	1025.5877
109365	3	3	3.7	1233	1	1	1	558.337
109365	3	3	3.7	1698	1	1	0	410.2314
109365	3	3	3.7	2910	1	1	0	538.6248
77313	2	2	3.1	1146	1	1	1	546.3085
77313	2	2	3.1	2350	1	1	1	702.3982
67827	2	2	3.7	1637	1	1	1	606.8504
67827	2	2	3.7	2209	1	0	0	684.8173
18045	2	2	16.9	1848	1	1	1	634.4025
18045	2	2	16.9	3108	1	1	1	563.2726
43105	2	2	7	2275	1	1	1	692.8927
43105	2	2	7	2517	1	1	1	728.8935
24005	3	3	15.2	564	1	0	0	472.7453
24005	3	3	15.2	1097	1	0	0	540.7878
24005	3	3	15.2	3238	1	1	1	578.3261
46504	2	2	8.2	775	1	1	1	498.3471
46504	2	2	8.2	5401	1	1	1	911.1412

97167	2	2	3.2	2095	1	1	1	666.3472
97167	2	2	3.2	3205	1	1	1	573.3657
93010	2	2	2.3	621	1	1	1	479.7696
93010	2	2	2.3	1082	1	1	1	538.7873
23079	3	3	22.7	981	1	1	1	524.2952
23079	3	3	22.7	2048	1	1	1	660.3607
23079	3	3	22.7	5726	1	1	1	981.5092
201443	2	2	1.4	1599	1	0	0	601.8483
201443	2	2	1.4	3234	1	1	1	865.9618
109254	3	3	3.6	1063	1	1	1	536.8294
109254	3	3	3.6	1698	1	0	0	410.2314
109254	3	3	3.6	2910	1	0	0	538.6248
36651	2	2	6.7	1112	1	1	1	543.3135
36651	2	2	6.7	1818	1	1	1	630.778
29708	3	3	15.7	617	1	1	1	479.2841
29708	3	3	15.7	1352	1	1	1	573.3026
29708	3	3	15.7	5652	1	1	1	1442.2201
40742	4	4	12.1	164	1	1	1	403.2324
40742	4	4	12.1	1065	1	1	1	537.2489
40742	4	4	12.1	1658	1	1	1	609.3479
40742	4	4	12.1	3083	1	1	1	560.6541
106684	2	2	2.5	5069	1	1	1	806.7425
106684	2	2	2.5	5215	1	1	1	858.7764
86221	2	2	4.6	2733	1	1	1	767.9347
86221	2	2	4.6	4969	1	1	1	789.8012
12741	2	2	17.4	677	1	1	1	486.2978
12741	2	2	17.4	1707	1	1	1	616.3131
55656	2	2	4.4	1953	1	1	0	647.3372
55656	2	2	4.4	2209	1	0	1	684.8173
43014	2	2	6.1	2191	1	1	1	681.8561
43014	2	2	6.1	2288	1	1	1	694.853
60657	2	2	3.5	480	1	1	1	459.2293
60657	2	2	3.5	1815	1	1	1	630.3332
62798	2	2	5.3	2617	1	1	1	750.9551
62798	2	2	5.3	3603	1	0	0	620.3172
67356	2	2	3.5	1218	1	1	1	555.8209
67356	2	2	3.5	1908	1	1	1	640.8478
15712	2	2	14.1	337	1	1	1	435.2817
15712	2	2	14.1	1487	1	1	1	587.8192
18698	2	2	23.2	1511	1	1	1	591.8297
18698	2	2	23.2	5846	1	1	1	1027.1821
12062	2	2	18.5	99	1	1	0	386.2341
12062	2	2	18.5	2698	1	1	1	508.5801
22104	2	2	15.8	587	1	1	1	475.7688
22104	2	2	15.8	4982	1	1	1	791.7383
92840	2	2	4.4	5496	1	1	1	925.8441
12046	2	2	18.5	99	1	0	0	386.2341
12046	2	2	18.5	2655	1	1	1	503.2484
143018	2	2	1.9	785	1	1	1	499.7973
143018	2	2	1.9	2986	1	1	1	822.4844
prot_mass	prot_matches	prot_sequenc	prot_cover	pep_query	pep_rank	pep_isbold	pep_isunique	pep_exp_mz
50361	47	25	82.7	571	1	1	1	488.7417
50361	47	25	82.7	758	1	1	0	514.2812
50361	47	25	82.7	883	1	1	0	533.2167
50361	47	25	82.7	1160	1	1	0	570.3502
50361	47	25	82.7	1189	1	1	0	573.7982
50361	47	25	82.7	1546	1	1	0	616.2921
50361	47	25	82.7	1704	1	1	0	637.8461

50361	47	25	82.7	2050	1	1	0	686.3451
50361	47	25	82.7	2281	1	1	0	717.8268
50361	47	25	82.7	2417	1	1	1	739.8338
50361	47	25	82.7	2761	1	1	0	811.9293
50361	47	25	82.7	2903	1	1	0	840.4018
50361	47	25	82.7	2953	1	1	0	848.9203
50361	47	25	82.7	3273	1	1	1	907.957
50361	47	25	82.7	3289	1	1	0	911.9651
50361	47	25	82.7	3623	1	1	0	976.4474
50361	47	25	82.7	3673	1	1	0	987.0014
50361	47	25	82.7	3943	1	1	0	1051.0485
50361	47	25	82.7	3958	1	1	0	704.3593
50361	47	25	82.7	4693	1	1	0	1184.0663
50361	47	25	82.7	5232	1	1	0	922.1115
50361	47	25	82.7	5303	1	1	0	933.45
50361	47	25	82.7	5807	1	1	1	801.1239
50361	47	25	82.7	5936	1	1	1	1109.5066
50361	47	25	82.7	6636	1	1	0	1525.377
50310	44	23	77	758	1	0	0	514.2812
50310	44	23	77	883	1	0	0	533.2167
50310	44	23	77	1160	1	0	0	570.3502
50310	44	23	77	1189	1	0	0	573.7982
50310	44	23	77	1546	1	0	0	616.2921
50310	44	23	77	1704	1	0	0	637.8461
50310	44	23	77	1783	1	1	1	649.8221
50310	44	23	77	2050	1	0	0	686.3451
50310	44	23	77	2281	1	0	0	717.8268
50310	44	23	77	2761	1	0	0	811.9293
50310	44	23	77	2903	1	0	0	840.4018
50310	44	23	77	2953	1	0	0	848.9203
50310	44	23	77	3289	1	0	0	911.9651
50310	44	23	77	3623	1	0	0	976.4474
50310	44	23	77	3673	1	0	0	987.0014
50310	44	23	77	3943	1	0	0	1051.0485
50310	44	23	77	3958	1	0	0	704.3593
50310	44	23	77	4693	1	0	0	1184.0663
50310	44	23	77	5232	1	0	0	922.1115
50310	44	23	77	5303	1	0	0	933.45
50310	44	23	77	5756	1	1	1	1051.1345
50310	44	23	77	6105	1	1	0	1133.839
50310	44	23	77	6636	1	0	0	1525.377
51385	48	25	78	883	1	0	0	533.2167
51385	48	25	78	1160	1	0	0	570.3502
51385	48	25	78	1189	1	0	0	573.7982
51385	48	25	78	1546	1	0	0	616.2921
51385	48	25	78	1704	1	0	0	637.8461
51385	48	25	78	1752	1	1	0	644.3341
51385	48	25	78	1779	1	1	1	648.8075
51385	48	25	78	2050	1	0	0	686.3451
51385	48	25	78	2108	1	1	0	692.8545
51385	48	25	78	2317	1	1	1	723.3276
51385	48	25	78	2324	1	1	0	723.8478
51385	48	25	78	2761	1	0	0	811.9293
51385	48	25	78	2903	1	0	0	840.4018
51385	48	25	78	2953	1	0	0	848.9203
51385	48	25	78	3184	1	1	1	889.9353
51385	48	25	78	3289	1	0	0	911.9651
51385	48	25	78	3411	1	1	1	936.9381

51385	48	25	78	3623	1	0	0	976.4474
51385	48	25	78	3673	1	0	0	987.0014
51385	48	25	78	3943	1	0	0	1051.0485
51385	48	25	78	3958	1	0	0	704.3593
51385	48	25	78	5208	1	1	0	917.7797
51385	48	25	78	5315	1	1	1	934.7714
51385	48	25	78	6105	1	0	0	1133.839
51385	48	25	78	6636	1	0	0	1525.377
51341	47	23	73.9	883	1	0	0	533.2167
51341	47	23	73.9	1160	1	0	0	570.3502
51341	47	23	73.9	1189	1	0	0	573.7982
51341	47	23	73.9	1546	1	0	0	616.2921
51341	47	23	73.9	1704	1	0	0	637.8461
51341	47	23	73.9	1752	1	0	0	644.3341
51341	47	23	73.9	1823	1	1	1	654.8248
51341	47	23	73.9	2050	1	0	0	686.3451
51341	47	23	73.9	2273	1	1	0	716.3194
51341	47	23	73.9	2281	1	0	0	717.8268
51341	47	23	73.9	2324	1	0	0	723.8478
51341	47	23	73.9	2761	1	0	0	811.9293
51341	47	23	73.9	2782	1	1	0	817.4
51341	47	23	73.9	2953	1	0	0	848.9203
51341	47	23	73.9	3174	1	1	1	887.4466
51341	47	23	73.9	3512	1	1	1	950.4707
51341	47	23	73.9	3623	1	0	0	976.4474
51341	47	23	73.9	3673	1	0	0	987.0014
51341	47	23	73.9	3943	1	0	0	1051.0485
51341	47	23	73.9	5173	1	1	1	913.1083
51341	47	23	73.9	5348	1	1	0	938.7834
51341	47	23	73.9	6105	1	0	0	1133.839
51341	47	23	73.9	6636	1	0	0	1525.377
51258	46	23	71.3	883	1	0	0	533.2167
51258	46	23	71.3	1160	1	0	0	570.3502
51258	46	23	71.3	1189	1	0	0	573.7982
51258	46	23	71.3	1546	1	0	0	616.2921
51258	46	23	71.3	1704	1	0	0	637.8461
51258	46	23	71.3	1752	1	0	0	644.3341
51258	46	23	71.3	2023	1	1	1	682.8256
51258	46	23	71.3	2050	1	0	0	686.3451
51258	46	23	71.3	2108	1	0	0	692.8545
51258	46	23	71.3	2273	1	0	0	716.3194
51258	46	23	71.3	2324	1	0	0	723.8478
51258	46	23	71.3	2761	1	0	0	811.9293
51258	46	23	71.3	2782	1	0	0	817.4
51258	46	23	71.3	2953	1	0	0	848.9203
51258	46	23	71.3	3289	1	0	0	911.9651
51258	46	23	71.3	3623	1	0	0	976.4474
51258	46	23	71.3	3673	1	0	0	987.0014
51258	46	23	71.3	3943	1	0	0	1051.0485
51258	46	23	71.3	3958	1	0	0	704.3593
51258	46	23	71.3	5208	1	0	0	917.7797
51258	46	23	71.3	5348	1	0	0	938.7834
51258	46	23	71.3	6105	1	0	0	1133.839
51258	46	23	71.3	6636	1	0	0	1525.377
50191	36	21	68	374	1	1	0	455.2551
50191	36	21	68	608	1	1	0	494.2771
50191	36	21	68	1741	1	1	0	642.2554
50191	36	21	68	2086	1	1	0	460.9039

50191	36	21	68	2403	1	1	0	737.4354
50191	36	21	68	2710	1	1	0	799.8872
50191	36	21	68	2941	1	1	1	845.9466
50191	36	21	68	3005	1	1	0	858.464
50191	36	21	68	3206	1	1	0	896.9679
50191	36	21	68	3374	1	1	0	929.4445
50191	36	21	68	3459	1	1	0	943.4767
50191	36	21	68	3474	1	1	0	630.3239
50191	36	21	68	3684	1	1	0	989.4444
50191	36	21	68	4478	1	1	0	760.7332
50191	36	21	68	4642	1	1	0	782.6753
50191	36	21	68	4727	1	1	0	1193.1
50191	36	21	68	4741	1	1	0	1198.0992
50191	36	21	68	4875	1	1	0	834.7083
50191	36	21	68	5033	1	1	0	881.0812
50191	36	21	68	5790	1	1	0	1065.2144
50191	36	21	68	5923	1	1	0	1107.9138
50194	36	21	68	374	1	0	0	455.2551
50194	36	21	68	608	1	0	0	494.2771
50194	36	21	68	1741	1	0	0	642.2554
50194	36	21	68	2086	1	0	0	460.9039
50194	36	21	68	2403	1	0	0	737.4354
50194	36	21	68	2710	1	0	0	799.8872
50194	36	21	68	2861	1	1	1	832.4403
50194	36	21	68	3005	1	0	0	858.464
50194	36	21	68	3206	1	0	0	896.9679
50194	36	21	68	3374	1	0	0	929.4445
50194	36	21	68	3459	1	0	0	943.4767
50194	36	21	68	3474	1	0	0	630.3239
50194	36	21	68	3684	1	0	0	989.4444
50194	36	21	68	4478	1	0	0	760.7332
50194	36	21	68	4642	1	0	0	782.6753
50194	36	21	68	4727	1	0	0	1193.1
50194	36	21	68	4741	1	0	0	1198.0992
50194	36	21	68	4875	1	0	0	834.7083
50194	36	21	68	5033	1	0	0	881.0812
50194	36	21	68	5790	1	0	0	1065.2144
50194	36	21	68	5923	1	0	0	1107.9138
47075	30	17	60.9	336	1	1	0	447.7412
47075	30	17	60.9	510	1	1	0	479.7639
47075	30	17	60.9	1077	1	1	0	557.8452
47075	30	17	60.9	1175	1	1	0	571.7899
47075	30	17	60.9	1925	1	1	1	668.8094
47075	30	17	60.9	2171	1	1	0	701.3698
47075	30	17	60.9	2325	1	1	1	724.3792
47075	30	17	60.9	2480	1	1	0	751.9429
47075	30	17	60.9	2652	1	1	1	788.4282
47075	30	17	60.9	2692	1	1	0	794.3545
47075	30	17	60.9	3299	1	1	0	914.4722
47075	30	17	60.9	3556	1	1	0	961.006
47075	30	17	60.9	3792	1	1	0	676.6362
47075	30	17	60.9	3885	1	1	0	1038.0451
47075	30	17	60.9	5119	1	1	0	1350.2018
47075	30	17	60.9	5493	1	1	0	965.8377
47075	30	17	60.9	5734	1	1	1	1569.3091
46960	30	17	60.2	336	1	0	0	447.7412
46960	30	17	60.2	443	1	1	1	468.2632
46960	30	17	60.2	510	1	0	0	479.7639

46960	30	17	60.2	573	1	1	1	488.7824
46960	30	17	60.2	1041	1	1	1	552.8296
46960	30	17	60.2	1077	1	0	0	557.8452
46960	30	17	60.2	1175	1	0	0	571.7899
46960	30	17	60.2	2171	1	0	0	701.3698
46960	30	17	60.2	2480	1	0	0	751.9429
46960	30	17	60.2	2692	1	0	0	794.3545
46960	30	17	60.2	3299	1	0	0	914.4722
46960	30	17	60.2	3556	1	0	0	961.006
46960	30	17	60.2	3792	1	0	0	676.6362
46960	30	17	60.2	3885	1	0	0	1038.0451
46960	30	17	60.2	5119	1	0	0	1350.2018
46960	30	17	60.2	5493	1	0	0	965.8377
46960	30	17	60.2	6306	1	1	1	1292.9231
50250	31	19	58.9	374	1	0	0	455.2551
50250	31	19	58.9	608	1	0	0	494.2771
50250	31	19	58.9	1741	1	0	0	642.2554
50250	31	19	58.9	2086	1	0	0	460.9039
50250	31	19	58.9	2542	1	1	1	766.4564
50250	31	19	58.9	2679	1	1	1	792.8792
50250	31	19	58.9	2945	1	1	1	846.4385
50250	31	19	58.9	3005	1	0	0	858.464
50250	31	19	58.9	3060	1	1	1	867.9657
50250	31	19	58.9	3406	1	1	0	934.9518
50250	31	19	58.9	3459	1	0	0	943.4767
50250	31	19	58.9	4478	1	0	0	760.7332
50250	31	19	58.9	4642	1	0	0	782.6753
50250	31	19	58.9	4727	1	0	0	1193.1
50250	31	19	58.9	4875	1	0	0	834.7083
50250	31	19	58.9	5111	1	1	1	899.0961
50250	31	19	58.9	5790	1	0	0	1065.2144
50250	31	19	58.9	5923	1	0	0	1107.9138
51883	24	14	43.7	1011	1	1	1	548.3262
51883	24	14	43.7	1121	1	1	1	565.3265
51883	24	14	43.7	1238	1	1	1	579.3281
51883	24	14	43.7	2126	1	1	1	695.3387
51883	24	14	43.7	2290	1	1	1	718.3381
51883	24	14	43.7	2918	1	1	1	841.4764
51883	24	14	43.7	3165	1	1	0	590.2665
51883	24	14	43.7	3256	1	1	1	905.9432
51883	24	14	43.7	3262	1	1	1	906.9894
51883	24	14	43.7	3564	1	1	1	962.0271
51883	24	14	43.7	3965	1	1	1	1057.5285
51883	24	14	43.7	4025	1	1	1	1075.0803
51883	24	14	43.7	5656	1	1	1	1018.2059
51883	24	14	43.7	6174	1	1	1	1718.3735
105902	37	29	41.2	144	1	1	1	399.7635
105902	37	29	41.2	559	1	1	1	486.7773
105902	37	29	41.2	800	1	1	0	519.768
105902	37	29	41.2	845	1	1	1	525.7698
105902	37	29	41.2	1087	1	1	0	558.799
105902	37	29	41.2	1242	1	1	0	579.8216
105902	37	29	41.2	1247	1	1	0	580.3006
105902	37	29	41.2	1306	1	1	0	587.2494
105902	37	29	41.2	1451	1	1	0	403.219
105902	37	29	41.2	1667	1	1	1	632.7713
105902	37	29	41.2	1840	1	1	0	657.8717
105902	37	29	41.2	1859	1	1	1	440.5776

105902	37	29	41.2	2099	1	1	0	691.8849
105902	37	29	41.2	2161	1	1	0	699.3723
105902	37	29	41.2	2206	1	1	1	706.328
105902	37	29	41.2	2412	1	1	0	738.8952
105902	37	29	41.2	2461	1	1	0	748.8686
105902	37	29	41.2	2942	1	1	0	846.4088
105902	37	29	41.2	3486	1	1	1	946.5274
105902	37	29	41.2	3709	1	1	0	662.6571
105902	37	29	41.2	3805	1	1	0	1017.0684
105902	37	29	41.2	4338	1	1	1	751.0372
105902	37	29	41.2	4409	1	1	0	1132.5789
105902	37	29	41.2	4745	1	1	0	799.765
105902	37	29	41.2	4864	1	1	1	831.1349
105902	37	29	41.2	4883	1	1	0	836.7988
105902	37	29	41.2	5069	1	1	0	666.8758
105902	37	29	41.2	5898	1	1	1	1103.5394
105902	37	29	41.2	6286	1	1	0	1273.0234
51237	30	19	59.2	664	1	1	1	502.745
51237	30	19	59.2	883	1	0	0	533.2167
51237	30	19	59.2	1160	1	0	0	570.3502
51237	30	19	59.2	1189	1	0	0	573.7982
51237	30	19	59.2	1546	1	0	0	616.2921
51237	30	19	59.2	1704	1	0	0	637.8461
51237	30	19	59.2	1768	1	1	1	647.3255
51237	30	19	59.2	2050	1	0	0	686.3451
51237	30	19	59.2	2108	1	0	0	692.8545
51237	30	19	59.2	2273	1	0	0	716.3194
51237	30	19	59.2	2324	1	0	0	723.8478
51237	30	19	59.2	2761	1	0	0	811.9293
51237	30	19	59.2	2953	1	0	0	848.9203
51237	30	19	59.2	3630	1	1	0	979.0052
51237	30	19	59.2	3909	1	1	0	695.7039
51237	30	19	59.2	5208	1	0	0	917.7797
51237	30	19	59.2	5369	1	0	1	943.4544
51237	30	19	59.2	6105	1	0	0	1133.839
51237	30	19	59.2	6636	1	0	0	1525.377
71712	30	20	44.5	1480	1	1	0	608.3295
71712	30	20	44.5	1533	1	1	0	614.817
71712	30	20	44.5	1544	1	1	1	615.7684
71712	30	20	44.5	1720	1	1	0	639.8182
71712	30	20	44.5	1837	1	1	0	657.3134
71712	30	20	44.5	2000	1	1	0	679.8111
71712	30	20	44.5	2114	1	1	0	693.8697
71712	30	20	44.5	2262	1	1	1	713.8856
71712	30	20	44.5	2399	1	1	0	737.3462
71712	30	20	44.5	2568	1	1	0	770.882
71712	30	20	44.5	2844	1	1	0	830.4508
71712	30	20	44.5	2858	1	1	0	832.4077
71712	30	20	44.5	2885	1	1	0	838.3679
71712	30	20	44.5	2907	1	1	0	840.9203
71712	30	20	44.5	3197	1	1	0	894.4982
71712	30	20	44.5	3659	1	1	0	984.0059
71712	30	20	44.5	4936	1	1	1	1280.6304
71712	30	20	44.5	5052	1	1	0	1329.6369
71712	30	20	44.5	5636	1	1	1	1009.5033
71712	30	20	44.5	5644	1	1	1	1012.5737
50755	29	16	50.1	883	1	0	0	533.2167
50755	29	16	50.1	1160	1	0	0	570.3502

50755	29	16	50.1	1189	1	0	0	573.7982
50755	29	16	50.1	1546	1	0	0	616.2921
50755	29	16	50.1	1704	1	0	0	637.8461
50755	29	16	50.1	2050	1	0	0	686.3451
50755	29	16	50.1	2079	1	1	1	690.824
50755	29	16	50.1	2108	1	0	0	692.8545
50755	29	16	50.1	2761	1	0	0	811.9293
50755	29	16	50.1	2903	1	0	0	840.4018
50755	29	16	50.1	2953	1	0	0	848.9203
50755	29	16	50.1	3630	1	0	0	979.0052
50755	29	16	50.1	3909	1	0	0	695.7039
50755	29	16	50.1	5348	1	0	0	938.7834
50755	29	16	50.1	6105	1	0	0	1133.839
50755	29	16	50.1	6636	1	0	0	1525.377
71559	26	18	38.4	1480	1	0	0	608.3295
71559	26	18	38.4	1533	1	0	0	614.817
71559	26	18	38.4	1720	1	0	0	639.8182
71559	26	18	38.4	1827	1	1	1	654.8557
71559	26	18	38.4	2000	1	0	0	679.8111
71559	26	18	38.4	2114	1	0	0	693.8697
71559	26	18	38.4	2194	1	1	1	704.3848
71559	26	18	38.4	2274	1	1	1	716.3753
71559	26	18	38.4	2399	1	0	0	737.3462
71559	26	18	38.4	2513	1	1	1	761.9034
71559	26	18	38.4	2844	1	0	0	830.4508
71559	26	18	38.4	2885	1	0	0	838.3679
71559	26	18	38.4	2889	1	1	1	839.4162
71559	26	18	38.4	2907	1	0	0	840.9203
71559	26	18	38.4	3197	1	0	0	894.4982
71559	26	18	38.4	3963	1	1	1	704.7043
71559	26	18	38.4	4957	1	0	0	1287.6368
71559	26	18	38.4	5052	1	0	0	1329.6369
136636	25	18	20	231	1	1	1	424.7529
136636	25	18	20	492	1	1	1	476.2479
136636	25	18	20	558	1	1	1	486.7717
136636	25	18	20	612	1	1	1	494.7832
136636	25	18	20	1216	1	1	1	576.8042
136636	25	18	20	1565	1	1	1	618.3062
136636	25	18	20	1691	1	1	1	635.3615
136636	25	18	20	1709	1	1	1	638.8283
136636	25	18	20	2189	1	1	1	703.9085
136636	25	18	20	2383	1	1	1	734.8951
136636	25	18	20	2431	1	1	1	742.3843
136636	25	18	20	2631	1	1	1	784.9184
136636	25	18	20	3580	1	1	1	644.3626
136636	25	18	20	3651	1	1	1	983.0145
136636	25	18	20	3939	1	1	1	700.6888
136636	25	18	20	4302	1	1	1	1121.1202
136636	25	18	20	4873	1	1	1	1251.1752
136636	25	18	20	6639	1	1	1	1147.3181
103616	28	22	32	113	1	1	1	392.7559
103616	28	22	32	800	1	0	0	519.768
103616	28	22	32	1087	1	0	0	558.799
103616	28	22	32	1242	1	0	0	579.8216
103616	28	22	32	1247	1	0	0	580.3006
103616	28	22	32	1306	1	0	0	587.2494
103616	28	22	32	1451	1	0	0	403.219
103616	28	22	32	1840	1	0	0	657.8717

103616	28	22	32	2099	1	0	0	691.8849
103616	28	22	32	2161	1	0	0	699.3723
103616	28	22	32	2259	1	1	1	713.8331
103616	28	22	32	2412	1	0	0	738.8952
103616	28	22	32	2461	1	0	0	748.8686
103616	28	22	32	2942	1	0	0	846.4088
103616	28	22	32	3709	1	0	0	662.6571
103616	28	22	32	3805	1	0	0	1017.0684
103616	28	22	32	4409	1	0	0	1132.5789
103616	28	22	32	4745	1	0	0	799.765
103616	28	22	32	4883	1	0	0	836.7988
103616	28	22	32	5069	1	0	0	666.8758
103616	28	22	32	6286	1	0	0	1273.0234
98766	19	15	22.4	737	1	1	1	511.2713
98766	19	15	22.4	1228	1	1	1	578.7742
98766	19	15	22.4	1267	1	1	1	582.306
98766	19	15	22.4	1288	1	1	1	584.3029
98766	19	15	22.4	1679	1	1	1	634.308
98766	19	15	22.4	1926	1	1	1	668.8405
98766	19	15	22.4	2183	1	1	1	702.4367
98766	19	15	22.4	2426	1	1	1	740.9148
98766	19	15	22.4	2624	1	1	1	783.4582
98766	19	15	22.4	3733	1	1	1	1000.0324
98766	19	15	22.4	3845	1	1	1	1027.5267
98766	19	15	22.4	3962	1	1	1	1056.5297
98766	19	15	22.4	4921	1	1	1	847.1077
98766	19	15	22.4	5056	1	1	1	887.7675
98766	19	15	22.4	5065	1	1	1	888.4987
49813	24	11	34.7	392	1	1	1	457.787
49813	24	11	34.7	498	1	1	1	477.2684
49813	24	11	34.7	748	1	1	1	513.3082
49813	24	11	34.7	1098	1	1	1	560.8029
49813	24	11	34.7	1786	1	1	1	649.8769
49813	24	11	34.7	2333	1	1	1	726.3401
49813	24	11	34.7	2685	1	1	1	793.9191
49813	24	11	34.7	2788	1	1	1	545.6379
49813	24	11	34.7	4546	1	1	1	1148.57
49813	24	11	34.7	4907	1	1	1	1261.6651
49813	24	11	34.7	5598	1	1	1	991.8135
71741	18	13	28.9	1401	1	1	1	599.3508
71741	18	13	28.9	1533	1	0	0	614.817
71741	18	13	28.9	1720	1	0	0	639.8182
71741	18	13	28.9	2000	1	0	0	679.8111
71741	18	13	28.9	2399	1	0	0	737.3462
71741	18	13	28.9	2844	1	0	0	830.4508
71741	18	13	28.9	2858	1	0	0	832.4077
71741	18	13	28.9	2885	1	0	0	838.3679
71741	18	13	28.9	2907	1	0	0	840.9203
71741	18	13	28.9	3197	1	0	0	894.4982
71741	18	13	28.9	3659	1	0	0	984.0059
71741	18	13	28.9	4957	1	0	0	1287.6368
71741	18	13	28.9	5753	1	1	1	1050.8396
41112	15	11	31.3	130	1	1	1	397.2265
41112	15	11	31.3	177	1	1	0	408.7505
41112	15	11	31.3	473	1	1	0	472.7979
41112	15	11	31.3	1311	1	1	0	587.8103
41112	15	11	31.3	1505	1	1	0	611.8184
41112	15	11	31.3	2421	1	1	0	740.3936

41112	15	11	31.3	2499	1	1	1	758.3617
41112	15	11	31.3	2919	1	1	1	841.9204
41112	15	11	31.3	3305	1	1	1	915.5025
41112	15	11	31.3	3342	1	1	1	923.9312
41112	15	11	31.3	4049	1	1	0	1079.0426
71456	21	16	31.1	1533	1	0	0	614.817
71456	21	16	31.1	1550	1	1	1	616.3272
71456	21	16	31.1	1720	1	0	0	639.8182
71456	21	16	31.1	1837	1	0	0	657.3134
71456	21	16	31.1	2000	1	0	0	679.8111
71456	21	16	31.1	2213	1	1	1	706.8777
71456	21	16	31.1	2399	1	0	0	737.3462
71456	21	16	31.1	2568	1	0	0	770.882
71456	21	16	31.1	2668	1	1	1	791.4133
71456	21	16	31.1	2844	1	0	0	830.4508
71456	21	16	31.1	2872	1	1	1	833.3977
71456	21	16	31.1	2885	1	0	0	838.3679
71456	21	16	31.1	2907	1	0	0	840.9203
71456	21	16	31.1	3197	1	0	0	894.4982
71456	21	16	31.1	3659	1	0	0	984.0059
71456	21	16	31.1	4957	1	0	0	1287.6368
615675	18	16	4.2	396	1	1	1	458.2972
615675	18	16	4.2	505	1	1	1	478.3005
615675	18	16	4.2	788	1	1	1	518.2952
615675	18	16	4.2	1071	1	1	1	557.2822
615675	18	16	4.2	2088	1	1	1	690.885
615675	18	16	4.2	2233	1	1	1	710.8933
615675	18	16	4.2	2418	1	1	1	739.8429
615675	18	16	4.2	2477	1	1	1	751.8655
615675	18	16	4.2	2571	1	1	1	771.4274
615675	18	16	4.2	2754	1	1	1	810.4538
615675	18	16	4.2	3462	1	1	1	943.5529
615675	18	16	4.2	3616	1	1	1	973.4983
615675	18	16	4.2	3867	1	1	1	689.702
615675	18	16	4.2	3924	1	1	1	1047.0509
615675	18	16	4.2	4147	1	1	1	1100.0366
615675	18	16	4.2	4911	1	1	1	843.0533
286993	12	10	5.9	1442	1	1	1	603.4152
286993	12	10	5.9	1560	1	1	1	617.8085
286993	12	10	5.9	2018	1	1	1	681.8767
286993	12	10	5.9	2063	1	1	1	687.8867
286993	12	10	5.9	3132	1	1	1	881.4819
286993	12	10	5.9	3830	1	1	1	1025.0564
286993	12	10	5.9	4133	1	1	1	1097.6198
286993	12	10	5.9	4297	1	1	1	747.397
286993	12	10	5.9	5172	1	1	1	912.8492
286993	12	10	5.9	5746	1	1	1	1047.9252
44960	15	12	43.9	1244	1	1	0	579.8293
44960	15	12	43.9	1262	1	1	1	581.797
44960	15	12	43.9	1700	1	1	0	637.326
44960	15	12	43.9	1711	1	1	0	638.8484
44960	15	12	43.9	2093	1	1	0	691.8317
44960	15	12	43.9	2136	1	1	0	696.8832
44960	15	12	43.9	2994	1	1	0	569.9804
44960	15	12	43.9	3231	1	1	0	900.9802
44960	15	12	43.9	3587	1	1	1	969.4852
44960	15	12	43.9	4236	1	1	0	742.0756
44960	15	12	43.9	5848	1	1	0	1082.1898

52347	12	8	24.7	1219	1	1	1	576.8604
52347	12	8	24.7	1531	1	1	1	614.7881
52347	12	8	24.7	2955	1	1	1	849.3846
52347	12	8	24.7	3044	1	1	1	864.8778
52347	12	8	24.7	3187	1	1	1	890.9081
52347	12	8	24.7	4032	1	1	1	1075.5693
52347	12	8	24.7	4130	1	1	1	731.7769
52347	12	8	24.7	4645	1	1	1	1174.0968
125058	16	13	20.9	226	1	1	1	422.7569
125058	16	13	20.9	783	1	1	1	518.2483
125058	16	13	20.9	876	1	1	1	532.3026
125058	16	13	20.9	1280	1	1	1	583.8061
125058	16	13	20.9	1569	1	1	1	618.8747
125058	16	13	20.9	2995	1	1	1	855.402
125058	16	13	20.9	3045	1	1	1	864.9397
125058	16	13	20.9	4715	1	1	1	1190.5691
125058	16	13	20.9	4877	1	1	1	1252.6306
125058	16	13	20.9	5275	1	1	1	696.8507
125058	16	13	20.9	5477	1	1	1	962.1509
125058	16	13	20.9	5798	1	1	1	1066.5445
125058	16	13	20.9	5914	1	1	1	1106.559
47236	12	9	32.9	286	1	1	0	433.7641
47236	12	9	32.9	1244	1	0	0	579.8293
47236	12	9	32.9	1283	1	1	0	584.2729
47236	12	9	32.9	1397	1	1	0	598.2826
47236	12	9	32.9	1776	1	1	0	648.3242
47236	12	9	32.9	2816	1	1	0	823.4491
47236	12	9	32.9	3663	1	1	1	984.4724
47236	12	9	32.9	4381	1	1	0	753.3647
47236	12	9	32.9	6295	1	1	1	1278.272
73315	13	11	24.6	1615	1	1	1	624.8451
73315	13	11	24.6	1616	1	1	1	625.2861
73315	13	11	24.6	1888	1	1	1	664.3221
73315	13	11	24.6	2013	1	1	1	681.3758
73315	13	11	24.6	2224	1	1	0	709.3708
73315	13	11	24.6	2415	1	1	1	739.3858
73315	13	11	24.6	3265	1	1	1	907.4804
73315	13	11	24.6	3597	1	1	1	970.5071
73315	13	11	24.6	3882	1	1	1	691.0355
73315	13	11	24.6	5367	1	1	1	942.5476
73315	13	11	24.6	5686	1	1	1	1026.8279
40949	13	11	36.6	177	1	0	0	408.7505
40949	13	11	36.6	473	1	0	0	472.7979
40949	13	11	36.6	1311	1	0	0	587.8103
40949	13	11	36.6	1505	1	0	0	611.8184
40949	13	11	36.6	2421	1	0	0	740.3936
40949	13	11	36.6	2499	1	0	1	758.3617
40949	13	11	36.6	3155	1	1	1	883.4594
40949	13	11	36.6	3230	1	1	1	900.9727
40949	13	11	36.6	3290	1	1	1	911.9858
40949	13	11	36.6	4049	1	0	0	1079.0426
40949	13	11	36.6	5168	1	1	1	912.7767
103283	13	10	12.9	733	1	1	1	510.2602
103283	13	10	12.9	1365	1	1	1	594.3221
103283	13	10	12.9	1717	1	1	1	639.3586
103283	13	10	12.9	1742	1	1	0	642.3153
103283	13	10	12.9	2253	1	1	1	712.8854
103283	13	10	12.9	2311	1	1	1	481.6072

103283	13	10	12.9	2313	1	1	1	722.3955
103283	13	10	12.9	2372	1	1	1	731.8881
103283	13	10	12.9	2812	1	1	1	822.9749
103283	13	10	12.9	4856	1	1	1	829.0775
65547	18	12	29.5	517	1	0	1	481.2137
65547	18	12	29.5	912	1	1	0	536.7767
65547	18	12	29.5	1368	1	1	0	594.3549
65547	18	12	29.5	1402	1	1	1	599.7791
65547	18	12	29.5	2380	1	1	1	733.9162
65547	18	12	29.5	2471	1	1	1	750.8465
65547	18	12	29.5	2572	1	1	0	514.6604
65547	18	12	29.5	3276	1	1	1	908.459
65547	18	12	29.5	4313	1	1	1	748.7341
65547	18	12	29.5	4769	1	1	0	1205.5708
65547	18	12	29.5	4974	1	1	1	862.1202
65547	18	12	29.5	5523	1	1	1	969.1654
37004	13	10	38.8	203	1	1	1	417.2369
37004	13	10	38.8	1165	1	1	1	570.8254
37004	13	10	38.8	1201	1	1	1	383.5608
37004	13	10	38.8	2284	1	1	1	717.8813
37004	13	10	38.8	2465	1	1	1	749.9271
37004	13	10	38.8	2888	1	1	1	559.6625
37004	13	10	38.8	3128	1	1	1	881.3964
37004	13	10	38.8	3802	1	1	1	1017.0404
37004	13	10	38.8	4083	1	1	1	1086.5077
37004	13	10	38.8	5024	1	1	1	877.4703
73174	12	9	18.6	1122	1	1	1	565.7881
73174	12	9	18.6	1378	1	1	1	595.8424
73174	12	9	18.6	1564	1	1	1	618.2909
73174	12	9	18.6	2223	1	1	1	709.3676
73174	12	9	18.6	2224	1	0	0	709.3708
73174	12	9	18.6	3285	1	1	1	911.4576
73174	12	9	18.6	3409	1	1	1	624.0041
73174	12	9	18.6	3597	1	0	1	970.5071
73174	12	9	18.6	4823	1	1	1	1228.7043
58863	13	11	34.8	504	1	1	1	478.2897
58863	13	11	34.8	998	1	1	1	546.303
58863	13	11	34.8	1854	1	1	1	659.3651
58863	13	11	34.8	2735	1	1	1	805.4426
58863	13	11	34.8	2835	1	1	1	826.4127
58863	13	11	34.8	2971	1	1	1	851.8725
58863	13	11	34.8	3670	1	1	1	657.6892
58863	13	11	34.8	4140	1	1	1	1099.0572
58863	13	11	34.8	4640	1	1	1	781.743
58863	13	11	34.8	5054	1	1	1	886.7869
58863	13	11	34.8	5773	1	1	1	1053.2116
94743	10	9	19.2	54	1	1	1	372.6948
94743	10	9	19.2	2341	1	1	1	726.8483
94743	10	9	19.2	2476	1	1	1	751.8423
94743	10	9	19.2	3228	1	1	1	900.4691
94743	10	9	19.2	3244	1	1	1	603.3137
94743	10	9	19.2	4377	1	1	1	1129.1051
94743	10	9	19.2	4748	1	1	1	800.7265
94743	10	9	19.2	5551	1	1	1	978.1487
94743	10	9	19.2	5828	1	1	1	806.4122
83408	10	8	14.5	812	1	1	1	521.7514
83408	10	8	14.5	1501	1	1	1	610.8259
83408	10	8	14.5	1635	1	1	1	628.8296

83408	10	8	14.5	1883	1	1	1	442.5651
83408	10	8	14.5	2393	1	1	1	735.9001
83408	10	8	14.5	2829	1	1	1	825.4001
83408	10	8	14.5	2873	1	1	1	833.4271
83408	10	8	14.5	4785	1	1	1	1208.6133
65220	8	6	18.1	1966	1	1	1	672.8779
65220	8	6	18.1	2801	1	1	1	821.4178
65220	8	6	18.1	3049	1	1	1	865.9204
65220	8	6	18.1	3608	1	1	1	648.6491
65220	8	6	18.1	4031	1	1	1	1075.5549
65220	8	6	18.1	5093	1	1	1	894.1208
49487	10	9	29.8	561	1	1	1	486.7905
49487	10	9	29.8	963	1	1	1	542.3002
49487	10	9	29.8	1572	1	1	1	619.3299
49487	10	9	29.8	1754	1	1	1	644.358
49487	10	9	29.8	2297	1	1	1	479.612
49487	10	9	29.8	3107	1	1	1	876.4351
49487	10	9	29.8	4015	1	1	1	1073.0583
49487	10	9	29.8	4053	1	1	1	1079.5767
49487	10	9	29.8	4163	1	1	1	1103.0719
62205	9	9	20.5	1435	1	1	1	602.8147
62205	9	9	20.5	1758	1	1	1	645.812
62205	9	9	20.5	2420	1	1	1	740.3764
62205	9	9	20.5	2833	1	1	1	825.8995
62205	9	9	20.5	3112	1	1	1	877.4673
62205	9	9	20.5	4003	1	1	1	713.7241
62205	9	9	20.5	4337	1	1	1	1125.6511
62205	9	9	20.5	4901	1	1	1	840.4981
62205	9	9	20.5	5412	1	1	1	950.5503
41937	11	8	32.4	570	1	1	0	488.7278
41937	11	8	32.4	1182	1	1	0	572.784
41937	11	8	32.4	1316	1	1	0	588.7714
41937	11	8	32.4	1403	1	1	0	599.856
41937	11	8	32.4	3178	1	1	0	887.9509
41937	11	8	32.4	3366	1	1	1	928.4683
41937	11	8	32.4	3625	1	1	0	977.5359
41937	11	8	32.4	5757	1	1	0	1051.2178
40001	13	8	27.5	1001	1	1	1	547.2396
40001	13	8	27.5	1395	1	1	0	597.3347
40001	13	8	27.5	1485	1	1	0	609.3057
40001	13	8	27.5	1730	1	1	1	640.8188
40001	13	8	27.5	2245	1	1	0	712.3356
40001	13	8	27.5	2432	1	1	1	742.4143
40001	13	8	27.5	2721	1	1	1	801.9387
40001	13	8	27.5	3803	1	1	1	678.3628
36185	14	9	34.2	462	1	1	1	472.258
36185	14	9	34.2	1744	1	1	1	642.3817
36185	14	9	34.2	1805	1	1	1	652.379
36185	14	9	34.2	1829	1	1	1	655.8137
36185	14	9	34.2	2080	1	1	1	690.8265
36185	14	9	34.2	2456	1	1	1	747.8804
36185	14	9	34.2	2497	1	1	1	505.2839
36185	14	9	34.2	2558	1	1	1	768.48
36185	14	9	34.2	3111	1	1	1	877.4322
63798	14	11	25.2	912	1	0	0	536.7767
63798	14	11	25.2	1352	1	1	1	592.7712
63798	14	11	25.2	1368	1	0	0	594.3549
63798	14	11	25.2	1702	1	1	1	637.8404

63798	14	11	25.2	2434	1	1	1	742.8485
63798	14	11	25.2	2562	1	1	1	513.2862
63798	14	11	25.2	2572	1	0	0	514.6604
63798	14	11	25.2	3217	1	1	1	898.8765
63798	14	11	25.2	3238	1	1	1	902.4322
63798	14	11	25.2	4132	1	1	1	732.0586
63798	14	11	25.2	4769	1	0	0	1205.5708
34389	9	7	38	214	1	1	1	421.2661
34389	9	7	38	1879	1	1	1	662.8953
34389	9	7	38	2056	1	1	1	687.3249
34389	9	7	38	2714	1	1	1	800.4112
34389	9	7	38	2854	1	1	1	831.9213
34389	9	7	38	4811	1	1	1	815.0617
34389	9	7	38	5886	1	1	1	1101.2362
10285	10	5	55.7	409	1	1	1	460.7471
10285	10	5	55.7	459	1	1	1	471.258
10285	10	5	55.7	840	1	1	1	524.7945
10285	10	5	55.7	2303	1	1	1	720.3466
10285	10	5	55.7	4046	1	1	1	1078.5155
154652	7	6	6.1	1258	1	1	1	581.3085
154652	7	6	6.1	1858	1	1	1	659.8838
154652	7	6	6.1	2327	1	1	1	724.4108
154652	7	6	6.1	2633	1	1	1	785.9124
154652	7	6	6.1	3480	1	1	1	946
154652	7	6	6.1	3777	1	1	1	672.6873
47389	9	5	17.4	1244	1	0	0	579.8293
47389	9	5	17.4	1880	1	1	1	663.3205
47389	9	5	17.4	2424	1	1	1	493.9596
47389	9	5	17.4	3561	1	1	1	961.9797
47389	9	5	17.4	3913	1	1	1	1044.5048
148890	6	4	5.1	2410	1	1	1	738.8904
148890	6	4	5.1	2529	1	1	1	763.4566
148890	6	4	5.1	3599	1	1	1	971.0322
148890	6	4	5.1	4834	1	1	1	823.0873
196643	11	10	9.4	855	1	1	1	528.2709
196643	11	10	9.4	1030	1	1	1	551.804
196643	11	10	9.4	1733	1	1	1	640.9005
196643	11	10	9.4	1853	1	1	1	659.3632
196643	11	10	9.4	2984	1	1	1	853.4331
196643	11	10	9.4	3993	1	1	1	1066.029
196643	11	10	9.4	4156	1	1	1	734.7327
196643	11	10	9.4	4692	1	1	1	789.3576
196643	11	10	9.4	4778	1	1	1	1206.604
196643	11	10	9.4	4890	1	1	1	837.7917
41563	13	9	23.4	88	1	1	1	384.2007
41563	13	9	23.4	405	1	1	0	459.2943
41563	13	9	23.4	1449	1	1	1	604.3195
41563	13	9	23.4	1464	1	1	1	606.3064
41563	13	9	23.4	2011	1	1	1	680.86
41563	13	9	23.4	2196	1	1	1	470.2454
41563	13	9	23.4	3799	1	1	1	1016.5135
41563	13	9	23.4	3953	1	1	1	702.7366
55711	6	6	22.1	694	1	1	1	505.7484
55711	6	6	22.1	1848	1	1	1	658.838
55711	6	6	22.1	2613	1	1	1	780.9082
55711	6	6	22.1	3971	1	1	1	1058.5106
55711	6	6	22.1	4709	1	1	1	1188.5605
55711	6	6	22.1	6095	1	1	1	1132.8888

29784	4	4	23.7	3585	1	1	1	968.02
29784	4	4	23.7	3921	1	1	1	697.7162
29784	4	4	23.7	4803	1	1	1	1216.1238
29784	4	4	23.7	4855	1	1	1	1242.6158
49607	7	5	15.9	1471	1	1	1	607.3428
49607	7	5	15.9	2694	1	1	1	794.8992
49607	7	5	15.9	2783	1	1	1	817.4044
49607	7	5	15.9	2876	1	1	1	834.4574
49607	7	5	15.9	3165	1	0	0	590.2665
73869	8	7	12.7	1226	1	1	1	578.3274
73869	8	7	12.7	1967	1	1	1	673.3259
73869	8	7	12.7	2455	1	1	1	747.8643
73869	8	7	12.7	2560	1	1	1	512.9214
73869	8	7	12.7	2844	1	0	0	830.4508
73869	8	7	12.7	3197	1	0	0	894.4982
73869	8	7	12.7	3840	1	1	0	1027.001
59440	10	10	27.9	380	1	1	1	456.2529
59440	10	10	27.9	1567	1	1	0	618.8135
59440	10	10	27.9	1769	1	1	1	647.343
59440	10	10	27.9	2045	1	1	0	685.3194
59440	10	10	27.9	2654	1	1	1	789.4288
59440	10	10	27.9	2745	1	1	0	807.96
59440	10	10	27.9	2878	1	1	0	835.4185
59440	10	10	27.9	3764	1	1	1	671.7334
59440	10	10	27.9	4927	1	1	1	637.3549
59440	10	10	27.9	6471	1	1	1	1036.5456
43168	9	9	35.9	86	1	1	0	383.2172
43168	9	9	35.9	310	1	1	1	437.2612
43168	9	9	35.9	582	1	1	0	490.2406
43168	9	9	35.9	1447	1	1	1	603.8086
43168	9	9	35.9	2346	1	1	0	727.3804
43168	9	9	35.9	2392	1	1	1	735.8709
43168	9	9	35.9	3254	1	1	1	905.4188
43168	9	9	35.9	4718	1	1	1	795.0948
58865	9	8	22.6	521	1	1	1	481.2715
58865	9	8	22.6	871	1	1	1	530.8057
58865	9	8	22.6	1492	1	1	1	609.8149
58865	9	8	22.6	1576	1	1	1	620.8195
58865	9	8	22.6	2179	1	1	1	702.3617
58865	9	8	22.6	3016	1	1	1	859.4503
58865	9	8	22.6	3577	1	1	1	965.0003
58865	9	8	22.6	6247	1	1	1	1206.6016
59235	8	7	20.3	1567	1	0	0	618.8135
59235	8	7	20.3	2045	1	0	0	685.3194
59235	8	7	20.3	2589	1	1	1	775.433
59235	8	7	20.3	2745	1	0	0	807.96
59235	8	7	20.3	2878	1	0	0	835.4185
59235	8	7	20.3	3756	1	1	1	669.7417
59235	8	7	20.3	5000	1	1	1	651.8488
86221	7	6	9.9	71	1	1	1	378.224
86221	7	6	9.9	756	1	1	1	513.8006
86221	7	6	9.9	1620	1	1	1	625.8222
86221	7	6	9.9	1908	1	1	1	665.8824
86221	7	6	9.9	3332	1	1	1	614.6477
86221	7	6	9.9	4582	1	1	1	1154.5838
65730	7	6	14.4	1029	1	1	1	551.7987
65730	7	6	14.4	1260	1	1	1	581.7688
65730	7	6	14.4	2184	1	1	1	702.8333

65730	7	6	14.4	2212	1	1	1	706.8775
65730	7	6	14.4	3815	1	1	1	1019.9765
65730	7	6	14.4	4235	1	1	1	742.0655
42056	8	6	23.3	570	1	0	0	488.7278
42056	8	6	23.3	1130	1	1	0	566.7688
42056	8	6	23.3	1182	1	0	0	572.784
42056	8	6	23.3	1403	1	0	0	599.856
42056	8	6	23.3	3202	1	1	1	895.9476
42056	8	6	23.3	5757	1	0	0	1051.2178
48286	6	5	12.4	62	1	1	1	374.721
48286	6	5	12.4	322	1	1	1	441.7115
48286	6	5	12.4	1992	1	1	1	679.3157
48286	6	5	12.4	2191	1	1	1	704.3104
48286	6	5	12.4	3065	1	1	1	869.4277
137407	8	8	9.9	785	1	1	0	518.2811
137407	8	8	9.9	1436	1	1	0	603.2805
137407	8	8	9.9	2053	1	1	0	686.4033
137407	8	8	9.9	2396	1	1	1	736.908
137407	8	8	9.9	2578	1	1	1	772.8989
137407	8	8	9.9	2751	1	1	1	809.9276
137407	8	8	9.9	3227	1	1	0	900.4148
137407	8	8	9.9	5250	1	1	1	694.627
100826	10	9	16.2	1056	1	1	1	555.2738
100826	10	9	16.2	1198	1	1	1	574.8013
100826	10	9	16.2	1593	1	1	1	622.3533
100826	10	9	16.2	1727	1	1	1	640.3267
100826	10	9	16.2	2576	1	1	1	515.284
100826	10	9	16.2	3103	1	1	1	583.9995
100826	10	9	16.2	3820	1	1	1	1023.0549
100826	10	9	16.2	5508	1	1	1	968.1759
100826	10	9	16.2	5709	1	1	1	779.1663
120769	8	7	9	1555	1	1	1	617.3165
120769	8	7	9	1715	1	1	1	639.3391
120769	8	7	9	1736	1	1	1	641.3157
120769	8	7	9	2026	1	1	1	682.8436
120769	8	7	9	2209	1	1	1	706.3739
120769	8	7	9	2607	1	1	1	778.438
120769	8	7	9	4862	1	1	1	830.4461
64169	6	6	14.5	819	1	1	0	522.2954
64169	6	6	14.5	1345	1	1	0	591.8584
64169	6	6	14.5	1410	1	1	1	600.3347
64169	6	6	14.5	1732	1	1	0	640.8877
64169	6	6	14.5	2964	1	1	0	850.953
64169	6	6	14.5	4949	1	1	1	857.1423
39964	6	6	24	1395	1	0	0	597.3347
39964	6	6	24	1485	1	0	0	609.3057
39964	6	6	24	2245	1	0	0	712.3356
39964	6	6	24	2389	1	1	1	735.4063
39964	6	6	24	4281	1	1	1	1118.5755
39964	6	6	24	4406	1	1	1	1131.0428
44356	7	5	21.7	627	1	1	1	496.7491
44356	7	5	21.7	866	1	1	1	530.2747
44356	7	5	21.7	3582	1	1	1	967.4706
44356	7	5	21.7	4153	1	1	1	1101.5518
44356	7	5	21.7	6011	1	1	1	1119.867
66087	6	4	7.7	994	1	1	1	545.7729
66087	6	4	7.7	2574	1	1	1	772.3866
66087	6	4	7.7	2923	1	1	1	842.4802

66087	6	4	7.7	4603	1	1	1	775.1005
194433	8	7	6.5	2035	1	1	0	683.8752
194433	8	7	6.5	2193	1	1	1	704.3596
194433	8	7	6.5	2295	1	1	0	718.3746
194433	8	7	6.5	2411	1	1	0	738.8933
194433	8	7	6.5	3787	1	1	0	675.6643
194433	8	7	6.5	3852	1	1	1	1029.54
194433	8	7	6.5	5060	1	1	0	888.1523
59765	10	8	22.1	569	1	1	1	488.2846
59765	10	8	22.1	1721	1	1	1	639.821
59765	10	8	22.1	2141	1	1	1	697.4114
59765	10	8	22.1	2257	1	1	1	713.3907
59765	10	8	22.1	2355	1	1	1	729.4236
59765	10	8	22.1	2448	1	1	1	746.8909
59765	10	8	22.1	3394	1	1	1	932.9753
59765	10	8	22.1	5755	1	1	1	1050.9179
17591	4	2	19.1	743	1	1	1	512.2987
17591	4	2	19.1	3908	1	1	1	1043.0374
59426	5	4	13.5	1462	1	1	1	605.8432
59426	5	4	13.5	3074	1	1	1	870.5068
59426	5	4	13.5	4464	1	1	1	1139.1612
59426	5	4	13.5	5012	1	1	1	874.1385
42064	7	5	22.3	570	1	0	0	488.7278
42064	7	5	22.3	1316	1	0	0	588.7714
42064	7	5	22.3	3178	1	0	0	887.9509
42064	7	5	22.3	3665	1	1	1	656.6983
42064	7	5	22.3	5759	1	0	1	1051.2183
23090	6	5	23.7	648	1	1	0	499.7845
23090	6	5	23.7	1394	1	1	0	398.544
23090	6	5	23.7	2182	1	1	1	702.404
23090	6	5	23.7	2612	1	1	1	520.6386
23090	6	5	23.7	2776	1	1	0	816.419
194402	7	6	5.8	2035	1	0	0	683.8752
194402	7	6	5.8	2295	1	0	0	718.3746
194402	7	6	5.8	2411	1	0	0	738.8933
194402	7	6	5.8	3787	1	0	0	675.6643
194402	7	6	5.8	3816	1	1	1	1020.5621
194402	7	6	5.8	5060	1	0	0	888.1523
52003	5	3	11.6	2884	1	1	1	837.9535
52003	5	3	11.6	3283	1	1	1	607.6081
52003	5	3	11.6	4854	1	1	1	1241.6743
63627	5	5	10.6	819	1	0	0	522.2954
63627	5	5	10.6	1345	1	0	0	591.8584
63627	5	5	10.6	1541	1	1	1	615.3529
63627	5	5	10.6	1732	1	0	0	640.8877
63627	5	5	10.6	2964	1	0	0	850.953
104379	6	5	6.5	606	1	1	1	493.7836
104379	6	5	6.5	726	1	1	1	509.3078
104379	6	5	6.5	1342	1	1	1	591.8143
104379	6	5	6.5	1921	1	1	1	668.3269
104379	6	5	6.5	3562	1	1	1	961.9813
41834	11	8	23.9	405	1	0	0	459.2943
41834	11	8	23.9	493	1	1	1	476.2481
41834	11	8	23.9	1532	1	1	1	614.8139
41834	11	8	23.9	2011	1	0	1	680.86
41834	11	8	23.9	2177	1	1	1	468.2528
41834	11	8	23.9	3831	1	1	1	1025.4918
41834	11	8	23.9	3922	1	1	1	697.7398

153955	5	5	6.3	1778	1	1	1	648.4238
153955	5	5	6.3	3607	1	1	1	648.3701
153955	5	5	6.3	3796	1	1	1	1015.0708
153955	5	5	6.3	3874	1	1	1	690.051
153955	5	5	6.3	3883	1	1	1	1037.5673
146599	10	9	9.1	660	1	1	1	502.2514
146599	10	9	9.1	954	1	1	1	541.3217
146599	10	9	9.1	1061	1	1	1	555.7564
146599	10	9	9.1	1882	1	1	0	663.3387
146599	10	9	9.1	1971	1	1	1	674.3398
146599	10	9	9.1	2150	1	1	1	698.3529
146599	10	9	9.1	2799	1	1	1	821.3821
146599	10	9	9.1	3208	1	1	0	897.5034
146599	10	9	9.1	5098	1	1	1	896.8286
94470	5	5	8.5	1322	1	1	0	589.3142
94470	5	5	8.5	2436	1	1	1	742.9581
94470	5	5	8.5	2762	1	1	1	812.4234
94470	5	5	8.5	3683	1	1	1	988.9768
94470	5	5	8.5	3774	1	1	0	672.3501
114752	7	6	9.2	82	1	1	1	380.2398
114752	7	6	9.2	1036	1	1	1	552.3058
114752	7	6	9.2	2596	1	1	1	776.4001
114752	7	6	9.2	3937	1	1	1	700.0427
114752	7	6	9.2	4063	1	1	1	1082.0625
114752	7	6	9.2	4962	1	1	1	1290.657
44974	4	3	11.4	2077	1	1	1	690.3608
44974	4	3	11.4	2580	1	1	1	773.898
44974	4	3	11.4	4051	1	1	1	1079.4988
81293	9	9	17.7	403	1	1	1	458.7844
81293	9	9	17.7	422	1	1	1	464.7769
81293	9	9	17.7	526	1	1	1	481.7688
81293	9	9	17.7	857	1	1	1	528.2959
81293	9	9	17.7	1641	1	1	1	629.327
81293	9	9	17.7	1929	1	1	1	668.8898
81293	9	9	17.7	2537	1	1	1	765.4142
81293	9	9	17.7	4044	1	1	1	718.7029
81293	9	9	17.7	6335	1	1	1	1013.4987
47005	5	4	13.4	490	1	1	1	475.7688
47005	5	4	13.4	2360	1	1	1	730.3914
47005	5	4	13.4	3636	1	1	1	981.4902
47005	5	4	13.4	3935	1	1	1	524.7987
59860	4	4	11.8	858	1	1	1	528.8062
59860	4	4	11.8	1112	1	1	1	563.7633
59860	4	4	11.8	3078	1	1	1	870.9857
59860	4	4	11.8	5136	1	1	1	903.7724
72129	4	3	7.3	2730	1	1	1	803.4274
72129	4	3	7.3	3172	1	1	1	886.0194
72129	4	3	7.3	3567	1	1	1	963.0166
45894	6	6	18.6	1244	1	0	0	579.8293
45894	6	6	18.6	1692	1	1	1	636.3461
45894	6	6	18.6	2234	1	1	1	474.2804
45894	6	6	18.6	3795	1	1	1	1015.0336
45894	6	6	18.6	4009	1	1	1	714.6788
45894	6	6	18.6	4630	1	1	1	781.0943
60195	4	3	11.3	1580	1	1	1	621.2915
60195	4	3	11.3	4477	1	1	1	1140.5836
60195	4	3	11.3	5732	1	1	1	1045.8563
100452	5	4	5.9	918	1	1	1	537.2875

100452	5	4	5.9	1241	1	1	1	579.8095
100452	5	4	5.9	1789	1	1	1	650.385
100452	5	4	5.9	4162	1	1	1	735.4006
90209	6	6	11.5	1346	1	1	1	592.3035
90209	6	6	11.5	1400	1	1	1	599.2985
90209	6	6	11.5	1831	1	1	1	437.5533
90209	6	6	11.5	4168	1	1	1	1104.0207
90209	6	6	11.5	4612	1	1	1	776.4031
90209	6	6	11.5	4761	1	1	1	1204.1296
116657	5	4	6.4	1591	1	1	1	622.3354
116657	5	4	6.4	2102	1	1	0	692.3505
116657	5	4	6.4	4206	1	1	0	739.7751
116657	5	4	6.4	4574	1	1	0	1152.0992
16590	6	3	31.8	4686	1	1	1	1182.1366
16590	6	3	31.8	4940	1	1	1	854.8044
16590	6	3	31.8	5330	1	1	1	936.4584
86932	7	5	6.7	574	1	1	1	488.7829
86932	7	5	6.7	769	1	1	1	515.7984
86932	7	5	6.7	2096	1	1	1	691.8487
86932	7	5	6.7	2845	1	1	1	553.9795
86932	7	5	6.7	2933	1	1	1	844.4745
109501	6	6	6.7	251	1	1	1	429.235
109501	6	6	6.7	516	1	1	1	480.7975
109501	6	6	6.7	682	1	1	1	503.7876
109501	6	6	6.7	792	1	1	1	518.7826
109501	6	6	6.7	2439	1	1	1	743.9272
109501	6	6	6.7	3091	1	1	1	874.4517
80376	7	6	9.9	16	1	1	1	357.7287
80376	7	6	9.9	1637	1	1	0	628.8513
80376	7	6	9.9	1761	1	1	0	646.3249
80376	7	6	9.9	1866	1	1	0	660.8332
80376	7	6	9.9	2533	1	1	1	764.4041
80376	7	6	9.9	2671	1	1	1	528.6014
47042	5	3	16.7	890	1	1	1	533.3026
47042	5	3	16.7	5633	1	1	1	1007.7668
47042	5	3	16.7	6170	1	1	1	858.9372
40903	3	2	8.3	2747	1	1	1	808.4265
40903	3	2	8.3	3129	1	1	1	881.4459
99776	3	3	6	2748	1	1	1	808.4561
99776	3	3	6	3398	1	1	1	933.5582
99776	3	3	6	4614	1	1	1	776.4273
65977	5	5	9.7	1331	1	1	1	591.2931
65977	5	5	9.7	1763	1	1	1	646.7922
65977	5	5	9.7	2536	1	1	1	765.411
65977	5	5	9.7	2561	1	1	1	768.9846
65977	5	5	9.7	4537	1	1	1	765.1225
59477	4	4	13.4	831	1	1	1	523.8138
59477	4	4	13.4	2610	1	1	1	778.8889
59477	4	4	13.4	3363	1	1	1	928.0329
59477	4	4	13.4	5809	1	1	1	1068.2233
42230	6	6	23	1243	1	1	1	579.8277
42230	6	6	23	1619	1	1	1	625.3134
42230	6	6	23	2266	1	1	1	714.4033
42230	6	6	23	2841	1	1	1	828.9567
42230	6	6	23	4814	1	1	1	815.4697
45049	7	7	18.7	759	1	1	1	514.3112
45049	7	7	18.7	862	1	1	1	529.3344
45049	7	7	18.7	928	1	1	1	538.3033

45049	7	7	18.7	1119	1	1	1	564.8042
45049	7	7	18.7	1126	1	1	1	566.2852
45049	7	7	18.7	1460	1	1	1	404.2228
45049	7	7	18.7	2522	1	1	1	762.9067
71322	4	4	7.2	643	1	1	1	498.8136
71322	4	4	7.2	669	1	1	1	502.7982
71322	4	4	7.2	2216	1	1	1	707.867
71322	4	4	7.2	3447	1	1	1	941.4749
137563	5	5	5.6	785	1	0	0	518.2811
137563	5	5	5.6	1436	1	0	0	603.2805
137563	5	5	5.6	2053	1	0	0	686.4033
137563	5	5	5.6	2797	1	1	1	820.429
137563	5	5	5.6	3227	1	0	0	900.4148
114571	7	7	8.4	830	1	1	1	523.8063
114571	7	7	8.4	1377	1	1	1	595.8301
114571	7	7	8.4	2364	1	1	1	730.8725
114571	7	7	8.4	2508	1	1	1	760.3985
114571	7	7	8.4	3354	1	1	1	925.4242
114571	7	7	8.4	3750	1	1	1	669.3185
114571	7	7	8.4	3917	1	1	1	697.0231
44425	6	4	16.8	579	1	1	1	489.7664
44425	6	4	16.8	2540	1	1	1	766.3656
44425	6	4	16.8	2920	1	1	1	841.9239
44425	6	4	16.8	5683	1	1	1	1026.5204
62339	4	4	7.9	715	1	1	1	508.7663
62339	4	4	7.9	1399	1	1	1	598.8713
62339	4	4	7.9	1678	1	1	1	634.2916
62339	4	4	7.9	2490	1	1	1	754.8864
59751	5	5	15.4	1998	1	1	1	679.4079
59751	5	5	15.4	2045	1	0	0	685.3194
59751	5	5	15.4	2534	1	1	1	764.4533
59751	5	5	15.4	3568	1	1	1	642.3981
59751	5	5	15.4	5623	1	1	1	999.1469
182693	3	3	2.7	1073	1	1	1	557.3293
182693	3	3	2.7	3277	1	1	1	908.4752
182693	3	3	2.7	3996	1	1	1	712.6739
20970	3	3	18.2	224	1	1	1	422.2496
20970	3	3	18.2	1336	1	1	1	591.3534
20970	3	3	18.2	2591	1	1	1	775.8853
67582	5	5	9.1	1015	1	1	1	549.8008
67582	5	5	9.1	1184	1	1	1	572.8359
67582	5	5	9.1	1348	1	1	1	592.3197
67582	5	5	9.1	1652	1	1	1	630.8449
67582	5	5	9.1	1905	1	1	1	665.8611
97167	4	4	7.5	854	1	1	1	527.7979
97167	4	4	7.5	1924	1	1	1	668.3489
97167	4	4	7.5	2806	1	1	1	822.4251
97167	4	4	7.5	5156	1	1	1	908.825
99113	4	4	6.3	1599	1	1	1	622.8945
99113	4	4	6.3	1775	1	1	1	647.8632
99113	4	4	6.3	2275	1	1	1	716.8451
99113	4	4	6.3	4657	1	1	1	784.1331
57763	5	4	13.1	1865	1	1	1	440.8813
57763	5	4	13.1	2605	1	1	1	778.3968
57763	5	4	13.1	3576	1	1	1	643.6543
57763	5	4	13.1	4908	1	1	1	841.4657
45355	2	2	4.6	3360	1	1	1	927.0351
45355	2	2	4.6	3759	1	1	1	670.3928

116128	4	2	3.4	3200	1	1	1	895.0222
116128	4	2	3.4	4592	1	1	1	771.7957
31696	5	5	20.6	485	1	1	1	475.277
31696	5	5	20.6	868	1	1	1	530.7597
31696	5	5	20.6	1498	1	1	1	610.3319
31696	5	5	20.6	2198	1	1	1	704.883
31696	5	5	20.6	3335	1	1	1	922.4784
82214	2	2	3.9	1948	1	1	1	671.3858
82214	2	2	3.9	2778	1	1	1	816.4405
59195	3	2	8.8	3598	1	1	1	970.5361
59195	3	2	8.8	5584	1	1	1	987.4999
110942	6	6	8.8	324	1	1	1	441.7271
110942	6	6	8.8	1538	1	1	1	615.2933
110942	6	6	8.8	1543	1	1	1	615.3661
110942	6	6	8.8	2156	1	1	1	698.8695
110942	6	6	8.8	3705	1	1	1	662.0159
110942	6	6	8.8	5497	1	1	1	966.4945
17118	3	2	14.5	3282	1	1	1	910.4538
17118	3	2	14.5	4770	1	1	1	804.0769
44788	4	3	10.3	782	1	1	1	517.7686
44788	4	3	10.3	2316	1	1	1	722.8315
44788	4	3	10.3	3388	1	1	1	621.3604
53612	3	2	6.8	807	1	1	1	520.3109
53612	3	2	6.8	5049	1	1	1	885.1406
77064	5	5	8.4	296	1	1	0	435.7739
77064	5	5	8.4	696	1	1	1	505.8032
77064	5	5	8.4	2370	1	1	0	731.3877
77064	5	5	8.4	2660	1	0	0	790.4075
77064	5	5	8.4	3108	1	1	1	876.4499
32347	3	3	11.7	1797	1	1	1	651.3582
32347	3	3	11.7	1814	1	1	1	654.3278
32347	3	3	11.7	2057	1	1	1	687.3455
71262	3	3	6.9	2353	1	1	1	728.8909
71262	3	3	6.9	2583	1	1	1	774.9047
71262	3	3	6.9	2611	1	1	1	779.9436
31671	4	4	18.2	929	1	1	1	538.761
31671	4	4	18.2	2299	1	1	1	719.3844
31671	4	4	18.2	2376	1	1	1	733.3938
31671	4	4	18.2	2734	1	1	1	805.4057
123905	5	5	5	253	1	1	1	429.2454
123905	5	5	5	817	1	1	1	522.2853
123905	5	5	5	1566	1	1	1	618.7969
123905	5	5	5	1945	1	1	1	671.3379
123905	5	5	5	2879	1	1	1	836.4706
271827	5	5	3.1	580	1	1	1	489.7729
271827	5	5	3.1	3006	1	1	1	858.509
271827	5	5	3.1	3185	1	1	1	593.9914
271827	5	5	3.1	3190	1	1	1	891.5072
271827	5	5	3.1	4768	1	1	1	803.773
109254	6	5	5.9	147	1	1	1	400.2556
109254	6	5	5.9	915	1	1	1	536.8293
109254	6	5	5.9	1534	1	1	0	410.2317
109254	6	5	5.9	2742	1	1	0	807.4354
109254	6	5	5.9	3373	1	1	0	619.6582
264216	3	2	1.3	2658	1	1	1	790.3574
264216	3	2	1.3	3612	1	1	1	973.0231
73187	6	4	8.2	1341	1	1	1	591.3663
73187	6	4	8.2	1559	1	1	1	617.352

73187	6	4	8.2	2359	1	1	1	730.3874
73187	6	4	8.2	3451	1	1	1	628.0395
406216	2	2	0.7	1748	1	1	1	643.392
406216	2	2	0.7	2597	1	1	1	776.4609
79562	4	2	5.2	3004	1	1	1	572.6418
79562	4	2	5.2	4672	1	1	1	1178.5791
47357	3	3	10.9	1684	1	1	1	634.8097
47357	3	3	10.9	3621	1	1	1	975.4362
47357	3	3	10.9	4221	1	1	1	1110.5721
105384	3	3	7.4	3255	1	1	1	604.0026
105384	3	3	7.4	3413	1	1	1	936.9776
105384	3	3	7.4	6329	1	1	1	1342.6817
57853	2	2	6.6	3121	1	1	1	879.9961
57853	2	2	6.6	3661	1	1	1	656.341
119640	3	3	3.7	1161	1	1	1	570.3608
119640	3	3	3.7	2219	1	1	1	708.3979
119640	3	3	3.7	3633	1	1	1	653.6644
17889	3	3	26.9	592	1	1	1	491.2922
17889	3	3	26.9	3138	1	1	1	882.4487
17889	3	3	26.9	3967	1	1	1	705.3912
55890	6	6	13.1	364	1	1	1	303.1904
55890	6	6	13.1	527	1	1	1	481.769
55890	6	6	13.1	808	1	1	1	520.311
55890	6	6	13.1	1064	1	1	1	556.7725
55890	6	6	13.1	2587	1	1	1	517.2874
55890	6	6	13.1	3797	1	1	1	1015.4575
19009	2	2	14.6	1642	1	1	1	629.374
19009	2	2	14.6	2109	1	1	1	692.8594
89064	3	3	6.3	2438	1	1	1	743.3796
89064	3	3	6.3	3754	1	1	1	1003.9864
89064	3	3	6.3	3781	1	1	1	1009.5239
92207	3	3	4.1	575	1	1	1	489.2616
92207	3	3	4.1	1542	1	1	1	615.3585
92207	3	3	4.1	2630	1	1	1	784.4745
109365	5	4	5.1	796	1	1	1	519.2666
109365	5	4	5.1	1534	1	0	0	410.2317
109365	5	4	5.1	2742	1	0	0	807.4354
109365	5	4	5.1	3373	1	0	0	619.6582
22118	4	3	17	341	1	1	1	449.7536
22118	4	3	17	1299	1	1	1	585.8054
22118	4	3	17	2669	1	1	1	791.9302
8974	3	2	33.8	5020	1	1	1	876.3576
8974	3	2	33.8	5502	1	1	1	968.0786
18045	3	2	16.9	1683	1	1	1	634.4024
18045	3	2	16.9	2929	1	1	1	844.405
112934	4	4	7	1000	1	1	1	546.8061
112934	4	4	7	2850	1	1	1	554.6383
112934	4	4	7	4071	1	1	1	1084.0609
112934	4	4	7	5679	1	1	1	1025.8601
69692	2	2	4.3	1369	1	1	1	594.8348
69692	2	2	4.3	3089	1	1	1	874.4459
118478	4	4	6.4	2044	1	1	1	684.8439
118478	4	4	6.4	2210	1	1	1	471.2746
118478	4	4	6.4	3033	1	1	1	862.0163
118478	4	4	6.4	5716	1	1	1	1040.5776
27673	3	3	13.2	143	1	1	1	399.7633
27673	3	3	13.2	1756	1	1	1	644.8368
27673	3	3	13.2	2246	1	1	1	712.3366

50306	2	2	8.8	3583	1	1	1	967.5197
20667	3	2	21	3919	1	1	1	697.3696
20667	3	2	21	4794	1	1	1	808.7273
67813	3	3	5.8	841	1	1	1	525.2668
67813	3	3	5.8	1527	1	1	1	613.8585
67813	3	3	5.8	3113	1	1	1	877.4859
40331	3	3	11.1	1372	1	1	1	595.3244
40331	3	3	11.1	1956	1	1	1	672.3613
40331	3	3	11.1	1987	1	1	1	676.825
38472	2	2	8.5	2263	1	1	1	713.8857
38472	2	2	8.5	3127	1	1	1	880.9543
48086	2	2	6.9	2110	1	1	1	692.8926
48086	2	2	6.9	3180	1	1	1	888.9577
196586	3	3	2.6	1428	1	1	1	601.8477
196586	3	3	2.6	2559	1	1	1	768.4802
196586	3	3	2.6	4115	1	1	1	729.7449
39436	3	3	9.9	1380	1	1	1	596.3047
39436	3	3	9.9	1676	1	1	1	633.8746
39436	3	3	9.9	2496	1	1	1	757.4147
16320	3	3	23.3	853	1	1	1	527.7848
16320	3	3	23.3	1239	1	1	1	579.3298
16320	3	3	23.3	2312	1	1	1	722.3691
28210	3	3	11.2	72	1	1	0	378.2605
28210	3	3	11.2	95	1	1	0	387.7652
28210	3	3	11.2	2637	1	1	1	786.4355
39816	2	2	10.4	1256	1	1	1	580.8429
39816	2	2	10.4	5538	1	1	1	975.8522
30915	2	2	13.5	3618	1	1	1	973.9824
30915	2	2	13.5	4116	1	1	1	730.0899
52204	2	2	5.8	1180	1	1	1	571.8483
52204	2	2	5.8	3309	1	1	1	917.4202
117180	2	2	2.9	1234	1	1	1	578.856
117180	2	2	2.9	4102	1	1	1	728.4026
50747	3	3	10	1123	1	1	1	565.7932
50747	3	3	10	1636	1	1	1	628.8384
50747	3	3	10	4840	1	1	1	1237.659
62798	4	4	9.4	1322	1	0	0	589.3142
62798	4	4	9.4	1784	1	1	1	649.8478
62798	4	4	9.4	2473	1	1	1	750.9555
62798	4	4	9.4	3774	1	0	0	672.3501
166282	3	3	2.7	1158	1	1	0	570.2935
166282	3	3	2.7	3419	1	1	1	937.4264
166282	3	3	2.7	3626	1	1	1	652.6625
35965	2	2	9.9	2441	1	1	1	744.8614
35965	2	2	9.9	3634	1	1	1	980.4872
92163	2	2	2.6	1207	1	1	1	575.806
92163	2	2	2.6	1849	1	1	1	658.8658
25092	2	2	13.1	2614	1	1	1	781.3648
25092	2	2	13.1	2976	1	1	1	852.9484
49361	2	2	6.6	1270	1	1	1	582.3174
49361	2	2	6.6	3527	1	1	1	954.0045
52150	5	4	11.1	227	1	1	1	422.7682
52150	5	4	11.1	986	1	1	1	544.7921
52150	5	4	11.1	1586	1	1	1	621.8216
52150	5	4	11.1	5051	1	1	1	886.1279
86221	2	2	4.1	777	1	1	1	516.8135
86221	2	2	4.1	4697	1	1	1	1184.1989
54004	3	3	6	747	1	1	1	513.2927

54004	3	3	6	1040	1	1	1	552.8116
54004	3	3	6	1215	1	1	1	576.8032
188232	2	2	1.9	1490	1	1	1	609.801
188232	2	2	1.9	3977	1	1	1	1060.5647
102646	2	2	2.3	766	1	1	1	515.3065
102646	2	2	2.3	1481	1	1	1	608.3327
276563	5	5	3.1	865	1	1	1	529.7956
276563	5	5	3.1	1101	1	1	1	561.319
276563	5	5	3.1	3224	1	1	1	899.4274
276563	5	5	3.1	4005	1	1	1	714.0598
276563	5	5	3.1	4109	1	1	1	728.6894
29708	3	3	16.1	823	1	1	0	523.242
29708	3	3	16.1	1187	1	1	1	573.3023
29708	3	3	16.1	5473	1	1	1	1442.2214
146592	3	3	3.7	1882	1	0	0	663.3387
146592	3	3	3.7	3208	1	0	0	897.5034
146592	3	3	3.7	4262	1	1	1	744.423
141519	2	2	2.2	1588	1	1	1	621.8868
141519	2	2	2.2	3015	1	1	1	859.4423
81995	3	3	5.1	1444	1	1	1	603.7933
81995	3	3	5.1	2339	1	1	1	484.6362
81995	3	3	5.1	2515	1	1	1	762.3418
32499	2	2	8.7	457	1	1	1	470.795
32499	2	2	8.7	3901	1	1	1	694.6497
124495	4	4	3.9	695	1	1	1	505.7745
124495	4	4	3.9	708	1	1	1	338.5585
124495	4	4	3.9	1781	1	1	1	648.8849
124495	4	4	3.9	3053	1	1	1	577.9958
67832	2	2	4	1886	1	1	1	663.8568
67832	2	2	4	2152	1	1	1	698.3883
64176	3	3	5.3	2647	1	1	1	787.9817
64176	3	3	5.3	2839	1	1	1	827.5003
64176	3	3	5.3	3387	1	1	1	620.7045
34706	3	2	9.1	922	1	1	1	537.3188
34706	3	2	9.1	3677	1	1	1	658.3824
30950	2	2	9.2	2127	1	1	1	695.343
30950	2	2	9.2	2840	1	1	1	828.4285
107161	2	2	3.3	2711	1	1	1	799.9416
107161	2	2	3.3	3988	1	1	1	709.6885
33703	2	2	14.8	2211	1	1	1	706.4241
33703	2	2	14.8	6233	1	1	1	1184.3096
105370	2	2	2.5	1289	1	1	1	584.3089
105370	2	2	2.5	2205	1	1	1	706.3199
60657	2	2	3.5	404	1	1	1	459.2295
60657	2	2	3.5	1647	1	1	1	630.3332
86361	3	3	4.3	186	1	1	1	412.2267
86361	3	3	4.3	1223	1	1	1	577.8483
86361	3	3	4.3	2834	1	1	1	826.0056
36651	2	2	7.6	1651	1	1	1	630.7778
36651	2	2	7.6	2823	1	1	1	550.2747
147050	2	2	1.9	3030	1	1	1	861.4872
86196	3	3	4.3	76	1	1	1	379.2319
86196	3	3	4.3	1523	1	1	1	613.823
86196	3	3	4.3	2492	1	1	1	504.6184
53639	2	2	7.4	1010	1	1	1	548.3016
53639	2	2	7.4	5480	1	1	1	963.1286
111275	2	2	2.1	764	1	1	1	515.2875
111275	2	2	2.1	1974	1	1	1	674.8478

60872	3	3	5.9	1039	1	1	1	552.777
60872	3	3	5.9	1614	1	1	1	624.8248
60872	3	3	5.9	1960	1	1	1	672.8565
22787	2	2	12.6	2318	1	1	1	723.3281
22787	2	2	12.6	2664	1	1	1	790.8846
15188	2	2	20	486	1	1	1	475.2895
15188	2	2	20	3751	1	1	1	1003.4886
93010	2	2	4.3	931	1	1	1	538.7877
93010	2	2	4.3	5435	1	1	1	954.1726
32983	2	2	6.2	487	1	1	1	475.7476
32983	2	2	6.2	861	1	1	1	529.3219
59093	3	3	7.5	1567	1	0	0	618.8135
59093	3	3	7.5	2045	1	0	0	685.3194
59093	3	3	7.5	3544	1	1	1	638.7228
14066	2	2	18.5	1252	1	1	1	580.817
14066	2	2	18.5	1281	1	1	0	583.8069
59922	2	2	5.4	2045	1	0	0	685.3194
59922	2	2	5.4	3628	1	1	1	652.7263
23079	2	2	17.2	836	1	1	1	524.2957
23079	2	2	17.2	5560	1	1	1	981.5093
14095	2	2	18.5	1191	1	1	1	573.8093
14095	2	2	18.5	1281	1	0	0	583.8069
92840	2	2	3.8	1946	1	1	1	671.3591
92840	2	2	3.8	3536	1	1	1	955.0232
24852	2	2	13	1629	1	1	1	627.3331
24852	2	2	13	3275	1	1	1	907.9928
29193	2	2	7.7	321	1	1	1	441.2949
29193	2	2	7.7	1798	1	1	1	651.8146
97570	3	3	5	667	1	1	1	502.7731
97570	3	3	5	4860	1	1	1	830.0875
40742	2	2	5.6	917	1	1	1	537.2488
40742	2	2	5.6	1487	1	1	1	609.3478
17142	3	3	21.2	195	1	1	1	415.2606
17142	3	3	21.2	2092	1	1	1	461.2888
17142	3	3	21.2	2133	1	1	1	696.8185
39776	2	2	6.3	1459	1	1	1	605.8273
39776	2	2	6.3	1578	1	1	1	620.8374
29898	2	2	7.7	470	1	1	1	472.7741
29898	2	2	7.7	2384	1	1	1	490.2938
28013	2	2	11.6	716	1	1	1	508.7697
28013	2	2	11.6	3742	1	1	1	668.3222
33803	2	2	6.6	1136	1	1	1	567.2619
33803	2	2	6.6	1579	1	1	1	620.8484
77766	2	2	5.1	903	1	1	1	534.8062
77766	2	2	5.1	4953	1	1	1	857.4619
86850	2	2	2.9	1298	1	1	1	585.7857
86850	2	2	2.9	1627	1	1	1	626.3196
15496	2	2	17.2	533	1	1	1	482.7275
15496	2	2	17.2	2951	1	1	1	566.0049
57702	2	2	9.3	2349	1	1	1	485.5835
57702	2	2	9.3	6318	1	1	1	1319.6928
prot_mass	prot_matches	prot_sequenc	prot_cover	pep_query	pep_rank	pep_isbold	pep_isunique	pep_exp_mz
50361	31	18	67.1	576	1	1	1	488.7421
50361	31	18	67.1	753	1	1	1	514.2822
50361	31	18	67.1	1145	1	1	0	570.3503
50361	31	18	67.1	1171	1	1	0	573.7982
50361	31	18	67.1	1459	1	1	0	608.2946
50361	31	18	67.1	1767	1	1	1	650.3141

50361	31	18	67.1	2187	1	1	0	701.8318
50361	31	18	67.1	2747	1	1	0	811.9294
50361	31	18	67.1	2794	1	1	0	824.4074
50361	31	18	67.1	2908	1	1	0	848.9196
50361	31	18	67.1	3163	1	1	0	911.9646
50361	31	18	67.1	3400	1	1	0	976.4465
50361	31	18	67.1	3435	1	1	0	987.0013
50361	31	18	67.1	4269	1	1	1	1184.0677
50361	31	18	67.1	4615	1	1	1	922.1119
50361	31	18	67.1	4659	1	1	0	933.4512
50361	31	18	67.1	4913	1	1	1	1067.4949
50361	31	18	67.1	4956	1	1	1	1104.1748
51385	27	15	46	858	1	1	0	530.2678
51385	27	15	46	1145	1	0	0	570.3503
51385	27	15	46	1171	1	0	0	573.7982
51385	27	15	46	1459	1	0	0	608.2946
51385	27	15	46	1757	1	1	1	648.8068
51385	27	15	46	2114	1	1	0	692.8536
51385	27	15	46	2350	1	1	1	723.3273
51385	27	15	46	2354	1	1	0	723.8474
51385	27	15	46	2747	1	0	0	811.9294
51385	27	15	46	2794	1	0	0	824.4074
51385	27	15	46	2908	1	0	0	848.9196
51385	27	15	46	3163	1	0	0	911.9646
51385	27	15	46	3400	1	0	0	976.4465
51385	27	15	46	3435	1	0	0	987.0013
51385	27	15	46	4666	1	1	1	934.7709
71559	27	19	40.5	1461	1	1	0	608.3295
71559	27	19	40.5	1496	1	1	1	613.7988
71559	27	19	40.5	1507	1	1	0	614.8178
71559	27	19	40.5	1693	1	1	0	639.8182
71559	27	19	40.5	1754	1	1	1	648.3354
71559	27	19	40.5	1815	1	1	1	654.8559
71559	27	19	40.5	1999	1	1	0	679.811
71559	27	19	40.5	2122	1	1	0	693.8677
71559	27	19	40.5	2208	1	1	1	704.3851
71559	27	19	40.5	2297	1	1	1	716.3755
71559	27	19	40.5	2425	1	1	0	737.3458
71559	27	19	40.5	2556	1	1	1	508.2719
71559	27	19	40.5	2689	1	1	1	798.9132
71559	27	19	40.5	2823	1	1	1	831.4196
71559	27	19	40.5	2856	1	1	0	838.3683
71559	27	19	40.5	2874	1	1	0	840.9208
71559	27	19	40.5	4450	1	1	0	1287.6384
71559	27	19	40.5	4455	1	1	0	863.4107
71559	27	19	40.5	4492	1	1	0	1329.6381
51258	27	14	43.7	858	1	0	0	530.2678
51258	27	14	43.7	1145	1	0	0	570.3503
51258	27	14	43.7	1171	1	0	0	573.7982
51258	27	14	43.7	1459	1	0	0	608.2946
51258	27	14	43.7	2114	1	0	0	692.8536
51258	27	14	43.7	2296	1	1	0	716.3189
51258	27	14	43.7	2354	1	0	0	723.8474
51258	27	14	43.7	2747	1	0	0	811.9294
51258	27	14	43.7	2763	1	1	0	817.4001
51258	27	14	43.7	2908	1	0	0	848.9196
51258	27	14	43.7	3163	1	0	0	911.9646
51258	27	14	43.7	3400	1	0	0	976.4465

51258	27	14	43.7	3435	1	0	0	987.0013
51258	27	14	43.7	4684	1	1	0	938.7836
71712	29	18	38.6	1461	1	0	0	608.3295
71712	29	18	38.6	1507	1	0	0	614.8178
71712	29	18	38.6	1512	1	1	1	615.7687
71712	29	18	38.6	1693	1	0	0	639.8182
71712	29	18	38.6	1827	1	1	0	657.3131
71712	29	18	38.6	1999	1	0	0	679.811
71712	29	18	38.6	2122	1	0	0	693.8677
71712	29	18	38.6	2284	1	1	1	713.8855
71712	29	18	38.6	2322	1	1	0	718.8799
71712	29	18	38.6	2425	1	0	0	737.3458
71712	29	18	38.6	2604	1	1	0	514.2573
71712	29	18	38.6	2653	1	1	1	783.9077
71712	29	18	38.6	2828	1	1	0	832.4083
71712	29	18	38.6	2856	1	0	0	838.3683
71712	29	18	38.6	2874	1	0	0	840.9208
71712	29	18	38.6	4434	1	1	1	1280.6289
71712	29	18	38.6	4455	1	0	0	863.4107
71712	29	18	38.6	4492	1	0	0	1329.6381
51341	27	14	42.3	858	1	0	0	530.2678
51341	27	14	42.3	1145	1	0	0	570.3503
51341	27	14	42.3	1171	1	0	0	573.7982
51341	27	14	42.3	1459	1	0	0	608.2946
51341	27	14	42.3	1810	1	1	1	654.8246
51341	27	14	42.3	2187	1	0	0	701.8318
51341	27	14	42.3	2296	1	0	0	716.3189
51341	27	14	42.3	2354	1	0	0	723.8474
51341	27	14	42.3	2747	1	0	0	811.9294
51341	27	14	42.3	2763	1	0	0	817.4001
51341	27	14	42.3	2908	1	0	0	848.9196
51341	27	14	42.3	3400	1	0	0	976.4465
51341	27	14	42.3	3435	1	0	0	987.0013
51341	27	14	42.3	4684	1	0	0	938.7836
50194	19	14	49.6	608	1	1	0	494.2772
50194	19	14	49.6	1647	1	1	0	634.2579
50194	19	14	49.6	2162	1	1	0	698.882
50194	19	14	49.6	2430	1	1	1	737.435
50194	19	14	49.6	2698	1	1	0	799.8873
50194	19	14	49.6	2829	1	1	1	832.4408
50194	19	14	49.6	2918	1	1	0	851.4559
50194	19	14	49.6	3092	1	1	1	896.967
50194	19	14	49.6	3234	1	1	1	929.4441
50194	19	14	49.6	3289	1	1	0	943.477
50194	19	14	49.6	3444	1	1	1	989.4445
50194	19	14	49.6	4204	1	1	0	777.3443
50194	19	14	49.6	4309	1	1	1	1198.0996
50194	19	14	49.6	4471	1	1	1	875.7481
46960	19	14	41	440	1	1	1	468.2633
46960	19	14	41	509	1	1	0	479.7638
46960	19	14	41	578	1	1	1	488.7822
46960	19	14	41	716	1	1	0	509.2767
46960	19	14	41	1050	1	1	0	557.8451
46960	19	14	41	1156	1	1	0	571.7898
46960	19	14	41	1289	1	1	0	587.3275
46960	19	14	41	2184	1	1	0	701.3696
46960	19	14	41	2517	1	1	0	751.9428
46960	19	14	41	2657	1	1	0	786.3575

46960	19	14	41	3174	1	1	0	914.4726
46960	19	14	41	3347	1	1	0	961.0044
46960	19	14	41	3548	1	1	0	676.6358
46960	19	14	41	3630	1	1	0	1030.048
47075	18	13	39.8	509	1	0	0	479.7638
47075	18	13	39.8	716	1	0	0	509.2767
47075	18	13	39.8	1050	1	0	0	557.8451
47075	18	13	39.8	1156	1	0	0	571.7898
47075	18	13	39.8	1289	1	0	0	587.3275
47075	18	13	39.8	2184	1	0	0	701.3696
47075	18	13	39.8	2358	1	1	1	724.3851
47075	18	13	39.8	2517	1	0	0	751.9428
47075	18	13	39.8	2657	1	0	0	786.3575
47075	18	13	39.8	3174	1	0	0	914.4726
47075	18	13	39.8	3347	1	0	0	961.0044
47075	18	13	39.8	3548	1	0	0	676.6358
47075	18	13	39.8	3630	1	0	0	1030.048
71741	16	11	25.3	1389	1	1	1	599.3509
71741	16	11	25.3	1507	1	0	0	614.8178
71741	16	11	25.3	1693	1	0	0	639.8182
71741	16	11	25.3	1999	1	0	0	679.811
71741	16	11	25.3	2322	1	0	0	718.8799
71741	16	11	25.3	2425	1	0	0	737.3458
71741	16	11	25.3	2828	1	0	0	832.4083
71741	16	11	25.3	2856	1	0	0	838.3683
71741	16	11	25.3	2874	1	0	0	840.9208
71741	16	11	25.3	4450	1	0	0	1287.6384
71741	16	11	25.3	4455	1	0	0	863.4107
71456	20	13	26.8	1507	1	0	0	614.8178
71456	20	13	26.8	1519	1	1	1	616.3271
71456	20	13	26.8	1616	1	1	1	630.8448
71456	20	13	26.8	1693	1	0	0	639.8182
71456	20	13	26.8	1827	1	0	0	657.3131
71456	20	13	26.8	1999	1	0	0	679.811
71456	20	13	26.8	2322	1	0	0	718.8799
71456	20	13	26.8	2425	1	0	0	737.3458
71456	20	13	26.8	2604	1	0	0	514.2573
71456	20	13	26.8	2856	1	0	0	838.3683
71456	20	13	26.8	2874	1	0	0	840.9208
71456	20	13	26.8	4450	1	0	0	1287.6384
71456	20	13	26.8	4455	1	0	0	863.4107
58863	19	15	37.8	435	1	1	1	467.237
58863	19	15	37.8	502	1	1	1	478.2896
58863	19	15	37.8	986	1	1	1	546.3027
58863	19	15	37.8	1236	1	1	1	581.2936
58863	19	15	37.8	1248	1	1	1	582.3242
58863	19	15	37.8	1855	1	1	1	659.3651
58863	19	15	37.8	2102	1	1	1	691.7967
58863	19	15	37.8	2152	1	1	1	697.377
58863	19	15	37.8	2723	1	1	1	805.443
58863	19	15	37.8	2805	1	1	1	826.4133
58863	19	15	37.8	2919	1	1	1	851.8722
58863	19	15	37.8	2955	1	1	1	860.9398
58863	19	15	37.8	3434	1	1	1	986.0309
58863	19	15	37.8	3873	1	1	1	1099.0582
58863	19	15	37.8	4490	1	1	1	886.4542
51237	16	10	26.9	858	1	0	0	530.2678
51237	16	10	26.9	1145	1	0	0	570.3503

51237	16	10	26.9	1171	1	0	0	573.7982
51237	16	10	26.9	1459	1	0	0	608.2946
51237	16	10	26.9	2114	1	0	0	692.8536
51237	16	10	26.9	2296	1	0	0	716.3189
51237	16	10	26.9	2354	1	0	0	723.8474
51237	16	10	26.9	2747	1	0	0	811.9294
51237	16	10	26.9	2908	1	0	0	848.9196
51237	16	10	26.9	3404	1	1	0	653.0054
10285	9	5	54.6	404	1	1	1	460.7471
10285	9	5	54.6	460	1	1	1	471.2578
10285	9	5	54.6	2330	1	1	1	720.3467
10285	9	5	54.6	2508	1	1	1	751.3593
10285	9	5	54.6	3797	1	1	1	1078.515
50250	13	9	28.4	608	1	0	0	494.2772
50250	13	9	28.4	1647	1	0	0	634.2579
50250	13	9	28.4	2162	1	0	0	698.882
50250	13	9	28.4	2585	1	1	1	766.4562
50250	13	9	28.4	2678	1	1	1	792.8796
50250	13	9	28.4	2918	1	0	0	851.4559
50250	13	9	28.4	3258	1	1	0	934.9525
50250	13	9	28.4	3289	1	0	0	943.477
50250	13	9	28.4	4204	1	0	0	777.3443
37004	10	8	37.3	189	1	1	1	417.2349
37004	10	8	37.3	2497	1	1	1	749.9275
37004	10	8	37.3	2859	1	1	1	559.6627
37004	10	8	37.3	2982	1	1	1	867.9108
37004	10	8	37.3	3024	1	1	1	881.3968
37004	10	8	37.3	3563	1	1	1	1017.0392
37004	10	8	37.3	3825	1	1	1	1086.5078
37004	10	8	37.3	4011	1	1	1	1125.5397
51883	15	11	31.1	166	1	1	1	412.2554
51883	15	11	31.1	1000	1	1	1	548.3264
51883	15	11	31.1	1103	1	1	1	565.3265
51883	15	11	31.1	1221	1	1	1	579.3291
51883	15	11	31.1	2132	1	1	1	695.3392
51883	15	11	31.1	2315	1	1	1	718.338
51883	15	11	31.1	2880	1	1	1	841.4781
51883	15	11	31.1	3058	1	1	0	590.2667
51883	15	11	31.1	3140	1	1	1	906.9896
51883	15	11	31.1	3354	1	1	1	962.0271
51883	15	11	31.1	3723	1	1	1	1057.5284
49813	17	12	37.4	389	1	1	1	457.7869
49813	17	12	37.4	679	1	1	1	336.5228
49813	17	12	37.4	742	1	1	1	513.3083
49813	17	12	37.4	744	1	1	1	513.7478
49813	17	12	37.4	1067	1	1	1	560.803
49813	17	12	37.4	1442	1	1	1	605.7918
49813	17	12	37.4	1765	1	1	1	649.8767
49813	17	12	37.4	2372	1	1	1	726.3399
49813	17	12	37.4	2764	1	1	1	545.6386
49813	17	12	37.4	4160	1	1	1	771.3799
49813	17	12	37.4	4406	1	1	1	1253.6677
49813	17	12	37.4	4806	1	1	1	986.4788
40001	12	8	27.2	1215	1	1	1	578.806
40001	12	8	27.2	1374	1	1	0	597.3351
40001	12	8	27.2	1466	1	1	0	609.3064
40001	12	8	27.2	1698	1	1	1	640.8181
40001	12	8	27.2	2271	1	1	0	712.3353

40001	12	8	27.2	2464	1	1	1	742.4141
40001	12	8	27.2	2712	1	1	1	801.9391
40001	12	8	27.2	3119	1	1	1	902.4705
41112	9	8	23.5	121	1	1	1	397.2261
41112	9	8	23.5	154	1	1	0	408.7505
41112	9	8	23.5	1479	1	1	0	611.8192
41112	9	8	23.5	2453	1	1	0	740.3932
41112	9	8	23.5	2533	1	1	1	758.3614
41112	9	8	23.5	2881	1	1	1	842.4222
41112	9	8	23.5	3144	1	1	1	907.5044
41112	9	8	23.5	3219	1	1	1	923.9314
73174	10	9	16.3	250	1	1	1	429.2639
73174	10	9	16.3	1104	1	1	1	565.7881
73174	10	9	16.3	1359	1	1	1	595.8418
73174	10	9	16.3	1529	1	1	1	618.2907
73174	10	9	16.3	2252	1	1	1	709.3669
73174	10	9	16.3	2253	1	1	0	709.3711
73174	10	9	16.3	2621	1	1	0	775.9017
73174	10	9	16.3	2730	1	1	1	538.6295
73174	10	9	16.3	3161	1	1	1	911.4565
73315	10	9	16.4	363	1	1	1	453.2326
73315	10	9	16.4	1572	1	1	1	624.8454
73315	10	9	16.4	1574	1	1	1	625.2859
73315	10	9	16.4	1750	1	1	1	647.7941
73315	10	9	16.4	1887	1	1	1	664.3225
73315	10	9	16.4	2018	1	1	1	681.3738
73315	10	9	16.4	2253	1	0	0	709.3711
73315	10	9	16.4	2621	1	0	0	775.9017
73315	10	9	16.4	3141	1	1	1	907.4805
40949	8	7	21.9	154	1	0	0	408.7505
40949	8	7	21.9	1479	1	0	0	611.8192
40949	8	7	21.9	2453	1	0	0	740.3932
40949	8	7	21.9	2533	1	0	1	758.3614
40949	8	7	21.9	2968	1	1	1	863.8913
40949	8	7	21.9	3049	1	1	1	883.4599
40949	8	7	21.9	3111	1	1	1	900.972
136636	8	7	7.5	226	1	1	1	424.7528
136636	8	7	7.5	1657	1	1	1	635.3616
136636	8	7	7.5	1678	1	1	1	638.8283
136636	8	7	7.5	2412	1	1	1	734.8952
136636	8	7	7.5	2463	1	1	1	742.3843
136636	8	7	7.5	2655	1	1	1	784.9191
136636	8	7	7.5	3984	1	1	1	1121.1203
52347	4	4	14.3	1203	1	1	1	576.8604
52347	4	4	14.3	2910	1	1	1	849.3833
52347	4	4	14.3	3780	1	1	1	1075.5691
52347	4	4	14.3	4236	1	1	1	1174.0976
166282	7	7	6.8	1143	1	1	0	570.2942
166282	7	7	6.8	1836	1	1	1	658.3521
166282	7	7	6.8	2482	1	1	1	746.8749
166282	7	7	6.8	3264	1	1	1	937.426
166282	7	7	6.8	3403	1	1	1	652.662
166282	7	7	6.8	3461	1	1	1	993.9752
166282	7	7	6.8	3818	1	1	1	723.6675
41937	8	8	32.6	575	1	1	0	488.7278
41937	8	8	32.6	1164	1	1	0	572.7831
41937	8	8	32.6	1390	1	1	0	599.8563
41937	8	8	32.6	2435	1	1	1	738.3411

41937	8	8	32.6	2535	1	1	0	758.378
41937	8	8	32.6	3233	1	1	1	928.4698
41937	8	8	32.6	3841	1	1	1	1092.0424
41937	8	8	32.6	4898	1	1	0	1051.2183
29784	5	3	19	1551	1	1	1	621.8286
29784	5	3	19	3371	1	1	1	968.0203
29784	5	3	19	4352	1	1	1	1216.1221
49607	6	5	17.4	1453	1	1	1	607.343
49607	6	5	17.4	2685	1	1	1	794.8992
49607	6	5	17.4	3058	1	0	0	590.2667
49607	6	5	17.4	3748	1	1	1	1065.0406
49607	6	5	17.4	3888	1	1	1	735.3964
36185	8	6	25.8	1715	1	1	1	642.3817
36185	8	6	25.8	1788	1	1	1	652.3788
36185	8	6	25.8	1883	1	1	1	663.8107
36185	8	6	25.8	2531	1	1	1	505.284
36185	8	6	25.8	3063	1	1	1	885.4291
36185	8	6	25.8	4179	1	1	1	774.4086
23090	6	4	23.2	640	1	1	0	499.7851
23090	6	4	23.2	1373	1	1	0	398.544
23090	6	4	23.2	2191	1	1	1	702.4025
23090	6	4	23.2	2756	1	1	0	816.4183
39964	6	6	22.9	1374	1	0	0	597.3351
39964	6	6	22.9	1466	1	0	0	609.3064
39964	6	6	22.9	2271	1	0	0	712.3353
39964	6	6	22.9	2416	1	1	1	735.4058
39964	6	6	22.9	2913	1	1	1	567.6447
39964	6	6	22.9	4039	1	1	1	1131.0426
94743	5	4	7.4	2375	1	1	1	726.8484
94743	5	4	7.4	2512	1	1	1	751.8427
94743	5	4	7.4	3108	1	1	1	900.4694
94743	5	4	7.4	4313	1	1	1	800.3922
49487	5	4	15.3	1732	1	1	1	644.3576
49487	5	4	15.3	3011	1	1	1	876.4355
49487	5	4	15.3	3770	1	1	1	1073.0566
49487	5	4	15.3	3887	1	1	1	1102.5702
105902	9	8	11.3	563	1	1	1	486.7769
105902	9	8	11.3	757	1	1	0	515.2881
105902	9	8	11.3	827	1	1	1	525.7699
105902	9	8	11.3	1227	1	1	0	580.3002
105902	9	8	11.3	2167	1	1	0	699.3726
105902	9	8	11.3	2445	1	1	0	738.8946
105902	9	8	11.3	3306	1	1	1	946.5283
105902	9	8	11.3	4407	1	1	0	836.7989
53612	7	6	19.6	791	1	1	1	520.3109
53612	7	6	19.6	1571	1	1	1	624.8201
53612	7	6	19.6	1872	1	1	1	662.3356
53612	7	6	19.6	2321	1	1	1	718.8236
53612	7	6	19.6	4489	1	1	1	884.8048
53612	7	6	19.6	4741	1	1	1	964.8345
59440	5	5	15.7	1749	1	1	1	647.3427
59440	5	5	15.7	2055	1	1	1	685.3194
59440	5	5	15.7	2847	1	1	1	835.418
59440	5	5	15.7	4374	1	1	1	1235.0927
59440	5	5	15.7	4393	1	1	1	831.7916
57763	5	4	13.1	1287	1	1	1	587.3
57763	5	4	13.1	2169	1	1	1	699.8432
57763	5	4	13.1	4077	1	1	1	1136.5866

57763	5	4	13.1	4420	1	1	1	841.4651
32499	4	4	21.1	456	1	1	1	470.795
32499	4	4	21.1	3247	1	1	1	933.0282
32499	4	4	21.1	3668	1	1	1	694.6488
32499	4	4	21.1	3836	1	1	1	1089.0217
73869	5	5	10	1213	1	1	0	578.3274
73869	5	5	10	1759	1	1	0	648.8142
73869	5	5	10	2488	1	1	0	747.8647
73869	5	5	10	2648	1	1	0	782.9169
73869	5	5	10	3619	1	1	1	1027.0088
41563	7	7	18.6	91	1	1	0	386.2272
41563	7	7	18.6	396	1	1	0	459.2944
41563	7	7	18.6	884	1	1	1	533.7511
41563	7	7	18.6	2012	1	1	1	680.8597
41563	7	7	18.6	2211	1	1	1	470.2454
41563	7	7	18.6	2356	1	1	0	723.875
41563	7	7	18.6	3605	1	1	1	1024.512
42056	7	7	26.3	575	1	0	0	488.7278
42056	7	7	26.3	1110	1	1	1	566.7668
42056	7	7	26.3	1164	1	0	0	572.7831
42056	7	7	26.3	1390	1	0	0	599.8563
42056	7	7	26.3	2535	1	0	0	758.378
42056	7	7	26.3	3089	1	1	1	895.9489
42056	7	7	26.3	4898	1	0	0	1051.2183
41834	7	7	16.1	91	1	0	0	386.2272
41834	7	7	16.1	396	1	0	0	459.2944
41834	7	7	16.1	769	1	1	1	516.7592
41834	7	7	16.1	1505	1	1	1	614.8137
41834	7	7	16.1	2012	1	0	1	680.8597
41834	7	7	16.1	2188	1	1	1	468.2526
41834	7	7	16.1	2356	1	0	0	723.875
65547	6	6	12.2	1105	1	1	1	566.2387
65547	6	6	12.2	1226	1	1	1	580.2543
65547	6	6	12.2	1349	1	1	1	594.3551
65547	6	6	12.2	1621	1	1	1	631.3432
65547	6	6	12.2	2504	1	1	1	500.8996
65547	6	6	12.2	3147	1	1	1	908.4588
44356	4	4	16.6	9	1	1	1	355.7009
44356	4	4	16.6	1692	1	1	1	639.816
44356	4	4	16.6	3316	1	1	1	951.4755
44356	4	4	16.6	5022	1	1	1	1119.8656
73801	5	5	10	1213	1	0	0	578.3274
73801	5	5	10	1759	1	0	0	648.8142
73801	5	5	10	2488	1	0	0	747.8647
73801	5	5	10	2648	1	0	0	782.9169
73801	5	5	10	3526	1	1	1	1010.0175
29729	3	3	26.3	3114	1	1	1	901.3997
29729	3	3	26.3	4711	1	1	1	951.5054
29729	3	3	26.3	4805	1	1	1	986.1578
59765	4	4	9.2	574	1	1	1	488.2847
59765	4	4	9.2	2283	1	1	1	713.3907
59765	4	4	9.2	2388	1	1	1	729.4234
59765	4	4	9.2	2483	1	1	1	746.8911
61584	2	2	5.7	2528	1	1	0	754.8872
61584	2	2	5.7	3390	1	1	1	973.5328
43168	3	3	8.1	582	1	1	1	490.2401
43168	3	3	8.1	1432	1	1	1	603.8068
43168	3	3	8.1	2420	1	1	1	735.8698

44960	5	5	20.6	1225	1	1	0	579.8288
44960	5	5	20.6	1667	1	1	1	637.3236
44960	5	5	20.6	1681	1	1	1	638.8488
44960	5	5	20.6	3375	1	1	1	968.9883
44960	5	5	20.6	4926	1	1	1	1082.1902
17591	2	2	21.1	1983	1	1	1	678.3342
17591	2	2	21.1	3676	1	1	1	1043.0364
187789	2	2	1.9	2891	1	1	1	844.4469
187789	2	2	1.9	3192	1	1	1	919.4569
77064	5	5	7.2	288	1	1	1	435.7739
77064	5	5	7.2	390	1	1	1	458.2476
77064	5	5	7.2	687	1	1	1	505.803
77064	5	5	7.2	2393	1	1	1	731.3885
77064	5	5	7.2	2668	1	1	1	790.4083
47236	4	4	14.4	1225	1	0	0	579.8288
47236	4	4	14.4	2790	1	1	0	822.9421
47236	4	4	14.4	3290	1	1	0	943.9547
47236	4	4	14.4	3429	1	1	1	984.4721
80376	4	4	7.4	1599	1	1	0	628.8512
80376	4	4	7.4	1742	1	1	0	646.321
80376	4	4	7.4	2571	1	1	1	764.4039
80376	4	4	7.4	2673	1	1	1	528.6017
27673	3	2	10	1735	1	1	1	644.8376
27673	3	2	10	2272	1	1	1	712.3376
148890	2	2	2.4	2565	1	1	1	763.4572
148890	2	2	2.4	3380	1	1	1	971.0334
40630	3	3	9.5	647	1	1	1	500.7921
40630	3	3	9.5	1719	1	1	1	643.3627
40630	3	3	9.5	2246	1	1	1	708.3852
22118	5	4	25.8	345	1	1	1	449.7531
22118	5	4	25.8	1278	1	1	1	585.8058
22118	5	4	25.8	2671	1	1	1	791.9308
22118	5	4	25.8	3091	1	1	1	598.3051
264216	4	4	2.3	2502	1	1	1	750.4215
264216	4	4	2.3	2639	1	1	1	779.9511
264216	4	4	2.3	2667	1	1	1	790.3558
264216	4	4	2.3	2940	1	1	1	857.9204
19009	2	2	14.6	1602	1	1	1	629.3738
19009	2	2	14.6	2115	1	1	1	692.8588
103283	3	3	4.3	727	1	1	1	510.2586
103283	3	3	4.3	2402	1	1	1	731.8879
103283	3	3	4.3	3190	1	1	1	918.9737
47389	4	4	12.9	1225	1	0	0	579.8288
47389	4	4	12.9	1879	1	1	1	663.3212
47389	4	4	12.9	2456	1	1	1	493.9588
47389	4	4	12.9	3351	1	1	1	961.9799
154652	2	2	2.4	2360	1	1	1	724.4127
154652	2	2	2.4	3301	1	1	1	945.9991
83408	2	2	3.5	1598	1	1	1	628.8297
83408	2	2	3.5	2837	1	1	1	833.4279
14066	2	2	18.5	1231	1	1	1	580.8172
14066	2	2	18.5	1258	1	1	0	583.8049
65730	2	2	5.7	2193	1	1	1	702.8332
65730	2	2	5.7	3930	1	1	1	1112.0938
30915	2	2	12.4	3216	1	1	1	923.5068
30915	2	2	12.4	3392	1	1	1	973.9813
47005	3	3	11.7	2619	1	1	1	774.9482
47005	3	3	11.7	3406	1	1	1	654.6636

47005	3	3	11.7	3697	1	1	1	699.396
65220	2	2	6.9	2974	1	1	1	865.9236
65220	2	2	6.9	4485	1	1	1	881.7886
16304	2	2	16	837	1	1	1	527.7844
16304	2	2	16	2344	1	1	1	722.3691
20970	2	2	12.8	1320	1	1	1	591.3532
20970	2	2	12.8	2121	1	1	1	693.4032
98766	3	3	5.4	2192	1	1	1	702.4367
98766	3	3	5.4	2650	1	1	1	783.4576
98766	3	3	5.4	3721	1	1	1	1056.5283
64169	2	2	4	1398	1	1	1	600.3352
64169	2	2	4	1699	1	1	1	640.8873
194402	2	2	1.4	834	1	1	1	527.2763
194402	2	2	1.4	2319	1	1	1	718.3748
196643	2	2	1.9	1700	1	1	1	640.9005
196643	2	2	1.9	4217	1	1	1	1169.0843
30060	2	2	9.2	1451	1	1	1	607.2848
30060	2	2	9.2	2741	1	1	1	810.4484
28210	3	3	9.9	67	1	1	1	378.2602
28210	3	3	9.9	96	1	1	1	387.7655
28210	3	3	9.9	2526	1	1	1	753.8907
40331	2	2	6.9	1355	1	1	1	595.3239
40331	2	2	6.9	1977	1	1	1	676.8246
42230	2	2	8.9	1575	1	1	1	625.3127
42230	2	2	8.9	4366	1	1	1	815.4696
120769	3	3	4.2	1569	1	1	1	624.798
120769	3	3	4.2	2331	1	1	1	720.3583
120769	3	3	4.2	4390	1	1	1	830.4458
286993	2	2	1.1	2020	1	1	1	681.8761
286993	2	2	1.1	3610	1	1	1	1025.0566
52150	2	2	4.5	970	1	1	1	544.7927
52150	2	2	4.5	1550	1	1	1	621.8216
14095	3	2	18.5	1175	1	1	1	573.8093
14095	3	2	18.5	1258	1	0	0	583.8049
29708	4	3	9.3	508	1	1	1	479.2842
29708	4	3	9.3	804	1	1	0	523.2419
29708	4	3	9.3	1167	1	1	1	573.3022
123856	2	2	2	800	1	1	1	522.2855
123856	2	2	2	2027	1	1	1	682.4026
31671	2	2	8.4	1828	1	1	1	657.3256
31671	2	2	8.4	2408	1	1	1	733.3941
45894	2	2	7.6	1225	1	0	0	579.8288
45894	2	2	7.6	3551	1	1	1	1015.0332
64599	2	2	4.1	1280	1	1	1	586.2724
64599	2	2	4.1	2939	1	1	1	857.9054
29898	2	2	8.8	1653	1	1	1	634.8171
29898	2	2	8.8	2414	1	1	1	490.294

ot Daemon interface and database TAIRplus (Van Leene et al., 2015). Proteins and peptides headers used in the table are listed be

p < 0.01); **prot_cover**: percentage of protein sequence covered by assigned peptide matches; **pep_query**: peptide query number;
e. Whenever the top ranking peptide match appears, it is shown in red. Thus, a bold red match is the highest scoring match to a pa
sted higher in the report.); **pep_isunique**: peptide is unique to protein; **pep_exp_mz**: observed m/z value (precursor); **pep_exp_m**
protein; **pep_miss**: number of missed enzyme cleavage sites; **pep_score:** peptide ions score; **pep_ident:** peptide score identity th
; **pep_res_after**: amino acid after peptide sequence; **pep_var_mod**: any variable modifications found in the peptide; **pep_var_mo**

pep_exp_mr	pep_exp_z	pep_calc_mr	pep_delta	pep_start	pep_end	pep_miss	pep_score	pep_ident
1058.5214	2	1058.5219	-0.0005	310	318	0	62.13	35
1138.6862	2	1138.6862	-0.0001	253	262	0	60.11	28
1145.5828	2	1145.5829	-0.0001	242	251	0	61.6	38
1230.5681	2	1230.5703	-0.0022	381	390	0	70.79	34
1286.6522	2	1286.6541	-0.0019	351	362	0	45.27	37
1295.5999	2	1295.5994	0.0005	36	46	0	89.28	36
1383.693	2	1383.6931	-0.0001	163	174	0	79.8	37
1444.6409	2	1444.6405	0.0004	47	58	0	102.8	33
1445.6802	2	1445.682	-0.0018	325	336	0	71	36
1637.8412	2	1637.8388	0.0024	263	276	0	93.19	37
1678.7887	2	1678.7906	-0.0019	63	77	0	77.48	37
1695.8232	2	1695.8257	-0.0024	337	350	0	71.85	38
1777.8558	2	1777.8557	0.0002	283	297	0	73.08	38
1821.9161	2	1821.9156	0.0006	3	19	0	119.17	38
1871.8597	2	1871.8625	-0.0028	20	35	0	101.65	36
1950.881	2	1950.8815	-0.0006	363	379	0	145.27	35
1971.989	2	1971.9902	-0.0012	104	121	0	141.86	37
2100.0858	3	2100.0851	0.0006	104	122	1	65	36
2125.0502	3	2125.0521	-0.0018	1	19	1	45.09	37
2766.3134	3	2766.3139	-0.0005	217	241	0	64.24	36
2801.2931	3	2801.2946	-0.0015	78	103	1	86.87	35
3398.4946	3	3398.5	-0.0054	123	154	0	49.99	31
975.4695	2	975.4695	0	351	359	0	77.48	37
1026.5495	2	1026.5498	-0.0003	310	318	0	78.35	34
1138.6862	2	1138.6862	-0.0001	253	262	0	60.11	28
1145.5828	2	1145.5829	-0.0001	242	251	0	61.6	38
1230.5681	2	1230.5703	-0.0022	381	390	0	70.79	34
1298.6137	2	1298.6143	-0.0006	47	58	0	91.27	35
1417.6443	2	1417.6444	-0.0001	163	174	0	82.16	34
1493.6484	2	1493.649	-0.0006	325	336	0	77.19	32
1637.8412	2	1637.8388	0.0024	263	276	0	93.19	37
1678.7887	2	1678.7906	-0.0019	63	77	0	77.48	37
1695.8232	2	1695.8257	-0.0024	337	350	0	71.85	38
1821.9161	2	1821.9156	0.0006	3	19	0	119.17	38
1950.881	2	1950.8815	-0.0006	363	379	0	145.27	35
1971.989	2	1971.9902	-0.0012	104	121	0	141.86	37
2100.0858	3	2100.0851	0.0006	104	122	1	65	36
2125.0502	3	2125.0521	-0.0018	1	19	1	45.09	37
2366.1192	2	2366.1212	-0.002	277	297	0	48.54	37
2763.3137	3	2763.3143	-0.0005	217	241	0	81.66	37
2797.3339	3	2797.3361	-0.0022	78	103	1	67.22	37
3199.4635	3	3199.4669	-0.0035	20	46	0	93.67	35
1058.5214	2	1058.5219	-0.0005	310	318	0	62.13	35
1138.6862	2	1138.6862	-0.0001	253	262	0	60.11	28
1145.5828	2	1145.5829	-0.0001	242	251	0	61.6	38
1230.5681	2	1230.5703	-0.0022	381	390	0	70.79	34
1286.6522	2	1286.6541	-0.0019	351	362	0	45.27	37
1383.693	2	1383.6931	-0.0001	163	174	0	79.8	37

1430.624	2	1430.6248	-0.0009	47	58	0	88.21	32
1445.6802	2	1445.682	-0.0018	325	336	0	71	36
1637.8412	2	1637.8388	0.0024	263	276	0	93.19	37
1664.7731	2	1664.775	-0.0019	63	77	0	72.11	36
1695.8232	2	1695.8257	-0.0024	337	350	0	71.85	38
1821.9161	2	1821.9156	0.0006	3	19	0	119.17	38
1950.881	2	1950.8815	-0.0006	363	379	0	145.27	35
1971.989	2	1971.9902	-0.0012	104	121	0	141.86	37
2100.0858	3	2100.0851	0.0006	104	122	1	65	36
2125.0502	3	2125.0521	-0.0018	1	19	1	45.09	37
2766.3134	3	2766.3139	-0.0005	217	241	0	64.24	36
2813.3296	3	2813.331	-0.0015	78	103	1	86.5	36
3398.4946	3	3398.5	-0.0054	123	154	0	49.99	31
1058.5214	2	1058.5219	-0.0005	310	318	0	62.13	35
1138.6862	2	1138.6862	-0.0001	253	262	0	60.11	28
1145.5828	2	1145.5829	-0.0001	242	251	0	61.6	38
1230.5681	2	1230.5703	-0.0022	381	390	0	70.79	34
1286.6522	2	1286.6541	-0.0019	351	362	0	45.27	37
1307.6344	2	1307.6357	-0.0013	36	46	0	52.18	38
1417.6443	2	1417.6444	-0.0001	163	174	0	82.16	34
1430.624	2	1430.6248	-0.0009	47	58	0	88.21	32
1445.6802	2	1445.682	-0.0018	325	336	0	71	36
1637.8412	2	1637.8388	0.0024	263	276	0	93.19	37
1664.7731	2	1664.775	-0.0019	63	77	0	72.11	36
1695.8232	2	1695.8257	-0.0024	337	350	0	71.85	38
1898.9268	2	1898.9275	-0.0007	20	35	0	119.2	37
1950.881	2	1950.8815	-0.0006	363	379	0	145.27	35
1971.989	2	1971.9902	-0.0012	104	121	0	141.86	37
2100.0858	3	2100.0851	0.0006	104	122	1	65	36
2736.3018	3	2736.3034	-0.0015	217	241	0	40.19	37
2813.3296	3	2813.331	-0.0015	78	103	1	86.5	36
3398.4946	3	3398.5	-0.0054	123	154	0	49.99	31
1001.5496	2	1001.5505	-0.001	456	464	1	45.3	38
1214.6432	2	1214.6441	-0.0009	164	175	0	80.8	37
1227.6204	2	1227.6207	-0.0003	29	39	0	46.57	35
1293.6179	2	1293.6176	0.0003	243	252	0	45.32	37
1328.6068	2	1328.6071	-0.0003	308	317	0	72.87	35
1357.6074	2	1357.6084	-0.001	546	556	0	67.42	34
1385.7208	2	1385.7224	-0.0016	604	615	0	57.7	37
1425.7568	2	1425.7576	-0.0008	335	348	0	68.99	36
1435.7458	2	1435.746	-0.0001	352	363	0	56.99	37
1472.6779	2	1472.6783	-0.0004	40	52	0	67.58	35
1539.75	2	1539.7504	-0.0004	306	317	1	62.39	37
1565.8018	2	1565.8011	0.0008	117	130	0	91	37
1662.803	2	1662.8036	-0.0006	60	74	0	107.58	38
1674.7215	2	1674.7234	-0.0019	227	242	0	93.87	32
1679.8254	2	1679.8267	-0.0013	145	159	0	43.45	38
2559.2444	2	2559.2452	-0.0008	368	393	0	137.6	37
2587.2104	3	2587.2126	-0.0022	5	28	0	57.62	36
2657.2612	2	2657.2609	0.0003	431	453	0	102.9	37
2764.1305	3	2764.1341	-0.0036	616	646	0	83.49	27
3025.4905	3	3024.4815	1.0089	279	305	0	53.72	36
3050.6908	3	3050.6937	-0.003	394	422	0	29.47	28
949.4865	2	949.4869	-0.0004	129	137	0	42.67	37
957.512	2	957.5131	-0.0011	118	125	0	48.79	36
1016.5388	2	1016.539	-0.0002	245	253	0	40.32	38
1113.6759	2	1113.6758	0.0002	331	340	0	78.91	31
1141.5658	2	1141.5663	-0.0005	138	148	0	61.91	35

1172.6407	2	1172.6401	0.0006	244	253	1	53.88	37
1400.725	2	1400.726	-0.0009	76	89	0	110.4	37
1446.7445	2	1446.7467	-0.0022	376	388	0	89.76	38
1501.8715	2	1501.8729	-0.0014	153	167	0	98.55	30
1574.8407	2	1574.8417	-0.001	375	388	1	105.36	36
1586.6944	2	1586.6956	-0.0012	184	196	0	96.88	33
1783.7522	3	1783.7544	-0.0022	302	317	0	39.96	31
1826.9303	2	1826.9315	-0.0012	53	68	1	105.81	37
1919.9944	2	1919.988	0.0064	396	412	0	68.53	37
2026.8861	3	2026.8875	-0.0015	300	317	1	72.91	33
2058.081	2	2058.082	-0.0009	213	231	0	110.23	36
2894.4922	3	2894.4927	-0.0005	341	365	0	107.19	35
3136.6048	3	3136.6049	-0.0002	90	117	0	57.86	35
902.4199	2	902.4208	-0.0008	395	401	0	34.3	33
986.5392	2	986.5397	-0.0005	327	336	0	70.01	37
1282.4969	2	1282.4967	0.0003	312	320	0	33.05	24
1472.857	2	1472.8562	0.0008	230	243	0	93.57	31
1597.7595	2	1597.7599	-0.0004	340	352	0	67.05	37
1689.8784	2	1689.8798	-0.0014	216	229	1	74.44	37
1700.8972	2	1700.8985	-0.0013	65	79	0	90.55	37
1791.9217	2	1791.9229	-0.0012	265	280	0	89.22	38
1856.8754	2	1856.8761	-0.0006	374	390	0	104.15	37
1884.9377	2	1884.9404	-0.0027	353	370	0	69.05	37
1976.8762	2	1976.8752	0.0009	41	60	0	125.64	34
2345.0034	3	2345.0059	-0.0025	403	422	0	51.27	32
2384.1856	3	2384.1873	-0.0016	85	105	1	79.51	37
2394.1842	2	2394.1856	-0.0013	244	264	0	88.14	37
2640.2193	3	2640.22	-0.0006	281	304	0	68.27	36
3192.6229	3	3192.6238	-0.0009	125	156	0	80.96	35
902.4199	2	902.4208	-0.0008	395	401	0	34.3	33
986.5392	2	986.5397	-0.0005	327	336	0	70.01	37
1282.4969	2	1282.4967	0.0003	312	320	0	33.05	24
1472.857	2	1472.8562	0.0008	230	243	0	93.57	31
1597.7595	2	1597.7599	-0.0004	340	352	0	67.05	37
1662.8629	2	1662.8689	-0.006	216	229	1	47.44	38
1700.8972	2	1700.8985	-0.0013	65	79	0	90.55	37
1791.9217	2	1791.9229	-0.0012	265	280	0	89.22	38
1856.8754	2	1856.8761	-0.0006	374	390	0	104.15	37
1884.9377	2	1884.9404	-0.0027	353	370	0	69.05	37
1976.8762	2	1976.8752	0.0009	41	60	0	125.64	34
2345.0034	3	2345.0059	-0.0025	403	422	0	51.27	32
2384.1856	3	2384.1873	-0.0016	85	105	1	79.51	37
2394.1842	2	2394.1856	-0.0013	244	264	0	88.14	37
2640.2193	3	2640.22	-0.0006	281	304	0	68.27	36
3192.6229	3	3192.6238	-0.0009	125	156	0	80.96	35
934.5121	2	934.5124	-0.0003	129	137	0	55.37	35
957.512	2	957.5131	-0.0011	118	125	0	48.79	36
975.5501	2	975.5502	-0.0001	376	384	0	64.35	36
1016.5388	2	1016.539	-0.0002	245	253	0	40.32	38
1103.6444	2	1103.6451	-0.0007	375	384	1	46.85	33
1113.6759	2	1113.6758	0.0002	331	340	0	78.91	31
1141.5658	2	1141.5663	-0.0005	138	148	0	61.91	35
1172.6407	2	1172.6401	0.0006	244	253	1	53.88	37
1400.725	2	1400.726	-0.0009	76	89	0	110.4	37
1501.8715	2	1501.8729	-0.0014	153	167	0	98.55	30
1586.6944	2	1586.6956	-0.0012	184	196	0	96.88	33
1783.7522	3	1783.7544	-0.0022	302	317	0	39.96	31
1826.9303	2	1826.9315	-0.0012	53	68	1	105.81	37

1919.9944	2	1919.988	0.0064	396	412	0	68.53	37
2026.8861	3	2026.8875	-0.0015	300	317	1	72.91	33
2058.081	2	2058.082	-0.0009	213	231	0	110.23	36
2894.4922	3	2894.4927	-0.0005	341	365	0	107.19	35
1001.5496	2	1001.5505	-0.001	456	464	1	45.3	38
1214.6432	2	1214.6441	-0.0009	164	175	0	80.8	37
1225.5833	2	1225.5827	0.0006	81	91	0	61.65	36
1227.6204	2	1227.6207	-0.0003	29	39	0	46.57	35
1293.6179	2	1293.6176	0.0003	243	252	0	45.32	37
1294.6558	2	1294.6557	0	308	317	0	55.71	37
1307.6973	2	1307.6972	0	133	144	0	71.15	36
1357.6074	2	1357.6084	-0.001	546	556	0	67.42	34
1385.7208	2	1385.7224	-0.0016	604	615	0	57.7	37
1406.7557	2	1406.7558	-0.0001	352	363	0	73.03	35
1430.7356	2	1430.7365	-0.0009	335	348	0	110.3	37
1472.6779	2	1472.6783	-0.0004	40	52	0	67.58	35
1521.7941	3	1521.7939	0.0002	306	317	1	50.44	37
1674.7215	2	1674.7234	-0.0019	227	242	0	93.87	32
1676.817	2	1676.8192	-0.0022	60	74	0	104.05	38
1679.8254	2	1679.8267	-0.0013	145	159	0	43.45	38
2573.2578	2	2573.2609	-0.003	368	393	0	114	37
2587.2104	3	2587.2126	-0.0022	5	28	0	57.62	36
2657.2612	2	2657.2609	0.0003	431	453	0	102.9	37
2975.4764	2	2974.4812	0.9953	279	305	0	107.94	36
891.5066	2	891.5066	0.0001	508	515	0	35.54	34
932.4606	2	932.4603	0.0002	411	418	0	42.03	38
954.565	2	954.5651	0	461	468	0	35.16	31
1090.5905	2	1090.591	-0.0005	496	504	0	59.47	36
1160.5728	2	1160.5727	0.0001	290	299	0	60.19	37
1162.634	2	1162.6346	-0.0006	395	403	1	52.31	36
1198.6335	2	1198.6346	-0.0011	280	289	0	41.35	35
1332.7108	2	1332.7112	-0.0003	219	230	0	67.97	37
1381.5803	2	1381.5795	0.0008	354	364	0	63.92	32
1392.7397	2	1392.7402	-0.0005	427	439	0	37.85	36
1608.8709	2	1608.8722	-0.0013	310	324	0	72.92	35
1650.8105	2	1650.8114	-0.0009	169	183	0	59.16	38
1701.7298	2	1701.7304	-0.0006	258	273	0	113.51	31
1719.8673	2	1719.8679	-0.0005	516	530	0	113.45	38
1970.0457	2	1970.0473	-0.0015	478	495	0	68.61	35
2196.0994	2	2196.1022	-0.0028	197	218	0	88.14	37
2640.3462	3	2640.3469	-0.0007	365	386	0	90.09	36
2704.1905	3	2704.1906	-0.0001	146	168	0	54.13	33
2782.3847	3	2782.3847	-0.0001	326	353	0	60.94	37
3156.6139	3	3156.6125	0.0014	116	145	0	67.56	34
1003.4752	2	1003.4757	-0.0004	351	359	0	42.11	36
1058.5214	2	1058.5219	-0.0005	310	318	0	62.13	35
1138.6862	2	1138.6862	-0.0001	253	262	0	60.11	28
1145.5828	2	1145.5829	-0.0001	242	251	0	61.6	38
1230.5681	2	1230.5703	-0.0022	381	390	0	70.79	34
1292.6348	2	1292.6361	-0.0013	36	46	0	71.58	38
1383.693	2	1383.6931	-0.0001	163	174	0	79.8	37
1430.624	2	1430.6248	-0.0009	47	58	0	88.21	32
1445.6802	2	1445.682	-0.0018	325	336	0	71	36
1637.8412	2	1637.8388	0.0024	263	276	0	93.19	37
1695.8232	2	1695.8257	-0.0024	337	350	0	71.85	38
1955.9954	3	1955.9953	0.0001	104	121	0	58.48	37
2766.3134	3	2766.3139	-0.0005	217	241	0	64.24	36
2827.3608	3	2827.3467	0.0141	78	103	1	38.73	37

3398.4946	3	3398.5	-0.0054	123	154	0	49.99	31
1001.5496	2	1001.5505	-0.001	456	464	1	45.3	38
1110.4932	2	1110.4942	-0.001	81	90	0	57.44	34
1196.6875	2	1196.6877	-0.0002	164	175	0	78.34	33
1227.6204	2	1227.6207	-0.0003	29	39	0	46.57	35
1293.6179	2	1293.6176	0.0003	243	252	0	45.32	37
1357.6074	2	1357.6084	-0.001	546	556	0	67.42	34
1435.7458	2	1435.746	-0.0001	352	363	0	56.99	37
1472.6779	2	1472.6783	-0.0004	40	52	0	67.58	35
1662.803	2	1662.8036	-0.0006	60	74	0	107.58	38
1674.7215	2	1674.7234	-0.0019	227	242	0	93.87	32
1679.8254	2	1679.8267	-0.0013	145	159	0	43.45	38
2573.2578	2	2573.2609	-0.003	368	393	0	114	37
2587.2104	3	2587.2126	-0.0022	5	28	0	57.62	36
3149.4826	3	3148.4724	1.0102	575	601	1	49.01	36
761.443	2	761.4436	-0.0005	170	175	0	35.88	35
934.5121	2	934.5124	-0.0003	131	139	0	55.37	35
957.512	2	957.5131	-0.0011	120	127	0	48.79	36
1016.5388	2	1016.539	-0.0002	247	255	0	40.32	38
1113.6759	2	1113.6758	0.0002	333	342	0	78.91	31
1172.6407	2	1172.6401	0.0006	246	255	1	53.88	37
1400.725	2	1400.726	-0.0009	78	91	0	110.4	37
1586.6944	2	1586.6956	-0.0012	186	198	0	96.88	33
1783.7522	3	1783.7544	-0.0022	304	319	0	39.96	31
1826.9303	2	1826.9315	-0.0012	55	70	1	105.81	37
1919.9944	2	1919.988	0.0064	398	414	0	68.53	37
2026.8861	3	2026.8875	-0.0015	302	319	1	72.91	33
2894.4922	3	2894.4927	-0.0005	343	367	0	107.19	35
902.4199	2	902.4208	-0.0008	395	401	0	34.3	33
986.5392	2	986.5397	-0.0005	327	336	0	70.01	37
1282.4969	2	1282.4967	0.0003	312	320	0	33.05	24
1530.8985	2	1530.8981	0.0004	230	243	0	117.02	28
1583.7435	2	1583.7443	-0.0008	340	352	0	80.01	36
1690.8613	2	1690.8638	-0.0025	216	229	1	58.51	38
1700.8972	2	1700.8985	-0.0013	65	79	0	90.55	37
1749.9117	3	1749.9124	-0.0007	265	280	0	40.32	37
1883.8872	2	1883.887	0.0002	374	390	0	96.14	37
1884.9377	2	1884.9404	-0.0027	353	370	0	69.05	37
2345.0034	3	2345.0059	-0.0025	403	422	0	51.27	32
2384.1856	3	2384.1873	-0.0016	85	105	1	79.51	37
2408.2008	2	2408.2012	-0.0004	244	264	0	99.31	37
2694.2654	3	2694.2669	-0.0015	281	304	0	49.78	36
3192.6229	3	3192.6238	-0.0009	125	156	0	80.96	35
1247.6758	2	1247.6761	-0.0004	393	403	0	70.72	36
1248.5575	2	1248.5582	-0.0007	547	558	0	62.19	34
1255.6115	2	1255.6118	-0.0003	173	183	1	54.59	36
1293.5734	2	1293.5758	-0.0024	618	629	0	51.25	34
1326.6297	2	1326.6303	-0.0006	581	592	0	71.32	36
1360.7344	2	1360.7351	-0.0006	160	172	0	56.36	37
1432.7232	2	1432.7232	0	377	389	0	93.27	38
1476.7558	2	1476.7573	-0.0015	107	120	0	96.67	38
1812.9477	2	1812.9469	0.0009	601	617	0	144.04	36
1821.8932	2	1821.8931	0.0001	218	234	0	78.45	38
1838.8824	2	1838.8833	-0.0009	467	483	0	60.12	37
3077.4622	3	3077.4618	0.0004	264	290	1	51.47	36
773.4281	2	773.4283	-0.0002	303	310	0	45.65	38
1118.5142	2	1118.5145	-0.0003	51	59	0	36.6	35
1139.6367	2	1139.6373	-0.0006	328	337	0	41.39	33

1433.7499	2	1433.7514	-0.0015	205	219	0	71.01	38
1497.8403	2	1497.8403	0	239	252	0	122	34
1675.9657	3	1675.9661	-0.0005	70	84	1	47.87	29
1760.7784	2	1760.7794	-0.001	314	327	0	87.68	33
2032.0647	3	2032.0663	-0.0017	172	190	0	66.97	37
2171.0008	2	2171.0019	-0.001	276	295	0	136.15	36
2249.062	2	2249.0668	-0.0047	146	166	0	106.63	37
847.4914	2	847.4916	-0.0002	445	452	0	61.37	35
971.5283	2	971.5287	-0.0005	41	48	0	44.07	36
987.5545	2	987.5535	0.0009	353	361	0	61.22	37
1151.5928	2	1151.5935	-0.0007	1073	1082	0	39.58	37
1268.7092	2	1268.7088	0.0003	512	524	0	70.24	34
1275.6427	2	1275.6427	-0.0001	230	240	0	73.87	38
1405.8029	3	1405.8041	-0.0013	832	844	0	32.42	31
1467.7749	2	1467.7755	-0.0007	599	611	0	53.34	36
1498.7492	2	1498.749	0.0002	389	400	0	81.69	37
1567.8252	2	1567.8246	0.0006	273	285	0	49.86	36
1964.014	2	1964.0136	0.0004	191	209	0	116.06	37
2240.2258	2	2240.2264	-0.0005	49	70	0	131.5	32
792.4378	2	792.4382	-0.0003	219	225	0	46.14	35
815.4863	2	815.4865	-0.0002	121	127	0	40.72	35
1173.6067	2	1173.607	-0.0003	191	200	0	73.56	38
1478.772	2	1478.7729	-0.0009	147	158	1	72.03	37
1514.708	2	1514.7101	-0.002	354	368	0	114.13	36
1681.8252	2	1681.8311	-0.006	88	101	0	65.64	37
1828.9936	2	1828.9903	0.0033	312	328	0	143.5	35
1845.8476	2	1845.8493	-0.0017	159	174	0	71.23	35
2157.0753	3	2156.0724	1.0029	129	146	1	37.7	37
799.5279	2	799.528	0	64	70	1	31.2	28
975.4405	2	975.441	-0.0005	21	30	0	76.81	33
1162.5897	2	1162.5903	-0.0006	318	328	0	38.75	38
1191.5216	2	1191.523	-0.0015	199	208	0	63.71	33
1197.6977	2	1197.6982	-0.0005	31	41	1	63.23	33
1474.6673	2	1474.6688	-0.0015	362	374	0	81.3	34
1773.8881	2	1773.8897	-0.0016	241	256	0	103.28	38
1854.9245	2	1854.9251	-0.0006	218	233	0	102.35	37
2198.0658	2	2198.0678	-0.0019	294	314	0	98.76	37
3150.6331	3	3150.635	-0.0019	150	179	0	102.31	34
1094.6378	2	1094.6376	0.0003	382	390	0	39.47	31
1156.644	2	1156.6452	-0.0012	316	326	0	101.72	36
1388.6627	2	1388.6646	-0.0019	330	341	0	66.59	36
1434.6628	2	1434.6626	0.0001	113	124	1	76.55	36
1680.9401	2	1680.941	-0.0009	342	357	0	108.74	32
1767.7799	3	1767.7787	0.0011	143	157	0	50.62	34
1827.9573	2	1827.9587	-0.0013	441	456	0	64	36
1906.0435	2	1906.0445	-0.001	93	112	0	64.25	33
2134.131	2	2134.131	0	283	301	0	72.23	35
797.5128	2	797.5123	0.0005	307	313	0	41.58	27
971.5381	2	971.54	-0.0019	566	574	0	42.93	37
1028.5608	2	1028.5614	-0.0007	330	339	0	49.41	36
1037.5204	2	1037.5216	-0.0012	829	836	0	45.09	37
1049.5254	2	1049.5254	0	521	529	0	62.97	37
1115.5824	2	1115.5822	0.0002	862	870	0	40.52	37
1157.6279	2	1157.6292	-0.0013	200	209	0	59.93	37
1158.5867	2	1158.588	-0.0013	506	516	0	81.15	37
1396.7296	2	1396.731	-0.0014	236	248	0	58.46	36
1410.6442	2	1410.6416	0.0027	875	887	0	38.98	36
1475.776	2	1475.7773	-0.0013	183	196	0	63.19	38

1511.7172	2	1511.7177	-0.0005	275	287	0	64.64	36
1891.0401	2	1891.0415	-0.0014	454	470	0	104.96	33
2251.0958	3	2250.0916	1.0041	481	500	0	93.7	37
2507.3749	3	2507.3748	0.0002	136	160	0	68.93	31
919.4779	2	919.4797	-0.0018	43	52	0	69.39	38
940.502	2	940.5018	0.0002	63	70	0	37.03	34
1438.6788	2	1438.6801	-0.0012	27	42	0	123.17	37
1514.7202	2	1514.7212	-0.001	76	89	0	132.45	36
2141.0008	2	2141.0025	-0.0016	71	89	1	103.97	37
913.5594	2	913.5597	-0.0003	419	427	0	55.98	33
1006.5444	3	1006.5447	-0.0003	165	172	1	42.4	36
1024.6019	2	1024.603	-0.0011	244	254	0	75.45	31
1025.4814	2	1025.4818	-0.0004	205	212	0	37.11	35
1119.5913	2	1119.5924	-0.0011	21	30	0	47.24	37
1297.7394	2	1297.7394	0	135	146	0	82.36	33
1450.6648	2	1450.6663	-0.0015	85	96	0	62.99	35
1633.8932	3	1633.894	-0.0009	6	20	0	49.25	34
2311.1205	3	2311.1228	-0.0023	392	411	1	62.42	37
2538.3137	3	2537.3121	1.0016	255	278	1	61.29	36
2972.4141	3	2972.4107	0.0034	101	129	0	77.05	37
942.5019	2	942.5022	-0.0003	191	198	1	51	34
1069.6388	2	1069.6383	0.0005	83	92	0	56.5	31
1302.743	2	1302.7435	-0.0005	136	146	0	63.19	33
1309.6126	2	1309.6125	0.0001	270	280	0	84.73	36
1379.6382	2	1379.6391	-0.0009	290	302	0	62.46	37
1512.8302	3	1512.83	0.0002	204	216	0	71.87	35
1752.8501	2	1752.8505	-0.0003	147	161	0	81.02	37
2320.2059	3	2320.2063	-0.0004	93	113	0	80.45	36
1241.6425	2	1241.6438	-0.0013	80	90	0	46.08	35
1934.0238	2	1934.0262	-0.0023	180	197	0	114.67	36
2430.2294	2	2430.2319	-0.0024	249	271	0	95.74	37
2483.216	2	2483.2166	-0.0006	91	113	0	129.4	37
2945.4693	3	2945.4692	0.0001	219	245	0	70.51	36
960.412	2	960.4124	-0.0003	544	552	0	52.32	29
1130.4633	2	1130.4629	0.0004	584	594	0	73.61	28
1158.4945	2	1158.4942	0.0003	602	613	0	36.48	31
1186.6952	2	1186.6962	-0.001	155	165	0	45.77	33
1213.5355	2	1213.5397	-0.0042	482	493	0	41.4	33
1260.6711	2	1260.6714	-0.0003	471	481	0	39.08	38
1499.6779	2	1499.6787	-0.0008	375	387	0	68.24	35
1545.7769	2	1545.7787	-0.0019	222	236	0	101.89	38
1597.8213	3	1597.8213	0	396	410	0	40.04	36
1814.9044	2	1814.905	-0.0007	319	335	0	70.33	37
2409.1276	2	2409.1304	-0.0028	200	221	0	60.78	36
2583.3366	3	2583.3392	-0.0026	92	117	1	45.38	35
815.4863	2	815.4865	-0.0002	121	127	0	40.72	35
1173.6067	2	1173.607	-0.0003	191	200	0	73.56	38
1478.772	2	1478.7729	-0.0009	147	158	1	72.03	37
1514.708	2	1514.7101	-0.0021	354	368	0	114.13	36
1725.7677	2	1725.7702	-0.0025	219	233	0	59.35	34
1799.9293	2	1799.9305	-0.0012	46	63	0	76.65	37
2157.0753	3	2156.0724	1.0029	129	146	1	37.7	37
770.4399	2	770.4399	0	346	353	0	57.81	32
916.5745	2	916.5746	-0.0001	367	374	0	49.35	26
1206.6245	2	1206.6245	0.0001	354	366	0	61.4	38
1359.7053	2	1359.7068	-0.0015	106	116	0	66.65	38
1445.7343	2	1445.7343	0	154	165	0	74.52	38
2047.0088	2	2047.0085	0.0004	76	95	0	102.66	37

937.486	2	937.4869	-0.0009	631	640	0	48.28	36
1138.5716	2	1138.5731	-0.0015	973	981	0	76.36	36
1172.6651	2	1172.6652	-0.0001	922	931	0	42.21	36
1174.6455	2	1174.6458	-0.0003	1035	1043	0	50.55	37
1314.6911	2	1314.6892	0.0019	1259	1271	0	66.21	37
1353.6517	2	1353.6525	-0.0007	1401	1414	0	74.77	37
1872.8386	2	1872.8391	-0.0005	582	597	0	70.95	34
1955.9668	3	1954.965	1.0019	982	997	0	46.29	37
1985.9366	2	1985.9371	-0.0005	614	630	0	49.71	36
2166.9748	3	2166.9777	-0.0029	535	553	0	73.37	34
2377.2182	3	2376.2107	1.0075	953	972	0	49.86	37
856.5128	2	856.513	-0.0002	545	551	0	40.86	35
946.4974	2	946.4971	0.0003	295	303	0	45.05	39
1129.5614	2	1129.5615	-0.0001	613	622	0	53.12	35
1189.6713	2	1189.6707	0.0006	398	408	0	58.93	35
1234.5678	2	1234.5677	0.0001	552	563	0	70.53	36
1255.6115	2	1255.6118	-0.0003	178	188	1	54.59	36
1416.719	2	1416.7209	-0.0019	112	125	0	61.34	37
1432.7232	2	1432.7232	0	382	394	0	93.27	38
1612.8682	3	1612.8685	-0.0003	352	364	1	53.02	35
1820.9012	2	1820.9017	-0.0005	472	488	0	97.98	38
1192.6557	2	1192.6564	-0.0007	138	147	0	59.88	35
1216.5982	2	1216.5989	-0.0007	321	330	0	53.25	37
1279.623	2	1279.6231	-0.0001	207	217	0	77.84	37
1422.6561	2	1422.6568	-0.0007	183	193	0	81.71	36
1482.8069	2	1482.8155	-0.0086	102	116	0	88.63	34
1585.8675	2	1585.8684	-0.0009	306	320	0	63.2	36
2275.1658	2	2275.1671	-0.0012	118	137	0	45.2	37
2485.2061	3	2484.202	1.0041	148	170	0	52.16	37
1157.6435	2	1157.6445	-0.0009	206	217	0	49.17	37
1166.5305	2	1166.5316	-0.0011	279	288	1	57.5	35
1194.5514	2	1194.5517	-0.0003	371	381	0	53.27	36
1294.6348	2	1294.634	0.0008	223	234	0	43.89	37
1885.893	2	1885.8945	-0.0015	140	155	0	49.01	37
1982.9228	2	1982.9255	-0.0028	293	309	0	145.13	36
1696.7567	2	1696.754	0.0027	73	87	0	92.27	34
2149.123	2	2149.1242	-0.0011	278	296	0	125.42	36
2346.1796	3	2346.1815	-0.0019	410	432	0	77.85	37
2822.4453	3	2822.4491	-0.0038	325	349	1	63.6	36
869.5328	2	869.5334	-0.0007	236	243	0	50.18	29
914.4818	2	914.4821	-0.0003	389	396	0	40.85	35
1009.5921	2	1009.592	0.0001	417	425	0	35.96	29
1380.6499	2	1380.6521	-0.0022	608	619	0	58.44	37
1460.7631	2	1460.7623	0.0008	185	197	0	107.5	38
1578.8038	2	1578.8042	-0.0004	133	146	0	56.31	38
1750.8871	2	1750.889	-0.0018	244	259	0	105.26	38
1847.999	3	1847.9993	-0.0003	400	416	1	40.9	35
799.5279	2	799.528	0	64	70	1	31.2	28
975.4405	2	975.441	-0.0005	21	30	0	76.81	33
1162.5897	2	1162.5903	-0.0006	318	328	0	38.75	38
1197.6977	2	1197.6982	-0.0005	31	41	1	63.23	33
1789.8838	2	1789.8846	-0.0008	241	256	0	82.44	38
1968.0778	3	1967.0727	1.005	98	115	0	42.09	33
3150.6331	3	3150.635	-0.0019	150	179	0	102.31	34
1155.5351	2	1155.5342	0.0008	224	233	0	41.24	35
1266.6027	2	1266.6026	0.0001	660	669	0	50.08	37
1335.6675	2	1335.667	0.0005	157	168	0	63.2	38
1402.8589	2	1402.8588	0.0002	562	574	0	83.62	20

1715.028	2	1715.0273	0.0007	869	883	0	56.1	22
2053.0382	2	2053.0401	-0.0019	115	133	0	70.58	37
2111.0452	3	2111.0436	0.0016	368	387	0	50.24	37
982.4913	2	982.4906	0.0007	197	205	0	54.5	35
1194.5764	2	1194.5768	-0.0004	112	121	0	53.67	37
1312.6272	2	1312.6299	-0.0027	78	89	0	50.99	36
1464.7715	2	1464.7725	-0.001	95	106	0	88.83	37
1608.7982	2	1608.7995	-0.0013	216	231	0	86.32	38
1948.9156	2	1948.9162	-0.0006	232	247	0	79.38	36
1018.5044	2	1018.5043	0	805	815	0	96.54	38
1282.6154	2	1282.6162	-0.0008	543	553	0	61.19	36
1461.7622	2	1461.7616	0.0006	683	694	0	78.64	38
1835.9326	2	1835.9339	-0.0012	112	127	0	110.72	38
1232.6173	2	1232.6183	-0.001	623	633	0	49.79	38
1257.6958	2	1257.6969	-0.0011	875	886	0	46.21	36
1276.6662	2	1276.6663	-0.0001	318	330	0	43.8	37
1280.6176	2	1280.6183	-0.0007	331	341	0	57.46	37
1554.8617	2	1554.8617	0	763	777	0	98.89	34
1558.7616	2	1558.7587	0.0029	474	488	0	87.85	37
1287.6603	2	1287.6605	-0.0002	95	106	0	66.08	38
1422.6549	2	1422.6627	-0.0078	28	40	0	71.89	36
1941.9831	2	1941.9836	-0.0005	11	27	0	119.16	37
1212.6713	2	1212.6714	-0.0001	296	306	0	98.76	35
1587.7841	2	1587.7852	-0.0011	228	243	0	92.23	37
1767.7799	3	1767.7787	0.0011	131	145	0	50.62	34
2128.0662	2	2128.0609	0.0053	193	210	0	58.7	37
1180.7187	2	1180.718	0.0008	174	184	0	80.93	26
1232.6881	2	1232.6877	0.0004	397	408	0	70.8	36
1881.0971	2	1881.0975	-0.0004	147	164	0	103.88	27
1159.5815	2	1159.5833	-0.0018	513	524	0	41.52	38
1198.6563	2	1198.6557	0.0005	286	296	0	67.09	35
1279.7599	2	1279.7612	-0.0013	161	173	0	69.32	28
1699.891	2	1699.8927	-0.0017	459	475	0	108.19	37
1899.0282	2	1899.0313	-0.0031	426	444	0	60.42	35
1192.6557	2	1192.6564	-0.0007	138	147	0	59.88	35
1216.5982	2	1216.5989	-0.0007	321	330	0	53.25	37
1422.6561	2	1422.6568	-0.0007	183	193	0	81.71	36
1468.7989	2	1468.7998	-0.0009	102	116	0	83.43	35
2485.2061	3	2484.202	1.0041	148	170	0	52.16	37
1746.8883	2	1746.89	-0.0017	32	48	0	138.86	38
1913.9578	2	1913.9597	-0.0019	181	196	0	59.95	37
2460.2726	2	2460.2723	0.0004	6	26	0	66.8	36
997.5556	2	997.5556	0	140	148	0	44.7	33
1192.6101	3	1192.6102	0	48	58	0	49.49	38
1402.7931	2	1402.7932	-0.0001	150	162	0	95.84	34
1630.8227	2	1630.8236	-0.0009	171	185	0	112.56	38
939.5754	2	939.5753	0.0001	125	132	0	60.75	28
1864.0405	2	1864.0418	-0.0013	68	84	0	124.75	31
2080.9256	3	2080.9273	-0.0017	107	124	0	41.36	34
2192.0212	2	2192.0242	-0.0029	140	159	0	60.28	36
1159.5815	2	1159.5833	-0.0018	509	520	0	41.52	38
1228.6912	2	1228.6914	-0.0002	282	292	0	56.66	36
1279.7599	2	1279.7612	-0.0013	157	169	0	69.32	28
1699.891	2	1699.8927	-0.0017	455	471	0	108.19	37
1899.0282	2	1899.0313	-0.0031	422	440	0	60.42	35
1451.683	2	1451.6834	-0.0004	244	255	0	57.99	37
1517.6633	2	1517.6668	-0.0035	593	606	0	99.8	32
1798.9241	2	1798.9254	-0.0013	771	786	0	56.78	37

2256.1944	2	2256.1961	-0.0017	33	55	0	76.42	35
1158.4945	2	1158.4942	0.0003	596	607	0	36.48	31
1186.6952	2	1186.6962	-0.001	167	177	0	45.77	33
1273.666	2	1273.6666	-0.0006	483	493	0	43.44	38
1483.6836	3	1483.6838	-0.0002	387	399	0	48.11	36
1536.8342	3	1536.8372	-0.003	457	470	1	45.3	35
1795.7386	2	1795.7398	-0.0012	569	588	0	96.33	28
2409.1276	2	2409.1304	-0.0028	212	233	0	60.78	36
1495.8297	2	1495.8286	0.0011	705	717	0	96.18	32
1543.783	2	1543.7842	-0.0012	1120	1133	0	79.94	37
1617.8425	2	1617.8403	0.0023	992	1006	0	54.88	37
1912.1393	2	1912.1397	-0.0004	1025	1041	0	55.29	22
1236.6453	2	1236.6462	-0.001	241	252	0	65.44	37
1750.8566	2	1750.856	0.0006	311	325	0	126.69	37
1300.6441	2	1300.6445	-0.0004	97	108	0	61.88	36
1542.8294	3	1542.8307	-0.0013	186	198	0	39.46	37
2044.0952	2	2044.0953	0	707	724	0	117.29	35
770.4399	2	770.4399	0	350	357	0	57.81	32
916.5745	2	916.5746	-0.0001	371	378	0	49.35	26
950.4815	2	950.4821	-0.0006	246	254	0	50.78	38
1227.6136	2	1227.6136	0	330	340	0	56.3	36
1359.7053	2	1359.7068	-0.0015	110	120	0	66.65	38
1445.7343	2	1445.7343	0	158	169	0	74.52	38
897.4917	2	897.492	-0.0003	24	31	0	52.01	33
1169.5964	2	1169.5968	-0.0004	178	187	0	52.22	35
1581.8462	2	1581.8475	-0.0013	75	89	0	63.94	35
1791.8932	3	1791.8944	-0.0013	97	113	0	46.38	38
1864.9336	2	1864.934	-0.0004	3	19	0	103.35	37
709.3872	2	709.3871	0.0001	171	176	0	38.56	32
884.4714	2	884.4716	-0.0001	237	245	0	36.92	34
1277.618	2	1277.6187	-0.0006	348	358	0	76.06	38
2201.0918	2	2201.0892	0.0026	314	332	0	91.18	37
3372.5701	3	3372.5721	-0.0021	193	222	0	66.42	35
1424.7668	2	1424.7664	0.0004	309	321	0	73.35	35
1491.7665	2	1491.7681	-0.0017	338	351	0	110.68	38
1863.9366	2	1863.9367	0	322	337	0	71.31	38
1025.5875	2	1025.5869	0.0006	424	433	0	32.62	32
1249.6302	2	1249.6302	0	277	287	0	61.83	38
2307.1472	2	2307.1495	-0.0023	672	693	0	150.51	38
1770.0259	2	1770.0251	0.0008	296	312	0	105.45	28
1924.0191	2	1924.0193	-0.0002	221	236	0	98.37	35
1038.6081	2	1038.6073	0.0008	64	72	0	38.14	29
1247.6256	2	1247.6258	-0.0003	73	83	0	47.46	39
1322.6568	2	1322.6605	-0.0038	429	439	0	66.36	38
2319.1918	2	2319.1933	-0.0014	245	265	0	37.12	36
2652.3997	3	2651.3919	1.0078	319	341	0	99.23	34
1419.7724	2	1419.7722	0.0003	2674	2686	0	85.11	36
1540.8404	2	1540.8395	0.0009	4372	4384	0	43.08	34
1618.8927	2	1618.893	-0.0004	3071	3085	0	79.99	34
2053.0036	2	2053.0019	0.0017	5353	5368	0	38.59	38
2790.3814	3	2790.3824	-0.001	2150	2173	0	37.56	37
977.5178	2	977.5182	-0.0004	37	44	0	54.88	37
1530.7175	2	1530.7161	0.0014	86	99	0	129.48	36
3108.5146	3	3107.5155	0.9991	45	72	0	50.54	37
999.5698	2	999.5713	-0.0015	268	276	0	46.79	36
1270.6985	2	1270.6993	-0.0008	93	104	0	66.9	36
1284.7111	2	1284.7112	-0.0001	177	188	0	49.89	35
1397.6681	3	1397.6688	-0.0007	165	176	0	43.86	36

1414.7559	2	1414.7569	-0.0009	254	267	0	97.59	37
1559.804	2	1559.8042	-0.0002	124	138	0	102.96	38
2375.1078	2	2375.107	0.0008	139	160	0	96.19	36
1157.6435	2	1157.6445	-0.0009	175	186	0	49.17	37
1161.579	2	1161.5778	0.0012	340	350	0	74.65	38
1275.6834	2	1275.6823	0.0011	192	203	0	51.1	37
1397.6414	2	1397.6431	-0.0018	351	362	0	49.48	35
2222.2021	3	2222.2019	0.0003	55	74	1	76.47	33
1048.5194	2	1048.5189	0.0005	314	322	0	43.18	38
1173.6273	2	1173.6354	-0.0081	379	389	0	67.37	38
1225.7027	2	1225.703	-0.0003	469	480	0	63.72	32
1367.6208	2	1367.6214	-0.0005	323	333	0	40.64	35
1752.9556	2	1752.9563	-0.0007	363	378	0	73.53	33
1410.8332	2	1410.8334	-0.0002	152	164	0	59.05	30
2989.6577	3	2989.66	-0.0023	67	94	0	50.99	28
3173.6371	3	3173.6391	-0.002	17	44	0	71.09	34
3549.9073	3	3549.9083	-0.0011	115	148	0	43.26	30
1560.7162	2	1560.7168	-0.0006	168	181	0	46.06	35
1656.7359	2	1656.738	-0.002	61	75	0	58.08	34
1703.8829	2	1703.8842	-0.0014	79	93	0	113.52	38
1188.6346	2	1188.635	-0.0004	182	194	0	105.78	38
1351.6351	2	1351.6368	-0.0017	331	342	0	70.38	37
1822.9434	2	1822.9465	-0.0031	43	59	0	50.81	37
1361.7381	2	1361.7377	0.0004	1194	1205	0	61.81	36
1760.9491	2	1760.9495	-0.0004	1939	1953	0	38.87	35
2048.0976	2	2048.0983	-0.0006	957	974	0	73.54	35
2193.2256	2	2193.2256	0	440	459	0	40.76	30
2239.1672	3	2239.1696	-0.0024	2495	2515	0	50.05	36
713.4434	2	713.4435	-0.0001	430	435	0	31.76	30
1255.6882	2	1255.6884	-0.0002	88	99	0	58.77	35
1290.6351	2	1290.6303	0.0048	34	45	0	51.61	38
1526.7921	2	1526.794	-0.0019	358	371	0	84.63	36
1582.7832	3	1582.7839	-0.0006	441	454	1	57.32	37
989.5546	2	989.5546	0	693	700	0	55.33	36
1041.4869	2	1041.488	-0.001	364	372	0	37.09	34
1255.6446	2	1255.6448	-0.0002	589	598	0	68.2	35
1664.8422	2	1664.841	0.0013	677	692	0	85.21	37
794.4285	2	794.4286	-0.0001	263	269	0	46.29	34
936.5505	2	936.5505	0	162	169	0	45.48	30
982.5333	2	982.5349	-0.0015	11	19	0	41.29	34
1032.5572	2	1032.5564	0.0008	344	352	0	50.1	37
1251.5808	2	1251.5812	-0.0004	195	204	0	44.29	36
1319.662	2	1319.6622	-0.0002	362	373	0	69.04	38
1595.6885	3	1595.6886	-0.0001	291	304	0	43.19	33
815.4862	2	815.4865	-0.0003	105	111	0	39.52	35
1157.6435	2	1157.6445	-0.0009	191	202	0	49.17	37
2012.9743	3	2012.9771	-0.0028	360	378	0	47.79	37
2028.0542	2	2028.0527	0.0015	246	264	0	58.87	36
1378.7086	2	1378.7092	-0.0006	376	389	0	97.71	38
2156.9906	2	2156.9862	0.0044	356	375	0	78.23	36
1425.7531	2	1425.7576	-0.0045	132	144	0	65.87	36
1759.8941	2	1759.8952	-0.0011	9	25	0	108.12	37
948.5381	2	948.5392	-0.0012	197	205	0	67.18	35
1218.6491	2	1218.6496	-0.0004	112	121	0	53.71	37
1407.7507	2	1407.7511	-0.0004	95	106	0	90.7	36
1196.5819	2	1196.5826	-0.0007	138	148	0	74.01	36
1534.7214	2	1534.7223	-0.0009	469	483	0	101.27	37
1729.8299	2	1729.8271	0.0028	517	532	0	63.95	37

2165.0919	3	2165.0926	-0.0007	427	446	0	42.67	37
2641.3365	3	2641.3347	0.0017	131	154	0	65.01	37
904.4432	2	904.4436	-0.0004	290	297	0	49.35	37
1033.522	2	1033.5226	-0.0006	344	352	0	55.05	39
1443.6504	3	1443.6491	0.0013	63	76	0	41.2	34
1928.7969	2	1928.7986	-0.0017	385	400	0	86.1	29
1212.5545	2	1212.5557	-0.0012	153	162	0	70.86	35
1618.8839	2	1618.8831	0.0008	200	213	0	80.97	35
2056.9606	2	2056.9623	-0.0016	244	262	1	46.01	36
1038.5773	2	1038.5783	-0.001	26	34	0	40.26	33
1059.5503	2	1059.5502	0.0001	79	86	0	39.6	38
2100.0506	2	2100.0521	-0.0014	56	75	0	94.49	37
992.5467	2	992.5477	-0.001	319	327	0	59.44	36
1576.7844	2	1576.7847	-0.0003	248	260	0	87.81	38
1014.5333	2	1014.5346	-0.0013	998	1007	0	43.9	35
1434.7342	2	1434.7354	-0.0012	1132	1144	0	85.84	38
2039.1092	2	2039.1092	0.0001	520	536	0	42.9	34
2660.4327	3	2660.4326	0	414	437	0	44.15	32
1264.5776	2	1264.5791	-0.0015	174	184	0	38.02	36
1944.032	2	1944.0316	0.0004	2070	2087	0	114.71	36
1180.692	2	1180.6928	-0.0007	129	139	0	65.64	30
1549.7548	2	1549.7559	-0.001	115	128	0	87.69	38
1157.6435	2	1157.6445	-0.0009	197	208	0	49.17	37
1478.8566	3	1478.8569	-0.0003	311	323	1	34.82	30
1937.9356	2	1937.9404	-0.0048	285	301	0	92.36	37
2086.9971	3	2086.9993	-0.0022	252	270	0	39.81	37
1088.6229	2	1088.623	-0.0001	118	127	0	62.92	34
1102.6053	2	1102.6056	-0.0003	20	30	0	51.97	38
2106.0856	3	2105.0827	1.0029	110	127	1	68.74	37
1256.7324	2	1256.734	-0.0016	84	95	0	88.51	33
1383.7036	2	1383.7034	0.0002	26	37	0	56.71	36
1259.676	2	1259.6761	-0.0001	468	478	0	59.44	37
1329.707	2	1329.7075	-0.0004	234	246	0	91.73	38
980.5109	2	980.5113	-0.0005	67	74	0	49.91	35
1357.7191	2	1357.7201	-0.001	19	31	0	53.49	38
1531.8227	2	1531.8246	-0.0019	38	51	0	74.5	37
1448.7005	3	1448.7008	-0.0003	221	233	0	47.41	38
2127.0212	2	2126.0168	1.0045	17	36	0	90.28	37
1340.7565	2	1340.7565	0.0001	511	523	0	76.49	34
1630.8684	2	1630.8679	0.0005	227	242	0	61.55	36
1673.8921	3	1673.893	-0.0009	21	33	0	52.19	37
2480.328	3	2480.3274	0.0006	88	110	0	66.37	33
1266.5685	2	1266.5696	-0.0012	136	147	0	56.87	35
2499.3674	2	2499.3684	-0.0009	533	557	0	71.14	31
1154.6399	2	1154.6408	-0.0008	192	203	0	55.89	33
1255.6115	2	1255.6118	-0.0003	159	169	1	54.59	36
1493.7148	2	1493.7151	-0.0002	68	80	0	60.56	37
961.5229	2	961.5233	-0.0004	322	329	0	71.37	38
1422.6083	3	1422.6099	-0.0016	231	241	0	42.36	31
2390.2337	3	2389.2278	1.0059	61	83	0	53.83	36
905.4974	2	905.4971	0.0003	522	529	0	40.9	37
1703.9814	2	1703.9821	-0.0007	322	336	0	94.98	29
764.4179	2	764.4181	-0.0002	281	287	0	36.59	34
1205.5994	2	1205.604	-0.0046	61	70	0	53	38
1452.7466	2	1452.7474	-0.0008	238	252	0	81.92	37
1159.6196	2	1159.6197	-0.0001	50	61	0	74.53	38
1165.5967	2	1165.5979	-0.0012	66	76	0	64.6	37
1089.566	2	1089.5666	-0.0006	172	181	0	53.67	39

1705.7753	3	1705.7768	-0.0016	493	507	0	81.45	35
747.4281	2	747.4279	0.0002	363	368	0	40.87	36
1736.8393	2	1736.8403	-0.001	291	305	0	95.45	37
1165.5985	2	1165.5979	0.0006	40	50	0	90.86	37
3196.6105	3	3196.6099	0.0006	236	265	0	42.89	36
943.534	2	943.5338	0.0002	246	253	0	42.7	37
1224.7257	2	1224.7264	-0.0007	40	49	0	31.56	31
1365.6704	2	1365.6711	-0.0008	175	187	0	74.8	38
1467.8591	3	1467.8595	-0.0004	38	49	1	43.95	29
1180.5724	2	1180.5724	0	410	420	0	75.26	37
1287.7074	2	1287.7074	0	437	447	0	50.59	37
1535.9537	2	1535.9538	-0.0001	559	572	0	35.12	20
922.4984	2	922.4984	0	204	211	0	41.61	35
1518.7834	2	1518.7831	0.0004	240	252	0	90.47	38
956.5538	2	956.5542	-0.0005	126	133	0	38.17	34
1144.5901	2	1144.591	-0.0009	134	142	0	58.46	37
2882.4284	2	2882.4284	0	94	120	0	66.03	37
1003.5815	2	1003.5814	0.0001	471	479	0	62.2	34
1880.9336	2	1880.9342	-0.0006	445	460	0	64.25	37
1682.9451	2	1682.9454	-0.0003	133	147	0	80.64	32
2322.2789	3	2322.2795	-0.0006	133	153	1	42.83	31
1176.6134	2	1176.6139	-0.0005	812	821	0	63.01	38
1483.9014	2	1483.9014	0	433	446	0	47.38	25
1857.9285	3	1857.9295	-0.001	540	555	0	42.59	37
939.5393	2	939.5389	0.0004	286	293	0	37	30
1267.604	2	1267.6044	-0.0005	51	61	0	49.11	37
1308.7432	2	1308.7442	-0.001	146	157	0	57.98	32
1948.8583	2	1948.8578	0.0005	396	411	0	34.04	33
1294.8336	2	1294.8336	0	618	629	0	73.12	20
1942.0889	3	1942.0887	0.0001	225	241	1	40.87	32
1056.6295	2	1056.6292	0.0003	57	67	0	78.46	34
1253.687	2	1253.688	-0.001	174	185	0	40.08	34
1239.6241	2	1239.6248	-0.0006	206	215	0	66.89	37
1943.9794	2	1943.9808	-0.0014	292	310	0	45.53	37
949.5224	2	949.5233	-0.0009	406	413	0	38.72	35
1547.8809	2	1547.881	-0.0002	139	152	0	68.52	31
1960.9664	3	1960.9676	-0.0013	318	333	0	39.34	38
1067.4954	2	1067.4957	-0.0004	112	120	0	43.63	35
2851.4931	3	2851.4967	-0.0037	227	253	0	69.1	34
1064.5907	2	1064.5906	0.0001	15	23	0	55.16	34
3020.2783	3	3020.2785	-0.0002	81	111	0	51.79	30
1042.5549	2	1042.556	-0.001	668	676	0	45.27	35
1340.6612	2	1340.6612	0	164	174	0	67.22	36
1368.6231	2	1368.6245	-0.0013	220	230	0	38.8	35
1613.9063	2	1613.9062	0.0001	428	441	0	67.66	33
1064.5907	2	1064.5906	0.0001	15	23	0	55.16	34
1323.6898	2	1323.6922	-0.0024	128	139	0	52.77	36
1194.6386	2	1194.6397	-0.0011	302	312	0	41.36	35
1855.0551	2	1854.0502	1.0049	437	453	0	46.77	30
3201.6493	3	3201.6517	-0.0025	405	436	0	44.54	35
1145.603	2	1145.604	-0.0011	50	61	0	47.81	38
1165.5967	2	1165.5979	-0.0012	66	76	0	64.6	37
1053.5583	2	1053.5567	0.0016	106	116	0	57.58	35
1442.7242	2	1442.7253	-0.0011	128	140	0	49.17	37
754.5063	2	754.5065	-0.0002	102	107	0	46.55	20
773.5165	2	773.5163	0.0002	83	88	0	41.74	22
1382.6868	2	1382.6864	0.0004	628	640	0	54.77	37
2217.2995	3	2216.3031	0.9964	378	398	0	39.52	24

822.439	2	822.4388	0.0002	730	736	0	42.16	36
1649.9963	2	1649.9967	-0.0004	21	35	0	49.6	22
1031.6124	2	1031.6128	-0.0004	482	490	0	38.75	32
1533.855	2	1533.8555	-0.0005	542	554	0	50.88	34
1071.6435	2	1071.6441	-0.0005	934	943	0	48.02	34
1612.8563	3	1612.8573	-0.001	646	659	0	39.52	36
1012.5667	2	1012.5665	0.0002	761	770	0	48.93	33
1703.9142	2	1703.9128	0.0015	549	564	0	38.49	36
1292.6615	2	1292.6612	0.0003	247	257	0	44.04	38
1367.6208	2	1367.6214	-0.0006	258	268	0	40.64	35
1132.5077	2	1132.5084	-0.0007	300	309	0	35	33
1239.6824	2	1239.6823	0.0001	203	213	0	44.19	34
949.5223	2	949.5233	-0.001	92	99	0	39.87	35
1062.6227	2	1062.6226	0.0001	60	68	0	34.25	32
1074.5664	2	1074.5669	-0.0005	181	190	1	42.13	39
1352.8276	2	1352.8279	-0.0002	226	237	0	30.12	26

pep_exp_mr	pep_exp_z	pep_calc_mr	pep_delta	pep_start	pep_end	pep_miss	pep_score	pep_ident
975.4683	2	975.4695	-0.0012	351	359	0	77.42	37
1026.5496	2	1026.5498	-0.0002	310	318	0	78.05	34
1076.5257	3	1076.525	0.0007	155	162	1	39.09	38
1138.686	2	1138.6862	-0.0002	253	262	0	63.43	28
1145.5827	2	1145.5829	-0.0002	242	251	0	68.26	38
1214.5771	2	1214.5754	0.0017	381	390	0	75.4	35
1266.7794	2	1266.7812	-0.0018	252	262	1	47.16	26
1298.6145	2	1298.6143	0.0003	47	58	0	70.2	36
1401.6498	2	1401.6495	0.0003	163	174	0	90.05	35
1637.8394	2	1637.8388	0.0006	263	276	0	95.76	37
1678.7882	2	1678.7906	-0.0024	63	77	0	67.01	37
1695.8238	2	1695.8257	-0.0019	337	350	0	77.72	37
1821.9147	2	1821.9156	-0.0009	3	19	0	95.95	38
1950.8803	2	1950.8815	-0.0013	363	379	0	146.15	35
1971.9875	2	1971.9902	-0.0026	104	121	0	161.7	37
2100.0837	3	2100.0851	-0.0015	104	122	1	71.63	37
2125.0505	3	2125.0521	-0.0016	1	19	1	42.32	37
2366.1186	2	2366.1212	-0.0026	277	297	0	76.95	36
2763.3142	3	2763.3143	0	217	241	0	107.1	37
2797.3393	3	2797.3361	0.0032	78	103	1	57.55	37
3199.4647	4	3199.4669	-0.0022	20	46	0	49.75	35
3325.4983	3	3325.5014	-0.0031	123	154	0	63.29	34
1058.5216	2	1058.5219	-0.0003	310	318	0	57.26	35
1076.5257	3	1076.525	0.0007	155	162	1	39.09	38
1138.686	2	1138.6862	-0.0002	253	262	0	63.43	28
1145.5827	2	1145.5829	-0.0002	242	251	0	68.26	38
1214.5771	2	1214.5754	0.0017	381	390	0	75.4	35
1266.7794	2	1266.7812	-0.0018	252	262	1	47.16	26
1286.6513	2	1286.6541	-0.0027	351	362	0	52.13	37
1295.5995	2	1295.5994	0.0002	36	46	0	89.3	36
1415.6827	2	1415.6829	-0.0002	163	174	0	81.16	36
1444.6386	2	1444.6405	-0.0019	47	58	0	102.66	33
1461.676	2	1461.6769	-0.0009	325	336	0	79.58	36
1637.8394	2	1637.8388	0.0006	263	276	0	95.76	37
1678.7882	2	1678.7906	-0.0024	63	77	0	67.01	37
1695.8238	2	1695.8257	-0.0019	337	350	0	77.72	37
1777.8546	2	1777.8557	-0.001	283	297	0	55.34	38
1821.9147	2	1821.9156	-0.0009	3	19	0	95.95	38
1871.8621	2	1871.8625	-0.0004	20	35	0	65.58	36
1950.8803	2	1950.8815	-0.0013	363	379	0	146.15	35
1971.9875	2	1971.9902	-0.0026	104	121	0	161.7	37

2100.0837	3	2100.0851	-0.0015	104	122	1	71.63	37
2125.0505	3	2125.0521	-0.0016	1	19	1	42.32	37
2766.3114	3	2766.3139	-0.0025	217	241	0	62.67	36
2801.2921	3	2801.2946	-0.0025	78	103	1	78.69	35
1058.5216	2	1058.5219	-0.0003	310	318	0	57.26	35
1076.5257	3	1076.525	0.0007	155	162	1	39.09	38
1138.686	2	1138.6862	-0.0002	253	262	0	63.43	28
1145.5827	2	1145.5829	-0.0002	242	251	0	68.26	38
1214.5771	2	1214.5754	0.0017	381	390	0	75.4	35
1266.7794	2	1266.7812	-0.0018	252	262	1	47.16	26
1286.6513	2	1286.6541	-0.0027	351	362	0	52.13	37
1307.6342	2	1307.6357	-0.0016	36	46	0	72.19	38
1401.6498	2	1401.6495	0.0003	163	174	0	90.05	35
1430.6243	2	1430.6248	-0.0006	47	58	0	81.01	32
1461.676	2	1461.6769	-0.0009	325	336	0	79.58	36
1637.8394	2	1637.8388	0.0006	263	276	0	95.76	37
1664.7729	2	1664.775	-0.002	63	77	0	68.28	37
1695.8238	2	1695.8257	-0.0019	337	350	0	77.72	37
1788.8722	2	1788.8716	0.0005	283	297	0	56.73	38
1898.9273	2	1898.9275	-0.0002	20	35	0	115.29	37
1950.8803	2	1950.8815	-0.0013	363	379	0	146.15	35
1971.9875	2	1971.9902	-0.0026	104	121	0	161.7	37
2100.0837	3	2100.0851	-0.0015	104	122	1	71.63	37
2736.3031	3	2736.3034	-0.0002	217	241	0	54.75	37
2813.327	3	2813.331	-0.004	78	103	1	101.02	36
1026.5496	2	1026.5498	-0.0002	310	318	0	78.05	34
1076.5257	3	1076.525	0.0007	155	162	1	39.09	38
1138.686	2	1138.6862	-0.0002	253	262	0	63.43	28
1145.5827	2	1145.5829	-0.0002	242	251	0	68.26	38
1214.5771	2	1214.5754	0.0017	381	390	0	75.4	35
1266.7794	2	1266.7812	-0.0018	252	262	1	47.16	26
1297.6297	2	1297.6302	-0.0005	47	58	0	54.33	36
1401.6498	2	1401.6495	0.0003	163	174	0	90.05	35
1637.8394	2	1637.8388	0.0006	263	276	0	95.76	37
1678.7882	2	1678.7906	-0.0024	63	77	0	67.01	37
1695.8238	2	1695.8257	-0.0019	337	350	0	77.72	37
1821.9147	2	1821.9156	-0.0009	3	19	0	95.95	38
1950.8803	2	1950.8815	-0.0013	363	379	0	146.15	35
1971.9875	2	1971.9902	-0.0026	104	121	0	161.7	37
2100.0837	3	2100.0851	-0.0015	104	122	1	71.63	37
2125.0505	3	2125.0521	-0.0016	1	19	1	42.32	37
2366.1186	2	2366.1212	-0.0026	277	297	0	76.95	36
2763.3142	3	2763.3143	0	217	241	0	107.1	37
2797.3393	3	2797.3361	0.0032	78	103	1	57.55	37
902.4203	2	902.4208	-0.0004	395	401	0	37.75	33
986.5402	2	986.5397	0.0005	327	336	0	65.58	37
1131.5592	2	1131.5594	-0.0002	113	121	0	40.62	37
1282.4963	2	1282.4967	-0.0003	312	320	0	45.17	24
1395.6859	2	1395.6857	0.0003	391	401	1	41.06	37
1395.7505	3	1395.751	-0.0005	85	96	0	38.04	35
1472.8563	2	1472.8562	0.0001	230	243	0	94.16	30
1597.7591	2	1597.7599	-0.0008	340	352	0	87.96	37
1662.8664	2	1662.8689	-0.0025	216	229	1	70.26	37
1700.8967	2	1700.8985	-0.0018	65	79	0	91.09	37
1791.9206	2	1791.9229	-0.0023	265	280	0	107.65	38
1856.8747	2	1856.8761	-0.0014	374	390	0	115.04	37
1884.9378	2	1884.9404	-0.0026	353	370	0	73.31	37
1976.8742	2	1976.8752	-0.0011	41	60	0	115.48	33

2345.0006	2	2345.0059	-0.0052	403	422	0	64.29	31
2384.1867	3	2384.1873	-0.0006	85	105	1	82.13	37
2394.1838	2	2394.1856	-0.0017	244	264	0	128.06	38
2640.2176	2	2640.22	-0.0023	281	304	0	84.91	36
3320.7187	3	3320.7187	-0.0001	124	156	1	118.55	34
5377.6621	4	5377.6704	-0.0083	167	214	0	44.04	31
902.4203	2	902.4208	-0.0004	395	401	0	37.75	33
986.5402	2	986.5397	0.0005	327	336	0	65.58	37
1131.5592	2	1131.5594	-0.0002	113	121	0	40.62	37
1282.4963	2	1282.4967	-0.0003	312	320	0	45.17	24
1395.6859	2	1395.6857	0.0003	391	401	1	41.06	37
1395.7505	3	1395.751	-0.0005	85	96	0	38.04	35
1472.8563	2	1472.8562	0.0001	230	243	0	94.16	30
1597.7591	2	1597.7599	-0.0008	340	352	0	87.96	37
1689.876	2	1689.8798	-0.0038	216	229	1	71.61	37
1700.8967	2	1700.8985	-0.0018	65	79	0	91.09	37
1791.9206	2	1791.9229	-0.0023	265	280	0	107.65	38
1856.8747	2	1856.8761	-0.0014	374	390	0	115.04	37
1884.9378	2	1884.9404	-0.0026	353	370	0	73.31	37
1976.8742	2	1976.8752	-0.0011	41	60	0	115.48	33
2345.0006	2	2345.0059	-0.0052	403	422	0	64.29	31
2384.1867	3	2384.1873	-0.0006	85	105	1	82.13	37
2394.1838	2	2394.1856	-0.0017	244	264	0	128.06	38
2640.2176	2	2640.22	-0.0023	281	304	0	84.91	36
3320.7187	3	3320.7187	-0.0001	124	156	1	118.55	34
5377.6621	4	5377.6704	-0.0083	167	214	0	44.04	31
949.4863	2	949.4869	-0.0006	129	137	0	44.68	37
957.5128	2	957.5131	-0.0002	118	125	0	59.11	35
1003.5345	2	1003.5338	0.0007	290	297	1	43.74	38
1016.5388	2	1016.539	-0.0002	245	253	0	50.57	38
1046.5076	2	1046.5073	0.0004	53	61	0	40.23	37
1113.6757	2	1113.6758	-0.0001	331	340	0	80.17	31
1141.5657	2	1141.5663	-0.0006	138	148	0	62.07	35
1172.6401	2	1172.6401	0	244	253	1	58.44	38
1400.7246	2	1400.726	-0.0014	76	89	0	113.04	37
1446.744	2	1446.7467	-0.0027	376	388	0	93.79	38
1501.8695	2	1501.8729	-0.0035	153	167	0	107.66	31
1574.8399	2	1574.8417	-0.0017	375	388	1	124.57	37
1586.6943	2	1586.6956	-0.0013	184	196	0	105.64	33
1588.8505	2	1588.8501	0.0004	200	212	0	54.43	36
1826.9299	2	1826.9315	-0.0016	53	68	1	112.17	37
1919.9959	2	1919.988	0.0079	396	412	0	54.79	37
1921.0328	2	1921.0349	-0.0021	197	212	1	56.1	35
2074.076	2	2074.0769	-0.0008	213	231	0	112.02	37
2698.3882	2	2698.3888	-0.0005	266	288	0	96.87	35
2894.4936	2	2894.4927	0.001	341	365	0	86.39	35
3136.6072	2	3136.6049	0.0023	90	117	0	88.86	35
934.5115	2	934.5124	-0.0009	129	137	0	58.86	35
957.5128	2	957.5131	-0.0002	118	125	0	59.11	35
975.5478	2	975.5502	-0.0023	376	384	0	61.73	36
1003.5345	2	1003.5338	0.0007	290	297	1	43.74	38
1016.5388	2	1016.539	-0.0002	245	253	0	50.57	38
1046.5076	2	1046.5073	0.0004	53	61	0	40.23	37
1103.6447	2	1103.6451	-0.0005	375	384	1	45.52	33
1113.6757	2	1113.6758	-0.0001	331	340	0	80.17	31
1141.5657	2	1141.5663	-0.0006	138	148	0	62.07	35
1172.6401	2	1172.6401	0	244	253	1	58.44	38
1400.7246	2	1400.726	-0.0014	76	89	0	113.04	37

1501.8695	2	1501.8729	-0.0035	153	167	0	107.66	31
1586.6943	2	1586.6956	-0.0013	184	196	0	105.64	33
1588.8505	2	1588.8501	0.0004	200	212	0	54.43	36
1826.9299	2	1826.9315	-0.0016	53	68	1	112.17	37
1919.9959	2	1919.988	0.0079	396	412	0	54.79	37
1921.0328	2	1921.0349	-0.0021	197	212	1	56.1	35
2074.076	2	2074.0769	-0.0008	213	231	0	112.02	37
2698.3882	2	2698.3888	-0.0005	266	288	0	96.87	35
2894.4936	2	2894.4927	0.001	341	365	0	86.39	35
3918.7588	3	3917.7942	0.9646	20	52	0	45.16	32
1227.6194	2	1227.6207	-0.0013	29	39	0	73.88	36
1230.6384	2	1230.6391	-0.0006	164	175	0	68.57	38
1293.6173	2	1293.6176	-0.0003	243	252	0	42.59	37
1305.7175	2	1305.718	-0.0005	133	144	0	61.42	35
1328.6062	2	1328.6071	-0.0009	308	317	0	65.49	35
1373.6017	2	1373.6033	-0.0016	546	556	0	66.77	32
1385.7219	2	1385.7224	-0.0005	604	615	0	54.58	37
1425.7568	2	1425.7576	-0.0008	335	348	0	77.38	36
1435.7455	2	1435.746	-0.0005	352	363	0	88	37
1472.6777	2	1472.6783	-0.0006	40	52	0	73.97	35
1555.7451	3	1555.7453	-0.0002	306	317	1	39.01	37
1565.8007	2	1565.8011	-0.0003	117	130	0	85.6	37
1662.803	2	1662.8036	-0.0006	60	74	0	107.4	38
1674.722	2	1674.7234	-0.0014	227	242	0	93.76	32
1679.8246	2	1679.8267	-0.0021	145	159	0	42.35	38
2559.2448	2	2559.2452	-0.0004	368	393	0	117.52	37
2587.2102	3	2587.2126	-0.0024	5	28	0	54.09	36
2657.2566	2	2657.2609	-0.0043	431	453	0	100.3	36
3019.3945	3	3019.3934	0.0011	576	601	0	53.26	35
3024.4786	3	3024.4815	-0.003	279	305	0	53.04	36
3050.6922	2	3050.6937	-0.0015	394	422	0	38.43	28
3147.4882	3	3147.4884	-0.0002	575	601	1	90.03	36
832.5056	2	832.5058	-0.0002	4069	4075	0	34.2	30
914.5799	2	914.58	-0.0001	1793	1801	0	53.34	29
1034.5761	2	1034.576	0.0001	11	19	0	42.28	35
1248.6897	2	1248.69	-0.0003	3736	3746	0	82.48	35
1263.6481	2	1263.6499	-0.0018	4098	4108	0	41.32	38
1419.772	2	1419.7722	-0.0001	2674	2686	0	98.93	36
1439.748	2	1439.7483	-0.0003	23	35	0	53.14	36
1477.6731	2	1477.6732	-0.0002	1896	1909	0	94.1	35
1540.8385	2	1540.8395	-0.001	4372	4384	0	62.1	34
1618.8925	2	1618.893	-0.0005	3071	3085	0	97.62	34
1706.9243	2	1706.9243	0.0001	4396	4410	0	106.61	35
1727.9391	2	1727.9393	-0.0002	227	241	0	74.69	35
1885.0929	2	1885.0924	0.0005	4280	4296	0	44.68	27
1928.9619	3	1928.9626	-0.0007	3156	3173	0	58.08	37
2029.084	2	2029.0844	-0.0003	5284	5302	0	89.54	35
2053.0026	2	2053.0019	0.0007	5353	5368	0	67.05	38
2066.0846	3	2066.0836	0.0009	135	152	0	63.44	36
2092.0862	2	2092.0874	-0.0012	4244	4261	0	120.99	36
2109.174	2	2109.1721	0.0019	3757	3775	0	34.58	31
2164.037	2	2164.0378	-0.0007	1614	1630	0	83.11	37
2198.06	2	2198.0605	-0.0005	995	1014	0	100.04	37
2530.3182	3	2530.318	0.0002	1139	1161	0	46.13	36
1225.5824	2	1225.5827	-0.0003	81	91	0	71.67	35
1227.6194	2	1227.6207	-0.0013	29	39	0	73.88	36
1230.6384	2	1230.6391	-0.0006	164	175	0	68.57	38
1293.6173	2	1293.6176	-0.0003	243	252	0	42.59	37

1294.6561	2	1294.6557	0.0004	308	317	0	56.65	37
1307.6972	2	1307.6972	0	133	144	0	58.13	36
1373.6017	2	1373.6033	-0.0016	546	556	0	66.77	32
1385.7219	2	1385.7224	-0.0005	604	615	0	54.58	37
1406.7557	2	1406.7558	-0.0001	352	363	0	105.13	35
1430.7374	2	1430.7365	0.0009	335	348	0	89.84	37
1472.6777	2	1472.6783	-0.0006	40	52	0	73.97	35
1521.7939	2	1521.7939	0	306	317	1	51.31	37
1674.722	2	1674.7234	-0.0014	227	242	0	93.76	32
1676.8173	2	1676.8192	-0.0019	60	74	0	104.93	38
1679.8246	2	1679.8267	-0.0021	145	159	0	42.35	38
2573.2572	2	2573.2609	-0.0036	368	393	0	150.66	37
2587.2102	3	2587.2126	-0.0024	5	28	0	54.09	36
2657.2566	2	2657.2609	-0.0043	431	453	0	100.3	36
3064.7296	3	3063.7254	1.0042	394	422	0	36.74	25
3194.4595	3	3194.4601	-0.0006	575	601	1	79.89	35
1003.4762	2	1003.4757	0.0005	351	359	0	46.11	36
1058.5216	2	1058.5219	-0.0003	310	318	0	57.26	35
1076.5257	3	1076.525	0.0007	155	162	1	39.09	38
1138.686	2	1138.6862	-0.0002	253	262	0	63.43	28
1145.5827	2	1145.5829	-0.0002	242	251	0	68.26	38
1214.5771	2	1214.5754	0.0017	381	390	0	75.4	35
1266.7794	2	1266.7812	-0.0018	252	262	1	47.16	26
1292.6362	2	1292.6361	0.0001	36	46	0	81.65	38
1415.6827	2	1415.6829	-0.0002	163	174	0	81.16	36
1430.6243	2	1430.6248	-0.0006	47	58	0	81.01	32
1461.676	2	1461.6769	-0.0009	325	336	0	79.58	36
1637.8394	2	1637.8388	0.0006	263	276	0	95.76	37
1695.8238	2	1695.8257	-0.0019	337	350	0	77.72	37
1955.9915	2	1955.9953	-0.0038	104	121	0	157.53	37
2766.3114	3	2766.3139	-0.0025	217	241	0	62.67	36
2827.3449	3	2827.3467	-0.0018	78	103	1	66.38	36
955.5019	2	955.5015	0.0004	1696	1703	0	59.68	34
1070.5363	2	1070.5356	0.0007	1206	1215	0	36.6	35
1204.816	2	1204.8159	0.0001	1902	1912	0	53.83	20
1359.7511	2	1359.751	0.0001	391	403	0	95.55	36
1361.7377	2	1361.7377	0	1194	1205	0	81.22	36
1444.7924	2	1444.7926	-0.0001	882	895	0	97.99	36
1447.7914	2	1447.7922	-0.0008	1775	1787	0	88.45	35
1594.9433	2	1594.9446	-0.0013	283	296	0	38.85	28
1760.9493	2	1760.9495	-0.0002	1939	1953	0	91.41	35
2048.0984	2	2048.0983	0.0002	957	974	0	107.81	35
2097.1814	2	2097.182	-0.0006	2083	2101	0	128.19	30
2193.2254	2	2193.2256	-0.0002	440	459	0	58.05	30
2237.3144	3	2237.3147	-0.0003	2208	2228	1	42.91	20
2239.167	2	2239.1696	-0.0026	2495	2515	0	106.87	36
2555.3474	2	2555.3483	-0.0008	1514	1536	0	81.11	34
3139.7381	4	3139.7394	-0.0013	1537	1565	0	47.37	28
1058.5216	2	1058.5219	-0.0003	311	319	0	57.26	35
1076.5257	3	1076.525	0.0007	156	163	1	39.09	38
1138.686	2	1138.6862	-0.0002	254	263	0	63.43	28
1145.5827	2	1145.5829	-0.0002	243	252	0	68.26	38
1214.5771	2	1214.5754	0.0017	382	391	0	75.4	35
1266.7794	2	1266.7812	-0.0018	253	263	1	47.16	26
1379.6334	2	1379.6317	0.0017	36	47	0	71.02	36
1415.6827	2	1415.6829	-0.0002	164	175	0	81.16	36
1637.8394	2	1637.8388	0.0006	264	277	0	95.76	37
1678.7882	2	1678.7906	-0.0024	64	78	0	67.01	37

1695.8238	2	1695.8257	-0.0019	338	351	0	77.72	37
1955.9915	2	1955.9953	-0.0038	105	122	0	157.53	37
2813.327	3	2813.331	-0.004	79	104	1	101.02	36
847.4912	2	847.4916	-0.0003	445	452	0	59.61	35
950.4821	2	950.4821	0	974	982	0	44.09	37
971.5288	2	971.5287	0	41	48	0	53.79	35
987.5536	2	987.5535	0.0001	353	361	0	65.39	37
1080.6906	2	1080.6907	-0.0001	730	739	0	36.74	21
1151.5933	2	1151.5935	-0.0002	1073	1082	0	59.35	37
1268.7084	2	1268.7088	-0.0004	512	524	0	67.11	34
1291.6374	2	1291.6377	-0.0002	230	240	0	63.07	38
1467.7753	2	1467.7755	-0.0003	599	611	0	55.44	36
1482.7542	2	1482.7541	0.0001	389	400	0	94.48	37
1567.8252	2	1567.8246	0.0006	273	285	0	78.34	36
1926.0733	3	1926.0721	0.0013	172	188	0	56.61	32
1947.0493	3	1947.0499	-0.0007	1135	1152	0	67.59	35
1964.0135	2	1964.0136	-0.0001	191	209	0	129.65	37
2006.1166	2	2006.1162	0.0005	1000	1016	0	70.65	31
2240.2254	2	2240.2264	-0.0009	49	70	0	132.06	32
2500.3341	3	2500.336	-0.0019	453	475	0	40.1	34
2659.3374	3	2659.3381	-0.0007	847	870	0	61.51	37
3336.6946	3	3336.699	-0.0044	871	901	0	53.37	35
1094.6374	2	1094.6376	-0.0001	382	390	0	51.85	31
1128.6387	2	1128.6391	-0.0003	465	476	0	51.62	35
1156.6437	2	1156.6452	-0.0015	316	326	0	101.83	36
1388.6649	2	1388.6646	0.0003	330	341	0	74.4	36
1680.9409	2	1680.941	-0.0001	342	357	0	115.01	32
1767.7786	3	1767.7787	-0.0002	143	157	0	59.44	34
1809.8719	2	1809.8745	-0.0026	127	142	0	111	37
1827.9563	3	1827.9587	-0.0024	441	456	0	53.41	36
1906.0435	2	1906.0445	-0.001	93	112	0	81.54	33
2066.0004	2	2066.003	-0.0026	266	282	0	94.5	37
2113.0424	2	2113.0426	-0.0002	205	222	0	130.49	37
2134.1308	2	2134.131	-0.0002	283	301	0	112.04	35
2578.2786	3	2578.2777	0.0008	262	282	1	62.1	37
3434.734	3	3434.7344	-0.0004	223	254	0	37.78	35
902.4203	2	902.4208	-0.0004	395	401	0	37.75	33
986.5402	2	986.5397	0.0005	327	336	0	65.58	37
1131.5592	2	1131.5594	-0.0002	113	121	0	40.62	37
1282.4963	2	1282.4967	-0.0003	312	320	0	45.17	24
1395.6859	2	1395.6857	0.0003	391	401	1	41.06	37
1395.7505	3	1395.751	-0.0005	85	96	0	38.04	35
1530.8972	2	1530.8981	-0.0009	230	243	0	112.63	28
1583.744	2	1583.7443	-0.0003	340	352	0	73.14	36
1690.8617	2	1690.8638	-0.0021	216	229	1	61.44	38
1700.8967	2	1700.8985	-0.0018	65	79	0	91.09	37
1749.9139	2	1749.9124	0.0015	265	280	0	94.92	37
1884.9378	2	1884.9404	-0.0026	353	370	0	73.31	37
2345.0006	2	2345.0059	-0.0052	403	422	0	64.29	31
2384.1867	3	2384.1873	-0.0006	85	105	1	82.13	37
2408.196	2	2408.2012	-0.0052	244	264	0	112.41	38
3320.7187	3	3320.7187	-0.0001	124	156	1	118.55	34
1036.5241	2	1036.5223	0.0018	686	694	0	51.34	37
1155.5339	2	1155.5342	-0.0003	224	233	0	38.98	35
1254.6677	2	1254.6681	-0.0004	828	840	0	82.05	35
1266.602	2	1266.6026	-0.0006	660	669	0	52.36	37
1276.6141	2	1276.6147	-0.0005	575	586	0	66.6	37
1335.6665	2	1335.667	-0.0005	157	168	0	66.88	38

1402.8586	2	1402.8588	-0.0001	562	574	0	86.97	20
1507.6968	3	1507.6977	-0.0009	102	114	1	49.25	36
1564.9005	2	1564.9011	-0.0006	671	685	0	76.86	29
1715.0272	2	1715.0273	-0.0001	869	883	0	38.64	22
1974.0351	3	1973.0326	1.0026	811	827	1	40.34	36
2053.0382	2	2053.0401	-0.0019	115	133	0	93.25	37
2111.0444	2	2111.0436	0.0008	368	387	0	122.99	37
2443.3969	3	2443.3978	-0.0008	862	883	1	45.46	26
2465.1369	3	2465.138	-0.0011	462	483	1	74.52	36
2676.2766	3	2676.2775	-0.0009	170	192	0	38.69	37
2991.4388	3	2991.4383	0.0005	193	218	0	85.09	36
832.4565	2	832.4555	0.001	8	15	0	48.32	38
1037.5389	2	1037.5393	-0.0004	256	264	1	42.65	36
1139.6371	2	1139.6373	-0.0002	328	337	0	68.2	33
1433.7509	2	1433.7514	-0.0005	205	219	0	38.81	38
1497.8394	2	1497.8403	-0.0009	239	252	0	117.56	34
1675.9661	3	1675.9661	0	70	84	1	56.58	29
1749.8028	2	1749.8032	-0.0005	131	145	0	56.72	36
1760.7803	2	1760.7794	0.0009	314	327	0	75.81	33
2032.064	2	2032.0663	-0.0023	172	190	0	129.29	37
2171	2	2171.0019	-0.0018	276	295	0	147.08	36
2249.0654	2	2249.0668	-0.0013	146	166	0	151.52	37
2922.3549	3	2922.356	-0.0011	85	111	1	47.48	35
779.4056	2	779.4065	-0.0009	167	172	0	37.9	37
913.5592	2	913.5597	-0.0004	419	427	0	57.74	33
1024.6017	2	1024.603	-0.0013	244	254	0	70.5	31
1025.4815	2	1025.4818	-0.0003	205	212	0	45.01	35
1119.5911	2	1119.5924	-0.0013	21	30	0	52.22	37
1297.7397	2	1297.7394	0.0003	135	146	0	104.26	33
1450.6652	2	1450.6663	-0.0011	85	96	0	77.53	36
1633.8927	3	1633.894	-0.0013	6	20	0	56.51	34
1636.867	2	1636.8672	-0.0001	213	227	0	86.01	36
1762.9156	2	1762.9176	-0.002	262	278	0	57.15	37
2311.1206	3	2311.1228	-0.0022	392	411	1	62.32	37
2505.32	3	2505.3223	-0.0023	255	278	1	59.31	35
2972.4074	2	2972.4107	-0.0033	101	129	0	132.85	36
934.5115	2	934.5124	-0.0009	131	139	0	58.86	35
957.5128	2	957.5131	-0.0002	120	127	0	59.11	35
1003.5345	2	1003.5338	0.0007	292	299	1	43.74	38
1016.5388	2	1016.539	-0.0002	247	255	0	50.57	38
1046.5076	2	1046.5073	0.0004	55	63	0	40.23	37
1113.6757	2	1113.6758	-0.0001	333	342	0	80.17	31
1172.6401	2	1172.6401	0	246	255	1	58.44	38
1400.7246	2	1400.726	-0.0014	78	91	0	113.04	37
1586.6943	2	1586.6956	-0.0013	186	198	0	105.64	33
1826.9299	2	1826.9315	-0.0016	55	70	1	112.17	37
1919.9959	2	1919.988	0.0079	398	414	0	54.79	37
2060.0592	2	2060.0612	-0.002	215	233	0	97.09	37
2698.3882	2	2698.3888	-0.0005	268	290	0	96.87	35
2894.4936	2	2894.4927	0.001	343	367	0	86.39	35
765.4862	2	765.4861	0.0001	200	206	1	24.65	23
865.5134	2	865.5134	0	350	356	0	36.15	30
1129.5795	2	1129.5801	-0.0007	320	329	0	37.12	36
1157.6439	2	1157.6445	-0.0005	207	218	0	43.33	37
1194.5516	2	1194.5517	-0.0001	372	382	0	63.69	36
1294.6351	2	1294.634	0.0012	224	235	0	63.34	37
1304.6575	2	1304.6581	-0.0005	383	394	0	74.08	38
1643.8692	2	1643.8705	-0.0013	236	251	0	67.14	37

1864.0138	3	1864.0128	0.001	179	194	0	52.71	34
1885.8933	2	1885.8945	-0.0012	141	156	0	77.02	37
1982.9245	2	1982.9255	-0.001	294	310	0	147.29	37
2032.0538	3	2032.0551	-0.0012	262	279	1	73.76	37
2256.06	3	2256.0658	-0.0058	396	415	0	72.97	36
2268.0083	3	2268.0103	-0.002	159	178	1	71.28	34
2719.5167	3	2719.5161	0.0006	115	140	1	69.56	28
3830.7808	3	3829.784	0.9968	69	102	0	67.09	34
1227.6194	2	1227.6207	-0.0013	29	39	0	73.88	36
1293.6173	2	1293.6176	-0.0003	243	252	0	42.59	37
1328.6062	2	1328.6071	-0.0009	308	317	0	65.49	35
1373.6017	2	1373.6033	-0.0016	546	556	0	66.77	32
1435.7455	2	1435.746	-0.0005	352	363	0	88	37
1472.6777	2	1472.6783	-0.0006	40	52	0	73.97	35
1555.7451	3	1555.7453	-0.0002	306	317	1	39.01	37
1664.781	2	1664.7828	-0.0018	60	74	0	100.45	37
1674.722	2	1674.7234	-0.0014	227	242	0	93.76	32
1679.8246	2	1679.8267	-0.0021	145	159	0	42.35	38
2573.2572	2	2573.2609	-0.0036	368	393	0	150.66	37
2587.2102	3	2587.2126	-0.0024	5	28	0	54.09	36
1196.6869	2	1196.6877	-0.0008	164	175	0	67.79	33
1227.6194	2	1227.6207	-0.0013	29	39	0	73.88	36
1277.6868	2	1277.6867	0.0001	133	144	0	57.89	36
1293.6173	2	1293.6176	-0.0003	243	252	0	42.59	37
1373.6017	2	1373.6033	-0.0016	546	556	0	66.77	32
1435.7455	2	1435.746	-0.0005	352	363	0	88	37
1472.6777	2	1472.6783	-0.0006	40	52	0	73.97	35
1662.803	2	1662.8036	-0.0006	60	74	0	107.4	38
1674.722	2	1674.7234	-0.0014	227	242	0	93.76	32
1679.8246	2	1679.8267	-0.0021	145	159	0	42.35	38
2573.2572	2	2573.2609	-0.0036	368	393	0	150.66	37
2587.2102	3	2587.2126	-0.0024	5	28	0	54.09	36
792.4384	2	792.4382	0.0002	219	225	0	43.52	35
815.4865	2	815.4865	0.0001	121	127	0	40.64	37
1173.6064	2	1173.607	-0.0006	191	200	0	73.91	38
1221.6233	2	1221.6241	-0.0008	149	158	0	58.8	37
1478.7711	2	1478.7729	-0.0018	147	158	1	72.24	37
1514.7101	2	1514.7101	0	354	368	0	122.3	36
1681.8266	2	1681.8311	-0.0045	88	101	0	88.07	37
1828.9904	2	1828.9903	0.0001	312	328	0	148.63	35
1845.8484	2	1845.8493	-0.0009	159	174	0	48.6	35
2156.0709	3	2156.0724	-0.0015	129	146	1	46.05	37
2212.1076	2	2212.1124	-0.0047	332	351	0	101.38	37
766.3868	2	766.3861	0.0007	326	332	0	52.94	36
770.44	2	770.4399	0.0002	346	353	0	64.99	32
916.5749	2	916.5746	0.0004	367	374	0	51.69	26
1206.6238	2	1206.6245	-0.0006	354	366	0	92.11	38
1210.5998	2	1210.5983	0.0015	228	237	0	81.93	37
1359.7045	2	1359.7068	-0.0022	106	116	0	73.33	38
1407.7135	3	1407.7147	-0.0011	134	145	1	45.87	38
1445.7338	2	1445.7343	-0.0005	154	165	0	80.89	38
2047.0124	2	2047.0085	0.004	76	95	0	102.9	37
2763.3638	2	2763.3656	-0.0018	184	210	0	146.13	37
770.44	2	770.4399	0.0002	350	357	0	64.99	32
916.5749	2	916.5746	0.0004	371	378	0	51.69	26
950.4817	2	950.4821	-0.0004	246	254	0	43.74	38
1031.5046	2	1031.5036	0.001	141	149	0	54.4	37
1181.6074	2	1181.6081	-0.0007	232	241	0	55.95	36

1190.6278	2	1190.6295	-0.0018	358	370	0	86.18	37
1227.6128	2	1227.6136	-0.0007	330	340	0	59.39	36
1359.7045	2	1359.7068	-0.0022	110	120	0	73.33	38
1401.735	2	1401.7365	-0.0015	138	149	1	42	38
1445.7338	2	1445.7343	-0.0005	158	169	0	80.89	38
1468.7487	2	1468.7497	-0.0009	255	267	0	82.88	37
2049.9822	2	2048.97	1.0123	80	99	0	115.27	37
2763.3638	2	2763.3656	-0.0018	188	214	0	146.13	37
799.529	2	799.528	0.0011	64	70	1	31.58	29
975.4407	2	975.441	-0.0003	21	30	0	80.61	33
1162.59	2	1162.5903	-0.0003	318	328	0	39.15	38
1191.5222	2	1191.523	-0.0008	199	208	0	60.65	33
1474.668	2	1474.6688	-0.0008	362	374	0	71.03	34
1514.7409	2	1514.7419	-0.0009	87	97	0	52.47	37
1773.8891	2	1773.8897	-0.0006	241	256	0	115.28	38
1854.9235	2	1854.9251	-0.0015	218	233	0	120.18	37
1947.8654	2	1947.8673	-0.0018	71	86	0	72.27	33
1953.0553	2	1953.0571	-0.0018	98	115	0	84.37	34
2198.0662	2	2198.0678	-0.0015	294	314	0	121.1	37
3150.634	3	3150.635	-0.001	150	179	0	84.45	34
3193.5091	3	3193.5134	-0.0043	259	286	0	58.67	36
856.4766	2	856.4767	0	51	57	0	34.76	33
870.4936	2	870.4923	0.0013	208	215	0	47.37	34
982.4902	2	982.4906	-0.0004	197	205	0	65.83	35
1194.5757	2	1194.5768	-0.0012	112	121	0	74.33	37
1312.6268	2	1312.6299	-0.0031	78	89	0	69.32	36
1436.7526	2	1436.7525	0.0001	255	268	0	64.11	36
1464.7718	2	1464.7725	-0.0007	95	106	0	84.94	37
1608.7985	2	1608.7995	-0.001	216	231	0	107.55	38
1916.9259	2	1916.9264	-0.0005	232	247	0	141.41	37
2772.3867	4	2771.384	1.0027	135	158	0	58.14	36
942.5017	2	942.5022	-0.0005	191	198	1	56.54	35
1069.6387	2	1069.6383	0.0004	83	92	0	83.97	31
1247.6868	3	1247.6874	-0.0006	66	77	0	57.27	36
1299.6823	2	1299.6823	0.0001	46	56	1	53.68	36
1302.7431	2	1302.7435	-0.0004	136	146	0	71.99	33
1309.6128	2	1309.6125	0.0003	270	280	0	77.13	37
1379.6386	2	1379.6391	-0.0005	290	302	0	81.15	37
1493.7467	2	1493.7474	-0.0007	14	26	0	84.72	38
1512.8287	3	1512.83	-0.0013	204	216	0	74.47	35
1534.944	3	1534.9447	-0.0007	162	175	1	43.86	20
1768.8443	2	1768.8454	-0.0011	147	161	0	63.48	37
2150.1721	3	2150.1736	-0.0014	57	77	1	59.66	32
2320.2026	2	2320.2063	-0.0037	93	113	0	114.44	36
797.5135	2	797.5123	0.0012	307	313	0	41.94	27
971.5398	2	971.54	-0.0002	566	574	0	47.75	37
1028.5609	2	1028.5614	-0.0005	330	339	0	48.46	36
1037.5208	2	1037.5216	-0.0007	829	836	0	45.77	37
1049.5259	2	1049.5254	0.0005	521	529	0	63.24	37
1115.5818	2	1115.5822	-0.0004	862	870	0	38.97	37
1157.6285	2	1157.6292	-0.0006	200	209	0	58.28	37
1158.5863	2	1158.588	-0.0017	506	516	0	95.13	37
1381.7561	2	1381.7565	-0.0004	288	300	1	70.96	34
1396.73	2	1396.731	-0.001	236	248	0	55.84	36
1475.7753	2	1475.7773	-0.002	183	196	0	65.97	38
1511.7162	2	1511.7177	-0.0016	275	287	0	75.43	37
1891.0386	2	1891.0415	-0.0028	454	470	0	113.26	33
1958.0367	3	1958.0374	-0.0007	654	672	1	42.46	36

1984.9495	2	1984.9499	-0.0004	743	759	0	100.72	37
2032.1191	3	2032.1218	-0.0027	210	227	0	51.59	32
2507.3751	3	2507.3748	0.0003	136	160	0	88.48	31
3815.0467	4	3815.0468	-0.0002	385	421	1	28.78	28
865.5134	2	865.5134	0	349	355	0	36.15	30
1129.5795	2	1129.5801	-0.0007	319	328	0	37.12	36
1157.6439	2	1157.6445	-0.0005	206	217	0	43.33	37
1194.5516	2	1194.5517	-0.0001	371	381	0	63.69	36
1294.6351	2	1294.634	0.0012	223	234	0	63.34	37
1304.6575	2	1304.6581	-0.0005	382	393	0	74.08	38
1643.8692	2	1643.8705	-0.0013	235	250	0	67.14	37
1864.0138	3	1864.0128	0.001	178	193	0	52.71	34
1885.8933	2	1885.8945	-0.0012	140	155	0	77.02	37
1982.9245	2	1982.9255	-0.001	293	309	0	147.29	37
2256.06	3	2256.0658	-0.0058	395	414	0	72.97	36
2268.0083	3	2268.0103	-0.002	158	177	1	71.28	34
3831.796	3	3830.8044	0.9916	68	101	0	52.04	34
792.4502	2	792.4494	0.0008	365	371	0	43.45	34
1130.4635	2	1130.4629	0.0006	584	594	0	35.72	28
1158.4947	2	1158.4942	0.0005	602	613	0	43.2	31
1186.6957	2	1186.6962	-0.0004	155	165	0	45.41	33
1213.5401	2	1213.5397	0.0004	482	493	0	65.79	34
1260.6699	2	1260.6714	-0.0015	471	481	0	42.07	38
1499.6773	2	1499.6787	-0.0014	375	387	0	101.77	35
1597.8201	3	1597.8213	-0.0011	396	410	0	42.04	36
1719.7403	2	1719.7419	-0.0016	283	296	0	56.49	32
1814.9051	2	1814.905	0.0001	319	335	0	89.32	37
1889.9351	2	1889.9371	-0.002	302	318	0	130.75	38
2409.1274	2	2409.1304	-0.003	200	221	0	77.09	36
2427.2338	2	2427.2381	-0.0042	93	117	0	86.34	37
2583.3372	3	2583.3392	-0.002	92	117	1	57.71	35
832.4809	2	832.4807	0.0002	1742	1749	0	52.98	37
1005.5468	2	1005.5495	-0.0026	616	624	0	36.02	35
1070.5216	2	1070.5219	-0.0003	1508	1515	0	42.41	35
1101.5923	2	1101.5931	-0.0008	629	638	0	64.34	38
1279.7854	2	1279.7864	-0.001	802	814	0	90.91	25
1316.7085	2	1316.7122	-0.0038	539	550	0	46.38	37
1432.7145	2	1432.7158	-0.0012	71	84	0	123.04	38
1704.8498	2	1704.8505	-0.0007	1271	1284	0	120.9	38
2336.1514	2	2336.1536	-0.0021	1401	1421	0	131.09	37
2411.2056	2	2411.193	0.0126	1008	1029	0	97.16	37
3381.5092	3	3381.5116	-0.0024	753	781	0	48.15	32
1151.7065	2	1151.7067	-0.0002	157	167	0	74.04	24
1696.7544	2	1696.754	0.0004	73	87	0	99.93	34
1795.7963	2	1795.7975	-0.0012	378	392	0	61.42	34
2149.1232	2	2149.1242	-0.0009	278	296	0	142.92	36
2346.1794	2	2346.1815	-0.0021	410	432	0	161.67	37
2822.444	3	2822.4491	-0.0051	325	349	1	89.9	36
1018.5031	2	1018.5043	-0.0013	805	815	0	81.41	39
1047.5355	2	1047.5349	0.0006	469	477	1	45.78	38
1186.6301	2	1186.6306	-0.0005	575	585	0	68.24	38
1276.7016	2	1276.7027	-0.0011	779	789	0	60.5	36
1282.6156	2	1282.6162	-0.0006	543	553	0	65.12	36
1302.7082	2	1302.7085	-0.0003	352	362	0	89.16	37
1423.7565	2	1423.7567	-0.0002	478	489	0	59.66	35
1442.7748	2	1442.7769	-0.0022	261	272	0	68.68	36
1461.7609	2	1461.7616	-0.0007	683	694	0	92.59	38
1643.935	2	1643.9354	-0.0004	310	323	0	76.56	31

1835.9332	2	1835.9339	-0.0007	112	127	0	86.09	38
2484.2101	3	2484.2107	-0.0006	385	404	1	97.14	37
971.5655	2	971.5651	0.0003	253	261	0	47.9	36
1189.6334	2	1189.6343	-0.0009	224	235	0	38.34	37
1236.645	2	1236.6462	-0.0012	241	252	0	62.86	37
1286.7001	2	1286.7016	-0.0015	400	411	0	59.01	37
1750.8556	2	1750.856	-0.0004	311	325	0	131.12	38
2142.0299	3	2142.0303	-0.0004	71	87	1	64.09	37
2144.099	2	2144.1001	-0.001	182	201	0	125.32	37
2189.1302	2	2189.1324	-0.0021	159	178	0	81.86	36
2203.129	2	2203.126	0.003	276	296	0	96.81	37
1028.5609	2	1028.5614	-0.0005	309	318	0	48.46	36
1037.5208	2	1037.5216	-0.0007	808	815	0	45.77	37
1115.5818	2	1115.5822	-0.0004	841	849	0	38.97	37
1157.6285	2	1157.6292	-0.0006	180	189	0	58.28	37
1158.5863	2	1158.588	-0.0017	485	495	0	95.13	37
1381.7561	2	1381.7565	-0.0004	267	279	1	70.96	34
1396.73	2	1396.731	-0.001	216	228	0	55.84	36
1425.6529	2	1425.6525	0.0004	854	866	0	62.69	36
1475.7753	2	1475.7773	-0.002	163	176	0	65.97	38
1511.7162	2	1511.7177	-0.0016	254	266	0	75.43	37
1958.0367	3	1958.0374	-0.0007	633	651	1	42.46	36
1984.9495	2	1984.9499	-0.0004	722	738	0	100.72	37
2032.1191	3	2032.1218	-0.0027	190	207	0	51.59	32
2507.3751	3	2507.3748	0.0003	116	140	0	88.48	31
3815.0467	4	3815.0468	-0.0002	364	400	1	28.78	28
932.461	2	932.4603	0.0007	411	418	0	39.67	38
1090.5912	2	1090.591	0.0001	496	504	0	39.43	36
1160.5725	2	1160.5727	-0.0002	290	299	0	50.6	37
1162.634	2	1162.6346	-0.0006	395	403	1	39.17	36
1198.6339	2	1198.6346	-0.0007	280	289	0	48.69	35
1332.7105	2	1332.7112	-0.0006	219	230	0	59.37	37
1608.8708	2	1608.8722	-0.0014	310	324	0	67.77	35
1701.7291	2	1701.7304	-0.0013	258	273	0	103.23	31
1719.8654	2	1719.8679	-0.0024	516	530	0	117.35	38
1970.0458	3	1970.0473	-0.0015	478	495	0	52.31	35
2196.1002	2	2196.1022	-0.002	197	218	0	100.66	37
2656.3417	3	2656.3418	-0.0001	365	386	0	69.27	37
2782.3846	3	2782.3847	-0.0001	326	353	0	74.25	37
2910.4793	3	2910.4797	-0.0004	325	353	1	47.62	36
983.5645	2	983.5651	-0.0006	96	105	0	49.11	32
1247.6756	2	1247.6761	-0.0006	393	403	0	79.1	36
1248.5573	2	1248.5582	-0.0009	547	558	0	49.76	34
1255.6107	2	1255.6118	-0.0011	173	183	1	62.72	36
1293.5753	2	1293.5758	-0.0005	618	629	0	52.64	34
1360.7351	2	1360.7351	0	160	172	0	60.43	37
1432.7226	2	1432.7232	-0.0006	377	389	0	75.39	38
1476.7554	2	1476.7573	-0.0018	107	120	0	76.06	38
1812.9469	2	1812.9469	0	601	617	0	122.26	36
1821.8923	2	1821.8931	-0.0007	218	234	0	76.91	38
1838.8845	2	1838.8833	0.0012	467	483	0	79.22	37
743.3751	2	743.3755	-0.0004	392	397	0	35.21	33
764.4068	2	764.4069	-0.0001	233	239	0	36.11	36
905.4609	2	905.4607	0.0002	558	565	0	40.4	38
1038.6541	2	1038.655	-0.0008	151	159	1	29.12	24
1248.7176	2	1248.719	-0.0014	712	722	1	36	32
1451.6831	2	1451.6834	-0.0003	244	255	0	73.23	37
1517.6656	2	1517.6668	-0.0011	593	606	0	107.56	32

1566.7236	2	1566.7236	0.0001	544	557	0	76.49	36
1798.9198	2	1798.9254	-0.0056	771	786	0	59.37	37
2256.1954	2	2256.1961	-0.0007	33	55	0	115.47	35
2398.1526	3	2398.1553	-0.0026	349	368	1	41.25	37
2931.4244	3	2931.425	-0.0006	94	120	0	118.48	37
1251.7544	2	1251.7551	-0.0007	277	288	0	80.89	27
1368.624	2	1368.6245	-0.0005	220	230	0	47.83	35
1548.85	2	1548.8512	-0.0011	105	119	0	98.7	34
1613.9068	2	1613.9062	0.0006	428	441	0	108.33	33
1668.821	2	1668.822	-0.001	176	191	0	136.24	37
2006.2038	3	2006.204	-0.0003	289	306	0	68.36	22
3066.3748	3	3066.3764	-0.0016	250	276	0	58.68	33
1171.5891	2	1171.5907	-0.0016	81	90	0	37.99	35
1192.6554	2	1192.6564	-0.001	138	147	0	58.62	35
1216.5975	2	1216.5989	-0.0014	321	330	0	53.61	36
1279.623	2	1279.6231	0	207	217	0	78.36	37
1422.6565	2	1422.6568	-0.0002	183	193	0	74.74	36
1482.8142	2	1482.8155	-0.0013	102	116	0	107.84	34
1585.8677	2	1585.8684	-0.0007	306	320	0	59.91	36
1802.9248	2	1802.9262	-0.0014	348	365	0	62.06	37
2032.0659	3	2032.0688	-0.003	346	365	1	47.71	37
2275.168	2	2275.1671	0.001	118	137	0	76.42	37
1241.6423	2	1241.6438	-0.0015	80	90	0	56.41	35
1934.0261	2	1934.0262	0	180	197	0	114.26	36
2430.232	2	2430.2319	0.0002	249	271	0	100.28	37
2483.216	2	2483.2166	-0.0006	91	113	0	126.7	37
2812.4985	3	2812.5011	-0.0025	246	271	1	54.96	33
2945.4675	3	2945.4692	-0.0017	219	245	0	98.86	36
4101.0821	4	4101.0866	-0.0045	124	164	1	33.86	33
919.479	2	919.4797	-0.0007	43	52	0	51.29	38
940.5019	2	940.5018	0.0001	63	70	0	41.23	34
1438.6787	2	1438.6801	-0.0013	27	42	0	118.74	37
1514.7201	2	1514.7212	-0.0011	76	89	0	137.56	36
2155.0158	2	2155.0181	-0.0023	71	89	1	94.69	37
1099.6601	2	1099.6601	0.0001	721	731	0	62.22	33
1232.6173	2	1232.6183	-0.001	623	633	0	55.36	38
1247.5814	2	1247.5816	-0.0001	579	589	0	55.89	36
1257.6962	2	1257.6969	-0.0007	875	886	0	39.61	36
1276.6657	2	1276.6663	-0.0006	318	330	0	67.23	37
1280.6179	2	1280.6183	-0.0004	331	341	0	65.34	37
1363.672	2	1363.6732	-0.0011	1007	1019	0	77.1	38
1405.5694	2	1405.5708	-0.0014	534	545	0	33.66	27
1554.8605	2	1554.8617	-0.0012	763	777	0	96.14	33
2202.1314	3	2201.1289	1.0025	740	759	0	70.04	37
2488.3173	3	2488.3173	0	385	407	0	80.49	34
974.5543	2	974.5549	-0.0006	227	237	0	42.36	36
1172.655	2	1172.6554	-0.0004	214	223	0	42.91	37
1277.6285	2	1277.6286	0	135	146	0	56.21	38
1392.8079	2	1392.8089	-0.001	150	163	0	44.76	28
1424.7664	2	1424.7664	0	309	321	0	104.32	35
1456.8326	2	1456.8323	0.0003	238	250	0	83.24	33
1491.767	2	1491.7681	-0.0011	338	351	0	122.1	38
1863.9361	2	1863.9367	-0.0006	322	337	0	86.93	38
2687.3679	3	2687.3687	-0.0008	460	483	1	55.65	36
856.4766	2	856.4767	0	51	57	0	34.76	33
870.4936	2	870.4923	0.0013	208	215	0	47.37	34
948.5392	2	948.5392	0	197	205	0	78.23	35
1073.5345	2	1073.5353	-0.0007	276	286	0	54.63	37

1218.6476	2	1218.6496	-0.002	112	121	0	66.88	38
1393.745	2	1393.7466	-0.0017	255	268	0	47.7	36
1407.7507	2	1407.7511	-0.0003	95	106	0	107.46	36
1574.83	2	1574.8304	-0.0005	216	231	0	76.53	37
1966.8723	2	1966.8727	-0.0004	232	247	0	88.54	34
2735.3338	3	2735.3364	-0.0026	135	158	0	66.12	37
718.4378	2	718.4377	0.0001	377	383	0	34.83	30
896.5694	2	896.5695	0	348	356	0	30.86	26
1158.4947	2	1158.4942	0.0005	596	607	0	43.2	31
1183.5292	2	1183.5292	0	494	505	0	87.24	34
1186.6957	2	1186.6962	-0.0004	167	177	0	45.41	33
1483.6827	3	1483.6838	-0.001	387	399	0	61.09	36
1536.8355	3	1536.8372	-0.0017	457	470	1	47.6	35
1547.794	2	1547.7944	-0.0004	408	422	0	95.5	38
1719.7403	2	1719.7419	-0.0016	295	308	0	56.49	32
1795.7387	2	1795.7398	-0.0011	569	588	0	89.33	28
1903.9505	2	1903.9527	-0.0022	314	330	0	55.44	38
2409.1274	2	2409.1304	-0.003	212	233	0	77.09	36
1290.7433	2	1290.7435	-0.0001	285	295	0	80.5	31
1442.7975	2	1442.7981	-0.0005	621	633	0	69.96	35
1770.0252	2	1770.0251	0.0001	296	312	0	139.02	29
1892.9189	2	1892.919	-0.0001	492	507	0	91.23	38
1924.0186	2	1924.0193	-0.0007	221	236	0	116.46	35
2526.147	3	2526.1481	-0.0012	349	370	0	53.32	35
959.5802	2	959.5804	-0.0002	711	718	0	45.47	32
1005.5619	2	1005.5607	0.0011	927	935	0	49.73	36
1035.5494	2	1035.5502	-0.0008	771	779	0	55.09	36
1132.6488	2	1132.6492	-0.0004	729	738	0	62.23	33
1485.8398	2	1485.8402	-0.0005	109	122	0	102.34	34
1746.8877	2	1746.89	-0.0023	32	48	0	105.24	38
1913.96	2	1913.9597	0.0003	181	196	0	89.47	37
2460.2712	2	2460.2723	-0.001	6	26	0	98.96	36
1023.472	2	1023.4729	-0.0009	185	192	0	40.01	36
1076.5168	2	1076.5172	-0.0004	246	255	0	55.24	37
1277.6177	2	1277.6187	-0.0009	348	358	0	84.47	38
1932.925	2	1932.9285	-0.0034	268	285	0	122.24	37
2201.0886	2	2201.0892	-0.0006	314	332	0	141.56	37
3373.5715	3	3372.5721	0.9993	193	222	0	72.77	35
989.5538	2	989.5546	-0.0008	693	700	0	57.47	36
1041.4875	2	1041.488	-0.0005	364	372	0	43.08	34
1235.634	2	1235.6333	0.0007	388	397	0	60.62	38
1255.6444	2	1255.6448	-0.0004	589	598	0	66.5	35
1324.6733	3	1324.6735	-0.0002	412	422	1	50.67	37
1469.7869	2	1469.7878	-0.0009	143	156	0	72.83	36
1644.8247	2	1644.8253	-0.0007	442	459	0	97.66	38
1648.787	2	1648.7879	-0.0008	258	272	0	89.4	37
1664.8403	2	1664.841	-0.0006	677	692	0	109.36	38
1715.9179	3	1715.9206	-0.0027	320	334	0	43.72	36
1145.5928	2	1145.5928	0	119	130	0	59.71	38
1237.5753	2	1237.5761	-0.0009	544	554	0	46.74	37
1656.8131	2	1656.8141	-0.001	181	195	0	70.7	37
1729.8259	2	1729.8271	-0.0013	517	532	0	100.29	37
2165.0904	2	2165.0926	-0.0021	427	446	0	114.55	37
2679.3395	3	2679.3405	-0.0011	486	510	0	71.3	37
1378.7092	2	1378.7092	0	376	389	0	102.85	38
1400.7219	2	1400.7259	-0.004	285	299	0	54.21	37
1545.7809	2	1545.7821	-0.0012	319	332	0	111.94	38
1617.8544	2	1617.8548	-0.0005	408	422	0	50.85	37

2156.9842	2	2156.9862	-0.002	356	375	0	136.26	35
3039.4885	3	3038.4873	1.0012	164	191	1	71.76	36
815.4865	2	815.4865	0.0001	121	127	0	40.64	37
1173.6064	2	1173.607	-0.0006	191	200	0	73.91	38
1221.6233	2	1221.6241	-0.0008	149	158	0	58.8	37
1478.7711	2	1478.7729	-0.0018	147	158	1	72.24	37
1514.7101	2	1514.7101	0	354	368	0	122.3	36
1799.9296	2	1799.9305	-0.001	46	63	0	68.6	37
1821.9571	3	1821.9584	-0.0013	335	351	0	41.03	36
2156.0709	3	2156.0724	-0.0015	129	146	1	46.05	37
1088.6214	2	1088.623	-0.0016	118	127	0	82.65	34
1102.605	2	1102.6056	-0.0007	20	30	0	67.51	38
1564.7522	2	1564.7522	0	60	73	0	109.83	38
1645.7611	2	1645.7617	-0.0006	128	142	0	89.25	36
2105.0823	3	2105.0827	-0.0003	110	127	1	62.42	37
2152.0323	3	2152.0338	-0.0015	80	97	0	79.56	37
2322.2334	2	2322.2359	-0.0024	39	59	0	66.32	35
737.4798	2	737.48	-0.0001	168	174	1	28.34	24
930.5384	2	930.5386	-0.0002	204	212	0	42	36
1157.6439	2	1157.6445	-0.0005	175	186	0	43.33	37
1272.6346	2	1272.635	-0.0004	329	339	0	63.12	37
1275.6832	2	1275.6823	0.0009	192	203	0	53.6	37
1311.5785	2	1311.5805	-0.002	366	375	0	38.33	34
1391.7508	2	1391.7516	-0.0009	104	115	0	84.33	36
1397.6426	2	1397.6431	-0.0006	351	362	0	70.08	35
1935.9605	2	1935.9612	-0.0007	262	278	0	97.21	38
2222.2021	3	2222.2019	0.0002	55	74	1	69.97	33
960.528	2	960.528	0	94	102	0	67.46	37
1239.6246	2	1239.6248	-0.0001	206	215	0	54.72	37
1395.7294	3	1395.7299	-0.0005	351	362	0	37.43	36
1402.7099	2	1402.7126	-0.0027	324	336	0	80.23	37
1601.7015	2	1601.7032	-0.0017	385	399	0	74.34	32
1716.8851	2	1716.8869	-0.0018	113	126	0	102.64	38
1927.9847	2	1927.9859	-0.0012	292	310	0	93.04	37
2931.4203	3	2931.418	0.0023	167	191	1	46.65	37
799.529	2	799.528	0.0011	64	70	1	31.58	29
975.4407	2	975.441	-0.0003	21	30	0	80.61	33
1013.4738	2	1013.4739	-0.0001	186	193	0	42.11	34
1162.59	2	1162.5903	-0.0003	318	328	0	39.15	38
1514.7409	2	1514.7419	-0.0009	87	97	0	52.47	37
1947.8654	2	1947.8673	-0.0018	71	86	0	72.27	33
1953.0553	2	1953.0571	-0.0018	98	115	0	84.37	34
2212.0816	2	2212.0834	-0.0018	294	314	0	107.96	37
3150.634	3	3150.635	-0.001	150	179	0	84.45	34
996.6331	2	996.6331	0	509	517	0	35.23	25
1165.5977	2	1165.5979	-0.0002	40	50	0	59.83	37
1301.7937	2	1301.7959	-0.0022	1057	1068	0	49.52	24
2169.1866	2	2169.1874	-0.0008	705	723	0	53.93	33
2176.1296	2	2176.131	-0.0014	204	223	0	96.72	36
2198.0995	3	2197.095	1.0045	416	434	0	39.87	37
2379.1238	2	2379.1246	-0.0007	874	893	0	43.57	37
2503.2458	2	2503.2476	-0.0017	270	292	0	77.02	37
2590.2312	3	2590.2333	-0.0021	518	541	0	59.67	37
2783.3719	4	2783.3739	-0.002	437	461	0	40.84	37
2798.5226	3	2798.5218	0.0008	159	183	0	52.12	31
897.4921	2	897.492	0.0001	24	31	0	52.09	33
1169.5958	2	1169.5968	-0.001	178	187	0	57.22	35
1239.6971	2	1239.6975	-0.0005	40	49	0	44.79	33

1374.7531	2	1374.7541	-0.0009	128	139	1	78.53	36
1581.8469	2	1581.8475	-0.0006	75	89	0	78.98	35
1791.8936	3	1791.8944	-0.0008	97	113	0	42.57	37
1864.933	2	1864.934	-0.001	3	19	0	114.42	37
1866.9832	3	1866.9839	-0.0008	40	55	1	38.21	36
1914.01	3	1913.0106	0.9995	143	159	1	69.24	36
1159.6518	2	1159.6522	-0.0005	282	291	0	67.92	35
1182.6238	2	1182.6244	-0.0007	433	443	0	82.74	35
1206.6605	2	1206.6608	-0.0003	373	383	0	85.6	36
1259.6759	2	1259.6761	-0.0002	468	478	0	82.68	37
1329.7054	2	1329.7075	-0.0021	234	246	0	93.5	38
1527.8496	2	1527.8508	-0.0012	135	150	0	53.82	34
3610.7128	3	3610.7131	-0.0003	309	341	1	50.96	36
856.512	2	856.513	-0.0011	545	551	0	36.42	33
983.5645	2	983.5651	-0.0006	101	110	0	49.11	32
1129.5607	2	1129.5615	-0.0008	613	622	0	57.04	35
1189.6704	2	1189.6707	-0.0003	398	408	0	75.9	35
1234.5669	2	1234.5677	-0.0008	552	563	0	69.96	36
1255.6107	2	1255.6118	-0.0011	178	188	1	62.72	36
1416.7196	2	1416.7209	-0.0013	112	125	0	61.41	37
1432.7226	2	1432.7232	-0.0006	382	394	0	75.39	38
1612.8656	3	1612.8685	-0.003	352	364	1	56.31	35
2455.3926	2	2455.3938	-0.0011	437	459	0	73.38	27
1122.5718	2	1122.5743	-0.0025	200	209	0	40.97	37
1262.6368	3	1262.6367	0	361	371	0	46.03	38
1315.6617	2	1315.6619	-0.0002	181	194	0	69.4	37
1559.8023	2	1559.8042	-0.002	124	138	0	96.08	38
2115.0064	2	2115.0062	0.0003	41	60	0	95.52	37
2375.1066	2	2375.107	-0.0004	139	160	0	128.33	36
2449.1529	3	2448.1526	1.0003	334	356	0	53.64	37
799.529	2	799.528	0.0011	64	70	1	31.58	29
975.4407	2	975.441	-0.0003	21	30	0	80.61	33
1131.5194	2	1131.5197	-0.0003	199	208	0	51.64	34
1162.59	2	1162.5903	-0.0003	318	328	0	39.15	38
1514.7409	2	1514.7419	-0.0009	87	97	0	52.47	37
1789.881	2	1789.8846	-0.0036	241	256	0	107.03	38
1968.0739	3	1967.0727	1.0012	98	115	0	35.21	34
1974.8756	3	1974.8782	-0.0026	71	86	0	35.39	34
2212.0816	2	2212.0834	-0.0018	294	314	0	107.96	37
3150.634	3	3150.635	-0.001	150	179	0	84.45	34
987.477	2	987.4774	-0.0004	198	205	0	38.58	34
1034.5512	2	1034.5509	0.0003	413	423	0	38.99	37
1204.5471	2	1204.5473	-0.0002	981	991	0	43.87	35
1248.6103	2	1248.6098	0.0004	816	827	0	40.17	38
1382.7441	2	1382.7445	-0.0004	591	602	0	69.45	35
1495.8294	2	1495.8286	0.0008	705	717	0	108.53	33
1617.8409	2	1617.8403	0.0007	992	1006	0	87.25	37
1912.1402	2	1912.1397	0.0005	1025	1041	0	93.89	22
939.5385	2	939.5389	-0.0004	286	293	0	36.35	30
1267.6035	2	1267.6044	-0.0009	51	61	0	47.17	37
1308.7441	2	1308.7442	-0.0001	146	157	0	81.7	32
1358.7812	2	1358.7809	0.0003	101	112	0	35.71	32
1371.7443	2	1371.7439	0.0005	179	190	0	67.82	37
1652.805	2	1652.8046	0.0004	131	143	0	81.4	37
1675.8141	2	1675.8152	-0.0011	264	278	0	56.53	38
1905.0092	2	1905.0095	-0.0003	191	206	0	94.43	36
1948.8598	2	1948.8578	0.0021	396	411	0	88.14	33
838.459	2	838.4589	0.0001	54	60	0	53.24	33

1180.5727	2	1180.5724	0.0003	410	420	0	49.67	37
1287.7028	2	1287.7074	-0.0046	437	447	0	49.78	37
1408.6901	2	1408.6908	-0.0007	82	93	0	55.73	38
1528.8072	2	1528.8079	-0.0006	278	291	0	64.14	37
1535.9538	3	1535.9538	0	559	572	0	79.82	20
1593.8626	3	1593.8627	-0.0001	384	398	0	45.1	34
1819.9073	2	1819.9098	-0.0025	185	202	0	117.42	38
910.4896	2	910.4913	-0.0016	125	131	0	39.36	34
1266.7282	2	1266.7296	-0.0014	282	293	0	49.15	31
1368.624	2	1368.6245	-0.0005	225	235	0	47.83	35
1576.8456	3	1576.8461	-0.0005	110	124	0	39.1	36
1613.9068	2	1613.9062	0.0006	433	446	0	108.33	33
1668.821	2	1668.822	-0.001	181	196	0	136.24	37
2228.2521	3	2228.2528	-0.0007	199	219	0	37.79	30
2491.3427	3	2491.3435	-0.0008	158	180	0	72.95	33
1181.7021	2	1181.702	0.0002	78	89	0	60.29	30
1198.6557	2	1198.6557	0	286	296	0	67.81	35
1279.7603	2	1279.7612	-0.0009	161	173	0	79.81	28
1551.7472	2	1551.749	-0.0019	542	555	0	73.44	38
2568.4062	2	2568.405	0.0012	297	320	0	128.47	31
754.4342	2	754.4337	0.0005	405	411	0	31.91	31
1025.587	2	1025.5869	0.0001	424	433	0	48.6	32
1042.5665	2	1042.5659	0.0006	363	373	0	44.33	35
1215.6174	2	1215.6169	0.0004	379	390	0	50.24	37
1241.607	3	1241.6074	-0.0004	413	423	0	42.13	36
1249.6297	2	1249.6302	-0.0005	277	287	0	66.87	38
1329.7499	2	1329.7504	-0.0005	448	459	0	74.17	35
2307.1486	2	2307.1495	-0.0009	672	693	0	151.72	38
949.5239	2	949.5233	0.0006	406	413	0	48.5	35
1216.7324	3	1216.7332	-0.0008	70	79	1	43.6	28
1291.652	3	1291.6521	0	307	317	0	44.38	38
1492.7226	2	1492.7232	-0.0006	271	284	0	68.74	38
1547.8812	2	1547.881	0.0002	139	152	0	102.78	31
1960.9654	3	1960.9676	-0.0022	318	333	0	39.09	37
2095.1654	3	2095.1677	-0.0023	341	359	0	82.48	31
2317.0328	2	2317.0328	0.0001	223	242	0	107.44	34
784.5062	2	784.5058	0.0004	267	273	0	45.02	31
825.4954	2	825.496	-0.0006	523	530	0	35.63	29
1015.5184	2	1015.5186	-0.0001	50	59	0	39.45	36
1195.7286	2	1195.7288	-0.0002	469	479	0	58.62	28
1266.5685	2	1266.5696	-0.0012	137	148	0	87.26	35
1414.7759	2	1414.778	-0.0021	153	165	0	68.77	36
1523.7534	2	1523.7541	-0.0007	213	225	0	71.88	37
1660.7949	2	1660.7944	0.0005	500	514	0	89.94	37
2616.4135	3	2616.411	0.0026	534	559	0	74.48	32
839.5115	2	839.5116	-0.0001	353	360	0	40.99	28
1064.5905	2	1064.5906	-0.0001	15	23	0	52.56	34
1369.6799	2	1369.6799	0	127	138	0	73.13	37
1418.7	2	1418.7041	-0.0042	303	315	1	43.29	38
1442.6546	2	1442.6565	-0.0019	69	80	0	67.64	34
2160.9136	3	2160.9173	-0.0037	178	195	0	35.61	30
2720.3548	3	2720.3545	0.0003	236	260	0	83.21	37
3020.2786	3	3020.2785	0.0001	81	111	0	94.56	29
3431.7165	4	3431.7184	-0.0019	268	297	1	69.43	36
764.4179	2	764.4181	-0.0002	281	287	0	37.17	34
872.509	2	872.508	0.001	292	300	0	41.47	38
978.466	2	978.4672	-0.0012	365	373	0	38.96	36
1205.5991	2	1205.604	-0.0049	61	70	0	73.16	38

1295.7699	3	1295.77	-0.0001	328	338	1	35.94	29
1452.7465	2	1452.7474	-0.0009	238	252	0	89.72	37
1501.7136	2	1501.7157	-0.0021	48	60	0	46.5	36
1661.8307	2	1661.8301	0.0006	374	387	1	61.66	38
1808.8227	2	1808.8251	-0.0023	75	91	0	90.37	36
2382.2639	3	2382.2656	-0.0017	92	113	0	38.81	34
2648.3283	3	2648.3293	-0.001	188	211	0	69.88	36
1157.6439	2	1157.6445	-0.0005	197	208	0	43.33	37
1340.6217	2	1340.6217	0.0001	374	385	0	65.83	36
1478.8561	3	1478.8569	-0.0008	311	323	1	34.23	30
1774.8658	3	1774.8672	-0.0014	388	402	1	42.08	38
1894.8979	2	1894.8983	-0.0003	155	171	0	79.36	37
1938.946	2	1937.9404	1.0056	285	301	0	116.45	37
2432.2588	3	2432.2621	-0.0033	70	90	1	57.51	36
928.5706	2	928.5705	0.0001	277	285	0	50.46	33
1337.7233	2	1337.7231	0.0002	262	273	0	68.19	35
1382.6851	2	1382.6864	-0.0013	628	640	0	114.42	37
2216.304	3	2216.3031	0.0009	378	398	0	71.47	23
2302.1828	2	2302.1845	-0.0017	399	420	0	137.2	36
1042.5563	2	1042.556	0.0004	668	676	0	48.54	36
1340.6611	2	1340.6612	-0.0001	164	174	0	72.04	36
1362.7908	2	1362.7911	-0.0003	68	79	0	100.95	29
1670.9272	2	1670.9276	-0.0004	891	905	0	103.16	34
2449.2748	2	2449.2741	0.0008	8	31	0	62.72	35
2719.4615	3	2718.4593	1.0023	6	31	1	67.06	33
1194.6384	2	1194.6397	-0.0013	302	312	0	51.49	35
1799.9848	2	1799.9881	-0.0033	88	105	0	93.86	35
1854.0512	2	1854.0502	0.001	437	453	0	98.45	30
2222.1828	2	2222.1835	-0.0006	163	183	0	115.37	34
3201.6505	3	3201.6517	-0.0013	405	436	0	48.65	34
799.529	2	799.528	0.0011	64	70	1	31.58	29
975.4407	2	975.441	-0.0003	21	30	0	80.61	33
1013.4738	2	1013.4739	-0.0001	186	193	0	42.11	34
1162.59	2	1162.5903	-0.0003	318	328	0	39.15	38
1191.5222	2	1191.523	-0.0008	199	208	0	60.65	33
1514.7409	2	1514.7419	-0.0009	87	97	0	52.47	37
1773.8891	2	1773.8897	-0.0006	241	256	0	115.28	38
1933.8481	3	1933.8516	-0.0035	71	86	0	37.74	33
1290.6821	2	1290.682	0.0001	1272	1283	0	76.12	37
1294.8338	2	1294.8336	0.0001	618	629	0	95.94	20
1374.8122	2	1374.8122	0	225	236	0	79.24	28
1942.0884	3	1942.0887	-0.0003	225	241	1	44.72	32
2028.1254	2	2028.1255	-0.0001	839	856	0	48.81	32
2073.1194	2	2073.1218	-0.0024	694	713	0	89.34	34
2634.3939	3	2634.3945	-0.0005	93	115	0	36.06	34
1188.6341	2	1188.635	-0.0009	182	194	0	87.04	38
1342.7086	2	1342.7092	-0.0006	195	209	0	79.97	37
1407.6667	2	1407.6671	-0.0003	293	305	0	57.34	37
1822.9454	2	1822.9465	-0.0011	43	59	0	111.17	36
2479.0672	2	2479.0697	-0.0024	106	127	0	98.54	31
977.5188	2	977.5182	0.0006	37	44	0	46.9	36
1020.5598	2	1020.5604	-0.0006	361	369	0	37.06	36
1352.7907	2	1352.7915	-0.0009	238	250	0	37.04	30
1530.7148	2	1530.7161	-0.0013	86	99	0	127.5	36
1681.83	2	1681.8312	-0.0012	320	334	0	94.01	38
3107.5153	3	3107.5155	-0.0002	45	72	0	70.96	37
1108.5333	2	1108.5335	-0.0002	456	464	0	40.72	37
1147.588	2	1147.5873	0.0007	468	477	0	43.86	38

1300.6427	2	1300.6445	-0.0018	97	108	0	36.69	36
1491.7921	2	1491.7933	-0.0012	544	557	0	75.9	37
1542.8306	3	1542.8307	-0.0001	186	198	0	46.4	37
2044.0958	2	2044.0953	0.0006	707	724	0	116.35	35
2510.0242	3	2510.0259	-0.0017	353	373	0	44.1	26
2885.5102	3	2884.5078	1.0024	375	401	0	35.92	34
3112.6351	4	3112.6379	-0.0028	159	185	1	54.44	33
1036.5241	2	1036.5223	0.0018	686	694	0	51.34	37
1254.6677	2	1254.6681	-0.0004	828	840	0	82.05	35
1276.6141	2	1276.6147	-0.0005	575	586	0	66.6	37
1402.8586	2	1402.8588	-0.0001	562	574	0	86.97	20
1564.9005	2	1564.9011	-0.0006	671	685	0	76.86	29
1701.012	2	1701.0117	0.0004	869	883	0	48.91	24
711.4281	2	711.4279	0.0002	219	225	0	30.68	28
1172.5859	2	1172.586	0	512	521	0	62.57	36
1319.621	3	1319.6218	-0.0008	372	383	0	48.69	37
1397.6713	2	1397.6715	-0.0002	206	217	0	63.28	37
1525.7666	2	1525.7664	0.0001	206	218	1	50.64	37
1927.9405	3	1927.9422	-0.0017	384	400	0	65.35	37
2271.159	2	2271.1595	-0.0004	85	106	0	87.15	37
2521.3746	3	2521.3751	-0.0005	437	461	0	68.85	31
3319.6366	3	3319.6401	-0.0035	289	317	0	40.42	36
1101.6552	2	1101.6546	0.0006	73	82	0	97.13	32
1310.781	2	1310.7809	0.0001	1205	1215	0	79.62	29
1543.7911	2	1543.7916	-0.0005	397	410	0	87.53	37
1724.8415	2	1723.8529	0.9886	1809	1823	0	92.11	37
2332.2633	3	2332.2638	-0.0005	657	677	0	40.23	33
784.5062	2	784.5058	0.0004	266	272	0	45.02	31
825.4954	2	825.496	-0.0006	522	529	0	35.63	29
1015.5184	2	1015.5186	-0.0001	49	58	0	39.45	36
1195.7286	2	1195.7288	-0.0002	468	478	0	58.62	28
1266.5685	2	1266.5696	-0.0012	136	147	0	87.26	35
1417.6863	2	1417.6878	-0.0015	228	239	0	59.48	37
1523.7534	2	1523.7541	-0.0007	212	224	0	71.88	37
1660.7949	2	1660.7944	0.0005	499	513	0	89.94	37
1192.6554	2	1192.6564	-0.001	138	147	0	58.62	35
1216.5975	2	1216.5989	-0.0014	321	330	0	53.61	36
1422.6565	2	1422.6568	-0.0002	183	193	0	74.74	36
1468.7985	2	1468.7998	-0.0013	102	116	0	105.11	35
2235.1356	2	2235.1358	-0.0001	118	137	0	81.64	37
840.5179	2	840.5181	-0.0002	269	276	0	45.56	30
1323.7762	2	1323.7762	0	139	151	0	59.89	28
1598.8068	2	1598.8086	-0.0018	161	174	0	86.41	37
1677.8197	2	1677.8243	-0.0046	239	253	0	91.95	38
3301.6891	3	3300.6878	1.0013	7	39	0	89.49	35
1182.5915	2	1182.5921	-0.0006	747	756	0	48.77	36
1196.5818	2	1196.5826	-0.0008	138	148	0	60.27	36
1309.6364	2	1309.6375	-0.0011	163	174	0	46.38	37
1490.7989	2	1490.798	0.0009	699	712	0	101.97	37
1534.7204	2	1534.7223	-0.0019	469	483	0	109.83	37
2406.2456	2	2406.2471	-0.0015	75	97	0	41.61	36
940.5454	2	940.5454	0.0001	190	198	1	33.81	30
1033.5229	2	1033.5226	0.0004	344	352	0	58.74	38
1443.6492	3	1443.6491	0	63	76	0	40.91	34
1571.7435	3	1571.7441	-0.0006	62	76	1	79.14	36
1612.7964	2	1612.7984	-0.002	266	279	1	58.43	37
1928.7984	2	1928.7986	-0.0002	385	400	0	90.96	29
2171.063	2	2171.0667	-0.0037	149	168	0	55.68	37

991.5337	2	991.5339	-0.0002	220	228	0	57.69	36
1157.6439	2	1157.6445	-0.0005	191	202	0	43.33	37
1270.6785	3	1270.6782	0.0003	208	219	0	40.18	36
1288.5965	2	1288.5976	-0.001	399	408	1	40.79	35
1419.82	3	1419.8198	0.0002	112	124	1	51.38	30
2028.0518	2	2028.0527	-0.0009	246	264	0	101.05	37
2068.9246	2	2068.9259	-0.0012	15	33	0	100.8	34
1255.6887	2	1255.6884	0.0003	88	99	0	50.66	35
1290.6305	2	1290.6303	0.0002	34	45	0	52.22	38
1319.6509	2	1319.651	-0.0001	299	309	0	46.09	38
1526.7933	2	1526.794	-0.0007	358	371	0	82.13	36
1582.7837	3	1582.7839	-0.0002	441	454	1	39.73	37
2239.1438	2	2239.1446	-0.0007	486	505	0	116.47	37
868.4917	2	868.4919	-0.0002	232	239	0	36.98	32
992.5481	2	992.5477	0.0003	319	327	0	61.35	35
1099.5017	2	1099.5015	0.0002	151	158	0	38.39	33
1576.7844	2	1576.7847	-0.0003	248	260	0	86.66	38
2137.1762	2	2137.1783	-0.0021	352	374	0	75.35	32
1044.5817	2	1044.5815	0.0002	169	179	0	57.12	37
1410.8321	2	1410.8334	-0.0012	152	164	0	53.63	30
2989.657	3	2989.66	-0.003	67	94	0	145.47	28
968.6011	2	968.6019	-0.0007	143	151	0	72.09	28
1697.9568	2	1697.9563	0.0004	59	75	0	144.4	32
1154.6405	2	1154.6408	-0.0003	192	203	0	65.82	34
1255.6107	2	1255.6118	-0.0011	159	169	1	62.72	36
1360.6327	2	1360.6333	-0.0006	571	581	0	53.34	35
1493.7148	2	1493.7151	-0.0002	68	80	0	60.53	37
2052.0046	2	2052.0052	-0.0005	313	330	0	116.91	38
1287.6609	2	1287.6605	0.0004	95	106	0	81.56	37
1422.6625	2	1422.6627	-0.0002	28	40	0	83	36
1941.9825	2	1941.9836	-0.0011	11	27	0	131.16	37
940.5454	2	940.5454	0.0001	191	199	1	33.81	30
1443.6492	3	1443.6491	0	64	77	0	40.91	34
1571.7435	3	1571.7441	-0.0006	63	77	1	79.14	36
1612.7964	2	1612.7984	-0.002	267	280	1	58.43	37
1653.8923	2	1653.8937	-0.0014	299	313	0	102.14	35
2171.063	2	2171.0667	-0.0037	150	169	0	55.68	37
1112.6439	2	1112.6441	-0.0002	821	831	0	95.57	33
1441.8293	2	1441.8293	0	285	298	0	97.43	33
1814.9333	2	1814.9342	-0.0009	299	313	0	99.52	37
1125.7004	2	1125.7009	-0.0005	280	289	0	83.31	26
1235.6866	2	1235.6874	-0.0008	431	441	0	50.05	34
1325.6978	2	1325.6979	-0.0002	371	381	0	67.36	35
1407.776	2	1407.7762	-0.0002	398	410	0	111.61	34
964.4864	2	964.4865	-0.0002	196	204	0	51.52	37
1244.6745	2	1244.6765	-0.002	100	111	0	63.63	37
1560.7154	2	1560.7168	-0.0015	168	181	0	68.49	35
1656.7367	2	1656.738	-0.0013	61	75	0	54.65	34
1703.8821	2	1703.8842	-0.0021	79	93	0	104.98	37
881.408	2	881.4065	0.0014	226	232	0	46.32	35
1240.6458	2	1240.6551	-0.0093	17	27	0	52.44	35
1265.6648	2	1265.6656	-0.0007	28	39	0	50.17	36
1406.6046	2	1406.6063	-0.0016	278	290	0	82.47	32
1736.8396	2	1736.8403	-0.0007	291	305	0	118.65	37
1217.665	2	1217.6656	-0.0006	350	359	0	52.09	37
1371.7801	2	1371.7802	-0.0001	70	80	0	58.43	34
1614.8982	2	1614.8981	0.0001	81	95	0	112.62	35
2326.2605	3	2326.2606	-0.0001	284	304	0	50.05	33

2521.2939	3	2520.2934	1.0005	208	229	0	59.83	36
1233.5588	2	1233.5587	0.0001	211	220	0	39.82	35
2034.9742	2	2034.9779	-0.0037	104	122	0	93.45	37
2677.2518	2	2677.2541	-0.0023	501	524	0	107.32	36
1181.7021	2	1181.702	0.0002	74	85	0	60.29	30
1228.6913	2	1228.6914	-0.0001	282	292	0	83.6	36
1279.7603	2	1279.7612	-0.0009	157	169	0	79.81	28
1551.7472	2	1551.749	-0.0019	538	551	0	73.44	38
2594.417	3	2594.4207	-0.0037	293	316	0	31.12	30
1407.7588	2	1407.7584	0.0004	318	329	0	54.81	35
1852.0557	2	1852.0557	0	348	364	0	140.17	30
1542.7605	2	1542.7606	-0.0001	356	368	0	104.6	37
1682.946	2	1682.9454	0.0006	133	147	0	111.67	32
1217.665	2	1217.6656	-0.0006	350	359	0	52.09	37
1371.7801	2	1371.7802	-0.0001	70	80	0	58.43	34
1614.8982	2	1614.8981	0.0001	81	95	0	112.62	35
1865.1028	2	1865.1026	0.0002	852	869	0	45.93	23
2326.2605	3	2326.2606	-0.0001	284	304	0	50.05	33
905.4971	2	905.4971	0.0001	518	525	0	55.38	37
1254.7011	2	1254.7013	-0.0002	409	418	0	54.71	33
1703.9824	2	1703.9821	0.0003	318	332	0	119.9	27
2186.1609	3	2186.1623	-0.0014	533	550	1	47.15	35
2919.6038	3	2919.6045	-0.0007	844	870	0	42.86	30
2280.1544	3	2279.1533	1.0011	88	109	0	75.16	37
2315.2036	3	2315.2042	-0.0006	506	527	0	80.78	36
3134.5366	3	3134.5442	-0.0076	325	354	0	50.45	36
1264.5773	2	1264.5791	-0.0019	174	184	0	53.04	36
1407.6793	2	1407.6783	0.001	1973	1984	0	82.4	38
1944.0308	2	1944.0316	-0.0008	2070	2087	0	78.92	36
2333.2368	3	2333.2379	-0.0011	265	287	0	61.92	35
879.446	2	879.445	0.001	228	235	0	40.93	37
936.5503	2	936.5505	-0.0001	162	169	0	46.99	30
982.5351	2	982.5349	0.0003	11	19	0	59.53	35
1032.557	2	1032.5564	0.0007	344	352	0	46.33	37
1248.6273	2	1248.6272	0.0001	215	225	0	44.1	38
1251.5808	2	1251.5812	-0.0004	195	204	0	45.64	36
1319.6587	2	1319.6622	-0.0035	362	373	0	59.22	38
1595.6874	2	1595.6886	-0.0012	291	304	0	68.3	32
1767.8897	3	1767.8904	-0.0007	101	115	0	38.75	37
961.5233	2	961.5233	0.0001	322	329	0	71.34	37
1038.6066	2	1038.6073	-0.0007	290	298	0	38.51	29
1380.7126	2	1380.7136	-0.001	30	42	0	63.73	37
1548.8417	3	1548.8412	0.0005	309	321	1	44.29	35
2161.9041	3	2161.907	-0.0029	91	111	0	50.46	28
2389.2278	3	2389.2278	0	61	83	0	79.52	37
1384.7921	2	1384.7926	-0.0005	415	427	0	77.41	34
2066.092	2	2066.0936	-0.0015	648	666	0	117.08	35
1070.6333	2	1070.6336	-0.0002	11	21	0	68.33	34
1315.6525	2	1315.6521	0.0005	59	69	0	61.34	37
1707.8722	2	1707.8719	0.0003	138	153	0	42.15	37
2154.9994	2	2154.9997	-0.0003	28	46	0	120.05	36
1130.6335	2	1130.6335	0	239	249	0	73.56	36
1190.6903	2	1190.6911	-0.0008	211	220	0	48.94	30
1264.6399	2	1264.6412	-0.0013	152	163	0	54.82	38
1872.9306	2	1872.933	-0.0024	221	238	0	89.82	37
3213.7267	3	3213.7285	-0.0018	250	277	0	42.8	31
1022.5844	2	1022.5834	0.001	26	34	0	55.34	32
1059.5497	2	1059.5502	-0.0005	79	86	0	39.22	38

2100.0498	2	2100.0521	-0.0022	56	75	0	112.07	37
1192.6106	3	1192.6102	0.0005	48	58	0	58.32	38
1403.7902	2	1402.7932	0.997	150	162	0	69.01	34
1630.8238	2	1630.8236	0.0003	171	185	0	116.63	38
880.5749	2	880.5746	0.0004	266	274	1	32.06	20
1301.6144	2	1301.614	0.0004	247	258	0	57.9	35
1333.7233	2	1333.7242	-0.0008	49	61	0	90.31	37
2191.1244	2	2191.126	-0.0016	71	91	0	94.89	37
1212.6717	2	1212.6714	0.0003	296	306	0	82.57	35
1587.7841	2	1587.7852	-0.0012	228	243	0	98.02	37
1767.7786	3	1767.7787	-0.0002	131	145	0	59.44	34
1372.6773	2	1372.6769	0.0004	133	144	0	73.53	37
1393.6487	2	1393.6489	-0.0002	264	274	0	40.91	37
1420.5996	2	1420.6003	-0.0007	243	254	0	47.17	31
1672.8893	2	1671.8831	1.0062	94	108	1	63.83	37
1747.9105	2	1747.9138	-0.0033	42	58	0	82.62	37
1357.7706	2	1357.7704	0.0002	448	459	0	90.12	34
2206.174	2	2206.1732	0.0008	815	834	0	88.59	35
1302.6668	2	1302.668	-0.0013	1248	1259	0	57.97	38
1434.7358	2	1434.7354	0.0004	1132	1144	0	67.73	38
2039.1102	2	2039.1092	0.0011	520	536	0	63.54	34
2660.433	3	2660.4326	0.0004	414	437	0	65.49	32
1243.7749	2	1243.7751	-0.0002	344	355	0	89.37	23
1293.7111	2	1293.7115	-0.0004	401	411	0	62.1	34
1884.0717	2	1884.072	-0.0003	289	305	0	76.57	30
912.5546	2	912.5545	0.0001	379	385	0	40.87	31
1673.8918	3	1673.893	-0.0012	21	33	0	73.73	37
1819.8018	3	1819.8047	-0.0028	64	78	0	40.44	34
2307.1114	3	2307.1131	-0.0017	327	346	1	38.01	37
2480.3242	2	2480.3274	-0.0032	88	110	0	76.01	33
1308.7441	2	1308.7442	-0.0001	146	157	0	81.7	32
1358.7812	2	1358.7809	0.0003	101	112	0	35.71	32
1652.805	2	1652.8046	0.0004	131	143	0	81.4	37
2235.1227	3	2235.1212	0.0016	365	382	0	43.12	37
2607.3154	3	2607.3156	-0.0001	286	308	0	57.45	37
1731.8839	2	1731.8832	0.0008	41	56	0	72.22	37
1945.9477	2	1945.9495	-0.0019	165	181	0	98.15	37
2187.2477	3	2187.2487	-0.001	97	115	1	58.54	27
869.5334	2	869.5334	-0.0001	236	243	0	59.29	30
1164.6178	2	1164.6179	-0.0001	307	316	0	52.87	37
1380.6507	2	1380.6521	-0.0014	608	619	0	57.03	37
1460.7616	2	1460.7623	-0.0007	185	197	0	89.49	38
754.5067	2	754.5065	0.0002	102	107	0	42.9	20
773.5167	2	773.5163	0.0004	83	88	0	43.35	22
1505.7663	2	1505.7667	-0.0004	197	208	0	58.14	38
1556.8382	2	1556.841	-0.0028	161	175	0	109.33	36
914.5072	2	914.5073	-0.0001	372	379	0	45.33	35
1250.6661	2	1250.6659	0.0002	444	453	0	69.56	36
1888.9271	2	1888.924	0.0031	109	125	0	102.95	38
3191.551	3	3191.551	-0.0001	220	248	0	39.17	37
1367.7772	2	1367.7773	-0.0001	24	35	0	81.81	32
1370.7768	2	1370.7769	-0.0001	53	64	0	115.27	35
1201.682	2	1201.6819	0.0001	375	385	0	58	36
1534.9449	2	1534.9447	0.0003	810	823	0	80.16	20
2058.1172	2	2058.1183	-0.0011	1687	1706	0	76.07	34
1056.6286	2	1056.6292	-0.0005	57	67	0	66.81	34
1070.5972	2	1070.5972	0.0001	44	54	0	43.28	34
1253.6875	2	1253.688	-0.0005	174	185	0	54.92	34

1413.7251	2	1413.7252	-0.0001	156	168	0	87.59	36
869.4717	2	869.4719	-0.0002	1417	1424	0	52.3	33
1401.7477	2	1401.7504	-0.0027	360	371	0	66.92	38
1781.0047	2	1781.0047	0	1464	1479	0	54.01	31
2407.2914	3	2407.292	-0.0006	2201	2222	0	57.02	33
1212.5548	2	1212.5557	-0.0009	153	162	0	49.45	35
1618.8814	2	1618.8831	-0.0017	200	213	0	81.23	35
1749.7869	3	1749.788	-0.0011	228	243	0	59.65	35
2056.9582	2	2056.9623	-0.004	244	262	1	70.36	36
927.539	2	927.5389	0.0001	239	246	0	48.84	33
1071.6692	2	1071.6692	0	153	162	0	31.68	28
1084.6396	2	1084.6393	0.0003	548	556	0	52.57	34
1256.6361	2	1256.6401	-0.004	569	579	0	49.58	36
1335.7647	2	1335.765	-0.0002	339	350	0	65.79	30
2153.0862	3	2153.0865	-0.0003	519	540	0	63.89	37
1437.8565	2	1437.8555	0.001	117	130	0	36.43	27
1643.8516	2	1643.8519	-0.0003	165	178	0	94.88	37
1666.7724	2	1666.772	0.0005	140	154	0	92.69	37
1209.6752	2	1209.6717	0.0035	5	15	0	36.92	33
1265.6971	2	1265.6979	-0.0009	299	310	0	41.59	34
2276.3052	2	2276.3104	-0.0051	506	526	0	58.76	26
2390.2502	2	2390.2515	-0.0013	163	183	0	104.52	36
755.4533	2	755.4541	-0.0008	2	9	0	34.89	30
1161.6385	2	1161.6394	-0.0008	12	22	0	67.59	35
1315.6525	2	1315.6521	0.0005	60	70	0	61.34	37
1479.7576	2	1479.7569	0.0007	81	94	0	95.8	38
1070.6333	2	1070.6336	-0.0002	18	28	0	68.33	34
1163.5814	2	1163.5822	-0.0009	144	154	0	42.76	38
1315.6525	2	1315.6521	0.0005	66	76	0	61.34	37
1609.8351	2	1609.8352	-0.0001	162	175	0	86.09	37
1161.6134	2	1161.6142	-0.0008	161	171	0	78.13	38
1904.9932	2	1904.9918	0.0015	198	215	0	111.74	37
842.4862	2	842.4862	0.0001	2	10	0	54.23	34
1180.6927	2	1180.6928	-0.0001	129	139	0	82.26	30
1549.7545	2	1549.7559	-0.0013	115	128	0	84.08	38
759.4283	2	759.4279	0.0004	23	30	0	40.89	40
943.5337	2	943.5338	-0.0001	246	253	0	41.25	37
963.5391	2	963.5389	0.0002	222	230	0	54.3	34
1224.727	2	1224.7264	0.0006	40	49	0	35.28	31
1365.6704	2	1365.6711	-0.0007	175	187	0	85.32	38
1614.8398	2	1614.8399	-0.0001	26	40	0	99.35	37
1775.8694	2	1775.8724	-0.003	41	57	0	85.51	38
999.5701	2	999.5713	-0.0012	268	276	0	43.27	36
1270.6978	2	1270.6993	-0.0015	93	104	0	65.52	36
1284.7111	2	1284.7112	0	177	188	0	49.76	35
1414.7557	2	1414.7569	-0.0012	254	267	0	99.18	37
1087.5696	2	1087.5696	0.0001	377	386	0	42.58	38
1241.6287	2	1241.6292	-0.0005	412	422	0	72.71	36
1481.8608	2	1481.8606	0.0002	278	291	0	85.61	29
1690.8195	3	1690.8209	-0.0014	254	268	0	43.6	38
1085.5863	2	1085.5869	-0.0006	877	885	0	41.04	36
1524.8985	2	1524.8988	-0.0002	1069	1083	0	84.91	29
1940.051	2	1940.052	-0.001	810	827	0	85.61	34
1465.7559	2	1465.7565	-0.0006	97	108	0	71.86	37
1567.7165	2	1567.7154	0.0011	83	96	0	61.4	35
1986.979	2	1986.9795	-0.0005	234	249	0	80.7	37
1757.9779	2	1757.9774	0.0005	73	88	0	117.98	34
2010.1446	3	2010.1441	0.0005	56	72	0	49.6	30

943.4761	2	943.4763	-0.0002	77	84	0	40.15	34
1042.6022	2	1042.6023	-0.0001	16	26	0	38.71	35
1079.5611	2	1079.5611	-0.0001	44	53	0	57.83	37
1209.6348	2	1209.6353	-0.0006	132	142	0	65.88	36
1332.7445	2	1332.7402	0.0043	85	97	0	84.84	34
1044.5267	2	1044.5274	-0.0007	375	383	0	45.49	37
1061.5406	2	1061.5393	0.0013	285	293	0	41.49	38
1087.639	2	1087.639	0	150	158	0	61.47	33
1688.8004	2	1688.8005	-0.0001	403	417	0	84.07	36
2199.061	2	2199.0616	-0.0006	194	213	0	56.33	37
1265.7346	2	1265.7343	0.0003	343	353	0	44.37	30
1512.8147	2	1512.8147	-0.0001	259	272	0	121.91	35
1702.8324	2	1702.8348	-0.0024	327	342	0	39.3	37
1243.7122	2	1243.7136	-0.0013	691	702	0	63.7	35
1483.9014	2	1483.9014	0.0001	433	446	0	42.55	25
1857.9297	3	1857.9295	0.0003	540	555	0	46.49	37
1975.9391	2	1975.9374	0.0017	84	100	0	78.65	37
951.5009	2	951.5025	-0.0016	76	84	0	44.4	35
1425.7563	2	1425.7576	-0.0012	132	144	0	66.21	36
1759.8934	2	1759.8952	-0.0017	9	25	0	95.64	37
871.4763	2	871.4763	0	608	615	0	43.25	35
1518.7833	2	1518.7831	0.0003	240	252	0	80.39	38
1551.8253	3	1551.8257	-0.0003	24	37	0	40.51	35
2089.0501	3	2088.0487	1.0014	217	234	1	72.54	37
2216.0816	4	2215.0804	1.0012	69	87	1	49.68	37
2585.2742	2	2585.2749	-0.0006	88	111	0	124.58	37
980.5698	2	980.5695	0.0003	100	107	0	36.89	31
1064.5497	2	1064.5502	-0.0005	55	63	0	61.05	37
1066.6127	2	1066.6135	-0.0008	64	72	0	63.15	33
1762.8803	2	1762.8836	-0.0033	12	27	0	77.44	38
754.5067	2	754.5065	0.0002	102	107	0	42.9	20
773.5167	2	773.5163	0.0004	83	88	0	43.35	22
1146.6033	2	1146.6033	0	231	239	0	56.41	39
1505.7663	2	1505.7667	-0.0004	197	208	0	58.14	38
2428.256	4	2428.2573	-0.0013	176	196	0	52.1	36
1340.7553	2	1340.7565	-0.0011	511	523	0	78.53	34
1630.8684	2	1630.8679	0.0006	227	242	0	92.16	36
1228.5714	2	1228.5724	-0.001	499	509	0	45.78	35
1228.7174	2	1228.718	-0.0006	593	603	0	43.09	34
1395.7244	2	1395.7245	-0.0001	604	616	0	50.61	37
1885.9156	2	1885.917	-0.0014	709	725	0	72.85	37
1982.0208	3	1982.0208	0.0001	405	423	0	50.86	36
1256.6976	2	1256.6976	0	167	177	0	58.62	36
1289.6094	2	1289.6099	-0.0005	425	436	0	71.03	36
2249.2868	2	2249.2882	-0.0014	289	310	0	53.21	27
1847.9612	2	1847.9629	-0.0017	59	75	0	109.53	37
1879.7479	3	1879.7499	-0.002	206	222	0	27.67	25
3139.6261	3	3138.6231	1.003	159	187	0	40.34	34
939.5758	2	939.5753	0.0005	125	132	0	51.68	28
2080.9262	3	2080.9273	-0.0011	107	124	0	49.59	34
2192.0208	2	2192.0242	-0.0033	140	159	0	88.13	36
1056.654	2	1056.6543	-0.0003	282	291	0	72.5	29
1088.5438	2	1088.5462	-0.0024	292	302	0	50.37	38
1127.594	2	1127.5934	0.0005	265	275	0	61.76	35
1130.5561	2	1130.5567	-0.0007	227	236	0	44.56	36
1053.5553	2	1053.5567	-0.0014	106	116	0	74.17	35
1153.6446	2	1153.6455	-0.0009	11	21	0	56.1	32
1442.7225	2	1442.7253	-0.0027	128	140	0	67.91	37

899.5072	2	899.5076	-0.0004	114	123	0	39.32	34
2004.9606	2	2004.9609	-0.0002	16	35	0	126.7	37
1003.5817	2	1003.5814	0.0003	471	479	0	62.01	34
1880.9327	2	1880.9342	-0.0015	445	460	0	102.39	37
1319.6185	2	1319.6205	-0.0019	880	893	0	106.47	36
2358.2476	3	2358.2471	0.0005	946	966	1	50.56	35
1510.8358	3	1510.8355	0.0003	174	188	0	55.96	34
2627.3097	3	2626.3027	1.007	120	143	0	104.97	37
1109.498	2	1109.4989	-0.0009	690	699	0	57.14	35
1155.6212	2	1155.6248	-0.0036	942	953	0	38.32	34
1234.6305	2	1234.6306	-0.0001	1077	1087	0	39.99	39
1324.6627	2	1324.6623	0.0004	1041	1052	0	55.26	37
1640.7506	3	1640.7503	0.0003	771	784	1	37.33	35
3064.6714	3	3063.6564	1.015	1171	1203	0	50.07	29
839.5115	2	839.5116	-0.0001	354	361	0	40.99	28
1064.5905	2	1064.5906	-0.0001	15	23	0	52.56	34
1442.6546	2	1442.6565	-0.0019	69	80	0	67.64	34
3347.6907	4	3346.6908	0.9999	269	297	1	55.02	35
1138.7072	2	1138.7074	-0.0002	831	840	0	68.19	26
1414.7826	2	1414.782	0.0006	718	729	0	88.7	35
2626.0497	3	2626.053	-0.0033	42	65	0	73.82	25
2901.2148	3	2901.2164	-0.0015	40	65	1	68.02	29
1105.7585	2	1105.7587	-0.0002	381	390	0	48.89	20
1752.9669	2	1752.9662	0.0007	454	469	0	101.47	33
1255.7499	2	1255.75	0	4357	4368	0	91.61	32
1998.1158	3	1998.115	0.0008	1319	1336	0	54.32	31
1202.6758	2	1202.6772	-0.0013	305	315	0	68.51	35
1626.808	2	1626.8076	0.0004	330	343	0	91.85	37
953.5913	2	953.591	0.0004	389	397	0	32.63	29
1205.5715	2	1205.5717	-0.0002	435	444	0	54.53	37
2220.0588	2	2220.06	-0.0012	300	322	0	94.73	37
1242.7174	2	1242.7183	-0.001	194	205	0	73.59	34
1391.6534	2	1391.6537	-0.0003	43	54	0	82.45	36
1403.6524	2	1403.6528	-0.0004	441	453	0	89.02	35
1411.7418	2	1411.7419	-0.0001	467	480	0	66.08	36
939.6114	2	939.6117	-0.0002	861	868	0	49.9	22
2139.1567	3	2139.1576	-0.0008	1496	1515	0	82.41	34
1091.5983	2	1091.5975	0.0008	6	15	0	46.8	36
3074.5585	3	3074.5594	-0.0009	194	222	0	107.96	36
1284.7702	2	1284.7693	0.0008	2912	2922	0	51.82	30
1550.9068	2	1550.9072	-0.0004	1927	1940	0	94.3	27
1194.6397	2	1194.6397	0	341	351	0	95.89	36
2503.2335	3	2503.2329	0.0005	725	747	0	55.66	37
996.608	2	996.608	0	10	18	0	41.49	28
2162.1112	2	2162.1106	0.0006	198	216	0	56.21	36
2579.3002	2	2579.3006	-0.0004	152	173	0	57.28	37
1122.5609	2	1122.5591	0.0019	610	620	0	41.45	37
1232.6881	2	1232.6877	0.0004	397	408	0	52.63	36
1881.0978	2	1881.0975	0.0003	147	164	0	69.34	27
1268.7703	2	1268.7704	-0.0001	155	165	0	69.76	29
1395.6914	2	1395.6922	-0.0008	375	386	0	79.39	37
980.5093	2	980.5113	-0.0021	67	74	0	42.75	37
1104.5844	2	1104.5849	-0.0005	78	87	0	44.96	38
1531.8229	2	1531.8246	-0.0017	38	51	0	94.8	37
1426.7917	2	1426.7919	-0.0002	260	272	0	105.81	35
1578.8009	2	1578.7963	0.0046	362	376	0	41.02	38
3201.6715	3	3201.6731	-0.0016	54	84	0	37.87	33
1153.7179	2	1153.7183	-0.0004	129	139	0	76.7	27

1274.6103	2	1274.6102	0.0001	58	69	0	70.05	37
1471.7792	2	1471.7783	0.0009	28	40	0	84.28	37
2406.2938	3	2406.2934	0.0004	46	65	1	58.13	33
1158.7376	2	1158.7376	0	442	452	0	89.21	20
1381.7695	2	1381.7678	0.0018	118	132	0	49.52	33
1488.8192	2	1488.8188	0.0004	26	38	0	92.79	36
2123.1436	2	2123.1449	-0.0012	177	194	0	48.24	34
1055.5977	2	1055.5975	0.0003	188	197	0	45.14	33
1739.9583	2	1739.957	0.0013	320	336	0	97.23	33
979.5336	2	979.5338	-0.0002	383	391	0	42.3	35
1293.6532	2	1293.6466	0.0066	132	144	0	60.13	38
1987.9575	3	1987.9581	-0.0006	116	131	0	50.64	37
2841.4806	3	2841.4813	-0.0007	485	510	0	50.87	35
1072.6235	2	1072.6241	-0.0005	42	52	0	52.07	35
1334.7667	2	1334.767	-0.0003	276	287	0	69.05	31
1972.1259	4	1972.1258	0	17	33	0	32.3	29
3286.7715	4	3286.7714	0.0001	108	136	1	41.83	31
1235.7278	2	1235.7278	0	229	239	0	67.57	28
1239.6826	2	1239.6823	0.0003	192	202	0	46.21	34
1906.1033	3	1906.104	-0.0007	128	146	1	49.2	26
1297.7486	2	1297.7493	-0.0007	617	628	0	49.72	32
1424.8025	2	1424.8027	-0.0002	661	672	0	72.07	33
2319.2024	3	2319.1998	0.0025	581	600	0	45.54	36
1159.619	2	1159.6197	-0.0007	50	61	0	78.3	38
1165.5981	2	1165.5979	0.0002	66	76	0	65.53	37
958.5346	2	958.5335	0.0011	519	527	0	43.66	37
1562.7143	2	1561.7123	1.002	355	367	0	96.28	36
1331.6496	2	1331.6503	-0.0007	27	37	0	42.11	37
1449.7531	2	1449.7537	-0.0006	74	85	0	98.03	38
1788.0285	2	1788.0284	0.0001	760	775	0	80.67	29
2204.2875	3	2204.2892	-0.0018	376	395	1	29.6	24
1225.7021	2	1225.703	-0.0009	469	480	0	77.17	33
1367.6201	2	1367.6214	-0.0012	323	333	0	52.98	36
1138.572	2	1138.5731	-0.0011	973	981	0	54.62	36
1172.6653	2	1172.6652	0.0001	922	931	0	58.01	36
1872.8406	2	1872.8391	0.0015	582	597	0	48.15	34
1583.8071	2	1583.8083	-0.0012	226	239	0	50.84	37
1598.8874	2	1598.8879	-0.0006	351	365	0	78.71	34
1463.7087	2	1463.7085	0.0002	605	615	0	57.38	37
2371.142	3	2371.1406	0.0014	630	650	0	50.64	37
1210.7274	2	1210.7285	-0.0011	662	672	0	49.7	29
1282.6302	2	1282.6306	-0.0004	378	389	0	60.17	37
1629.8801	2	1629.8807	-0.0005	614	626	0	45.9	36
2362.26	2	2362.2607	-0.0006	86	106	0	55.91	34
2561.3913	3	2561.3927	-0.0015	84	106	1	51.18	32
1074.5669	2	1074.5669	0	181	190	1	68.33	38
1352.8279	2	1352.8279	0.0001	226	237	0	56.09	26
1159.6712	2	1159.6713	-0.0001	148	158	0	86.77	34
2940.5295	3	2940.5307	-0.0011	321	348	0	35.42	34
974.5543	2	974.5549	-0.0006	168	178	0	42.36	36
1948.991	2	1948.9894	0.0015	262	277	0	79.89	37
1128.6292	2	1128.6291	0	314	323	0	45.25	36
1506.7464	2	1506.7487	-0.0023	167	179	0	67.33	38
1441.7485	3	1441.7486	-0.0001	311	322	0	43.63	36
2135.0682	2	2135.0674	0.0009	180	198	0	64.11	37
1067.4957	2	1067.4957	0	112	120	0	44.76	35
2851.4923	3	2851.4967	-0.0044	227	253	0	76.46	34
1038.6074	2	1038.6073	0.0001	64	72	0	37.41	29

1322.6543	2	1322.6605	-0.0063	429	439	0	66.65	38
2319.1925	3	2319.1933	-0.0008	245	265	0	42.67	36
1086.5682	2	1086.5709	-0.0027	47	55	0	40	36
1132.649	2	1132.6492	-0.0002	88	97	0	71.25	33
1102.5486	2	1102.5506	-0.002	15	23	1	51.38	37
1825.0394	2	1825.0383	0.0011	101	117	0	71.9	31
981.5649	2	981.5648	0.0002	560	567	0	58.72	32
1295.7551	2	1295.7561	-0.0011	632	643	0	59.19	31
1357.7028	2	1357.7024	0.0004	130	141	0	70.71	37
1697.7678	2	1697.7685	-0.0007	432	445	0	45.65	35
1140.5553	2	1140.5539	0.0014	151	159	0	42.48	35
1151.5886	2	1151.5896	-0.001	162	171	0	68.78	36
1200.6235	2	1200.6237	-0.0003	111	121	0	45.23	37
1406.7282	2	1406.7293	-0.0011	90	102	0	77.49	38
1441.7485	3	1441.7486	-0.0001	311	322	0	43.63	36
1239.6826	2	1239.6823	0.0003	203	213	0	46.21	34
1251.7225	2	1251.7227	-0.0002	240	250	0	43.61	30
1906.1033	3	1906.104	-0.0007	139	157	1	49.2	26
1369.7196	3	1369.7202	-0.0006	209	221	1	36.8	36
3113.665	3	3113.6648	0.0002	450	478	0	40.69	32
3448.9045	3	3448.9069	-0.0024	171	202	1	45.34	27
1012.5678	2	1012.5665	0.0013	43	51	0	38.19	33
1697.8151	2	1697.8148	0.0003	52	66	0	71.85	37
1145.6047	2	1145.604	0.0006	50	61	0	53	38
1165.5981	2	1165.5979	0.0002	66	76	0	65.53	37
1100.6077	2	1100.6077	0	200	209	0	75.42	36
2079.8506	3	2079.852	-0.0014	224	241	0	34.74	28
1037.5611	2	1037.5618	-0.0006	405	414	0	37.58	35
1094.5894	2	1094.5873	0.0021	32	40	0	49.41	36
2627.1676	3	2626.1783	0.9892	54	80	0	42.69	34
2886.3606	3	2886.3613	-0.0007	456	482	0	37.73	36
1380.8454	3	1380.8453	0.0001	43	55	1	44.52	25
1391.6236	2	1391.6239	-0.0002	28	39	0	36.35	34
1915.0202	3	1915.0203	-0.0001	77	93	0	54.42	36
1252.6608	2	1252.6663	-0.0055	58	69	0	45.14	36
1577.7422	2	1577.7436	-0.0014	41	52	0	61.73	36
934.5122	2	934.5124	-0.0001	242	249	0	38.53	35
1128.5711	2	1128.5717	-0.0006	255	264	0	67.8	35
1448.7002	3	1448.7008	-0.0007	221	233	0	39.75	37
1803.9518	2	1803.9519	-0.0001	176	190	0	70.46	37
1216.63	2	1216.6299	0	149	160	0	64.86	38
3073.7413	3	3073.7427	-0.0014	120	148	0	38.04	23
1114.6595	2	1114.6598	-0.0002	838	848	0	50.13	33
1227.6722	3	1227.6724	-0.0002	411	422	0	37.06	36
1612.8526	3	1612.8573	-0.0047	646	659	0	45.7	36
1090.6024	2	1090.6022	0.0002	17	25	0	40.11	35
1402.7819	2	1402.782	-0.0001	144	155	0	64.31	35
1211.6863	2	1211.6874	-0.0011	453	464	0	48.77	33
1367.6201	2	1367.6214	-0.0012	316	326	0	52.98	36
1266.7904	2	1266.7911	-0.0007	72	84	0	54.16	23
1686.796	3	1686.7962	-0.0002	101	115	0	46.45	37
1383.7708	2	1383.7722	-0.0013	264	278	0	39.57	34
1455.7725	2	1455.7722	0.0003	387	399	0	64.97	36
943.4761	2	943.4763	-0.0002	76	83	0	40.15	34
1079.5611	2	1079.5611	-0.0001	43	52	0	57.83	37
1731.9564	3	1731.9559	0.0005	165	179	0	44.04	34
994.6795	2	994.679	0.0005	198	206	0	41.01	20
2730.4019	3	2730.4004	0.0015	93	117	0	54.93	36

1330.6798	2	1330.6803	-0.0004	223	234	0	61.36	38
1717.0753	3	1717.0753	0	316	331	0	39.16	20
957.5246	2	957.5243	0.0003	6	13	1	50.25	36
1075.56	2	1075.5622	-0.0022	260	270	0	55.87	39
1046.5759	2	1046.576	-0.0001	72	80	0	51.82	37
1318.7068	2	1318.7067	0	141	151	0	39.4	37
2941.5059	3	2941.5073	-0.0014	85	109	1	43.15	35
1201.682	2	1201.6819	0.0001	387	397	0	58	36
1729.909	2	1729.908	0.0011	1392	1405	0	39.14	37
1071.6442	2	1071.6441	0.0002	934	943	0	48.23	34
1227.6722	3	1227.6724	-0.0002	411	422	0	37.06	36
1612.8526	3	1612.8573	-0.0047	646	659	0	45.7	36
1084.6125	2	1084.6128	-0.0003	278	287	0	51.55	34
1259.5414	2	1259.5419	-0.0004	304	315	0	48.34	31
956.5537	2	956.5542	-0.0006	126	133	0	45.34	34
1144.5907	2	1144.591	-0.0003	134	142	0	47.66	37
2882.4256	2	2882.4284	-0.0028	94	120	0	37.55	37
804.4502	2	804.4494	0.0008	309	315	0	42.06	37
1072.4832	2	1072.4825	0.0007	280	288	0	45.91	32
1216.6813	2	1216.6815	-0.0003	183	193	0	41.44	36
1678.9405	3	1678.9406	-0.0001	61	76	0	36.58	32
2417.2056	3	2417.2061	-0.0005	745	767	0	44.87	37
2573.3073	3	2573.3072	0.0001	745	768	1	48.65	36
1533.8549	2	1533.8555	-0.0006	542	554	0	60.15	34
2366.3816	3	2366.3824	-0.0008	329	352	0	31.06	21
970.581	2	970.5811	-0.0001	98	106	0	38.36	34
1230.6116	2	1230.6139	-0.0023	33	42	0	59.08	37
1292.6598	2	1292.6612	-0.0014	247	257	0	41.75	38
1367.6201	2	1367.6214	-0.0013	258	268	0	52.98	36
1361.6976	2	1361.698	-0.0004	169	179	0	40.07	38
1387.6914	2	1387.6943	-0.0029	123	135	0	53.12	37
916.444	2	916.4443	-0.0003	490	496	0	48.19	35
1258.6518	2	1258.6517	0.0001	335	345	0	44.32	38
1499.8957	2	1499.8963	-0.0006	131	144	0	40.44	27
1857.9297	3	1857.9295	0.0003	238	253	0	46.49	37
1109.6273	2	1109.6273	0	507	515	0	36.39	32
1279.6811	2	1279.6812	-0.0001	192	203	0	47.43	36
868.5488	2	868.5494	-0.0006	10	17	0	30.45	25
1173.6239	2	1173.6241	-0.0002	93	103	0	53.8	38
1181.6449	2	1181.6444	0.0004	23	32	0	36.21	35
3078.5245	3	3078.5294	-0.0049	134	161	1	49.64	37
770.4535	2	770.4538	-0.0002	51	57	0	35.16	33
1522.7184	3	1522.7198	-0.0014	92	104	0	50.49	37
949.5231	2	949.5233	-0.0002	92	99	0	37.45	35
2372.193	3	2372.1934	-0.0004	118	139	0	39.54	37
2774.5106	3	2774.5113	-0.0007	342	367	0	33.06	31
770.4535	2	770.4538	-0.0002	51	57	0	35.16	33
1506.7235	3	1506.7249	-0.0014	92	104	0	41.23	37
997.58	2	997.5808	-0.0008	394	402	1	31.46	30
1642.9542	2	1642.9545	-0.0004	828	842	0	31.39	29

pep_exp_mr	pep_exp_z	pep_calc_mr	pep_delta	pep_start	pep_end	pep_miss	pep_score	pep_ident
975.4689	2	975.4695	-0.0007	351	359	0	77.66	37
1026.5479	2	1026.5498	-0.0019	310	318	0	71.82	34
1064.4188	2	1064.4201	-0.0013	298	306	0	33.07	26
1138.6858	2	1138.6862	-0.0004	253	262	0	73.82	28
1145.5818	2	1145.5829	-0.0011	242	251	0	44.4	38
1230.5696	2	1230.5703	-0.0007	381	390	0	71.7	35
1273.6776	2	1273.6779	-0.0003	242	252	1	44.7	38

1370.6757	2	1370.6765	-0.0008	380	390	1	52.99	36
1433.6391	2	1433.6393	-0.0003	163	174	0	95.58	34
1477.653	2	1477.6541	-0.001	325	336	0	73.42	34
1621.844	2	1621.8439	0.0001	263	276	0	89.78	37
1678.789	2	1678.7906	-0.0016	63	77	0	72.6	37
1695.826	2	1695.8257	0.0004	337	350	0	80.01	37
1813.8994	2	1813.8999	-0.0005	47	62	1	81.3	37
1821.9157	2	1821.9156	0.0001	3	19	0	110.72	38
1950.8802	2	1950.8815	-0.0013	363	379	0	119.49	35
1971.9883	2	1971.9902	-0.0019	104	121	0	161.16	37
2100.0824	2	2100.0851	-0.0027	104	122	1	136.44	37
2110.0562	3	2109.0571	0.9991	1	19	1	59.63	37
2366.118	2	2366.1212	-0.0032	277	297	0	67.55	36
2763.3128	3	2763.3143	-0.0014	217	241	0	106.25	37
2797.3283	3	2797.3361	-0.0078	78	103	1	77.35	36
3200.4665	4	3199.4669	0.9995	20	46	0	39.48	35
3325.498	3	3325.5014	-0.0034	123	154	0	55.03	34
4573.1092	3	4572.1135	0.9957	175	213	0	37.76	33
1026.5479	2	1026.5498	-0.0019	310	318	0	71.82	34
1064.4188	2	1064.4201	-0.0013	298	306	0	33.07	26
1138.6858	2	1138.6862	-0.0004	253	262	0	73.82	28
1145.5818	2	1145.5829	-0.0011	242	251	0	44.4	38
1230.5696	2	1230.5703	-0.0007	381	390	0	71.7	35
1273.6776	2	1273.6779	-0.0003	242	252	1	44.7	38
1297.6297	2	1297.6302	-0.0006	47	58	0	54.04	36
1370.6757	2	1370.6765	-0.0008	380	390	1	52.99	36
1433.6391	2	1433.6393	-0.0003	163	174	0	95.58	34
1621.844	2	1621.8439	0.0001	263	276	0	89.78	37
1678.789	2	1678.7906	-0.0016	63	77	0	72.6	37
1695.826	2	1695.8257	0.0004	337	350	0	80.01	37
1821.9157	2	1821.9156	0.0001	3	19	0	110.72	38
1950.8802	2	1950.8815	-0.0013	363	379	0	119.49	35
1971.9883	2	1971.9902	-0.0019	104	121	0	161.16	37
2100.0824	2	2100.0851	-0.0027	104	122	1	136.44	37
2110.0562	3	2109.0571	0.9991	1	19	1	59.63	37
2366.118	2	2366.1212	-0.0032	277	297	0	67.55	36
2763.3128	3	2763.3143	-0.0014	217	241	0	106.25	37
2797.3283	3	2797.3361	-0.0078	78	103	1	77.35	36
3150.3817	3	3149.3819	0.9998	20	46	0	102.02	32
3398.4952	3	3398.5	-0.0048	123	154	0	71.48	31
4573.1092	3	4572.1135	0.9957	175	213	0	37.76	33
1064.4188	2	1064.4201	-0.0013	298	306	0	33.07	26
1138.6858	2	1138.6862	-0.0004	253	262	0	73.82	28
1145.5818	2	1145.5829	-0.0011	242	251	0	44.4	38
1230.5696	2	1230.5703	-0.0007	381	390	0	71.7	35
1273.6776	2	1273.6779	-0.0003	242	252	1	44.7	38
1286.6536	2	1286.6541	-0.0005	351	362	0	43.73	37
1295.6005	2	1295.5994	0.0012	36	46	0	80.86	36
1370.6757	2	1370.6765	-0.0008	380	390	1	52.99	36
1383.6944	2	1383.6931	0.0014	163	174	0	81.19	37
1444.6407	2	1444.6405	0.0002	47	58	0	102.65	33
1445.6811	2	1445.682	-0.001	325	336	0	82.73	36
1621.844	2	1621.8439	0.0001	263	276	0	89.78	37
1678.789	2	1678.7906	-0.0016	63	77	0	72.6	37
1695.826	2	1695.8257	0.0004	337	350	0	80.01	37
1777.856	2	1777.8557	0.0003	283	297	0	82.48	38
1821.9157	2	1821.9156	0.0001	3	19	0	110.72	38
1871.8616	2	1871.8625	-0.0009	20	35	0	86.04	36

1950.8802	2	1950.8815	-0.0013	363	379	0	119.49	35
1971.9883	2	1971.9902	-0.0019	104	121	0	161.16	37
2100.0824	2	2100.0851	-0.0027	104	122	1	136.44	37
2110.0562	3	2109.0571	0.9991	1	19	1	59.63	37
2750.3174	3	2750.319	-0.0017	217	241	0	59.81	37
2801.2925	3	2801.2946	-0.0022	78	103	1	95.57	35
3398.4952	3	3398.5	-0.0048	123	154	0	71.48	31
4573.1092	3	4572.1135	0.9957	175	213	0	37.76	33
1064.4188	2	1064.4201	-0.0013	298	306	0	33.07	26
1138.6858	2	1138.6862	-0.0004	253	262	0	73.82	28
1145.5818	2	1145.5829	-0.0011	242	251	0	44.4	38
1230.5696	2	1230.5703	-0.0007	381	390	0	71.7	35
1273.6776	2	1273.6779	-0.0003	242	252	1	44.7	38
1286.6536	2	1286.6541	-0.0005	351	362	0	43.73	37
1307.6351	2	1307.6357	-0.0006	36	46	0	74.91	38
1370.6757	2	1370.6765	-0.0008	380	390	1	52.99	36
1430.6242	2	1430.6248	-0.0006	47	58	0	88.53	32
1433.6391	2	1433.6393	-0.0003	163	174	0	95.58	34
1445.6811	2	1445.682	-0.001	325	336	0	82.73	36
1621.844	2	1621.8439	0.0001	263	276	0	89.78	37
1632.7854	2	1632.7851	0.0003	63	77	0	66.07	37
1695.826	2	1695.8257	0.0004	337	350	0	80.01	37
1772.8785	2	1772.8767	0.0018	283	297	0	38.68	38
1898.9269	2	1898.9275	-0.0006	20	35	0	111.98	37
1950.8802	2	1950.8815	-0.0013	363	379	0	119.49	35
1971.9883	2	1971.9902	-0.0019	104	121	0	161.16	37
2100.0824	2	2100.0851	-0.0027	104	122	1	136.44	37
2736.303	3	2736.3034	-0.0004	217	241	0	63.4	37
2813.3283	3	2813.331	-0.0027	78	103	1	97.05	36
3398.4952	3	3398.5	-0.0048	123	154	0	71.48	31
4573.1092	3	4572.1135	0.9957	175	213	0	37.76	33
1064.4188	2	1064.4201	-0.0013	298	306	0	33.07	26
1138.6858	2	1138.6862	-0.0004	253	262	0	73.82	28
1145.5818	2	1145.5829	-0.0011	242	251	0	44.4	38
1230.5696	2	1230.5703	-0.0007	381	390	0	71.7	35
1273.6776	2	1273.6779	-0.0003	242	252	1	44.7	38
1286.6536	2	1286.6541	-0.0005	351	362	0	43.73	37
1363.6365	2	1363.6368	-0.0002	36	46	0	57.24	37
1370.6757	2	1370.6765	-0.0008	380	390	1	52.99	36
1383.6944	2	1383.6931	0.0014	163	174	0	81.19	37
1430.6242	2	1430.6248	-0.0006	47	58	0	88.53	32
1445.6811	2	1445.682	-0.001	325	336	0	82.73	36
1621.844	2	1621.8439	0.0001	263	276	0	89.78	37
1632.7854	2	1632.7851	0.0003	63	77	0	66.07	37
1695.826	2	1695.8257	0.0004	337	350	0	80.01	37
1821.9157	2	1821.9156	0.0001	3	19	0	110.72	38
1950.8802	2	1950.8815	-0.0013	363	379	0	119.49	35
1971.9883	2	1971.9902	-0.0019	104	121	0	161.16	37
2100.0824	2	2100.0851	-0.0027	104	122	1	136.44	37
2110.0562	3	2109.0571	0.9991	1	19	1	59.63	37
2750.3174	3	2750.319	-0.0017	217	241	0	59.81	37
2813.3283	3	2813.331	-0.0027	78	103	1	97.05	36
3398.4952	3	3398.5	-0.0048	123	154	0	71.48	31
4573.1092	3	4572.1135	0.9957	175	213	0	37.76	33
908.4956	2	908.4967	-0.0011	157	164	1	46.2	33
986.5397	2	986.5397	0.0001	327	336	0	44.67	37
1282.4962	2	1282.4967	-0.0005	312	320	0	53.32	24
1379.6899	3	1379.6907	-0.0008	391	401	1	41.64	38

1472.8563	2	1472.8562	0	230	243	0	98.59	30
1597.7599	2	1597.7599	-0.0001	340	352	0	80.05	37
1689.8787	2	1689.8798	-0.0011	216	229	1	47.46	37
1714.9134	2	1714.9142	-0.0008	65	79	0	90.85	36
1791.9213	2	1791.9229	-0.0016	265	280	0	102.14	38
1856.8744	2	1856.8761	-0.0016	374	390	0	121.23	37
1884.9388	2	1884.9404	-0.0016	353	370	0	65.51	37
1887.95	3	1887.9513	-0.0013	106	121	1	56.5	37
1976.8742	2	1976.8752	-0.001	41	60	0	117.83	33
2279.1778	3	2279.1798	-0.0019	65	84	1	36.93	36
2345.004	3	2345.0059	-0.0019	403	422	0	55.38	32
2384.1854	2	2384.1873	-0.0018	85	105	1	96.16	37
2394.1838	2	2394.1856	-0.0017	244	264	0	125.3	38
2501.1031	3	2501.107	-0.0039	402	422	1	114.63	33
2640.2217	3	2640.22	0.0018	281	304	0	72.04	36
3192.6214	3	3192.6238	-0.0024	125	156	0	98.9	35
3320.7196	3	3320.7187	0.0008	124	156	1	120.75	34
908.4956	2	908.4967	-0.0011	157	164	1	46.2	33
986.5397	2	986.5397	0.0001	327	336	0	44.67	37
1282.4962	2	1282.4967	-0.0005	312	320	0	53.32	24
1379.6899	3	1379.6907	-0.0008	391	401	1	41.64	38
1472.8563	2	1472.8562	0	230	243	0	98.59	30
1597.7599	2	1597.7599	-0.0001	340	352	0	80.05	37
1662.866	2	1662.8689	-0.0029	216	229	1	40.28	37
1714.9134	2	1714.9142	-0.0008	65	79	0	90.85	36
1791.9213	2	1791.9229	-0.0016	265	280	0	102.14	38
1856.8744	2	1856.8761	-0.0016	374	390	0	121.23	37
1884.9388	2	1884.9404	-0.0016	353	370	0	65.51	37
1887.95	3	1887.9513	-0.0013	106	121	1	56.5	37
1976.8742	2	1976.8752	-0.001	41	60	0	117.83	33
2279.1778	3	2279.1798	-0.0019	65	84	1	36.93	36
2345.004	3	2345.0059	-0.0019	403	422	0	55.38	32
2384.1854	2	2384.1873	-0.0018	85	105	1	96.16	37
2394.1838	2	2394.1856	-0.0017	244	264	0	125.3	38
2501.1031	3	2501.107	-0.0039	402	422	1	114.63	33
2640.2217	3	2640.22	0.0018	281	304	0	72.04	36
3192.6214	3	3192.6238	-0.0024	125	156	0	98.9	35
3320.7196	3	3320.7187	0.0008	124	156	1	120.75	34
893.4678	2	893.4681	-0.0003	389	395	0	40.8	37
957.5133	2	957.5131	0.0002	118	125	0	48.42	36
1113.6758	2	1113.6758	0	331	340	0	73.29	31
1141.5652	2	1141.5663	-0.001	138	148	0	58.79	35
1335.6043	2	1335.6055	-0.0012	2	14	0	42.03	35
1400.7251	2	1400.726	-0.0009	76	89	0	101.75	37
1446.7438	2	1446.7467	-0.0029	376	388	0	101.46	38
1501.8713	2	1501.8729	-0.0016	153	167	0	92.86	30
1574.8418	2	1574.8417	0.0001	375	388	1	110.34	37
1586.6945	2	1586.6956	-0.0011	184	196	0	105.88	33
1826.9299	2	1826.9315	-0.0016	53	68	1	101.14	37
1919.9974	2	1919.988	0.0094	396	412	0	68.6	37
2026.8867	3	2026.8875	-0.0008	300	317	1	100.1	33
2074.0756	2	2074.0769	-0.0012	213	231	0	108.68	37
2698.389	2	2698.3888	0.0003	266	288	0	86.29	35
2894.4912	3	2894.4927	-0.0014	341	365	0	127.63	35
3136.6036	2	3136.6049	-0.0013	90	117	0	36.72	35
893.4678	2	893.4681	-0.0003	389	395	0	40.8	37
934.5118	2	934.5124	-0.0006	129	137	0	43.34	35
957.5133	2	957.5131	0.0002	118	125	0	48.42	36

975.5502	2	975.5502	0	376	384	0	69.86	36
1103.6446	2	1103.6451	-0.0006	375	384	1	50.61	33
1113.6758	2	1113.6758	0	331	340	0	73.29	31
1141.5652	2	1141.5663	-0.001	138	148	0	58.79	35
1400.7251	2	1400.726	-0.0009	76	89	0	101.75	37
1501.8713	2	1501.8729	-0.0016	153	167	0	92.86	30
1586.6945	2	1586.6956	-0.0011	184	196	0	105.88	33
1826.9299	2	1826.9315	-0.0016	53	68	1	101.14	37
1919.9974	2	1919.988	0.0094	396	412	0	68.6	37
2026.8867	3	2026.8875	-0.0008	300	317	1	100.1	33
2074.0756	2	2074.0769	-0.0012	213	231	0	108.68	37
2698.389	2	2698.3888	0.0003	266	288	0	86.29	35
2894.4912	3	2894.4927	-0.0014	341	365	0	127.63	35
3875.7475	3	3875.7472	0.0002	20	52	0	79.67	33
908.4956	2	908.4967	-0.0011	157	164	1	46.2	33
986.5397	2	986.5397	0.0001	327	336	0	44.67	37
1282.4962	2	1282.4967	-0.0005	312	320	0	53.32	24
1379.6899	3	1379.6907	-0.0008	391	401	1	41.64	38
1530.8982	2	1530.8981	0.0001	230	243	0	116.73	28
1583.7439	2	1583.7443	-0.0004	340	352	0	79.54	36
1690.8624	2	1690.8638	-0.0014	216	229	1	56.66	38
1714.9134	2	1714.9142	-0.0008	65	79	0	90.85	36
1733.9168	2	1733.9174	-0.0007	265	280	0	79.69	37
1867.889	2	1867.892	-0.003	374	390	0	117.78	37
1884.9388	2	1884.9404	-0.0016	353	370	0	65.51	37
2279.1778	3	2279.1798	-0.0019	65	84	1	36.93	36
2345.004	3	2345.0059	-0.0019	403	422	0	55.38	32
2384.1854	2	2384.1873	-0.0018	85	105	1	96.16	37
2501.1031	3	2501.107	-0.0039	402	422	1	114.63	33
2694.2665	3	2694.2669	-0.0004	281	304	0	43.44	36
3192.6214	3	3192.6238	-0.0024	125	156	0	98.9	35
3320.7196	3	3320.7187	0.0008	124	156	1	120.75	34
1094.6379	2	1094.6376	0.0003	382	390	0	42.26	31
1128.6384	2	1128.6391	-0.0006	465	476	0	62.31	35
1156.6417	2	1156.6452	-0.0035	316	326	0	98.31	36
1388.6628	2	1388.6646	-0.0018	330	341	0	70.23	36
1434.6616	2	1434.6626	-0.001	113	124	1	79.8	36
1680.9381	2	1680.941	-0.0029	342	357	0	105.45	32
1767.7778	3	1767.7787	-0.001	143	157	0	59.85	33
1809.8719	2	1809.8745	-0.0025	127	142	0	114.61	37
1811.9643	2	1811.9638	0.0005	441	456	0	74.93	36
1922.0396	2	1922.0394	0.0002	93	112	0	130.56	34
2113.0424	2	2113.0426	-0.0002	205	222	0	134.71	37
2148.146	2	2148.1467	-0.0006	283	301	0	106.09	35
3051.5959	3	3051.5951	0.0008	330	357	1	67.19	34
3434.7324	2	3434.7344	-0.002	223	254	0	69.57	35
797.5124	2	797.5123	0.0001	307	313	0	52.18	26
971.5401	2	971.54	0.0001	566	574	0	54.76	36
1037.5214	2	1037.5216	-0.0002	829	836	0	49.18	37
1049.525	2	1049.5254	-0.0004	521	529	0	58.81	37
1115.5835	2	1115.5822	0.0013	862	870	0	44.47	37
1157.6287	2	1157.6292	-0.0005	200	209	0	66.17	37
1158.5866	2	1158.588	-0.0014	506	516	0	80.95	37
1172.4843	2	1172.4842	0.0001	693	701	0	55.48	29
1206.6353	3	1206.6357	-0.0004	438	447	1	52.04	37
1263.528	2	1263.5302	-0.0022	548	558	0	48.92	30
1313.7289	2	1313.7303	-0.0014	199	209	1	36.93	36
1318.7109	3	1318.7105	0.0003	519	529	1	37.85	37

1381.7552	2	1381.7565	-0.0013	288	300	1	74.08	34
1396.73	2	1396.731	-0.001	236	248	0	58.23	36
1410.6414	2	1410.6416	-0.0002	875	887	0	59.04	35
1475.7759	2	1475.7773	-0.0014	183	196	0	72.21	38
1495.7225	2	1495.7228	-0.0003	275	287	0	74.41	37
1690.8029	2	1690.8024	0.0005	678	692	0	71.91	37
1891.0402	2	1891.0415	-0.0012	454	470	0	109.53	33
1984.9494	3	1984.9499	-0.0005	743	759	0	42.36	37
2032.1222	2	2032.1218	0.0005	210	227	0	79.09	32
2250.0899	3	2250.0916	-0.0017	481	500	0	71.72	37
2263.1432	2	2263.1485	-0.0052	707	728	0	115	37
2396.273	3	2395.2669	1.0062	767	788	1	59.51	34
2490.3829	3	2490.3846	-0.0017	449	470	1	31.62	29
2507.3745	3	2507.3748	-0.0003	136	160	0	94.13	31
2663.4742	4	2663.4759	-0.0017	135	160	1	51.42	29
3307.5964	3	3306.5892	1.0072	581	611	0	58.96	36
3816.0484	3	3815.0468	1.0015	385	421	1	34.68	29
1003.4755	2	1003.4757	-0.0002	351	359	0	50.79	36
1064.4188	2	1064.4201	-0.0013	298	306	0	33.07	26
1138.6858	2	1138.6862	-0.0004	253	262	0	73.82	28
1145.5818	2	1145.5829	-0.0011	242	251	0	44.4	38
1230.5696	2	1230.5703	-0.0007	381	390	0	71.7	35
1273.6776	2	1273.6779	-0.0003	242	252	1	44.7	38
1292.6363	2	1292.6361	0.0003	36	46	0	71.09	38
1370.6757	2	1370.6765	-0.0008	380	390	1	52.99	36
1383.6944	2	1383.6931	0.0014	163	174	0	81.19	37
1430.6242	2	1430.6248	-0.0006	47	58	0	88.53	32
1445.6811	2	1445.682	-0.001	325	336	0	82.73	36
1621.844	2	1621.8439	0.0001	263	276	0	89.78	37
1695.826	2	1695.8257	0.0004	337	350	0	80.01	37
1955.9959	2	1955.9953	0.0006	104	121	0	127.56	37
2084.0898	3	2084.0902	-0.0005	104	122	1	75.31	36
2750.3174	3	2750.319	-0.0017	217	241	0	59.81	37
2827.3415	3	2827.3467	-0.0052	78	103	1	75.16	36
3398.4952	3	3398.5	-0.0048	123	154	0	71.48	31
4573.1092	3	4572.1135	0.9957	175	213	0	37.76	33
1214.6444	2	1214.6441	0.0003	164	175	0	74.12	37
1227.6195	2	1227.6207	-0.0013	29	39	0	56.17	36
1229.5223	2	1229.5234	-0.0011	81	91	0	72.83	30
1277.6219	2	1277.6227	-0.0008	243	252	0	50.39	38
1312.6121	2	1312.6122	0	308	317	0	42.09	36
1357.6077	2	1357.6084	-0.0007	546	556	0	64.67	34
1385.7248	2	1385.7224	0.0024	604	615	0	46.93	37
1425.7566	2	1425.7576	-0.001	335	348	0	58.84	36
1472.6778	2	1472.6783	-0.0006	40	52	0	80.67	35
1539.7495	2	1539.7504	-0.0009	306	317	1	58.41	37
1658.8871	2	1658.8879	-0.0008	176	191	0	88.34	37
1662.8008	2	1662.8036	-0.0027	60	74	0	82.47	38
1674.7213	2	1674.7234	-0.0021	227	242	0	93.85	32
1679.826	2	1679.8267	-0.0007	145	159	0	48.97	38
1786.9819	2	1786.9828	-0.001	176	192	1	54.57	34
1965.9973	2	1965.9982	-0.0009	352	367	1	83.92	37
2559.2462	2	2559.2452	0.001	368	393	0	137.6	37
2657.2592	2	2657.2609	-0.0017	431	453	0	102.91	36
3025.4881	3	3024.4815	1.0065	279	305	0	43.83	36
3034.6993	3	3034.6988	0.0004	394	422	0	47.8	26
1064.4188	2	1064.4201	-0.0013	299	307	0	33.07	26
1138.6858	2	1138.6862	-0.0004	254	263	0	73.82	28

1145.5818	2	1145.5829	-0.0011	243	252	0	44.4	38
1230.5696	2	1230.5703	-0.0007	382	391	0	71.7	35
1273.6776	2	1273.6779	-0.0003	243	253	1	44.7	38
1370.6757	2	1370.6765	-0.0008	381	391	1	52.99	36
1379.6334	2	1379.6317	0.0017	36	47	0	84.46	36
1383.6944	2	1383.6931	0.0014	164	175	0	81.19	37
1621.844	2	1621.8439	0.0001	264	277	0	89.78	37
1678.789	2	1678.7906	-0.0016	64	78	0	72.6	37
1695.826	2	1695.8257	0.0004	338	351	0	80.01	37
1955.9959	2	1955.9953	0.0006	105	122	0	127.56	37
2084.0898	3	2084.0902	-0.0005	105	123	1	75.31	36
2813.3283	3	2813.331	-0.0027	79	104	1	97.05	36
3398.4952	3	3398.5	-0.0048	124	155	0	71.48	31
4573.1092	3	4572.1135	0.9957	176	214	0	37.76	33
1214.6444	2	1214.6441	0.0003	164	175	0	74.12	37
1227.6195	2	1227.6207	-0.0013	29	39	0	56.17	36
1277.6219	2	1277.6227	-0.0008	243	252	0	50.39	38
1307.6968	2	1307.6972	-0.0005	133	144	0	61.71	36
1357.6077	2	1357.6084	-0.0007	546	556	0	64.67	34
1385.7248	2	1385.7224	0.0024	604	615	0	46.93	37
1406.755	2	1406.7558	-0.0008	352	363	0	87.45	35
1430.7361	2	1430.7365	-0.0004	335	348	0	86.58	37
1472.6778	2	1472.6783	-0.0006	40	52	0	80.67	35
1521.7923	2	1521.7939	-0.0017	306	317	1	53.08	37
1658.8871	2	1658.8879	-0.0008	176	191	0	88.34	37
1674.7213	2	1674.7234	-0.0021	227	242	0	93.85	32
1676.8178	2	1676.8192	-0.0014	60	74	0	83.87	38
1679.826	2	1679.8267	-0.0007	145	159	0	48.97	38
1786.9819	2	1786.9828	-0.001	176	192	1	54.57	34
2111.091	3	2111.0939	-0.0029	81	98	1	45.21	36
2573.259	2	2573.2609	-0.0018	368	393	0	129.61	37
2657.2592	2	2657.2609	-0.0017	431	453	0	102.91	36
847.4912	2	847.4916	-0.0003	445	452	0	58.94	35
950.4812	2	950.4821	-0.0009	974	982	0	44.99	38
971.5288	2	971.5287	0	41	48	0	50.89	35
987.5518	2	987.5535	-0.0018	353	361	0	54.71	38
1151.5939	2	1151.5935	0.0004	1073	1082	0	46.09	37
1234.5977	2	1234.5976	0.0002	717	727	0	44.33	38
1268.7084	2	1268.7088	-0.0004	512	524	0	58.51	34
1275.6419	2	1275.6427	-0.0008	230	240	0	88.11	38
1405.8025	2	1405.8041	-0.0016	832	844	0	58.52	31
1467.7757	2	1467.7755	0.0002	599	611	0	66.16	36
1482.754	2	1482.7541	0	389	400	0	102.59	37
1567.8223	2	1567.8246	-0.0023	273	285	0	60.65	36
1930.0661	3	1930.0676	-0.0015	1025	1042	1	37.31	33
1964.0145	2	1964.0136	0.0009	191	209	0	106.03	36
2099.0444	3	2098.0405	1.0039	384	400	1	63.98	37
2240.2258	2	2240.2264	-0.0005	49	70	0	140.85	32
2500.3358	2	2500.336	-0.0001	453	475	0	82.73	34
4585.2433	4	4585.2435	-0.0002	246	285	1	37.42	33
783.4973	2	783.4967	0.0007	286	292	0	36.71	29
1037.5214	2	1037.5216	-0.0002	808	815	0	49.18	37
1115.5835	2	1115.5822	0.0013	841	849	0	44.47	37
1157.6287	2	1157.6292	-0.0005	180	189	0	66.17	37
1158.5866	2	1158.588	-0.0014	485	495	0	80.95	37
1172.4843	2	1172.4842	0.0001	672	680	0	55.48	29
1206.6353	3	1206.6357	-0.0004	417	426	1	52.04	37
1313.7289	2	1313.7303	-0.0014	179	189	1	36.93	36

1381.7552	2	1381.7565	-0.0013	267	279	1	74.08	34
1396.73	2	1396.731	-0.001	216	228	0	58.23	36
1425.6517	2	1425.6525	-0.0007	854	866	0	65.54	35
1475.7759	2	1475.7773	-0.0014	163	176	0	72.21	38
1495.7225	2	1495.7228	-0.0003	254	266	0	74.41	37
1690.8029	2	1690.8024	0.0005	657	671	0	71.91	37
1984.9494	3	1984.9499	-0.0005	722	738	0	42.36	37
2032.1222	2	2032.1218	0.0005	190	207	0	79.09	32
2263.1432	2	2263.1485	-0.0052	686	707	0	115	37
2396.273	3	2395.2669	1.0062	746	767	1	59.51	34
2507.3745	3	2507.3748	-0.0003	116	140	0	94.13	31
2663.4742	4	2663.4759	-0.0017	115	140	1	51.42	29
3816.0484	3	3815.0468	1.0015	364	400	1	34.68	29
1020.5281	2	1020.5274	0.0008	686	694	0	50.98	38
1155.5338	2	1155.5342	-0.0004	224	233	0	48.58	35
1162.5974	2	1162.5982	-0.0008	48	56	0	50.54	38
1166.5912	2	1166.5931	-0.0019	57	66	0	45.13	37
1266.6014	2	1266.6026	-0.0012	660	669	0	57.65	37
1335.6664	2	1335.667	-0.0006	157	168	0	63.86	38
1402.8589	2	1402.8588	0.0001	562	574	0	90.82	20
1479.815	2	1479.8158	-0.0008	828	842	1	78.81	34
1564.9018	2	1564.9011	0.0007	671	685	0	74.71	29
1998.0502	2	1998.0535	-0.0032	843	861	0	128.11	36
2053.0388	2	2053.0401	-0.0013	115	133	0	90.17	37
2111.0448	2	2111.0436	0.0012	368	387	0	102.6	37
2538.3011	3	2538.2999	0.0012	115	137	1	64.91	36
2660.2807	3	2660.2826	-0.0019	170	192	0	41.93	37
2662.4743	3	2661.4629	1.0114	562	586	1	45.21	29
913.5594	2	913.5597	-0.0002	419	427	0	41.98	33
952.5223	2	952.5229	-0.0006	173	180	1	34.71	34
1024.6019	2	1024.603	-0.0011	244	254	0	84.81	31
1119.5913	2	1119.5924	-0.0011	21	30	0	44.02	37
1297.7393	2	1297.7394	-0.0001	135	146	0	83.89	33
1450.6655	2	1450.6663	-0.0008	85	96	0	59.47	36
1585.8237	2	1585.8239	-0.0002	167	179	1	61.94	37
1633.892	3	1633.894	-0.0021	6	20	0	57.7	35
2295.1254	2	2295.1279	-0.0025	392	411	1	95.38	37
2521.3156	2	2521.3172	-0.0016	255	278	1	79.79	35
2972.4186	3	2972.4107	0.0078	101	129	0	72.87	37
1196.6871	2	1196.6877	-0.0006	164	175	0	84.93	33
1227.6195	2	1227.6207	-0.0013	29	39	0	56.17	36
1277.6219	2	1277.6227	-0.0008	243	252	0	50.39	38
1357.6077	2	1357.6084	-0.0007	546	556	0	64.67	34
1472.6778	2	1472.6783	-0.0006	40	52	0	80.67	35
1658.8871	2	1658.8879	-0.0008	176	191	0	88.34	37
1662.8008	2	1662.8036	-0.0027	60	74	0	82.47	38
1674.7213	2	1674.7234	-0.0021	227	242	0	93.85	32
1679.826	2	1679.8267	-0.0007	145	159	0	48.97	38
1786.9819	2	1786.9828	-0.001	176	192	1	54.57	34
1965.9973	2	1965.9982	-0.0009	352	367	1	83.92	37
2573.259	2	2573.2609	-0.0018	368	393	0	129.61	37
3149.497	3	3148.4724	1.0246	575	601	1	41.23	36
792.4385	2	792.4382	0.0004	219	225	0	46.55	35
815.4865	2	815.4865	0	121	127	0	41.33	37
943.5812	2	943.5814	-0.0002	121	128	1	33.84	33
1173.606	2	1173.607	-0.001	191	200	0	73.98	38
1221.6222	2	1221.6241	-0.0019	149	158	0	50.3	38
1478.7726	2	1478.7729	-0.0003	147	158	1	81.19	37

1514.7087	2	1514.7101	-0.0013	354	368	0	119.1	36
1681.8262	2	1681.8311	-0.0049	88	101	0	71.78	37
1828.9904	2	1828.9903	0.0002	312	328	0	133.37	35
1845.8478	2	1845.8493	-0.0015	159	174	0	86.38	35
2156.0706	2	2156.0724	-0.0018	129	146	1	51.58	37
1227.6195	2	1227.6207	-0.0013	29	39	0	56.17	36
1230.6398	2	1230.6391	0.0008	164	175	0	70.55	38
1277.6219	2	1277.6227	-0.0008	243	252	0	50.39	38
1312.6121	2	1312.6122	0	308	317	0	42.09	36
1357.6077	2	1357.6084	-0.0007	546	556	0	64.67	34
1411.7409	2	1411.742	-0.0011	335	348	0	74.23	36
1472.6778	2	1472.6783	-0.0006	40	52	0	80.67	35
1539.7495	2	1539.7504	-0.0009	306	317	1	58.41	37
1580.812	2	1580.812	0	117	130	0	50.7	37
1658.8871	2	1658.8879	-0.0008	176	191	0	88.34	37
1664.7809	2	1664.7828	-0.0019	60	74	0	63.77	37
1674.7213	2	1674.7234	-0.0021	227	242	0	93.85	32
1679.826	2	1679.8267	-0.0007	145	159	0	48.97	38
1786.9819	2	1786.9828	-0.001	176	192	1	54.57	34
1965.9973	2	1965.9982	-0.0009	352	367	1	83.92	37
2573.259	2	2573.2609	-0.0018	368	393	0	129.61	37
914.5798	2	914.58	-0.0002	1793	1801	0	52.38	29
954.5865	2	954.5862	0.0003	858	866	0	40.04	30
1034.5758	2	1034.576	-0.0003	11	19	0	50.28	35
1112.5498	2	1112.5502	-0.0004	1291	1299	0	54.78	35
1379.7553	2	1378.7497	1.0057	4357	4368	0	60.46	34
1419.7721	2	1419.7722	-0.0001	2674	2686	0	93.7	36
1477.6713	2	1477.6732	-0.0019	1896	1909	0	53.16	35
1501.7164	2	1501.7195	-0.0031	4262	4275	0	58.56	36
1540.8403	2	1540.8395	0.0008	4372	4384	0	45.91	35
1618.893	2	1618.893	0	3071	3085	0	103.39	34
1885.0912	2	1885.0924	-0.0012	4280	4296	0	51.63	27
1944.9821	2	1944.9833	-0.0011	1587	1602	0	43.18	37
2066.0842	3	2066.0836	0.0006	135	152	0	47.15	36
2092.0872	2	2092.0874	-0.0002	4244	4261	0	103.64	36
2198.0586	2	2198.0605	-0.0019	995	1014	0	109.13	37
2526.1382	3	2526.1412	-0.003	2688	2708	0	40.98	34
1204.8159	2	1204.8159	0.0001	1902	1912	0	51.56	20
1233.6025	2	1233.6023	0.0001	1954	1964	0	46.71	38
1361.7389	2	1361.7377	0.0012	1194	1205	0	67.47	36
1373.7589	2	1373.7588	0.0001	1301	1313	0	69.13	36
1760.9493	2	1760.9495	-0.0001	1939	1953	0	94.71	35
2048.0982	2	2048.0983	0	957	974	0	113.82	35
2193.225	2	2193.2256	-0.0006	440	459	0	112.67	30
2239.1692	3	2239.1696	-0.0004	2495	2515	0	90.47	36
2735.5257	3	2735.5255	0.0002	436	459	1	67.07	27
3140.7538	3	3139.7394	1.0144	1537	1565	0	51.47	26
1157.6441	2	1157.6445	-0.0004	175	186	0	39.64	37
1161.5795	2	1161.5778	0.0017	340	350	0	75.18	38
1272.6374	2	1272.635	0.0024	329	339	0	69.83	37
1275.6822	2	1275.6823	0	192	203	0	64.91	37
1381.6488	2	1381.6482	0.0006	351	362	0	62.71	37
1391.7518	2	1391.7516	0.0002	104	115	0	84.77	36
1706.9193	3	1706.9203	-0.001	204	219	1	51.13	36
1799.9458	2	1799.9491	-0.0033	153	167	0	71.86	36
1936.9559	2	1935.9612	0.9947	262	278	0	93.5	38
2223.205	3	2222.2019	1.0031	55	74	1	63.95	32
3243.5476	3	3243.5506	-0.0031	120	149	0	75.78	36

1151.7063	2	1151.7067	-0.0004	157	167	0	52.9	24
1227.5615	2	1227.5628	-0.0012	172	181	0	44.64	34
1696.7546	2	1696.754	0.0006	73	87	0	100.03	34
1727.7411	2	1727.743	-0.0019	222	236	0	50.83	31
1779.8017	2	1779.8025	-0.0008	378	392	0	57.87	35
2149.124	2	2149.1242	-0.0001	278	296	0	143.82	36
2192.3088	3	2192.3085	0.0002	148	167	1	51.79	21
2346.179	2	2346.1815	-0.0025	410	432	0	157.26	37
843.4993	2	843.5	-0.0007	75	82	0	58.11	37
1034.482	2	1034.4821	-0.0001	88	95	0	48.52	36
1062.5906	2	1062.5921	-0.0014	96	105	0	45.79	36
1165.5976	2	1165.5979	-0.0003	40	50	0	80.29	37
1235.7349	2	1235.735	-0.0001	970	980	0	41.85	28
1708.7894	2	1708.7913	-0.0018	902	915	0	76.87	36
1727.8647	2	1727.8665	-0.0017	462	477	0	61.63	37
2379.1236	2	2379.1246	-0.0009	874	893	0	82.32	37
2503.2466	2	2503.2476	-0.0009	270	292	0	120.38	37
2783.3739	4	2783.3739	0	437	461	0	39.82	37
2883.4308	3	2883.4324	-0.0016	1004	1029	0	41.68	37
3196.6117	3	3196.6099	0.0018	236	265	0	41.31	36
3316.6552	3	3315.6445	1.0106	478	508	0	67.55	36
865.5136	2	865.5134	0.0003	349	355	0	44.82	30
1157.6441	2	1157.6445	-0.0004	206	217	0	39.64	37
1166.5312	2	1166.5316	-0.0004	279	288	1	60.96	35
1194.5506	2	1194.5517	-0.0011	371	381	0	91.99	35
1294.6339	2	1294.634	-0.0001	223	234	0	47.65	37
1644.8836	2	1643.8705	1.0131	235	250	0	78.79	36
1966.9303	2	1966.9306	-0.0003	293	309	0	151.24	37
2257.0724	3	2256.0658	1.0066	395	414	0	50.6	37
3831.7942	3	3830.8044	0.9898	68	101	0	58.16	34
1247.6756	2	1247.6761	-0.0006	393	403	0	69.07	36
1248.5577	2	1248.5582	-0.0005	547	558	0	50.58	34
1326.6296	2	1326.6303	-0.0007	581	592	0	80.29	36
1360.737	2	1360.7351	0.0019	160	172	0	54.27	37
1416.727	2	1416.7283	-0.0013	377	389	0	94.8	38
1476.757	2	1476.7573	-0.0002	107	120	0	48.92	38
1812.9462	2	1812.9469	-0.0007	601	617	0	125.35	36
1938.9996	2	1937.9993	1.0003	408	427	0	55.49	36
2070.0847	3	2070.0844	0.0003	599	617	1	56.38	36
2824.6208	3	2824.6202	0.0007	428	454	1	65.07	23
3077.4619	3	3077.4618	0.0001	264	290	1	73.48	36
815.4865	2	815.4865	0	121	127	0	41.33	37
943.5812	2	943.5814	-0.0002	121	128	1	33.84	33
1173.606	2	1173.607	-0.001	191	200	0	73.98	38
1221.6222	2	1221.6241	-0.0019	149	158	0	50.3	38
1478.7726	2	1478.7729	-0.0003	147	158	1	81.19	37
1514.7087	2	1514.7101	-0.0013	354	368	0	119.1	36
1764.9042	2	1764.9046	-0.0005	88	102	0	107.5	37
1799.9308	2	1799.9305	0.0003	46	63	0	74.9	37
1821.957	2	1821.9584	-0.0014	335	351	0	93.59	36
2156.0706	2	2156.0724	-0.0018	129	146	1	51.58	37
2735.3084	3	2735.3079	0.0005	64	87	0	38.22	37
1018.5057	2	1018.5043	0.0014	805	815	0	91.13	38
1186.6296	2	1186.6306	-0.001	575	585	0	66.74	38
1276.7026	2	1276.7027	-0.0001	779	789	0	43	35
1282.6161	2	1282.6162	-0.0001	543	553	0	58.79	36
1423.7563	2	1423.7567	-0.0004	478	489	0	79.25	35
1441.7997	3	1441.8001	-0.0005	573	585	1	46.89	36

1442.7764	2	1442.7769	-0.0005	261	272	0	53.81	36
1461.7617	2	1461.7616	0.0001	683	694	0	79.74	38
1643.9353	2	1643.9354	-0.0001	310	323	0	109.19	31
2484.2107	3	2484.2107	0	385	404	1	94.08	37
960.4128	2	960.4124	0.0004	544	552	0	54.7	29
1071.5387	2	1071.5383	0.0005	133	142	0	41.74	35
1186.6952	2	1186.6962	-0.0009	155	165	0	43	33
1197.5436	2	1197.5448	-0.0012	482	493	0	65.03	35
1465.8178	2	1465.8187	-0.001	176	188	1	51.35	33
1499.6784	2	1499.6787	-0.0003	375	387	0	71.76	35
1540.9594	3	1540.9592	0.0002	155	168	1	41.07	20
1814.9034	2	1814.905	-0.0017	319	335	0	106.2	37
2243.1805	3	2243.1811	-0.0006	390	410	1	61.71	36
2409.127	2	2409.1304	-0.0034	200	221	0	77.01	36
2583.3389	3	2583.3392	-0.0003	92	117	1	74.77	35
2904.4744	3	2904.477	-0.0026	411	435	0	41.49	36
832.4593	2	832.4555	0.0038	8	15	0	45.83	37
1139.6363	2	1139.6373	-0.001	328	337	0	55.77	33
1147.6605	3	1147.6601	0.0004	303	313	1	42.39	33
1433.748	2	1433.7514	-0.0034	205	219	0	41.49	38
1497.8397	2	1497.8403	-0.0006	239	252	0	115.54	34
1675.9656	3	1675.9661	-0.0005	70	84	1	56.38	29
1760.7782	2	1760.7794	-0.0012	314	327	0	75.41	33
2032.0662	2	2032.0663	-0.0001	172	190	0	109.33	36
2171.0008	2	2171.0019	-0.001	276	295	0	108.57	36
2629.3891	3	2629.3898	-0.0007	167	190	1	73.5	34
1129.5616	2	1129.5615	0.0001	613	622	0	61.54	36
1189.6703	2	1189.6707	-0.0003	398	408	0	52.16	35
1234.5672	2	1234.5677	-0.0005	552	563	0	74.63	36
1416.7205	2	1416.7209	-0.0003	112	125	0	56.4	38
1416.727	2	1416.7283	-0.0013	382	394	0	94.8	38
1820.9007	2	1820.9017	-0.001	472	488	0	105.45	38
1868.9904	3	1867.9891	1.0014	606	622	1	52.48	35
1938.9996	2	1937.9993	1.0003	413	432	0	55.49	36
2455.394	2	2455.3938	0.0003	437	459	0	87.29	27
954.5649	2	954.5651	-0.0002	461	468	0	39.03	31
1090.5913	2	1090.591	0.0003	496	504	0	41.4	37
1316.7157	2	1316.7162	-0.0005	219	230	0	61.05	37
1608.8707	2	1608.8722	-0.0016	310	324	0	61.25	35
1650.8108	2	1650.8114	-0.0006	169	183	0	83.1	38
1701.7304	2	1701.7304	-0.0001	258	273	0	101.55	31
1970.0456	3	1970.0473	-0.0017	478	495	0	45.52	35
2196.0998	2	2196.1022	-0.0024	197	218	0	90.57	37
2342.2073	3	2341.1968	1.0105	280	299	1	80.46	36
2657.3388	3	2656.3418	0.997	365	386	0	70.17	37
3156.613	3	3156.6125	0.0005	116	145	0	70.39	35
743.3751	2	743.3755	-0.0004	392	397	0	39.7	33
1451.682	2	1451.6834	-0.0014	244	255	0	66.25	36
1501.67	2	1501.6718	-0.0019	593	606	0	137.51	34
1798.9235	2	1798.9254	-0.0018	771	786	0	70.69	37
1806.9194	3	1806.9224	-0.003	464	479	1	53.77	38
2256.1956	2	2256.1961	-0.0005	33	55	0	109.74	35
2399.1577	3	2398.1553	1.0024	349	368	1	71.14	37
2931.4242	3	2931.425	-0.0008	94	120	0	103.31	37
3221.6198	4	3221.6188	0.001	321	348	0	36.57	36
1041.4881	2	1041.488	0.0002	364	372	0	49.94	34
1219.6373	2	1219.6383	-0.0011	388	397	0	75.43	38
1255.6446	2	1255.6448	-0.0002	589	598	0	70.09	35

1324.6736	3	1324.6735	0.0001	412	422	1	40.27	37
1469.7857	2	1469.7878	-0.0021	143	156	0	59.99	36
1648.7857	2	1648.7879	-0.0022	258	272	0	113.25	37
1664.8396	2	1664.841	-0.0014	677	692	0	113.75	38
2415.212	2	2415.2142	-0.0022	563	585	0	118.17	37
1343.7413	2	1343.7409	0.0004	84	96	0	90.75	37
1640.821	2	1640.8192	0.0018	181	195	0	41.84	37
1729.8263	2	1729.8271	-0.0008	517	532	0	103.33	37
1942.9255	3	1942.9261	-0.0006	376	391	0	39.14	37
2149.0952	2	2149.0977	-0.0024	427	446	0	162.24	37
2679.3406	3	2679.3405	0.0001	486	510	0	65.68	37
971.5664	2	971.5651	0.0013	253	261	0	41.78	36
1082.5857	2	1082.5873	-0.0015	26	35	1	36.86	34
1236.6453	2	1236.6462	-0.001	241	252	0	61.05	37
1286.7015	2	1286.7016	-0.0001	400	411	0	45.27	37
1435.8141	3	1435.8147	-0.0005	337	349	1	35.09	31
1750.8557	2	1750.856	-0.0003	311	325	0	126.05	38
2144.102	2	2144.1001	0.002	182	201	0	119.03	37
2157.1388	2	2157.1425	-0.0037	159	178	0	82.04	36
2204.1292	2	2203.126	1.0032	276	296	0	115.42	37
1203.6149	2	1203.6136	0.0013	82	92	0	45.84	38
1289.6094	2	1289.6099	-0.0005	425	436	0	87.66	36
1478.7382	2	1478.7405	-0.0024	242	254	0	60.6	38
1649.7844	2	1649.7857	-0.0012	125	141	0	149.81	37
1752.92	2	1752.9192	0.0008	103	119	0	118.28	36
2138.1503	3	2138.1518	-0.0014	59	79	1	50.41	34
2249.2876	2	2249.2882	-0.0006	289	310	0	83.17	27
2518.4723	3	2518.4734	-0.0011	289	312	1	34.76	22
2848.6289	3	2848.6273	0.0016	284	310	1	38.74	23
975.4409	2	975.441	-0.0001	21	30	0	63.16	33
1143.5534	2	1143.5529	0.0005	42	52	0	67.35	35
1175.5282	2	1175.5281	0.0001	199	208	0	48.59	34
1197.6975	2	1197.6982	-0.0007	31	41	1	49.12	33
1773.8873	2	1773.8897	-0.0024	241	256	0	98.46	38
1854.922	2	1854.9251	-0.0031	218	233	0	98.16	37
1953.0573	2	1953.0571	0.0002	98	115	0	55.13	34
3150.6316	3	3150.635	-0.0034	150	179	0	96.21	34
1092.4647	2	1092.4658	-0.0011	72	80	0	33.59	31
1192.6548	2	1192.6564	-0.0016	138	147	0	65.85	35
1216.5968	2	1216.5989	-0.0021	321	330	0	53.94	36
1279.623	2	1279.6231	-0.0001	207	217	0	78.08	37
1422.6565	2	1422.6568	-0.0002	183	193	0	60.2	36
1482.8139	2	1482.8155	-0.0015	102	116	0	95.8	34
1601.8628	2	1601.8633	-0.0005	306	320	0	93.83	36
2032.0665	3	2032.0688	-0.0023	346	365	1	37.83	36
942.5014	2	942.5022	-0.0008	191	198	1	42.78	35
1282.7489	2	1282.7496	-0.0008	115	127	0	41.33	32
1302.7435	2	1302.7435	0	136	146	0	53.81	33
1309.6128	2	1309.6125	0.0003	270	280	0	84.25	37
1379.6384	2	1379.6391	-0.0007	290	302	0	40.09	37
1493.7463	2	1493.7474	-0.0011	14	26	0	86.22	38
1512.8298	3	1512.83	-0.0002	204	216	0	69.72	35
1534.9455	2	1534.9447	0.0008	162	175	1	58.73	20
1752.8498	2	1752.8505	-0.0007	147	161	0	72.42	37
1071.5387	2	1071.5383	0.0005	145	154	0	41.74	35
1183.5279	2	1183.5292	-0.0013	494	505	0	69.89	34
1186.6952	2	1186.6962	-0.0009	167	177	0	43	33
1273.6662	2	1273.6666	-0.0004	483	493	0	52.67	38

1483.6824	2	1483.6838	-0.0014	387	399	0	63.3	36
1536.8366	3	1536.8372	-0.0006	457	470	1	37.88	35
1540.9594	3	1540.9592	0.0002	167	180	1	41.07	20
1795.7385	2	1795.7398	-0.0013	569	588	0	107.93	28
1802.8498	2	1802.8509	-0.0011	331	347	0	61.58	36
2193.1539	3	2193.1542	-0.0003	402	422	1	58.41	35
2409.127	2	2409.1304	-0.0034	212	233	0	77.01	36
840.5177	2	840.5181	-0.0004	269	276	0	42.89	29
1323.7761	2	1323.7762	-0.0001	139	151	0	61.75	28
1372.6353	2	1372.6358	-0.0004	257	268	0	45.18	35
1598.8079	2	1598.8086	-0.0007	161	174	0	88.51	37
1661.8281	2	1661.8294	-0.0013	239	253	0	109.57	38
2442.1634	3	2442.1633	0.0001	46	67	0	58.49	37
3300.6868	3	3300.6878	-0.001	7	39	0	82.22	35
919.4797	2	919.4797	0.0001	43	52	0	55.85	38
940.5014	2	940.5018	-0.0004	63	70	0	38.97	34
1047.5744	2	1047.5746	-0.0002	43	53	1	39.99	37
1438.6786	2	1438.6801	-0.0015	27	42	0	99.57	37
2155.0164	2	2155.0181	-0.0017	71	89	1	119.31	37
1160.6025	2	1160.6037	-0.0012	259	268	0	59.07	38
1317.753	2	1317.7544	-0.0014	793	804	0	49.71	33
1446.8071	2	1446.8082	-0.0011	330	343	0	113	34
1569.8103	2	1569.8111	-0.0008	1288	1302	0	108.97	36
1889.9854	2	1889.9847	0.0007	440	458	0	149.34	37
2015.0402	3	2015.0411	-0.0009	845	860	0	41.97	37
1157.6441	2	1157.6445	-0.0004	197	208	0	39.64	37
1324.6264	2	1324.6268	-0.0004	374	385	0	52.41	36
1478.8569	3	1478.8569	0	311	323	1	49.74	30
1921.9448	2	1921.9455	-0.0007	285	301	0	122.58	38
2086.995	2	2086.9993	-0.0043	252	270	0	108.01	37
1475.7663	2	1475.7654	0.0009	580	592	0	59.12	38
1524.8987	2	1524.8988	0	1069	1083	0	109.56	29
1940.0499	2	1940.052	-0.0021	810	827	0	123.35	34
2466.2402	3	2465.2285	1.0117	1146	1169	1	82.57	37
1054.5272	2	1054.527	0.0003	1508	1515	0	45.92	35
1101.5934	2	1101.5931	0.0003	629	638	0	49.3	38
1279.7864	2	1279.7864	0.0001	802	814	0	86.63	24
1316.7119	2	1316.7122	-0.0003	539	550	0	48.28	37
1704.8517	2	1704.8505	0.0012	1271	1284	0	93.58	38
2130.0434	2	2130.0441	-0.0006	39	60	0	103.45	37
2201.1761	3	2201.1766	-0.0005	363	382	0	42.15	35
2365.0511	3	2365.0517	-0.0006	1643	1665	1	39	34
2411.1934	2	2411.193	0.0004	1008	1029	0	58.2	37
2510.3534	3	2509.3403	1.0131	1097	1117	1	34.31	33
766.3868	2	766.3861	0.0007	326	332	0	51.03	36
916.574	2	916.5746	-0.0006	367	374	0	31.23	26
1206.6245	2	1206.6245	0.0001	354	366	0	50.24	38
1210.5982	2	1210.5983	-0.0001	228	237	0	59.71	37
1359.7054	2	1359.7068	-0.0013	106	116	0	62.16	38
1407.7143	3	1407.7147	-0.0004	134	145	1	40.88	38
2031.0124	2	2031.0136	-0.0011	76	95	0	87.54	37
2105.188	3	2105.1885	-0.0004	354	374	1	50.14	29
1009.4822	2	1009.4829	-0.0007	475	483	0	38.26	36
1315.6615	2	1315.6619	-0.0004	181	194	0	64.58	37
1559.8018	2	1559.8042	-0.0024	124	138	0	97.34	38
2115.0066	2	2115.0062	0.0005	41	60	0	101.68	37
2375.1064	2	2375.107	-0.0006	139	160	0	128.12	36
3395.6446	3	3395.6442	0.0003	422	454	1	51.48	36

1934.0255	2	1934.0262	-0.0006	180	197	0	109.89	36
2090.1268	3	2090.1273	-0.0004	180	198	1	77.63	34
2430.233	2	2430.2319	0.0012	249	271	0	104.19	37
2483.217	2	2483.2166	0.0004	91	113	0	129.6	37
1212.671	2	1212.6714	-0.0004	296	306	0	87.6	35
1587.7839	2	1587.7852	-0.0013	228	243	0	108.6	37
1632.7942	2	1631.7865	1.0077	249	262	0	62.57	37
1666.9003	2	1666.9002	0.0001	325	340	0	107.63	34
1767.7778	3	1767.7787	-0.001	131	145	0	59.85	33
1154.6401	2	1154.6408	-0.0006	192	203	0	55.73	33
1344.6373	2	1344.6384	-0.0011	571	581	0	37.89	36
1493.714	2	1493.7151	-0.0011	68	80	0	75.8	37
1535.7423	3	1535.744	-0.0018	54	67	0	44.55	38
1658.8871	2	1658.8879	-0.0008	204	219	0	88.34	37
1786.9819	2	1786.9828	-0.001	204	220	1	54.57	34
2051.9874	2	2052.0052	-0.0177	313	330	0	104.14	37
910.4913	2	910.4913	0.0001	125	131	0	47.28	34
1235.6125	2	1235.6146	-0.002	16	26	1	39.04	38
1292.6714	2	1292.6725	-0.0011	92	102	0	64.41	38
1368.6243	2	1368.6245	-0.0002	225	235	0	39.7	35
1576.843	2	1576.8461	-0.003	110	124	0	86.09	37
1613.9054	2	1613.9062	-0.0008	433	446	0	95.4	33
1668.8224	2	1668.822	0.0004	181	196	0	83.56	37
2012.1783	3	2012.1782	0	294	311	0	50.82	23
2545.3904	4	2544.3846	1.0058	197	219	1	39.74	31
4142.1533	4	4142.1549	-0.0016	158	196	1	35.4	31
764.4198	2	764.4181	0.0017	281	287	0	38.56	35
872.5079	2	872.508	-0.0001	292	300	0	45.68	38
978.4666	2	978.4672	-0.0006	365	373	0	52.8	36
1205.6027	2	1205.604	-0.0013	61	70	0	68.14	38
1452.7462	2	1452.7474	-0.0011	238	252	0	84.08	37
1469.7272	2	1469.7258	0.0014	48	60	0	116.03	37
1808.8229	2	1808.8251	-0.0021	75	91	0	109.56	36
2382.2627	3	2382.2656	-0.0029	92	113	0	39.84	35
960.5284	2	960.528	0.0004	94	102	0	43.9	37
1059.5967	2	1059.5964	0.0003	77	85	0	43.41	36
1217.6153	2	1217.6153	0	147	157	0	46.6	38
1239.6244	2	1239.6248	-0.0004	206	215	0	71.37	37
1402.7089	2	1402.7126	-0.0037	324	336	0	76.68	37
1716.8861	2	1716.8869	-0.0007	113	126	0	108.23	37
1927.9861	2	1927.9859	0.0002	292	310	0	62.28	37
3616.783	3	3615.7879	0.9951	44	76	0	70.68	36
1235.6125	2	1235.6146	-0.002	16	26	1	39.04	38
1368.6243	2	1368.6245	-0.0002	220	230	0	39.7	35
1548.8515	2	1548.8512	0.0003	105	119	0	112.12	34
1613.9054	2	1613.9062	-0.0008	428	441	0	95.4	33
1668.8224	2	1668.822	0.0004	176	191	0	83.56	37
2006.2034	3	2006.204	-0.0007	289	306	0	55.59	22
2603.3663	4	2603.3676	-0.0013	192	214	1	42.71	35
754.4334	2	754.4337	-0.0003	405	411	0	45.22	31
1025.5866	2	1025.5869	-0.0003	424	433	0	40.07	32
1249.6298	2	1249.6302	-0.0004	277	287	0	73.26	38
1329.7502	2	1329.7504	-0.0002	448	459	0	69.66	35
1840.9213	3	1840.922	-0.0007	288	302	1	44.4	37
2307.153	2	2307.1495	0.0035	672	693	0	143.47	38
1101.5828	2	1101.5818	0.001	356	364	0	50.83	38
1161.5231	2	1161.5237	-0.0006	204	212	0	45.07	34
1403.652	2	1403.6528	-0.0008	441	453	0	86.68	35

1411.7404	2	1411.7419	-0.0016	467	480	0	90.02	36
2037.9384	2	2037.9426	-0.0041	365	383	0	109.85	36
2223.1746	3	2222.1736	1.0011	96	115	0	63.68	35
975.4409	2	975.441	-0.0001	21	30	0	63.16	33
1131.5231	2	1131.5197	0.0035	199	208	0	37.44	34
1143.5534	2	1143.5529	0.0005	42	52	0	67.35	35
1197.6975	2	1197.6982	-0.0007	31	41	1	49.12	33
1789.8806	2	1789.8846	-0.004	241	256	0	68.41	38
3150.6316	3	3150.635	-0.0034	150	179	0	96.21	34
747.4274	2	747.4279	-0.0005	363	368	0	43.7	36
881.4084	2	881.4065	0.0019	226	232	0	49.59	34
1356.6167	2	1356.6166	0.0002	380	391	0	52.1	35
1406.6063	2	1406.6063	0.0001	278	290	0	82.32	32
1736.8408	2	1736.8403	0.0005	291	305	0	122.02	37
1034.5476	2	1034.5509	-0.0033	413	423	0	54.71	37
1204.5464	2	1204.5473	-0.0009	981	991	0	44.61	35
1370.7921	2	1370.7922	-0.0001	483	494	0	55.55	33
1471.8015	2	1471.8035	-0.002	967	980	0	51.9	36
1543.7832	2	1543.7842	-0.001	1120	1133	0	81.36	37
1617.8407	2	1617.8403	0.0004	992	1006	0	73.84	37
1798.815	2	1798.8156	-0.0006	919	936	0	107.6	35
2774.4789	4	2773.4762	1.0027	750	775	0	44.51	33
1108.533	2	1108.5335	-0.0005	456	464	0	40.69	37
1147.588	2	1147.5873	0.0007	468	477	0	43.76	38
1242.6921	2	1242.6932	-0.001	772	783	0	70.9	37
1278.6388	2	1278.639	-0.0003	686	697	0	69.77	38
1542.8303	3	1542.8307	-0.0004	186	198	0	42.67	37
1748.9766	3	1748.9785	-0.0019	755	771	0	36.66	32
2044.0952	2	2044.0953	0	707	724	0	107.52	35
2901.5058	3	2900.5027	1.003	375	401	0	46.03	34
3112.6362	4	3112.6379	-0.0017	159	185	1	34.83	33
1232.6184	2	1232.6183	0.0001	623	633	0	53.6	38
1276.6636	2	1276.6663	-0.0028	318	330	0	69.34	38
1280.6168	2	1280.6183	-0.0015	331	341	0	48.46	37
1363.6727	2	1363.6732	-0.0005	1007	1019	0	81.48	38
1410.7333	2	1410.7354	-0.0022	54	66	0	43.03	36
1554.8615	2	1554.8617	-0.0002	763	777	0	95.9	34
2488.3166	3	2488.3173	-0.0008	385	407	0	76.6	35
1042.5763	2	1042.5771	-0.0008	104	113	0	79.63	36
1181.7023	2	1181.702	0.0004	78	89	0	67.81	30
1198.6549	2	1198.6557	-0.0009	286	296	0	79.88	34
1279.7608	2	1279.7612	-0.0004	161	173	0	60.89	28
1699.8914	2	1699.8927	-0.0013	459	475	0	100.39	37
2568.4052	3	2568.405	0.0002	297	320	0	53.22	31
1192.6548	2	1192.6564	-0.0016	138	147	0	65.85	35
1216.5968	2	1216.5989	-0.0021	321	330	0	53.94	36
1422.6565	2	1422.6568	-0.0002	183	193	0	60.2	36
1468.7981	2	1468.7998	-0.0017	102	116	0	82.68	35
2235.1364	2	2235.1358	0.0007	118	137	0	72.85	37
2260.071	2	2260.0707	0.0004	346	367	0	97.42	37
991.4837	2	991.4831	0.0006	185	192	0	41.32	38
1058.5349	2	1058.5356	-0.0007	338	347	0	40.77	37
1932.9266	2	1932.9285	-0.0019	268	285	0	89.31	37
2201.089	2	2201.0892	-0.0002	314	332	0	84.45	37
3356.5792	3	3356.5772	0.002	193	222	0	64.1	35
1089.5312	2	1089.5414	-0.0102	313	323	0	48.47	37
1542.7586	2	1542.7606	-0.002	356	368	0	61.43	37
1682.9459	2	1682.9454	0.0005	133	147	0	102.62	32

2322.2796	3	2322.2795	0.0002	133	153	1	92.37	31
1365.7359	2	1365.7365	-0.0006	368	380	0	49.1	35
1406.7047	2	1406.7042	0.0006	1145	1156	0	60.22	38
1434.7346	2	1434.7354	-0.0008	1132	1144	0	90.14	38
1475.7721	2	1475.7732	-0.0011	151	163	0	63.15	38
2023.971	3	2023.9707	0.0003	1308	1325	0	45.39	37
2057.0654	2	2057.0656	-0.0001	520	536	0	48.81	36
2661.4351	3	2660.4326	1.0024	414	437	0	79.19	32
974.5547	2	974.5549	-0.0002	227	237	0	44.21	36
1277.6274	2	1277.6286	-0.0011	135	146	0	40.52	38
1392.8082	2	1392.8089	-0.0007	150	163	0	32.75	28
1424.7669	2	1424.7664	0.0005	309	321	0	46.54	35
1456.8327	2	1456.8323	0.0004	238	250	0	95.47	33
1491.7671	2	1491.7681	-0.001	338	351	0	81.19	38
1863.936	2	1863.9367	-0.0007	322	337	0	74.15	38
3149.7319	3	3149.7336	-0.0017	194	223	1	38.62	29
1022.5829	2	1022.5834	-0.0006	26	34	0	50.47	32
2084.0602	2	2084.0572	0.0031	56	75	0	118.65	37
1209.6718	2	1209.6717	0.0001	5	15	0	34.99	33
1738.999	2	1738.9981	0.0008	437	453	0	90.65	30
2276.3078	2	2276.3104	-0.0025	506	526	0	98.2	26
2619.3938	3	2619.3942	-0.0004	161	183	1	60.26	34
975.4409	2	975.441	-0.0001	21	30	0	63.16	33
1175.5282	2	1175.5281	0.0001	199	208	0	48.59	34
1773.8873	2	1773.8897	-0.0024	241	256	0	98.46	38
1967.073	3	1967.0727	0.0003	98	115	0	36.05	33
3150.6331	3	3150.635	-0.0019	150	179	0	79.25	34
997.5545	2	997.5556	-0.0011	140	148	0	57.41	33
1192.6103	3	1192.6102	0.0001	48	58	0	42.53	38
1402.7935	2	1402.7932	0.0003	150	162	0	84.31	34
1558.8939	3	1558.8943	-0.0005	149	162	1	38.97	31
1630.8234	2	1630.8236	-0.0001	171	185	0	109.76	38
1365.7359	2	1365.7365	-0.0006	368	380	0	49.1	35
1434.7346	2	1434.7354	-0.0008	1132	1144	0	90.14	38
1475.7721	2	1475.7732	-0.0011	151	163	0	63.15	38
2023.971	3	2023.9707	0.0003	1308	1325	0	45.39	37
2039.1096	2	2039.1092	0.0005	520	536	0	57.45	34
2661.4351	3	2660.4326	1.0024	414	437	0	79.19	32
1673.8924	2	1673.893	-0.0006	21	33	0	72.36	37
1819.8024	3	1819.8047	-0.0023	64	78	0	44.94	34
2481.334	2	2480.3274	1.0066	88	110	0	101.26	33
1042.5763	2	1042.5771	-0.0008	100	109	0	79.63	36
1181.7023	2	1181.702	0.0004	74	85	0	67.81	30
1228.6913	2	1228.6914	-0.0001	282	292	0	70.48	36
1279.7608	2	1279.7612	-0.0004	157	169	0	60.89	28
1699.8914	2	1699.8927	-0.0013	455	471	0	100.39	37
985.5526	2	985.5556	-0.0031	125	133	0	49.31	36
1016.601	2	1016.6019	-0.0008	442	450	0	56.13	34
1181.6141	2	1181.6153	-0.0011	798	808	0	40.35	36
1334.6392	2	1334.6401	-0.0009	270	282	0	90.42	37
1921.948	2	1921.9489	-0.0009	838	856	0	112.66	38
916.574	2	916.5746	-0.0006	371	378	0	31.23	26
950.4817	2	950.4821	-0.0004	246	254	0	41.02	38
1227.6133	2	1227.6136	-0.0003	330	340	0	52.33	36
1359.7054	2	1359.7068	-0.0013	110	120	0	62.16	38
1401.7365	3	1401.7365	0	138	149	1	47.48	38
2048.969	2	2048.97	-0.0009	80	99	0	83.84	37
2090.1975	3	2089.1936	1.004	358	378	1	51.1	28

1294.833	2	1294.8336	-0.0006	618	629	0	87.01	20
1942.0885	3	1942.0887	-0.0002	225	241	1	55.95	32
2028.127	2	2028.1255	0.0015	839	856	0	41.04	32
2067.1311	3	2066.1313	0.9998	947	965	0	35.73	33
2073.12	2	2073.1218	-0.0018	694	713	0	127.49	34
1002.4882	2	1002.4883	-0.0001	972	980	0	50.49	37
1080.6289	2	1080.6291	-0.0002	1022	1032	0	35.71	32
1109.4983	2	1109.4989	-0.0006	690	699	0	57.22	35
1324.6629	2	1324.6623	0.0006	1041	1052	0	68.24	37
1346.665	2	1346.6678	-0.0027	708	719	0	74.16	37
1394.6913	2	1394.6929	-0.0016	1096	1108	0	44.6	37
1640.7496	2	1640.7503	-0.0006	771	784	1	49.2	35
1792.9923	2	1792.9935	-0.0011	176	191	0	50.1	32
2687.4641	3	2687.4646	-0.0005	466	490	1	50.28	30
1176.6138	2	1176.6139	0	812	821	0	70.82	38
1483.9017	2	1483.9014	0.0003	433	446	0	51.77	25
1622.8322	2	1622.8304	0.0018	570	583	0	62.3	37
1975.9391	2	1975.9374	0.0017	84	100	0	116.98	37
2014.0283	3	2014.0306	-0.0023	540	556	1	38.98	37
758.4651	2	758.465	0	291	297	0	47.11	35
1102.597	2	1102.5983	-0.0012	848	857	0	41.09	38
1550.7856	2	1550.7875	-0.0019	117	132	0	51.43	37
2097.1062	3	2097.1066	-0.0004	731	749	1	77.41	36
2162.1104	2	2162.1106	-0.0002	198	216	0	60.86	36
2579.2994	2	2579.3006	-0.0012	152	173	0	97.88	37
1378.707	2	1378.7092	-0.0022	376	389	0	80.04	38
1545.7815	2	1545.7821	-0.0006	319	332	0	110.86	38
2156.983	2	2156.9862	-0.0032	356	375	0	88.33	35
915.5542	2	915.5542	0	183	190	0	36.75	35
927.5391	2	927.5389	0.0002	239	246	0	38.42	33
961.523	2	961.5233	-0.0003	588	595	0	64.02	38
1054.5773	2	1054.5771	0.0002	228	238	0	35.67	34
1256.6394	2	1256.6401	-0.0007	569	579	0	50.27	36
1335.7651	2	1335.765	0.0001	339	350	0	81.26	30
1528.8138	2	1528.8137	0.0001	596	609	0	45.84	37
2153.0869	3	2153.0865	0.0004	519	540	0	56.5	37
4049.9657	4	4049.9681	-0.0024	468	505	0	67.84	35
949.5229	2	949.5233	-0.0003	406	413	0	38.47	35
1458.7683	2	1458.7752	-0.0069	96	108	0	56.22	37
1960.9658	2	1960.9676	-0.0018	318	333	0	133.65	38
2095.1659	4	2095.1677	-0.0019	341	359	0	58.25	31
1055.5978	2	1055.5975	0.0004	188	197	0	46.81	33
1125.512	2	1125.5124	-0.0004	129	138	0	75.72	35
1739.9569	2	1739.957	-0.0001	320	336	0	105.27	34
2708.2953	3	2708.2963	-0.001	93	118	0	81.47	37
1604.8402	2	1604.841	-0.0008	603	616	0	94.79	37
1770.0242	2	1770.0251	-0.0009	296	312	0	69.41	29
1924.0185	2	1924.0193	-0.0008	221	236	0	89.14	35
1157.6441	2	1157.6445	-0.0004	191	202	0	39.64	37
1270.6777	2	1270.6782	-0.0005	208	219	0	50.33	36
1419.8193	3	1419.8198	-0.0004	112	124	1	47.55	30
2028.0526	2	2028.0527	-0.0001	246	264	0	119.47	36
2141.0147	3	2141.0164	-0.0017	40	56	1	42.27	37
2340.2611	3	2340.2689	-0.0078	243	264	1	54.67	34
1240.5684	2	1240.5659	0.0025	361	371	0	35.94	35
2279.1526	2	2279.1533	-0.0006	88	109	0	154.35	37
3134.5471	3	3134.5442	0.0029	325	354	0	65.26	36
1072.5603	2	1072.5625	-0.0022	99	108	0	39.79	38

1157.6045	2	1157.604	0.0005	313	322	0	59.18	36
1298.7554	2	1298.7558	-0.0004	284	295	0	107.82	33
2203.1801	3	2202.1757	1.0044	52	73	0	74.01	35
1182.5923	2	1182.5921	0.0003	747	756	0	45.65	36
1196.5824	2	1196.5826	-0.0001	138	148	0	78.82	37
1309.6382	3	1309.6375	0.0007	163	174	0	42.92	37
2206.0268	2	2206.0324	-0.0056	245	263	0	93.48	36
2326.1875	3	2326.1879	-0.0004	531	550	0	37.4	37
2406.2446	2	2406.2471	-0.0025	75	97	0	49.34	36
1242.6563	2	1242.6568	-0.0005	426	435	0	40.02	36
1382.6864	2	1382.6864	0	626	638	0	84.45	37
2216.3035	3	2216.3031	0.0004	378	398	0	43.79	23
2302.1838	2	2302.1845	-0.0007	399	420	0	117.74	36
2362.2586	2	2362.2607	-0.002	86	106	0	53.08	34
2561.3913	3	2561.3927	-0.0015	84	106	1	75.03	32
2806.3535	3	2805.3545	0.999	45	69	0	54.79	37
975.5513	2	975.5502	0.0011	250	258	0	48.8	36
1029.5822	2	1029.5818	0.0004	57	66	0	45.91	37
1381.6828	2	1381.6838	-0.001	164	176	0	86.39	38
1658.9166	3	1657.9151	1.0014	57	71	1	56.17	34
1686.9343	2	1686.9345	-0.0002	420	434	0	45.45	34
856.4554	2	856.4555	-0.0002	61	68	0	40.71	34
959.5804	2	959.5804	0	711	718	0	44.81	32
1005.5605	2	1005.5607	-0.0002	927	935	0	42.89	36
1035.5506	2	1035.5502	0.0004	771	779	0	54.82	37
1485.8399	2	1485.8402	-0.0003	109	122	0	84.97	34
1746.8889	2	1746.89	-0.0011	32	48	0	95.3	38
713.4429	2	713.4435	-0.0007	430	435	0	44.42	30
1255.6881	2	1255.6884	-0.0003	88	99	0	46.83	35
1290.6352	2	1290.6303	0.0049	34	45	0	49.8	38
1319.6519	2	1319.651	0.0009	299	309	0	45.95	38
1526.7936	2	1526.794	-0.0004	358	371	0	106.65	36
1582.7823	3	1582.7839	-0.0015	441	454	1	56.92	37
1064.5906	2	1064.5906	-0.0001	15	23	0	41.84	34
3020.2786	3	3020.2785	0.0001	81	111	0	75.59	29
3431.7198	4	3431.7184	0.0014	268	297	1	64.88	36
1614.8385	2	1614.8399	-0.0014	26	40	0	75.85	37
1760.8772	2	1759.8774	0.9997	41	57	0	99.41	38
1614.8976	2	1614.8981	-0.0005	81	95	0	112.19	35
1865.1018	2	1865.1026	-0.0008	852	869	0	59.99	23
2326.26	3	2326.2606	-0.0006	284	304	0	73.47	33
1180.5716	2	1180.5724	-0.0008	410	420	0	84.19	37
1291.5698	2	1291.5714	-0.0016	399	409	0	55.13	33
1528.8074	2	1528.8079	-0.0005	278	291	0	68.57	37
1535.9546	2	1535.9538	0.0008	559	572	0	60.93	20
2292.3456	3	2292.3457	0	559	579	1	27.64	22
1045.6131	2	1045.6131	-0.0001	372	381	0	68.94	33
1555.7632	2	1555.763	0.0001	210	223	0	66.95	37
1854.0512	2	1854.0502	0.001	437	453	0	63.12	30
3201.6481	3	3201.6517	-0.0037	405	436	0	77.9	35
1157.6409	2	1157.6405	0.0005	351	361	0	38.52	37
1248.6121	2	1248.6132	-0.0011	135	146	0	84.92	38
1426.792	2	1426.7919	0.0001	260	272	0	88.04	35
1655.8988	2	1655.8995	-0.0007	273	288	0	39.16	35
2443.3872	3	2443.3872	-0.0001	7	29	0	66.36	27
1026.6078	2	1026.6073	0.0005	182	191	0	39.39	30
1056.6542	2	1056.6543	-0.0001	282	291	0	49.46	29
1074.592	2	1074.5921	0	248	258	0	60.53	37

1127.5939	2	1127.5934	0.0005	265	275	0	68.79	35
1130.5558	2	1130.5567	-0.0009	227	236	0	54.58	36
1209.6465	3	1209.6466	0	237	247	1	36.7	35
1523.7988	2	1523.7984	0.0005	303	316	0	74.75	37
995.6127	2	995.6127	-0.0001	311	319	0	36.81	26
1003.5819	2	1003.5814	0.0005	471	479	0	64.4	34
1413.7195	2	1413.7212	-0.0017	193	204	0	66.6	36
1880.9353	2	1880.9342	0.0011	445	460	0	112.04	37
1034.5476	2	1034.5509	-0.0033	413	423	0	54.71	37
1204.5464	2	1204.5473	-0.0009	980	990	0	44.61	35
1370.7921	2	1370.7922	-0.0001	483	494	0	55.55	33
1638.8435	2	1638.8465	-0.0029	776	791	0	42.69	37
1798.815	2	1798.8156	-0.0006	918	935	0	107.6	35
1045.5981	2	1045.6019	-0.0037	375	384	0	53.66	34
1189.6455	2	1189.6455	0	751	760	0	41.35	38
1459.7305	2	1459.7307	-0.0002	697	709	0	58.99	38
1518.7825	2	1518.7831	-0.0006	240	252	0	56.93	38
1848.8338	2	1848.8337	0	903	920	0	70.03	35
2004.9336	3	2004.9348	-0.0013	903	921	1	40.09	36
2088.0474	3	2088.0487	-0.0013	217	234	1	50.28	37
977.5183	2	977.5182	0.0001	37	44	0	46.67	36
1530.7167	2	1530.7161	0.0006	86	99	0	84.88	36
1681.8333	2	1681.8312	0.0021	320	334	0	66.01	37
3076.5394	3	3075.5256	1.0137	45	72	0	49.75	36
1015.5181	2	1015.5186	-0.0005	50	59	0	38.47	36
1195.728	2	1195.7288	-0.0008	469	479	0	60.89	28
1266.5687	2	1266.5696	-0.001	137	148	0	76.17	35
1507.7583	2	1507.7592	-0.0009	213	225	0	102.64	38
1356.8013	2	1356.8017	-0.0004	157	169	0	53.43	30
1368.6243	2	1368.6245	-0.0002	224	234	0	39.7	35
1526.892	2	1526.892	0.0001	432	445	0	86.45	30
1924.1724	3	1923.1669	1.0054	293	310	0	51.52	20
2994.4189	3	2993.4142	1.0047	254	280	0	41.85	36
1112.6441	2	1112.6441	0	821	831	0	106.81	33
1814.9358	2	1814.9342	0.0016	299	313	0	78.78	37
2134.9998	3	2134.0007	0.9991	241	257	0	46.88	36
842.4847	2	842.4862	-0.0015	2	10	0	60.6	34
1180.6923	2	1180.6928	-0.0005	129	139	0	90.73	30
1549.7561	2	1549.7559	0.0002	115	128	0	83.17	38
1097.5871	2	1097.5869	0.0002	225	233	1	39.83	34
1143.6573	2	1143.6573	0	282	291	0	61.26	35
1182.6249	2	1182.6244	0.0005	433	443	0	57.85	36
1259.6753	2	1259.6761	-0.0008	468	478	0	60.29	37
1329.7077	2	1329.7075	0.0002	234	246	0	83.89	37
1053.5813	2	1053.5819	-0.0006	100	109	0	34.94	34
1334.6832	2	1334.683	0.0002	524	535	0	82.57	38
1642.8357	2	1642.8355	0.0002	763	777	0	95.53	37
2723.4532	3	2722.4502	1.0031	812	839	0	45.2	33
1243.7745	2	1243.7751	-0.0006	344	355	0	90.04	23
1293.7119	2	1293.7115	0.0004	401	411	0	43.07	34
1431.6756	2	1431.6742	0.0014	179	190	0	73.64	36
2349.3775	3	2349.3784	-0.0009	289	309	1	39.61	23
1319.6221	3	1319.6218	0.0003	372	383	0	43.63	36
1554.7791	2	1554.7786	0.0006	21	35	0	95.38	37
1927.9412	3	1927.9422	-0.001	384	400	0	47.13	37
2521.3752	3	2521.3751	0.0001	437	461	0	62.97	31
1852.0557	2	1852.0557	0	348	364	0	134.2	30
2008.1567	3	2008.1568	-0.0002	347	364	1	54.65	27

1788.0299	2	1788.0284	0.0014	760	775	0	81.16	29
2312.3651	3	2311.3603	1.0049	935	954	0	50.51	23
948.5395	2	948.5392	0.0003	197	205	0	60.2	35
1059.5048	2	1059.5131	-0.0082	160	169	0	55.1	35
1218.6492	2	1218.6496	-0.0004	112	121	0	53.51	37
1407.7514	2	1407.7511	0.0003	95	106	0	82.26	36
1842.9423	2	1842.9435	-0.0013	269	286	1	39.61	37
1340.7569	2	1340.7565	0.0005	511	523	0	89.46	34
1630.8664	2	1630.8679	-0.0015	227	242	0	97.58	37
1939.0577	2	1939.0561	0.0016	278	296	0	78.4	34
2959.478	3	2958.4757	1.0023	120	147	0	71.93	36
881.4397	2	881.4396	0.0001	727	733	0	40.5	36
1228.5721	2	1228.5724	-0.0004	499	509	0	63.61	35
1228.7176	2	1228.718	-0.0003	593	603	0	59	34
1395.7244	2	1395.7245	-0.0001	604	616	0	40.81	37
1983.0258	3	1982.0208	1.005	405	423	0	61.23	36
2896.4617	3	2896.4607	0.001	745	770	1	42.68	36
1818.893	2	1818.8921	0.0009	26	42	0	93.59	38
2409.2088	3	2409.2097	-0.0009	26	47	1	40.8	37
1033.5226	2	1033.5226	0	344	352	0	59.47	38
1443.6485	2	1443.6491	-0.0006	63	76	0	90.83	33
1861.0594	3	1861.0607	-0.0013	127	145	0	47.12	29
1038.6072	2	1038.6073	-0.0001	64	72	0	46.86	29
2652.3999	3	2651.3919	1.008	319	341	0	87.54	34
869.5333	2	869.5334	-0.0001	236	243	0	51.76	30
1009.5919	2	1009.592	-0.0001	417	425	0	31.86	29
1460.7607	2	1460.7623	-0.0016	185	197	0	102.22	38
1578.8005	2	1578.8042	-0.0037	133	146	0	44.56	38
1750.8853	2	1750.889	-0.0037	244	259	0	48.49	37
1300.7018	2	1300.7027	-0.0009	157	168	0	90.56	37
1306.641	2	1306.6418	-0.0008	146	156	0	61.34	38
1372.6765	2	1372.6769	-0.0004	133	144	0	72.3	37
1455.7672	2	1455.7722	-0.0049	158	171	0	59.76	36
1547.7949	2	1547.7944	0.0006	94	108	0	72.57	38
1557.8726	2	1557.8726	0	342	356	0	80.66	34
1075.5075	2	1075.508	-0.0005	160	169	0	59.89	37
1436.7542	2	1436.7525	0.0018	255	268	0	50.9	36
1464.773	2	1464.7725	0.0005	95	106	0	92.49	37
1608.7969	2	1608.7995	-0.0026	216	231	0	46.25	38
856.4763	2	856.4766	-0.0004	35	42	0	39.99	33
1042.556	2	1042.556	0.0001	668	676	0	49.56	36
1235.5793	2	1235.5782	0.0011	1039	1049	0	47.43	37
1340.6613	2	1340.6612	0	164	174	0	65.84	36
1670.9267	2	1670.9276	-0.001	891	905	0	72.25	34
977.5311	2	977.5294	0.0017	520	527	0	39.01	37
1715.0035	2	1715.0022	0.0013	2116	2129	0	34.75	27
1778.9523	3	1778.9527	-0.0004	856	871	0	48.52	35
1780.9998	2	1781.0047	-0.0048	1464	1479	0	78.82	31
2408.297	3	2407.292	1.005	2201	2222	0	59.85	33
798.4967	2	798.4963	0.0004	134	140	0	33.31	31
1071.6441	2	1071.6441	0.0001	934	943	0	65.91	34
1227.6733	3	1227.6724	0.0009	411	422	0	43.29	36
1612.8563	2	1612.8573	-0.001	646	659	0	74.96	36
1855.9528	3	1855.9541	-0.0012	611	626	1	38.15	37
1578.7002	2	1578.6984	0.0018	1940	1952	0	46.8	34
1944.0317	2	1944.0316	0.0001	2070	2087	0	116.01	36
1180.7181	2	1180.718	0.0001	174	184	0	63.83	26
1232.6895	2	1232.6877	0.0018	397	408	0	57.84	35

1458.7602	2	1458.7678	-0.0076	361	374	0	42.99	37
1881.0966	3	1881.0975	-0.0009	147	164	0	43.47	27
1284.7695	2	1284.7693	0.0002	2912	2922	0	41.11	30
1550.9073	2	1550.9072	0.0001	1927	1940	0	127.93	28
1714.9035	3	1714.9036	-0.0001	657	671	1	37.61	37
2355.1436	2	2355.1457	-0.002	630	650	0	81.03	37
1267.6049	2	1267.6044	0.0004	51	61	0	40.53	37
1948.8579	2	1948.8578	0.0001	396	411	0	100.26	33
2219.1296	2	2219.1263	0.0034	365	382	0	68.09	37
1808.9859	3	1808.9859	0.0001	439	453	1	36.54	33
1871.9406	2	1871.9411	-0.0005	675	692	0	98.1	37
4025.0233	3	4025.0269	-0.0037	220	256	1	55.44	34
1757.9776	2	1757.9774	0.0002	73	88	0	95.74	34
1966.0012	3	1966.0021	-0.0009	359	377	1	76.1	37
1138.707	2	1138.7074	-0.0004	831	840	0	66.75	25
1414.7812	2	1414.782	-0.0008	718	729	0	84.01	36
1957.9715	3	1956.9687	1.0028	81	98	0	47	37
980.5699	2	980.5695	0.0004	100	107	0	33.74	31
1762.8828	2	1762.8836	-0.0008	12	27	0	97.03	38
2113.1518	3	2113.1531	-0.0013	55	72	1	62.17	34
906.5495	3	906.5511	-0.0017	351	357	1	26.86	25
961.5235	2	961.5233	0.0003	322	329	0	68.12	37
1038.6074	2	1038.6073	0.0001	290	298	0	32.15	29
1111.5304	2	1111.5298	0.0006	335	343	0	43.15	36
1548.8403	3	1548.8412	-0.001	309	321	1	51.53	35
2028.9004	2	2028.9025	-0.002	418	435	0	63.38	34
1256.7334	2	1256.734	-0.0005	84	95	0	96.84	33
1383.7043	2	1383.7034	0.0009	26	37	0	69.13	36
1484.7447	2	1484.7471	-0.0024	428	441	0	55.63	37
2005.9582	2	2005.9574	0.0008	277	292	0	69.71	37
2017.0332	2	2017.0368	-0.0035	120	138	0	76.22	37
976.5085	2	976.509	-0.0005	390	397	0	40.11	38
1228.7025	2	1228.7027	-0.0002	609	619	0	79.6	35
1566.9344	2	1566.9345	-0.0001	430	444	0	77.93	27
1036.5186	2	1036.5189	-0.0003	110	118	0	56.35	37
1227.6733	3	1227.6724	0.0009	411	422	0	43.29	36
1612.8563	2	1612.8573	-0.001	646	659	0	74.96	36
1855.9528	3	1855.9541	-0.0012	611	626	1	38.15	37
897.4926	2	897.492	0.0007	24	31	0	55.89	33
1169.5962	2	1169.5968	-0.0006	178	187	0	58.79	35
1581.8458	2	1581.8475	-0.0016	75	89	0	75.02	35
2626.0509	3	2626.053	-0.0021	42	65	0	58.14	26
2901.2141	3	2901.2164	-0.0023	40	65	1	90.58	29
1266.7902	2	1266.7911	-0.001	72	84	0	42.62	23
1686.7954	2	1686.7962	-0.0008	101	115	0	93.18	37
1091.5977	2	1091.5975	0.0002	6	15	0	51.49	36
1660.893	3	1660.8937	-0.0007	488	502	0	43.13	36
2166.1072	2	2165.0946	1.0127	856	872	0	87.82	37
3074.5585	3	3074.5594	-0.0009	194	222	0	43.82	36
1187.655	2	1187.6584	-0.0034	295	304	0	95.87	37
1746.8772	2	1746.8788	-0.0016	386	401	0	61.43	38
1367.6732	2	1367.6755	-0.0023	388	399	0	78.77	37
1410.8019	3	1410.8024	-0.0005	409	419	1	40.52	33
1722.018	2	1722.0192	-0.0012	91	105	0	48.67	26
3118.711	3	3118.71	0.001	617	644	0	44.18	29
797.5121	2	797.5123	-0.0002	109	116	0	28.89	26
1287.6591	2	1287.6605	-0.0014	95	106	0	74.65	37
1422.6586	2	1422.6627	-0.0041	28	40	0	86.33	36

1933.0249	2	1933.023	0.0019	53	70	0	96.22	36
2089.0871	3	2089.0877	-0.0007	110	127	1	65.09	37
2423.1601	3	2423.1618	-0.0017	80	99	1	46.58	37
1048.5191	2	1048.5189	0.0002	314	322	0	45.16	38
1225.7024	2	1225.703	-0.0006	469	480	0	98.74	32
1752.9573	2	1752.9563	0.001	363	378	0	38.76	33
1188.6342	2	1188.635	-0.0008	182	194	0	74.18	38
1342.7081	2	1342.7092	-0.0012	195	209	0	58.54	37
1351.6355	2	1351.6368	-0.0013	331	342	0	55.66	37
1425.7568	2	1425.7576	-0.0007	132	144	0	55.16	36
1759.8941	2	1759.8952	-0.0011	9	25	0	97.79	37
1383.7706	2	1383.7722	-0.0015	283	297	0	58.64	34
1775.9008	2	1774.8924	1.0085	355	370	0	95.34	38
1201.6808	2	1201.6819	-0.0011	375	385	0	51.9	35
1534.9459	2	1534.9447	0.0013	810	823	0	80.83	20
2186.2129	3	2186.2133	-0.0004	1687	1707	1	36.1	31
1190.5949	2	1190.5931	0.0018	177	187	0	47.85	38
1265.7346	2	1265.7343	0.0003	343	353	0	41.12	30
1512.8149	2	1512.8147	0.0002	259	272	0	97.32	36
1053.5551	2	1053.5567	-0.0016	106	116	0	71.37	35
1156.6451	2	1156.6452	-0.0001	11	21	0	42.7	36
1442.7237	2	1442.7253	-0.0016	128	140	0	73.62	37
754.5064	2	754.5065	-0.0001	102	107	0	46.59	20
773.5159	2	773.5163	-0.0005	83	88	0	45.75	22
1570.8564	2	1570.8566	-0.0002	161	175	0	67.33	35
1159.6712	2	1159.6713	-0.0001	148	158	0	85.23	34
2924.5348	3	2924.5357	-0.0009	321	348	0	59.06	34
1945.9501	2	1945.9495	0.0006	165	181	0	76.3	37
2187.2479	3	2187.2487	-0.0009	97	115	1	64.84	27
1141.6821	2	1141.6819	0.0002	444	454	0	89.08	31
1832.8259	2	1832.8217	0.0042	221	236	0	50.06	35
1155.6974	2	1155.6975	-0.0001	478	488	0	95.41	32
2182.1858	3	2182.1899	-0.0041	380	398	1	41.1	33
1129.5719	2	1129.5728	-0.0008	85	95	0	71.84	36
1255.6622	2	1255.6633	-0.0011	123	136	0	46.74	35
2473.3034	2	2473.3064	-0.003	278	300	0	51.28	35
1176.6138	2	1176.6139	0	510	519	0	70.82	38
1297.681	2	1297.6813	-0.0002	207	218	0	47.74	35
1499.8964	2	1499.8963	0.0001	131	144	0	35.25	27
2014.0283	3	2014.0306	-0.0023	238	254	1	38.98	37
1138.5723	2	1138.5731	-0.0007	973	981	0	56.38	36
1872.8382	2	1872.8391	-0.0009	582	597	0	63.38	34
1954.9657	3	1954.965	0.0008	982	997	0	48.72	37
1487.7083	2	1487.7104	-0.0021	283	295	0	97.92	36
1958.9597	2	1958.9625	-0.0028	172	188	0	42.19	37
1149.5975	2	1149.599	-0.0014	127	137	0	84.43	37
1315.717	2	1315.7169	0.0001	411	421	0	58.75	37
1560.715	2	1560.7168	-0.0018	168	181	0	69.11	35
1703.8822	2	1703.8842	-0.002	79	93	0	68.15	37
1162.6202	2	1161.6142	1.006	161	171	0	55.42	37
1905.9945	2	1904.9918	1.0027	198	215	0	75.78	37
843.5218	2	843.5218	0	387	393	0	44.18	35
1087.5695	2	1087.5696	0	377	386	0	42.79	38
1241.6287	2	1241.6292	-0.0005	412	422	0	60.25	36
2655.3619	3	2655.3656	-0.0037	204	227	1	45.47	36
1031.6125	2	1031.6128	-0.0003	482	490	0	51.34	32
2366.3832	2	2366.3824	0.0008	329	352	0	74.12	21
1024.5708	2	1024.5706	0.0003	303	310	0	34.02	33

1103.6085	2	1103.6087	-0.0002	227	236	0	51.97	36
1151.5919	2	1151.5934	-0.0016	130	140	0	79.95	37
1217.5875	2	1217.5888	-0.0013	1502	1513	0	41.44	36
2119.1148	2	2119.1161	-0.0013	1116	1135	0	92.25	36
1028.5984	2	1028.5978	0.0005	93	102	0	56.73	35
1214.6508	2	1214.6506	0.0001	390	400	0	80.13	37
1057.5766	2	1057.5768	-0.0001	1865	1874	0	43.42	36
1120.6235	2	1120.624	-0.0005	2101	2110	0	39.03	35
1796.8401	2	1796.8369	0.0032	1395	1408	0	43.26	36
2139.1576	3	2139.1576	0.0001	1496	1515	0	49.3	34
2183.0465	3	2183.0494	-0.003	885	903	0	55.65	37
1044.4694	2	1044.4699	-0.0005	208	216	0	44.23	32
1144.59	2	1144.591	-0.001	134	142	0	73.2	37
2882.4282	2	2882.4284	-0.0002	94	120	0	38.95	37
1324.6629	2	1324.6623	0.0006	1042	1053	0	68.24	37
1792.9923	2	1792.9935	-0.0011	160	175	0	50.1	32
2230.2471	3	2230.2473	-0.0002	453	473	0	32.27	30
1241.759	2	1241.7595	-0.0005	1064	1075	0	79.22	28
1716.8701	2	1715.873	0.9971	600	615	0	48.77	38
1205.572	2	1205.5717	0.0003	434	443	0	56.15	37
1450.8869	3	1450.8871	-0.0002	388	400	1	28.48	22
1522.669	2	1522.6689	0.0002	185	197	0	69.34	34
939.5755	2	939.5753	0.0002	125	132	0	58.14	28
2080.9272	3	2080.9273	-0.0001	107	124	0	63.45	34
1009.5345	2	1009.5345	0	888	895	0	47	36
1012.6537	3	1012.6546	-0.0009	401	408	1	31.62	26
1295.7553	2	1295.7561	-0.0009	637	648	0	42.6	31
1730.9657	3	1729.9627	1.0029	194	207	1	62.27	33
1325.6989	2	1325.6979	0.001	371	381	0	55.2	35
1394.7621	2	1394.763	-0.0009	232	244	0	70.63	34
1573.9489	2	1573.9483	0.0005	140	153	0	44.35	23
1652.9861	2	1652.9865	-0.0004	435	448	0	61.08	25
1859.0916	3	1859.092	-0.0004	140	155	1	35.3	24
1072.623	2	1072.6241	-0.0011	42	52	0	60.45	36
1972.1254	3	1972.1258	-0.0004	17	33	0	52.24	29
1388.6714	2	1387.6812	0.9901	240	250	0	51.76	36
1654.8424	2	1653.8363	1.0061	206	220	0	69.34	36
1597.8685	2	1597.8684	0.0002	90	102	0	56.48	35
2126.0438	3	2126.0433	0.0005	662	679	0	62.77	37
1410.8337	2	1410.8334	0.0003	153	165	0	41.54	28
3549.907	3	3549.9083	-0.0014	116	149	0	71.6	30
1166.6033	2	1166.6044	-0.001	319	328	0	51.53	37
1410.6253	2	1410.6263	-0.0009	329	341	0	66.49	34
916.4444	2	916.4443	0.0001	490	496	0	46.78	35
1258.6519	2	1258.6517	0.0001	335	345	0	66.19	38
822.4387	2	822.4388	-0.0001	730	736	0	39.33	36
1153.682	2	1153.6819	0.0001	442	451	0	67.88	31
1649.9967	2	1649.9967	0	21	35	0	30.48	22
1259.541	2	1259.5419	-0.0008	304	315	0	46.81	31
1647.8022	3	1647.8045	-0.0024	316	328	1	61.96	38
1720.9599	2	1720.9512	0.0087	738	752	0	69.43	32
756.4492	2	756.4494	-0.0002	585	590	0	37.61	33
1225.6314	2	1225.6303	0.0011	378	389	0	41.85	36
1510.8334	3	1510.8355	-0.0021	182	196	0	59.12	34
1094.5886	2	1094.5873	0.0013	32	40	0	49.27	36
2886.364	3	2886.3613	0.0027	456	482	0	59.02	37
1028.5604	2	1028.5614	-0.001	855	863	0	41.42	37
1347.6811	2	1347.6823	-0.0012	726	737	0	70.36	38

1103.5395	2	1103.5393	0.0002	390	399	0	40.45	37
1247.635	2	1247.6366	-0.0016	508	518	0	54.91	39
1343.6985	2	1343.698	0.0005	450	461	0	54.45	37
1444.6417	2	1444.6412	0.0006	177	187	0	43.54	33
1579.7547	2	1579.7552	-0.0005	164	176	0	62.37	37
948.5644	2	948.5644	0	36	43	0	35.2	30
2004.9626	2	2004.9609	0.0018	16	35	0	70.42	37
1075.5608	2	1075.5622	-0.0014	260	270	0	68.07	39
2859.496	3	2858.5079	0.9881	717	741	0	35.99	34
949.4806	2	949.4804	0.0003	85	92	0	45.09	38
1056.6292	2	1056.6292	0.0001	57	67	0	62.54	34
1235.6125	2	1235.6146	-0.002	16	26	1	39.04	38
1368.6243	2	1368.6245	-0.0002	223	233	0	39.7	35
1913.1466	3	1913.1462	0.0004	292	309	0	47.42	21
1159.6194	2	1159.6197	-0.0003	50	61	0	67.99	38
1165.5993	2	1165.5979	0.0014	66	76	0	38.57	37
1368.6243	2	1368.6245	-0.0002	227	237	0	39.7	35
1955.1571	3	1955.1568	0.0004	296	313	0	54.95	26
1046.5768	2	1046.576	0.0007	72	80	0	37.41	37
2941.506	3	2941.5073	-0.0013	85	109	1	64.27	35
1145.6039	2	1145.604	-0.0001	50	61	0	66.41	39
1165.5993	2	1165.5979	0.0014	66	76	0	38.57	37
1340.7036	2	1340.7048	-0.0012	53	65	0	46.96	36
1908.0318	2	1908.0316	0.0001	616	635	0	51.89	34
1252.6517	2	1252.6524	-0.0007	31	42	0	51.19	36
1813.971	2	1813.9713	-0.0003	200	215	0	42.74	36
880.5752	2	880.5746	0.0006	266	274	1	23.2	20
1301.6146	2	1301.614	0.0006	247	258	0	67.47	35
1003.5317	2	1003.5298	0.0019	100	109	0	39.39	38
2487.2408	3	2487.2434	-0.0026	437	458	0	53.53	37
1072.483	2	1072.4825	0.0005	280	288	0	44.22	32
1216.681	2	1216.6815	-0.0005	183	193	0	48.51	36
828.5066	2	828.5069	-0.0003	115	121	0	34.71	34
1380.8447	3	1380.8453	-0.0006	43	55	1	39.4	26
1391.6225	2	1391.6239	-0.0014	28	39	0	41.17	34
1209.6399	2	1209.6394	0.0006	146	155	0	50.67	36
1239.6602	2	1239.6612	-0.001	285	296	0	38.27	35
943.5337	2	943.5338	-0.0001	246	253	0	37.82	37
1467.8596	3	1467.8595	0	38	49	1	48.99	30
1015.5248	2	1015.5233	0.0015	164	174	0	39.69	37
2001.9449	3	2001.9392	0.0057	129	147	0	42.76	37
1132.5093	2	1132.5084	0.0009	300	309	0	36.87	33
1239.6822	2	1239.6823	-0.0001	203	213	0	40.23	34
1067.5978	2	1067.5975	0.0003	494	503	0	36.58	33
2569.3638	3	2569.364	-0.0002	170	194	0	40.54	34
1169.5568	2	1169.5564	0.0004	182	191	0	38.85	35
1250.6247	2	1250.625	-0.0003	752	763	0	41.03	38
963.4404	2	963.441	-0.0005	100	107	0	40.34	35
1694.993	3	1694.9931	-0.0001	53	67	1	29.38	28
1453.7286	3	1453.7273	0.0013	77	89	1	38.34	37
3956.0566	3	3956.0571	-0.0006	213	248	0	33.25	33

pep_exp_mr	pep_exp_z	pep_calc_mr	pep_delta	pep_start	pep_end	pep_miss	pep_score	pep_ident
975.4696	2	975.4695	0.0001	351	359	0	71	37
1026.5499	2	1026.5498	0.0001	310	318	0	69.41	34
1138.6861	2	1138.6862	-0.0001	253	262	0	64.51	28
1145.5818	2	1145.5829	-0.0011	242	251	0	50.37	38
1214.5747	2	1214.5754	-0.0007	381	390	0	73.61	35
1298.6137	2	1298.6143	-0.0006	47	58	0	76.81	35

1401.6491	2	1401.6495	-0.0004	163	174	0	71.54	35
1621.8443	2	1621.8439	0.0004	263	276	0	66.69	37
1646.8003	2	1646.8008	-0.0005	63	77	0	68.18	37
1695.8247	2	1695.8257	-0.0009	337	350	0	82.13	37
1821.9147	2	1821.9156	-0.0009	3	19	0	108.91	38
1950.8784	2	1950.8815	-0.0031	363	379	0	132.92	35
1971.988	2	1971.9902	-0.0021	104	121	0	119.57	37
2366.1208	2	2366.1212	-0.0004	277	297	0	71.56	37
2763.3138	3	2763.3143	-0.0005	217	241	0	82.33	37
2797.3318	3	2797.3361	-0.0043	78	103	1	69.61	37
3199.4629	3	3199.4669	-0.0041	20	46	0	85.8	35
3309.5026	3	3309.5064	-0.0039	123	154	0	93.24	34
1058.5211	2	1058.5219	-0.0008	310	318	0	41	35
1138.6861	2	1138.6862	-0.0001	253	262	0	64.51	28
1145.5818	2	1145.5829	-0.0011	242	251	0	50.37	38
1214.5747	2	1214.5754	-0.0007	381	390	0	73.61	35
1295.5991	2	1295.5994	-0.0003	36	46	0	75.48	36
1383.6927	2	1383.6931	-0.0004	163	174	0	58.76	36
1444.6401	2	1444.6405	-0.0004	47	58	0	102.88	33
1445.6802	2	1445.682	-0.0018	325	336	0	83.14	36
1621.8443	2	1621.8439	0.0004	263	276	0	66.69	37
1646.8003	2	1646.8008	-0.0005	63	77	0	68.18	37
1695.8247	2	1695.8257	-0.0009	337	350	0	82.13	37
1821.9147	2	1821.9156	-0.0009	3	19	0	108.91	38
1950.8784	2	1950.8815	-0.0031	363	379	0	132.92	35
1971.988	2	1971.9902	-0.0021	104	121	0	119.57	37
2801.2908	3	2801.2946	-0.0038	78	103	1	93.09	35
1214.6445	2	1214.6441	0.0004	164	175	0	85.22	37
1225.583	2	1225.5827	0.0004	81	91	0	63.08	36
1227.621	2	1227.6207	0.0003	29	39	0	61.25	36
1277.6219	2	1277.6227	-0.0008	243	252	0	45.69	38
1294.6563	2	1294.6557	0.0006	308	317	0	77.81	37
1307.6972	2	1307.6972	-0.0001	133	144	0	68.59	36
1357.6075	2	1357.6084	-0.0009	546	556	0	70.43	34
1385.7209	2	1385.7224	-0.0015	604	615	0	60.24	37
1406.7556	2	1406.7558	-0.0002	352	363	0	86.71	35
1430.7364	2	1430.7365	-0.0001	335	348	0	98.27	37
1472.6771	2	1472.6783	-0.0013	40	52	0	78.71	35
1521.7938	3	1521.7939	-0.0001	306	317	1	50.34	37
1595.8119	2	1595.8116	0.0003	117	130	0	73.97	37
1660.8246	2	1660.8243	0.0003	60	74	0	106.86	38
1674.722	2	1674.7234	-0.0014	227	242	0	93.66	32
1679.827	2	1679.8267	0.0002	145	159	0	51.17	38
2573.2622	2	2573.2609	0.0014	368	393	0	155.4	37
2587.2103	3	2587.2126	-0.0022	5	28	0	39.36	36
2657.2616	2	2657.2609	0.0007	431	453	0	81.43	37
1058.5211	2	1058.5219	-0.0008	310	318	0	41	35
1138.6861	2	1138.6862	-0.0001	253	262	0	64.51	28
1145.5818	2	1145.5829	-0.0011	242	251	0	50.37	38
1214.5747	2	1214.5754	-0.0007	381	390	0	73.61	35
1383.6927	2	1383.6931	-0.0004	163	174	0	58.76	36
1430.6232	2	1430.6248	-0.0016	47	58	0	83.6	32
1445.6802	2	1445.682	-0.0018	325	336	0	83.14	36
1621.8443	2	1621.8439	0.0004	263	276	0	66.69	37
1632.7856	2	1632.7851	0.0004	63	77	0	57.77	37
1695.8247	2	1695.8257	-0.0009	337	350	0	82.13	37
1821.9147	2	1821.9156	-0.0009	3	19	0	108.91	38
1950.8784	2	1950.8815	-0.0031	363	379	0	132.92	35

1971.988	2	1971.9902	-0.0021	104	121	0	119.57	37
2813.329	3	2813.331	-0.002	78	103	1	66.36	36
1214.6445	2	1214.6441	0.0004	164	175	0	85.22	37
1227.621	2	1227.6207	0.0003	29	39	0	61.25	36
1229.5228	2	1229.5234	-0.0006	81	91	0	50.96	31
1277.6219	2	1277.6227	-0.0008	243	252	0	45.69	38
1312.6117	2	1312.6122	-0.0004	308	317	0	73.34	36
1357.6075	2	1357.6084	-0.0009	546	556	0	70.43	34
1385.7209	2	1385.7224	-0.0015	604	615	0	60.24	37
1425.7564	2	1425.7576	-0.0012	335	348	0	57.93	36
1435.7452	2	1435.746	-0.0007	352	363	0	57.43	37
1472.6771	2	1472.6783	-0.0013	40	52	0	78.71	35
1539.7502	3	1539.7504	-0.0002	306	317	1	58.19	37
1565.8008	2	1565.8011	-0.0002	117	130	0	93.03	38
1662.802	2	1662.8036	-0.0015	60	74	0	102.92	37
1674.722	2	1674.7234	-0.0014	227	242	0	93.66	32
1679.827	2	1679.8267	0.0002	145	159	0	51.17	38
2559.2432	2	2559.2452	-0.002	368	393	0	137.79	37
2587.2103	3	2587.2126	-0.0022	5	28	0	39.36	36
2657.2616	2	2657.2609	0.0007	431	453	0	81.43	37
1058.5211	2	1058.5219	-0.0008	310	318	0	41	35
1138.6861	2	1138.6862	-0.0001	253	262	0	64.51	28
1145.5818	2	1145.5829	-0.0011	242	251	0	50.37	38
1214.5747	2	1214.5754	-0.0007	381	390	0	73.61	35
1307.6346	2	1307.6357	-0.0011	36	46	0	50.04	38
1401.6491	2	1401.6495	-0.0004	163	174	0	71.54	35
1430.6232	2	1430.6248	-0.0016	47	58	0	83.6	32
1445.6802	2	1445.682	-0.0018	325	336	0	83.14	36
1621.8443	2	1621.8439	0.0004	263	276	0	66.69	37
1632.7856	2	1632.7851	0.0004	63	77	0	57.77	37
1695.8247	2	1695.8257	-0.0009	337	350	0	82.13	37
1950.8784	2	1950.8815	-0.0031	363	379	0	132.92	35
1971.988	2	1971.9902	-0.0021	104	121	0	119.57	37
2813.329	3	2813.331	-0.002	78	103	1	66.36	36
986.5398	2	986.5397	0.0001	327	336	0	69.57	37
1266.5012	2	1266.5018	-0.0006	312	320	0	52.43	26
1395.7494	2	1395.751	-0.0016	85	96	0	40	36
1472.8555	2	1472.8562	-0.0007	230	243	0	99.98	30
1597.7601	2	1597.7599	0.0002	340	352	0	77.52	37
1662.8671	2	1662.8689	-0.0017	216	229	1	52.99	37
1700.8972	2	1700.8985	-0.0013	65	79	0	76.13	37
1791.9195	2	1791.9229	-0.0034	265	280	0	111.16	38
1856.8737	2	1856.8761	-0.0023	374	390	0	113.24	37
1884.9395	2	1884.9404	-0.0009	353	370	0	88.78	37
1976.8744	2	1976.8752	-0.0009	41	60	0	102.84	33
2329.0111	3	2329.011	0.0002	403	422	0	67.36	32
2394.1846	2	2394.1856	-0.0009	244	264	0	105.2	37
2624.2226	3	2624.225	-0.0025	281	304	0	48.97	36
934.512	2	934.5124	-0.0004	129	137	0	49.95	35
957.5131	2	957.5131	0.0001	118	125	0	51.34	36
975.5498	2	975.5502	-0.0004	376	384	0	60.94	36
1016.5388	2	1016.539	-0.0001	245	253	0	40.58	38
1113.6756	2	1113.6758	-0.0001	331	340	0	78.84	32
1141.565	2	1141.5663	-0.0013	138	148	0	58.18	35
1172.6404	2	1172.6401	0.0004	244	253	1	42.99	38
1400.7246	2	1400.726	-0.0014	76	89	0	112.7	37
1501.871	2	1501.8729	-0.002	153	167	0	70.3	30
1570.7004	2	1570.7007	-0.0003	184	196	0	94.01	34

1826.9306	2	1826.9315	-0.0009	53	68	1	88.84	37
1919.9942	2	1919.988	0.0062	396	412	0	76.11	37
2026.8855	3	2026.8875	-0.002	300	317	1	82.78	33
2058.0814	2	2058.082	-0.0005	213	231	0	129.53	36
957.5131	2	957.5131	0.0001	118	125	0	51.34	36
1016.5388	2	1016.539	-0.0001	245	253	0	40.58	38
1113.6756	2	1113.6758	-0.0001	331	340	0	78.84	32
1141.565	2	1141.5663	-0.0013	138	148	0	58.18	35
1172.6404	2	1172.6401	0.0004	244	253	1	42.99	38
1400.7246	2	1400.726	-0.0014	76	89	0	112.7	37
1446.7557	2	1446.7467	0.009	376	388	0	72.48	38
1501.871	2	1501.8729	-0.002	153	167	0	70.3	30
1570.7004	2	1570.7007	-0.0003	184	196	0	94.01	34
1826.9306	2	1826.9315	-0.0009	53	68	1	88.84	37
1919.9942	2	1919.988	0.0062	396	412	0	76.11	37
2026.8855	3	2026.8875	-0.002	300	317	1	82.78	33
2058.0814	2	2058.082	-0.0005	213	231	0	129.53	36
1196.6872	2	1196.6877	-0.0005	164	175	0	78.2	33
1227.621	2	1227.6207	0.0003	29	39	0	61.25	36
1277.6219	2	1277.6227	-0.0008	243	252	0	45.69	38
1357.6075	2	1357.6084	-0.0009	546	556	0	70.43	34
1435.7452	2	1435.746	-0.0007	352	363	0	57.43	37
1472.6771	2	1472.6783	-0.0013	40	52	0	78.71	35
1662.802	2	1662.8036	-0.0015	60	74	0	102.92	37
1674.722	2	1674.7234	-0.0014	227	242	0	93.66	32
1679.827	2	1679.8267	0.0002	145	159	0	51.17	38
2573.2622	2	2573.2609	0.0014	368	393	0	155.4	37
2587.2103	3	2587.2126	-0.0022	5	28	0	39.36	36
1227.621	2	1227.6207	0.0003	29	39	0	61.25	36
1230.6396	2	1230.6391	0.0006	164	175	0	54.58	38
1259.675	2	1259.6761	-0.0011	133	144	0	66.96	37
1277.6219	2	1277.6227	-0.0008	243	252	0	45.69	38
1312.6117	2	1312.6122	-0.0004	308	317	0	73.34	36
1357.6075	2	1357.6084	-0.0009	546	556	0	70.43	34
1435.7452	2	1435.746	-0.0007	352	363	0	57.43	37
1472.6771	2	1472.6783	-0.0013	40	52	0	78.71	35
1539.7502	3	1539.7504	-0.0002	306	317	1	58.19	37
1674.722	2	1674.7234	-0.0014	227	242	0	93.66	32
1679.827	2	1679.8267	0.0002	145	159	0	51.17	38
2573.2622	2	2573.2609	0.0014	368	393	0	155.4	37
2587.2103	3	2587.2126	-0.0022	5	28	0	39.36	36
932.4594	2	932.4603	-0.0009	411	418	0	39.11	38
954.5647	2	954.5651	-0.0004	461	468	0	35.26	31
1090.5909	2	1090.591	-0.0001	496	504	0	48.89	36
1160.5726	2	1160.5727	-0.0001	290	299	0	60.58	37
1162.6338	2	1162.6346	-0.0008	395	403	1	58.88	36
1316.7156	2	1316.7162	-0.0006	219	230	0	59.17	37
1381.5788	2	1381.5795	-0.0007	354	364	0	80.73	31
1392.7394	2	1392.7402	-0.0007	427	439	0	42.62	36
1608.8714	2	1608.8722	-0.0009	310	324	0	68.66	34
1650.812	2	1650.8114	0.0006	169	183	0	73.84	37
1701.7299	2	1701.7304	-0.0006	258	273	0	118.57	31
1719.865	2	1719.8679	-0.0029	516	530	0	122.99	38
1970.0473	2	1970.0473	0	478	495	0	63.8	35
2196.1018	2	2196.1022	-0.0004	197	218	0	94.04	37
2656.3407	3	2656.3418	-0.0011	365	386	0	67.04	37
1058.5211	2	1058.5219	-0.0008	310	318	0	41	35
1138.6861	2	1138.6862	-0.0001	253	262	0	64.51	28

1145.5818	2	1145.5829	-0.0011	242	251	0	50.37	38
1214.5747	2	1214.5754	-0.0007	381	390	0	73.61	35
1383.6927	2	1383.6931	-0.0004	163	174	0	58.76	36
1430.6232	2	1430.6248	-0.0016	47	58	0	83.6	32
1445.6802	2	1445.682	-0.0018	325	336	0	83.14	36
1621.8443	2	1621.8439	0.0004	263	276	0	66.69	37
1695.8247	2	1695.8257	-0.0009	337	350	0	82.13	37
1955.9945	3	1955.9953	-0.0008	104	121	0	50	37
919.4795	2	919.4797	-0.0001	43	52	0	91.11	38
940.5011	2	940.5018	-0.0007	63	70	0	36.33	34
1438.6788	2	1438.6801	-0.0013	27	42	0	129.16	37
1500.7041	2	1500.7056	-0.0015	76	89	0	129.39	36
2155.0154	2	2155.0181	-0.0027	71	89	1	85.86	37
986.5398	2	986.5397	0.0001	327	336	0	69.57	37
1266.5012	2	1266.5018	-0.0006	312	320	0	52.43	26
1395.7494	2	1395.751	-0.0016	85	96	0	40	36
1530.8979	2	1530.8981	-0.0001	230	243	0	116.84	28
1583.7446	2	1583.7443	0.0003	340	352	0	76.12	36
1700.8972	2	1700.8985	-0.0013	65	79	0	76.13	37
1867.8904	2	1867.892	-0.0016	374	390	0	119.13	37
1884.9395	2	1884.9404	-0.0009	353	370	0	88.78	37
2329.0111	3	2329.011	0.0002	403	422	0	67.36	32
832.4552	2	832.4555	-0.0003	8	15	0	43.21	38
1497.8404	2	1497.8403	0.0001	239	252	0	104.51	34
1675.9662	3	1675.9661	0.0001	70	84	1	56.37	29
1733.8071	2	1733.8083	-0.0012	131	145	0	74.13	36
1760.779	2	1760.7794	-0.0004	314	327	0	78.23	33
2032.0638	2	2032.0663	-0.0025	172	190	0	73.5	37
2171.001	2	2171.0019	-0.0008	276	295	0	128.52	36
2249.0648	2	2249.0668	-0.0019	146	166	0	129.51	37
822.4962	2	822.4963	-0.0001	185	191	0	30.1	28
1094.6382	2	1094.6376	0.0006	382	390	0	43.14	31
1128.6383	2	1128.6391	-0.0007	465	476	0	62.35	35
1156.6436	2	1156.6452	-0.0016	316	326	0	80.95	36
1388.6639	2	1388.6646	-0.0007	330	341	0	74.5	36
1434.6614	2	1434.6626	-0.0012	113	124	1	74.05	36
1680.9416	2	1680.941	0.0006	342	357	0	97.79	32
1767.7782	3	1767.7787	-0.0005	143	157	0	70.35	33
1811.9647	2	1811.9638	0.001	441	456	0	50.85	35
1922.0396	2	1922.0394	0.0002	93	112	0	85.17	34
2113.0422	2	2113.0426	-0.0004	205	222	0	83.76	37
913.5592	2	913.5597	-0.0005	419	427	0	39.85	33
1006.5466	3	1006.5447	0.0019	165	172	1	42.35	37
1024.6021	2	1024.603	-0.0009	244	254	0	69.99	29
1025.481	2	1025.4818	-0.0008	205	212	0	44.77	35
1119.5914	2	1119.5924	-0.001	21	30	0	55.92	37
1209.5691	2	1209.5699	-0.0009	42	51	1	39.51	36
1297.7389	2	1297.7394	-0.0005	135	146	0	89.47	33
1450.6652	2	1450.6663	-0.0011	85	96	0	68.5	36
1633.8939	3	1633.894	-0.0002	6	20	0	62.54	34
2311.1178	3	2311.1228	-0.005	392	411	1	50.18	37
2505.3208	2	2505.3223	-0.0014	255	278	1	59.34	35
2956.4146	3	2956.4158	-0.0012	101	129	0	66.35	36
1155.5974	2	1155.5958	0.0016	81	90	0	36.18	36
1192.6556	2	1192.6564	-0.0008	138	147	0	79.12	35
1216.5983	2	1216.5989	-0.0006	321	330	0	54.09	37
1279.6217	2	1279.6231	-0.0014	207	217	0	81.52	37
1422.656	2	1422.6568	-0.0007	183	193	0	74.69	36

1482.8137	2	1482.8155	-0.0017	102	116	0	88.64	34
1601.8636	2	1601.8633	0.0003	306	320	0	58.02	36
1802.9265	2	1802.9262	0.0003	348	365	0	109.24	37
792.4376	2	792.4382	-0.0005	219	225	0	50.43	35
815.4864	2	815.4865	-0.0001	121	127	0	36.5	35
1221.6239	2	1221.6241	-0.0002	149	158	0	54.23	37
1478.7719	2	1478.7729	-0.001	147	158	1	79.86	37
1514.7082	2	1514.7101	-0.0019	354	368	0	125.94	36
1682.8298	2	1681.8311	0.9987	88	101	0	87.98	37
1812.9942	2	1812.9954	-0.0012	312	328	0	82	35
1845.8482	2	1845.8493	-0.0011	159	174	0	80.24	35
856.5133	2	856.513	0.0002	545	551	0	37.29	35
1129.5617	2	1129.5615	0.0002	613	622	0	52.38	36
1189.6691	2	1189.6707	-0.0015	398	408	0	59.31	34
1234.5669	2	1234.5677	-0.0008	552	563	0	64.48	36
1416.7192	2	1416.7209	-0.0016	112	125	0	66.65	37
1416.7277	2	1416.7283	-0.0006	382	394	0	108.08	38
1549.7889	2	1549.7889	0	193	206	0	41.65	38
1612.8665	3	1612.8685	-0.002	352	364	1	68.69	35
1820.8984	2	1820.9017	-0.0033	472	488	0	118.62	38
904.4507	2	904.4502	0.0006	291	298	0	48.65	38
1247.6762	2	1247.6761	0	393	403	0	69.08	36
1248.5573	2	1248.5582	-0.0009	547	558	0	65.35	34
1293.5736	2	1293.5758	-0.0022	618	629	0	56.43	34
1326.6304	2	1326.6303	0.0001	581	592	0	67.25	36
1360.7331	2	1360.7351	-0.002	160	172	0	49.57	37
1416.7277	2	1416.7283	-0.0006	377	389	0	108.08	38
1549.7889	2	1549.7889	0	188	201	0	41.65	38
1812.9464	2	1812.9469	-0.0004	601	617	0	106.97	36
815.4864	2	815.4865	-0.0001	121	127	0	36.5	35
1221.6239	2	1221.6241	-0.0002	149	158	0	54.23	37
1478.7719	2	1478.7729	-0.001	147	158	1	79.86	37
1514.7082	2	1514.7101	-0.0019	354	368	0	125.94	36
1725.768	2	1725.7702	-0.0022	219	233	0	62.33	34
1764.9052	2	1764.9046	0.0006	88	102	0	79	37
1799.9294	2	1799.9305	-0.0011	46	63	0	57.61	37
847.4911	2	847.4916	-0.0005	445	452	0	50.14	34
1268.7087	2	1268.7088	-0.0001	512	524	0	81.16	34
1275.642	2	1275.6427	-0.0008	230	240	0	72.87	38
1467.7759	2	1467.7755	0.0003	599	611	0	65.89	36
1482.754	2	1482.7541	-0.0001	389	400	0	80.53	37
1567.8237	2	1567.8246	-0.0008	273	285	0	55.81	36
2240.226	2	2240.2264	-0.0003	49	70	0	98.59	32
1151.7063	2	1151.7067	-0.0004	157	167	0	42.08	24
1696.752	2	1696.754	-0.002	73	87	0	95.62	34
2149.1236	2	2149.1242	-0.0005	278	296	0	121.01	36
2346.1806	2	2346.1815	-0.0009	410	432	0	142.16	37
1138.5739	2	1138.5731	0.0009	973	981	0	79.64	37
1314.6897	2	1314.6892	0.0006	1259	1271	0	52.33	37
1491.7353	2	1491.7358	-0.0005	1044	1055	0	43.03	38
1872.8374	2	1872.8391	-0.0017	582	597	0	107.92	34
1954.9642	3	1954.965	-0.0008	982	997	0	66.41	37
1985.9358	2	1985.9371	-0.0013	614	630	0	64.02	36
2167.9805	3	2166.9777	1.0028	535	553	0	72.11	35
975.441	2	975.441	0	21	30	0	59.64	33
1143.5517	2	1143.5529	-0.0012	42	52	0	37.14	35
1197.6981	2	1197.6982	-0.0001	31	41	1	55.5	33
1474.6676	2	1474.6688	-0.0012	362	374	0	77.45	34

1514.7414	2	1514.7419	-0.0005	87	97	0	38.17	37
1854.9251	2	1854.9251	0	218	233	0	119.22	37
2182.0702	2	2182.0729	-0.0026	294	314	0	41.39	38
3150.6331	3	3150.635	-0.0019	150	179	0	79.14	34
1241.6426	2	1241.6438	-0.0012	80	90	0	40.07	35
1934.0261	2	1934.0262	-0.0001	180	197	0	115.28	36
2430.2296	2	2430.2319	-0.0022	249	271	0	111.72	37
1212.6715	2	1212.6714	0.0001	296	306	0	106.31	35
1587.7839	2	1587.7852	-0.0014	228	243	0	89.2	37
1767.7782	3	1767.7787	-0.0005	131	145	0	70.35	33
2128.0666	2	2128.0609	0.0057	193	210	0	53.78	37
2203.1673	3	2203.1711	-0.0038	263	281	1	44.55	35
1282.7489	2	1282.7496	-0.0007	115	127	0	36.56	32
1302.7431	2	1302.7435	-0.0004	136	146	0	60.31	33
1325.6068	2	1325.6074	-0.0007	270	280	0	65.6	36
1512.8302	3	1512.83	0.0002	204	216	0	71.82	35
1768.8436	2	1768.8454	-0.0018	147	161	0	58.06	37
2320.204	3	2320.2063	-0.0023	93	113	0	49.99	36
997.5556	2	997.5556	0	140	148	0	61.48	33
1192.6102	3	1192.6102	0	48	58	0	46.58	38
1402.7905	2	1402.7932	-0.0028	150	162	0	94.98	34
1630.822	2	1630.8236	-0.0015	171	185	0	104.64	38
1192.6556	2	1192.6564	-0.0008	138	147	0	79.12	35
1216.5983	2	1216.5989	-0.0006	321	330	0	54.09	37
1422.656	2	1422.6568	-0.0007	183	193	0	74.69	36
1468.7969	2	1468.7998	-0.0029	102	116	0	95.83	35
1699.9124	3	1699.9113	0.0011	305	320	0	44.36	36
2260.0706	2	2260.0707	0	346	367	0	55.86	37
1451.6823	2	1451.6834	-0.0011	244	255	0	49.68	37
1501.6708	2	1501.6718	-0.0011	593	606	0	96.34	34
1798.9243	2	1798.9254	-0.0011	771	786	0	75.33	37
2398.1547	3	2398.1553	-0.0005	349	368	1	60.27	37
1286.7007	2	1286.7016	-0.0009	400	411	0	50.78	37
1750.8564	2	1750.856	0.0004	311	325	0	93.65	38
2144.0986	2	2144.1001	-0.0014	182	201	0	103.15	37
2203.1258	2	2203.126	-0.0002	276	296	0	44.89	37
971.5393	2	971.54	-0.0007	566	574	0	40.77	37
1028.5616	2	1028.5614	0.0001	330	339	0	45.69	36
1049.5252	2	1049.5254	-0.0001	521	529	0	62.5	37
1158.5859	2	1158.588	-0.0021	506	516	0	83.12	37
1396.7306	2	1396.731	-0.0004	236	248	0	62.9	36
1475.7747	2	1475.7773	-0.0025	183	196	0	47.57	38
1891.042	2	1891.0415	0.0005	454	470	0	69.13	33
2507.3748	3	2507.3748	0	136	160	0	32.83	31
1038.6072	2	1038.6073	-0.0001	64	72	0	49.74	29
1247.6256	2	1247.6258	-0.0003	73	83	0	47.17	39
1322.6567	2	1322.6605	-0.0038	429	439	0	68.98	38
1435.6326	2	1435.6323	0.0003	113	124	0	56.4	33
2651.3926	3	2651.3919	0.0007	319	341	0	67.52	34
2891.4818	3	2890.4859	0.9959	218	244	0	50.03	36
1292.6709	2	1292.6725	-0.0016	92	102	0	50.25	38
1368.6243	2	1368.6245	-0.0002	225	235	0	57	35
1668.8215	2	1668.822	-0.0005	181	196	0	92.88	37
2468.1708	2	2468.1707	0.0001	453	475	0	76.38	37
2492.3531	3	2491.3435	1.0096	158	180	0	37.4	32
1172.5854	2	1172.586	-0.0006	512	521	0	65.98	36
1397.6717	2	1397.6715	0.0003	206	217	0	66.51	36
2271.1586	2	2271.1595	-0.0008	85	106	0	111.14	37

2521.3735	3	2521.3751	-0.0016	437	461	0	36.27	31
939.5755	2	939.5753	0.0002	125	132	0	54.43	28
1864.0419	2	1864.0418	0.0002	68	84	0	102.1	31
2080.9247	3	2080.9273	-0.0026	107	124	0	48.62	34
2176.0288	2	2176.0293	-0.0004	140	159	0	58.17	37
1154.6403	2	1154.6408	-0.0004	192	203	0	57.38	33
1295.6138	2	1295.6146	-0.0007	333	342	0	43.68	37
1493.7148	2	1493.7151	-0.0002	68	80	0	70.4	37
1563.8193	2	1563.8218	-0.0025	145	158	0	42.26	37
2052.003	2	2052.0052	-0.0021	313	330	0	92.62	38
770.4399	2	770.4399	0	346	353	0	60.61	32
916.5742	2	916.5746	-0.0003	367	374	0	55.75	26
1065.4877	2	1065.488	-0.0003	137	145	0	37.41	36
1359.7048	2	1359.7068	-0.0019	106	116	0	52.26	38
1407.7145	3	1407.7147	-0.0002	134	145	1	41.95	38
1445.7354	2	1445.7343	0.0011	154	165	0	49.21	38
2047.0094	2	2047.0085	0.001	76	95	0	63.53	37
975.441	2	975.441	0	21	30	0	59.64	33
1131.519	2	1131.5197	-0.0007	199	208	0	41.19	34
1143.5517	2	1143.5529	-0.0012	42	52	0	37.14	35
1197.6981	2	1197.6982	-0.0001	31	41	1	55.5	33
1514.7414	2	1514.7419	-0.0005	87	97	0	38.17	37
1789.8832	2	1789.8846	-0.0014	241	256	0	54.65	38
3150.6331	3	3150.635	-0.0019	150	179	0	79.14	34
770.4399	2	770.4399	0	350	357	0	60.61	32
916.5742	2	916.5746	-0.0003	371	378	0	55.75	26
1031.5038	2	1031.5036	0.0002	141	149	0	45.48	37
1227.6128	2	1227.6136	-0.0008	330	340	0	59.59	36
1359.7048	2	1359.7068	-0.0019	110	120	0	52.26	38
1401.736	3	1401.7365	-0.0004	138	149	1	47.93	38
1445.7354	2	1445.7343	0.0011	158	169	0	49.21	38
1130.4627	2	1130.4629	-0.0001	584	594	0	64.38	28
1158.494	2	1158.4942	-0.0002	602	613	0	34.49	30
1186.6956	2	1186.6962	-0.0006	155	165	0	46.11	33
1260.6718	2	1260.6714	0.0005	471	481	0	40.88	38
1499.6771	3	1499.6787	-0.0016	375	387	0	42.61	35
1814.903	2	1814.905	-0.0021	319	335	0	82.11	37
709.3873	2	709.3871	0.0002	171	176	0	34.68	32
1277.6175	2	1277.6187	-0.0012	348	358	0	76.27	38
1900.9364	2	1900.9386	-0.0022	268	285	0	84.32	37
3356.575	3	3356.5772	-0.0022	193	222	0	55.82	35
1154.6403	2	1154.6408	-0.0004	192	203	0	57.38	33
1295.6138	2	1295.6146	-0.0007	333	342	0	43.68	37
1493.7148	2	1493.7151	-0.0002	68	80	0	70.4	37
1563.8193	2	1563.8218	-0.0025	145	158	0	42.26	37
2018.0204	2	2018.0208	-0.0004	313	330	0	84.49	38
1800.7848	2	1800.7861	-0.0013	62	80	0	104.76	32
2851.4942	3	2851.4967	-0.0025	227	253	0	59.06	34
2955.4516	3	2955.4502	0.0014	254	281	0	52.73	36
974.5549	2	974.5549	0	227	237	0	37.6	36
1424.7668	2	1424.7664	0.0004	309	321	0	64.27	35
1456.8323	2	1456.8323	0	238	250	0	64.45	33
1491.7677	2	1491.7681	-0.0005	338	351	0	87.82	38
1507.7599	2	1507.7592	0.0007	212	224	0	49.15	38
1945.051	2	1945.052	-0.001	479	498	0	137.76	35
978.4657	2	978.4672	-0.0015	365	373	0	53.25	36
1205.5991	2	1205.604	-0.0049	61	70	0	80.25	38
1469.7251	2	1469.7258	-0.0008	48	60	0	85.57	37

1157.6431	2	1157.6445	-0.0014	175	186	0	41.55	37
1272.6326	2	1272.635	-0.0024	329	339	0	73.99	37
1275.6831	2	1275.6823	0.0009	192	203	0	47.5	37
1935.962	2	1935.9612	0.0008	262	278	0	50.49	38
3243.5488	3	3243.5506	-0.0019	120	149	0	65.63	36
1354.6538	2	1353.6598	0.994	95	106	0	72.78	37
2084.0582	2	2084.0572	0.0011	56	75	0	106.5	37
1686.8792	2	1686.8788	0.0003	749	764	0	112.41	37
1836.8992	2	1836.9006	-0.0014	912	928	0	68.9	38
869.5333	2	869.5334	-0.0001	236	243	0	50.11	30
914.4807	2	914.4821	-0.0014	389	396	0	36.23	34
1009.5914	2	1009.592	-0.0007	417	425	0	38.24	29
1460.7624	2	1460.7623	0.0001	185	197	0	93.65	38
1578.802	2	1578.8042	-0.0022	133	146	0	62.44	38
1157.6431	2	1157.6445	-0.0014	206	217	0	41.55	37
1643.8696	2	1643.8705	-0.0009	235	250	0	41.94	37
1885.8947	2	1885.8945	0.0002	140	155	0	46.37	37
1966.9297	2	1966.9306	-0.0009	293	309	0	116.69	37
1255.6878	2	1255.6884	-0.0006	88	99	0	53.12	35
1290.6274	2	1290.6303	-0.0029	34	45	0	50.43	37
1526.7932	2	1526.794	-0.0008	358	371	0	88	36
1582.7834	3	1582.7839	-0.0005	441	454	1	46.11	37
1287.6606	2	1287.6605	0.0001	95	106	0	78.6	37
1422.6607	2	1422.6627	-0.002	28	40	0	79.25	36
1524.8998	2	1524.8988	0.001	1069	1083	0	71.78	29
1940.0522	2	1940.052	0.0002	810	827	0	92.26	34
999.5696	2	999.5713	-0.0017	268	276	0	47.95	36
1284.7108	2	1284.7112	-0.0004	177	188	0	50.57	36
1414.7559	2	1414.7569	-0.001	254	267	0	103.97	37
897.4917	2	897.492	-0.0003	24	31	0	41.73	33
1169.597	2	1169.5968	0.0002	178	187	0	54.26	35
1581.8471	2	1581.8475	-0.0004	75	89	0	68.03	35
1791.8936	3	1791.8944	-0.0009	97	113	0	53.92	37
1498.8285	2	1498.8296	-0.0011	2051	2062	0	50.34	35
1557.8877	2	1557.8878	-0.0001	1244	1257	0	44.71	32
1578.697	2	1578.6984	-0.0014	1940	1952	0	55.48	34
1713.8263	2	1713.8284	-0.002	1714	1728	0	56.62	37
1256.733	2	1256.734	-0.001	84	95	0	92.22	33
1383.7031	2	1383.7034	-0.0003	26	37	0	66.3	37
1018.5026	2	1018.5043	-0.0018	805	815	0	97.03	39
1461.7613	2	1461.7616	-0.0003	683	694	0	56.94	38
1835.9328	2	1835.9339	-0.001	112	127	0	44.54	38
1157.6431	2	1157.6445	-0.0014	197	208	0	41.55	37
1324.6278	2	1324.6268	0.001	374	385	0	39.61	37
1478.8545	3	1478.8569	-0.0024	311	323	1	46.83	30
1921.9453	2	1921.9455	-0.0002	285	301	0	81.47	38
1446.8108	2	1446.8082	0.0026	330	343	0	68.7	34
1889.9837	2	1889.9847	-0.0009	440	458	0	75.97	37
1255.6449	2	1255.6448	0	589	598	0	55.72	35
1664.8413	2	1664.841	0.0004	677	692	0	89.31	37
1159.6199	2	1159.6197	0.0002	50	61	0	84.95	38
1165.5953	2	1165.5979	-0.0026	66	76	0	60.74	37
1403.6519	2	1403.6528	-0.0009	441	453	0	88.69	35
2222.173	2	2222.1736	-0.0005	96	115	0	45.44	35
1844.999	2	1844.9996	-0.0005	246	261	0	81.25	35
1945.948	2	1945.9495	-0.0015	165	181	0	53.2	37
1547.8818	2	1547.881	0.0007	139	152	0	59.84	31
1960.9691	3	1960.9676	0.0015	318	333	0	51.09	37

2095.1662	3	2095.1677	-0.0015	341	359	0	52.84	31
1729.8326	2	1729.8271	0.0055	517	532	0	64.95	37
2642.3441	3	2641.3347	1.0093	131	154	0	68.28	37
1053.5543	2	1053.5567	-0.0024	106	116	0	70.39	35
1442.7236	2	1442.7253	-0.0016	128	140	0	67.6	37
1180.6919	2	1180.6928	-0.0009	129	139	0	52.44	30
1384.7918	2	1384.7926	-0.0008	78	90	0	78.74	34
1402.8589	2	1402.8588	0.0001	562	574	0	46.59	20
1564.9006	2	1564.9011	-0.0005	671	685	0	52.6	29
2111.042	2	2111.0436	-0.0016	368	387	0	38.63	37
1198.6558	2	1198.6557	0	286	296	0	62.23	35
1279.7601	2	1279.7612	-0.0011	161	173	0	62.9	28
1052.5379	2	1051.5298	1.0081	232	241	0	40.03	35
1434.735	2	1434.7354	-0.0005	1132	1144	0	82.33	38
1279.7865	2	1279.7864	0.0001	802	814	0	47.89	24
2336.154	2	2336.1536	0.0005	1401	1421	0	67.82	38
1212.5551	2	1212.5557	-0.0006	153	162	0	58.9	35
1618.8822	2	1618.8831	-0.0009	200	213	0	64.77	35
754.5059	2	754.5065	-0.0006	102	107	0	41.8	20
773.5164	2	773.5163	0	83	88	0	43.1	22
1505.7668	2	1505.7667	0.0001	197	208	0	53.34	38
1188.6333	2	1188.635	-0.0017	182	194	0	62.53	38
1351.6346	2	1351.6368	-0.0021	331	342	0	57.53	37
1248.6108	2	1248.6132	-0.0024	135	146	0	76.4	38
2443.3869	3	2443.3872	-0.0004	7	29	0	36.83	27
1247.5814	2	1247.5816	-0.0001	579	589	0	39.55	36
1438.702	2	1438.7013	0.0007	709	720	0	60.91	37
2488.3155	3	2488.3173	-0.0018	385	407	0	48.27	35
1361.7376	2	1361.7377	-0.0001	1194	1205	0	47.74	36
2048.0986	2	2048.0983	0.0004	957	974	0	62.99	35
1087.5708	2	1087.5696	0.0012	377	386	0	38.48	38
1241.6287	2	1241.6292	-0.0005	412	422	0	70.88	36
1145.604	2	1145.604	0	50	61	0	49.82	39
1165.5953	2	1165.5979	-0.0026	66	76	0	60.74	37
956.5539	2	956.5542	-0.0003	126	133	0	39.14	34
1044.4693	2	1044.4699	-0.0006	208	216	0	44.43	31
1144.5899	2	1144.591	-0.0011	134	142	0	43.68	37
1042.5565	2	1042.556	0.0006	669	677	0	42.42	36
1362.7907	2	1362.7911	-0.0004	68	79	0	53.44	30
1312.6366	2	1312.6299	0.0067	78	89	0	38.87	36
1464.7736	2	1464.7725	0.0011	95	106	0	52.94	37
1157.6431	2	1157.6445	-0.0014	191	202	0	41.55	37
2028.0518	2	2028.0527	-0.0009	246	264	0	52.84	37
1170.5302	2	1170.5305	-0.0003	540	549	0	34.32	34
1713.7961	2	1713.7973	-0.0012	480	493	0	42.75	36
1267.6196	2	1267.6197	-0.0001	135	145	0	38.88	37
1467.8601	3	1467.8595	0.0006	38	49	1	38.84	29

low.

; **pep_rank**: rank of the peptide match, 1
articular query listed under the highest
ır: experimental relative molecular mass;
ıreshold; **pep_expect:** expectation value
ɔd_pos: position of variable modifications

pep_expect	pep_res_befc	pep_seq	pep_res_afte	pep_var_moc	pep_var_mod_pos	
2.20E-05	R	YLTASAMFR	G			
6.60E-06	K	LAVNLIPFPR	L			
5.00E-05	R	FPGQLNSDLR	K			
2.50E-06	R	VSEQFTAMFR	R	Oxidation (M)	0.0000000200.0	
0.0015	K	STVCDIPPTGL	M			
5.30E-08	R	YTGDSDLQLEI	I			
5.30E-07	R	MMLTFSVFPS	V			
1.20E-09	R	INVYYNEASCC	F			
3.30E-06	K	EVDEQMLNV(	N			
2.60E-08	R	LHFFMVGFAP	G	Oxidation (M)	0.00002000000000.0	
9.20E-07	R	AVLMDLEPGT	S	2 Oxidation (N	0.000200000020000.0	
4.10E-06	K	NSSYFVEWIPI	S			
3.20E-06	R	SLTVPELTQQI	N	Oxidation (M)	0.000000000020000.0	
8.20E-11	R	EILHIQGGQCC	F			
2.80E-09	K	FWEVVCAEHC	Y			
1.00E-13	K	MASTFIGNSTS	R	2 Oxidation (N	0.20000000000000200.0	
3.80E-13	K	GHYTEGAELID	K			
1.50E-05	K	GHYTEGAELID	E			
0.0019	-	MREILHIQGG(	F	Oxidation (M)	0.2000000000000000000.0	
1.90E-05	K	LTTPSFGDLNI	F	Oxidation (M)	0.0000000000000000200000000.0	
7.80E-08	R	SGPYGQTFRPI	G			
0.00015	K	EAENCDCLQG	I	Oxidation (M)	0.00000000000000000000000020000000.	emPAI
9.80E-07	K	SSVCDIAPK	G			
4.10E-07	R	YLTASAVFR	G			
6.60E-06	K	LAVNLIPFPR	L			
5.00E-05	R	FPGQLNSDLR	K			
2.50E-06	R	VSEQFTAMFR	R	Oxidation (M)	0.0000000200.0	
3.00E-08	R	IDVYFNEASG(	Y			
1.70E-07	R	MMMTFSVFP	V	Oxidation (M)	0.020000000000.0	
3.70E-07	K	EVDEQMMNI	N	Oxidation (M)	0.000000200000.0	
2.60E-08	R	LHFFMVGFAP	G	Oxidation (M)	0.00002000000000.0	
9.20E-07	R	AVLMDLEPGT	S	2 Oxidation (N	0.000200000020000.0	
4.10E-06	K	NSSYFVEWIPI	S			
8.20E-11	R	EILHIQGGQCC	F			
1.00E-13	K	MASTFIGNSTS	R	2 Oxidation (N	0.20000000000000200.0	
3.80E-13	K	GHYTEGAELID	K			
1.50E-05	K	GHYTEGAELID	E			
0.0019	-	MREILHIQGG(	F	Oxidation (M)	0.2000000000000000000.0	
0.0007	R	GSQQYSALSVI	N			
3.60E-07	K	LANPTFGDLNI	F	Oxidation (M)	0.0000000000000000200000000.0	
9.80E-06	R	SGPFGQIFRPD	G			
1.50E-08	K	FWEVICDEHG	I			emPAI
2.20E-05	R	YLTASAMFR	G			
6.60E-06	K	LAVNLIPFPR	L			
5.00E-05	R	FPGQLNSDLR	K			
2.50E-06	R	VSEQFTAMFR	R	Oxidation (M)	0.0000000200.0	
0.0015	K	STVCDIPPTGL	M			
5.30E-07	R	MMLTFSVFPS	V			

2.60E-08	R	VNVYYNEASC(	F			
3.30E-06	K	EVDEQMINVC	N			
2.60E-08	R	LHFFMVGFAP	G	Oxidation (M)	0.00002000000000.0	
3.00E-06	R	AVLMDLEPGT	S	2 Oxidation (M	0.000200000020000.0	
4.10E-06	K	NSSYFVEWIPN	S			
8.20E-11	R	EILHIQGGQCC	F			
1.00E-13	K	MASTFIGNSTS	R	2 Oxidation (M	0.20000000000000200.0	
3.80E-13	K	GHYTEGAELID	K			
1.50E-05	K	GHYTEGAELID	E			
0.0019	-	MREILHIQGG(	F	Oxidation (M)	0.2000000000000000000.0	
1.90E-05	K	LTTPSFGDLNH	F	Oxidation (M)	0.000000000000000200000000.0	
1.00E-07	R	SGPYGQIFRPD	G			
0.00015	K	EAENCDCLQG	I	Oxidation (M)	0.00000000000000000000000020000000.	emPAI
2.20E-05	R	YLTASAMFR	G			
6.60E-06	K	LAVNLIPFPR	L			
5.00E-05	R	FPGQLNSDLR	K			
2.50E-06	R	VSEQFTAMFR	R	Oxidation (M)	0.0000000200.0	
0.0015	K	STVCDIPPTGL	M			
0.00044	R	YVGDSELQLEF	V			
1.70E-07	R	MMMTFSVFP	V	Oxidation (M)	0.020000000000.0	
2.60E-08	R	VNVYYNEASC(	Y			
3.30E-06	K	EVDEQMLNV(	N			
2.60E-08	R	LHFFMVGFAP	G	Oxidation (M)	0.00002000000000.0	
3.00E-06	R	AVLMDLEPGT	S	2 Oxidation (M	0.000200000020000.0	
4.10E-06	K	NSSYFVEWIPN	S			
7.30E-11	K	FWEVVNLEHG	Y			
1.00E-13	K	MASTFIGNSTS	R	2 Oxidation (M	0.20000000000000200.0	
3.80E-13	K	GHYTEGAELID	K			
1.50E-05	K	GHYTEGAELID	E			
0.0049	K	LSTPSFGDLNH	F			
1.00E-07	R	SGPYGQIFRPD	G			
0.00015	K	EAENCDCLQG	I	Oxidation (M)	0.00000000000000000000000020000000.	emPAI
0.0022	R	TKDNNLLGK	F			
4.90E-07	K	DAGVIAGLNV	I			
0.00086	R	VEIIANDQGNI	T			
0.0017	R	MVNHFVQEFI	R	Oxidation (M)	0.2000000000.0	
1.70E-06	R	FEELNMDLFR	K	Oxidation (M)	0.0000020000.0	
5.00E-06	K	NALENYAYNM	N			
9.50E-05	K	ELESICNPIIAK	M			
5.20E-06	K	STVHDVVLVG	I			
0.00012	K	VQQLLQDFFN	E			
6.00E-06	R	TTPSYVAFTDS	L			
3.50E-05	R	ARFEELNMDL	K			
4.90E-08	K	EFAAEEISSMV	M			
1.10E-09	K	NQVAMNPVN	R	Oxidation (M)	0.000020000000000.0	
7.10E-09	K	ATAGDTHLGG	M			
0.0032	K	NAVVTVPAYF	Q			
9.50E-13	K	SINPDEAVAY(	V			
7.90E-05	K	GEGPAIGIDLG	V			
2.60E-09	K	EQVFSTYSDN(	A			
2.60E-08	K	MYQGAGGEA	I	2 Oxidation (M	0.2000000000000000200000000000000.0	
0.00021	R	TLSSTAQTTIEI	A			
0.0077	K	VQDLLLLDVTF	N	Oxidation (M)	0.000000000000000000000002000000.0	emPAI
0.0033	R	ALGDYQGVK	V			
0.00055	R	ELAQQIEK	V			
0.0069	R	DELTLEGIK	Q			
1.90E-07	R	VLITTDLLAR	G			
2.50E-05	K	VHACVGGTSV	E			

0.00025	K	RDELTLEGIK	Q			
5.40E-10	K	GLDVIQQAQS	T			
8.00E-08	K	GVAINFVTLDI	M			
1.60E-09	R	ILQAGVHVVV	V			
1.40E-09	R	KGVAINFVTLD	M			
4.70E-09	K	MFVLDEADEN	G	2 Oxidation (N	0.2000000002000.0	
0.0015	R	DHTVSATHGD	D			
1.50E-09	R	GIYAYGFEKPS	G			
8.20E-06	K	FYNVVVEELPS	-			
1.10E-06	R	SRDHTVSATH	D			
4.30E-10	K	IQVGVFSATM	K			
7.50E-10	R	GIDVQQVSLVI	I			
5.70E-05	K	TATFCSGVLQ(	E			emPAI
0.008	K	FDLMYAK	R	Oxidation (M)	0.0002000.0	
5.90E-06	K	DVNAAVGTIK	T			
0.0015	K	YMACCLMYR	G	Oxidation (M)	0.000000200.0	
5.70E-09	R	LVSQVISSLTAS	F			
1.10E-05	R	TIQFVDWCPT	C			
2.20E-06	R	SLNIERPTYTNI	L			
4.90E-08	R	AVFVDLEPTVI	T			
8.30E-08	R	IHFMLSSYAPV	A			
2.00E-09	R	AVCMISNSTS\	I			
7.00E-06	K	CGINYQPPTV\	V			
6.90E-12	K	TVGGGDDAFN	H			
0.00012	R	AFVHWYVGE(	E	Oxidation (M)	0.00000000002000000000.0	
6.80E-07	R	QLFHPEQLISG	G			
9.70E-08	R	FDGALNVDVT	I			
6.30E-06	K	AFHEQLSVAEI	C	2 Oxidation (N	0.00000000000000000002200.0	
3.10E-07	K	LADNCTGLQG	L			emPAI
0.008	K	FDLMYAK	R	Oxidation (M)	0.0002000.0	
5.90E-06	K	DVNAAVGTIK	T			
0.0015	K	YMACCLMYR	G	Oxidation (M)	0.000000200.0	
5.70E-09	R	LVSQVISSLTAS	F			
1.10E-05	R	TIQFVDWCPT	C			
0.0012	R	SLSIERPTYTNI	L			
4.90E-08	R	AVFVDLEPTVI	T			
8.30E-08	R	IHFMLSSYAPV	A			
2.00E-09	R	AVCMISNSTS\	I			
7.00E-06	K	CGINYQPPTV\	V			
6.90E-12	K	TVGGGDDAFN	H			
0.00012	R	AFVHWYVGE(	E	Oxidation (M)	0.00000000002000000000.0	
6.80E-07	R	QLFHPEQLISG	G			
9.70E-08	R	FDGALNVDVT	I			
6.30E-06	K	AFHEQLSVAEI	C	2 Oxidation (N	0.00000000000000000002200.0	
3.10E-07	K	LADNCTGLQG	L			emPAI
0.00011	R	ALGDYLGVK	V			
0.00055	R	ELAQQIEK	V			
1.50E-05	K	GVAINFVTR	D			
0.0069	R	DELTLEGIK	Q			
0.00044	R	KGVAINFVTR	D			
1.90E-07	R	VLITTDLLAR	G			
2.50E-05	K	VHACVGGTSV	E			
0.00025	K	RDELTLEGIK	Q			
5.40E-10	K	GLDVIQQAQS	T			
1.60E-09	R	ILQAGVHVVV	V			
4.70E-09	K	MFVLDEADEN	G	2 Oxidation (N	0.2000000002000.0	
0.0015	R	DHTVSATHGD	D			
1.50E-09	R	GIYAYGFEKPS	G			

8.20E-06	K	FYNVVVEELPS	-			
1.10E-06	R	SRDHTVSATH	D			
4.30E-10	K	IQVGVFSATM	K			
7.50E-10	R	GIDVQQVSLV	I			
0.0022	R	TKDNNLLGK	F			
4.90E-07	K	DAGVIAGLNV	I			
3.00E-05	R	FTDSSVQSDIK	L			
0.00086	R	VEIIANDQGN	T			
0.0017	R	MVNHFVQEF	R	Oxidation (M)	0.2000000000.0	
0.00016	R	FEELNIDLFR	K			
3.60E-06	R	EIAEAYLGTTI	N			
5.00E-06	K	NALENYAYN	N			
9.50E-05	K	ELESICNPIIAK	M			
2.00E-06	K	VQQLLVDFFN	E			
5.70E-10	K	NSIDDVVLVG	I			
6.00E-06	R	TTPSYVAFTDS	L			
0.0005	R	ARFEELNIDLF	K			
7.10E-09	K	ATAGDTHLGG	M			
2.70E-09	K	NQVAMNPIN	R	Oxidation (M)	0.000020000000000.0	
0.0032	K	NAVVTVPAYF	Q			
2.20E-10	K	SINPDEAVAY	V			
7.90E-05	K	GEGPAIGIDLG	V			
2.60E-09	K	EQVFSTYSDN	A			
7.60E-10	R	TLSSTAQTTIE	A			emPAI
0.0073	K	VDTAFIVK	H			
0.004	R	INAEDPFK	G			
0.0039	K	LIVWAPTR	E			
5.60E-05	K	LILDVEDFK	N			
6.00E-05	K	FGNVVHFGER	D			
0.00027	R	YKQEDIVLR	G			
0.0026	R	HIEFQVLADK	F			
9.70E-06	R	VANEIGFPVM	A	Oxidation (M)	0.000000000200.0	
6.50E-06	R	GSFYFMEMN	I			
0.0077	R	ITSYLPSGGPF	M			
1.70E-06	K	LLEEAPSPALT	K			
7.70E-05	R	DHGINFIGPN	V			
6.70E-11	K	SEAAAAFGND	F			
3.10E-10	K	HEEELAEPQE	D			
5.00E-06	R	ALNDTIITGVP	L			
9.00E-08	K	NAGVPTVPGS	V			
4.50E-08	R	IQVEHPVTEM	V			
7.70E-05	R	GCTMLHPGY	D	Oxidation (M)	0.00020000000000000000000.0	
4.10E-05	K	AMGDAAVAA	G	Oxidation (M)	0.0200000000000000000000000000.0	
5.50E-06	K	LADEAVCIGE	G			emPAI
0.0029	K	SSVCDIAPR	G			
2.20E-05	R	YLTASAMFR	G			
6.60E-06	K	LAVNLIPFPR	L			
5.00E-05	R	FPGQLNSDLR	K			
2.50E-06	R	VSEQFTAMFR	R	Oxidation (M)	0.0000000200.0	
5.10E-06	R	YVGNSDLQLE	V			
5.30E-07	R	MMLTFSVFPS	V			
2.60E-08	R	VNVYYNEASC	Y			
3.30E-06	K	EVDEQMINV	N			
2.60E-08	R	LHFFMVGFAP	G	Oxidation (M)	0.00002000000000.0	
4.10E-06	K	NSSYFVEWIP	S			
7.80E-05	K	GHYTEGAELI	K			
1.90E-05	K	LTTPSFGDLN	F	Oxidation (M)	0.0000000000000000200000000.0	
0.0073	R	TGPYGQIFRP	G			

0.00015	K	EAENCDCLQG	I	Oxidation (M)	0.00000000000000000000000020000000.	emPAI
0.0022	R	TKDNNLLGK	F			
5.10E-05	R	FSDASVQSDR	Q			
3.10E-07	K	DAGVIAGLNV	I			
0.00086	R	VEIIANDQGNI	T			
0.0017	R	MVNHFVQEFI	R	Oxidation (M)	0.2000000000.0	
5.00E-06	K	NALENYAYNM	N			
0.00012	K	VQQLLQDFFN	E			
6.00E-06	R	TTPSYVAFTDS	L			
1.10E-09	K	NQVAMNPVN	R	Oxidation (M)	0.000020000000000.0	
7.10E-09	K	ATAGDTHLGG	M			
0.0032	K	NAVVTVPAYF	Q			
2.20E-10	K	SINPDEAVAY(	V			
7.90E-05	K	GEGPAIGIDLG	V			
0.00056	K	KVEDSIEEAIQ'	M	3 Methyl (DE)	0.000000010000010000010000000.0	emPAI
0.0091	R	VFDLLR	R			
0.00011	R	ALGDYLGVK	A			
0.00055	R	ELAQQIEK	V			
0.0069	R	DELTLEGIK	Q			
1.90E-07	R	VLITTDLLAR	G			
0.00025	K	RDELTLEGIK	Q			
5.40E-10	K	GLDVIQQAQS	T			
4.70E-09	K	MFVLDEADEN	G	2 Oxidation (N	0.2000000002000.0	
0.0015	R	DHTVSATHGD	D			
1.50E-09	R	GIYAYGFEKPS	G			
8.20E-06	R	FYNVVVEELPS	-			
1.10E-06	R	SRDHTVSATH(	D			
7.50E-10	R	GIDVQQVSLVI	I			emPAI
0.008	K	FDLMYAK	R	Oxidation (M)	0.0002000.0	
5.90E-06	K	DVNAAVGTIK	T			
0.0015	K	YMACCLMYR	G	Oxidation (M)	0.000000200.0	
1.40E-11	R	LISQIISSLTTSL	F			
4.80E-07	R	TVQFVDWCPT	C			
9.50E-05	R	SLDIERPTYTNI	L			
4.90E-08	R	AVFVDLEPTVI	T			
0.0048	R	IHFMLSSYAPV	A	Oxidation (M)	0.00020000000000000.0	
1.30E-08	R	AVCMISNNTA	I	Oxidation (M)	0.00020000000000000.0	
7.00E-06	K	CGINYQPPTV\	V			
0.00012	R	AFVHWYVGE(	E	Oxidation (M)	0.0000000000200000000.0	
6.80E-07	R	QLFHPEQLISG	G			
7.20E-09	R	FDGAINVDITE	I			
0.00051	K	AYHEQLSVPEI	C	2 Oxidation (N	0.0000000000000000000002200.0	
3.10E-07	K	LADNCTGLQG	L			emPAI
3.80E-06	K	VQEIVSEIFGK	S			
1.80E-05	R	SSGGLSDDEIN	M			
0.00015	K	MKETAEAYLG	S	Oxidation (M)	0.20000000000.0	
0.00021	R	TAMAGEDVEI	A	Oxidation (M)	0.002000000000.0	
3.40E-06	R	NSADTTIYSVE	S			
0.00014	K	FSPSQIGANVL	M			
3.30E-08	K	EVDEVLLVGGI	V	Oxidation (M)	0.0000000000200.0	
1.60E-08	R	QAVTNPTNTII	R			
1.80E-13	K	IPAEIASEIETA'	T			
9.90E-07	R	IINEPTAAALSY	E	Oxidation (M)	0.00000000000002000.0	
5.60E-05	K	SQVFSTAADN	V	Oxidation (M)	0.00000000000200000.0	
0.00033	K	ATNGDTFLGG	S			emPAI
0.0018	K	AGIALSDK	F			
0.0076	K	YDSVHGQWK	H			
0.0015	R	VVDLIVHMSK	A			

5.50E-06	R	AASFNIIPSSTG	A			
1.60E-11	R	VPTVDVSVVD	L			
0.00015	K	TLLFGEKPVTV	N			
3.90E-08	K	LVSWYDNEW	V			
1.00E-05	R	FGIVEGLMTT\	T			
1.10E-12	K	GILGYTEDDV\	S			
1.20E-09	K	SDLDIVSNASC	V			emPAI
2.40E-05	K	VGAFSVLR	E			
0.0016	K	IDLDLEVR	L			
4.00E-05	K	CLAGLIVSR	S			
0.0062	K	HVVDDGLELR	K			
2.40E-06	K	ALSSPVLAAVG	Y			
2.90E-06	R	TNIQMIGALCI	A			
0.0088	K	QHLALLSLGEI	R			
0.00021	R	AELPSCLPVLV	M			
3.90E-07	K	MDVFNTFIDLI	Q	Oxidation (M)	0.200000000000.0	
0.00045	R	EYSLQALESFLI	C			
1.20E-10	K	TVTCIASLASSL	A			
1.20E-12	R	LSSIILQQLDD\	C			
0.00086	K	TDGLFLK	C			
0.0032	R	SLNLTLR	K			
3.40E-06	R	VAEYAFLYAK	T			
3.70E-06	K	TRYDDVDLITII	E			
1.70E-10	R	TADLGGSSTTT	A			
1.60E-05	R	TQSFLTWESLE	R			
1.50E-13	K	NLANPTALLLS	H	Oxidation (M)	0.00000000000000200.0	
2.90E-06	R	ENTEGEYSGLE	G			
0.0099	K	ELNLYANVRP(	T			emPAI
0.0057	K	RGILTLK	Y			
5.10E-07	K	AGFAGDDAPF	A			
0.009	K	EITALAPSSMK	I	Oxidation (M)	0.00000000020.0	
8.90E-06	R	GYMFTTTAER	E	Oxidation (M)	0.0020000000.0	
9.60E-06	R	AVFPSIVGRPR	H			
2.20E-07	K	SEYDESGPSIVI	K			
3.10E-09	K	NYELPDGQVIT	F			
3.30E-09	K	LAYVALDYEQI	S			
7.90E-09	K	DLYGNIVLSGG	M	Oxidation (M)	0.000000000000020000000.0	
1.70E-09	R	TTGIVLDSGDG	L			emPAI
0.0014	K	FEAIIYVLK	K			
2.80E-09	K	VGETVDLVGLI	E			
1.00E-05	R	SYTVTGVEMF	I			
9.20E-07	K	KYDEIDAAPEE	A			
2.40E-10	K	ILDEALAGDN\	G			
0.00023	R	HYAHVDCPGH	N			
1.90E-05	K	IVVELIVPVACI	F	Oxidation (M)	0.0000000000000020.0	
8.60E-06	K	TTLTAALTMAI	K			
2.20E-06	R	QTELPFLLAVE	G			
0.00041	R	VVQILAR	R			
0.0027	K	AEIANVLSR	G			
0.0005	K	TAIAEGLAQR	I			
0.0016	R	LDEMIVFR	Q	Oxidation (M)	0.00020000.0	
2.90E-05	R	HAQLPEEAR	E			
0.0055	K	EIELQVTER	F			
5.40E-05	R	VLELSLEEAR	Q			
4.30E-07	K	AIDLIDEAGSR	V			
6.90E-05	R	VLENLGADPSI	T			
0.0052	R	VVDEGFDPSY(	P			
3.30E-05	R	GSGFVAVEIPF	A			

1.70E-05	K	MPTLEEYGTN	L	Oxidation (M)	0.2000000000000.0	
6.60E-10	K	VPEPTVEEAIQ	E			
2.50E-08	R	YTDEALVAAA(	F			
1.90E-06	R	LGHNFVGTEQ	V			emPAI
8.30E-06	R	SGAVASAVMI	K			
0.005	K	ISWVPDPK	T			
2.70E-11	R	GYAATAAQGS	S			
2.70E-12	R	PETGSNEIDAA	A	Methyl (DE)	0.01000000000000.0	
2.10E-09	K	TGYYRPETGSI	A			
6.20E-05	R	QTVAVGVIK	S			
0.0026	K	ARYDEIIK	E			
3.60E-07	K	IGGIGTVPVGF	V			
0.0072	R	STNLDWYK	G			
0.0011	K	STTTGHLIYK	L			
1.20E-07	R	EHALLAFTLGV	Q			
1.90E-05	K	YYCTVIDAPGI	D			
0.00036	K	FHINIVVIGHV	S			
3.40E-05	K	MTPTKPMVVI	F	2 Oxidation (I	0.200000200000000000000.0	
3.30E-05	R	VETGMIKPGN	S	2 Oxidation (I	0.000020000020000000000000000.0	
1.00E-06	K	NMITGTSQAD	D	Oxidation (M)	0.020000000000000000000000000000.0	emPAI
0.00024	K	KPDVDIEK	I			
3.40E-05	R	ADIVVDVLVK	N			
1.10E-05	K	IPLTLIYDDIK	S			
1.70E-07	K	DFGSALWDM	G			
2.90E-05	K	GNIDVDTPFG,	L	Oxidation (M)	0.0000000000020.0	
2.30E-06	K	FSLEETVAILH\	L			
4.90E-07	K	STYNDINPGM	I			
3.90E-07	K	NPNPVPIPLID	K			
0.00093	K	STVVNTHLMP	S	Oxidation (M)	0.00000000200.0	
1.50E-10	K	AADQGLVILQ!	R			
1.50E-08	R	EDGEPVGYNE	K			
6.60E-12	K	SSEVEALISEITI	L			
3.90E-06	K	EGQVLCYIEQL	I			
5.20E-05	R	SGFGSFGMR	S	Oxidation (M)	0.000000020.0	
3.30E-07	R	SSGFGDFGSDI	S			
0.0029	R	SSFGGFGSND(	R			
0.00053	K	TLAFGIPIIDK	I			
0.0015	R	GSASMFEGIG!	S	Oxidation (M)	0.000020000000.0	
0.0078	R	FTELPSIAVER	G			
5.00E-06	K	CEALHGDISQS	E			
4.60E-09	R	QLDYGVDVAV	V			
0.0049	R	DGHFNILVATI	G			
5.60E-06	K	LADGITTYSIIA	A			
4.10E-05	R	ESAPSLDTICLY	Q	Oxidation (M)	0.0000000000000000000020.0	
0.0011	R	RVGDSESVGG	A			emPAI
0.0032	R	SLNLTLR	K			
3.40E-06	R	VAEYAFLYAK	T			
3.70E-06	K	TRYDDVDLITII	E			
1.70E-10	R	TADLGGSSTT1	A	Methyl (DE)	0.000000000001000.0	
3.10E-05	K	TDGLFLQCCDI	Y			
1.20E-06	K	ATLFPGDGIGF	Q			
0.0099	K	ELNLYANVRP(	T			emPAI
3.00E-05	K	GAGANILR	A			
5.30E-05	K	LQLIVFGK	K			
4.70E-05	R	AVAGAGVLSG	L			
1.50E-05	K	LLIQNQDEMII	A	Oxidation (M)	0.00000000200.0	
2.40E-06	R	YFPTQALNFAI	D			
3.20E-09	K	GFTNFALDFLI	T	Oxidation (M)	0.00000000002000000000.0	emPAI

0.00071	R	AGVSGYASVK	S			
1.20E-06	R	HNTLLEEQR	H			
0.0025	R	ELLEQLLSTK	M			
0.00047	R	NLLYQQQLR	Q			
1.30E-05	R	NNSLLSGIIDG(	S			
1.90E-06	R	TAASSYSGIEG	R			
2.50E-06	R	SFSVYNNPSA(	K			
0.0015	R	HIDPLWPSDH	S			
0.0005	R	SFLDFSTQDEE	A			
1.40E-06	K	TESAHAHISN(	L			
0.00054	R	SLEYQLLQQE(	H	Oxidation (M)	0.00000000000020000000.0	emPAI
0.0027	K	VQQITIR	S			
0.0026	K	TTEGIDLAK	D			
0.00019	K	EIEDAVADLR	S			
4.10E-05	K	VQSIVAEIFGK	S			
3.60E-06	R	SSGGLSEDDIC	M			
0.00015	K	MKETAEAYLG	S	Oxidation (M)	0.20000000000.0	
4.60E-05	R	QAVTNPTNTV	R			
3.30E-08	K	EVDEVLLVGGI	V	Oxidation (M)	0.0000000000200.0	
0.00018	R	SRFETLVNHLII	T			
1.10E-08	K	SQVFSTAADN	V			emPAI
3.60E-05	R	HENVDIVVIR	E			
0.00024	R	HLQFPSFADR	L			
9.80E-07	K	LADGLFLESCR	E			
2.80E-07	K	YAFEYAYLNNI	K			
4.20E-08	K	TPVGGGVSSLI	K			
2.00E-05	K	ANPVALLLSSA	H			
0.0016	K	ELDLFASLVNC	H			
0.00035	R	ENTEGEYAGLI	V			emPAI
0.00062	K	GVLLYGPPGT(	T			
6.10E-05	K	RFDSEVSGDR	E			
0.00021	R	STDDFNGAQL	A			
0.0024	R	ACAAQTNATF	L			
0.00067	K	DSYLILDTLPSE	V			
1.50E-13	R	TMLELLNQLD(	I	Methyl (DE); (	0.02000000000000010.0	emPAI
1.70E-08	R	GLAYDTSDDQ	G			
1.20E-11	R	VPIICTGNDFS	D			
9.60E-07	R	VQLAETYLSQ/	G			
1.90E-05	K	IKDEDIVTLVD(	A			emPAI
8.30E-05	R	IAGLEVLR	I			
0.0026	R	TPVENSLR	D			
0.0025	R	IPAVQDLVR	K			
8.10E-05	K	NQADSVVYQT	Q			
1.40E-09	K	QFAAEEISAQ\	K			
0.00017	R	QAVVNPENTF	R			
1.90E-09	R	IINEPTAASLAY	K			
0.0028	K	LSFKDIDEVILV	I			emPAI
0.0057	K	RGILTLK	Y			
5.10E-07	K	AGFAGDDAPF	A			
0.009	K	EITALAPSSMK	I	Oxidation (M)	0.00000000020.0	
9.60E-06	R	AVFPSIVGRPR	H			
3.80E-07	K	NYELPDGQVIT	F			
0.0015	R	VAPEEHPILLTI	A			
1.70E-09	R	TTGIVLDSGDC	L			emPAI
0.0027	R	TCNYLTSAAR	Y			
0.00054	R	MLQYGEQNIR	R	Oxidation (M)	0.2000000000.0	
3.20E-05	R	NLAGEIAQEYT	R			
5.00E-09	R	FLPLGLGLLYL(	Q			

4.30E-06	K	YIPLSPILEGFII	E			
4.90E-06	K	YLSDILSVLALT	E			
0.00057	R	QNLAATFVNA	L			emPAI
0.00013	K	YLSGMGIAR	Q	Oxidation (M)	0.000020000.0	
0.00024	K	LDLDSTFEQK	N			
0.00036	R	ALAESAQDAF	L			
7.60E-08	R	NQIQAYVFDV	A			
1.50E-07	R	NSVLAFSESVP	D			
5.70E-07	K	DVMDMVLVT	E	2 Oxidation (M	0.00202000000000000.0	emPAI
1.60E-08	R	TGGGGLDLSSI	R			
3.60E-05	R	TMAQLGLCAF	A	Oxidation (M)	0.02000000000.0	
9.90E-07	K	AFEVLNSLEVV	L			
5.40E-10	K	TLVMLEDFLNI	E	Oxidation (M)	0.00020000000000000.0	emPAI
0.00076	K	VVSAMVNND	S	Oxidation (M)	0.00002000000.0	
0.0011	R	TTFVPSTPPAL	N			
0.0026	K	IPGVISASSFD(	I			
9.90E-05	K	IGIYNIEGCSR	Y			
3.30E-09	K	VLDSGGLSPEL	D			
8.50E-08	K	DQAVDGLSSD	L			emPAI
1.60E-05	R	GLCAIAQAESL	Y			
2.70E-06	R	ELAEDGYSGVI	V			
6.80E-11	K	FVADGVFYAEI	E			
4.40E-09	K	VGEEVEILGLR	E			
3.50E-08	R	GSALSALQGTI	Q			
0.00023	R	HYAHVDCPGH	N			
8.00E-05	K	VDVVDDPELLI	E	Oxidation (M)	0.0000000000000002000.0	emPAI
3.60E-08	R	SPSVLVLLPTR	E			
3.40E-06	K	FATLVATNVA/	G			
2.20E-10	K	TLAFVLPILESL	S			
0.0049	K	NAGVNGSVVS	V			
7.00E-06	R	DLVGVLEDAIF	G			
7.50E-07	K	VVAAGANPVL	G			
8.60E-10	K	AAVEEGIVVG(	L			
3.00E-05	K	LSGGVAVIQV(	E			emPAI
3.60E-05	R	HENVDIVVIR	E			
0.00024	R	HLQFPSFADR	L			
2.80E-07	K	YAFEYAYLNNF	K			
1.50E-07	K	TPVGGGVSSLI	K			
0.00035	R	ENTEGEYAGLI	V			emPAI
8.90E-13	R	ALSDAADQAN	L			
5.90E-05	K	YCLDNFAAPLT	T			
9.50E-06	R	ETLVFLSQCFLI	R			emPAI
0.00077	R	QAVDISPLR	R			
0.00079	K	HATFVPHTAG	Y			
6.70E-09	R	VNQAIFLLTTG	E			
3.70E-10	K	TIAECLADELIN	G			emPAI
6.20E-06	R	LLILTDPR	T			
5.00E-12	R	VIVAIENPQDII	P			
0.0022	R	HTPGTFTNQM	L	Oxidation (M)	0.000000000200000000.0	
4.50E-05	K	EGALGNIPIIAF	F	Oxidation (M)	0.00000000000000000020.0	emPAI
0.0049	K	NAGVNGSVVS	V			
9.50E-05	R	DIISILEDAIK	G			
7.50E-07	K	VVAAGANPVL	G			
8.60E-10	K	AAVEEGIVVG(	L			
3.00E-05	K	LSGGVAVIQV(	E			emPAI
8.20E-05	R	LWGENFFDPA	K			
1.90E-09	R	PMEEGLAEAII	I	Oxidation (M)	0.02000000000000.0	
0.00011	K	AYLPVVESFGF	A			

8.30E-07	K	STLTDSLVAAA	M			emPAI
0.0029	R	SSFGGFGSND(	R			
0.00053	K	TLAFGIPIIDK	I			
0.003	R	FNELPSIAVER	G			
0.00069	K	CEALHGDISQ/	E			
0.00097	K	KGSAILIHGQD	A	Methyl (DE)	0.00000000001000.0	
1.60E-09	R	SSGFGSFGSG(	S			
4.10E-05	R	ESAPSLDTICLY	E	Oxidation (M)	0.0000000000000000000020.0	emPAI
4.70E-09	R	LSFLYLITGNLD	L			
5.70E-07	R	SLLDTNPTIES(	T			
0.0002	R	GPPALVFDFS(	L			
4.70E-06	R	VFLSILQTIPLV	R			emPAI
1.60E-05	K	AVANSTSATFL	V			
1.30E-11	R	TMLELLNQLD	G			
3.20E-05	K	SELLPCLGAAD	N			
0.0062	R	LLQFFQSPHAS	K			
6.50E-11	R	LLDFLEIASQQ	E			emPAI
3.00E-05	K	GAGANILR	A			
5.30E-05	K	LQLIVFGK	K			
0.00054	K	SDGIAGLYR	G			
9.70E-05	K	SSFDAFSQIVK	K			
1.50E-05	K	LLIQNQDEML	A	Oxidation (M)	0.00000000200.0	
2.40E-06	R	YFPTQALNFAF	D			emPAI
0.00014	K	VIEVEGPR	G			
0.00023	K	FLDGIYVSEK	S			
1.40E-05	R	TALSHVDNLIS	G			
0.0015	R	FVYAHFPINAS	S			
2.80E-09	K	TILSSETMDIPI	V	Oxidation (M)	0.00000002000000000.0	emPAI
0.0027	R	HGDLLR	T			
0.0053	K	SAGDPVLAR	I			
1.60E-06	R	AQEFVCGLAQ	I			
4.60E-08	R	LEVYTADDYAI	W			
8.30E-06	R	TVQHLIGSGM	A	Oxidation (M)	0.00000000020000000000000000000.0	emPAI
1.60E-06	R	VGLTGLTVAEY	D			
5.70E-10	R	FTQANSEVSAI	I			
5.10E-06	R	DAEGQDVLLF	F			emPAI
0.0089	K	AVDSLVPIGR	G			
4.80E-05	R	AAELTNLFESR	I			
5.80E-14	R	EVAAFAQFGSI	G			emPAI
2.20E-10	K	TSAADVLQLLT	M			
5.30E-09	K	QELLAELYSTEI	G			emPAI
0.0013	R	ALLIDLEPR	V			
0.0015	R	VINGIQNGDYI	N			
1.50E-05	R	DIIESLVDEYK	A			
0.0095	R	YPGYMNNDL\	C	Oxidation (M)	0.000020000000000000000.0	
3.50E-09	K	YISILNIIQGEVI	I			emPAI
1.30E-07	K	SLFESLNSVGV	R			
0.0015	K	LLAENLNCLLN	D			
2.70E-07	K	TATVAQILENV	D			
0.0091	K	MNYLDSFPFP'	D	Oxidation (M)	0.2000000000000000.0	
0.0097	R	VEHLVDEYNSI	S			emPAI
0.00016	K	SEVLDFIR	S			
5.10E-12	K	IADAEENLGES	E			
0.00047	R	SHGMAPLYET	A	2 Oxidation (N	0.0002000000000000000000000020.0	emPAI
0.001	R	ESAIAQIIR	T			
8.80E-06	R	LGNLGTISQN\	V			
0.0004	R	TCGPLLDVVAI	E			
0.002	K	VTFHNPGVQE	T			

9.50E-09	K	VPYGQGAVEL	E			emPAI
3.40E-09	R	LEGASEESLLS(	V			
1.10E-08	K	VFDDSVESFTS	G			emPAI
0.00062	K	GVLLYGPPGT(	T			
2.50E-06	K	LAEGFNGADL	N			
0.00048	R	AIASNIDANFL	V			
0.00043	R	NICTEAGMFA	A	Oxidation (M)	0.000000020000.0	
5.10E-07	K	SLQSVGQIIGE	L			emPAI
0.0031	R	FLALDEADR	M			
1.30E-05	R	VGSSTDLIVQR	V			
8.40E-06	R	TPILVATDVAA	G			
0.0033	R	MLDMGFEPQ	K	2 Oxidation (N	0.20020000000.0	
1.00E-06	R	LAADFLANYIF	V			emPAI
1.30E-05	K	GTVEIITPVELI	Q			
5.80E-05	R	IHSENTGNTAI	G			
2.40E-06	K	LCQLIDEYTQIL	K			
0.00055	R	VGLVAPIDVV\	I			emPAI
0.00086	R	SLDSHIEDQFA	L			
4.10E-05	R	LDTGNYSWGS	K			
2.90E-10	R	VLDVVYNASN	T			emPAI
1.90E-09	K	SVAAGSATVA	S			
5.10E-06	R	YGNATVVENT	G			
0.00043	K	QFSGVDILSGE	V			emPAI
3.10E-05	K	DLPAVMTFLIS	A			
0.0051	R	SQLLSFMDQLI	T			
1.50E-06	K	SGPLPVDTFTF	I			
0.00094	R	NPDTISQISDLI	T			
0.00043	K	VVIDVLSSIVSA	R			emPAI
0.008	K	IAELLR	Y			
4.60E-05	K	ADLVNNLGTI/	S			
0.00046	R	ELISNSSDALDI	I			
1.70E-07	K	GIVDSEDLPLN	E			
9.30E-05	K	SGDELTSLKDY	M			emPAI
0.00013	K	VLDFELVR	D			
0.0054	R	GINDTYFGR	L			
6.00E-06	K	SLLEEYFSIR	L			
1.90E-07	K	APNSFGEFLGE	V			emPAI
0.00063	R	VSYTVAR	A			
0.00032	R	VLAHTQIR	K			
0.0023	R	HGSLGFLPR	K			
0.00054	R	QSLLTQTSR	L			
0.0016	K	VDFAYSFFEK	Q			
8.50E-06	K	FIDTASIFGHGI	F			
0.00096	K	VGTEAHTAMT	T	Oxidation (M)	0.00000000200000.0	emPAI
0.0042	R	ILSTINR	E			
0.00062	R	GVLLYGPPGT(	T			
0.001	K	ISAAEIAAICQE	K	Oxidation (M)	0.0000000000000020000.0	
6.30E-05	K	ENAPAIIFIDEV	F			emPAI
1.20E-08	R	SSIFDANAGIG	S			
6.20E-07	K	GILGYTDEDV\	S			
1.10E-05	R	TGNENAQLTP	L			
9.50E-10	R	TESAAVSTIVN	E			emPAI
6.50E-06	K	YLSGLGIAR	Q			
0.00026	K	LLLDDVFEQK	N			
3.70E-08	R	GQIQAYVFDV	A			emPAI
1.90E-06	K	GVDGLFESFAI	L			
4.00E-09	R	AESQQVSESS(	S			emPAI
2.10E-05	R	AGQYGTVTSL\	D			

0.0032	R	TMVFANTVEA	A	Oxidation (M)	0.020000000000000000000.0	
1.60E-05	K	THGYLAPIIDQ	E			emPAI
0.00072	K	MVNADLAR	I	Oxidation (M)	0.20000000.0	
0.00025	R	MSLLAEAQR	V	Oxidation (M)	0.200000000.0	
0.0021	K	AGHQTSAESW	A			
2.00E-08	K	TMISDSDYTEF	W	Oxidation (M)	0.0200000000000000.0	emPAI
2.90E-06	R	TCYAQSSQIR	Q			
2.90E-07	K	ATQGIYPLQN	K			
0.0012	K	VDRPADEMA	-	Oxidation (M)	0.0000000200000000000.0	emPAI
0.002	K	IMFALTSIK	G	Oxidation (M)	0.020000000.0	
0.0075	K	IPDWFLNR	Q			
2.10E-08	R	AGELSAAEIDN	Q	Oxidation (M)	0.000000000000020000000.0	
5.20E-05	K	IGMVGLFTR	G			
1.30E-07	K	FASFETIVEMI	Y			emPAI
0.0016	K	SPEQVSAAVK	A			
2.00E-07	R	EGLVSDAIESFI	A			
0.0014	K	QVGYTPDYLFI	T			
0.00067	K	FQSVPVQAGC	G			emPAI
0.0065	K	LVAMATPEDN	I	2 Oxidation (M	0.00020000020.0	
1.50E-10	R	DLFEGILQAGS	T			emPAI
2.80E-06	R	APLGQNTVLLI	G			
1.10E-07	K	AGGECLTFDQ	A			emPAI
0.00062	K	GVLLYGPPGT	T			
0.0033	R	IDILDQALLRP	I			
3.50E-08	R	TMLELLNQLD	I	Oxidation (M)	0.020000000000000000.0	
0.0053	R	EHAPSIIFMDE	M			emPAI
1.40E-05	R	DAVLLVFANK	Q			
0.00041	R	ILMVGLDAAG	T	Oxidation (M)	0.00200000000.0	
7.30E-06	R	MLNEDELRDA	Q	Oxidation (M)	0.2000000000000000000.0	emPAI
3.30E-08	R	SIVGATLEVIQI	K			
0.0001	R	SDSQVFLFLNS	C			emPAI
6.60E-05	R	IEATTWDALIK	L			
4.20E-08	R	TLLANCVAAGI	K			emPAI
0.00039	K	IACYVTVR	G			
0.00029	K	LVLNISVGESG	L			
2.00E-06	K	VLEQLSGQTP	A			emPAI
0.0012	R	VGVHHLDDSC	N			
5.40E-08	K	SYEVTPSPADS	A			emPAI
5.80E-07	K	NPAIIVGAGLF	T			
3.40E-05	R	FASEVAGVQD	G			emPAI
0.00032	R	HLVFPIFEFLQI	Q			
5.20E-06	K	SLEEAAAPLVS	A			emPAI
6.80E-05	K	SVAAGMNAM	R	2 Oxidation (M	0.000002002000.0	
1.10E-06	R	TALVDAASVSS	D			emPAI
6.40E-05	K	DAGVIAGLNV	I			
0.00015	K	MKETAEAYLG	K	Oxidation (M)	0.20000000000.0	
5.50E-05	R	ITPSWVGFTDS	L			emPAI
4.70E-06	R	IGDLELFR	T			
0.00091	R	FEAHSNQQFC	Y			
0.00019	K	LTGSVLSSFLDI	L			emPAI
0.0043	R	VDSVFALR	S			
2.50E-09	R	VINLLLQYLSN	N			emPAI
0.0069	R	SGAYIVR	Q			
0.00035	K	ANVDYEQIVR	K			
3.50E-07	R	FVIGGPHGDA	K			emPAI
2.70E-06	K	AGALGDSVTIT	E			
1.90E-05	K	ITVTADGQFSK	R			emPAI
0.00035	R	SSETVAQLQK	T			

2.60E-07	K	IENHHQDDGL	K		emPAI
0.0037	R	FELLAR	L		
1.70E-08	R	TMLEIVNQLD(	G	Oxidation (M) 0.020000000000000.0	emPAI
4.80E-08	R	SSAESLFNLAK	Q		
0.0021	R	TLTESLNNGNI	F		emPAI
0.0032	K	LTIIEEAR	K		
0.0089	R	ECLPLVLIIR	N		
2.10E-06	K	FDVGNVVMV	N	Oxidation (M) 0.0000000200000.0	
0.00039	K	SRECLPLVLIIR	N		emPAI
1.70E-06	R	LADTYGSGELF	L		
0.00045	K	TEALLQEPFLK	N		
0.00037	R	VTELVPLVAEII	E		emPAI
0.0026	R	NHLANLNK	Y		
6.40E-08	K	VATELGLWQE	S		emPAI
0.0045	R	DIVELQLK	Q		
8.50E-05	K	QLDCELVIR	K		
1.30E-05	K	ESSASSPELATI	L		emPAI
1.90E-05	R	SALNALLFR	T		
2.30E-05	K	GLLLFIDEADA	N		emPAI
1.70E-07	K	VIELSDVILEVL	D		
0.0007	K	VIELSDVILEVL	C		emPAI
3.50E-05	R	LVELANEFSR	S		
6.30E-05	K	TVAFLLPAIEA\	S		
0.0029	K	VLVLDEADHLI	R	Oxidation (M) 0.0000000000002000.0	emPAI
0.0022	K	LPDIEVVR	V		
0.00067	K	SIESSDLNFTR	Y		
2.90E-05	K	LAIFTALAFSQI	L		
0.0097	K	SLEPFVNWLEI	-		emPAI
4.90E-08	R	VQIASLIGLLIR	H		
0.0014	R	SFVNLIESLLIKI	I		emPAI
3.70E-07	R	GLLGTVGTIAR	E		
0.0027	R	ALWTGLGPN\	N		emPAI
1.10E-05	K	IVNDNYLYAR	V		
0.0015	K	MSDIAPNLAA	L	Oxidation (M) 0.20000000000000000000.0	emPAI
0.0052	K	VVDSLYVR	S		
2.00E-06	K	EYVEALALLSTI	E		
0.0075	R	HLSSLYDTLLE(	L		emPAI
0.0015	K	VGDFEMNLK	R	Oxidation (M) 0.000002000.0	
3.70E-06	K	EGQVIGYLHQ	L		emPAI
8.30E-05	K	FYEILGVPK	S		
6.60E-05	K	EGMGGGGGG	Q	Oxidation (M) 0.00200000000000000000000000000000.0	emPAI
0.0011	K	WAEIIGQAR	H		
8.90E-06	K	LLSEEVFDFSR	G		emPAI
0.0051	R	NATWTLSNFC	G		
4.10E-06	R	IITVCLEGLENI	V		emPAI
8.30E-05	K	FYEILGVPK	T		
0.00024	K	VSLEDVYLGTT	K		emPAI
0.0027	K	YFVEAGAIAVF	R		
0.00022	R	EQLAIAEFADA	V		
0.0011	R	TLESNTVVAG(	E		emPAI
0.0013	K	AGALGDSVSIT	E		
1.90E-05	K	ITVTADGQFSK	R		emPAI
6.50E-05	K	TPGPGAQSAL	A		
0.00067	R	IEDVTPIPTDST	R		emPAI
2.20E-05	K	ILQLLR	L		
0.00012	K	LLFIIR	I		emPAI
0.00019	K	MGYGSAAVEL	Y	Oxidation (M) 0.2000000000000.0	
0.00034	K	LSQVELLVIDE/	S		emPAI

0.0029	K	TGFVFPR	V			
1.80E-05	K	LLPLPELLQSIS.	A			emPAI
0.0021	R	VLINAFTVR	A			
0.00021	K	AFQAVEQFLQ	Q			emPAI
0.00041	K	LAPSGFVLLR	D			
0.0049	R	IVSLLSESYNPH	Y			emPAI
0.0003	R	NPTLAGALTR	L			
0.0063	R	SDTIASLLAALI	T	Oxidation (M)	0.0000000000020000.0	emPAI
0.0027	R	VTYLVLDEADF	M			
0.0033	R	MLDMGFDPQ	K	Methyl (DE); 2	0.20020010000.0	emPAI
0.0078	R	DHACVVGGYF	M			
0.001	R	TNVIPIIEDAR	H			emPAI
0.004	K	DVIFVTTR	R			
0.0062	K	AVVIYVPFR	L			emPAI
0.0051	R	SKVEGAIESR	G			
0.0045	K	SLPLQTLIDILK	Q			emPAI
pep_expect	pep_res_befo	pep_seq	pep_res_after	pep_var_mod	pep_var_mod_pos	
0.0000011	K	SSVCDIAPK	G			
0.00000043	R	YLTASAVFR	G			
0.0086	K	IREEYPDR	M			
0.000003	K	LAVNLIPFPR	L			
0.000011	R	FPGQLNSDLR	K			
0.00000095	R	VSEQFTAMFR	R			
0.000087	R	KLAVNLIPFPR	L			
0.0000039	R	IDVYFNEASG(	Y			
0.000000035	R	MMMTFSVFP	V			
0.000000014	R	LHFFMVGFAP	G	Oxidation (M)	0.00002000000000.0	
0.00001	R	AVLMDLEPGT	S	2 Oxidation (M	0.000200000020000.0	
0.0000011	K	NSSYFVEWIPN	S			
0.000000018	R	EILHIQGGQC(	F			
8.5E-14	K	MASTFIGNSTS	R	2 Oxidation (M	0.20000000000000200.0	
4E-15	K	GHYTEGAELID	K			
0.0000036	K	GHYTEGAELID	E			
0.0036	-	MREILHIQGG(	F	Oxidation (M)	0.20000000000000000000.0	
0.00000099	R	GSQQYSALSVI	N			
0.000000001	K	LANPTFGDLNI	F	Oxidation (M)	0.0000000000000000200000000.0	
0.000092	R	SGPFGQIFRPD	G			
0.00037	K	FWEVICDEHG	I			
0.000013	K	EAENSDCLQG	I	Oxidation (M)	0.00000000000000000000000020000000.	emPAI
0.000068	R	YLTASAMFR	G			
0.0086	K	IREEYPDR	M			
0.000003	K	LAVNLIPFPR	L			
0.000011	R	FPGQLNSDLR	K			
0.00000095	R	VSEQFTAMFR	R			
0.000087	R	KLAVNLIPFPR	L			
0.00031	K	STVCDIPPTGL	M			
0.000000053	R	YTGDSDLQLEI	I			
0.00000035	R	MMLTFSVFPS	V	2 Oxidation (M	0.220000000000.0	
1.1E-09	R	INVYYNEASC(	F			
0.00000046	K	EVDEQMLNV(	N	Oxidation (M)	0.000002000000.0	
0.000000014	R	LHFFMVGFAP	G	Oxidation (M)	0.00002000000000.0	
0.00001	R	AVLMDLEPGT	S	2 Oxidation (M	0.000200000020000.0	
0.0000011	K	NSSYFVEWIPN	S			
0.00019	R	SLTVPELTQQN	N	Oxidation (M)	0.000000000020000.0	
0.000000018	R	EILHIQGGQC(	F			
0.000012	K	FWEVVCAEH(	Y			
8.5E-14	K	MASTFIGNSTS	R	2 Oxidation (M	0.20000000000000200.0	
4E-15	K	GHYTEGAELID	K			

0.0000036	K	GHYTEGAELID	E			
0.0036	-	MREILHIQGG(	F	Oxidation (M)	0.2000000000000000000.0	
0.000027	K	LTTPSFGDLNH	F	Oxidation (M)	0.000000000000000200000000.0	
0.00000052	R	SGPYGQTFRPI	G			emPAI
0.000068	R	YLTASAMFR	G			
0.0086	K	IREEYPDR	M			
0.000003	K	LAVNLIPFPR	L			
0.000011	R	FPGQLNSDLR	K			
0.00000095	R	VSEQFTAMFR	R			
0.000087	R	KLAVNLIPFPR	L			
0.00031	K	STVCDIPPTGL	M			
0.0000044	R	YVGDSELQLEF	V			
0.000000035	R	MMMTFSVFP	V			
0.00000014	R	VNVYYNEASC(	Y			
0.00000046	K	EVDEQMLNV(	N	Oxidation (M)	0.000002000000.0	
0.000000014	R	LHFFMVGFAP	G	Oxidation (M)	0.00002000000000.0	
0.0000075	R	AVLMDLEPGT	S	2 Oxidation (M	0.000200000020000.0	
0.0000011	K	NSSYFVEWIPN	S			
0.00013	R	NLTVPELTQQI	N	Oxidation (M)	0.000000000020000.0	
1.8E-10	K	FWEVVNLEHG	Y			
8.5E-14	K	MASTFIGNSTS	R	2 Oxidation (M	0.20000000000000200.0	
4E-15	K	GHYTEGAELID	K			
0.0000036	K	GHYTEGAELID	E			
0.00017	K	LSTPSFGDLNH	F			
3.6E-09	R	SGPYGQIFRPD	G			emPAI
0.00000043	R	YLTASAVFR	G			
0.0086	K	IREEYPDR	M			
0.000003	K	LAVNLIPFPR	L			
0.000011	R	FPGQLNSDLR	K			
0.00000095	R	VSEQFTAMFR	R			
0.000087	R	KLAVNLIPFPR	L			
0.00016	R	INVYFNEASG(	Y			
0.000000035	R	MMMTFSVFP	V			
0.000000014	R	LHFFMVGFAP	G	Oxidation (M)	0.00002000000000.0	
0.00001	R	AVLMDLEPGT	S	2 Oxidation (M	0.000200000020000.0	
0.0000011	K	NSSYFVEWIPN	S			
0.000000018	R	EILHIQGGQC(	F			
8.5E-14	K	MASTFIGNSTS	R	2 Oxidation (M	0.20000000000000200.0	
4E-15	K	GHYTEGAELID	K			
0.0000036	K	GHYTEGAELID	E			
0.0036	-	MREILHIQGG(	F	Oxidation (M)	0.2000000000000000000.0	
0.00000099	R	GSQQYSALSVI	N			
0.000000001	K	LANPTFGDLNI	F	Oxidation (M)	0.000000000000000200000000.0	
0.000092	R	SGPFGQIFRPD	G			emPAI
0.0034	K	FDLMYAK	R	Oxidation (M)	0.0002000.0	
0.000015	K	DVNAAVGTIK	T			
0.0048	K	EIVDLCLDR	I			
0.000088	K	YMACCLMYR	G	Oxidation (M)	0.020000000.0	
0.0046	R	IDHKFDLMYAI	R	Oxidation (M)	0.00000002000.0	
0.0061	R	QLFHPEQLISG	E			
4.5E-09	R	LVSQVISSLTAS	F			
0.000000085	R	TIQFVDWCPT	C			
0.0000058	R	SLSIERPTYTNL	L			
0.000000041	R	AVFVDLEPTVI	T			
1.2E-09	R	IHFMLSSYAPV	A			
1.6E-10	R	AVCMISNSTSV	I			
0.0000026	K	CGINYQPPTVV	V			
7E-11	K	TVGGGDDAFN	H			

0.0000058	R	AFVHWYVGE(	E	Oxidation (M)	0.00000000002000000000.0	
0.00000036	R	QLFHPEQLISG	G			
9.9E-12	R	FDGALNVDVT	I			
0.00000014	K	AFHEQLSVAEI	C	2 Oxidation (N	0.000000000000000000002200.0	
3.9E-11	R	KLADNCTGLQ	L			
0.00061	K	LGFTVYPSPQ\	R			emPAI
0.0034	K	FDLMYAK	R	Oxidation (M)	0.0002000.0	
0.000015	K	DVNAAVGTIK	T			
0.0048	K	EIVDLCLDR	I			
0.000088	K	YMACCLMYR	G	Oxidation (M)	0.020000000.0	
0.0046	R	IDHKFDLMYAI	R	Oxidation (M)	0.00000002000.0	
0.0061	R	QLFHPEQLISG	E			
4.5E-09	R	LVSQVISSLTA!	F			
0.000000085	R	TIQFVDWCPT	C			
0.0000043	R	SLNIERPTYTNI	L			
0.000000041	R	AVFVDLEPTVI	T			
1.2E-09	R	IHFMLSSYAPV	A			
1.6E-10	R	AVCMISNSTS\	I			
0.0000026	K	CGINYQPPTV\	V			
7E-11	K	TVGGGDDAFN	H			
0.0000058	R	AFVHWYVGE(	E	Oxidation (M)	0.00000000002000000000.0	
0.00000036	R	QLFHPEQLISG	G			
9.9E-12	R	FDGALNVDVT	I			
0.00000014	K	AFHEQLSVAEI	C	2 Oxidation (N	0.000000000000000000002200.0	
3.9E-11	R	KLADNCTGLQ	L			
0.00061	K	LGFTVYPSPQ\	R			emPAI
0.002	R	ALGDYQGVK	V			
0.000048	R	ELAQQIEK	V			
0.003	R	KVDWLTDK	M			
0.00065	R	DELTLEGIK	Q			
0.0055	R	GIYAYGFEK	P			
0.00000013	R	VLITTDLLAR	G			
0.000024	K	VHACVGGTSV	E			
0.000096	K	RDELTLEGIK	Q			
2.9E-10	K	GLDVIQQAQS	T			
0.000000033	K	GVAINFVTLDI	M			
2.3E-10	R	ILQAGVHVVV	V			
1.8E-11	R	KGVAINFVTLD	M			
6.2E-10	K	MFVLDEADEN	G	2 Oxidation (N	0.2000000002000.0	
0.00017	K	DQIYDIFQLLPI	I			
3.4E-10	R	GIYAYGFEKPS	G			
0.0002	K	FYNVVVEELPS	-			
0.000086	R	GFKDQIYDIFQ	I			
3.6E-10	K	IQVGVFSATM	K	Oxidation (M)	0.0000000002000000000.0	
0.000000008	K	LETLCDLYETL/	R			
0.000000088	R	GIDVQQVSLVI	I			
0.000000045	K	TATFCSGVLQ(	E			emPAI
0.000051	R	ALGDYLGVK	V			
0.000048	R	ELAQQIEK	V			
0.000028	K	GVAINFVTR	D			
0.003	R	KVDWLTDK	M			
0.00065	R	DELTLEGIK	Q			
0.0055	R	GIYAYGFEK	P			
0.0006	R	KGVAINFVTR	D			
0.00000013	R	VLITTDLLAR	G			
0.000024	K	VHACVGGTSV	E			
0.000096	K	RDELTLEGIK	Q			
2.9E-10	K	GLDVIQQAQS	T			

2.3E-10	R	ILQAGVHVVV	V			
6.2E-10	K	MFVLDEADEN	G	2 Oxidation (M	0.2000000002000.0	
0.00017	K	DQIYDIFQLLPI	I			
3.4E-10	R	GIYAYGFEKPS	G			
0.0002	K	FYNVVVEELPS	-			
0.000086	R	GFKDQIYDIFQ	I			
3.6E-10	K	IQVGVFSATM	K	Oxidation (M)	0.0000000002000000000.0	
0.000000008	K	LETLCDLYETLA	R			
0.000000088	R	GIDVQQVSLVI	I			
0.00054	K	LNEVLEGQDEI	G	3 Methyl (DE)	0.0010010010000000000000000200000000	emPAI
0.0000016	R	VEIIANDQGNI	T			
0.0000091	K	DAGVIAGLNV	I	Oxidation (M)	0.000000000020.0	
0.0032	R	MVNHFVQEFI	R	Oxidation (M)	0.2000000000.0	
0.000024	R	EIAEAYLGVTIK	N			
0.00001	R	FEELNMDLFR	K	Oxidation (M)	0.0000020000.0	
0.0000042	K	NALENYAYNM	N	Oxidation (M)	0.00000000020.0	
0.00019	K	ELESICNPIIAK	M			
0.00000075	K	STVHDVVLVG	I			
0.000000092	K	VQQLLQDFFN	E			
0.0000014	R	TTPSYVAFTDS	L			
0.0068	R	ARFEELNMDL	K	Oxidation (M)	0.000000020000.0	
0.00000017	K	EFAAEEISSMV	M			
1.2E-09	K	NQVAMNPVN	R	Oxidation (M)	0.000020000000000.0	
7.1E-09	K	ATAGDTHLGG	M			
0.004	K	NAVVTVPAYF	Q			
9.7E-11	K	SINPDEAVAYG	V			
0.00018	K	GEGPAIGIDLG	V			
4.5E-09	K	EQVFSTYSDN	A			
0.00017	K	IEDSIEQAIQW	M			
0.00023	R	TLSSTAQTTIEI	A			
0.00098	K	VQDLLLLDVTF	N	Oxidation (M)	0.00000000000000000000000002000000.0	
0.000000042	K	KIEDSIEQAIQV	M			emPAI
0.004	R	ALFELLK	L			
0.000039	K	TSLILALGK	Y			
0.002	K	LALETFSVR	C			
0.0000002	K	ALSGLQFLLCK	V			
0.0052	K	GLLYQPAYDPI	H			
5.4E-09	K	SLFESLNSVGV	R			
0.00024	K	VAAFPCFTSILS	G			
0.000000015	R	VFACQNPSTQ	K			
0.000019	K	LLAENLNCLLN	D			
4.7E-09	K	TATVAQILENV	D			
7.6E-10	R	LFLEENLASIFA	D			
0.0000012	R	DAVWCGIQILS	C			
0.00021	R	SLLSFIEILIPIAC	A			
0.000088	K	MDGIQNLISLI	L	Oxidation (M)	0.2000000000000000000.0	
0.00000004	R	QSYGSNPLQQ	F			
0.000013	K	MNYLDSFPFPY	D	Oxidation (M)	0.2000000000000000.0	
0.000019	R	ALHFNTSLLGS	A			
3.9E-11	K	QLFDNITAMLI	T	Oxidation (M)	0.000000002000000000.0	
0.0045	K	LPISDLLEPIISL	V			
0.00000029	K	ESNVVDPIINF	L			
5.8E-09	K	ALYDGFSMFFI	I	Oxidation (M)	0.00000002000000000000.0	
0.0011	R	GGHWIVLDEL	L			emPAI
0.0000027	R	FTDSSVQSDIK	L			
0.0000016	R	VEIIANDQGNI	T			
0.0000091	K	DAGVIAGLNV	I	Oxidation (M)	0.000000000020.0	
0.0032	R	MVNHFVQEFI	R	Oxidation (M)	0.2000000000.0	

0.00013	R	FEELNIDLFR	K			
0.000072	R	EIAEAYLGTTIK	N			
0.0000042	K	NALENYAYNM	N	Oxidation (M)	0.00000000020.0	
0.00019	K	ELESICNPIIAK	M			
1.2E-09	K	VQQLLVDFFN	E			
0.000000064	K	NSIDDVVLVG(	I			
0.0000014	R	TTPSYVAFTDS	L			
0.00041	R	ARFEELNIDLFI	K			
7.1E-09	K	ATAGDTHLGG	M			
2.2E-09	K	NQVAMNPIN	R	Oxidation (M)	0.000020000000000.0	
0.004	K	NAVVTVPAYF	Q			
4.6E-14	K	SINPDEAVAY(	V			
0.00018	K	GEGPAIGIDLG	V			
4.5E-09	K	EQVFSTYSDN(	A			
0.00071	K	VQDLLLLDVTF	N			
0.00000035	K	KIEDSIEAAIEW	M			emPAI
0.0011	K	SSVCDIAPR	G			
0.000068	R	YLTASAMFR	G			
0.0086	K	IREEYPDR	M			
0.000003	K	LAVNLIPFPR	L			
0.000011	R	FPGQLNSDLR	K			
0.00000095	R	VSEQFTAMFR	R			
0.000087	R	KLAVNLIPFPR	L			
0.00000047	R	YVGNSDLQLEI	V			
0.00000035	R	MMLTFSVFPS	V	2 Oxidation (N	0.220000000000.0	
0.00000014	R	VNVYYNEASC	Y			
0.00000046	K	EVDEQMINVC	N	Oxidation (M)	0.000002000000.0	
0.000000014	R	LHFFMVGFAP	G	Oxidation (M)	0.00002000000000.0	
0.0000011	K	NSSYFVEWIPN	S			
1E-14	K	GHYTEGAELID	K			
0.000027	K	LTTPSFGDLNH	F	Oxidation (M)	0.000000000000000200000000.0	
0.000011	R	TGPYGQIFRPD	G			emPAI
0.000031	R	DGYLTLFK	F			
0.0075	R	ALADPNTDVR	G			
0.0000041	R	VLPLIIPILSK	G			
0.000000013	K	LSILSAVASWA	S			
0.00000035	K	DLPAVMTFLIS	A			
7.2E-09	R	SPIVSAAAFEN	L			
0.000000055	R	QSSVELLGDLL	V			
0.00093	K	YALELLPVILPC	H			
0.000000029	R	SQLLSFMDQLI	T			
5.4E-10	K	SGPLPVDTFTF	I			
1.7E-12	R	VVLVIDEEGVE	G			
0.000018	R	NPDTISQISDLI	T			
0.000064	K	SLKPLLPVFLQ(	E			
9E-10	K	VVIDVLSSIVSA	R			
0.00000024	K	NPEISSLVPTLL	H			
0.00013	R	HALDTLLQTTF	G			emPAI
0.000068	R	YLTASAMFR	G			
0.0086	K	IREEYPDR	M			
0.000003	K	LAVNLIPFPR	L			
0.000011	R	FPGQLNSDLR	K			
0.00000095	R	VSEQFTAMFR	R			
0.000087	R	KLAVNLIPFPR	L			
0.0000036	R	YNGDSADLQL	I			
0.00000035	R	MMLTFSVFPS	V	2 Oxidation (N	0.220000000000.0	
0.000000014	R	LHFFMVGFAP	G	Oxidation (M)	0.00002000000000.0	
0.00001	R	AVLMDLEPGT	S	2 Oxidation (N	0.000200000020000.0	

0.0000011	K	NSSYFVEWIPN	S			
1E-14	K	GHYTEGAELID	K			
3.6E-09	R	SGPYGQIFRPD	G			emPAI
0.000037	K	VGAFSVLR	E			
0.0024	R	TTSPAAFTR	A			
0.00016	K	IDLDLEVR	L			
0.000015	K	CLAGLIVSR	S			
0.00031	K	VLPQALTLVK	S			
0.000068	K	HVVDDGLELR	K			
0.0000054	K	ALSSPVLAAV(	Y			
0.000035	R	TNIQMIGALCI	A	Oxidation (M)	0.00002000000.0	
0.00013	R	AELPSCLPVLV	M			
0.000000019	K	MDVFNTFIDLI	Q			
0.00000063	R	EYSLQALESFLI	C			
0.000039	K	LLNTLLLQLGC	K			
0.0000061	K	CPSAVLAVLDS	T			
5.5E-12	K	TVTCIASLASSI	A			
0.0000012	K	LDEIIFPQISSFI	D			
1.1E-12	R	LSSIILQQLDD\	C			
0.0029	R	ELVVVLPDCLA	A			
0.000036	K	DLSAHAGIETI	S			
0.00017	K	SAASYALGNIA	K			emPAI
0.000082	K	FEAIIYVLK	K			
0.00027	K	TVGAGVIGTIL	-			
2.9E-09	K	VGETVDLVGLI	E			
0.0000017	R	SYTVTGVEMF	I			
5E-11	K	ILDEALAGDN\	G			
0.000029	R	HYAHVDCPGH	N			
4.9E-10	R	GITINTATVEYI	H			
0.00022	K	IVVELIVPVACI	F	Oxidation (M)	0.0000000000000020.0	
0.00000016	K	TTLTAALTMAI	K			
0.000000021	K	IYELMDAVDD	Q	Oxidation (M)	0.00002000000000000.0	
5.5E-12	K	EDQVDDAELL	E			
2.3E-10	R	QTELPFLLAVE	G			
0.000037	K	WVDKIYELMD	Q			
0.0059	R	ELLSSYEFNGD	V			emPAI
0.0034	K	FDLMYAK	R	Oxidation (M)	0.0002000.0	
0.000015	K	DVNAAVGTIK	T			
0.0048	K	EIVDLCLDR	V			
0.000088	K	YMACCLMYR	G	Oxidation (M)	0.020000000.0	
0.0046	R	IDHKFDLMYAI	R	Oxidation (M)	0.00000002000.0	
0.0061	R	QLFHPEQLISG	E			
3.6E-11	R	LISQIISSLTTSL	F			
0.0000024	R	TVQFVDWCPT	C			
0.000047	R	SLDIERPTYTNI	L			
0.000000041	R	AVFVDLEPTVI	T			
0.000000017	R	IHFMLSSYAPV	A	Oxidation (M)	0.0002000000000000.0	
0.0000026	K	CGINYQPPTV\	V			
0.0000058	R	AFVHWYVGE(	E	Oxidation (M)	0.00000000002000000000.0	
0.00000036	R	QLFHPEQLISG	G			
3.7E-10	R	FDGAINVDITE	I			
3.9E-11	R	KLADNCTGLQ	L			emPAI
0.0004	K	VTVMDTLSR	L	Oxidation (M)	0.000200000.0	
0.0045	R	TCNYLTSAAR	Y			
0.00000021	R	VGQAVDVVG(	P			
0.00032	R	MLQYGEQNIR	R	Oxidation (M)	0.2000000000.0	
0.000013	K	QESVEATAEVS	T			
0.000015	R	NLAGEIAQEYT	R			

2.4E-09	R	FLPLGLGLLYL(	Q			
0.00058	K	AFHETMADSD	Y	Oxidation (M)	0.0000020000000.0	
0.00000018	R	AVPLALGLLCI!	V			
0.00024	K	YIPLSPILEGFIII	E			
0.0045	R	MMLTVDENLI	V	2 Oxidation (N	0.22000000000000000.0	
0.000000026	K	YLSDILSVLALT	E			
3E-11	R	QNLAATFVNA	L			
0.00014	R	AELATDKYIPL!	E			
0.0000016	K	NDCDPALALL(	I			
0.007	R	QSEEASIDDLN	H	Oxidation (M)	0.00000000000000000000020.0	
0.00000015	K	HNAETEAVDL	T	Oxidation (M)	0.00000000000200000000000000.0	
0.00096	R	IGINGFGR	I			
0.0024	K	AATYDEIKK	A			
0.0000031	R	VVDLIVHMSK	A			
0.009	R	AASFNIIPSSTG	A			
4.5E-11	R	VPTVDVSVVD	L			
0.000019	K	TLLFGEKPVTV	N			
0.000093	K	DAPMFVVGVI	S	Oxidation (M)	0.000200000000000.0	
0.0000006	K	LVSWYDNEW	V			
6.1E-12	R	FGIVEGLMTT\	T			
8.5E-14	K	GILGYTEDDV\	S			
3.8E-14	K	SDLDIVSNASC	V			
0.00063	R	NPEDIPWGEA	A			emPAI
0.0083	R	YDEIIK	E			
0.000041	R	QTVAVGVIK	S			
0.0000011	K	IGGIGTVPVGF	V			
0.0012	R	STNLDWYK	G			
0.00035	K	STTTGHLIYK	L			
7.9E-10	R	EHALLAFTLGV	Q			
0.00000071	K	YYCTVIDAPGH	D			
0.000068	K	FHINIVVIGHVI	S			
0.00000011	K	GPTLLEALDQI	R			
0.00012	K	PGMVVTFAPT	S	Oxidation (M)	0.00200000000000000.0	
0.000035	K	MTPTKPMVVI	F	2 Oxidation (N	0.2000002000000000000.0	
0.000038	R	VETGMIKPGN	S			
2.5E-12	K	NMITGTSQAD	D	Oxidation (M)	0.0200000000000000000000000000.0	
0.000051	R	ALGDYLGVK	A			
0.000048	R	ELAQQIEK	V			
0.003	R	KVDWLTDK	M			
0.00065	R	DELTLEGIK	Q			
0.0055	R	GIYAYGFEK	P			
0.00000013	R	VLITTDLLAR	G			
0.000096	K	RDELTLEGIK	Q			
2.9E-10	K	GLDVIQQAQS	T			
6.2E-10	K	MFVLDEADEN	G	2 Oxidation (N	0.2000000002000.0	
3.4E-10	R	GIYAYGFEKPS	G			
0.0002	R	FYNVVVEELPS	-			
0.000000012	K	VQVGVFSATN	K	Oxidation (M)	0.0000000002000000000.0	
0.000000008	K	LETLCDLYETL/	R			
0.000000088	R	GIDVQQVSLVI	I			emPAI
0.0086	K	LGVRPPK	G			
0.0028	R	ILQIHSR	K			
0.0093	R	ADILDPALMR	S	Oxidation (M)	0.0000000020.0	
0.0024	K	GVLLYGPPGT(	T			
0.000019	R	STDDFNGAQL	A			
0.000029	R	ACAAQTNATF	L			
0.0000029	K	AVCVEAGML/	R	Oxidation (M)	0.000000020000.0	
0.000011	K	LAGPQLVQMI	L			

0.00015	K	QIQELVEAIVLI	E	Oxidation (M)	0.0000000000002000.0	
0.000001	K	DSYLILDTLPSE	V			
9.4E-14	R	TMLELLNQLD	I	Oxidation (M)	0.020000000000000000.0	
0.0000024	K	EKAPCIIFIDEID	R			
0.0000024	R	DATEVNHEDF	K			
0.0000019	K	AMEVDEKPTE	Q	Oxidation (M)	0.0200000000000000000.0	
0.00000073	R	QTIFLPVVGLV	D			
0.0000056	K	QLPYLVGNIVE	K	Oxidation (M)	0.00000000000000200000000000000000	emPAI
0.0000016	R	VEIIANDQGNI	T			
0.0032	R	MVNHFVQEFI	R	Oxidation (M)	0.2000000000.0	
0.00001	R	FEELNMDLFR	K	Oxidation (M)	0.0000020000.0	
0.0000042	K	NALENYAYNM	N	Oxidation (M)	0.00000000020.0	
0.000000092	K	VQQLLQDFFN	E			
0.0000014	R	TTPSYVAFTDS	L			
0.0068	R	ARFEELNMDL	K	Oxidation (M)	0.000000020000.0	
0.000000005	K	NQVAMNPTN	R	Oxidation (M)	0.000020000000000.0	
7.1E-09	K	ATAGDTHLGG	M			
0.004	K	NAVVTVPAYF	Q			
4.6E-14	K	SINPDEAVAY(	V			
0.00018	K	GEGPAIGIDLG	V			
0.0000034	K	DAGVIAGLNV	I			
0.0000016	R	VEIIANDQGNI	T			
0.000072	R	EIAEAFLGTTVI	N			
0.0032	R	MVNHFVQEFI	R	Oxidation (M)	0.2000000000.0	
0.0000042	K	NALENYAYNM	N	Oxidation (M)	0.00000000020.0	
0.000000092	K	VQQLLQDFFN	E			
0.0000014	R	TTPSYVAFTDS	L			
1.2E-09	K	NQVAMNPVN	R	Oxidation (M)	0.000020000000000.0	
7.1E-09	K	ATAGDTHLGG	M			
0.004	K	NAVVTVPAYF	Q			
4.6E-14	K	SINPDEAVAY(	V			
0.00018	K	GEGPAIGIDLG	V			emPAI
0.0017	K	TDGLFLK	C			
0.0053	R	SLNLTLR	K			
0.0000028	R	VAEYAFLYAK	T			
0.000079	R	YDDVDLITIR	E			
0.0000035	K	TRYDDVDLITII	E			
2.4E-11	R	TADLGGSSTTI	A			
0.000000096	R	TQSFLTWESLE	R			
5E-14	K	NLANPTALLLS	H	Oxidation (M)	0.00000000000000200.0	
0.00054	R	ENTEGEYSGLE	G			
0.0015	K	ELNLYANVRP(	T			
3.9E-09	K	FNEQAEQIHS/	Y			emPAI
0.00021	K	SSLDAFK	Q			
0.0000058	K	GAGANILR	A			
0.000031	K	LQLIVFGK	K			
0.000000039	R	AVAGAGVLSG	L			
0.00000034	R	QFDGLVDVYR	K			
0.0000031	K	LLIQNQDEMII	A	Oxidation (M)	0.00000000200.0	
0.0017	R	TIKDEGFGSLW	G			
0.00000054	R	YFPTQALNFAI	D			
3.1E-09	K	GFTNFALDFLN	T	Oxidation (M)	0.00000000002000000000.0	
1.4E-13	K	WFAGNLASG(	T			emPAI
0.0000058	K	GAGANILR	A			
0.000031	K	LQLIVFGK	K			
0.0027	K	SDGIAGLYR	G			
0.00021	R	DEGIGSLWR	G			
0.00011	R	QFNGLVDVYK	K			

0.00000015	R	AVAGAGVLAC	L			
0.000048	K	SSFDAFSQIVK	K			
0.0000031	K	LLIQNQDEML	A	Oxidation (M)	0.00000000200.0	
0.0044	R	TIRDEGIGSLW	G			
0.00000054	R	YFPTQALNFAI	D			
0.00000026	R	GFNISCAGIIVY	G			
1.7E-10	K	GFTNFAIDFMI	T			
1.4E-13	K	WFAGNLASGC	T			emPAI
0.0068	K	RGILTLK	Y			
0.00000021	K	AGFAGDDAPF	A			
0.009	K	EITALAPSSMK	I	Oxidation (M)	0.00000000020.0	
0.00002	R	GYMFTTTAER	E	Oxidation (M)	0.0020000000.0	
0.0000024	K	SEYDESGPSIVI	K			
0.0003	K	IWHHTFYNELI	V			
1.9E-10	K	NYELPDGQVIT	F			
5.5E-11	K	LAYVALDYEQI	S			
0.0000014	K	YPIEHGIVSNW	I	Oxidation (M)	0.0000000000000200.0	
0.0000001	R	VAPEEHPVLLT	A			
4.6E-11	K	DLYGNIVLSGC	M	Oxidation (M)	0.000000000000020000000.0	
0.0000001	R	TTGIVLDSGDC	L			
0.00006	R	CPEVLFQPSLIC	C	2 Oxidation (N	0.0000000000002000000000000020.0	emPAI
0.0082	K	VQQLDVR	C			
0.00049	R	QAIVDGLR	N			
0.0000099	K	YLSGMGIAR	Q	Oxidation (M)	0.000020000.0	
0.0000019	K	LDLDSTFEQK	N			
0.0000053	R	ALAESAQDAFY	L			
0.000019	K	SNSVFIPHGPC	D			
0.00000019	R	NQIQAYVFDV	A			
1.1E-09	R	NSVLAFSESVP	D			
4.3E-13	K	DVMDMVLVT	E			
0.000075	K	AMSHYGYEIV	R	Oxidation (M)	0.0200000000000000000000000.0	emPAI
0.000071	K	KPDVDIEK	I			
0.000000062	R	ADIVVDVLVK	N			
0.000085	K	PTADVSAIHIPI	I			
0.0002	K	VKDFIHDIGEK	L			
0.0000015	K	IPLTLIYDDIK	S			
0.00000097	K	DFGSALWDM	G			
0.00000038	K	GNIDVDTPFG	L	Oxidation (M)	0.0000000000020.0	
0.00000023	R	VVHQEGDVEI	S			
0.0000013	K	FSLEETVAILH\	L			
0.000041	R	IKVDLIVDVPV	L			
0.000026	K	STYNDINPGM	I	Oxidation (M)	0.000000000200000.0	
0.00002	K	LEGTIGFGKPT	I			
1.6E-10	K	NPNPVPIPLID	K			
0.00038	R	VVQILAR	R			
0.0009	K	AEIANVLSR	G			
0.00063	K	TAIAEGLAQR	I			
0.0014	R	LDEMIVFR	Q	Oxidation (M)	0.00020000.0	
0.000028	R	HAQLPEEAR	E			
0.007	K	EIELQVTER	F			
0.000079	R	VLELSLEEAR	Q			
0.000000018	K	AIDLIDEAGSR	V			
0.0000024	K	LAEEGKLDPV\	Q			
0.00013	R	VLENLGADPSI	T			
0.000017	R	GSGFVAVEIPF	A			
0.0000014	K	MPTLEEYGTN	L	Oxidation (M)	0.2000000000000.0	
1E-10	K	VPEPTVEEAIQ	E			
0.0024	K	NPNRPIASFIFS	S			

4.6E-09	K	AHPDVFNMM	L			
0.00014	R	QLGHNYIGSEI	E			
0.000000021	R	LGHNFVGTEQ	V			
0.0099	R	QSDEIILFIDEV	G			emPAI
0.0028	R	ILQIHSR	K			
0.0093	R	ADILDPALMR	S	Oxidation (M)	0.0000000020.0	
0.0024	K	GVLLYGPPGT(	T			
0.000019	R	STDDFNGAQL	A			
0.000029	R	ACAAQTNATF	L			
0.0000029	K	AVCVEAGML/	R	Oxidation (M)	0.000000020000.0	
0.000011	K	LAGPQLVQMI	L			
0.00015	K	QIQELVEAIVLI	E	Oxidation (M)	0.0000000000002000.0	
0.000001	K	DSYLILDTLPSE	V			
9.4E-14	R	TMLELLNQLD(	I	Methyl (DE); (	0.02000000000000010.0	
0.0000024	R	DATEVNHEDF	K			
0.0000019	K	AMEVDEKPTE	Q	Oxidation (M)	0.02000000000000000000.0	
0.00019	K	QLPYLVGNIVE	K	2 Methyl (DE)	0.000000000000002000110000000000(	emPAI
0.0014	R	LSYALAR	S			
0.002	R	SSGFGDFGSDI	S			
0.00062	R	SSFGGFGSND(	R			
0.00063	K	TLAFGIPIIDK	I			
0.0000068	R	GSASMFEGIG	S	Oxidation (M)	0.000020000000.0	
0.0042	R	FTELPSIAVER	G			
2.1E-09	K	CEALHGDISQS	E			
0.003	R	DGHFNILVATI	G			
0.000041	R	QSMMFSATM	S	3 Oxidation (I	0.00220000200000.0	
0.00000007	K	LADGITTYSIIA	A			
5.9E-12	K	YLNNPLTVDL\	L			
0.00000095	R	ESAPSLDTICLY	Q	Oxidation (M)	0.0000000000000000000020.0	
0.00000012	R	VGDSESVGGD	A			
0.000067	R	RVGDSESVGG	A			emPAI
0.00025	R	GALANLFK	A			
0.0095	K	GVEYLIANK	L			
0.0019	R	FMDNLFLR	N	Oxidation (M)	0.02000000.0	
0.000027	R	NPASVAQFLR	S			
2.8E-09	R	GGLVSILNLGL	R			
0.0014	K	GSSLQCLVNVI	S			
3.5E-11	K	VTSAEVAQQA	S			
5.5E-11	R	NCALEVLFDLL	G			
4.9E-12	R	DASYTTQPLEL	K			
0.000000011	K	LPSESVVEFFT/	Q			
0.00028	R	MNATNDPED(	L	Oxidation (M)	0.2000000000000000000000000000.0	emPAI
0.00000012	K	VPLILGIWGGI	G			
2.7E-09	R	GLAYDTSDDQ	G			
0.000022	R	EGPPVFEQPEI	L	Oxidation (M)	0.000000000020000.0	
2.1E-13	R	VPIICTGNDFS	D			
4E-15	R	VQLAETYLSQ/	G			
0.000000046	K	IKDEDIVTLVD(	A			emPAI
0.00000059	R	TGGGGLDLSSI	R			
0.0021	R	KPTFPESSR	A			
0.0000098	R	ELLAQGVSQSI	Y			
0.000036	R	LQSLAFQLTEK	L			
0.000015	R	TMAQLGLCAF	A	Oxidation (M)	0.02000000000.0	
0.000000065	R	VWGNLVAFLE	V			
0.000042	R	AMMDILVSLI\	N			
0.0000067	R	FELVDQLTHLT	I			
0.000000041	K	AFEVLNSLEVV	L			
0.00000034	K	CVLNMLTILDI	Y			

0.00000016	K	TLVMLEDFLNI	E	Oxidation (M)	0.0002000000000000.0	
0.000000011	R	LRDEPMFLAL/	M	Oxidation (M)	0.00000200000000000000.0	emPAI
0.00066	R	VVGSELIQK	Y			
0.0085	K	GVILYGEPGTC	T			
0.000028	K	AVANSTSATFL	V			
0.000068	K	AICTEAGLLALI	E			
4.9E-12	R	TMLELLNQLD	G			
0.000022	R	IKDYLLMEEEF	L	Oxidation (M)	0.00000020000000000.0	
1.6E-11	K	APLESYADIGG	E			
0.0000003	K	VLSVVGILQDE	V	2 Oxidation (N	0.00000000000000200020.0	
0.000000011	R	VADDLSPSIVF	R			emPAI
0.00063	K	TAIAEGLAQR	I			
0.0014	R	LDEMIVFR	Q	Oxidation (M)	0.00020000.0	
0.007	K	EIELQVTER	F			
0.000079	R	VLELSLEEAR	Q			
0.000000018	K	AIDLIDEAGSR	V			
0.0000024	K	LAEEGKLDPV\	Q			
0.00013	R	VLENLGADPSI	T			
0.000022	R	VVDEGYNPSY	P			
0.000017	R	GSGFVAVEIPF	A			
0.0000014	K	MPTLEEYGTN	L	Oxidation (M)	0.2000000000000.0	
0.0024	K	NPNRPIASFIFS	S			
4.6E-09	K	AHPDVFNMM	L			
0.00014	R	QLGHNYIGSEI	E			
0.000000021	R	LGHNFVGTEQ	V			
0.0099	R	QSDEIILFIDEV	G			emPAI
0.0068	R	INAEDPFK	G			
0.0057	K	LILDVEDFK	N			
0.00053	K	FGNVVHFGER	D			
0.0055	R	YKQEDIVLR	G			
0.00051	R	HIEFQVLADK	F			
0.000073	R	VANEIGFPVM	A	Oxidation (M)	0.000000000200.0	
0.0000056	K	LLEEAPSPALT/	K			
7.2E-10	K	SEAAAAFGND	F			
1.2E-10	K	HEEELAEPQEI	D			
0.00021	R	ALNDTIITGVP	L			
5.1E-09	K	NAGVPTVPGS	V			
0.0000063	R	IQVEHPVTEM	V	Oxidation (M)	0.0000000002000000000000.0	
0.0000019	K	AMGDAAVAA	G	Oxidation (M)	0.0200000000000000000000000000.0	
0.00078	R	KAMGDAAVA	G	Oxidation (M)	0.00200000000000000000000000000.0	emPAI
0.0002	K	GELLVGTPAK	R			
0.00000056	K	VQEIVSEIFGK	S			
0.00032	R	SSGGLSDDEIN	M			
0.000023	K	MKETAEAYLG	S	Oxidation (M)	0.20000000000.0	
0.00016	R	TAMAGEDVEI	A	Oxidation (M)	0.002000000000.0	
0.000048	K	FSPSQIGANVL	M			
0.0000021	K	EVDEVLLVGGI	V	Oxidation (M)	0.0000000000200.0	
0.0000018	R	QAVTNPTNTII	R			
2.7E-11	K	IPAEIASEIETA	T			
0.0000014	R	IINEPTAAALSY	E	Oxidation (M)	0.00000000000002000.0	
0.00000069	K	SQVFSTAADN	V	Oxidation (M)	0.00000000000200000.0	emPAI
0.0067	R	FFAFGR	V			
0.0098	K	FGVVESK	M			
0.007	K	SDPVVSFR	E			
0.0037	R	IRPVLTVNK	M			
0.0041	R	RVIYASQITAK	P			
0.0000025	R	LWGENFFDPA	K			
3.2E-10	R	PMEEGLAEAII	I	Oxidation (M)	0.02000000000000.0	

0.000001	K	DLQDDFMGG	S	Oxidation (M)	0.00000020000000.0	
0.000061	K	AYLPVVESFGF	A			
1E-10	K	STLTDSLVAAA	M			
0.0041	R	YRVENLYEGPI	N			
7.2E-11	R	DGNEYLINLID	I			emPAI
0.000000045	K	IQTVIQAGVVF	L			
0.00063	R	NATWTLSNFC	G			
3.5E-09	R	SPPIEEVISAG\	F			
3.5E-10	R	IITVCLEGLENI	V			
1.3E-12	R	EQAVWALGN'	C			
0.00000024	K	LVELLLHHSPS'	T			
0.000034	R	LIHSDDEEVLT	I			emPAI
0.0063	R	VPPEVMESIR	K	Oxidation (M)	0.0000020000.0	
0.000046	R	HENVDIVVIR	E			
0.00021	R	HLQFPSFADR	L			
0.00000087	K	LADGLFLESCR	E			
0.0000014	K	YAFEYAYLNNI	K			
4.6E-10	K	TPVGGGVSSLI	K			
0.000042	K	ANPVALLLSSA	H			
0.000037	K	DLGGTSTTQE'	L			
0.00085	R	TKDLGGTSTT(	L			
0.0000012	K	ELDLFASLVNC	H			emPAI
0.000088	K	STVVNTHLMP	S	Oxidation (M)	0.00000000200.0	
1.7E-10	K	AADQGLVILQ!	R			
0.000000005	R	EDGEPVGYND	K			
1.2E-11	K	SSEVEALISEITI	L			
0.000075	K	ILREDGEPVGY	K			
5.7E-09	K	EGQVLCYIEQL	I			
0.0099	R	KLTDESSPPPC	T			emPAI
0.00057	R	SGAVASAVMI	K			
0.0019	K	ISWVPDPK	T			
7.4E-11	R	GYAATAAQGS	S			
8.4E-13	R	PETGSNEIDA/	A	Methyl (DE)	0.01000000000000.0	
0.000000018	K	TGYYRPETGSI	A	Methyl (DE)	0.0000001000000000000.0	emPAI
0.000012	K	TLVLALATGNI	K			
0.00021	K	VVSAMVNND	S	Oxidation (M)	0.00002000000.0	
0.00013	K	EAVDQCITANI	M			
0.005	R	TTFVPSTPPAL	N			
0.000012	K	IPGVISASSFD(	I			
0.000016	K	IGIYNIEGCSR	Y			
0.0000015	R	LFNETSEALGC	A			
0.0025	K	DFMPSDTDFS	G	Oxidation (M)	0.002000000000.0	
5.8E-09	K	VLDSGGLSPEL	D			
0.0000056	K	LFESYAEILASC	Y	Oxidation (M)	0.0000000000000000020.0	
0.00000028	K	GTSSILSEVFLF	T			
0.0025	K	IGLFGGAGVGI	T			
0.0026	K	VVDLLAPYQR	G			
0.00017	R	TIAMDGTEGL'	G	Oxidation (M)	0.000200000000.0	
0.00026	K	VLNTGAPITVF	A			
1.3E-09	R	VGLTGLTVAE'	D			
0.00000011	K	TVLIMELINNV	A			
4.1E-11	R	FTQANSEVSAI	I			
0.00000014	R	DAEGQDVLLF	F			
0.00013	K	NLQDIIAILGM	A	Oxidation (M)	0.000000000020000000000000.0	emPAI
0.0082	R	VQQLDVR	C			
0.00049	R	QAIVDGLR	D			
0.0000005	K	YLSGLGIAR	Q			
0.0002	R	DGLLQGSSAN	-			

0.000013	K	LLLDDVFEQK	N			
0.00076	K	SSAVFIPHGPG	D			
7.7E-10	R	GQIQAYVFDV	A			
0.0000013	R	DSVLGFAVNV	D			
0.000000039	K	DVMDMVLVT	E	2 Oxidation (M	0.0020000000000000020.0	
0.000013	K	AMSAYGYEIV(	R	Oxidation (M)	0.020000000000000000000000.0	emPAI
0.0037	R	LAFGLAK	S			
0.0038	R	ASIIGPLVK	E			
0.00062	R	SSFGGFGSND(	R			
0.000000054	R	GSASMFEGVG	S	Oxidation (M)	0.000020000000.0	
0.00063	K	TLAFGIPIIDK	I			
0.000031	K	CEALHGDISQ/	E			
0.00055	K	KGSAILIHGQD	A	Methyl (DE)	0.00000000001000.0	
0.00000002	R	DGNFSILVATD	G			
0.000041	R	QSMMFSATM	S	3 Oxidation (M	0.00220000200000.0	
7.8E-09	R	SSGFGSFGSG(	S			
0.0002	K	YLNNPLTIDLV	L			
0.00000095	R	ESAPSLDTICLY	E	Oxidation (M)	0.00000000000000000000020.0	emPAI
0.00000012	K	LLIEYLTIADK	T			
0.0000039	K	TELQVLVNEG	I			
1.1E-13	K	TSAADVLQLLT	M			
0.00000005	R	LIDVCVENAEE	F			
8.6E-11	K	QELLAELYSTE	G			
0.00017	K	MAFDQFGSM	L	2 Oxidation (M	0.20000000020000000000000.0	emPAI
0.00047	R	LLQAFLQK	A			
0.00043	K	QFLVASVSR	L			
0.00013	K	GVWSALFTR	V			
0.000013	R	LSQVLGIFEK	L			
1.6E-09	R	IQSQLSEALTV	H			
1.9E-09	R	ALSDAADQAN	L			
0.000000067	K	YCLDNFAAPLT	T			
5.9E-09	R	ETLVFLSQCFLI	R			emPAI
0.0043	R	VDMLMVER	T	2 Oxidation (M	0.00202000.0	
0.00019	R	ICGTIAADEK	R			
0.00000024	R	AQEFVCGLAQ	I			
3.5E-11	K	LLEIDPNGAVS	K	2 Oxidation (M	0.0000000000000000220.0	
4.1E-13	R	LEVYTADDYAI	W			
0.0000019	R	TVQHLIGSGM	A	Oxidation (M)	0.0000000002000000000000000000000.0	emPAI
0.00008	K	VLDFELVR	D			
0.0014	R	GINDTYFGR	L			
0.000058	R	FMVQNVVDLI	A	Oxidation (M)	0.0200000000.0	
0.0000088	K	SLLEEYFSIR	L			
0.00044	K	KINEIHSEAER	N			
0.0000023	K	STWQGSGPTF	A			
0.000000011	R	AAVSGAADGN	P	Oxidation (M)	0.000000000200000000.0	
0.000000065	R	VLLNNCQEAFI	L			
7.5E-10	K	APNSFGEFLGE	V			
0.0021	K	IVHHIVQELLG	A			emPAI
0.000079	K	DVIVAAETGS(	T			
0.0011	K	MGQPVETAFS	K	Oxidation (M)	0.20000000000.0	
0.0000045	R	MVNGLVDED(	V	Oxidation (M)	0.200000000000000.0	
4.8E-09	R	AGQYGTVTSL	D			
2.1E-10	R	TMVFANTVEA	A	Oxidation (M)	0.02000000000000000000.0	
0.0000039	R	GVDVPNVSH\	I			emPAI
3.4E-09	R	SSIFDANAGIG	S			
0.00023	R	GASQNIIPSST(	A			
4.4E-10	R	VPTSNVSVVD	L			
0.00045	R	VLDLIEHMAL\	-			

9.3E-13	K	GILGYTDEDV\	S			
0.0000031	K	RDPSEIPWAD	A			emPAI
0.0053	R	SLNLTLR	K			
0.0000028	R	VAEYAFLYAK	T			
0.000079	R	YDDVDLITIR	E			
0.0000035	K	TRYDDVDLITII	E			
2.4E-11	R	TADLGGSSTT	A	Methyl (DE)	0.000000000001000.0	
0.0000074	K	ATLFPGDGIGF	Q			
0.0036	K	QAEQIHSAIIN	Y			
0.0015	K	ELNLYANVRP(	T			emPAI
0.00000015	R	DAVLLVFANK	Q			
0.000011	R	ILMVGLDAAG	T	Oxidation (M)	0.00200000000.0	
7.3E-10	K	NISFTVWDVG	I			
0.00000005	K	QDLPNAMNA	L	Oxidation (M)	0.000000200000000.0	
0.000032	R	MLNEDELRDA	Q	Oxidation (M)	0.2000000000000000000.0	
0.00000065	R	HYFQNTQGLII	D			
0.0000076	K	LGEIVTTIPTIG	N			emPAI
0.004	R	VGIKPPK	G			
0.0027	K	VVSSAIIDK	Y			
0.0024	K	GVLLYGPPGT(	T			
0.000026	K	HGEIDYEAIVK	L			
0.00027	R	AIASNIDANFL	V			
0.0039	R	DYVIHEDFMK	A	Oxidation (M)	0.0000000020.0	
0.00000016	R	VVLDMTTLTIN	A			
0.0000036	R	NICTEAGMFA	A	Oxidation (M)	0.000000020000.0	
0.000000013	R	TLMELLNQLD	V	Oxidation (M)	0.00200000000000000.0	
0.0000023	K	SLQSVGQIIGE	L			emPAI
0.000011	K	FSLGLAEPK	L			
0.00018	K	IVNDNYLYAR	V			
0.0086	K	YGLIFHSSFIGR	A			
0.00000058	K	CPSSTLQILGAI	A			
0.0000007	R	IDCFADGATTA	L			
3.4E-09	K	IPCQSNEFVLE	G			
0.000000027	K	MSDIAPNLAA	L			
0.0011	R	VDNMVIQAIF	V	2 Oxidation (N	0.00020000000000000000000020.0	emPAI
0.0068	K	RGILTLK	Y			
0.00000021	K	AGFAGDDAPF	A			
0.0018	R	DLTDYLMK	I	Oxidation (M)	0.00000020.0	
0.009	K	EITALAPSSMK	I	Oxidation (M)	0.00000000020.0	
0.0003	K	IWHHTFYNELI	V			
0.0000014	K	YPIEHGIVSNW	I	Oxidation (M)	0.0000000000000200.0	
0.0000001	R	VAPEEHPVLLT	A			
9.7E-10	K	DLYGNIVLSGC	M	Oxidation (M)	0.000000000000020000000.0	
0.0000001	R	TTGIVLDSGDC	L			emPAI
0.0011	K	LLVLLQNGK	Q			
0.000062	R	SSAESLFNLAK	Q			
0.00003	K	ILIVFAEVLTGK	D			
0.000082	K	EGFFPWIDQV	F			
9.7E-09	K	IAALNAVISFV(	D			
0.0055	K	NLDQVVSMVI	V	Oxidation (M)	0.0000000200000000000.0	
0.0023	K	ASFLPFFDELS!	D	Oxidation (M)	0.00000000000000002000.0	
0.0000011	R	QLVDIVGSML	H			
0.000055	K	QMVQEGALTA	Y	Oxidation (M)	0.0200000000000000000000000.0	
0.0043	R	WAAINAIGQL	V			
0.000082	K	LQESAFLILAQI	E			emPAI
0.00013	K	VIEVEGPR	G			
0.000066	K	FLDGIYVSEK	S			
0.00069	K	HLNLDFQLIK	D			

0.00000064	R	KVEMLDGVTI'	S	Oxidation (M)	0.000200000000.0	
0.00000042	R	TALSHVDNLIS	G			
0.0034	R	FVYAHFPINAS	S			
2.2E-10	K	TILSSETMDIPI	V	Oxidation (M)	0.00000002000000000.0	
0.006	K	HLNLDFQLIKD	K			
0.0000051	K	VKDEIVLDGNI	S			emPAI
0.0000059	K	IADVLLLMEK	E	Oxidation (M)	0.0000000200.0	
0.0000002	R	ASSSTYSVLLR	V			
0.00000012	K	DLLSIYASLGR	E			
0.00000031	R	IEATTWDALIK	L			
0.000000028	R	TLLANCVAAGI	K			
0.00012	K	TIVSAPGLSIGS	W			
0.00034	K	GATNDISGME	H			emPAI
0.0054	K	VQQITIR	S			
0.0002	K	GELLVGTPAK	R			
0.000078	K	EIEDAVADLR	S			
0.00000083	K	VQSIVAEIFGK	S			
0.0000042	R	SSGGLSEDDIC	M			
0.000023	K	MKETAEAYLG	S	Oxidation (M)	0.20000000000.0	
0.000045	R	QAVTNPTNTV	R			
0.0000021	K	EVDEVLLVGGI	V	Oxidation (M)	0.0000000000200.0	
0.000089	R	SRFETLVNHLII	T			
0.00000027	K	ELLLLDVTPLSI	L			
0.0046	K	GMQVLEFVGI	E	Oxidation (M)	0.0200000000.0	
0.002	R	LESLHSEGVHF	L			
0.0000067	R	EAGSGAPSLLE	A			
0.000000018	R	LEGASEESLLS(	V			
0.000000015	K	STGGWGWGF	A			
6.5E-12	K	VFDDSVESFTS	G			
0.00023	K	AADMATGFTI	T	2 Oxidation (I	0.0002000000000000000002000.0	emPAI
0.0068	K	RGILTLK	Y			
0.00000021	K	AGFAGDDAPF	A			
0.0002	R	GYSFTTTAER	E			
0.009	K	EITALAPSSMK	I	Oxidation (M)	0.00000000020.0	
0.0003	K	IWHHTFYNELI	V			
1.3E-09	K	NYELPDGQVIT	F			
0.0079	R	VAPEEHPILLTI	A			
0.0077	K	YPIEHGIVNNV	I	Oxidation (M)	0.0000000000000200.0	
9.7E-10	K	DLYGNIVLSGC	M	Oxidation (M)	0.000000000000020000000.0	
0.0000001	R	TTGIVLDSGDC	L			emPAI
0.0043	K	YVLEGHDR	G			
0.0068	R	GTGGSAVFIAF	N			
0.0014	R	GWSESSSPNV	G			
0.0071	K	GIFEGGLESAN	G			
0.0000039	R	AITINATEYIFK	L			
3E-10	R	LSFLYLITGNLD	L			
0.00000012	R	GPPALVFDFSC	L			
6.9E-10	R	VFLSILQTIPLV	R			emPAI
0.0026	K	LPDIEVVR	V			
0.001	K	SIESSDLNFTR	Y			
0.00000011	K	LAIFTALAFSQI	L			
0.0048	R	EAILPSVVYIQI	I			
0.0000088	K	GIVLSFVTDFFI	E			
0.00000045	R	FLQSLELFEENI	K			
0.00014	K	VTEESNVDEVI	Q			
0.000000018	K	EYLVENSLEDL	R			
0.000000038	K	SLEPFVNWLEI	-			emPAI
0.00011	K	DGFFILK	E			

0.00062	R	LADTYGSGELF	L			
0.00059	K	TEALLQEPFLK	N			
0.00018	K	LFMENGIEELA	K	Oxidation (M)	0.002000000000.0	
0.000022	R	FGFNLLVGGFI	R			
0.000000013	R	VTELVPLVAEII	E			
0.00094	K	QEGLSFVGLHY	L			
1.2E-10	K	GLASVGLTSLC	N	Oxidation (M)	0.000000000000020000.0	emPAI
0.0033	R	FVEFLTR	E			
0.00018	K	IQSVIEAGVVP	L			
0.00063	R	NATWTLSNFC	G			
0.0057	R	SPPIEEVIDAG\	F			
3.5E-10	R	IITVCLEGLENI	V			
1.3E-12	R	EQAVWALGN'	C			
0.0019	R	DLVLGQGALIF	L			
0.0000011	K	VVIEHGAVPIF	E			emPAI
0.00001	K	LADLVGVTLGI	G			
0.0000059	R	DLVGVLEDAIF	G			
0.000000067	K	VVAAGANPVL	G			
0.0000029	K	YEDLMAAGIID	V	Oxidation (M)	0.00002000000000.0	
2E-12	R	GGYPILIIAEDII	L			emPAI
0.0083	K	APGILER	K			
0.00024	K	AVDSLVPIGR	G			
0.0012	R	TGSIVDVPAGI	A			
0.00059	R	VVDAMGVPID	G	Oxidation (M)	0.000020000000.0	
0.0029	K	SVHEPMQTGL	A	Oxidation (M)	0.00000200000.0	
0.000015	R	AAELTNLFESR	I			
0.0000014	K	TTIAIDTILNQK	Q			
4.3E-14	R	EVAAFAQFGSI	G			
0.00052	K	VVDSLYVR	S			
0.00028	K	LRPFFSLIPK	A			
0.0028	K	AQLEDDPIVHI	H			
0.0000088	K	AGLQYVGPDL	A	Oxidation (M)	0.00000000000020.0	
7.4E-10	K	EYVEALALLSTI	E			
0.0077	R	HLSSLYDTLLE(	L			
0.000000086	R	VEIAHIAELIGL	K			
5.2E-10	K	TGYSYFFEAFE:	A			emPAI
0.00043	K	VLELALK	K			
0.0024	K	AGIIDPLK	V			
0.0053	R	GVEDLADAVK	V			
0.0000098	K	IGVQIIQNALK	T			
0.000000062	K	SVAAGMNAM	R	2 Oxidation (M	0.000002002000.0	
0.0000059	K	LAVDTVVTNL(	A			
0.0000041	K	TLFNELEVVEG	I	Oxidation (M)	0.0000000000020.0	
0.000000058	K	LLEQDNPDLG'	G			
0.00000065	R	TALVDAASVSS	E			emPAI
0.00061	K	ALEAVLPK	P			
0.00015	K	FYEILGVPK	S			
0.0000025	K	VSLEDVYLGTN	K	Oxidation (M)	0.000000000020.0	
0.0033	K	SNPGEVVKPD	A			
0.0000051	R	EIYDQYGEDAI	E			
0.0032	R	QLGPGMIQQI	G	2 Oxidation (M	0.000002000200000000.0	
0.00000027	K	ITFEGQADEAF	E			
3.5E-09	K	EGMGGGGGG	Q	Oxidation (M)	0.0020000000000000000000000000000.0	
0.0000047	R	KGEDLFVEHTI	S			emPAI
0.006	R	SGAYIVR	Q			
0.0048	K	SIVASGLAR	R			
0.0061	K	TAAYGHFGR	D			
0.0000033	K	ANVDYEQIVR	K			

0.0022	K	IPDKEILEIVK	E			
0.000000059	R	FVIGGPHGDA	K			
0.0011	K	TNMVMVFGE	A	2 Oxidation (N	0.00202000000000.0	
0.000047	R	DDADFTWEV\	S			
0.000000041	R	EIGFVSADVGL	V			
0.0041	K	VLVNIEQQSPI	K			
0.0000051	R	VHTVLISTQHI	E			emPAI
0.0024	K	GVLLYGPPGT(	T			
0.00001	K	AVCTEAGMFA	E	Oxidation (M)	0.000000020000.0	
0.0038	R	IDILDQALLRP(	I			
0.0043	R	RVHVTQEDFE	V	Oxidation (M)	0.000000000020000.0	
0.00000066	K	VPDSTYDMIG	E	Oxidation (M)	0.00000002000000000.0	
1.4E-10	R	TMLELLNQLD	I	Oxidation (M)	0.02000000000000000.0	
0.000082	R	MLREELQLLQI	V	Oxidation (M)	0.200000000000000000000.0	emPAI
0.0002	R	SIVALIASR	G			
0.0000048	K	AVVTFFDAILD	T			
2.1E-10	K	MGYGSAAVEL	Y	Oxidation (M)	0.2000000000000.0	
0.00000015	K	LSQVELLVIDE/	S			
9E-13	K	SLLGPYLVFLSS	S			emPAI
0.00059	K	WAEIIGQAR	H			
0.0000029	K	LLSEEVFDFSR	G			
7.6E-10	K	FFALQVLEGVI	Y			
1.4E-09	R	NIAETGLNLLLI	N			
0.000021	R	DLSQPIDVGVI	E			
0.0000039	K	LRDLSQPIDVG	E			emPAI
0.00026	K	YFVEAGAIAVF	R			
0.000000015	R	EVGDGTTSVV	R			
1.7E-09	R	EQLAIAEFADA	V			
8.9E-11	K	LISGDSDFFAN	M			
0.00043	R	TLESNTVVAG(	E			emPAI
0.0068	K	RGILTLK	Y			
0.00000021	K	AGFAGDDAPF	A			
0.0018	R	DLTDYLMK	I	Oxidation (M)	0.00000020.0	
0.009	K	EITALAPSSMK	I	Oxidation (M)	0.00000000020.0	
0.00002	R	GYMFTTTAER	E	Oxidation (M)	0.0020000000.0	
0.0003	K	IWHHTFYNELI	I			
1.9E-10	K	NYELPDGQVIT	F			
0.0035	K	YPIEHGVVSN\	I	Oxidation (M)	0.0000000000000200.0	
0.0000014	R	EATTGFLTNLP	I			
2.5E-10	R	VQIASLIGLLIR	H			
0.000000088	R	SFVNLIESLLIK	D			
0.00056	R	SFVNLIESLLIKI	I			
0.00024	K	NLFPSLLSIIEQ	G			
0.000000034	R	SASGWQVSN/	K			
0.0075	K	LPEESIYGLAYI	G			emPAI
0.00000014	K	SVAAGSATVA'	S			
0.00000059	K	SGAAPVVSAA	V			
0.00011	R	FGSWPDGLDS	I			
3.8E-10	K	QFSGVDILSGE	V			
0.000000002	K	ADGLVDMPYI	F	Oxidation (M)	0.0000002000000000000000.0	emPAI
0.00098	K	SEVLDFIR	S			
0.0081	K	NALYQATIK	Q			
0.0023	K	VVDAPEILTVL	I			
7.6E-12	K	IADAEENLGES	E			
0.000000025	K	AFGVSVDFIDC	F			
0.0000042	R	SHGMAPLYET	A	2 Oxidation (N	0.00020000000000000000000000020.0	emPAI
0.0051	R	SISCWTLSR	F			
0.0031	K	YLIQESGNPK	G			

0.01	K	SELLPCLGAAD	N			
0.0000014	R	IVYDAIGTLAD	E			
0.0012	R	LLQFFQSPHAS	K			
8E-11	R	LLDFLEIASQQ	E			
0.00019	R	LHGSEDFDDD	K			
0.0073	K	CSAAAIDVLSN	N			
0.000087	K	ICEDIPHVLDTI	L			emPAI
0.0004	K	VTVMDTLSR	L	Oxidation (M)	0.000200000.0	
0.00000021	R	VGQAVDVVG(	P			
0.000013	K	QESVEATAEV!	T			
2.4E-09	R	FLPLGLGLLYL(	Q			
0.00000018	R	AVPLALGLLCI!	V			
0.000036	K	YIPLSPILEGFV	E			
0.006	K	IGIGQPK	R			
0.000027	R	VDEIITCAPR	R			
0.00069	R	GASHHVLDEA	S			
0.000024	K	DSFLDEGFILDI	K			
0.00054	K	DSFLDEGFILDI	I			
0.000017	R	SLHDALCVLSC	V			
0.0000001	K	VQDDEVGDG	E			
0.000002	R	ALVAIPTTIADI	A			
0.0037	R	QLIYNFPEELF/	L			emPAI
3.6E-09	K	ALDALIAFLR	A			
0.000000099	K	EIIEVLDVLLR	W			
0.0000001	K	AGCLNLVDVIE	T			
0.000000036	K	VDIFSQLQNA!	T			
0.002	K	NVQVQQQVIE	F			emPAI
0.00043	K	VLELALK	R			
0.0024	K	AGIIDPLK	V			
0.0053	K	GVEDLADAVK	V			
0.0000098	K	IGVQIIQNALK	T			
0.000000062	K	SVAAGMNAM	R	2 Oxidation (M	0.000002002000.0	
0.000067	R	GYTSPYFITNQ	T			
0.0000041	K	TLFNELEVVEG	L	Oxidation (M)	0.0000000000020.0	
0.000000058	K	LLEQDNPDLG	G			emPAI
0.000046	R	HENVDIVVIR	E			
0.00021	R	HLQFPSFADR	L			
0.0000014	K	YAFEYAYLNNI	K			
0.000000001	K	TPVGGGVSSLI	K			
0.00000037	K	ELDLFASLVNC	H			emPAI
0.00032	K	LAIVNVGR	Q			
0.000008	R	AVAVVVDPIQ	G			
0.00000012	R	SINPQTIMLG(	Q	Oxidation (M)	0.00000002000000.0	
0.000000045	K	TNEQTVQEMI	Y	Oxidation (M)	0.000000002000000.0	
0.000000036	R	IFGAGGGLGH.	M			emPAI
0.00056	R	DLNEYVGFVK	S			
0.000045	K	GVDGLFESFAI	L			
0.0013	K	IGDHLQSADA(	E			
3.4E-09	R	VLESAFTALEG	Q			
5.6E-10	R	AESQQVSESS(	S			
0.0031	R	GQSAPLSSAPI	D			emPAI
0.0049	K	NSIGIRPGK	G			
0.000093	R	MSLLAEAQR	V	Oxidation (M)	0.200000000.0	
0.0021	K	AGHQTSAESW	A			
0.00000055	K	KAGHQTSAES	A			
0.000077	K	LESIYGSFEKPS	K			
6.6E-09	K	TMISDSDYTEI	W	Oxidation (M)	0.0200000000000000.0	
0.00015	K	IENVPEMPLV	T	Oxidation (M)	0.00000020000000000000.0	emPAI

0.000083	R	VVGSEFVQK	Y			
0.0024	R	GVLLYGPPGT(	T			
0.0041	K	AVANHTTAAF	V			
0.0028	K	KPDTDFEFYK	-			
0.000078	R	ELLKPSASVALI	H			
3.9E-09	K	ENAPAIIFIDEV	F			
2.6E-09	K	ASPALMDLST/	L	Oxidation (M)	0.0000020000000000000.0	emPAI
0.0003	K	ADLVNNLGTI/	S			
0.00041	R	ELISNSSDALDI	I			
0.0017	K	HFSVEGQLEFI	A			
0.00000027	K	GIVDSEDLPLN	E			
0.0056	K	SGDELTSLKDY	M			
1.2E-10	K	GIEVLYMVDA	E			
0.0034	K	GLAPLWGR	Q			
0.000025	K	IGMVGLFTR	G			
0.0033	K	FGFYEYFK	K			
0.00000017	K	FASFETIVEMI	Y			
0.00000048	K	VFVGLPTTGG	A			emPAI
0.00011	K	VGSSEAALLAI	L			
0.000046	K	GTVEIITPVELI	Q			
2.1E-14	R	IHSENSGNTAI	G	Methyl (DE)	0.0001000000000000000000000000.0	
0.00000039	R	ALLDVGLIR	T			
6.1E-14	K	DIVAQIVSASI/	A			emPAI
0.0000068	K	DAGVIAGLNV.	I			
0.000023	K	MKETAEAYLG	K	Oxidation (M)	0.20000000000.0	
0.00018	R	NALETYVYNM	N	Oxidation (M)	0.00000000020.0	
0.000055	R	ITPSWVGFTD!	L			
1.4E-10	R	VEIESLFDGVD	A			emPAI
0.00000043	R	GLCAIAQAESL	Y			
0.00000024	R	ELAEDGYSGVI	V			
4.2E-12	K	FVADGVFYAEI	E			emPAI
0.0049	K	NSIGIRPGK	G			
0.0021	K	AGHQTSAESV	A			
0.00000055	K	KAGHQTSAES	A			
0.000077	K	LESIYGSFEKPS	K			
2.1E-09	R	IINSDEVQSVV	D			
0.00015	K	IENVPEMPLV	T	Oxidation (M)	0.00000020000000000000.0	emPAI
5.9E-09	R	AGVDDLLGILK	N			
4.2E-09	K	VVGLVGELFSL	V			
6.6E-09	R	VISEEFDSIFLEI	R			emPAI
0.000000019	K	IADVLLLLEK	E			
0.00031	R	VSSNVYSVLLR	V			
0.000007	K	DLLSVYGYLQF	E			
1.8E-10	R	YNEVLAAILAF	I			emPAI
0.0004	R	ADGYILEGK	E			
0.000024	K	SAIVQVDAAPI	Q			
0.0000049	R	SLDSHIEDQFA	L			
0.000094	R	LDTGNYSWG!	K			
0.000000002	R	VLDVVYNASN	T			emPAI
0.00079	R	TDACFIR	V			
0.00023	R	PLDEDDIALLK	T			
0.00044	K	TYGLGPYSAPI	K			
0.000000098	R	FDDGVGGDNI	T			
8.2E-11	R	TMLEIVNQLD	G	Oxidation (M)	0.020000000000000.0	emPAI
0.00031	R	NIDQVLLEFK	E			
0.000039	K	LVYLYLINYAK	S			
1.7E-10	K	SQPDLAILAVI	D			
0.00022	K	MAPPLVTLLS/	N	Oxidation (M)	0.200000000000000000000.0	

0.00005	K	LLTALNECTEW	Y			emPAI
0.0035	R	SFSMEFLETK	D	Oxidation (M)	0.0002000000.0	
0.000000025	K	AEMTLDETLE/	Q	Oxidation (M)	0.00200000000000000000.0	
9.2E-10	R	SAADLVIESLE(	L			
0.00001	K	LADLVGVTLGI	G			
0.00000019	R	DIISILEDAIK	G			
0.000000067	K	VVAAGANPVL	G			
0.0000029	K	YEDLMAAGIID	V	Oxidation (M)	0.00002000000000.0	
0.009	K	GGYPLLIIAEDI	L			emPAI
0.00012	K	FVFNSLLPAICI	A			
1.1E-13	R	LLVDEAVLQE\	G			
1.8E-09	K	IPSFEAVDDFL'	V			
1.2E-10	K	VIELSDVILEVL	D			emPAI
0.00031	R	NIDQVLLEFK	E			
0.000039	K	LVYLYLINYAK	S			
1.7E-10	K	SQPDLAILAVN	D			
0.000059	R	GIPFLIELTAIV(	C			
0.00022	K	MAPPLVTLLS/	N	Oxidation (M)	0.200000000000000000000.0	emPAI
0.00015	R	VDSVFALR	S			
0.000082	K	FIQFVVDIFK	R			
6.3E-12	R	VINLILQYLSNS	S			
0.00074	K	DLDEIRPVLPQ	L			
0.00054	K	VCILGLTSLFSL	A			emPAI
0.0000018	K	SQDSEVGDGT	E			
0.00000036	K	INAINAATEAA	N			
0.00043	R	VAAAAGGTV(	Q			emPAI
0.0002	K	LVAMATPEDN	I	2 Oxidation (M	0.00020000020.0	
0.00000041	K	NSFVETLEGW	T			
0.00000057	R	DLFEGILQAGS	T			
0.000021	K	IGSSLIAQAAD'	I			emPAI
0.0044	K	GYEGVVTR	W			
0.00022	R	VLAHTQIR	K			
0.000035	R	HGSLGFLPR	K			
0.0013	R	QSLLTQTSR	L			
0.003	K	DEMIDIIGVTK	G	Oxidation (M)	0.00200000000.0	
0.0012	K	VDFAYSFFEK	Q			
0.000083	K	FIDTASIFGHGI	F			
0.0000029	K	VGTEAHTAMT	T	Oxidation (M)	0.00000000200000.0	
0.0078	R	SLNTVWAQHI	R			emPAI
0.0000046	R	IGDLELFR	T			
0.0013	R	LLLGEIPER	S			
0.000022	K	EIAALIDTGSYT	E			
0.0012	K	ALRPYFELTNA	I			
0.000067	K	GDEHDMEVD'	H	Oxidation (M)	0.000002000000000000000.0	
0.00000058	K	LTGSVLSSFLDI	L			emPAI
0.0000005	R	AILSSEIAVVDL	G			
7.5E-11	R	VAAFADTVNE	V			
0.0000041	K	LLLIGDSGVGK	S			
0.00004	K	LQIWDTAGQE	F			
0.0036	K	AFADELGIPFLI	N			
4.5E-11	R	FADDSYLDSYI:	I			emPAI
0.0000022	R	FIADAVASLPK	L			
0.00016	R	LLTLINDVYK	Y			
0.00024	K	AFVSSNLSLGD	Q			
0.000000065	K	YVDSVVGGQI,	F			
0.0007	K	LPPQVFDNLVI	T			emPAI
0.000051	K	IMFALTSIK	G			
0.0081	K	IPDWFLNR	Q			

3.7E-10	R	AGELSAAEIDN	Q	Oxidation (M) 0.00000000000020000000.0	
0.0001	K	HATFVPHTAG	Y		
0.0000035	R	VNQAIFLLTTG	E		
1.4E-10	K	TIAECLADELIN	G		emPAI
0.00062	K	VGIALALKP	-		
0.000062	K	SFFTVSGEVDS	A		
0.000000048	K	GSLFLGDVAT(	N		
0.000000016	K	VSTDSSLLTTLT	V		emPAI
0.00000018	K	VGEEVEILGLR	E		
9.4E-09	R	GSALSALQGTI	Q		
0.000029	R	HYAHVDCPGH	N		
0.0000024	R	MQADNTLPLA	R	Oxidation (M) 0.200000000000.0	
0.0042	K	FYSGFPVYCVF	I		
0.00027	K	YPYTGSLDCAI	T	Oxidation (M) 0.000000000020.0	
0.000022	K	AIESNDGKPLP	A		
0.00000033	R	IQLGQGSAASI	N	Oxidation (M) 0.00000000000000200.0	emPAI
0.00000003	K	ELLDSILDTIVK	I		
0.000000045	K	LILLASLSDSLE	L		emPAI
0.00011	K	LQQFQGAVDA	K		
0.000012	R	EGLVSDAIESFI	A		
0.000012	K	QVGYTPDYLFI	T		
0.0000048	K	FQSVPVQAGC	G		emPAI
2.8E-09	R	SIATLAITTLLK	T		
0.000018	R	SLMTFLSNILR	E		
0.00000025	R	ELTPAITVLQLI	P		emPAI
0.0012	R	WIVNLIR	T		
0.0000022	R	HLVFPIFEFLQI	Q		
0.0023	K	SLYHTEDAPQI	R	Oxidation (M) 0.000000000002000.0	
0.0093	R	VEDGNFSTVPI	L		
0.0000006	K	SLEEAAAPLVS	A		
0.00000011	K	LAIFTALAFSQI	L		
0.0048	R	EAILPSVVYIQK	I		
0.00000045	R	FLQSLELFEENI	K		
0.0028	R	SLYELDVLAED	K		
0.000098	K	LPETEVVHVIV	N		emPAI
0.0000036	K	FTSLPPLFENSI	E		
9.4E-09	R	QTAPDATYVY	T		
0.0000074	R	NRIPQSPQENI	L		emPAI
0.000013	R	IAGLEVLR	I		
0.00026	R	VVDWLASTFK	K		
0.00012	K	NQADSVVYQT	Q		
0.000000086	K	QFAAEEISAQV	K		emPAI
0.000051	K	ILQLLR	L		
0.000083	K	LLFIIR	I		
0.0001	K	EANNFLWPFC	A		
5.7E-10	R	IALTDNSIVDQ	H		emPAI
0.0012	K	IAELVENK	T		
0.0000052	K	ELLQAFIDFR	C		
3.3E-09	K	EATESYMSYAI	A		
0.0062	R	LDPLAEAVLLS	E		emPAI
0.00000012	K	LVVIVDVVDQI	A		
9.7E-11	R	LSLTDIVIDINR	V		emPAI
0.000063	K	ILLENAGAVFR	T		
9.6E-09	R	LLGLDTILNIVV	R		
0.00000067	R	APLVVAAIQTL	K		emPAI
0.0000053	R	GLLGTVGTIAR	E		
0.0014	K	SALAGDVTLPK	Y		
0.000088	R	ALWTGLGPNV	N		

0.000000086	R	YSGALNAYSTI	Q			emPAI
0.00014	R	TSISGHLR	N			
0.000013	R	SWNVDILVDT	Q			
0.000053	K	AIQTIDADIAQ	R			
0.00005	K	IDLLPEIVEAPC	A			emPAI
0.00039	R	TCYAQSSQIR	Q			
0.00000028	K	ATQGIYPLQN	K			
0.00004	K	LMDVHGDYS/	V	Oxidation (M)	0.0200000000000000.0	
0.0000042	K	VDRPADEMA\	-	Oxidation (M)	0.00000002000000000000.0	emPAI
0.00032	R	IIDLIEGR	S			
0.0047	K	TLAFGIPIIK	R			
0.00014	K	GWVTLQLIR	D			
0.00054	R	SVTGFLSDLYR	T			
0.0000033	R	TILSDLITVYAK	G			
0.000023	K	GTDALAAALA	S			emPAI
0.0012	K	TSLNGIPLLVL(	I			
0.00000002	K	NSTNIDQVID\	H			
0.000000028	K	EALTDEMGLT	E	Oxidation (M)	0.000000200000000.0	emPAI
0.0042	R	VLNPNAEVLN	S			
0.0021	K	GIDPPSLDLLA	E			
0.0000055	R	QLINSGPVIAS	A			
1.5E-09	K	LYEGLADQLTD	K			emPAI
0.0032	M	APVSALAK	Y			
0.0000068	K	LVFLGDQSVGI	T			
0.00004	R	LQLWDTAGQI	F			
0.000000017	R	DSSVAVIVYD\	Q			emPAI
0.0000041	K	LLLIGDSGVGK	S			
0.0034	K	GQALADEYGII	F			
0.00004	K	LQIWDTAGQE	F			
0.00000013	K	TNLNVEEVFFS	D			emPAI
0.0000011	K	NPVLTSFATGF	A			
4E-10	K	AVVSSPLGGEI	S			emPAI
0.00012	M	GIDLIAGGK	S			
0.000000061	R	APLGQNTVLLI	G			
0.00000026	K	AGGECLTFDQ	A			emPAI
0.0091	K	LGGAFAPK	P			
0.0044	K	LTIIEEAR	K			
0.00011	R	LGNVYTIGK	G			
0.004	R	ECLPLVLIIR	N			
0.00000019	K	FDVGNVVMV	N	Oxidation (M)	0.0000000200000.0	emPAI
6.3E-09	R	LQAQTEAVNL	T			
0.00000019	K	TQSNPENTVG	G	Oxidation (M)	0.00000000000002000.0	emPAI
0.0019	R	ESAIAQIIR	T			
0.000012	R	LGNLGTISQN\	V			
0.00042	R	TCGPLLDVVAI	E			
7.3E-09	K	VPYGQGAVEL	E			emPAI
0.0037	K	VLSMAPGLER	L	Oxidation (M)	0.0002000000.0	
0.0000023	R	SQDFLFISGTK	M			
0.000000022	K	NPVLLLNPLG(	A			
0.0028	R	NPVHNGHALL	R	Oxidation (M)	0.000000000020000.0	emPAI
0.0034	R	DGVLWLIDR	Y			
0.000000026	R	LSGLVNNLISI(	E			
0.000000074	R	LASFQNAPSVI	Q			emPAI
0.000004	K	EQIQAYVFDV\	A			
0.000029	K	ASADDAFYEL(	E			
0.0000005	K	EVMDLIMITQ	D			emPAI
4.2E-11	K	QSNILEDLATL	L			
0.00011	R	YVYQPIEALFLI	Q			emPAI

0.003	R	AITSAYYR	G			
0.005	K	VVLIGDSGVGI	S			
0.000096	K	STIGVEFATR	S			
0.000011	R	HLVAVQTEDA	S			
0.000000097	R	GAVGALLVYD	H			emPAI
0.0015	K	HLSEMVVSK	A	Oxidation (M)	0.000020000.0	
0.0052	K	NLSEIPDFK	M			
0.000018	K	IAFILEQVR	L			
0.00000019	K	DSNEILNSWA	L			
0.00013	K	EGDNMVEEAI	R	Oxidation (M)	0.00002000000000000000.0	emPAI
0.00043	R	LALPQIEDIVR	A			
2.5E-11	K	LAEEGISAEVI	S			
0.0066	R	IAGADVPMPY	L	Oxidation (M)	0.0000000200000000.0	emPAI
0.000015	K	AIILVTSDVSAF	G			
0.00019	K	TVAFLLPAIEA	S			
0.0012	K	VLVLDEADHLI	R	Oxidation (M)	0.00000000000002000.0	
0.00000075	K	SLIEDEAELSD	T			emPAI
0.0012	K	YSGTVQGLK	H			
0.0000098	R	TGNENAQLTP	L			
0.000000017	R	TESAAVSTIVN	E			emPAI
0.0018	R	LAAELAER	R			
0.00000065	K	VATELGLWQE	S			
0.0034	K	QDALQALHDL	R			
0.0000034	R	DRPDLSAPESL	F			emPAI
0.00066	K	KLEDIVPSSHN	V			
1.8E-11	R	VDYQLIDITED	D			emPAI
0.0027	K	LAVLQFYK	V			
0.000047	R	TLADYNIQK	E			
0.0000099	K	ESTLHLVLR	L			
0.0000014	K	TITLEVESSDTI	A			emPAI
0.000051	K	ILQLLR	L			
0.000083	K	LLFIIR	I			
0.00019	R	ENFINELIR	R			
0.0001	K	EANNFLWPFC	A			
0.00026	K	HGIICTEDLIHE	E			emPAI
0.00000038	K	NPAIIVGAGLF	T			
0.000000029	R	FASEVAGVQD	G			emPAI
0.00094	K	SDTSVFDVFGI	V			
0.0013	R	ALQFNTLPVVI	T			
0.00045	K	TATEPLNPSEL	M			
0.000003	R	GLAEDIENGV	K			
0.00041	K	SVETTLAELGV	L			emPAI
0.000059	K	TVQGLIEELQK	K			
0.0000037	K	VGAATETELEI	K			
0.00003	R	APLLIIAEDVTC	L			
6.1E-10	K	EIALQQALDQI	G			
0.0055	K	GELEGEMGD	L	3 Oxidation (N	0.00000020000202000.0	
0.0026	K	AIGVNTENLLL	S			emPAI
0.00005	R	LLILTDPR	T			
0.00032	R	HTPGTFTNQN	L	Oxidation (M)	0.000000000200000000.0	
0.000000076	K	EGALGNIPIIAF	F	Oxidation (M)	0.00000000000000000020.0	emPAI
0.00000052	K	GLVLLSQSLK	E			
0.00067	K	ETGGVEAASLI	V			
0.000023	R	AQGEAEAILAF	A			
0.0017	R	AQILESEGER	Q			emPAI
0.0000015	K	TPGPGAQSAL	A			
0.000046	K	VENVTLGPAV	E			
0.0000092	R	IEDVTPIPTDS	R			emPAI

0.0036	K	GSAITGPIGK	E		
1.3E-11	R	MSLGLPVAAT	N	Oxidation (M) 0.20000000000000000000.0	emPAI
0.00002	R	SALNALLFR	T		
3.5E-09	K	GLLLFIDEADA	N		emPAI
1.1E-09	K	LATEAAESAAS	T		
0.00034	K	SDQFKPLDVTI	G		emPAI
0.000069	K	SSLETVLTVLH	F		
1.8E-09	R	GLHHLVYEILD	V		emPAI
0.000066	K	TPSADYSVDR	Q		
0.0046	R	QLSNAATGPI	E		
0.0081	R	QIQAQFQTSG	Q		
0.00016	K	TVSSAITESFQI	G		
0.007	R	DRDANIQDVN	D		
0.000096	R	NLLALAAAGAI	V		emPAI
0.00061	K	AIEAVLPK	P		
0.00015	K	FYEILGVPK	T		
0.0000051	R	EIYDQYGEDAI	E		
0.00012	R	KGEDLFVEHTI	R		emPAI
0.00000066	K	GVLQILLDLR	F		
0.000000052	K	WLSDELSAILLI	D		emPAI
0.00000016	K	GTCVSASNCA	G		
0.0000015	R	FKGTCVSASN	G		emPAI
0.000013	R	LLPLVIGLLR	T		
1.5E-09	R	TLSSEAFVNLL	I		emPAI
0.000000011	R	LGALSLLLETAI	R		
0.00005	K	VLNFLVDLLSV	R		
0.0000053	R	VVSNFVAQAL	K		
0.000000036	R	SFQFVSDLVEC	L		emPAI
0.0046	K	VALVQIPSK	V		
0.00021	K	STYSGFELFR	I		
0.000000018	K	GSADEFSIGGF	W		emPAI
0.0000013	K	LGSQLDVLASI	Y		
0.00000027	R	CEEICLSGGLVI	Q		emPAI
0.000000046	K	SANGEDTQVN	F		
0.000011	K	NLPATAQVAS	Y		emPAI
0.000019	K	LLILALER	L		
0.00000015	R	TDVIQALGGVI	G		emPAI
0.00099	R	GLSVFISDVR	N		
6.5E-10	R	DLGVLTSSTSLI	C		emPAI
0.000071	R	DLEIPLVVFLK	L		
2.3E-09	R	VLAFANLVYSII	N		emPAI
0.000000011	R	VSAADFLGIFR	I		
0.00016	K	EAVEDEAAIDI	C		emPAI
0.00054	K	GVNVQVLLR	C		
0.00011	R	GLEEEIVTSAN	G		
0.000098	K	VTFLELYNEEIT	V		emPAI
0.004	K	SAGSMSLEVV	V	Oxidation (M) 0.00002000000.0	
0.00022	K	FATLVATNVA	G		
0.00000062	K	TLAFVLPILESL	S		emPAI
0.00000086	R	LLNTLLDLVQK	E		
0.00000069	R	SPEFISLFVDDI	L		emPAI
0.0027	K	IACYVTVR	G		
0.0024	K	AMQLLESGLK	V	Oxidation (M) 0.0200000000.0	
0.000000018	K	VLEQLSGQTP	A		emPAI
8.6E-10	K	LVAEVLLDEVA	S		
0.0056	R	APGTAMEFSL	F	Oxidation (M) 0.000002000000000.0	
0.0038	K	MVADTLLSDIT	N	Oxidation (M) 0.200000000000000000000000000000.0	emPAI
0.00000011	R	VAIGQVLLSVR	C		

0.000005	K	ENVSSEALEA/	I			emPAI
0.00000021	K	TVESALNGLIN	S			
0.000036	K	TEKPQLLEEDE	K			emPAI
1.2E-09	R	FGLLGLLSVIK	M			
0.00027	R	GGLGVSPILGN	M			emPAI
0.000000021	R	NFVETVELQIG	N			
0.00042	K	QLFQNVQMS\	K	Oxidation (M)	0.000000020000000000.0	emPAI
0.00067	K	AVLAVADLER	R			
4.7E-09	R	WVGGVELELI/	I			emPAI
0.0019	R	TTLNGISFK	N			
0.000063	R	ASGGFGSFVVI	A			
0.00051	K	DQIGSFFYFPSI	A			
0.00028	R	STIQVYPGAW	T			emPAI
0.00023	R	VVGVLLGSSSF	G			
0.0000018	R	SVIALHNLINN	L			
0.0054	K	VVVHPLVLLSI\	V			
0.00097	K	LRENDLDVHA	E			emPAI
0.0000013	R	ILALNASFFLK	T			
0.00063	R	TNVIPIIEDAR	H			
0.000058	K	LAAAILGGVDI	V			emPAI
0.00019	R	SITIAEVEPLVK	D			
0.0000013	R	AALTQLLLYYT	L			
0.0012	K	SNTSLFVEELL\	F			emPAI
0.0000011	K	AGALGDSVTIT	E			
0.000017	K	ITVTADGQFSK	R			emPAI
0.0027	R	IDDIVSGIK	K			
9.4E-09	K	IGDDFFSFIVD(	E			emPAI
0.0037	K	ALNECLNEITR	A			
0.00000001	R	IIEQAIFSELCK	M			emPAI
0.000000077	K	YLSPLVDATLLI	A			
0.0029	K	LDTVLNIVLGA	G			emPAI
0.00000041	R	TPILVATDVAA	G			
0.0002	R	MLDMGFEPQ	K	2 Oxidation (N	0.20020000000.0	emPAI
0.00017	R	HNTLLEEQR	H			
0.000064	R	ELLEQLLSTK	M			
0.00046	R	SFSVYNNPSA(	K			emPAI
0.00042	R	LLSLPDFYDSSI	I			
0.00000037	R	VLGLIQNDTEL	L			emPAI
0.00011	R	VQEFDVLDW\	Q			
0.0005	R	DILSIPVSAAAF	E	Oxidation (M)	0.000000000000000002000.0	
0.000091	K	SILLEIPSLAR	Q			
0.000049	K	DLSVTGAGFN	G			
0.0011	K	LLTPVYFQFFLI	L			emPAI
0.000073	K	DMTALDIPVV	D	Oxidation (M)	0.020000000000000000000.0	
0.00013	K	AKDMTALDIP\	D	Oxidation (M)	0.000200000000000000000000.0	
0.000011	R	SKVEGAIESR	G			
0.000011	K	SLPLQTLIDILK	Q			emPAI
0.000000056	K	AIGNFGLSLLR	Q			
0.0089	R	IAAPGISVWNI	G	Oxidation (M)	0.00000000000000020000000000000.0	emPAI
0.0025	K	IGLFGGAGVGI	T			
0.00000063	R	DVNEQDVLLF	F			emPAI
0.0014	R	VLIASDVWAR	G			
0.000014	K	LLVLDESDEMI	G	Oxidation (M)	0.0000000002000.0	emPAI
0.0021	K	LSIEDVEHLLM	S	Oxidation (M)	0.000000000020.0	
0.000024	K	SALLYLAYTSVI	L			emPAI
0.0012	K	VGDFEMNLK	R	Oxidation (M)	0.000002000.0	
0.00000068	K	EGQVIGYLHQ	L			emPAI
0.0017	R	ALLIDLEPR	V			

0.000014	R	DIIESLVDEYK	A			
0.0027	R	YPGYMNNDL\	C	Oxidation (M)	0.000020000000000000000.0	emPAI
0.005	R	YLEDVIAHK	Q			
0.0000016	K	SAQFVLDLLK	N			emPAI
0.00039	K	KLEIDDDQK	L			
0.00000081	R	GCIVSPDLSVL	K			emPAI
0.000021	K	FVFLLSTR	A			
0.000016	K	LALDALVIQQ(	L			emPAI
0.000005	R	VEPLVNMGQI	A	Oxidation (M)	0.000000200000.0	
0.00094	K	GQIYPEPGFEY	Q			emPAI
0.0022	R	WLMQFGGFR	E			
0.0000064	R	LLADDLFMAK	L	Oxidation (M)	0.0000000200.0	
0.0017	K	ESGIELESLPK	D			emPAI
0.0000012	K	EAAVSYLEGVI	L			
0.0021	K	LSIEDVEHLLM	S	Oxidation (M)	0.000000000020.0	emPAI
0.00063	R	TNVIPIIEDAR	H			
0.00045	R	ILALNASYFLK	S			
0.000058	K	LAAAILGGVDI	V			emPAI
0.0088	K	KLGGTVDDTH	G			
0.0014	K	SFAEALEVIPYT	N			
0.00017	K	VVSQYSTLLAP	D			
0.0038	R	NIVEQAAIR	D			
0.0000035	R	DVQEASVYEG	L			emPAI
0.00035	K	AGALGDSVSIT	E			
0.000017	K	ITVTADGQFSK	R			emPAI
0.0000014	K	EGLLQLVSDK	A			
0.0022	K	YAADEDAFFAI	L	Oxidation (M)	0.000000000000000020.0	emPAI
0.0057	R	ASSLPSHALR	S			
0.00047	K	SLLFFQGQR	S			
0.0014	R	ASSGLSDGSA/	F			
0.0084	R	DDYSHAEPAT	S			emPAI
0.00014	K	KGLTPSQIGVII	D			
0.0073	K	TTPQDVDESIC	F			
0.00016	K	AHGLAPEIPED	K			emPAI
0.0014	K	QSLPPGLSVQI	-			
0.000032	R	YLYTLCVFDQE	A			emPAI
0.0051	R	VYVAIDQK	T			
0.0000065	R	GFGFVNFVSR	E			emPAI
0.0063	R	VGVHHLDDSC	N			
0.0000046	R	NLIVFNLQNP(	R			emPAI
0.000023	K	VAVDGQSDSI	D			
0.00038	K	AISSVPIHLSIYS	V			emPAI
0.00023	K	LPTAVLSTSVK	A			
0.0087	K	FSATAGLGVIH	G			
0.0012	R	IVSLLSESYNPH	Y			emPAI
0.0037	K	EFVDIILSR	T			
0.000015	R	ITPSLAYLEQIR	Q			emPAI
0.00031	R	TPVLVATDVA/	G			
0.0002	R	MLDMGFEPQ	K	2 Oxidation (M	0.20020000000.0	emPAI
0.0000096	K	VTVVPSAAAL\	A			
0.0012	K	HNGNISFDDV	I			emPAI
0.0033	R	AAALNIVPTST	A			
0.000014	R	VVDLADIVANI	-			emPAI
0.003	R	AITSAYYR	G			
0.000096	K	STIGVEFATR	T			
0.0012	K	AFQTILGEIYHI	K			emPAI
0.000079	R	LLLPLLVSK	S			
0.00014	R	SYGFTDSQIST	K			emPAI

0.00005	R	VVCEATLSPEV	I			
0.00012	K	QALPGLVPLLL	Q			emPAI
0.00047	R	KLQGEIDR	V			
0.00021	K	SSLAASASQVF	S			emPAI
0.00036	R	IFEGEALLR	R			
0.0064	K	QLVNIPSFMVI	L	Oxidation (M)	0.00000000200.0	
0.0018	R	YGLLDESQNKI	R			emPAI
0.000063	K	ILLENAGAVFR	T			
0.0069	K	VFESVLFPIFDY	H			emPAI
0.00039	K	LAPSGFVLLR	D			
0.0087	K	FSATAGLGVIH	G			
0.0012	R	IVSLLSESYNPH	Y			emPAI
0.00018	K	ILQEEIGGVK	G			
0.00021	K	SFSSGGEDGYV	L			emPAI
0.00087	R	DIVELQLK	Q			
0.001	K	QLDCELVIR	K			
0.009	K	ESSASSPELATI	L			emPAI
0.0037	K	FIDAIAR	N			
0.0005	K	SLADADYYR	V			
0.003	R	YAPGIEILSVR	V			
0.0036	R	GGALLNIITEP	L			emPAI
0.002	K	TIDVLSAADSII	R			
0.00068	K	TIDVLSAADSII	H			emPAI
0.000025	K	AFQAVEQFLQ	Q			
0.0011	K	LLPLLASSLEFG	M			emPAI
0.0042	R	GDSVIIVLR	N			
0.000065	K	NNTQVLINCR	N			emPAI
0.005	R	VTYLVLDEADF	M			
0.0002	R	MLDMGFDPQ	K	Methyl (DE); 2	0.20020010000.0	emPAI
0.0074	R	WVLPDSYLDV	N			
0.00028	K	TLAQIVGSEDE	M			emPAI
0.00053	R	YGDFFLR	Q			
0.0023	K	LVDESVNSQLF	D			emPAI
0.00051	K	TVAFLLPSIEAV	A			
0.0012	K	VLVLDEADHLI	R	Oxidation (M)	0.0000000000002000.0	emPAI
0.0039	R	VIFAWLAYK	Q			
0.00072	R	AAEILFGLGFD	E			emPAI
0.0033	K	AVILLQGR	Y			
0.00031	K	EVATLDALQSH	D			emPAI
0.0077	K	GYIPLSTYLR	T			
0.00056	K	GPKPGFMVEG	G	2 Oxidation (M	0.000000200020000000000000000.0	emPAI
0.0062	K	LLTEAPK	F			
0.00046	R	MVSAHSSQQI	A	Oxidation (M)	0.2000000000000.0	emPAI
0.0067	K	DVIFVTTR	R			
0.0062	R	TLTSVHEAMLI	R	Oxidation (M)	0.0000000020000000000000.0	emPAI
0.0071	R	SQIQTEAVQV	T			emPAI
0.0062	K	LLTEAPK	F			
0.0044	R	MVAAHSSQQ	A	Oxidation (M)	0.2000000000000.0	emPAI
0.0084	R	KPSPTVIEK	L			
0.0068	K	DIVSSFLPEVIL	E			emPAI
pep_expect	pep_res_befo	pep_seq	pep_res_after	pep_var_mod	pep_var_mod_pos	
0.00000096	K	SSVCDIAPK	G			
0.0000019	R	YLTASAVFR	G			
0.0021	K	NMMCAADPR	H			
0.00000028	K	LAVNLIPFPR	L			
0.0025	R	FPGQLNSDLR	K			
0.0000021	R	VSEQFTAMFR	R	Oxidation (M)	0.0000000200.0	
0.0023	R	FPGQLNSDLRI	L			

0.00024	R	RVSEQFTAMF	R		
7.2E-09	R	MMMTFSVFP	V	2 Oxidation (N	0.202000000000.0
0.0000013	K	EVDEQMMNI(	N		
0.000000061	R	LHFFMVGFAP	G		
0.0000028	R	AVLMDLEPGT	S	2 Oxidation (N	0.000200000020000.0
0.00000061	K	NSSYFVEWIPN	S		
0.00000043	R	IDVYFNEASG(	A		
5.8E-10	R	EILHIQGGQC(	F		
3.9E-11	K	MASTFIGNSTS	R	2 Oxidation (N	0.20000000000000200.0
4.5E-15	K	GHYTEGAELID	K		
1.2E-12	K	GHYTEGAELID	E		
0.000063	-	MREILHIQGG(	F		
0.0000087	R	GSQQYSALSVI	N		
1.3E-09	K	LANPTFGDLNI	F	Oxidation (M)	0.000000000000000200000000.0
0.00000092	R	SGPFGQIFRPD	G		
0.0036	K	FWEVICDEHG	I		
0.000085	K	EAENSDCLQG	I	Oxidation (M)	0.000000000000000000000000020000000.0
0.0035	K	VSDTVVEPYN,	T	2 Methyl (DE)	0.00100010000000000000000002000000(emPAI
0.0000019	R	YLTASAVFR	G		
0.0021	K	NMMCAADPF	H		
0.00000028	K	LAVNLIPFPR	L		
0.0025	R	FPGQLNSDLR	K		
0.0000021	R	VSEQFTAMFR	R	Oxidation (M)	0.0000000200.0
0.0023	R	FPGQLNSDLRI	L		
0.00017	R	INVYFNEASG(	Y		
0.00024	R	RVSEQFTAMF	R		
7.2E-09	R	MMMTFSVFP	V	2 Oxidation (N	0.202000000000.0
0.000000061	R	LHFFMVGFAP	G		
0.0000028	R	AVLMDLEPGT	S	2 Oxidation (N	0.000200000020000.0
0.00000061	K	NSSYFVEWIPN	S		
5.8E-10	R	EILHIQGGQC(	F		
3.9E-11	K	MASTFIGNSTS	R	2 Oxidation (N	0.20000000000000200.0
4.5E-15	K	GHYTEGAELID	K		
1.2E-12	K	GHYTEGAELID	E		
0.000063	-	MREILHIQGG(	F		
0.0000087	R	GSQQYSALSVI	N		
1.3E-09	K	LANPTFGDLNI	F	Oxidation (M)	0.000000000000000200000000.0
0.00000092	R	SGPFGQIFRPD	G		
1.1E-09	K	FWEVICGEHG	I		
0.0000011	K	EAENCDCLQG	I	Oxidation (M)	0.000000000000000000000000020000000.0
0.0035	K	VSDTVVEPYN,	T	2 Methyl (DE)	0.00100010000000000000000002000000(emPAI
0.0021	K	NMMCAADPF	H		
0.00000028	K	LAVNLIPFPR	L		
0.0025	R	FPGQLNSDLR	K		
0.0000021	R	VSEQFTAMFR	R	Oxidation (M)	0.0000000200.0
0.0023	R	FPGQLNSDLRI	L		
0.0024	K	STVCDIPPTGL	M		
0.00000037	R	YTGDSDLQLEF	I		
0.00024	R	RVSEQFTAMF	R		
0.0000004	R	MMLTFSVFPS	V		
1.2E-09	R	INVYYNEASC(	F		
0.00000022	K	EVDEQMLNV(	N		
0.000000061	R	LHFFMVGFAP	G		
0.0000028	R	AVLMDLEPGT	S	2 Oxidation (N	0.000200000020000.0
0.00000061	K	NSSYFVEWIPN	S		
0.00000037	R	SLTVPELTQQI	N	Oxidation (M)	0.000000000020000.0
5.8E-10	R	EILHIQGGQC(	F		
0.00000011	K	FWEVVCAEH(	Y		

3.9E-11	K	MASTFIGNSTS	R	2 Oxidation (M	0.20000000000000200.0
4.5E-15	K	GHYTEGAELID	K		
1.2E-12	K	GHYTEGAELID	E		
0.000063	-	MREILHIQGG(	F		
0.000056	K	LTTPSFGDLNH	F		
0.000000011	R	SGPYGQTFRPI	G		
0.0000011	K	EAENCDCLQG	I	Oxidation (M)	0.00000000000000000000000020000000.0
0.0035	K	VSDTVVEPYN,	T	2 Methyl (DE)	0.00100010000000000000000002000000(emPAI
0.0021	K	NMMCAADPF	H		
0.00000028	K	LAVNLIPFPR	L		
0.0025	R	FPGQLNSDLR	K		
0.0000021	R	VSEQFTAMFR	R	Oxidation (M)	0.0000000200.0
0.0023	R	FPGQLNSDLRI	L		
0.0024	K	STVCDIPPTGL	M		
0.0000022	R	YVGDSELQLEF	V		
0.00024	R	RVSEQFTAMF	R		
0.000000024	R	VNVYYNEASC	Y		
7.2E-09	R	MMMTFSVFP	V	2 Oxidation (M	0.202000000000.0
0.00000022	K	EVDEQMLNV(	N		
0.000000061	R	LHFFMVGFAP	G		
0.000014	R	AVLMDLEPGT	S		
0.00000061	K	NSSYFVEWIPN	S		
0.0092	R	NLTVPELTQQI	N		
3.9E-10	K	FWEVVNLEHC	Y		
3.9E-11	K	MASTFIGNSTS	R	2 Oxidation (M	0.20000000000000200.0
4.5E-15	K	GHYTEGAELID	K		
1.2E-12	K	GHYTEGAELID	E		
0.000024	K	LSTPSFGDLNH	F		
9.1E-09	R	SGPYGQIFRPD	G		
0.0000011	K	EAENCDCLQG	I	Oxidation (M)	0.00000000000000000000000020000000.0
0.0035	K	VSDTVVEPYN,	T	2 Methyl (DE)	0.00100010000000000000000002000000(emPAI
0.0021	K	NMMCAADPF	H		
0.00000028	K	LAVNLIPFPR	L		
0.0025	R	FPGQLNSDLR	K		
0.0000021	R	VSEQFTAMFR	R	Oxidation (M)	0.0000000200.0
0.0023	R	FPGQLNSDLRI	L		
0.0024	K	STVCDIPPTGL	M		
0.000099	R	YQGENDLQLE	V		
0.00024	R	RVSEQFTAMF	R		
0.0000004	R	MMLTFSVFPS	V		
0.000000024	R	VNVYYNEASC	F		
0.00000022	K	EVDEQMINVC	N		
0.000000061	R	LHFFMVGFAP	G		
0.000014	R	AVLMDLEPGT	S		
0.00000061	K	NSSYFVEWIPN	S		
5.8E-10	R	EILHIQGGQC(	F		
3.9E-11	K	MASTFIGNSTS	R	2 Oxidation (M	0.20000000000000200.0
4.5E-15	K	GHYTEGAELID	K		
1.2E-12	K	GHYTEGAELID	E		
0.000063	-	MREILHIQGG(	F		
0.000056	K	LTTPSFGDLNH	F		
9.1E-09	R	SGPYGQIFRPD	G		
0.0000011	K	EAENCDCLQG	I	Oxidation (M)	0.00000000000000000000000020000000.0
0.0035	K	VSDTVVEPYN,	T	2 Methyl (DE)	0.00100010000000000000000002000000(emPAI
0.00053	R	LSVDYGKK	S		
0.0019	K	DVNAAVGTIK	T		
0.000014	K	YMACCLMYR	G	Oxidation (M)	0.000000200.0
0.0046	R	IDHKFDLMYAI	R		

1.6E-09	R	LVSQVISSLTAS	F			
0.00000053	R	TIQFVDWCPT	C			
0.0011	R	SLNIERPTYTNI	L			
0.00000004	R	AVFVDLEPTVI	T	Methyl (DE)	0.000000000000100.0	
4.3E-09	R	IHFMLSSYAPV	A			
4E-11	R	AVCMISNSTS\	I			
0.000015	K	CGINYQPPTV\	V			
0.00014	R	GHYTIGKEIVD	I			
4.1E-11	K	TVGGGDDAFN	H			
0.0097	R	AVFVDLEPTVI	Q			
0.000047	R	AFVHWYVGE(	E	Oxidation (M)	0.00000000002000000000.0	
0.000000015	R	QLFHPEQLISG	G			
1.9E-11	R	FDGALNVDVT	I			
8E-11	K	RAFVHWYVGI	E	Oxidation (M)	0.000000000002000000000.0	
0.0000027	K	AFHEQLSVAEI	C	2 Oxidation (M	0.0000000000000000000002200.0	
5.1E-09	K	LADNCTGLQG	L			
2.4E-11	R	KLADNCTGLQ	L			emPAI
0.00053	R	LSVDYGKK	S			
0.0019	K	DVNAAVGTIK	T			
0.000014	K	YMACCLMYR	G	Oxidation (M)	0.000000200.0	
0.0046	R	IDHKFDLMYAI	R			
1.6E-09	R	LVSQVISSLTAS	F			
0.00000053	R	TIQFVDWCPT	C			
0.0055	R	SLSIERPTYTNI	L			
0.00000004	R	AVFVDLEPTVI	T	Methyl (DE)	0.000000000000100.0	
4.3E-09	R	IHFMLSSYAPV	A			
4E-11	R	AVCMISNSTS\	I			
0.000015	K	CGINYQPPTV\	V			
0.00014	R	GHYTIGKEIVD	I			
4.1E-11	K	TVGGGDDAFN	H			
0.0097	R	AVFVDLEPTVI	Q			
0.000047	R	AFVHWYVGE(	E	Oxidation (M)	0.00000000002000000000.0	
0.000000015	R	QLFHPEQLISG	G			
1.9E-11	R	FDGALNVDVT	I			
8E-11	K	RAFVHWYVGI	E	Oxidation (M)	0.000000000002000000000.0	
0.0000027	K	AFHEQLSVAEI	C	2 Oxidation (M	0.0000000000000000000002200.0	
5.1E-09	K	LADNCTGLQG	L			
2.4E-11	R	KLADNCTGLQ	L			emPAI
0.0049	R	MLFDIQK	F			
0.00057	R	ELAQQIEK	V			
0.00000064	R	VLITTDLLAR	G			
0.000045	K	VHACVGGTSV	E			
0.0025	M	AGSAPEGTQF	Q			
3.9E-09	K	GLDVIQQAQS	T			
5.6E-09	K	GVAINFVTLDI	M			
5.9E-09	R	ILQAGVHVVV	V			
4.7E-10	R	KGVAINFVTLD	M			
5.7E-10	K	MFVLDEADEN	G	2 Oxidation (M	0.2000000002000.0	
4.3E-09	R	GIYAYGFEKPS	G			
0.0000079	K	FYNVVVEELPS	-			
2.2E-09	R	SRDHTVSATH	D			
7.9E-10	K	IQVGVFSATM	K	Oxidation (M)	0.0000000002000000000.0	
0.000000092	K	LETLCDLYETL/	R			
6.9E-12	R	GIDVQQVSLVI	I			
0.0077	K	TATFCSGVLQ(	E			emPAI
0.0049	R	MLFDIQK	F			
0.0018	R	ALGDYLGVK	V			
0.00057	R	ELAQQIEK	V			

0.0000042	K	GVAINFVTR	D			
0.00019	R	KGVAINFVTR	D			
0.00000064	R	VLITTDLLAR	G			
0.000045	K	VHACVGGTSV	E			
3.9E-09	K	GLDVIQQAQS	T			
5.9E-09	R	ILQAGVHVVV	V			
5.7E-10	K	MFVLDEADEN	G	2 Oxidation (N	0.2000000002000.0	
4.3E-09	R	GIYAYGFEKPS	G			
0.0000079	K	FYNVVVEELPS	-			
2.2E-09	R	SRDHTVSATH	D			
7.9E-10	K	IQVGVFSATM	K	Oxidation (M)	0.0000000002000000000.0	
0.000000092	K	LETLCDLYETL	R			
6.9E-12	R	GIDVQQVSLVI	I			
0.00000022	K	LNEVLEGQDE	G	Oxidation (M)	0.00000000000000000000000020000000	emPAI
0.00053	R	LSVDYGKK	S			
0.0019	K	DVNAAVGTIK	T			
0.000014	K	YMACCLMYR	G	Oxidation (M)	0.000000200.0	
0.0046	R	IDHKFDLMYAI	R			
1.5E-11	R	LISQIISSLTTSL	F			
0.00000055	R	TVQFVDWCPT	C			
0.00014	R	SLDIERPTYTNI	L			
0.00000004	R	AVFVDLEPTVI	T	Methyl (DE)	0.000000000000100.0	
0.00000057	R	IHFMLSSYAPV	A			
9.7E-11	R	AVCMISNNTA	I			
0.000015	K	CGINYQPPTV\	V			
0.0097	R	AVFVDLEPTVI	Q			
0.000047	R	AFVHWYVGE(	E	Oxidation (M)	0.00000000002000000000.0	
0.000000015	R	QLFHPEQLISG	G			
8E-11	K	RAFVHWYVGI	E	Oxidation (M)	0.000000000002000000000.0	
0.0022	K	AYHEQLSVPEI	C	2 Oxidation (N	0.0000000000000000000002200.0	
5.1E-09	K	LADNCTGLQG	L			
2.4E-11	R	KLADNCTGLQ	L			emPAI
0.00078	K	FEAIIYVLK	K			
0.000023	K	TVGAGVIGTIL	-			
6.6E-09	K	VGETVDLVGLI	E			
0.0000045	R	SYTVTGVEMF	I			
0.00000045	K	KYDEIDAAPEE	A			
5.4E-10	K	ILDEALAGDN\	G			
0.000026	R	HYAHVDCPGH	N			
2.1E-10	R	GITINTATVEYI	H			
0.0000013	K	IVVELIVPVACI	F			
2.7E-12	K	TTLTAALTMAI	K	Oxidation (M)	0.00000000200000000000.0	
2.1E-12	K	EDQVDDAELL	E			
8.3E-10	R	QTELPFLLAVE	G	Methyl (DE)	0.0010000000000000000.0	
0.0000054	R	SYTVTGVEMF	G			
0.0000039	R	ELLSSYEFNGD	V			emPAI
0.000029	R	VVQILAR	R			
0.00016	K	AEIANVLSR	G			
0.00063	R	LDEMIVFR	Q	Oxidation (M)	0.00020000.0	
0.000075	R	HAQLPEEAR	E			
0.0022	K	EIELQVTER	F			
0.000013	R	VLELSLEEAR	Q			
0.00000047	K	AIDLIDEAGSR	V			
0.000025	R	LDMSEFMER	H	Oxidation (M)	0.000000200.0	
0.00037	K	HIEKDPALER	R			
0.00016	R	SQDFEMAGSH	D			
0.0093	K	RVLELSLEEAR	Q			
0.0084	R	LRHAQLPEEAI	E			

0.0000012	K	LAEEGKLDPV\	Q		
0.000073	R	VLENLGADPSI	T		
0.000048	R	VVDEGFDPSY(	P		
0.0000042	R	GSGFVAVEIPF	A		
0.0000022	K	MPTLEEYGTN	L		
0.0000037	K	ALAAYYFGSEE	L		
2.3E-10	K	VPEPTVEEAIQ	E		
0.0032	K	AHPDVFNMN	L		
0.00000022	R	QLGHNYIGSEI	E		
0.0000038	R	YTDEALVAAA(	F		
1.8E-10	K	LIGSPPGYVGY	R		
0.000035	R	TVDFKNTLLIN	G		
0.006	R	FQPVKVPEPT\	E		
5.7E-09	R	LGHNFVGTEQ	V		
0.000058	R	RLGHNFVGTE	V		
0.000056	K	AENEAEEGGP	V		
0.0029	R	QSDEIILFIDEV	G		emPAI
0.00039	K	SSVCDIAPR	G		
0.0021	K	NMMCAADPF	H		
0.00000028	K	LAVNLIPFPR	L		
0.0025	R	FPGQLNSDLR	K		
0.0000021	R	VSEQFTAMFR	R	Oxidation (M) 0.0000000200.0	
0.0023	R	FPGQLNSDLRI	L		
0.0000054	R	YVGNSDLQLEI	V		
0.00024	R	RVSEQFTAMF	R		
0.0000004	R	MMLTFSVFPS	V		
0.000000024	R	VNVYYNEASC	Y		
0.00000022	K	EVDEQMINVC	N		
0.000000061	R	LHFFMVGFAP	G		
0.00000061	K	NSSYFVEWIPN	S		
9.6E-12	K	GHYTEGAELID	K		
0.0000013	K	GHYTEGAELID	E		
0.000056	K	LTTPSFGDLNH	F		
0.0000015	R	TGPYGQIFRPD	G		
0.0000011	K	EAENCDCLQG	I	Oxidation (M) 0.000000000000000000000000020000000.0	
0.0035	K	VSDTVVEPYN	T	2 Methyl (DE) 0.0010001000000000000000000020000000	emPAI
0.0000023	K	DAGVIAGLNV	I		
0.000097	R	VEIIANDQGNI	T		
0.00000062	R	FSDSSVQSDM	L		
0.0006	R	MVNHFVQEFI	R		
0.0025	R	FEELNMDLFR	K		
0.0000092	K	NALENYAYNN	N		
0.0011	K	ELESICNPIIAK	M		
0.000054	K	STVHDVVLVG	I		
0.0000003	R	TTPSYVAFTDS	L		
0.000083	R	ARFEELNMDL	K		
0.000000079	R	IINEPTAAAIAY	K		
0.00000036	K	NQVAMNPVN	R	Oxidation (M) 0.000020000000000.0	
6.9E-09	K	ATAGDTHLGG	M		
0.00088	K	NAVVTVPAYF	Q		
0.00011	R	IINEPTAAAIAY	A		
0.00000025	K	VQQLLQDFFN	S		
9.5E-13	K	SINPDEAVAY(	V		
2.5E-09	K	EQVFSTYSDN(	A		
0.002	R	TLSSTAQTTIEI	A		
0.000077	K	VQDLLLLDVTF	N		emPAI
0.0021	K	NMMCAADPF	H		
0.00000028	K	LAVNLIPFPR	L		

0.0025	R	FPGQLNSDLR	K			
0.0000021	R	VSEQFTAMFR	R	Oxidation (M)	0.0000000200.0	
0.0023	R	FPGQLNSDLRI	L			
0.00024	R	RVSEQFTAMF	R			
0.00000016	R	YNGDSADLQL	I			
0.0000004	R	MMLTFSVFPS	V			
0.000000061	R	LHFFMVGFAP	G			
0.0000028	R	AVLMDLEPGT	S	2 Oxidation (M	0.000200000020000.0	
0.00000061	K	NSSYFVEWIPI	S			
9.6E-12	K	GHYTEGAELID	K			
0.0000013	K	GHYTEGAELID	E			
9.1E-09	R	SGPYGQIFRPD	G			
0.0000011	K	EAENCDCLQG	I	Oxidation (M)	0.00000000000000000000000000020000000.0	
0.0035	K	VSDTVVEPYN	T	2 Methyl (DE)	0.0010001000000000000000000002000000	emPAI
0.0000023	K	DAGVIAGLNV	I			
0.000097	R	VEIIANDQGNI	T			
0.0006	R	MVNHFVQEFI	R			
0.00003	R	EIAEAYLGTTIK	N			
0.0000092	K	NALENYAYNM	N			
0.0011	K	ELESICNPIIAK	M			
0.000000071	K	VQQLLVDFFN	E			
0.00000014	K	NSIDDVVLVG	I			
0.0000003	R	TTPSYVAFTDS	L			
0.00028	R	ARFEELNIDLFI	K			
0.000000079	R	IINEPTAAAIAY	K			
6.9E-09	K	ATAGDTHLGG	M			
0.00000027	K	NQVAMNPINT	R	Oxidation (M)	0.000020000000000.0	
0.00088	K	NAVVTVPAYF	Q			
0.00011	R	IINEPTAAAIAY	A			
0.0014	R	FTDSSVQSDIK	S			
5.9E-12	K	SINPDEAVAYG	V			
2.5E-09	K	EQVFSTYSDN	A			
0.000043	K	VGAFSVLR	E			
0.002	R	TTSPAAFTR	A			
0.00032	K	IDLDLEVR	L			
0.00026	K	CLAGLIVSR	S			
0.0013	K	HVVDDGLELR	K			
0.0025	K	SCSENISLAVR	N			
0.000039	K	ALSSPVLAAVG	Y			
0.00000011	R	TNIQMIGALCI	A			
0.000022	K	QHLALLSLGEI	R			
0.00001	R	AELPSCLPVLV	M			
2.9E-09	K	MDVFNTFIDLI	Q			
0.000039	R	EYSLQALESFLI	C			
0.0041	R	AAVSALSTFAH	G			
1.2E-09	K	TVTCIASLASSL	A			
0.000022	R	EENVKMDVFN	Q	Oxidation (M)	0.00000200000000000.0	
1.4E-13	R	LSSIILQQLDDV	C			
0.00000015	R	ELVVVLPDCLA	A			
0.0044	R	FGTHLGNTVPY	C			emPAI
0.0019	R	VVQILGR	R			
0.00063	R	LDEMIVFR	Q	Oxidation (M)	0.00020000.0	
0.0022	K	EIELQVTER	F			
0.000013	R	VLELSLEEAR	Q			
0.00000047	K	AIDLIDEAGSR	V			
0.000025	R	LDMSEFMER	H	Oxidation (M)	0.000000200.0	
0.00037	K	HIEKDPALER	R			
0.0093	K	RVLELSLEEAR	Q			

0.0000012	K	LAEEGKLDPV\	Q		
0.000073	R	VLENLGADPSI	T		
0.000011	R	VVDEGYNPSY(	P		
0.0000042	R	GSGFVAVEIPF	A		
0.0000022	K	MPTLEEYGTN	L		
0.0000037	K	ALAAYYFGSEE	L		
0.0032	K	AHPDVFNMM	L		
0.00000022	R	QLGHNYIGSEI	E		
1.8E-10	K	LIGSPPGYVGY	R		
0.000035	R	TVDFKNTLLIM	G		
5.7E-09	R	LGHNFVGTEQ	V		
0.000058	R	RLGHNFVGTE	V		
0.0029	R	QSDEIILFIDEV	G		emPAI
0.00052	K	VTVMDTLSR	L		
0.0005	R	TCNYLTSAAR	Y		
0.0006	K	QNLELYVER	V		
0.0017	R	VQDPNPELQK	A		
0.00009	R	MLQYGEQNIR	R	Oxidation (M) 0.2000000000.0	
0.00003	R	NLAGEIAQEYT	R		
9.9E-10	R	FLPLGLGLLYL(	Q		
0.00000039	R	VGQAVDVVG(	T		
0.00000031	R	AVPLALGLLCI!	V		
6.3E-12	K	TITGFQTHSTP	A		
0.000000055	K	YLSDILSVLALT	E		
3.3E-09	R	QNLAATFVNA	L		
0.000015	K	YLSDILSVLALT	F		
0.0033	R	QSEEASIDDLM	H		
0.00026	R	FLPLGLGLLYL(	T		emPAI
0.0016	R	QTVAVGVIK	S		
0.0087	K	EVSSYLKK	V		
0.000000042	K	IGGIGTVPVGF	V		
0.0023	K	STTTGHLIYK	L		
0.000000086	R	EHALLAFTLGV	Q		
0.000046	K	YYCTVIDAPGH	D		
0.000033	R	YDEIIKEVSSYL	K		
0.000058	K	FHINIVVIGHV	S		
0.000000018	K	MTPTKPMVVI	F	Oxidation (M) 0.20000000000000000000.0	
0.0000004	R	VETGMIKPGM	S	Oxidation (M) 0.00002000000000000000000.0	
0.0000028	K	NMITGTSQAD	D	Oxidation (M) 0.02000000000000000000000000000.0	emPAI
0.000000067	K	DAGVIAGLNV	I		
0.000097	R	VEIIANDQGNI	T		
0.0006	R	MVNHFVQEFI	R		
0.0000092	K	NALENYAYNM	N		
0.0000003	R	TTPSYVAFTDS	L		
0.000000079	R	IINEPTAAAIAY	K		
0.00000036	K	NQVAMNPVN	R	Oxidation (M) 0.000020000000000.0	
6.9E-09	K	ATAGDTHLGG	M		
0.00088	K	NAVVTVPAYF	Q		
0.00011	R	IINEPTAAAIAY	A		
0.00000025	K	VQQLLQDFFN	S		
5.9E-12	K	SINPDEAVAY(	V		
0.0036	K	KVEDSIEEAIQ\	M	3 Methyl (DE) 0.000000010000010000010000000.0	emPAI
0.00083	K	TDGLFLK	C		
0.0045	R	SLNLTLR	K		
0.0084	R	SLNLTLRK	E		
0.0000028	R	VAEYAFLYAK	T		
0.0006	R	YDDVDLITIR	E		
0.00000045	K	TRYDDVDLITII	E		

5.3E-11	R	TADLGGSSTT	A		
0.0000041	R	TQSFLTWESLE	R		
1.7E-12	K	NLANPTALLLS	H	Oxidation (M) 0.00000000000002000.0	
0.00000009	R	ENTEGEYSGLE	G		
0.00041	K	ELNLYANVRP(	T		emPAI
0.000097	R	VEIIANDQGNI	T		
0.000006	K	DAGVISGLNVI	I		
0.0006	R	MVNHFVQEFI	R		
0.0025	R	FEELNMDLFR	K		
0.0000092	K	NALENYAYNM	N		
0.0000017	K	SSVHDVVLVG	I		
0.0000003	R	TTPSYVAFTDS	L		
0.000083	R	ARFEELNMDL	K		
0.00046	K	QFSAEEISSMV	M		
0.000000079	R	IINEPTAAAIAY	K		
0.000023	K	NQVAMNPTN	R	Oxidation (M) 0.000020000000000.0	
6.9E-09	K	ATAGDTHLGG	M		
0.00088	K	NAVVTVPAYF	Q		
0.00011	R	IINEPTAAAIAY	A		
0.00000025	K	VQQLLQDFFN	S		
5.9E-12	K	SINPDEAVAY(	V		
0.000048	K	TSLILALGK	Y		
0.0011	R	LIGVLEGVR	G		
0.00031	K	LALETFSVR	C		
0.00012	R	EGYYILAER	L		
0.000027	R	DVLLSQFSPVF	E		
0.000000018	K	SLFESLNSVGV	R		
0.00017	R	VFACQNPSTQ	K		
0.000065	R	TVGSTHLDSC(	T		
0.00084	K	LLAENLNCLLN	D		
1.2E-09	K	TATVAQILENV	D		
0.000042	R	SLLSFIEILIPIA(	A		
0.0027	R	FTEIWVPPITD	S		
0.0008	R	ALHFNTSLLGS	A		
2.1E-09	K	QLFDNITAMLI	T	Oxidation (M) 0.000000002000000000.0	
7.1E-10	K	ALYDGFSMFFI	I	Oxidation (M) 0.00000002000000000000.0	
0.0024	R	SYQQWLVESN	F		emPAI
0.000007	R	VLPLIIPILSK	G		
0.0016	R	TALCDSALEVR	E		
0.0000082	K	DLPAVMTFLIS	A		
0.0000055	R	AVSTCLSPLVL	Q		
0.000000013	R	SQLLSFMDQLI	T		
1.4E-10	K	SGPLPVDTFTF	I		
6.1E-11	R	NPDTISQISDLI	T		
0.000000037	K	VVIDVLSSIVSA	R		
0.0000011	R	IICRNPDTISQI	T		
0.000035	R	HALDTLLQTTF	G		emPAI
0.0057	K	GVLLYGPPGT(	T		
0.0000022	K	LAEGFNGADLI	N		
0.0000055	K	HGEIDYEAIVK	L		
0.00002	R	AIASNIDANFLI	V		
0.000027	R	NICTEAGMFA	A		
0.00000013	R	VVLDMTTLTIN	A		
0.00032	K	VVSSAIIDKYIG	L		
0.0000032	R	ESIELPLMNPE	V		
0.000000029	R	TLMELLNQLD	V	Oxidation (M) 0.00200000000000000.0	
0.0000076	K	SLQSVGQIIGE	L		
0.0000012	R	EVDPVVYNMI	E		

0.000016	K	VPLILGIWGG	G			
0.0011	K	SFQCELVMAK	M	Oxidation (M)	0.0000000200.0	
2.6E-09	R	GLAYDTSDDQ	G			
0.00012	K	MCCLFINDLD	M	Oxidation (M)	0.200000000000000.0	
0.000057	R	EGPPVFEQPEI	L			
1.8E-13	R	VPIICTGNDFS	D			
0.0000099	K	NFLTLPNIKVP	G			
1.1E-14	R	VQLAETYLSQ	G			
0.00008	R	AMAAVLLR	K			
0.00064	R	DDAYLWPR	L			
0.0011	R	LSLSTQSSLK	S			
0.00000056	R	SSAESLFNLAK	Q			
0.00045	R	LNVVIQLPNA	Q			
0.0000009	R	IAICIFDDVAE	E			
0.000038	R	VLPALAAAMD	V			
0.00000031	K	ASFLPFFDELS	D	Oxidation (M)	0.00000000000000002000.0	
5.1E-11	R	QLVDIVGSML	H			
0.0053	R	WAAINAIGQL	V			
0.0035	R	DSIDSSQVLPA	V			
0.003	R	TLTESLNNGN	F			
0.0000076	R	VQAHAASAVL	L			emPAI
0.00038	R	ILQIHSR	K			
0.0057	K	GVLLYGPPGT	T			
0.000029	K	RFDSEVSGDR	E			
0.000000025	R	STDDFNGAQL	A			
0.001	R	ACAAQTNATF	L			
0.00000063	K	LAGPQLVQMI	L			
4.3E-14	R	TMLELLNQLD	I	Methyl (DE)	0.00000000000000010.0	
0.00047	R	DATEVNHEDF	K			
0.000046	K	QLPYLVGNIVE	K	2 Methyl (DE)	0.0000000000000020011000000000000	emPAI
0.0000056	K	VQEIVSEIFGK	S			
0.00026	R	SSGGLSDDEIN	M			
0.00000043	R	NSADTTIYSVE	S			
0.0002	K	FSPSQIGANVL	M			
0.000000021	K	EVDEVLLVGGI	V			
0.00093	R	QAVTNPTNTII	R			
1.3E-11	K	IPAEIASEIETA	T			
0.00013	K	GVNPDEAVA	G			
0.0001	R	EKIPAEIASEIE	T			
0.00000062	R	GDVKDLLLLD	L			
0.0000021	K	ATNGDTFLGG	S			emPAI
0.0045	R	SLNLTLR	K			
0.0084	R	SLNLTLRK	E			
0.0000028	R	VAEYAFLYAK	T			
0.0006	R	YDDVDLITIR	E			
0.00000045	K	TRYDDVDLITII	E			
5.3E-11	R	TADLGGSSTT	A	Methyl (DE)	0.000000000001000.0	
1.1E-09	R	TNSFLTWDNL	N			
0.0000017	K	ATLFPGDGIG	Q			
0.00000002	K	QAEQIHSAIIN	Y			
0.00041	K	ELNLYANVRP	T			
0.0081	K	QVFTAADVVII	T			emPAI
0.000000053	R	TGGGGLDLSSI	R			
0.000014	R	ELLAQGVSQSI	Y			
0.0018	R	LQSLAFQLTEK	L			
0.000063	R	TMAQLGLCAF	A	Oxidation (M)	0.02000000000.0	
0.00000046	R	AMMDILVSLI	N			
0.00082	R	VRELLAQGVS	Y			

0.00019	R	FELVDQLTHLT	I			
0.00000077	K	AFEVLNSLEVV	L			
1.7E-10	K	CVLNMLTILDI	Y			
0.000000022	R	LRDEPMFLAL/	M	Oxidation (M)	0.00000200000000000000.0	emPAI
0.000031	R	SGFGSFGMR	S	Oxidation (M)	0.000000020.0	
0.0025	K	AVLEPAMEGR	D			
0.001	K	TLAFGIPIIDK	I			
0.00001	R	GSASMFEGIG!	S			
0.00017	R	GRNPLCLVLAI	E			
0.0000023	K	CEALHGDISQ!	E			
0.000078	K	TLAFGIPIIDKII	Y			
1.5E-09	K	LADGITTYSIIA	A			
0.000028	R	TLAGFRDGHFI	G			
0.00000097	R	ESAPSLDTICLY	Q	Oxidation (M)	0.00000000000000000000020.0	
0.0000013	R	RVGDSESVGG	A			
0.003	R	GLDVPNVDLII	T			emPAI
0.0014	R	IGINGFGR	I			
0.000054	R	VVDLIVHMSK	A			
0.0013	K	AGIALSDKFVK	L			
0.0048	R	AASFNIIPSSTC	A			
7.2E-11	R	VPTVDVSVVD	L			
0.000021	K	TLLFGEKPVTV	N			
0.00000065	K	LVSWYDNEW	V			
5.7E-10	R	FGIVEGLMTT\	T			
6.1E-10	K	GILGYTEDDV\	S			
0.0000012	K	VINDRFGIVEG	T			emPAI
0.000032	K	EIEDAVADLR	S			
0.0002	K	VQSIVAEIFGK	S			
0.0000014	R	SSGGLSEDDIC	M			
0.00015	R	QAVTNPTNTV	R			
0.000000021	K	EVDEVLLVGGI	V			
0.000000002	K	SQVFSTAADN	V			
0.00021	K	IPSEIAKEIEDA	S			
0.00013	K	GVNPDEAVAN	G			
0.00000001	K	ELLLLDVTPLSL	L			
0.0016	K	LIVWAPTR	E			
0.0041	K	LILDVEDFK	N			
0.000042	R	VANEIGFPVM	A			
0.000025	K	LLEEAPSPALT/	K			
0.00000031	R	DHGINFIGPNI	V			
1.1E-09	K	SEAAAAFGND	F			
0.001	R	ALNDTIITGVP	L			
0.000000053	K	NAGVPTVPGS	V			
0.00000042	R	HIEFQVLADKF	D			
0.0000048	R	IQVEHPVTEM	V	Oxidation (M)	0.00000000020000000000000.0	
0.000003	K	LADEAVCIGE/	G			emPAI
0.0024	R	FFAFGR	V			
0.000011	R	LWGENFFDPA	K			
4.7E-13	R	PMEEGLAEAII	I			
0.0000045	K	AYLPVVESFGF	A			
0.00027	K	NATLTNEKEVI	A			
3.8E-10	K	STLTDSLVAAA	M			
0.0000043	R	YRVENLYEGPI	N			
2.4E-09	R	DGNEYLINLID	I			
0.009	R	VMQTWLPAS	Y	Oxidation (M)	0.02000000000000000000000000000.0	emPAI
0.00028	R	GINDTYFGR	L			
0.0000018	R	FMVQNVVDLI	A			
0.0000039	K	SLLEEYFSIR	L			

0.0048	K	KINEIHSEAER	N			
0.000044	K	STWQGSGPTF	A			
2.7E-10	R	VLLNNCQEAFI	L			
2.7E-10	K	APNSFGEFLGE	V			
8.5E-11	R	SQPQPQPQA/	K			emPAI
0.000000045	K	SLGLSDNVSIA	D			
0.0035	R	MVNGLVDED(	V			
2.4E-09	R	AGQYGTVTSL'	D			
0.0066	K	HMFQDAVWV	N			
3.3E-15	R	TMVFANTVEA	A			
0.000014	R	GVDVPNVSHV	I			emPAI
0.0027	R	VVGSELIQK	Y			
0.0059	K	KFEPAAPPAR	V			
0.000045	K	AVANSTSATFL	V			
0.0016	K	AICTEAGLLALI	E			
0.0044	R	IESLDPALLRP(	I			
1.6E-11	R	TMLELLNQLD	G			
6.6E-11	K	APLESYADIGG	E			
0.00000026	K	VLSVVGILQDE	V			
1.5E-10	R	VADDLSPSIVF	R			emPAI
0.0019	R	NVVLDEFGSPI	V			
0.00000008	K	VGAATETELEI	K			
0.000063	R	GYISPQFVTNF	L			
6.3E-14	K	TNDSAGDGTT	E			
6.4E-11	R	AIELPNAMEN.	E			
0.00026	R	AALQAGIDKL/	G			
0.000000028	R	APLLIIAEDVT(	L			
0.00063	R	APLLIIAEDVT(	G			
0.00033	K	TTQLRAPLLIIA	L			emPAI
0.000012	K	AGFAGDDAPF	A			
0.0000069	R	HTGVMVGM(	D			
0.00038	R	GYMFTTTAER	E			
0.00025	R	AVFPSIVGRPR	H			
9.2E-09	K	NYELPDGQVI1	F			
8.9E-09	K	LAYVALDYEQI	S			
0.000085	R	VAPEEHPVLLT	A			
6.9E-09	R	TTGIVLDSGD(	L			
0.0058	K	YDVHGEMSR	V			
0.000009	R	HENVDIVVIR	E			
0.0002	R	HLQFPSFADR	L			
0.00000093	K	LADGLFLESCR	E			
0.000041	K	YAFEYAYLNNI	K			
7.4E-09	K	TPVGGGVSSLI	K			
0.000000019	K	ANPVALLLSSA	H	Oxidation (M)	0.000000000002000.0	
0.008	R	TKDLGGTSTT(	L			emPAI
0.0017	K	KPDVDIEK	I			
0.0012	K	LVSGLIPDAGT	A			
0.000098	K	IPLTLIYDDIK	S			
0.00000019	K	DFGSALWDM	G			
0.0049	K	GNIDVDTPFG.	L	Oxidation (M)	0.0000000000020.0	
0.00000016	R	VVHQEGDVEI	S			
0.000004	K	FSLEETVAILH\	L			
0.0000013	R	IKVDLIVDVPV	L			
0.0000036	K	STYNDINPGM	I			
0.0025	K	AVLEPAMEGR	D			
0.0000028	R	GSASMFEGV(	S	Oxidation (M)	0.000020000000.0	
0.001	K	TLAFGIPIIDK	I			
0.00035	R	FNELPSIAVER	G			

0.000019	K	CEALHGDISQ	E			
0.0052	K	KGSAILIHGQD	A	Methyl (DE)	0.00000000001000.0	
0.000078	K	TLAFGIPIIDKI	F			
1.1E-10	R	SSGFGSFGSG	S			
0.000032	K	LADGITMYSIA	A			
0.000052	R	TLAGFRDGNF	G			
0.00000097	R	ESAPSLDTICL	E	Oxidation (M)	0.00000000000000000000020.0	emPAI
0.00042	K	LAIVNVGR	Q			
0.0000052	R	AVAVVVDPIQ	G			
0.0012	K	AVQEEDELSP	L			
0.000000072	R	SINPQTIMLG	Q	Oxidation (M)	0.00000002000000.0	
7.7E-10	K	TNEQTVQEMI	Y			
0.000074	R	AGVPMEVM	V			
0.00000021	R	IFGAGGGLGH	M			
0.00019	R	SGAVASAVM	K			
0.0035	K	ISWVPDPK	T			
0.0052	R	SGAVASAVM	K			
6.1E-09	R	GYAATAAQGS	S			
6.5E-11	K	TGYYRPETGS	A	Methyl (DE)	0.0000001000000000000.0	
0.000091	K	LINSTSEEIR	S			
0.00024	K	VSSYLVDVVLP	F			
1.5E-10	K	SGFEAATLIGLI	L			
6.2E-10	R	TNALNSAVLGI	G			
7E-14	K	VSSDFVDAAL	C			
0.0033	K	HLPHLWDLCL	S			emPAI
0.0057	K	GVLLYGPPGT	T			
0.00028	K	AVCTEAGMF	E			
0.00011	R	IDILDQALLRP	I			
3.7E-11	R	TMLELLNQLD	I			
8.2E-10	R	EHAPSIIFMDE	M			
0.000085	R	ILLDDGTSNIL	S	Oxidation (M)	0.0000000000020.0	
9E-11	R	LSGLVNNLISI	E			
1.3E-11	R	LASFQNAPSV	Q			
0.00000032	K	TDAASAFLASL	K			emPAI
0.00093	R	FMDNLFLR	N			
0.00081	R	NPASVAQFLR	S			
6.7E-09	R	GGLVSILNLGL	R			
0.0009	K	GSSLQCLVNVI	S			
0.00000003	R	NCALEVLFDLL	G			
2.8E-09	K	VVTQAAPSSI	T			
0.0022	R	ILSLELLQGML	N			
0.0034	K	TTSGLADDAS	L			
0.000088	K	LPSESVVEFFT	Q			
0.0083	R	AELTNFTFQN	N			emPAI
0.00033	K	SSLDAFK	Q			
0.0035	K	LQLIVFGK	K			
0.00061	R	AVAGAGVLSG	L			
0.000059	R	QFDGLVDVYR	K			
0.000043	K	LLIQNQDEMI	A	Oxidation (M)	0.00000000200.0	
0.0053	R	TIKDEGFGSL	G			
0.00000011	K	GFTNFALDFL	T			
0.000094	R	AVAGAGVLSG	K			emPAI
0.0072	K	ASTFNDSLR	S			
0.00002	R	EAGSGAPSLLE	A			
0.000000013	R	LEGASEESLLS	V			
3.6E-09	K	STGGWGWG	A			
6.8E-12	K	VFDDSVESFTS	G			
0.00029	R	GKAETMAGY	G			emPAI

4.5E-10	K	AADQGLVILQ!	R			
0.00000054	K	AADQGLVILQ!	S			
0.000000002	R	EDGEPVGYNE	K			
6.3E-12	K	SSEVEALISEITI	L			emPAI
0.000000057	K	VGEEVEILGLR	E			
8.3E-10	R	GSALSALQGTI	Q			
0.000032	K	LMDAVDEYIPI	V			
5.1E-10	K	ILDNGQAGDN	G			
0.000026	R	HYAHVDCPGH	N			
0.000067	K	DAGVIAGLNV.	I			
0.0071	R	NALETYVYNM	N			
0.0000016	R	ITPSWVGFTD!	L			
0.0022	K	NGHVEIIAND(	I			
0.000000079	R	IINEPTAAAIAY	K			
0.00011	R	IINEPTAAAIAY	G			
2.3E-09	R	VEIESLFDGVD	A			
0.0005	R	FVEFLTR	E			
0.0087	R	YKVAVDAEEG	R			
0.000026	R	SLQLEATTQFR	K			
0.0041	R	NATWTLSNFC	G			
0.00000013	R	SPPIEEVIDAG'	F			
6.4E-09	R	IITVCLEGLENI	V			
0.00000024	R	EQAVWALGN'	C			
0.00002	R	LVELLQHQSPS	S			
0.0016	R	CRDLVLGQGA	L			
0.0042	K	VVIEHGAVPIF	C			emPAI
0.0055	R	SGAYIVR	Q			
0.0018	K	SIVASGLAR	R			
0.00025	K	TAAYGHFGR	D			
0.00001	K	ANVDYEQIVR	K			
0.00000022	R	FVIGGPHGDA	K			
1.3E-10	K	TNMVMVFGE	A			
5E-10	R	EIGFVSADVGL	V			
0.0034	K	VLVNIEQQSPI	K			
0.0025	K	FSLGLAEPK	L			
0.002	R	SFLELNLPK	V			
0.0016	K	SQLGLAHSYSF	A			
0.000004	K	IVNDNYLYAR	V			
0.0000013	K	CPSSTLQILGAI	A			
9.2E-10	K	IPCQSNEFVLE	G			
0.000032	K	MSDIAPNLAA	L			
0.0000034	R	VVQLTAFHPFI	S	Oxidation (M)	0.00000000000000000000000000020000(	emPAI
0.0087	R	YKVAVDAEEG	R			
0.0041	R	NATWTLSNFC	G			
1.7E-10	R	SPPIEEVISAG\	F			
6.4E-09	R	IITVCLEGLENI	V			
0.00000024	R	EQAVWALGN'	C			
0.0000046	K	LVELLLHHSPS'	T			
0.0019	R	CRDLVLGCGA	L			emPAI
0.00039	K	APGILER	K			
0.0017	K	AVDSLVPIGR	G			
0.0000034	R	AAELTNLFESR	I			
0.0000041	K	TTIAIDTILNQK	Q			
0.002	R	IRNFYANFQVI	V			
2.9E-13	R	EVAAFAQFGSI	G			
0.00055	R	VLATDEWLR	V			
0.00091	R	HSVIDCFER	A			
0.000000079	K	SANGEDTQVN	F			

0.000000046	K	NLPATAQVAS	Y			
4.5E-10	R	VEGCDGVYAL	R			
0.000016	R	NFFLFTPLLPS\	S			emPAI
0.000012	K	AGFAGDDAPF	A			
0.0056	R	GYSFTTTAER	E			
0.0000069	R	HTGVMVGM(	D			
0.00025	R	AVFPSIVGRPR	H			
0.0000097	K	NYELPDGQVI	F			
6.9E-09	R	TTGIVLDSGD(	L			
0.0021	R	FELLAR	L			
0.00034	R	TDACFIR	V			
0.0002	R	SVCTEAGMYA	A			
0.000000099	R	FDDGVGGDNI	T			
3.7E-11	R	TMLEIVNQLD	G	Oxidation (M)	0.020000000000000.0	emPAI
0.00019	R	GTGGSAVFIAF	N			
0.0011	R	GWSESSSPNV	G			
0.000063	R	LVLGELQTPFV	Y			
0.00027	R	AFSSSPVVPLA	G			
0.00000041	R	SLLDTNPTIES(	T			
0.0000025	R	GPPALVFDFS(	L			
5.8E-10	K	SSLAAEQAAA(	L	Oxidation (M)	0.000000000000000020.0	
0.00079	K	ILENAGHLPLA	L			emPAI
0.005	R	SISCWTLSR	F			
0.0032	K	YLIQESGNPK	G			
0.0000042	K	ALVENSAITLG	L			
0.0000076	R	QSAFALMGDL	V			
0.0028	R	LLQFFQSPHAS	K			
0.0038	K	VVSSLGLILQH	A			
6.1E-10	R	LLDFLEIASQQ	E			
0.00079	K	CSAAAIDVLSN	N	Oxidation (M)	0.000000000000000000000200000.0	
0.008	K	ICEDIPHVLDTI	L			emPAI
0.00032	K	VVSAMVNND	S	Oxidation (M)	0.00002000000.0	
0.0000081	K	IPGVISASSFD(	I			
0.00076	K	IGIYNIEGCSR	Y			
0.00000053	R	LFNETSEALG(	A			
0.0023	R	DLPLVGEIPSSI	F			
6.6E-09	K	VLDSGGLSPEL	D			
0.00000072	K	GTSSILSEVFLF	T			emPAI
0.00000054	R	IVNDGVTVAR	E			
0.0000018	K	LADLVGVTLGI	G			
0.0000003	R	DLVGVLEDAIF	G			
0.0000055	K	VVAAGANPVL	G			
0.000000005	K	AAVEEGIVVG(	L			
0.000069	R	GGYPILIIAEDII	L			emPAI
0.000009	R	HENVDIVVIR	E			
0.0002	R	HLQFPSFADR	L			
0.000041	K	YAFEYAYLNNI	K			
0.00000018	K	TPVGGGVSSLI	K			
0.0000027	K	ELDLFASLVNC	H			
0.00000001	R	TEDLGGNSTT(	-			emPAI
0.0048	R	VDMLMVER	T			
0.0047	K	LEGLTGEGQR	A			
0.00000007	K	LLEIDPNGAVS	K	2 Oxidation (I	0.000000000000000220.0	
0.00000021	R	LEVYTADDYAI	W			
0.000015	R	TVQHLIGSGM	A			
0.00084	K	SSGNDASIALR	N			
0.000037	K	IPSFEAVDDFL'	V			
9.7E-10	K	VIELSDVILEVL	D			

0.000000008	K	VIELSDVILEVL	C		emPAI
0.00041	R	GNLPGAENLV	F		
0.000064	R	ADDTTQFLEVI	A		
0.000000073	R	EGLVSDAIESFI	A		
0.000033	R	TANLANNQIIN	C		
0.0017	R	GCFNELISLME	A		
0.00065	K	QVGYTPDYMI	T		
0.00000023	K	FQSVPVQAGC	G		emPAI
0.0016	K	IGLFGGAGVGI	T		
0.0063	R	TIAMDGTEGL'	G	Oxidation (M) 0.000200000000.0	
0.0041	K	VLNTGAPITVF	A		
0.00078	R	VGLTGLTVAEY	D		
6.8E-09	K	TVLIMELINNV	A		
0.00000052	R	FTQANSEVSAI	I		
0.0000026	R	DAEGQDVLLF	F		
0.0011	R	DAPALVDLAT(	G		emPAI
0.00016	K	IMFALTSIK	G		
8.3E-11	R	AGELSAAEIDN	Q		
0.0071	R	VLNPNAEVLN	S		
0.00000001	R	AQLGVEAFAN	T		
6.1E-10	R	QLINSGPVIAS(	A		
0.000025	R	TKLYEGLADQI	K		emPAI
0.000012	K	AGFAGDDAPF	A		
0.00038	R	GYMFTTTAER	E		
9.2E-09	K	NYELPDGQVIT	F		
0.0062	R	IAPEEHPVLLTI	A		
0.00000034	R	TTGIVLDSGDC	L		emPAI
0.00004	R	QAVDISPLR	R		
0.0039	K	HATFVPHTAG	Y		
0.0000001	R	VNQAIFLLTTG	E		
0.0017	R	RVNQAIFLLTT	E		
7.1E-10	K	TIAECLADELIN	G		emPAI
0.00041	R	GNLPGAENLV	F		
0.000000073	R	EGLVSDAIESFI	A		
0.000033	R	TANLANNQIIN	C		
0.0017	R	GCFNELISLME	A		
0.000048	K	QVGYTPDYLFI	T		
0.00000023	K	FQSVPVQAGC	G		emPAI
0.0000031	R	HLVFPIFEFLQI	Q		
0.00081	K	SLYHTEDAPQI	R	Oxidation (M) 0.000000000002000.0	
1.6E-09	K	SLEEAAAPLVS	A		emPAI
0.00000054	R	IVNDGVTVAR	E		
0.0000018	K	LADLVGVTLGI	G		
0.0000039	R	DIISILEDAIK	G		
0.0000055	K	VVAAGANPVL	G		
0.000000005	K	AAVEEGIVVG(	L		emPAI
0.00053	K	VLAALQESR	R		
0.000062	R	ILEFIAVGR	L		
0.0042	K	ESAQIAHTVAF	K		
0.000000054	R	LADFGAAICG/	H		
3.6E-10	K	DGPSAGCTMI	K		
0.0035	K	LQLIVFGK	K		
0.0051	K	SDGIAGLYR	G		
0.00024	K	SSFDAFSQIVK	K		
0.000043	K	LLIQNQDEML	A	Oxidation (M) 0.00000000200.0	
0.0012	R	TIRDEGIGSLW	G		
0.00000022	K	GFTNFAIDFMI	T		
0.000053	R	AVAGAGVLAC	K		emPAI

0.000000002	R	VQIASLIGLLIR	H			
0.000042	R	SFVNLIESLLIKI	I			
0.0014	K	NLFPSLLSIIEQ	G			
0.0059	K	TNAHLFPVVLI	N			
5.2E-12	R	SASGWQVSN/	K			emPAI
0.00047	K	SNADALWAR	I			
0.0044	K	ELAAIGPALAR	S			
0.000065	K	TPSADYSVDR	Q			
0.0000079	K	TVSSAITESFQI	G			
0.0000024	R	NLDVSSVEEISI	S			
0.0021	R	SSVESSVIPSFE	A			
0.00046	R	DRDANIQDVN	D			
0.00017	R	LQGEIQPQLE\	Y			
0.00011	R	AEEAFFNQVI/	N			emPAI
0.0000065	R	LVELANEFSR	S			
0.000023	K	TVAFLLPAIEA\	S			
0.000034	R	QTFLFSATVPE	Q			
1.1E-10	K	SLIEDEAELSD\	T			
0.0072	K	VLVLDEADHLI	D	Oxidation (M)	0.00000000000020000.0	emPAI
0.00063	K	SLLTLGR	V			
0.0059	K	QVTDVSSLVR	S			
0.00045	K	SAPCGGLPAE/	A			
0.00000076	K	SDTDKLIADLT	R			
0.00004	R	GLEEEIVTSAN	G			
8.6E-09	K	VTFLELYNEEIT	V			emPAI
0.00000067	R	SSIFDANAGIG	S			
5.7E-10	R	VPTSNVSVVD	L			
0.000000056	K	GILGYTDEDV\	S			
0.0069	K	FLVLAPTR	E			
0.0036	R	IIDLIEGR	S			
0.000026	K	IFLIADDR	I			
0.0072	R	GVDVVVGTP(	I			
0.00045	R	SVTGFLSDLYR	T			
0.000000093	R	TILSDLITVYAK	G			
0.0015	R	IQGAVFDLPEE	E			
0.00012	K	GTDALAAALA	S			
0.0000059	R	DVGCHFEFISP	F			emPAI
0.0053	K	VVDSLYVR	S			
0.00014	K	IPGTTDLQITL(	E			
2.7E-12	R	HLSSLYDTLLE(	L			
0.000023	R	VEIAHIAELIGL	K			emPAI
0.00045	K	AVLAVADLER	R			
0.00000094	R	IAEGYEMASR	V			
7.5E-10	R	WVGGVELELI/	I			
0.00000038	R	SQDYEIGDGT	Q			emPAI
0.000000019	K	ILSAFVETEDV(	E			
0.0000011	K	TSAADVLQLLT	M			
0.000000046	K	QELLAELYSTEI	G			emPAI
0.0057	R	GVLLYGPPGT(	T			
0.00041	K	AVANHTTAAF	V			
0.0002	R	ELLKPSASVALI	H			
5.5E-11	K	ENAPAIIFIDEV	F			
0.0032	R	QLEFTDIQEEY	N			
0.000088	R	LAKENAPAIIFI	F			emPAI
0.0096	R	FNIFSGCPSGR	T			
2.1E-14	K	SQDSEVGDGT	E			
0.000013	R	VAAAAGGTV(	Q			emPAI
0.0066	K	NQQVSASALR	H			

0.000058	K	SQIVQDELAR	L			
3.7E-10	R	IALVDTLASQIF	S			
0.0000013	K	SAVLNSLIGHP	A			emPAI
0.0012	R	DLNEYVGFVK	S			
0.00000073	K	GVDGLFESFAF	L			
0.0029	K	IGDHLQSADA	E			
0.000000022	R	GLEVAVANLQ	L			
0.0096	K	AIFTCLLDQVS	A			
0.00053	R	GQSAPLSSAPF	D			emPAI
0.0047	K	LLQQLDEQSR	A	Methyl (DE)	0.0000010000.0	
0.0000002	K	MGYGSAAVEL	Y	Oxidation (M)	0.2000000000000.0	
0.000088	K	LSQVELLVIDE	S			
7.9E-11	K	SLLGPYLVFLSS	S			emPAI
0.00015	K	DMTALDIPVV	D	Oxidation (M)	0.0200000000000000000.0	
0.00000054	K	AKDMTALDIP	D	Oxidation (M)	0.00020000000000000000.0	
0.00018	K	VSVTNPYSHSI	E			emPAI
0.00055	R	LGYVGVVNR	S			
0.0014	K	SSVLEALVGR	D			
0.00000015	K	VPVGDQPSDII	I			
0.000073	K	SSVLEALVGRD	G			
0.00084	R	SALFVPDVPFE	R			emPAI
0.0023	R	HAAAVNFK	N			
0.00055	R	LLQAFLQK	A			
0.0021	K	QFLVASVSR	L			
0.00017	K	GVWSALFTR	V			
0.000000087	R	IQSQLSEALTV	H			
0.00000002	R	ALSDAADQAN	L			emPAI
0.00043	K	IAELLR	Y			
0.00072	K	ADLVNNLGTIA	S			
0.0007	R	ELISNSSDALDI	I			
0.0019	K	HFSVEGQLEFK	A			
9.6E-10	K	GIVDSEDLPLN	E			
0.00011	K	SGDELTSLKDY	M			
0.0018	K	FYEILGVPK	S			
0.00000027	K	EGMGGGGGG	Q	Oxidation (M)	0.0020000000000000000000000000000.0	
0.000013	R	KGEDLFVEHTI	S			emPAI
0.0000014	R	LQAQTEAVNL	T			
7.6E-09	K	TQSNPENTVG	G			emPAI
1.9E-10	K	SQPDLAILAVN	D			
0.0000023	R	GIPFLIELTAIV	C			
0.000001	K	MAPPLVTLLSA	N	Oxidation (M)	0.2000000000000000000.0	emPAI
0.0000002	R	LADTYGSGELF	L			
0.000077	R	LQADDMDELA	L	Oxidation (M)	0.00000200000.0	
0.0000081	R	FGFNLLVGGFF	R			
0.00000097	R	VTELVPLVAEII	E			
0.0032	R	VTELVPLVAEII	E			emPAI
0.0000027	K	TSSAVSLILR	G			
0.000012	R	DSYLLNGYALN	A			
0.0000059	R	EQLAIAEFADA	V			
0.00000052	R	TLESNTVVAG	E			emPAI
0.0074	R	VVVDGNVITSI	A			
0.00000023	K	LAATCATAVES	V			
0.000000052	K	LVAEVLLDEVA	S			
0.0044	K	SFDLIVLPGGLI	F			
0.0000014	K	TVLIPIAHGTEI	R			emPAI
0.0014	K	IVEAINVAAK	D			
0.00011	K	GLVLLSQSLK	E			
0.000047	K	SSVILASEAAK	M			

0.0000046	R	AQGEAEAILAF	A			
0.00017	R	AQILESEGER	Q			
0.0083	R	QSHINIADGKK	S			
0.0000017	R	VAEQYITAFGN	E			emPAI
0.001	R	ILGQPSLIR	E			
0.000011	R	SALNALLFR	T			
0.000011	R	IATEEQIQAQC	E			
3.9E-10	K	GLLLFIDEADA	N			emPAI
0.00019	R	GTGGSAVFIAF	N			
0.0011	R	GWSESSSPNV	S			
0.000063	R	LVLGELQTPFV	Y			
0.0028	R	LATELGDNVPS	T			
5.8E-10	K	SSLAAEQAAA(	L	Oxidation (M)	0.000000000000000020.0	emPAI
0.00013	R	SALLSELVSK	G			
0.0046	K	EIFQAQVISR	R			
0.000081	R	EEAAPLIEAAY(	R			
0.00015	K	VATELGLWQE	S			
0.0000033	R	QTTEVSGPSAI	R			
0.0045	R	QTTEVSGPSAI	S			
0.00056	R	DRPDLSAPESL	F			emPAI
0.001	K	SEVLDFIR	S			
0.00000014	K	IADAEENLGES	E			
0.000015	K	AFGVSVDFID(	F			
0.00049	R	SHGMAPLYET	A			
0.0066	R	GVEDLADAVK	V			
0.0000053	K	IGVQIIQNALK	T			
0.00000079	K	SVAAGMNAM	R	2 Oxidation (M	0.000002002000.0	
3.6E-09	K	TLFNELEVVEG	I			emPAI
0.000053	R	VIIDSGAVPLFV	L			
0.0041	R	NATWTLSNFC	G			
0.000000026	R	VVTVTLEGLEN	V			
0.0000088	R	LVQLLAHPSPS	T			
0.003	R	LLHSTDEEVLT	I			emPAI
4.4E-10	R	AGVDDLLGILK	N			
0.00000078	R	VISEEFDSIFLEI	R			
0.001	R	FSSSQIDYHEV	C			emPAI
0.000022	M	GIDLIAGGK	S			
8.7E-09	R	APLGQNTVLLI	G			
0.00000034	K	AGGECLTFDQ	A			emPAI
0.0033	K	SFKGEVLYR	T			
0.000027	K	IADVLLLMEK	E			
0.000069	R	ASSSTYSVLLR	V			
0.000054	R	IEATTWDALIK	L			
0.00000026	R	TLLANCVAAGI	K			emPAI
0.0083	K	TLSAPVPDVR	S			
0.00000042	R	TAAYEALNEVV	C			
0.000000015	R	NGILEAYSGIF(	N			
0.00062	K	TAIGVLGDLAD	E			emPAI
2.5E-09	R	SIATLAITTLLK	T			
0.0014	R	SLMTFLSNILR	E			
0.0000019	R	WSNEVQEGIC	S			
0.00023	R	ELTPAITVLQLI	F			emPAI
0.0021	R	GASHHVLDEA	S			
0.000000017	R	MASFVGAMA	S	Oxidation (M)	0.200000000000000.0	
0.0012	R	SLHDALCVLSC	V			
0.0000078	R	ALVAIPTTIADI	A			emPAI
4.4E-13	R	LLVDEAVLQEV	G			
0.000021	K	RLLVDEAVLQE	G			emPAI

0.000000063	K	YLSPLVDATLLI	A			
0.000018	R	MIVVHPQLVP	G			emPAI
0.000032	K	YLSGLGIAR	Q			
0.00011	R	AMNEINAAAF	M			
0.00027	K	LLLDDVFEQK	N			
0.00000026	R	GQIQAYVFDV	A			
0.006	R	DVASQIRDGLI	-			emPAI
0.000000029	K	NPAIIVGAGLF	T			
9.1E-09	R	FASEVAGVQD	G			emPAI
0.00000038	R	MNTIAPNLTA	L			
0.0000028	R	SQFTELISGLGI	Y	Oxidation (M)	0.000000000000000020000000000.0	emPAI
0.004	K	QLGDFFR	T			
0.000016	K	SDTSVFDVFGI	V			
0.000032	R	ALQFNTLPVVI	T			
0.0043	K	TATEPLNPSEL	M			
0.000037	K	SVETTLAELGV	L			
0.0026	R	SIWAFGPDKQ	N			emPAI
0.00000003	K	GVDLDALLDM	L			
0.005	K	GVDLDALLDM	I			emPAI
0.000079	R	MSLLAEAQR	V	Oxidation (M)	0.200000000.0	
0.000000021	K	AGHQTSAESW	A			
0.00016	R	HAIVSAIAATA	G			emPAI
0.00019	R	ALLIDLEPR	V			
0.000000051	K	YISILNIIQGEVI	I			emPAI
0.000076	R	IAGLEVLR	I			
0.0064	R	IPAVQDLVR	K			
4.5E-09	K	QFAAEEISAQV	K			
0.0025	R	QAVVNPENTF	R			
0.00088	R	IINEPTAASLAY	K			emPAI
0.000000047	R	ISADEGVLALW	G			
0.00005	R	NYTNAFHALTI	I			
0.000003	R	MQADNTLPLA	R	Oxidation (M)	0.200000000000.0	emPAI
0.000047	R	GPASDLVPVLF	Q			
0.000004	K	SDLGSSPLAQC	L			
0.00000023	K	NADVILTTLTG	K			emPAI
0.000052	R	AMNEINAASR	M			
0.00041	K	SNSVFIPHGPG	D			
0.00000003	R	NQIQAYVFDV	A			
0.0016	R	NSVLAFSESVP	D			emPAI
0.0025	R	AAADQILR	D			
0.00047	K	WAEIIGQAR	H			
0.00097	K	DLYAEEAAAQ	E			
0.000012	K	LLSEEVFDFSR	G			
0.0000017	R	NIAETGLNLLLI	N			emPAI
0.0069	K	TAYNLIQR	E			
0.0018	R	LLVDLLQFLEPI	N			
0.0005	R	STHPELVTVIEC	I			
0.00000019	K	AIQTIDADIAQ	R			
0.000024	K	IDLLPEIVEAPC	A			emPAI
0.0063	R	LVAIVER	M			
0.0000066	K	LAPSGFVLLR	D			
0.0021	K	FSATAGLGVIH	G			
0.0000014	R	IVSLLSESYNPI	Y			
0.0078	R	QLLHFAVSDVS	T			emPAI
0.00055	R	TVEYIPENSCD	A			
1.1E-10	R	DLFEGILQAGS	T			emPAI
0.0000018	R	SPSVLVLLPTR	E			
0.000058	K	FATLVATNVA	G			

0.0031	K	VQVSELSGLLD	A			
0.00024	K	TLAFVLPILESL	S			
0.00084	R	DLEIPLVVFLK	L			
1.1E-12	R	VLAFANLVYSII	N			emPAI
0.0091	K	TSLRPETVEAL	E			
0.00000046	R	DILSIPVSAAAF	E			
0.0048	K	SIESSDLNFTR	Y			
2.3E-09	K	SLEPFVNWLEI	-			
0.0000086	R	SLYELDVLAED	K			emPAI
0.0055	K	LMGLKELLQAI	C	Oxidation (M)	0.020000000000000.0	
0.000000008	R	NAAGTPLVQII	V			
0.000075	R	LDPLAEAVLLS	L			emPAI
0.000000007	K	QSNILEDLATL	L			
0.0000015	K	RPDQPFPTGQ	W			emPAI
0.00000076	K	GVLQILLDLR	F			
0.00000017	K	WLSDELSAILLI	D			
0.0011	K	SLCESISANISSI	S			emPAI
0.0056	K	LAVLQFYK	V			
0.000000015	K	TITLEVESSDTI	A			
0.000018	R	TLADYNIQKES	L			emPAI
0.0073	R	LRHNVIR	T			
0.0000097	R	IGDLELFR	T			
0.0056	R	LLLGEIPER	S			
0.0023	K	FLDTFAQDR	T			
0.00024	K	ALRPYFELTNA	I			
0.000013	K	ETGDIYSTNEP	I			emPAI
4.9E-09	R	SIVGATLEVIQI	K			
0.000006	R	SDSQVFLFLNS	C			emPAI
0.00015	K	GVVDSDDLPLI	E			
0.0000061	K	NYSQFVGFPIY	S			
0.0000014	R	FLSVTEPSLLGI	I			emPAI
0.0068	R	TNQQFLAR	I			
0.00000041	K	DITTVLDLALR	Q			
0.000000085	R	LVLILGLADEL/	A			emPAI
0.00013	K	AIDEYASLR	S			
0.0021	K	FSATAGLGVIH	G			
0.0000014	R	IVSLLSESYNPH	Y			
0.0078	R	QLLHFAVSDV!	T			emPAI
0.000057	K	VIEVEGPR	G			
0.000046	K	FLDGIYVSEK	S			
0.0000011	R	TALSHVDNLIS	G			
0.0000064	K	GTCVSASNCAI	G			
0.000000008	R	FKGTCVSASN(	G			emPAI
0.00014	K	VTVVPSAAAL\	A			
0.000000025	K	HNGNISFDDV	I			
0.00029	R	GLSVFISDVR	N			
0.0022	K	VSAYILGEYGH	Q			
0.000000092	K	FLQPLQLTSEE	A			
0.0017	R	DLGVLTSSTSLI	C			emPAI
0.000000014	K	ISDVLLLMER	E			
0.000046	R	SLLPLYADIGD!	R			emPAI
0.00000082	R	TASMDFVTEL\	K			
0.0018	K	FIQFVVDIFKR	Y			
0.000067	R	NQILVFVSQVI	V			
0.00031	R	AISTILESISSLP	K			emPAI
0.0062	K	LLGGLAVR	R			
0.0000021	R	GLCAIAQAESL	Y			
0.0000001	R	ELAEDGYSGVI	V			emPAI

0.000000011	K	WLDLATLMVT	I		
0.000017	R	MLNEDELRDA	Q		
0.0013	R	HYFQNTQGLI	V		emPAI
0.002	R	FLALDEADR	M		
2.7E-09	R	TPILVATDVAA	G		
0.0033	R	LAADFLANYIF	V		emPAI
0.0000026	K	SVAAGSATVA	S		
0.000082	K	SGAAPVVSAA	V		
0.00016	R	YGNATVVENT	G		emPAI
0.00013	R	TGNENAQLTP	L		
0.00000001	R	TESAAVSTIVN	E		emPAI
0.000041	R	AAALNIVPTST	A		
0.00000002	K	GILDVCDAPLV	C		emPAI
0.00024	K	ILLENAGAVFR	T		
8.3E-09	R	LLGLDTILNIVV	R		
0.0033	R	APLVVAAIQTL	N		emPAI
0.0012	K	VLAPYSAEDAI	G		
0.00091	R	LALPQIEDIVR	A		
7.6E-09	K	LAEEGISAEVII	S		emPAI
0.0000029	K	TPGPGAQSAL	A		
0.0026	K	VETVTLGPSVF	E		
0.0000024	R	IEDVTPIPTDST	R		emPAI
0.000022	K	ILQLLR	L		
0.000048	K	LLFIIR	I		
0.0000068	R	IALTDNSIVEQ	H		emPAI
0.000000081	K	AIGNFGLSLLR	Q		
0.000037	R	IAAPGISVWNI	G		emPAI
0.0000015	R	QTAPDATYVY	T		
0.0000017	R	NRIPQSPQENI	L		emPAI
0.000000019	K	LAVNTIGSLVR	A		
0.00032	K	SIDAGDYDYAI	I		emPAI
5.3E-09	R	ILSALLTGVNR	A		
0.0016	K	AVVIDEVDSFL	A		emPAI
0.0000031	R	GEGTSTIVNPF	K		
0.00082	K	VIGVGGGGSN	M		
0.00026	K	LLTAVSQSTPV	Q		emPAI
0.0000065	R	LVELANEFSR	S		
0.00063	K	SPCQILVATPG	L		
0.0016	K	TVAFLLPSIEA\	A		
0.0072	K	VLVLDEADHLI	E	Oxidation (M) 0.000000000000020000.0	emPAI
0.00012	R	HNTLLEEQR	H		
0.000014	R	SFSVYNNPSA(	K		
0.00083	R	HIDPLWPSDH	S		emPAI
7.5E-09	R	VVVEVEQEDA	R		
0.0036	K	GIYPTEIYNLSD	L		emPAI
0.00000021	R	SSVSSLQSSIR	R		
0.000072	K	LVSMIENLLER	I		emPAI
0.0000042	R	SLDSHIEDQFA	L		
0.0000095	R	VLDVVYNASN	T		emPAI
0.00016	K	NPVLTSFATGF	A		
0.0000015	K	AVVSSPLGGEI	S		emPAI
0.0013	R	LNILPFK	V		
0.0035	K	VLSMAPGLER	L	Oxidation (M) 0.0002000000.0	
0.00004	R	SQDFLFISGTK	M		
0.0013	K	AGNWLIGGDI	F		emPAI
0.00012	R	VLINAFTVR	A		
0.000000052	K	LLPLLASSLEFG	M		emPAI
0.009	R	YLTVQFVR	F		

0.00031	K	ALFGVNLQSR	L		
0.00000059	K	SALSNYSLAAR	S		emPAI
0.0035	R	ASDLSPEVSSA	Q		
0.000000026	K	SVNIDNAVTLS	A		emPAI
0.000078	K	VAQSITAAIR	N		
0.0000006	K	LISDVELAAER	R		emPAI
0.0022	K	TAEEVAALVR	S		
0.0047	R	AISATNLYLR	V		
0.0022	R	YIQPWESEFID	V		
0.00031	R	TDVIQALGGVI	G		
0.00015	R	EELGLIEQAYD	I		emPAI
0.00061	K	SPMAGTFYR	S	Oxidation (M) 0.002000000.0	
0.0000028	K	QLDCELVIR	K		
0.0065	K	ESSASSPELATI	L		emPAI
0.0000079	K	TVSSAITESFQI	G		
0.00017	R	LQGEIQPQLE\	Y		
0.006	K	AFFNQVIALSE	R		emPAI
0.000000088	R	LASLISLDGILK	Q		
0.00085	R	TGDALQSQDII	A		emPAI
0.00013	K	STYSGFELFR	I		
0.0026	K	VALVQIPSKVE	Q		
0.0000032	R	SGPDTEVFWN	Q		emPAI
0.000011	R	LLILTDPR	T		
0.000013	R	HTPGTFTNQM	L	Oxidation (M) 0.000000000200000000.0	emPAI
0.00084	R	YAQVFQVR	Y		
0.0028	K	VLRPFLLR	R		
0.00074	K	LALDALVIQQC	L		
0.000015	K	LRDYQLAGLN'	L		emPAI
0.00011	K	DLLSVYGYLQF	E		
0.0000027	R	TLLANHVATSI	T		emPAI
0.000086	K	TLAFLIPAVELL	E		
0.000003	K	ALLVLIPEELQF	Y		
0.00077	K	TLAFLIPAVELL	F		emPAI
0.00004	R	VVGVLLGSSSF	G		
0.000055	K	VVVHPLVLLSI'	V		emPAI
0.00033	K	TYGFLTPEFWI	E		
0.0000058	K	VLQFAGIDDVI	G		emPAI
0.000073	K	VLPEMILICQN	N		
0.000032	K	TQTTHAQPDD	S		emPAI
0.00055	K	GTVEIITPVELI	K		
0.00000081	R	VGLVAPIDVV\	I		emPAI
0.00037	K	HNEIQTANIK	S		
0.0000069	K	SIGADYEVTDC	L		emPAI
0.00074	R	YGDFFLR	Q		
0.000015	K	LVDESVNSQLI	D		emPAI
0.0055	K	TGFVFPR	V		
0.0000021	R	ILGEQVQIVR	D		
0.0014	K	LLPLPELLQSIS	A		emPAI
0.00028	K	SFSSGGEDGY\	L		
0.00004	R	LHHFDSDYFNI	-		emPAI
0.000002	K	FLSTLQNIPSVS	S		emPAI
0.0035	K	ILNIER	K		
0.0026	R	TIDGGTHIEGV	A		
0.000034	K	SSLETVLTVLH,	F		emPAI
0.00049	K	SLLFFQGQR	S		
0.000064	R	DDYSHAEPAT'	S		emPAI
0.0039	K	IASLQNEVR	K		
0.0000067	R	AVFGEVYPDP'	V		emPAI

0.0053	R	NLQDAMSVAI	N			
0.00026	K	TAIEAACMLLF	I			
0.00022	R	TLAQNCGVNV	T			emPAI
0.001	R	YFDFASPWEQ	S			
0.000036	R	VVSEPIEMTQI	Y	Oxidation (M)	0.0000000200000.0	emPAI
0.0036	K	NLYIISVK	G			
0.0000055	R	MSLGLPVAAT	N	Oxidation (M)	0.20000000000000000000.0	emPAI
0.000013	K	SSLAASASQVF	S			
0.0076	R	NPAITPQTFPC	L			emPAI
0.002	R	QCLFGGLR	I			
0.000014	R	GLLGTVGTIAR	E			emPAI
0.0087	R	YKVAVDAEEG	R			
0.0041	R	NATWTLSNFC	G			
0.000026	R	LIQLLGHSSPS	T			emPAI
0.000012	K	AGALGDSVTIT	E			
0.0085	K	ITVTADGQFSK	R			emPAI
0.0041	R	NATWTLSNFC	G			
0.000013	R	LVELLGHQSPT	T			emPAI
0.0095	R	IFEGEALLR	R			
0.000014	R	YGLLDESQNKI	R			emPAI
0.000018	K	AGALGDSVSIT	E			
0.0085	K	ITVTADGQFSK	R			emPAI
0.0009	K	IASETGVPVGC	L			
0.00019	K	ATPEVATATPL	K			emPAI
0.00034	K	HIDATLGSGNI	E			
0.0022	K	ELAPLQELIESI	-			emPAI
0.0048	K	VGIALALKP	-			
0.0000069	K	SFFTVSGEVDS	A			emPAI
0.0086	R	TLGSSALEAR	H			
0.00026	R	IFEFLHSPDSGI	I			emPAI
0.0007	K	SLADADYYR	V			
0.00058	R	YAPGIEILSVR	V			emPAI
0.0087	R	LILVESR	I			
0.00046	K	KGLTPSQIGVII	D			
0.0023	K	TTPQDVDESIC	F			emPAI
0.00036	R	SPLVSFLYER	G			
0.0048	R	SGGVFVGTTFI	Y			emPAI
0.0096	K	LTIIEEAR	K			
0.00014	K	SRECLPLVLIIR	N			emPAI
0.0059	R	AMIGQVAGG(	T			
0.0028	R	ASGDYAIVIAH	I			emPAI
0.005	R	DHACVVGGYF	M			
0.0025	R	TNVIPIIEDAR	H			emPAI
0.005	R	AVVDEVLAPR	D			
0.0027	K	LVASADDLSVI	A			emPAI
0.0052	R	YSEVETSTVR	I			
0.0057	K	TMGEILASLLN	-	Oxidation (M)	0.000000000020.0	emPAI
0.0036	R	ANLNDFDR	F			
0.0085	R	LSLTDIVIDINR	K			emPAI
0.0081	K	TQIHVAADDS	H			
0.0096	K	SQQDTFLPVIE	C			emPAI
pep_expect	pep_res_befo	pep_seq	pep_res_after	pep_var_mod	pep_var_mod_pos	
4.40E-06	K	SSVCDIAPK	G			
3.10E-06	R	YLTASAVFR	G			
2.40E-06	K	LAVNLIPFPR	L			
0.00064	R	FPGQLNSDLR	K			
1.40E-06	R	VSEQFTAMFR	R			
8.30E-07	R	IDVYFNEASG(	Y			

2.40E-06	R	MMMTFSVFP	V		
1.20E-05	R	LHFFMVGFAP	G		
9.20E-06	R	AVLMDLEPGT	S		
3.80E-07	K	NSSYFVEWIPN	S		
9.00E-10	R	EILHIQGGQCG	F		
1.80E-12	K	MASTFIGNSTS	R	2 Oxidation (N 0.20000000000000200.0	
6.50E-11	K	GHYTEGAELID	K		
3.60E-06	R	GSQQYSALSVI	N		
3.10E-07	K	LANPTFGDLNI	F	Oxidation (M) 0.00000000000000000200000000.0	
5.50E-06	R	SGPFGQIFRPD	G		
8.90E-08	K	FWEVICDEHG	I		
1.30E-08	K	EAENSDCLQG	I		emPAI
0.0029	R	YLTASAMFR	G		
2.40E-06	K	LAVNLIPFPR	L		
0.00064	R	FPGQLNSDLR	K		
1.40E-06	R	VSEQFTAMFR	R		
1.20E-06	R	YTGDSDLQLEI	I		
6.50E-05	R	MMLTFSVFPS	V		
1.10E-09	R	INVYYNEASCG	F		
2.00E-07	K	EVDEQMLNVC	N		
1.20E-05	R	LHFFMVGFAP	G		
9.20E-06	R	AVLMDLEPGT	S		
3.80E-07	K	NSSYFVEWIPN	S		
9.00E-10	R	EILHIQGGQCG	F		
1.80E-12	K	MASTFIGNSTS	R	2 Oxidation (N 0.20000000000000200.0	
6.50E-11	K	GHYTEGAELID	K		
1.80E-08	R	SGPYGQTFRPI	G		emPAI
1.80E-07	K	DAGVIAGLNV	I		
2.00E-05	R	FTDSSVQSDIK	L		
3.20E-05	R	VEIIANDQGNI	T		
0.0018	R	MVNHFVQEFI	R		
9.70E-07	R	FEELNIDLFR	K		
6.50E-06	R	EIAEAYLGTTIK	N		
2.50E-06	K	NALENYAYNM	N		
5.30E-05	K	ELESICNPIIAK	M		
8.50E-08	K	VQQLLVDFFN	E		
9.30E-09	K	NSIDDVVLVGG	I		
4.70E-07	R	TTPSYVAFTDS	L		
0.00053	R	ARFEELNIDLFI	K		
2.50E-06	K	EFSAEEISSMIL	M		
1.40E-09	K	NQVAMNPINT	R		
7.20E-09	K	ATAGDTHLGG	M		
0.00052	K	NAVVTVPAYF	Q		
1.60E-14	K	SINPDEAVAYG	V		
0.0053	K	GEGPAIGIDLG	V		
3.60E-07	K	EQVFSTYSDNG	A		emPAI
0.0029	R	YLTASAMFR	G		
2.40E-06	K	LAVNLIPFPR	L		
0.00064	R	FPGQLNSDLR	K		
1.40E-06	R	VSEQFTAMFR	R		
6.50E-05	R	MMLTFSVFPS	V		
7.60E-08	R	VNVYYNEASC	F		
2.00E-07	K	EVDEQMINVC	N		
1.20E-05	R	LHFFMVGFAP	G		
9.30E-05	R	AVLMDLEPGT	S		
3.80E-07	K	NSSYFVEWIPN	S		
9.00E-10	R	EILHIQGGQCG	F		
1.80E-12	K	MASTFIGNSTS	R	2 Oxidation (N 0.20000000000000200.0	

6.50E-11	K	GHYTEGAELID	K			
1.10E-05	R	SGPYGQIFRPD	G			emPAI
1.80E-07	K	DAGVIAGLNV	I			
3.20E-05	R	VEIIANDQGNI	T			
0.0001	R	FSDSSVQSDM	L			
0.0018	R	MVNHFVQEFI	R			
1.90E-06	R	FEELNMDLFR	K			
2.50E-06	K	NALENYAYNM	N			
5.30E-05	K	ELESICNPIIAK	M			
6.60E-05	K	STVHDVVLVG	I			
0.00011	K	VQQLLQDFFN	E			
4.70E-07	R	TTPSYVAFTDS	L			
9.20E-05	R	ARFEELNMDL	K			
3.10E-08	K	EFAAEEISSMV	M			
3.20E-09	K	NQVAMNPVN	R	Oxidation (M)	0.000020000000000.0	
7.20E-09	K	ATAGDTHLGG	M			
0.00052	K	NAVVTVPAYF	Q			
9.20E-13	K	SINPDEAVAY(	V			
0.0053	K	GEGPAIGIDLG	V			
3.60E-07	K	EQVFSTYSDN(	A			emPAI
0.0029	R	YLTASAMFR	G			
2.40E-06	K	LAVNLIPFPR	L			
0.00064	R	FPGQLNSDLR	K			
1.40E-06	R	VSEQFTAMFR	R			
0.00069	R	YVGDSELQLEI	V			
2.40E-06	R	MMMTFSVFP	V			
7.60E-08	R	VNVYYNEASC	Y			
2.00E-07	K	EVDEQMLNV(	N			
1.20E-05	R	LHFFMVGFAP	G			
9.30E-05	R	AVLMDLEPGT	S			
3.80E-07	K	NSSYFVEWIPI	S			
1.80E-12	K	MASTFIGNSTS	R	2 Oxidation (I	0.20000000000000200.0	
6.50E-11	K	GHYTEGAELID	K			
1.10E-05	R	SGPYGQIFRPD	G			emPAI
6.10E-06	K	DVNAAVGTIK	T			
2.40E-05	K	YMACCLMYR	G			
0.0044	R	QLFHPEQLISG	E			
1.20E-09	R	LVSQVISSLTAS	F			
9.60E-07	R	TIQFVDWCPT	C			
0.0003	R	SLSIERPTYTNI	L			
1.30E-06	R	AVFVDLEPTVI	T			
5.30E-10	R	IHFMLSSYAPV	A			
2.50E-10	R	AVCMISNSTS\	I			
7.40E-08	K	CGINYQPPTV\	V			
1.30E-09	K	TVGGGDDAFI	H			
3.50E-06	R	AFVHWYVGE(	E			
1.90E-09	R	FDGALNVDVT	I			
0.00062	K	AFHEQLSVAEI	C	Oxidation (M)	0.000000000000000000000200.0	emPAI
0.00038	R	ALGDYLGVK	V			
0.00029	R	ELAQQIEK	V			
3.30E-05	K	GVAINFVTR	D			
0.0065	R	DELTLEGIK	Q			
2.10E-07	R	VLITTDLLAR	G			
5.20E-05	K	VHACVGGTSV	E			
0.0034	K	RDELTLEGIK	Q			
3.10E-10	K	GLDVIQQAQS	T			
1.10E-06	R	ILQAGVHVVV	V			
1.10E-08	K	MFVLDEADEI	G	Oxidation (M)	0.2000000000000.0	

7.30E-08	R	GIYAYGFEKPS	G		
1.40E-06	K	FYNVVVEELPS	-		
1.20E-07	R	SRDHTVSATH	D		
5.00E-12	K	IQVGVFSATM	K		
0.00029	R	ELAQQIEK	V		
0.0065	R	DELTLEGIK	Q		
2.10E-07	R	VLITTDLLAR	G		
5.20E-05	K	VHACVGGTSV	E		
0.0034	K	RDELTLEGIK	Q		
3.10E-10	K	GLDVIQQAQS	T		
3.60E-06	K	GVAINFVTLDI	M		
1.10E-06	R	ILQAGVHVVV	V		
1.10E-08	K	MFVLDEADEN	G	Oxidation (M) 0.2000000000000.0	
7.30E-08	R	GIYAYGFEKPS	G		
1.40E-06	K	FYNVVVEELPS	-		
1.20E-07	R	SRDHTVSATH	D		
5.00E-12	K	IQVGVFSATM	K		
3.20E-07	K	DAGVIAGLNV	I		
3.20E-05	R	VEIIANDQGNI	T		
0.0018	R	MVNHFVQEFI	R		
2.50E-06	K	NALENYAYNM	N		
0.00011	K	VQQLLQDFFN	E		
4.70E-07	R	TTPSYVAFTDS	L		
3.20E-09	K	NQVAMNPVN	R	Oxidation (M) 0.000020000000000.0	
7.20E-09	K	ATAGDTHLGG	M		
0.00052	K	NAVVTVPAYF	Q		
1.60E-14	K	SINPDEAVAY(	V		
0.0053	K	GEGPAIGIDLG	V		emPAI
3.20E-05	R	VEIIANDQGNI	T		
0.00024	K	DAGVISGLNVI	I		
1.20E-05	R	EIAEAFLGSPVI	N		
0.0018	R	MVNHFVQEFI	R		
1.90E-06	R	FEELNMDLFR	K		
2.50E-06	K	NALENYAYNM	N		
0.00011	K	VQQLLQDFFN	E		
4.70E-07	R	TTPSYVAFTDS	L		
9.20E-05	R	ARFEELNMDL	K		
7.20E-09	K	ATAGDTHLGG	M		
0.00052	K	NAVVTVPAYF	Q		
1.60E-14	K	SINPDEAVAY(	V		
0.0053	K	GEGPAIGIDLG	V		emPAI
0.0078	R	INAEDPFK	G		
0.0038	K	LIVWAPTR	E		
0.00065	K	LILDVEDFK	N		
5.50E-05	K	FGNVVHFGER	D		
5.90E-05	R	YKQEDIVLR	G		
6.50E-05	R	VANEIGFPVM	A		
1.10E-07	R	GSFYFMEMNT	I		
0.0026	R	ITSYLPSGGPF	M		
4.20E-06	K	LLEEAPSPALT,	K		
2.60E-06	R	DHGINFIGPNI	V		
2.10E-11	K	SEAAAAFGND	F		
3.30E-11	K	HEEELAEPQEI	D		
1.50E-05	R	ALNDTIITGVP	L		
2.30E-08	K	NAGVPTVPGS	V		
1.10E-05	R	IQVEHPVTEM	V	Oxidation (M) 0.000000000200000000000.0	emPAI
0.0029	R	YLTASAMFR	G		
2.40E-06	K	LAVNLIPFPR	L		

0.00064	R	FPGQLNSDLR	K			
1.40E-06	R	VSEQFTAMFR	R			
6.50E-05	R	MMLTFSVFPS	V			
7.60E-08	R	VNVYYNEASC	Y			
2.00E-07	K	EVDEQMINVC	N			
1.20E-05	R	LHFFMVGFAP	G			
3.80E-07	K	NSSYFVEWIPI	S			
0.00054	K	GHYTEGAELIC	K			emPAI
6.00E-08	R	SGAVASAVMI	K			
0.0062	K	ISWVPDPK	T			
6.70E-12	R	GYAATAAQGS	S			
5.00E-12	R	PETGSNEIDAA	A			
1.40E-07	K	TGYYRPETGSI	A	Methyl (DE)	0.0000001000000000000.0	
6.10E-06	K	DVNAAVGTIK	T			
2.40E-05	K	YMACCLMYR	G			
0.0044	R	QLFHPEQLISG	E			
1.30E-11	R	LISQIISSLTTSL	F			
1.20E-06	R	TVQFVDWCPT	C			
1.30E-06	R	AVFVDLEPTVI	T			
7.10E-11	R	AVCMISNNTA	I			
7.40E-08	K	CGINYQPPTV\	V			
3.50E-06	R	AFVHWYVGE(	E			
0.0034	R	IGINGFGR	I			
9.10E-10	R	VPTVDVSVVD	L			
2.00E-05	K	TLLFGEKPVTV	N			
1.90E-06	K	DAPMFVVGVI	S			
3.40E-07	K	LVSWYDNEW	V			
2.30E-06	R	FGIVEGLMTT\	T			
6.20E-12	K	GILGYTEDDV\	S			
6.10E-12	K	SDLDIVSNASC	V			emPAI
0.0063	K	EHILLAK	Q			
0.00062	K	FEAIIYVLK	K			
2.30E-05	K	TVGAGVIGTIL	-			
3.50E-07	K	VGETVDLVGLI	E			
1.70E-06	R	SYTVTGVEMF	I			
1.70E-06	K	KYDEIDAAPEE	A			
2.70E-09	K	ILDEALAGDN\	G			
2.30E-06	R	HYAHVDCPGI	N			
0.00033	K	IVVELIVPVACI	F			
9.50E-08	K	TTLTAALTMAI	K	Oxidation (M)	0.00000000200000000000.0	
2.60E-07	K	EDQVDDAELL	E			emPAI
0.0026	R	QTVAVGVIK	S			
0.003	K	ARYDEIIK	E			
9.90E-07	K	IGGIGTVPVGI	V			
0.0012	R	STNLDWYK	G			
0.00015	K	STTTGHLIYK	L			
0.0047	R	FEKEAAEMNK	R	Methyl (DE)	0.0001000000.0	
2.40E-08	R	EHALLAFTLGV	Q			
5.70E-06	K	YYCTVIDAPGI	D			
1.70E-05	K	FHINIVVIGHVI	S			
0.00055	K	MTPTKPMVVI	F	2 Oxidation (I	0.20000020000000000000.0	
3.70E-05	R	VETGMIKPGI	S			
1.10E-05	K	NMITGTSQAC	D			
0.0098	R	VPPEVMESIR	K			
4.10E-07	R	HENVDIVVIR	E			
0.0002	R	HLQFPSFADR	L			
4.10E-07	K	LADGLFLESCR	E			
1.40E-06	K	YAFEYAYLNNI	K			

3.80E-08	K	TPVGGGVSSLI	K			
7.30E-05	K	ANPVALLLSSA	H	Oxidation (M)	0.000000000002000.0	
7.00E-10	K	DLGGTSTTQE	L			emPAI
0.00032	K	TDGLFLK	C			
0.0083	R	SLNLTLR	K			
0.00024	R	YDDVDLITIR	E			
6.00E-07	K	TRYDDVDLITII	E			
1.10E-11	R	TADLGGSSTT	A			
9.10E-08	R	TQSFLTWESLE	R			
2.00E-07	K	NLANPTALLLS	H			
3.70E-07	R	ENTEGEYSGLE	G			emPAI
0.0062	K	VQQITIR	S			
0.00026	K	EIEDAVADLR	S			
3.20E-05	K	VQSIVAEIFGK	S			
1.50E-05	R	SSGGLSEDDIC	M			
1.40E-05	R	QAVTNPTNTV	R			
1.00E-09	K	EVDEVLLVGGI	V			
0.0048	K	AVVTVPAYFN	Q			
4.90E-06	R	SRFETLVNHLII	T			
9.60E-11	K	SQVFSTAADN	V			emPAI
0.001	R	SDNIDLTK	D			
5.60E-06	K	VQEIVSEIFGK	S			
8.80E-06	R	SSGGLSDDEIN	M			
6.30E-05	R	TAMAGEDVEI	A	Oxidation (M)	0.002000000000.0	
9.00E-06	R	NSADTTIYSVE	S			
0.00068	K	FSPSQIGANVL	M			
1.00E-09	K	EVDEVLLVGGI	V			
0.0048	K	AVVTVPAYFN	Q			
9.30E-10	K	IPAEIASEIETA	T			emPAI
0.0083	R	SLNLTLR	K			
0.00024	R	YDDVDLITIR	E			
6.00E-07	K	TRYDDVDLITII	E			
1.10E-11	R	TADLGGSSTT	A	Methyl (DE)	0.000000000001000.0	
1.50E-05	K	TDGLFLQCCDI	Y			
7.50E-07	R	TNSFLTWDNL	N			
9.30E-05	K	ATLFPGDGIGF	Q			emPAI
0.00027	K	VGAFSVLR	E			
2.00E-07	K	ALSSPVLAAVC	Y			
3.60E-06	R	TNIQMIGALCI	A			
1.10E-05	R	AELPSCLPVLV	M			
4.70E-07	K	MDVFNTFIDLI	Q			
0.00011	R	EYSLQALESFLI	C			
2.40E-09	R	LSSIILQQLDD\	C			emPAI
0.00019	K	VPLILGIWGGK	G			
6.90E-09	R	GLAYDTSDDQ	G			
3.30E-11	R	VPIICTGNDFS	D			
3.50E-13	R	VQLAETYLSQ/	G			emPAI
5.60E-07	R	HNTLLEEQR	H			
0.00032	R	NNSLLSGIIDG(	S			
0.0034	R	QELFEQGLPFE	S			
4.70E-10	R	SFSVYNNPSA(	K			
1.40E-05	R	HIDPLWPSDH	S			
1.80E-05	R	SFLDFSTQDEE	A			
2.10E-06	K	TESAHAHISN(	L			emPAI
2.70E-05	K	AGFAGDDAPF	A			
0.0066	R	HTGVMVGM(	D			
5.70E-05	R	AVFPSIVGRPR	H			
5.30E-07	K	SEYDESGPSIVI	K			

0.0079	K	IWHHTFYNELI	V			
6.80E-11	K	LAYVALDYEQI	S			
0.0046	K	DLYGNIVLSGC	M			
3.50E-07	R	TTGIVLDSGDC	L			emPAI
0.0038	K	STVVNTHLMP	S	Oxidation (M)	0.00000000200.0	
1.30E-10	K	AADQGLVILQ	R			
3.70E-10	R	EDGEPVGYND	K			
7.80E-10	K	VGEEVEILGLR	E			
7.30E-08	R	GSALSALQGTI	Q			
2.30E-06	R	HYAHVDCPGH	N			
0.00024	K	VDVVDDPELLI	E	Oxidation (M)	0.000000000000002000.0	
0.0014	R	VLDKPFLMPIE	G			emPAI
0.0037	K	LVSGLIPDAGT	A			
2.20E-05	K	IPLTLIYDDIK	S			
1.10E-05	K	DFGSALWDM	G	Oxidation (M)	0.00000000200.0	
2.30E-06	K	FSLEETVAILH\	L			
8.90E-05	K	STYNDINPGM	I	Oxidation (M)	0.000000000200000.0	
0.00044	K	NPNPVPIPLID	K			
1.60E-05	R	QAVDISPLR	R			
0.0015	K	HATFVPHTAG	Y			
8.30E-09	R	VNQAIFLLTTG	E			
2.30E-09	K	TIAECLADELIN	G			
4.10E-07	R	HENVDIVVIR	E			
0.0002	R	HLQFPSFADR	L			
1.40E-06	K	YAFEYAYLNNI	K			
9.10E-09	K	TPVGGGVSSLI	K			
0.0017	K	NANPVALLLSS	H			
0.00014	R	TEDLGGNSTT(	-			emPAI
0.00055	R	LWGENFFDPA	K			
6.20E-09	R	PMEEGLAEAII	I			
1.50E-06	K	AYLPVVESFGF	A			
5.20E-05	R	YRVENLYEGPI	N			emPAI
0.00046	K	AICTEAGLLALI	E			
2.80E-08	R	TMLELLNQLD	G			
2.60E-09	K	APLESYADIGG	E			
0.0018	R	VADDLSPSIVF	R			emPAI
0.0045	K	AEIANVLSR	G			
0.0012	K	TAIAEGLAQR	I			
3.20E-05	R	HAQLPEEAR	E			
2.90E-07	K	AIDLIDEAGSR	V			
2.50E-05	R	VLENLGADPSI	T			
0.0012	R	GSGFVAVEIPF	A			
2.70E-06	K	VPEPTVEEAIQ	E			
0.0076	R	LGHNFVGTEQ	V			emPAI
9.80E-05	R	ALLIDLEPR	V			
0.0016	R	VINGIQNGDYI	N			
8.40E-06	R	DIIESLVDEYK	A			
5.70E-05	K	GVEEEIMDMI	E			
5.50E-06	K	YISILNIIQGEVI	I			
0.00042	R	LHLTNPTFAQT	Y			emPAI
0.00066	R	SLQLEATTQFR	K			
7.70E-05	R	NATWTLSNFC	G			
2.80E-08	R	EQAVWALGN	C			
1.30E-06	K	VTGNTGDVNI	I			
0.0033	K	VVIEHGAVPIF	E			emPAI
1.20E-05	R	VDEIITCAPR	R			
1.10E-05	K	DSFLDEGFILDI	K			
4.10E-10	K	VQDDEVGDG	E			

0.0037	R	ALVAIPTTIADI	A			emPAI
2.70E-05	R	LLILTDPR	T			
9.30E-10	R	VIVAIENPQDII	P			
0.00041	R	HTPGTFTNQM	L	Oxidation (M)	0.000000000200000000.0	
8.10E-05	K	EGALGNIPIIAF	F			emPAI
4.60E-05	K	DAGVIAGLNV.	I			
0.0023	R	FEELNNDLFR	K			
5.70E-06	R	ITPSWVGFTDS	L			
0.0036	K	VFSPEEISAMII	M			
3.60E-08	R	VEIESLFDGVD	A			emPAI
1.60E-05	K	GAGANILR	A			
1.20E-05	K	LQLIVFGK	K			
0.0079	K	DEGFGSLWR	G			
0.00039	K	LLIQNQDEMIk	A	Oxidation (M)	0.00000000200.0	
0.0041	R	TIKDEGFGSLW	G			
0.00083	R	YFPTQALNFAF	D			
2.70E-05	K	GFTNFALDFLN	T	Oxidation (M)	0.00000000002000000000.0	emPAI
2.70E-05	K	AGFAGDDAPF	A			
0.0021	R	GYSFTTTAER	E			
0.0066	R	HTGVMVGMC	D			
5.70E-05	R	AVFPSIVGRPR	H			
0.0079	K	IWHHTFYNELI	V			
0.00023	K	NYELPDGQVIT	F			
3.50E-07	R	TTGIVLDSGDC	L			emPAI
1.60E-05	K	GAGANILR	A			
1.20E-05	K	LQLIVFGK	K			
0.0016	R	DEGIGSLWR	G			
4.70E-05	K	SSFDAFSQIVK	K			
0.00039	K	LLIQNQDEML	A	Oxidation (M)	0.00000000200.0	
0.0011	R	TIRDEGIGSLW	G			
0.00083	R	YFPTQALNFAF	D			emPAI
2.50E-06	R	SSGFGDFGSDI	S			
0.0041	R	SSFGGFGSND(	R			
0.00053	K	TLAFGIPIIDK	I			
0.0053	R	FTELPSIAVER	G			
0.0018	K	CEALHGDISQS	E			
3.70E-07	K	LADGITTYSIIA	A			emPAI
0.0066	R	HGDLLR	T			
1.60E-06	R	AQEFVCGLAQ	I			
2.20E-07	K	LLEIDPNGAVS	K			
0.0001	R	TVQHLIGSGM	A			emPAI
4.60E-05	K	DAGVIAGLNV.	I			
0.0023	R	FEELNNDLFR	K			
5.70E-06	R	ITPSWVGFTDS	L			
0.0036	K	VFSPEEISAMII	M			
2.30E-07	R	VEIESLFDGVD	A			emPAI
6.20E-10	K	SSTTTISDGSSI	K			
3.60E-05	K	EGQVIGYLHQ	L			
0.00026	K	LLSDDGDSVG'	-			emPAI
0.0076	K	IGLFGGAGVGI	T			
1.30E-05	R	VGLTGLTVAEY	D			
8.60E-06	K	TVLIMELINNV	A			
1.10E-07	R	FTQANSEVSAI	I			emPAI
0.00084	K	TLFNELEVVEG	L			
5.30E-13	K	TPVYTIASNAG	L			emPAI
0.00023	K	TAAYGHFGR	D			
6.40E-07	K	ANVDYEQIVR	K			
1.40E-07	K	TNMVMVFGE	A			emPAI

0.0036	K	GVLLYGPPGT(	T			
2.00E-06	K	HGEIDYEAIVK	L			
0.0011	R	AIASNIDANFL	V			
0.00061	R	TLMELLNQLD	V	Oxidation (M)	0.00200000000000000.0	
1.30E-05	R	EVDPVVYNMI	E			emPAI
3.00E-06	K	YSQVVSNALD	L			
1.30E-09	R	AGELSAAEIDN	Q			emPAI
3.20E-10	R	SPSSDLLSILQ(	S			
8.30E-06	R	FGDPSYGQLQ	L			emPAI
0.00011	R	IAGLEVLR	I			
0.007	R	TPVENSLR	D			
0.0015	R	IPAVQDLVR	K			
3.30E-08	K	QFAAEEISAQ\	K			
4.00E-05	R	QAVVNPENTF	R			emPAI
0.0036	K	GVLLYGPPGT(	T			
0.0037	K	LAGPQLVQMI	L			
0.0012	K	DSYLILDTLPSE	V			
1.20E-10	R	TMLELLNQLD	I	Methyl (DE)	0.00000000000000010.0	emPAI
0.00017	K	ADLVNNLGTI/	S			
0.00056	R	ELISNSSDALDI	I			
7.10E-08	K	GIVDSEDLPLN	E			
0.0013	K	SGDELTSLKDY	M			emPAI
8.60E-07	R	GLCAIAQAESL	Y			
5.80E-07	R	ELAEDGYSGVI	V			emPAI
5.60E-07	R	LSGLVNNLISI(	E			
1.60E-08	R	LASFQNAPSVI	Q			emPAI
0.00077	R	ESAIAQIIR	T			
0.00036	R	TCGPLLDVVAI	E			
2.20E-09	K	VPYGQGAVEL	E			emPAI
0.0015	K	VIEVEGPR	G			
0.00014	K	FLDGIYVSEK	S			
5.30E-06	R	TALSHVDNLIS	G			
0.00025	R	FVYAHFPINAS	S			emPAI
0.00032	R	EQLPLFIIANW	G			
0.0006	K	SLQFLPSIINAS	E			
7.40E-05	R	TVEYIPENSCD	A			
0.00012	R	EDAFFLAVTEL	K			emPAI
1.40E-08	R	SIVGATLEVIQI	K			
1.20E-05	R	SDSQVFLFLNS	C			emPAI
1.60E-08	R	TGGGGLDLSSI	R			
0.00014	K	AFEVLNSLEVV	L			
0.0022	K	TLVMLEDFLNI	E	Oxidation (M)	0.0002000000000000.0	emPAI
0.0036	K	GVLLYGPPGT(	T			
0.0055	K	AVCTEAGMF/	E			
0.00021	R	IDILDQALLRP(	I			
4.80E-07	R	TMLELLNQLD	I			emPAI
3.50E-06	K	SGFEAATLIGLI	L			
1.50E-06	K	VSSDFVDAALI	C			emPAI
0.00011	K	SLLEEYFSIR	L			
7.30E-08	K	APNSFGEFLGE	V			emPAI
2.40E-07	K	AGALGDSVTIT	E			
5.10E-05	K	ITVTADGQFSK	R			emPAI
5.00E-08	K	SANGEDTQVN	F			
0.001	R	NFFLFTPLLPS\	S			emPAI
2.90E-07	K	EIPSLQLINQTI	E			
0.00029	R	QTAPDATYVY	T			emPAI
1.50E-05	K	EYVEALALLSTI	E			
0.00048	R	HLSSLYDTLLE(	L			

8.20E-05	R	VEIAHIAELIGL	K			emPAI
1.70E-05	R	AGQYGTVTSL	D			
7.50E-06	K	THGYLAPIIDQ	E			emPAI
3.60E-06	K	TPGPGAQSAL	A			
9.50E-06	R	IEDVTPIPTDS	R			emPAI
5.80E-05	R	APLGQNTVLLI	G			
3.70E-07	K	IAVLVGTITDD	V			emPAI
2.60E-05	R	FLPLGLGLLYL	Q			
4.80E-05	R	AVPLALGLLCI	V			
0.0083	R	QNLAATFVNA	L			emPAI
2.10E-05	R	DLVGVLEDAIF	G			
3.30E-06	K	VVAAGANPVL	G			emPAI
0.0039	K	SFNAGQITSK	L			
4.40E-07	R	EGLVSDAIESF	A			emPAI
5.00E-05	R	GGLVSILNLGL	R			
1.00E-05	R	DASYTTQPLEL	K			emPAI
4.60E-05	R	TCYAQSSQIR	Q			
1.20E-05	K	ATQGIYPLQN	K			emPAI
6.60E-05	K	ILQLLR	L			
8.80E-05	K	LLFIIR	I			
0.0003	K	EANNFLWPFC	A			emPAI
4.00E-05	K	SVAAGSATVA	S			
9.30E-05	R	YGNATVVENT	G			emPAI
1.70E-06	K	LAATCATAVE	V			
0.0013	K	TVLIPIAHGTE	R			emPAI
0.0054	K	EAVDQCITANI	M			
5.00E-05	R	SYAELLQDLMI	T			
0.00049	K	GTSSILSEVFLH	T			emPAI
0.0008	K	DLPAVMTFLIS	A			
1.60E-05	K	SGPLPVDTFTF	I			emPAI
0.0097	K	VLSMAPGLER	L	Oxidation (M)	0.0002000000.0	
3.40E-06	R	SQDFLFISGTK	M			emPAI
0.00083	K	AGALGDSVSIT	E			
5.10E-05	K	ITVTADGQFSK	R			emPAI
0.0033	R	DIVELQLK	Q			
0.00056	K	SPMAGTFYR	S	Oxidation (M)	0.002000000.0	
0.0025	K	QLDCELVIR	K			emPAI
0.0024	K	WAEIIGQAR	Q			
4.50E-05	K	FFALQVLEGVI	Y			emPAI
0.0063	R	ALAESAQDAF	L			
0.00026	R	NQIQAYVFDV	A			emPAI
0.0036	R	GVLLYGPPGT	T			
0.00026	K	ENAPAIIFIDEV	F			emPAI
0.0099	R	EANPYYSTAR	L			
0.0021	R	AYSPDFMFIW	I			emPAI
0.0069	K	GIPYLNTYDGF	T			
0.0013	K	SRECLPLVLIIR	N			emPAI

7.97

7.3

6.54

6.08

2.45

3.72

3.85

3.56

1.79

2.66

2.7

1.07

1.81

1.98

0.72

1.67

0.37

1.25

1.38

1.15

0.59

11.16

1.5

1.51

1.03

0.83

0.81

0.93

0.23

0.52

0.84

0.57
0.26

0.37

0.66

0.24

0.77

0.13

0.17

0.55

0.36

0.18

0.27

0.46

0.09

0.68

0.45

0.27

0.14

0.4

0.09
0.2

0.13

0.55

1.25

0.41

0.17

0.11

0.33
0.03

0.23

0.45
0.12

0.4

0.21

0.31

0.96

0.05

8.95

6.97

6.51

6.85

5.98

5.98

5.11

5.55

2.31

0.13

1.79

0.21

2.92

0.21

2.53

0.68

2.24

3.28

0.98

3.03

2.61

2.2

1.96

0.82

1.81

1.59

1.76

2.18

2.8

2.81

0.74

1.46

1.21

0.23

0.5

0.47

0.97

0.61

1.07

0.65

0.52

0.51

1.68

1.25

8.22

0.32

0.85

1.85

0.86

0.35

0.29

0.51

0.44

0.52

0.61

0.95

2.17

1.1

0.6

1.39

0.34

2.86

0.44

0.58

0.47

1.21

0.22

0.78

0.52

0.51

0.33

0.38

0.79

0.55

0.79

1.17

0.67

0.14

0.16

0.29

0.15

0.46

0.61

0.36

0.7

0.07

0.49

0.58

0.55

0.27

0.61

0.59

0.31

0.69

0.42

0.3

0.23

0.39

0.5

0.2

0.05

0.82

0.37

0.19

0.27

0.22

0.15

0.17

0.23

0.06

1.11
0.39

0.1

0.7

0.6

0.28

0.08

0.07
0.1

0.42

0.38

0.34

0.54

0.12

0.46

0.05

0.49

0.25

0.54

0.13

0.53
0.83

0.16

0.26

0.06

0.32

0.26

0.14

0.27

0.41

0.94

0.15
0.22

0.31

0.72

0.47

0.06

0.05

0.1

0.03
0.07

0.18
0.03

0.16
0.02

12.46

10.29

12.59

11.81

11.88

6.42

6.42

4.73

4.75

4.8

3.1

1.93

4.28

2.6

4.06

2.04

0.76

1.29

0.81

2.84

1.16

1.81

1.46

0.09

0.14

1.58

0.9

0.48

0.9

0.72

1.62

0.47

1.31

1.9

0.58

0.96

0.38

0.44

0.45

0.85

1.06

1.31

2.23

0.96

1.02

15.06

0.15

0.78

0.09

0.19

1.41

0.39

0.5

0.45

0.34

0.67

0.6

0.51

0.28

0.39

0.66

0.46

0.2

0.36

0.23

0.33

0.58

0.61

0.26

0.14

0.96

0.29

0.54

0.91

0.12

0.26

0.27

0.19

1.07

0.11

0.23

0.17

0.23

0.49

0.13

0.14

1.91

0.19

0.18

0.31

0.1

0.6

0.29

0.13

0.12

4.77

3.4

2.17

3.42

2.45

3.4

1.98

2.21

2

0.98

1.35

1.68

1.44

11.16

1.07

1.27

1.42

1.83

1.31

0.94

0.52

0.52

0.81

0.2

0.26

0.14

0.78

0.5

0.45

0.95

1.17

0.58

0.18

0.36

0.3

0.41

0.29

0.3

0.45

0.23

0.67

0.32

0.1
0.03
