# Supplementary material for: Late Embryogenesis Abundant (LEA)5 Regulates Translation in Mitochondria and Chloroplasts to Enhance Growth and Stress Tolerance
Source: Front Plant Sci. 2022 Jun 16;13:875799. doi: 10.3389/fpls.2022.875799 (PMC9244843; doi:10.3389/fpls.2022.875799)
Supplement: Supplementary file 2 [file Table_2.pdf]

Supplemental Table S2. Sequences used for PCR analysis

Oligo	Forward sequence	Reverse sequence
RH22-attB (Cloning)	5’-GGGGACAAGTTTGTACAAA AAAGCAGGCTCCACCATGATTC TCTCACGCTCTGTCTCC-3’	5’- GGGGACCACTTTGTA CAAGAAAGCTGGGTC ATA TCTCACAGCTTGAGGCTCCTC-3’
LEA5-attB1 (Cloning)	5’- GGGGACAAGTTTGTACAAA AAAGCAGGCTCCACCATGGCTC GTTCTATCTCTAACG-3'	5’- GGGGACCACTTTGTACAAGAAA GCTGGGTCCTGCTTGTTGTTCAAGA GAGCTGC -3’
23S-4.5S probe (Northern)	5’-TTCAGAACGTCGTGAGACA GTTCGGTC-3'	5’-CAAATCGTTCGTTCGTTAGGATGCCTC-3'
LEA5 (qPCR)	5’-GTTTCGAGCGGTGGAAGAAGT-3’	5’- GTAGCTCAGCCGCGTCAATCT-3’
Actin 11 (qPCR)	5’- CGACAATGGAACCGGAATG-3’	5’-CCCTTGGCGCATCATCTC-3’
